# Photoactive Iminobismuthanes for Catalytic C–H Amination

**DOI:** 10.1021/jacs.6c04805

**Published:** 2026-05-05

**Authors:** Takuya Tsuruta, Hye Won Moon, Markus Leutzsch, Benedict A. Williams, Davide Spinnato, Raquel Maray-Antolín, Aaron Ullman, Josep Cornella

**Affiliations:** Max-Planck-Institut für Kohlenforschung, Kaiser-Wilhelm-Platz 1, Mülheim an der Ruhr 45470, Germany

## Abstract

Metal-nitrenoids are employed in the direct amination of ubiquitous carbon–hydrogen bonds. Traditionally, their synthetic utility has relied on the redox flexibility and the coordinative reactivity of the metal center, conferred by the *d*-orbitals. Here, we demonstrate that bismuth—a main-group element lacking accessible frontier *d*-orbitals—can nevertheless exhibit catalytic activity analogous to that of transition-metal systems. When an iminobismuthane is subjected to light irradiation, a ligand-to-ligand charge transfer ensues, leading to C–H bond abstraction and C–N bond formation. The unique electronic properties of bismuth permit complementary reactivity beyond that of transition metals, as exemplified by the azidation of unprotected secondary amines. This work reveals main-group imidos as catalytically competent species, rather than merely precursors in transition-metal catalysis.

## Graphical abstract

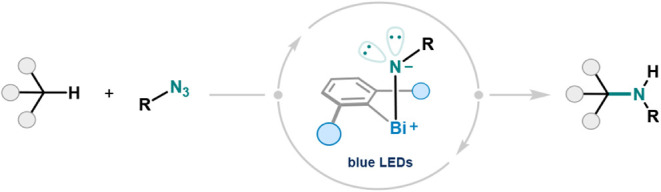

## Introduction

Transition-metal-catalyzed direct C­(sp^3^)–H functionalization has become an indispensable tool for the construction of chemical bonds, which avoids the preactivation of substrates and shortens synthetic sequences.^1,2^ Among the various bond formations targeted with this strategy, the construction of C–N bonds has become prevalent, since the resulting amine products are one of the most relevant moieties in biologically active compounds.^3–5^ To this end, many distinct catalytic strategies have appeared in the literature; yet, catalytic C–H amination via metal-nitrenoid intermediates (M–NR) remains among the most successful approaches due to their broad reactivity and controlled selectivity (Figure 1A).^3,6,7^ In this regard, various transition-metal-based strategies have been studied, where metal-nitrenoid intermediates have been exploited in intra- and intermolecular C–H aminations. For example, polar reactivity employing singlet reactive intermediates has been invoked, where the metal-nitrenoid leads to a concerted mechanism of C–H insertion.^3^ Alternatively, open-shell metal-nitrenoids may be employed, where the reactivity is dictated by hydrogen atom transfer (HAT) processes enabled by an *N*-centered radical.^8^ Regardless of the mechanism, the success of these strategies hinges on the involvement of frontier *d*-orbitals, as the reactivity is governed by their energetic disposition in the transition-metal complexes.

**Figure 1:**
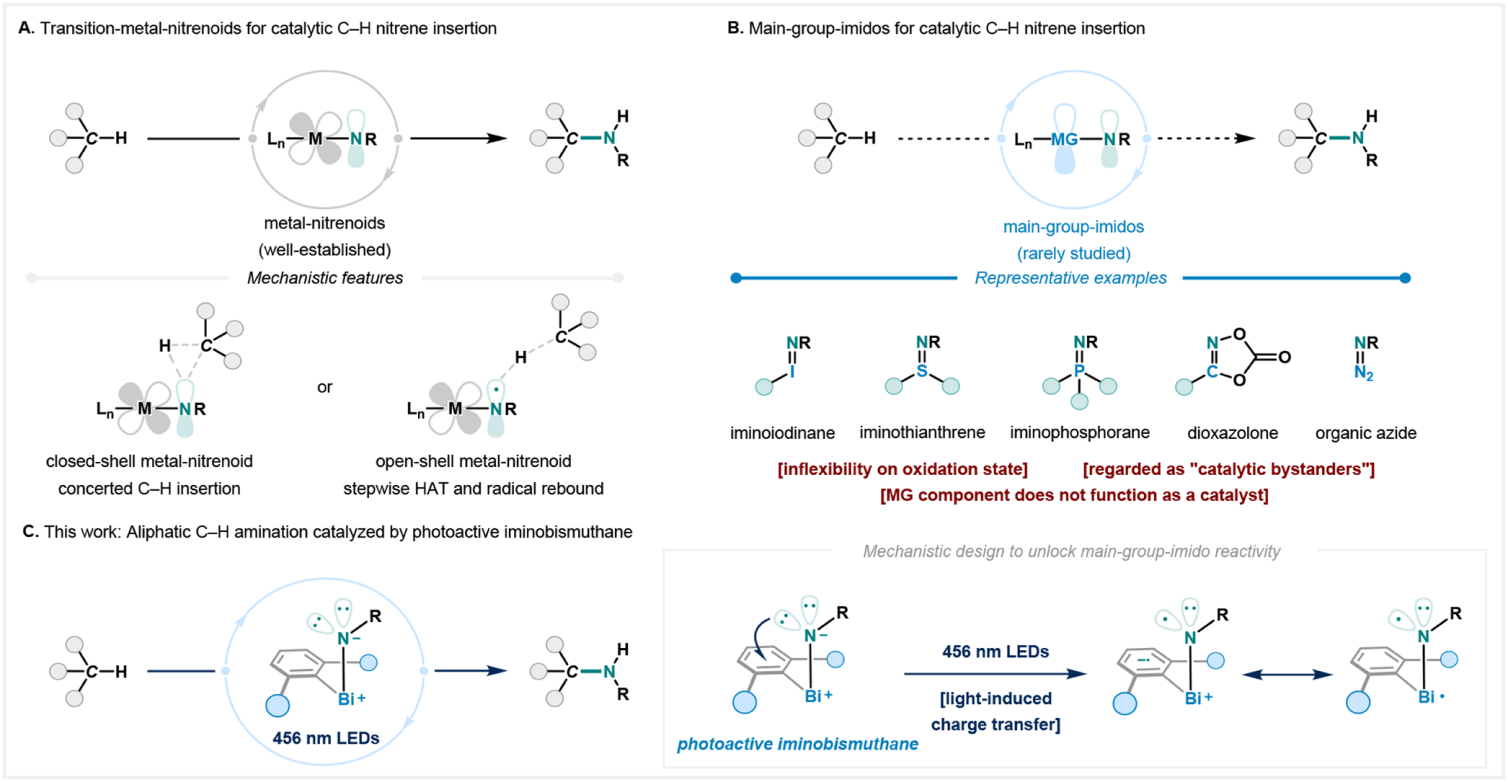
Overview of C–H amination with nitrenoid intermediates. (A) Well-established transition-metal-nitrenoids for catalytic C–H insertion via either concerted or stepwise mechanisms: a simplified orbital description. (B) Representation of an idealized catalytic C–H amination with main-group imidos and their examples as catalytically inactive reagents. (C) This work: catalytically photoactive iminobismuthanes for direct C–H amination.

Despite the remarkable advances of transition-metal-nitrenoids in C–H amination, analogous reactivity at main-group centers remains underdeveloped.^9^ Although formal C–H amination in Cadogan-type reactions has been reported under phosphorus catalysis,^10^ these reactions proceed through 3-membered oxazaphosphiranes. Consequently, the catalytic reactivity of main-group imidos for C–H amination remains largely unexplored. This gap in the field originates from fundamental electronic differences: unlike transition metals, *p*-block elements lack accessible *d*-orbitals, resulting in a restricted range of oxidation states and, thus, reduced electronic flexibility at the reactive center. Historically, such limitations have led main-group elements to be regarded as redox-innocent or “catalytic bystander” components, and their catalytic redox capabilities have therefore been underappreciated. Indeed, most of the nitrene precursors in transition-metal catalysis are main-group imido components,^11,12^ such as iminoiodinanes,^13^ iminothianthrenes,^14^ iminophosphoranes,^15^ dioxazolones,^16,17^ organic azides,^18^ etc. Even though reports on certain main-group elements exhibiting C–H amination reactivity in a stoichiometric manner exist,^19–23^ *p*-*block* imidos capable of engaging in formal and catalytic C–H nitrene insertion are rare (Figure 1B). Based on the demonstrated ability of bismuth to maneuver between oxidation states,^24–30^ we envisaged that a bismuth analogue of a metal-nitrenoid complex would be a suitable candidate. In our previous work, we demonstrated that *N,C,N*-pincer ligand-supported Bi­(I) species react readily with organic azides, leading to well-defined iminobismuthanes.^30^ However, the highly polarized bond between Bi–N prevented metal-nitrenoid-like reactivity, thus reinforcing the notion that controlling the reactivity of a nitrene bound to bismuth is a major challenge.^31,32^ Herein, we report that upon visible light irradiation, an iminobismuthane is capable of an internal ligand-to-ligand charge transfer (LLCT) process,^27–29^ leading to *N*-centered radicals (Figure 1C). This intermediate engages in a HAT process with a C–H bond of the substrate, ultimately leading to the formation of C–N bond. In contrast with the electrophilic nature of metal-nitrenoid complexes,^33,34^ the iminobismuthane is inherently nucleophilic, overcoming substrate-scope limitations in metal-catalyzed systems, where prevalent cyclic amines are prone to *N*-amination by metal-nitrenoids. Furthermore, the low proclivity for coordination to bismuth permits the use of unprotected amines as starting materials, a feature that is currently beyond the capabilities of transition-metal catalysts.^35,36^ This reactivity is demonstrated in the derivatization of natural products, drugs, pharmaceuticals, and other bioactive compounds.

## Results and Discussion

### Stoichiometric Reactivity of Iminobismuthanes

Consistent with our previous work,^30^ 2,4,6-triisopropyl arylsulfonyl azide **2a** reacted immediately with bismuth complex **1** in acetonitrile (MeCN), affording iminobismuthane **3a** in 98% yield as a pale-yellow solid (Figure 2A). Ultraviolet–visible (UV–Vis) spectroscopy of complex **3b** from tosyl (Ts) azide revealed an absorption band starting at around λ = 470 nm (green line, Figure 2B). For the assignment of the observed absorption, time-dependent density functional theory (TD-DFT) calculations were conducted. As shown in Figure 2B, TD-DFT calculations of the optimized structure of **3b** indicate that transitions at 448 and 424 nm are dominated by the highest occupied molecular orbital (HOMO) to the lowest occupied molecular orbital (LUMO) and LUMO + 1 transitions. The HOMO is mainly derived from the lone pair on the anionic nitrogen. On the other hand, the LUMO and LUMO + 1 can be described as π*-orbitals delocalized throughout the *N,C,N*-pincer ligand. To investigate a possible LLCT process leading to a putative *N*-centered radical, **3a**, bearing a pendant weak C–H bond, was subjected to blue light irradiation (456 nm light-emitting diodes, LEDs). Gratifyingly, the five-membered sultam **4a** was obtained in 58% yield (Figure 2A). More importantly, 85% of **1** was recovered after irradiation, suggesting that a catalytic process should be feasible. Optimization of the reaction parameters revealed that **4a** could be obtained in 93% yield with 10 mol % of **1** (Figure 2C, entry 1). The reactivity was maintained even when 1.0 mol % of **1** was used, leading to **4a** in 91% isolated yield (entry 2). Control experiments revealed that the reaction did not proceed thermally (up to 80 °C), in the absence of light or without the catalyst (entries 3–5). To provide evidence that a catalytic and intermolecular HAT process could be feasible, we subjected γ-terpinene—a potent hydrogen atom donor—to iminobismuthane **3b,** which was dehydrogenated to form *para*-cymene under light irradiation (Figure 2D). As shown in Figure 2E, the conversion of various aryl sulfonyl azides bearing tertiary C–H moieties to five-membered sultams occurred in good yields (**4a**, **4c**–**4e**). When a substrate with an acetal moiety was used, the expected sultam was formed, followed by ring-opening, affording **4f** in high yield. Similar to cobalt-catalyzed systems,^37^ the amination of secondary C–H bonds remained challenging, likely due to a higher bond dissociation energy and subsequent instability of the resulting secondary alkyl radical. Hence, slightly modified conditions (see Supporting Information) to obtain **4g** in moderate yields were required. The presence of only one *ortho*-ethyl group remained a challenge for this system (**4h**).

**Figure 2:**
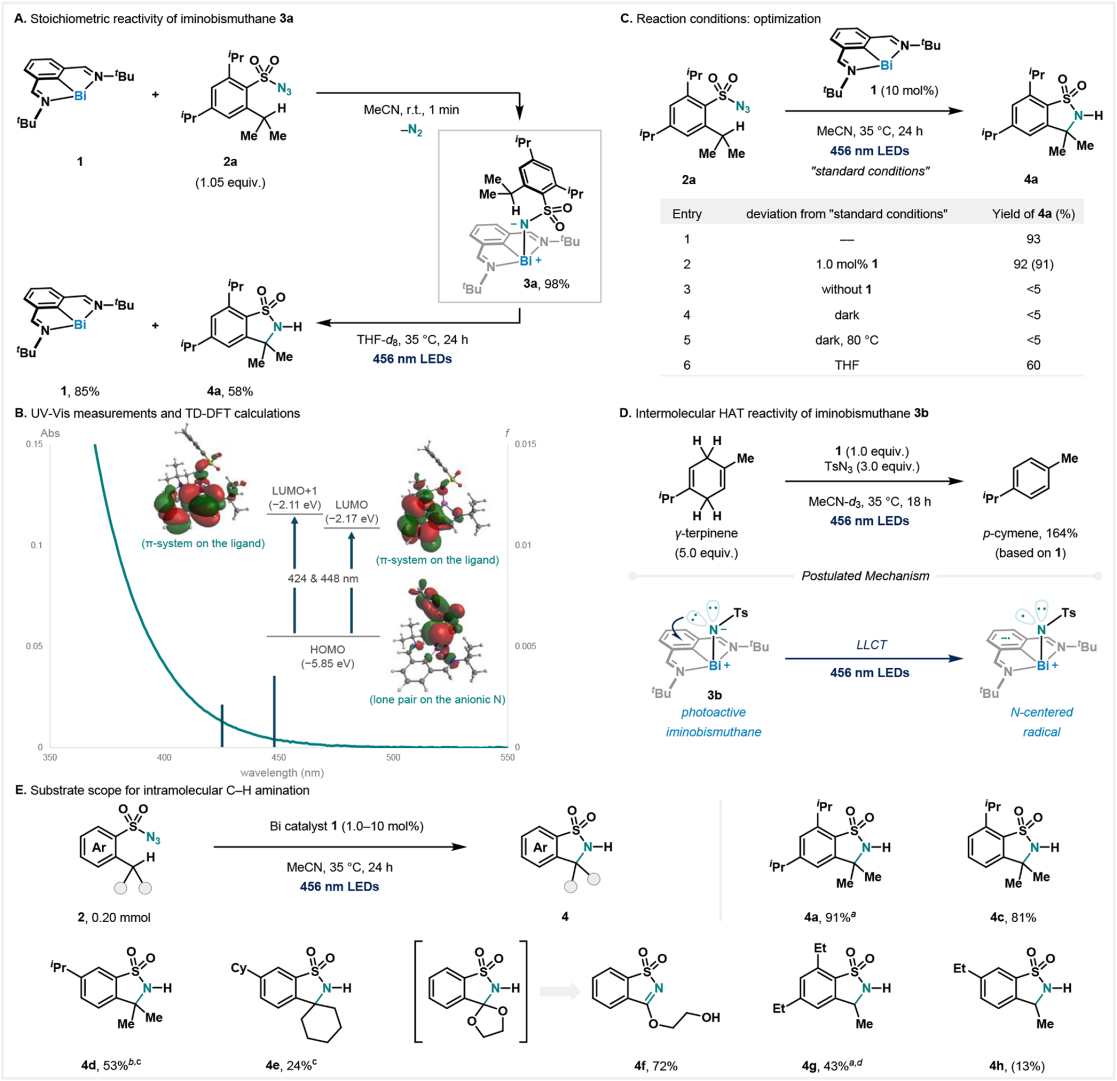
(A) Stoichiometric reactivity of a photoactive iminobismuthane. (B) UV–vis measurements (green trace) and TD-DFT calculations (blue bars) on iminobismuthane **3b** at the PBE0/ZORA-def2-TZVP level of theory. (C) Optimization of the bismuth-catalyzed intramolecular C–H amination of **2a**. Yields were determined by ^1^H nuclear magnetic resonance (NMR) spectroscopy, using trichloroethylene as an internal standard. Isolated yield is shown in parentheses. Reaction conditions: **2a** (0.050 mmol) and **1** (10 mol %) in a 0.10 M solution of MeCN at 35 °C for 24 h under 456 nm LED irradiation. (D) Intermolecular HAT reactivity of iminobismuthane **3b** under light irradiation. (E) Substrate scope for the bismuth-catalyzed intramolecular C–H amination. Isolated yields were reported on a 0.20 mmol scale unless noted otherwise. Yield determined by ^1^H NMR spectroscopy is shown in parentheses. Reaction conditions: **2** (0.20 mmol) and **1** (10 mol %) in a 0.10 M solution of MeCN at 35 °C for 24 h under 456 nm LED irradiation. *^a^* 1.0 mol % of **1**. *^b^* With CsI (1.0 equiv). *^c^* DMSO was used as the solvent. *^d^* With NaI (1.0 equiv).

### Intermolecular C–H Amination

We next sought to apply the C–H nitrene insertion to an intermolecular transformation (Figure 3). In order to benchmark the reactivity, we selected the α-C–H amination of cyclic amines as a model reaction.^38–41^ After a small optimization campaign (see Supporting Information), 2,2,2-trichloroethoxycarbonyl (Troc) azide was found to be a suitable nitrene source for the α-C–H amination of *N*-*tert*-butyloxycarbonyl-pyrrolidine (*N*-Boc-pyrrolidine) **5a,** and the product **7a** was obtained in 77% isolated yield. This catalytic protocol was suitable for a range of common secondary amine protecting groups: benzyloxycarbonyl (Cbz, **7b**), acetyl (Ac, **7c**), and Troc (**7d**) derivatives were aminated in good yields. Azabicyclic substrates were also successfully aminated in moderate yields (**7e** and **7f**). In this particular case (**7f**), a bis-aminated product was observed in *ca*. 5% yield. During the substrate scope campaign, *N*-protected piperidines were found to be less reactive than *N*-protected pyrrolidines. In an attempt to improve the catalytic activity, we discovered that the addition of an inorganic base, such as sodium ethoxide (NaOEt) or cesium carbonate (Cs_2_CO_3_), significantly improved the reaction efficiency. We hypothesized that acidic side products were responsible for the lower activity, thus quenching the bismuth-imido intermediate. With these inorganic bases present, *N*-protected piperidines with different protective groups were amenable to give the desired aminated product (**7g**–**7j**). *N*-Boc-azepane was also converted into the amination product (**7k**). To test the extent of our protocol, we explored *N*-Boc-piperidines with various functional groups. Piperidines bearing cyano (CN, **5l**), hydroxy (OH, **5m**), bromo (Br, **5n**), benzyl (CH_2_Ph, **5o**), phenyl (Ph, **5p**), amide (**5q**), and pyrazole (**5r**) groups at the δ-position were tolerated and transformed into the aminated product in high diastereoselectivity. NMR spectroscopic analysis revealed that the predominant relative configuration was *trans* between the substituents at the δ-position and the NHTroc group at the α-position due to steric hindrance. Moreover, the NHTroc group is placed in the axial position, presumably due to an anomeric effect, suggesting that the reaction proceeds via an iminium cation, in which the substituents at the δ-position are located in a pseudoequatorial configuration.^42^ These results were consistent with those reported in the literature.^43^ The α-C–H amination was also compatible with spirobicyclic amines (**7s** and **7t**). Not only cyclic amines but also the acyclic variant **5u** proved amenable to the current catalytic system. Importantly, our catalytic protocol was capable of functionalizing naturally derived compounds. For instance, *N*-Boc-methyl isonipecotate (**5v**), a fragment molecule of risperidone (**5w**), and *N*-Boc-troxipide (**5x**) were successfully converted to the aminated product. The observed *cis*-configuration on **7x** further supports the formation of a possible iminium cation intermediate.^42^ In the case of (−)-nicotine (**5y**), the transiently C–H aminated product was further oxidized to form an amidine, consistent with literature precedents for this particular substrate.^41^ Finally, *N*-Boc-protected (l)-proline bearing a carboxylic acid was also tolerated in our protocol to afford the product in high diastereoselectivity (**7z**). It is important to mention that in transition-metal catalysis, the amination of α-C–H bonds of cyclic amines remains challenging due to the competing *N*-amination.^34,44^ The electrophilic nature of metal-nitrenoids is prone to nucleophilic attack by amines, leading to N–N bond formation. By contrast, owing to the reversed electronic properties of iminobismuthanes compared to metal-nitrenoids, the system reported herein does not suffer from such limitations.

**Figure 3:**
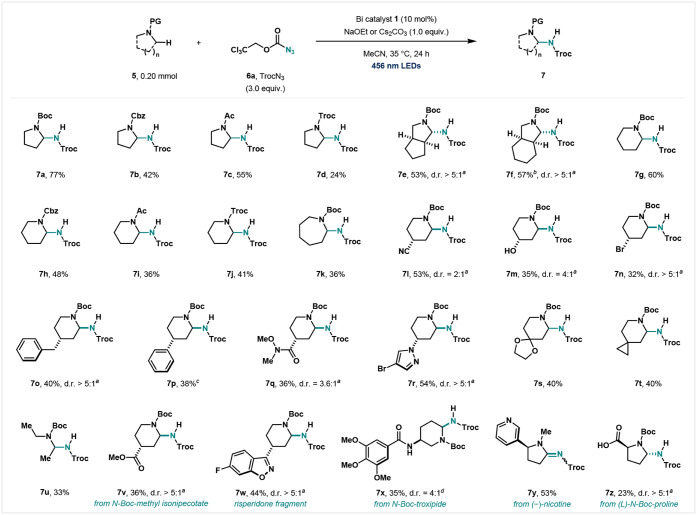
Substrate scope for α-C–H amination. Isolated yields were reported on a 0.20 mmol scale unless noted otherwise. ^1^H NMR yield is shown in parentheses. Reaction conditions: **5** (0.20 mmol), **6a** (0.60 mmol), and **1** (10 mol %) in a 1.0 M solution of MeCN at 35 °C for 24 h under 456 nm LED irradiation. NaOEt or Cs_2_CO_3_ (1.0 equiv) was added for less reactive substrates (see Supporting Information). *^a^* The d.r. ratio was determined from the ^1^H NMR spectroscopy of the crude reaction mixture. *^b^* Bis-aminated product was obtained. *^c^* The d.r. ratio was not determined due to the complex mixture of diastereomers and rotamers. *^d^* The d.r. ratio was determined after purification, as the ^1^H NMR spectroscopy of the crude reaction mixture was intractable owing to the complex mixture of rotamers.

### Intermolecular C–H Azidation

During optimization, we observed that when unprotected pyrrolidine (**8a**) was used as a substrate, two main products were formed: in addition to the C–H amination product **7d**, *N*-Troc protection followed by C–H azidation (**9a**) was also observed in a 1:3 ratio, respectively (Figure 4A). These results encouraged us to explore a selective C–H azidation protocol, as limited catalytic alternatives exist for this particular transformation.^45–47^ After optimization, less electron-deficient BocN_3_ or CbzN_3_ were found to be suitable for obtaining **9b** and **9c** selectively over C–H amination. Control experiments revealed that, in the absence of either the catalyst or light, **5d** was obtained exclusively, suggesting that protection of the *N*-atom can occur without the catalyst, and that azidation occurs in a subsequent step from **5d**. Pyrrolidine afforded the desired products in high yields (**9b** and **9c**) (Figure 4B). To demonstrate the synthetic utility of this reaction, we scaled this protocol to a 2.0 mmol scale, affording the desired product **9c** in 43% yield. Substituents, such as a methyl or phenyl group, at the α-position of pyrrolidine have a slightly negative effect on the reactivity, and the products were obtained in moderate yields (**9d** and **9e**). (L)-Proline *tert*-butyl (^*t*^Bu) ester was tolerated under our reaction conditions, yielding product **9f** as a single diastereomer. This protocol also accommodated piperidines; **9g** and **9h** were obtained in 37% and 41% yields, respectively. Similar to the aforementioned amination reaction, the *trans*-selective product with the azide group at the axial position was the major diastereomer obtained in the case of δ-phenyl piperidine (**9i**), indicating the putative intermediacy of an iminium cation.^42^ Morpholine provided the amination product in the α-position to the nitrogen atom (**9j**). Azepane could also be converted to the azidated product (**9k**). For a spirocyclic substrate containing both four- and five-membered rings, the azidation proceeded at the five-membered ring, furnishing the two regioisomers in a 1.5:1 ratio (**9l**). A range of other azidoformates were also successfully employed for *N*-protection and subsequent C–H azidation reactions. For example, fluorenylmethyloxycarbonyl (Fmoc) azide and trimethylsilylethoxycarbonyl (Teoc) azide were used to give **9m** and **9n** in 20% and 57% yields, respectively. Not only conventional protecting groups but also neopentyloxycarbonyl azide was tolerated under our reaction conditions (**9o**). The azidoformate bearing a triple bond did not negatively interfere with the formation of the α-azido product (**9p**). In addition, some azidoformates derived from natural products, such as (−)-menthol, (*S*)-(−)-lactate, and (−)-borneol, afforded the desired products in good yields (**9q**–**9s**). It is important to note that transition-metal-catalyzed C–H aminations have, in general, been limited to substrates that require protection of aliphatic amines, since binding to the electrophilic metal center usually results in inhibition of the catalyst.^35,36^

**Figure 4:**
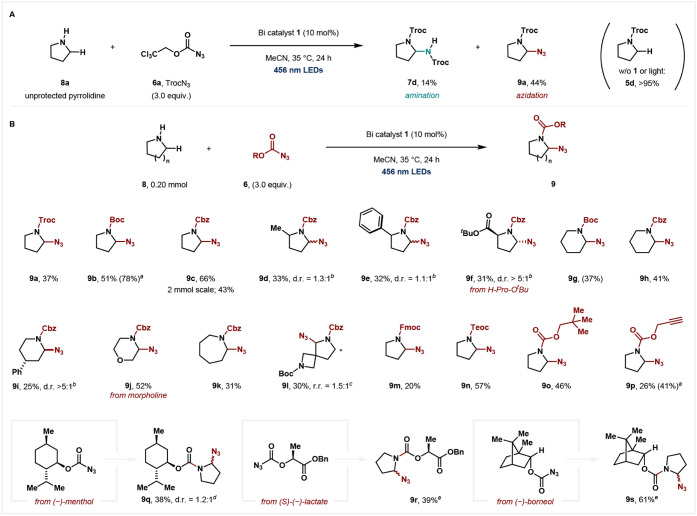
(A) Initial findings for *N*-protection/α-C–H azidation using unprotected pyrrolidine. (B) Substrate scope for *N*-protection/α-C–H azidation. Isolated yields were reported on a 0.20 mmol scale unless otherwise noted. Yield determined by ^1^H NMR spectroscopy is shown in parentheses. Reaction conditions: **8** (0.20 mmol), **6** (0.60 mmol), and **1** (10 mol %) in a 0.10 M solution of MeCN at 35 °C for 24 h under 456 nm LED irradiation. *^a^* Both ^1^H NMR yields and isolated yields are shown due to the instability and volatility of the products. *^b^* The d.r. ratio was determined from ^1^H NMR spectroscopy of the crude reaction mixture. *^c^* The r.r. ratio was determined from the ^1^H NMR spectroscopy of the crude reaction mixture. *^d^* The d.r. ratio was determined after purification, as the ^1^H NMR spectroscopy of the crude reaction mixture was intractable owing to the complex mixture of rotamers. *^e^* The d.r. ratio was not determined due to the complex mixture of diastereomers and rotamers.

The obtained α-azido amines proved to be versatile, valuable synthetic linchpins for accessing a variety of synthetically useful molecules. As illustrated in Figure 5, **9c** was converted into a diverse range of functionalized products. For example, **9c** underwent [3 + 2]-cycloaddition reactions with 3-ethynylthiophene, affording the corresponding tetrazole (**10a**). **9c** was reduced by trimethyl phosphite, affording phosphoroamidate **10b** in 76% yield. Leveraging the nucleofugality of the azide group, we conducted nucleophilic substitution reactions with various nucleophiles. Subjecting **9c** to polar solvents such as methanol or water efficiently led to methoxylated and hydroxylated products (**10c** and **10d**), respectively. Additionally, treatment with allyltrimethylsilane gave the allylation product in good yield (**10e**). Moreover, an organotrifluoroborate salt afforded the corresponding arylation product in 48% yield (**10f**).

**Figure 5:**
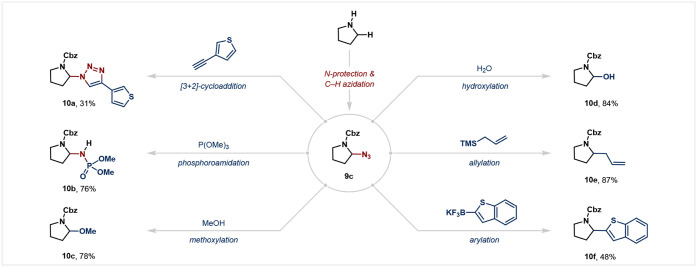
Azidation product as a synthetic linchpin. Isolated yields were reported on a 0.10 mmol scale unless otherwise noted. See the Supporting Information for detailed reaction conditions. TMS = trimethylsilyl.

### Proposed Mechanisms

Based on experimental observations and guided by precedents in transition-metal catalysis, the proposed reaction mechanisms are illustrated in Figure 6. We propose that the α-C–H amination of a protected amine with TrocN_3_, as well as the α-C–H azidation starting from an unprotected cyclic amine, share the same catalytic cycle involving a bismuth redox event (Figure 6A). The cycle begins with the reduction of organic azide by the Bi­(I) complex **1** to give iminobismuthane **3** after extrusion of N_2_. This iminobismuthane undergoes LLCT upon blue light irradiation, generating a transient *N*-centered radical species (**3′**). At this point, the *N*-centered radical undergoes C–H abstraction to form a radical pair. In-cage radical single electron transfer (SET) leads to an iminium cation and a nitrogen anion with concomitant regeneration of the Bi­(I) complex **1**.^26^ In the case of C–H amination, nucleophilic addition occurs to form the observed amination product after SET (Figure 6B, top). In the case of C–H azidation, *in situ* protection of the secondary cyclic amine releases hydrogen azide (HN_3_), which is intercepted by the iminium cation to afford the *N*-protected/α-C–H azidated product (Figure 6B, bottom). Overall, azidoformate plays several roles in this catalytic reaction, as it acts as a HAT agent, as well as a protecting and azidating reagent, a feature of this protocol when compared to other catalytic systems using similar azides. Efforts to detect the putative open-shell intermediates are currently ongoing.

**Figure 6:**
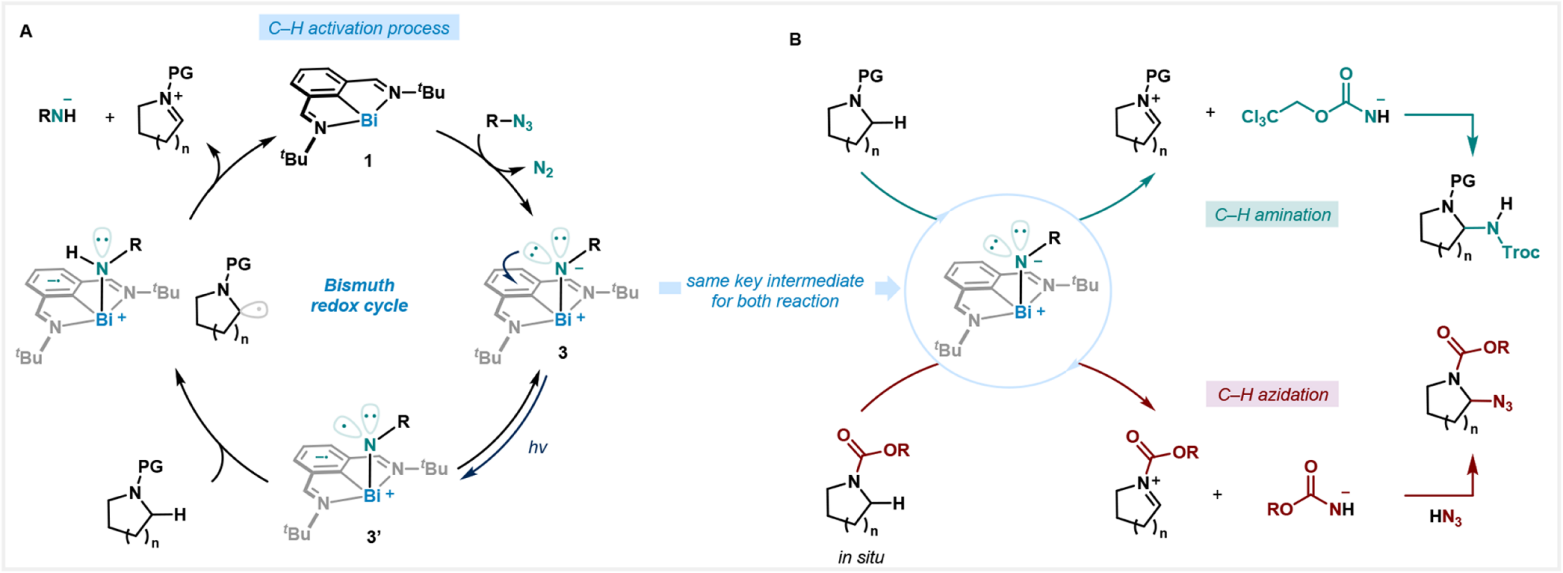
Proposed mechanism. (A) Bismuth redox cycle involving C–H abstraction to generate an iminium cation intermediate. (B) Iminobismuthane as a unifying key intermediate for both amination and azidation.

## Conclusion

In this study, a photoactive iminobismuthane catalyzes direct α-C–H amination and azidation reactions, mimicking the reactivity of transition-metal-nitrenoids. Stoichiometric reactions and UV–vis spectroscopy combined with TD-DFT revealed that, upon blue-light irradiation, the iminobismuthane undergoes an LLCT process capable of HAT reactivity. With this mechanistic blueprint, a bismuth-catalyzed direct C–H amination protocol was demonstrated using azides. Altering the substrate/organic azide combinations causes the iminobismuthane to form different nitrogenated products: the use of protected amines with TrocN_3_ delivered α-C–H amination products, while the use of unprotected amines with azidoformates furnished α-C–H azidation products. Due to the distinct electronic nature of the iminobismuthane compared to other metal-nitrenoids, our reaction system did not suffer from undesired *N*-amination reactions or catalyst deactivation by amine coordination to the bismuth center. We believe that this work provides evidence that main-group imido intermediates have real potential in synthesis, challenging their traditional supporting role as ancillary components in catalysis.

## Supplementary Material



## Notes

Additional experimental details, materials, methods, theoretical calculations, and characterization data (^1^H, ^13^C, ^19^F and ^31^P NMR and HRMS) for all new compounds (PDF)
