# Correction to “Photoactive Iminobismuthanes for Catalytic C–H Amination”

**DOI:** 10.1021/jacs.6c11027

**Published:** 2026-06-24

**Authors:** Takuya Tsuruta, Hye Won Moon, Markus Leutzsch, Benedict A. Williams, Davide Spinnato, Raquel Maray-Antolín, Aaron Ullman, Josep Cornella

The Funding statement was not present in the online version of this publication. Complete funding information is presented below:

