# Supplementary material for: Photoactive Iminobismuthanes for Catalytic C–H Amination
Source: J Am Chem Soc. 2026 May 5;148(19):20129–36. doi: 10.1021/jacs.6c04805 (PMC13195651; doi:10.1021/jacs.6c04805)
Supplement: Supplementary file 1 [file ja6c04805_si_001.pdf]

Supporting Information

Photoactive Iminobismuthanes for Catalytic C–H Amination

Takuya Tsuruta, Hye Won Moon, Markus Leutzsch, Benedict A. Williams, Davide Spinnato, Raquel Maray-Antolín, Aaron Ullman, and Josep Cornella*

Max-Planck-Institut für Kohlenforschung, Kaiser-Wilhelm-Platz 1, 45470,

Mülheim an der Ruhr, Germany.

cornella@kofo.mpg.de

Table of Contents

1. General Information 4

2. General Experimental Procedure.. 6

2.1. General procedure for intramolecular catalytic C–H amination 6

2.2. General procedure for intermolecular catalytic C–H amination 6

2.3. General procedure for intermolecular catalytic C–H azidation............................. 7

3. Substrate Preparation Procedure... 8

3.1. Preparation of arylsulfonyl azides 8

3.2. Preparation of azidoformates.. 10

3.2.1 General procedure for the synthesis of azidoformates from chloroformates 10

3.2.2 General procedure for the synthesis of azidoformates from alcohols 12

3.2.3 Synthesis of 2-(trimethylsilyl)ethyl carbonazidate (**6n**)................................ 14

3.3. Preparation of *N*-protected amines 15

3.3.1 General Procedure A for the Preparation of *N*-Boc-protected amines 15

3.3.2. General Procedure B for the Preparation of *N*-Boc-protected cyclic amines 16

3.3.3. Preparation of *N*-protected cyclic amines.. 16

4. Reaction Development 18

4.1. Stoichiometric experiments of **3a**.. 18

4.2. Reaction optimization for intramolecular C–H amination of **2a**......................... 20

4.3. Demonstration of HAT reactivity of iminobismuthane **3b** 21

4.4. Reaction optimization for intramolecular C–H amination of **2g**......................... 22

4.5. Reaction optimization for intermolecular C–H amination 23

4.6. Reaction optimization for intermolecular C–H amination for *N*-Boc-piperidine 24

4.7. Reaction optimization for intermolecular C–H azidation.................................... 25

5. UV-Vis measurement 26

6. Product Characterization 27

6.1. Characterization of intramolecular C–H amination products **4**........................... 27

6.2. Characterization of intermolecular C–H amination products **7**........................... 31

6.3. Characterization of intermolecular C–H azidation products **9** 57

7. Derivatization of α-azido product **9c** 80
7.1. [3+2]-cycloaddition 80
7.2. Phosphoroamidation 81
7.3. Methoxylation 82
7.4. Hydroxylation 83
7.5. Allylation 84
7.6. Arylation 85
8. TD-DFT calculations 86
9. EPR experiments 89
10. NMR Spectra 90
11. References 329

1. General Information

Unless otherwise stated, all manipulations were performed under an argon atmosphere using standard Schlenk line techniques or in an MBraun argon-filled glove box.

Chemicals: Unless otherwise noted, all reagents were obtained from commercial suppliers (Sigma - Aldrich, TCI Deutschland GmbH, BLD Pharmatech GmbH, ABCR GmbH) and used without further purification. Anhydrous MeCN (1.00 L, 99.8%) were purchased from Sigma-Aldrich, stored directly in the glovebox. 3 Å and 4 Å molecular sieves were activated at 250 °C under high vacuum (1 ×10−4bar) for 3 days. The commercially obtained acetonitrile-d_3 and THF-d_8 was dried over molecular sieves. Chloroform-d_1 was neutralized with Na_2CO_3. The bismuth catalysts **1** were synthesized according to literature methods.[1]

Column Chromatography: Column chromatography was carried out using Merck silica gel 60 (40-63 µm) or preparative TLC (Silica gel 60 F254, 1 mm, 20×20 cm, Sigma-Aldrich).

Nuclear Magnetic Resonance Spectroscopy: NMR data were recorded on a Bruker AVIII HD 300 MHz, Bruker AVIII HD 400 MHz, Bruker AVIII 500 MHz or Bruker AVNeo 600 MHz NMR spectrometer. ^{1}H and ^{13}C chemical shifts are reported relative to the solvent residual peaks as an internal reference. For ^{1}H NMR the following residual proton peaks of the deuterated solvents were used: chloroform-d_3 (δ = 7.26 ppm); THF-d_8 (δ = 3.58 ppm), acetonitrile-d_3 (δ = 1.94 ppm), DMSO-d_6 (δ = 2.50 ppm). For ^{13}C NMR: chloroform-d_3 (δ = 77.16 ppm), DMSO-d_6 (δ = 39.52 ppm), acetone-d_6 (δ = 29.84 ppm). ^{13}C spectra were acquired with broadband ^{1}H decoupling unless mentioned otherwise. ^{19}F NMR spectra measured at 565 MHz was generally acquired with broadband ^{1}H decoupling unless mentioned otherwise. ^{31}P NMR spectra measured at 243 MHz. Data is reported as follows: s = singlet, d = doublet, t = triplet, q = quartet, quin = quintet, sext = sextet, sept = septet, m = multiplet, brs =broad singlet, brd =broad doublet.

Electron Paramagnetic Resonance Spectroscopy: EPR was recorded on a Bruker Elexsys E500 CW EPR spectrometer equipped with a ER4116DM resonator and an Oxford ESR900 cryostat at the corresponding temperature.

Mass Spectroscopy: Electron Ionization (EI) mass spectrometry (MS) was performed on a Finnigan MAT 8200 (70 eV) or MAT 8400 (70 eV) spectrometer. Electrospray ionization (ESI) mass spectrometry was conducted on a ESQ 3000 spectrometer (Bruker). High resolution mass spectra were determined on a APEX III FT-MS (7 T magnet, Bruker) or MAT 95 (Finnigan).

UV-Vis Spectroscopy: UV-Vis absorption spectra were recorded on a Cary 6000i UV-Vis-NIR Spectrophotometer.

Abbreviations: Me = methyl, Et = ethyl, ^{i}Pr = isopropyl, ^{n}Bu = normal butyl, Cy = cyclohexyl, Ts = tosyl, Troc = 2,2,2-trichloroethoxycarbonyl, Boc = *tert*-butyloxycarbonyl, Cbz = benzyloxycarbonyl, Fmoc = fluorenylmethyloxycarbonyl, Teoc = trimethylsilylethoxycarbonyl, Bn = benzyl, Bz = benzoyl, Ms = methanesulfonyl, Ac = acethyl, TMS = trimethylsilyl, MeCN = acetonitrile, THF = tetrahydrofuran, Et_2O = diethylether, DMSO = dimethyl sulfoxide, DCM = dichloromethane, EtOAc = ethyl acetate.

Photochemical Setup: The light irradiation reaction was performed with two 456 nm Kessil lamps at 100% intensity with a cooling fan. The two lamps were assembled at ca. 23 cm from each other. The reaction vessels will be then located at ca. 6 cm from each lamp. The reactor was set up on top of a stirring plate, and the temperature of the reactions was maintained around 35 °C by using a cooling fan placed on top of the reactor.

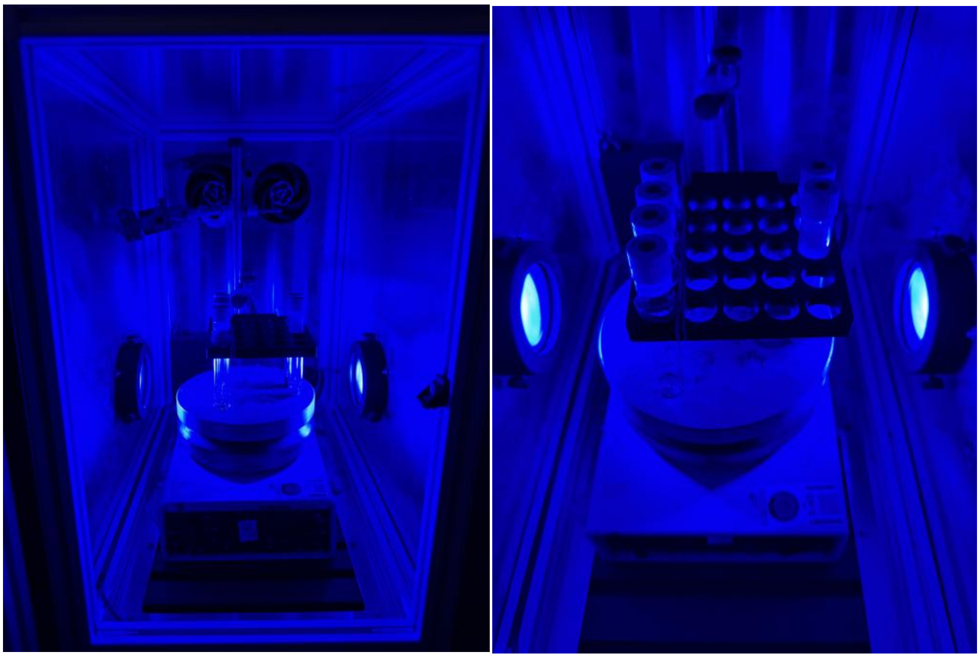

Figure S1. Photochemical setup

CAUTION: Azide compounds are known to be potentially explosive. Organic azides were stored in a fridge under argon atmosphere. Moreover, potentially formed hydrogen azide (HN_3) is both highly toxic and explosive. A full safety protocol has been observed all the time. While we did not encounter any issues during the reaction development, proper precautions were taken.

2. General Experimental Procedure

2.1. General procedure for intramolecular catalytic C–H amination

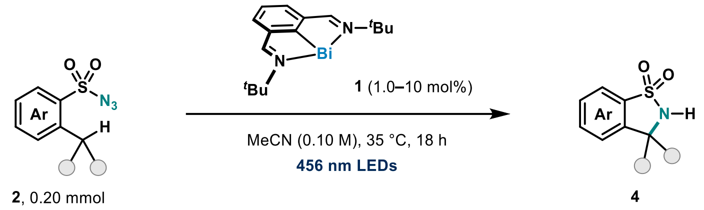

In an argon-filled glovebox, bismuth complex **1** (9.0 mg, 0.020 mmol, 10 mol%) was dissolved in dry MeCN or DMSO (0.050–0.20 M) in an oven-dried reaction vial equipped with a Teflon-coated magnetic stir bar. Then arylsulfonyl azide **2** (0.20 mmol, 1.0 equiv.) was added and the reaction mixture was stirred for 1 min until the dark green color fades. For some substrates, NaI or CsI (0.20 mmol, 1.0 equiv.) was added. The vial was sealed with a screw cap, taken out of the glovebox, and placed between two 456 nm Kessil lamps at 100% intensity with stirring. After 18 h, the reaction mixture was concentrated under reduced pressure. The crude material was purified by flash column chromatography or by preparative TLC to give the corresponding product.

2.2. General procedure for intermolecular catalytic C–H amination

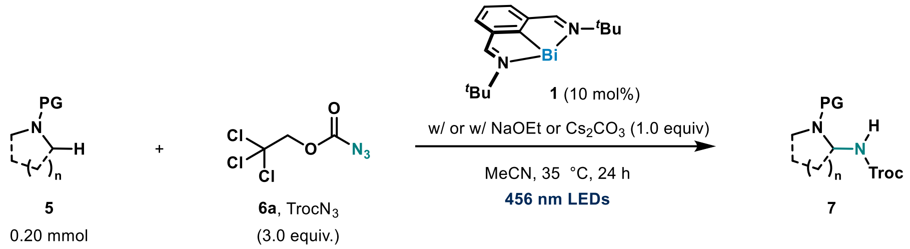

In an argon-filled glovebox, bismuth complex **1** (9.0 mg, 0.020 mmol, 10 mol%) was dissolved in dry MeCN (0.20 mL, 1.0 M) in an oven-dried reaction vial equipped with a Teflon-coated magnetic stir bar. Then $TrocN_3$ (0.60 mmol, 3.0 equiv.) was added and the reaction mixture was stirred for 1 min until the dark green color fades. Subsequently *N*-protected amine (0.20 mmol, 1.0 equiv.) and base (0.20 mmol, 1.0 equiv., if necessary) were added. The vial was sealed with a screw cap, taken out of the glovebox, and placed between two 456 nm Kessil lamps at 100% intensity with stirring. After 18 h, the reaction mixture was concentrated under reduced pressure. The crude material was purified by flash column chromatography to give the corresponding product.

Remarks:

1. $TrocN_3$ should be stored under inert atmosphere in the refrigerator; otherwise, it gradually decomposes.
2. As the Bi(I) complex **1** is potentially strongly reducing enough to react with an electrophilic moiety of a substrate, $TrocN_3$ should be added first.

3. As some of amination products were found to hydrolyze under the acidic environment of silica gel, Et_3N should therefore be added to the eluent to neutralize the silica. Additionally preparative TLC was found not to be suitable for purification.
4. As some of amination products were found to be hydrolyzed by DCl in $CDCl_3$, it must be neutralized through basic Al_2O_3, or stored over Na_2CO_3.
5. Although the efficiency of the model reaction (see section 4.5) did not change when a non-oven-dried reaction vial was used, oven-dried one was employed to exclude any possible variables.

2.3. General procedure for intermolecular catalytic C–H azidation

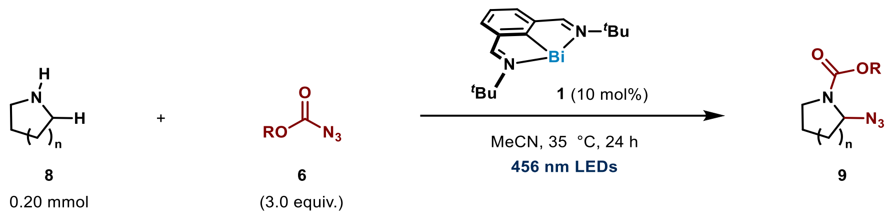

In an argon-filled glovebox, bismuth complex **1** (9.0 mg, 0.020 mmol, 10 mol%) was dissolved in dry MeCN (1.0 mL, 0.20 M) in an oven-dried reaction vial equipped with a Teflon-coated magnetic stir bar. Azidoformate (0.60 mmol, 3.0 equiv.) was then added and the reaction mixture was stirred for 1 1 min until the dark green color fades. Subsequently unprotected azacycle (0.20 mmol, 1.0 equiv.) was added. The vial was sealed with a screw cap, taken out of the glovebox, and placed between two 456 nm Kessil lamps at 100% intensity with stirring. After 18 h, the reaction mixture was concentrated under reduced pressure. The crude material was purified by flash column chromatography give the corresponding product.

Remarks:

1. Azidoformates should be stored under inert atmosphere in the refrigerator; otherwise, they gradually decompose.
2. As the Bi(I) complex **1** is potentially strongly reducing enough to react with an electrophilic moiety of a substrate, azidoformates should be added first.
3. By contrast to amination products, the azide products are stable enough to be separated with silica gel without neutralization. However, preparative TLC was found not to be suitable for purification.
4. Some of azidation products were prone to undergo E2 elimination to form the corresponding olefin.
5. Although the efficiency of the model reaction (see section 4.5) did not change when a non-oven-dried reaction vial was used, oven-dried one was employed to exclude any possible variables.

3. Substrate Preparation Procedure

3.1. Preparation of arylsulfonyl azides

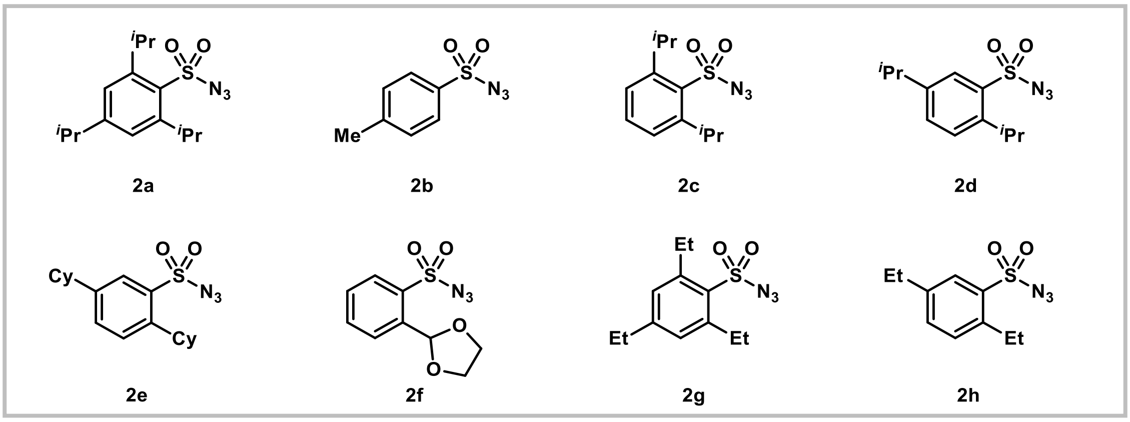

The arylsulfonyl azides depicted above (**2a**, **2b**, **2d**, **2e**, **2g**, **2h**) have been already described in the literature and were prepared according to the reported procedure.[2] Arylsulfonyl azides (**2c**, **2f**) were synthesized according to the following procedure.

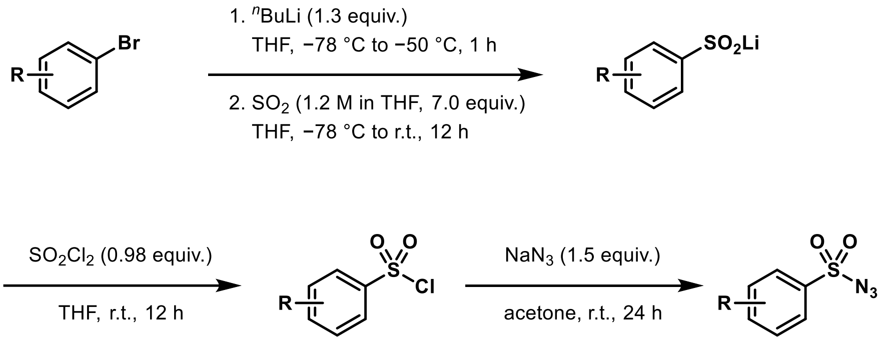

Arylsulfonyl chloride was prepared according to a literature procedure.[3] To a stirred solution of arylbromide (1.0 equiv.) in anhydrous THF (0.50 M), ^{n}BuLi (1.3 equiv., 2.5 M in hexane) was added at −78 °C under an argon atmosphere and the mixture was warmed to −50 °C and stirred for 1 h. The resulting suspension was then added to a stirred sulfur dioxide solution in THF (7.0 equiv., 1.2 M) at −78 °C under an argon atmosphere. After stirring for 12 h at 25 °C, lithium sulfinate crashed out as a white solid, which was isolated by filtration and washed with copious Et_2O. The resulting crude solid was then used in the next reaction without further purification.

To a suspension of lithium sulfinate in anhydrous THF (15 mL), sulfuryl chloride (0.98 equiv.) was added dropwise over a period of 20 min at 0 °C. After stirring for 10 min, the solvent was removed under reduced pressure and the reaction mixture was taken up in EtOAc and washed with water. The organic phase was dried over anhydrous Na_2SO_4, and concentrated under reduced pressure. The resulting crude material was then purified by flash column chromatography.

To a stirred solution of the corresponding arylsulfonyl chloride in acetone at 25 °C, NaN_3 (1.5 equiv.) was added portion-wise. After stirring for 24 h at 25 °C, the acetone was removed under reduced pressure and the crude product was extracted from the water using DCM. The organic phase was dried over anhydrous Na_2SO_4, filtered and concentrated under reduced pressure. The resulting crude material was then purified by flash column chromatography.

2,6-diisopropylbenzenesulfonyl azide (**2c**)

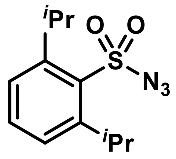

Following the general procedure starting from 2-bromo-1,3-diisopropylbenzene, the corresponding arylsulfonyl azide was obtained in 35% yield as a colorless oil.

R_f = 0.7 (EtOAc/pentane = 1:9, visualized under UV light)

NOTE: The presence of sulfonyl azide moiety was confirmed by HRMS.

^{1}H NMR (600 MHz, CDCl$_3$, 298 K) δ 7.54 (ddt, *J* = 8.1, 7.5, 0.5 Hz, 1H), 7.40 (d, *J* = 7.8 Hz, 2H), 4.11–4.03 (m, 2H), 1.29 (d, *J* = 6.7 Hz, 12H).

^{13}C NMR (151 MHz, CDCl$_3$, 298 K) δ 151.0, 134.8, 134.0, 126.2, 30.1, 24.9.

HRMS (ESI positive): calc'd for $C_{12}H_{17}N_3O_2S_1Na_1$ $[M+Na]^+$: 290.09337, found: 290.09328.

2-(1,3-dioxolan-2-yl)benzenesulfonyl azide (2f)

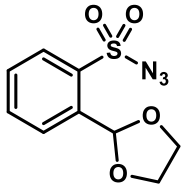

Following the general procedure starting from 2-(2-bromophenyl)-1,3-dioxolane (2.3 g, 10 mmol),[4] the corresponding arylsulfonyl azide was obtained in 42% yield (1.1 g, 4.2 mmol) as a white solid.

R_f = 0.15 (EtOAc/pentane = 1:9, visualized under UV)

NOTE: The presence of sulfonyl azide moiety was confirmed by HRMS.

^{1}H NMR (600 MHz, CDCl$_3$, 298 K) δ 8.08 (ddd, *J* = 8.0, 1.3, 0.4 Hz, 1H), 7.94 (ddt, *J* = 7.8, 1.4, 0.5 Hz, 1H), 7.73 (dddd, *J* = 7.8, 7.4, 1.3, 0.5 Hz, 1H), 7.58 (dddd, *J* = 8.0, 7.4, 1.4, 0.3 Hz, 1H), 6.53 (s, 1H), 4.19–4.07 (m, 4H).

^{13}C NMR (151 MHz, CDCl$_3$, 298 K) δ 137.5, 137.3, 134.9, 130.3, 129.9, 128.3, 99.0, 65.7.

HRMS (ESI positive): calc'd for $C_9H_9N_3O_4S_1Na_1$ $[M+Na]^+$: 278.020598, found: 278.020310.

3.2. Preparation of azidoformates

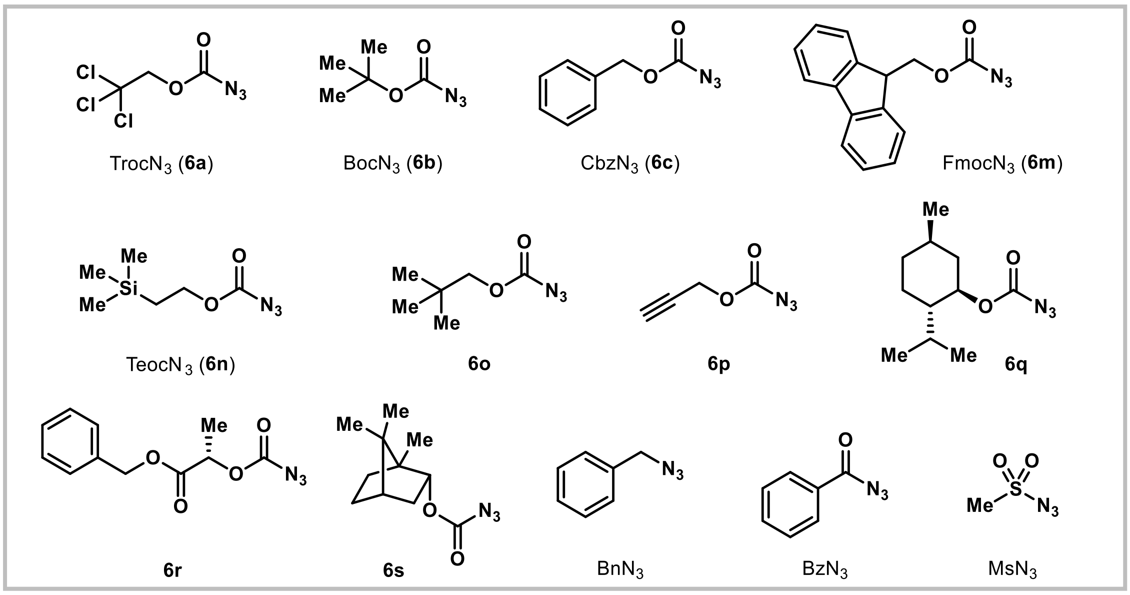

Azidoformates depicted above (**6a–6c**, **6m**, BnN_3, BzN_3, MsN_3,) have been previously described and were prepared according to the reported procedure.[5–9] Azides (**6n–6s**) were newly synthesized according to the following procedure.

3.2.1 General procedure for the synthesis of azidoformates from chloroformates

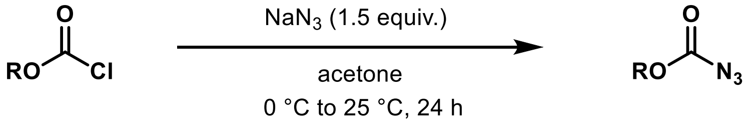

Azidoformates were prepared from the corresponding chloroformate according to the literature procedure.[5] To a well-stirred suspension of NaN_3 (1.5 equiv.) in acetone was added chloroformate dropwise at 0 °C. The reaction mixture was warmed to 25 °C and stirred for 24 h. The reaction mixture was then diluted in DCM and water. The reaction mixture was extracted with DCM (20 mL × 2), dried over anhydrous Na_2SO_4, filtered and concentrated under reduced pressure. The resulting mixture was then purified by flash column chromatography.

Neopentyl carbonazidate (**6o**)

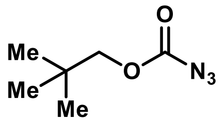

Following the general procedure starting from neopenthyl chloroformate (1.5 g, 10 mmol), neopentyl carbonazidate was obtained in 64% yield (1.01 g, 6.43 mmol) as a colorless oil.

R_f = 0.4 (pentane)

^{1}H NMR (600 MHz, CDCl$_3$, 298 K) δ 3.91 (s, 2H), 0.96 (s, 9H).

^{13}C NMR (151 MHz, CDCl$_3$, 298 K) δ 157.8, 77.9, 31.7, 26.3.

HRMS (ESI positive): calc'd for $C_6H_{11}N_3O_2Na_1$ [M+Na]$^+$: 180.074345, found: 180.074550.

Prop-2-yn-1-yl carbonazidate (**6p**)

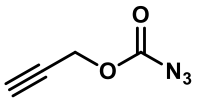

Following the general procedure starting from propargyl chloroformate (594 mg, 5.00 mmol), prop-2-yn-1-yl carbonazidate was obtained in 43% yield (268 mg, 2.15 mmol) as a colorless oil.

R_f = 0.5 (EtOAc/pentane = 5:95)

^{1}H NMR (600 MHz, CDCl$_3$, 298 K) δ 4.79 (d, *J* = 2.5 Hz, 2H), 2.57 (t, *J* = 2.5 Hz, 1H).

^{13}C NMR (151 MHz, CDCl$_3$, 298 K) δ 157.2, 76.5, 76.3, 55.7.

HRMS (ESI positive): calc'd for $C_4H_3N_3O_2Na_1$ [M+Na]$^+$: 148.01175, found: 148.01169.

(1R,2S,5R)-2-isopropyl-5-methylcyclohexyl carbonazidate (**6q**)

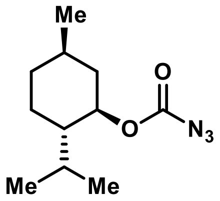

Following the general procedure starting from (−)-menthyl chloroformate (1.09 g, 5.00 mmol), (1R,2S,5R)-2-isopropyl-5-methylcyclohexyl carbonazidate was obtained in 84% yield (0.95 g, 4.2 mmol) as a colorless oil. The crude material was used for the next reaction without purification by flash column chromatography.

R_f = 0.8 (EtOAc/pentane = 1:9)

^{1}H NMR (600 MHz, CDCl$_3$, 298 K) δ 4.65 (td, *J* = 10.9, 4.5 Hz, 1H), 2.07 (dddd, *J* = 12.0, 4.4, 3.4, 1.9 Hz, 1H), 1.90 (pd, *J* = 7.0, 2.7 Hz, 1H), 1.69 (ddd, *J* = 15.0, 5.4, 3.3 Hz, 2H), 1.49 (dddt, *J* = 15.5, 8.9, 6.7, 3.4 Hz, 1H), 1.42 (dddd, *J* = 12.5, 10.9, 3.5, 2.7 Hz, 1H), 1.10–1.01 (m, 2H), 0.92 (d, *J* = 6.6 Hz, 3H), 0.90 (d, *J* = 7.0 Hz, 3H), 0.89–0.83 (m, 1H), 0.79 (d, *J* = 7.0 Hz, 3H).

^{13}C NMR (151 MHz, CDCl$_3$, 298 K) δ 157.3, 79.6, 47.0, 40.6, 34.1, 31.5, 26.3, 23.5, 22.1, 20.8, 16.4.

HRMS (ESI positive): calc'd for $C_{11}H_{19}N_3O_2Na_1$ [M+Na]$^+$: 248.136945, found: 248.136920.

3.2.2 General procedure for the synthesis of azidoformates from alcohols

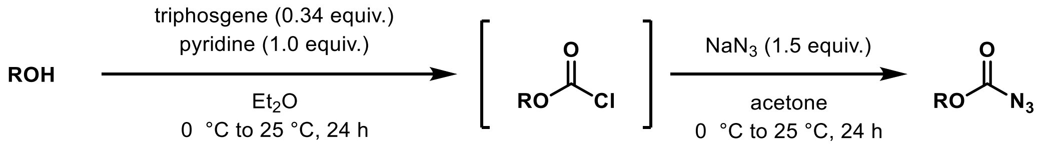

To a solution of triphosgene (0.34 equiv.) in Et_2O (0.34 M) was added dropwise a solution of alcohol (1.0 equiv.) and pyridine (1.0 equiv.) in Et_2O (0.50 M) at 0 °C over 30 min. The reaction mixture was warmed to 25 °C and stirred for 24 h. The resulting suspension was filtered and washed with Et_2O. The resulting filtrate was then washed with water. The organic layer was dried over anhydrous Na_2SO_4, filtered and concentrated under reduced pressure. The resulting crude chloroformate was used in the next reaction without further purification.

To a well-stirred suspension of NaN_3 (1.5 equiv.) in acetone was added chloroformate dropwise at 0 °C. The reaction mixture was warmed to 25 °C and stirred for 24 h. The reaction mixture was then diluted in dichloromethane and water. The reaction mixture was extracted with dichloromethane (20 mL × 2). The combined organic layers were dried over anhydrous Na_2SO_4, filtered and concentrated under reduced pressure. The resulting mixture was then purified by flash column chromatography.

benzyl (S)-2-((azidocarbonyl)oxy)propanoate (**6r**)

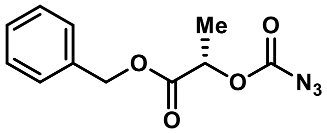

Following the general procedure starting from benzyl (*S*)-(−)-lactate (0.90 g, 5.0 mmol), the corresponding azidoformate was obtained in 63% yield (790 mg, 3.17 mmol) as a colorless oil.

R_f = 0.5 (EtOAc/pentane = 5:95, visualized under UV light)

NOTE: The presence of azide moiety was confirmed by HRMS.

^{1}H NMR (600 MHz, $CDCl_3$, 298 K) δ 7.41–7.33 (m, 5H), 5.21 (AB system, *J* = 12.3 Hz, 2H), 5.14 (q, *J* = 7.1 Hz, 1H), 1.55 (d, *J* = 7.1 Hz, 3H).

^{13}C NMR (151 MHz, $CDCl_3$, 298 K) δ 169.6, 157.2, 135.1, 128.8, 128.7, 128.3, 72.2, 67.5, 16.9.

HRMS (ESI positive): calc'd for $C_{11}H_{11}N_3O_4Na_1$ $[M+Na]^+$: 272.064175, found: 272.064080.

(1S,2R,4S)-1,7,7-trimethylbicyclo[2.2.1]heptan-2-yl carbonazidate (**6s**)

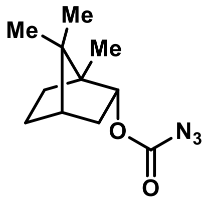

Following the general procedure starting from (−)-borneol (0.77 g, 5.0 mmol), the corresponding azidoformate was obtained in 88% yield (980 mg, 4.39 mmol) as a colorless oil.

R_f = 0.75 (EtOAc/Pentane = 5:95, visualized under UV light and stained with $KMnO_4$)

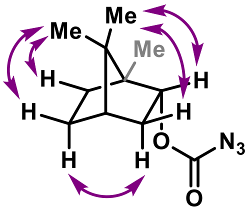

NOTE: The presence of azide moiety was confirmed by HRMS. The configuration of azidoformate was supported by the characteristic NOEs.

^{1}H NMR (600 MHz, $CDCl_3$, 298 K) δ 4.90 (ddd, *J* = 9.9, 3.4, 2.2 Hz, 1H), 2.38 (dddd, *J* = 14.0, 9.9, 4.5, 3.4 Hz, 1H), 1.88 (ddd, *J* = 13.1, 9.5, 4.6 Hz, 1H), 1.80–1.71 (m, 1H), 1.71 (t, *J* = 4.5 Hz, 1H), 1.32 (dddd, *J* = 13.1, 11.9, 4.5, 2.2 Hz, 1H), 1.25 (ddd, *J* = 12.2, 9.5, 4.4 Hz, 1H), 1.09 (dd, *J* = 14.0, 3.4 Hz, 1H), 0.90 (s, 3H), 0.88 (s, 3H), 0.87 (s, 3H).

^{13}C NMR (151 MHz, $CDCl_3$, 298 K) δ 157.9, 85.0, 49.2, 48.2, 44.8, 36.5, 28.0, 26.9, 19.8, 18.9, 13.6.

HRMS (ESI positive): calc'd for $C_{11}H_{17}N_3O_2Na_1$ $[M+Na]^+$: 246.12130, found: 246.12132.

3.2.3 Synthesis of 2-(trimethylsilyl)ethyl carbonazidate (6n)

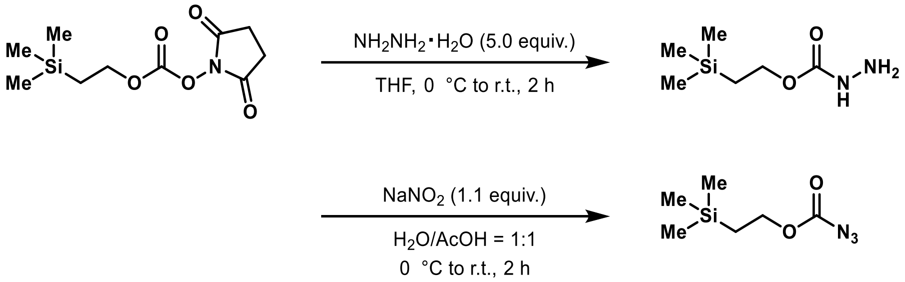

To a well-stirred suspension of 1-(2-(trimethylsilyl)ethoxycarbonyloxy)pyrrolidin-2,5-dione (Teoc-OSu, 2.6 g, 10 mmol) in THF (20 mL) , hydrazine monohydrate (2.4 mL, 5.0 equiv.) was added dropwise at 0 °C. The reaction mixture was then stirred for 2 h at the same temperature. The reaction mixture was concentrated under reduced pressure and the residue was diluted with EtOAc and extracted with EtOAc (20 mL × 2). The combined organic layers were dried over anhydrous Na_2SO_4, filtered and concentrated under reduced pressure. The crude material was used directly in the next step without further purification.

The carbamoyl hydrazine was dissolved in a 1:1 v/v H_2O/AcOH (20 mL) solution and stirred for 10 min at 0 °C. $NaNO_2$ (1.5 g, 1.1 equiv.) was then added portion-wise, and the reaction mixture was stirred for 2 h at 25 °C. The reaction mixture was diluted with water and neutralized with aq. sat. $NaHCO_3$. The aqueous phase was extracted with Et_2O (20 mL × 2). The combined organic layers were washed with aq. sat. $NaHCO_3$, dried over anhydrous Na_2SO_4, filtered and concentrated under reduced pressure. The crude material was then purified by flash column chromatography (EtOAc/pentane = 5:95) to afford the product in 40% yield (1.5 g, 8.0 mmol) over two steps as a pale-yellow oil.

R_f = 0.6 (EtOAc/pentane = 1:9, visualized under UV light)

^{1}H NMR (600 MHz, $CDCl_3$, 298 K) δ 4.33–4.28 (m, 2H), 1.10–1.03 (m, 2H), 0.05 (s, 9H).

^{13}C NMR (151 MHz, $CDCl_3$, 298 K) δ 157.6, 67.5, 17.5, −1.4.

refocused-INEPT ^{29}Si{^{1}H} NMR (119 MHz, $CDCl_3$, 298 K) δ −0.05.

HRMS (ESI positive): calc'd for $C_6H_{13}N_3O_2Si_1Na_1$ $[M+Na]^+$: 210.06692, found: 210.06691.

3.3. Preparation of *N*-protected amines

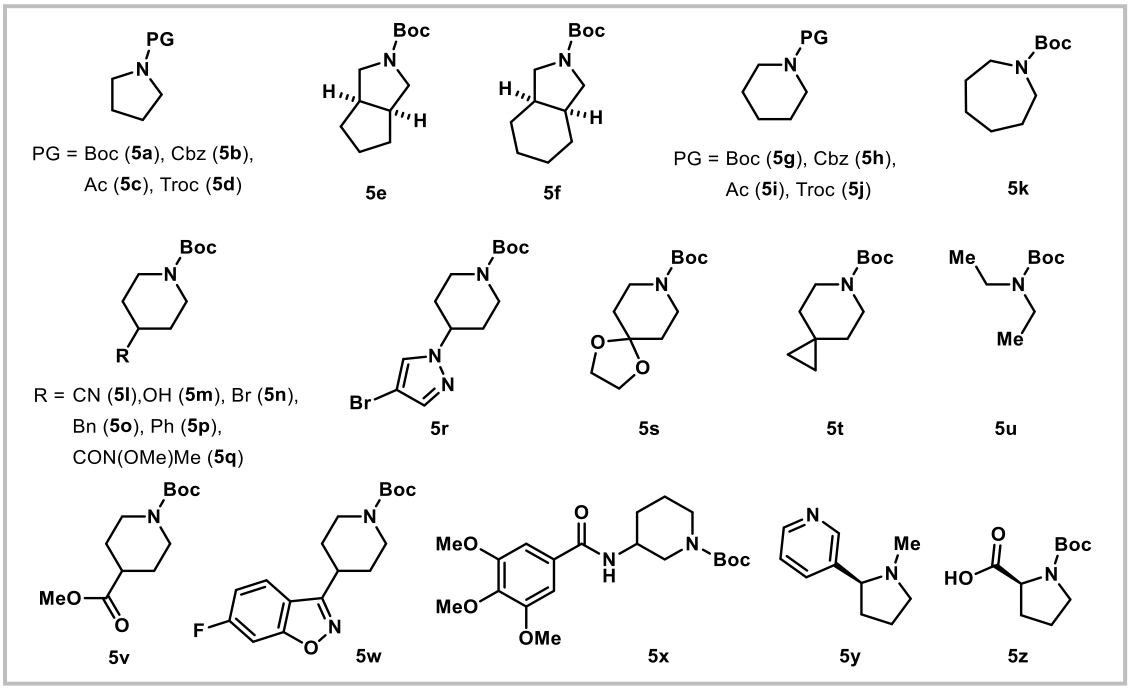

N-Boc-protected amines were prepared according to the following general procedure. Among them, compounds **5a**,[10] **5f**,[11] **5g**,[10] **5i**,[12] **5o**,[13] **5p**,[14] **5q**,[15] **5s**,[16] **5t**,[17] **5u**,[18] **5w**,[19] and **5x**,[20] have been previously reported and the NMR data matched published spectral data. Other *N*-protected azacycles **5b**,[21] **5c**,[22] **5d**,[23] **5h**,[21] **5i**,[22] and **5k**[24] were prepared according to separate literature and the NMR data matched. The compounds **5l**–**5n**, **5r**, **5v, 5y**, and **5z** were commercially available.

3.3.1 General Procedure A for the Preparation of *N*-Boc-protected amines

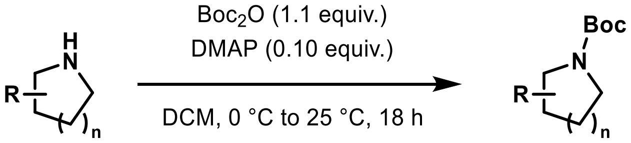

N-Boc-protected cyclic amines was prepared according to the literature procedure.[25] To a stirred solution of amine (1.0 equiv.) and DMAP (0.10 equiv.) in dry DCM (0.50 M), Boc_2O (1.1 equiv.) in dry DCM (0.50 M) was added dropwise at 0 °C under an argon atmosphere. The reaction mixture was then warmed to 25 °C and stirred for 18 h. It was quenched by the slow addition of water. The aqueous phase was extracted with DCM, dried over anhydrous Na_2SO_4, filtered and concentrated under reduced pressure. The resulting crude material was then purified by flash column chromatography to give the product.

3.3.2. General Procedure B for the Preparation of *N*-Boc-protected cyclic amines

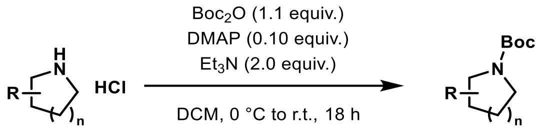

N-Boc-protected cyclic amines were prepared from an adapted literature procedure.[18] To a stirred solution of amine HCl salt (1.0 equiv.), DMAP (0.10 equiv.), and triethylamine (2.0 equiv.) in dry DCM (0.50 M), Boc_2O (1.1 equiv.) in dry DCM (0.50 M) was added dropwise at 0 °C under an argon atmosphere. The reaction mixture was warmed to 25 °C, and stirred for 18 h, after which it was quenched by the slow addition of water. The aqueous phase was extracted with DCM, dried over anhydrous Na_2SO_4, filtered and concentrated under reduced pressure. The resulting crude material was then purified by flash column chromatography to give the product.

3.3.3. Preparation of *N*-protected cyclic amines

***tert*-butyl hexahydrocyclopenta[c]pyrrole-2(1H)-carboxylate** (**5e**)

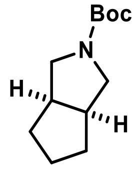

Following the general procedure B starting from Octahydrocyclopenta[c]pyrrolhydrochlorid (738 mg, 5.00 mmol), the corresponding *N*-Boc protected product was obtained in 92% yield (975 mg, 4.62 mmol) as a colorless oil.

R_f = 0.7 (EtOAc/Pentane = 2:8, stained with $KMnO_4$)

^{1}H NMR (600 MHz, $CDCl_3$, 298 K) δ 3.52 (s, 2H), 3.17–3.00 (m, 2H), 2.64–2.56 (m, 2H), 1.83–1.70 (m, 3H), 1.61–1.54 (m, 2H), 1.50–1.40 (m, 1H), 1.45 (s, 9H)

^{13}C NMR (151 MHz, $CDCl_3$, 298 K) δ 154.7, 79.1, 52.4, 52.0, 43.5, 42.6, 32.2, 28.7, 25.7.

HRMS (ESI positive): calc'd for $C_{12}H_{21}N_1O_2Na_1$ $[M+Na]^+$: 234.14645, found: 234.14662.

2,2,2-trichloroethyl piperidine-1-carboxylate (5j)

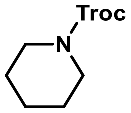

5j was prepared according to a literature procedure.[23] In a 100 mL round-bottomed flask, 2,2,2,-trichloroethyl chloroformate (2.54 g, 12.0 mmol, 1.20 equiv.) was added dropwise to a well stirred solution of triethylamine (4.18 mL, 30.0 mmol, 3.00 equiv.), piperidine (0.988 mL, 10.0 mmol, 1.00 equiv.), and DCM (35 mL) at 0 °C. The reaction was then stirred at 25 °C for 4 h. Afterwards, the solution was quenched with water and the organic layer was then washed with 5% aqueous HCl solution (20 mL) and once with aq. sat. $NaHCO_3$ (20 mL). The combined organic layers were dried over anhydrous $MgSO_4$, filtered and concentrated in vacuo. The resulting crude material was then purified by column chromatography to afford the product in 26% yield (665 mg, 2.56 mmol) as a white solid.

R_f = 0.5 (EtOAc/pentane = 2:8, stained with $KMnO_4$)

^{1}H NMR (600 MHz, $CDCl_3$, 298 K) δ 4.75 (s, 2H), 3.50 (m, 4H), 1.65–1.60 (m, 2H), 1.60–1.56 (m, 4H).

^{13}C NMR (151 MHz, $CDCl_3$, 298 K) δ 153.6, 96.0, 75.2, 45.4, 45.3, 26.0, 25.7, 24.4.

HRMS (ESI positive): calc'd for $C_8H_{12}N_1O_2Cl_3Na_1$ $[M+Na]^+$: 281.982582, found: 281.982440.

4. Reaction Development

4.1. Stoichiometric experiments of 3a

Procedure: Iminobismuthane **3a** was synthesized according to a previously reported procedure.[26] In an argon-filled glovebox, bismuth complex **1** (45 mg, 0.10 mmol, 1.0 equiv.) was dissolved in dry MeCN (5 mL, 0.02 M) in Schlenk flask. Then **2a** (32.5 mg, 0.105 mmol, 1.05 equiv.) was added, and the reaction mixture was stirred until all the dark green color of **1** was consumed. The flask was taken out of the glovebox, and the solvent was then removed under vacuum. The resulting yellow solid was washed with dry pentane under an argon atmosphere and dried under reduced pressure to afford **3a** in 98% yield as a pale-yellow solid.

In an argon-filled glovebox, **3a** (7.3 mg, 0.010 mmol, 1.0 equiv.) and trimethoxybenzene (1.7 mg, 0.010 mmol, internal standard) were dissolved in dry THF-d_8 (0.50 mL, 0.020 M) in an NMR tube. The tube was sealed with a cap, and taken out of the glovebox. Then the ^{1}H NMR spectrum was measured (Fig. S3. top). The sample was placed between two 456 nm Kessil lamps at 100% intensity with a cooling fan to keep the temperature around 35 °C and irradiated for 24 h, after which the color turned dark green, indicating the formation of **1**. In the ^{1}H NMR spectrum, **4a** was observed in 58% yield along with the regeneration of **1** in 85% yield (Fig. S3. bottom). At the same time sulfonamide was also observed as a side product in 42% yield.

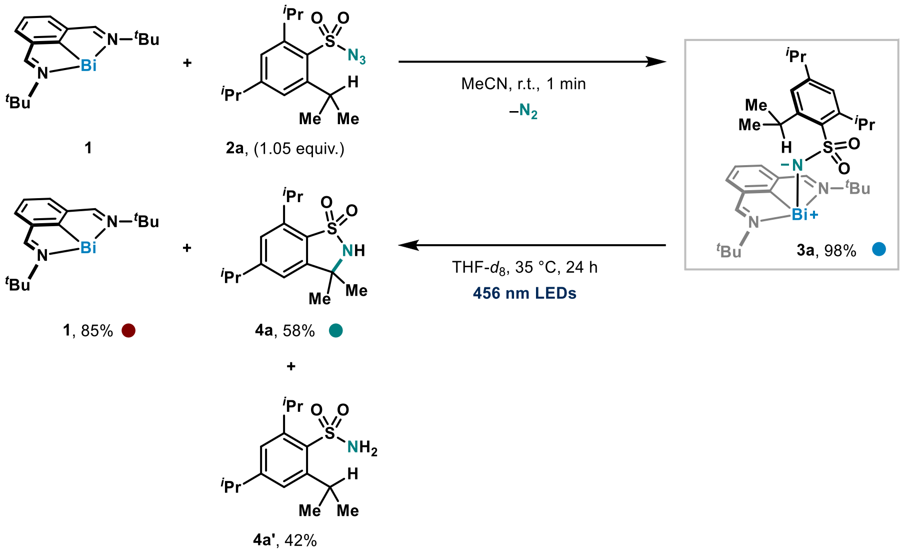

Figure S2. Stoichiometric reactivity of **3a** under 456 nm irradiation.

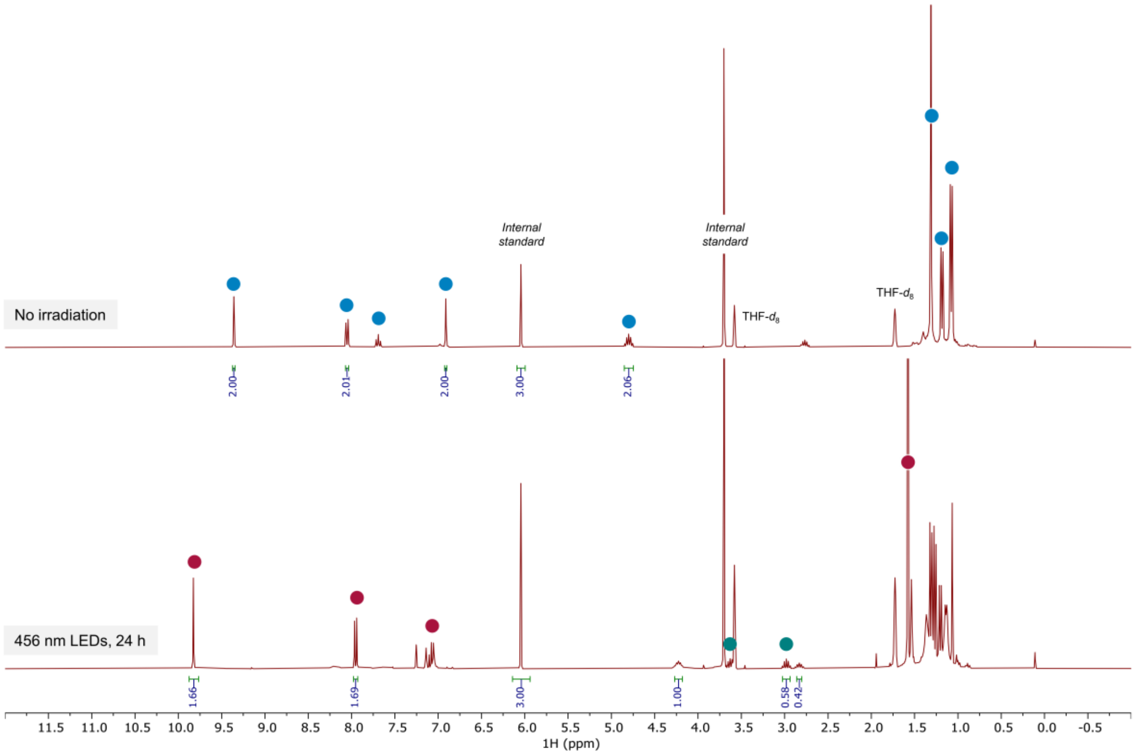

Figure S3. ^{1}H NMR spectra before irradiation (top) and after irradiation for 24 h (bottom). **3a** (blue dots), **1** (red dots), and **4a** (green dots).

4.2. Reaction optimization for intramolecular C–H amination of 2a

Procedure: In an argon-filled glovebox, bismuth complex **1** (2.3 mg, 0.0050 mmol, 10 mol%) was dissolved in dry MeCN (0.50 mL, 0.10 M) in an oven-dried reaction vial equipped with a Teflon-coated magnetic stir bar. Then 2,4,6-triisopropylbenzenesulfonyl azide **2a** (15.5 mg, 0.0500 mmol, 1.00 equiv.) was added and the reaction mixture was stirred for 1 min until the dark green color faded. The vial was sealed with a screw cap, taken out of the glovebox and placed between two 456 nm Kessil lamps at 100% intensity with stirring for 24 h. After which the reaction mixture was concentrated under reduced pressure with a rotary evaporator. The resulting residue was diluted with $CDCl_3$ and the yield was determined by ^1H NMR spectroscopy using trichloroethylene (6.6 mg, 0.050 mmol, 1.0 equiv.) as an internal standard.

Table S1. Optimization with 2,4,6-triisopropylbenzenesulfonyl azide (**2a**)

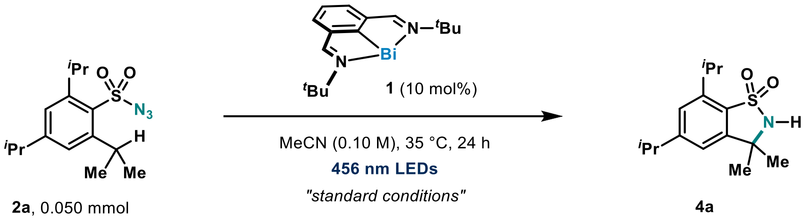

2a, 0.050 mmol

4a

Entry	deviations from *"standard conditions"*	Yield of **4a** (%)[a]
1	---	**93**
2[b]	1.0 mol% **1**	**92** (**91**)
3	without **1**	<5
4	dark	<5
5	dark, 80 °C	<5
6	THF	60

[a]Yields were determined by ^1H NMR spectroscopy with trichloroethylene as the internal standard.
[b]The reaction was conducted in 0.2 mmol scale. Isolated yield was shown in parenthese.

4.3. Demonstration of HAT reactivity of iminobismuthane 3b

Procedure: In an argon-filled glovebox, bismuth complex **1** (4.5 mg, 0.010 mmol, 1.0 equiv.) was dissolved in dry MeCN-*d*3 (0.50 mL, 0.020 M) in an NMR tube. Then TsN_3 **2b** (5.9 mg, 0.030 mmol, 3.0 equiv.) was added, and the NMR tube was shaken until all the dark green color of **1** is consumed. γ-Terpinene (6.8 mg, 0.050 mmol, 5.0 equiv.) was subsequently added to the reaction mixture, and the tube was sealed with a cap. The NMR tube was taken out of the glovebox, and ^{1}H NMR spectrum was measured. After which the tube was placed between two 456 nm Kessil lamps at 100% intensity and irradiated for 18 h. The tube was ported into the glovebox in which trimethoxybenzene (1.7 mg, 0.010 mmol, 1.0 equiv.) was added as an internal standard. The ^{1}H NMR spectrum was measured and *p*-cymene was observed in 164% yield based on **1**, indicating that the resulting iminobismuthane **3b** is capable of C–H abstraction.

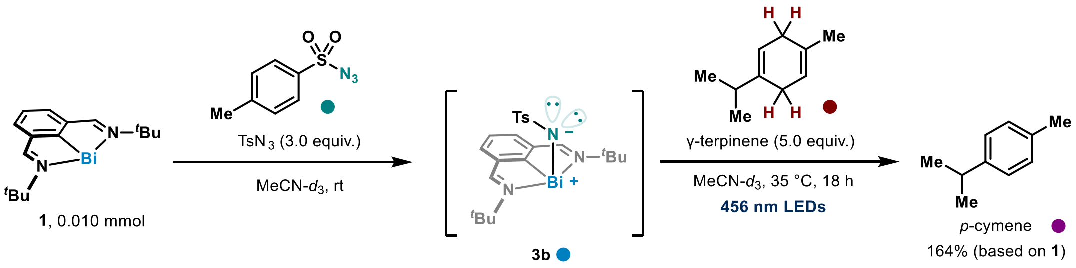

Figure S4. HAT reactivity of **3b**.

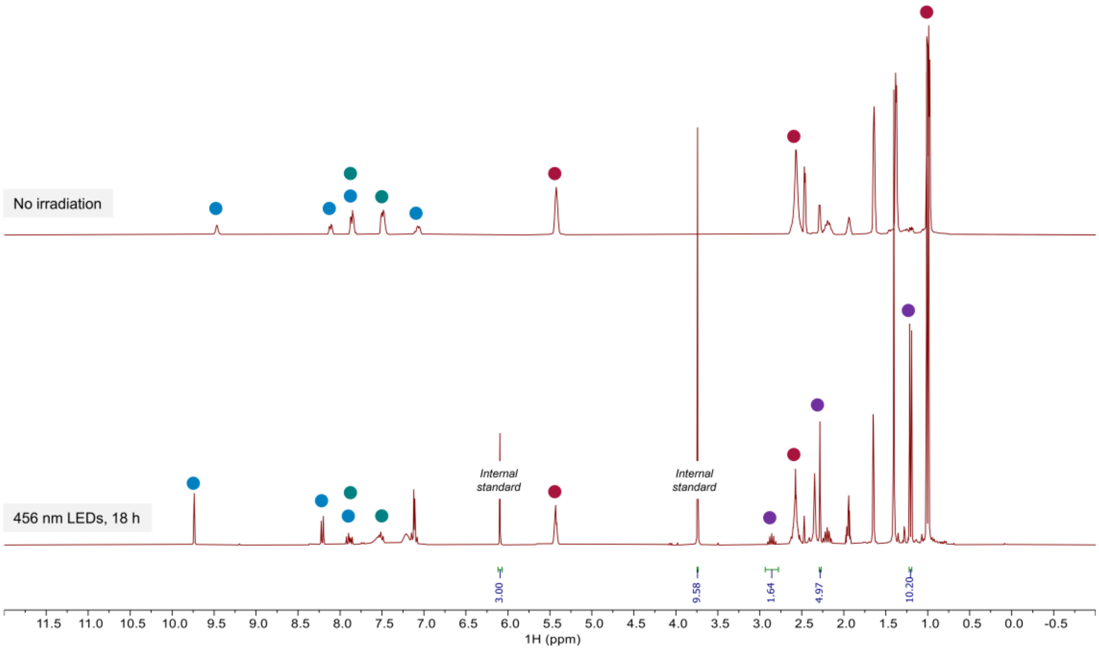

Figure S5. ^{1}H NMR spectra before irradiation (top) and after irradiation for 18 h (bottom). **3b** (blue dots), γ-terpinene (red dots), TsN_3 (green dots) and *p*-cymene (purple dots).

4.4. Reaction optimization for intramolecular C–H amination of 2g

Procedure: In an argon-filled glovebox, bismuth complex **1** (2.3 mg, 0.0050 mmol, 10 mol%) was dissolved in dry MeCN (0.10 M) in an oven-dried reaction vial equipped with a Teflon-coated magnetic stir bar. Then 2,4,6-triethylbenzenesulfonyl azide **2g** (13.4 mg, 0.0500 mmol, 1.00 equiv.) and additive (0.25 mmol, 5.0 equiv.) were added and the reaction mixture was stirred for 1 min until the dark green color faded. The vial was sealed with a screw cap, taken out of the glovebox and placed between two 456 nm Kessil lamps at 100% intensity with a cooling fan to keep the temperature around 35 °C and stirred for 24 h. The reaction mixture was concentrated under reduced pressure with a rotary evaporator. The crude residue was diluted with $CDCl_3$, and the yield was determined by ^{1}H NMR spectroscopy using trichloroethylene (6.6 mg, 0.050 mmol, 1.0 equiv) as an internal standard.

The addition of NaI was found to improve the reaction efficiency for secondary C–H bonds (Entry 5), presumably through iodide coordination to the cationic Bi center and promoting homolysis of the Bi–N bond [27]. The addition of CsI was also effective for other substrates.

Table S2. Optimization of C–H insertion with 2,4,6-triethylbenzenesulfonyl azide (**2g**).

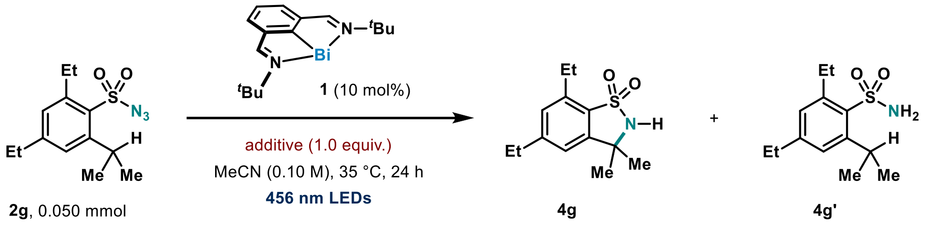

Entry	additive	Yield of **4g** (%)[a]	Yield of **4g'** (%)[a]
1	---	11	30
2	Pyridine	10	47
3	DBU	16	35
4	Na_3PO_4	15	34
5	NaI	**70**	12
6	NaI, w/o **1**	n.d.	n.d.
7	KI	46	17
8	NaF	8	27
9	NaCl	12	28

[a]Yields were determined by ^{1}H NMR spectroscopy with trichloroethylene as the internal standard.

4.5. Reaction optimization for intermolecular C–H amination

Procedure: In an argon-filled glovebox, bismuth complex **1** (2.3 mg, 0.0050 mmol, 10 mol%) was dissolved in dry solvent (1.0 mL, 0.050 M) in an oven-dried reaction vial equipped with a Teflon-coated magnetic stir bar. Then organic azide (0.15 mmol, 3.0 equiv.) was added, followed by *N*-Boc-pyrrolidine (8.6 mg, 0.050 mmol, 1.0 equiv.) to the reaction mixture, and the reaction was stirred for 1 min until the dark green color faded. The vial was sealed with a screw cap, taken out of the glovebox and placed between two 456 nm Kessil lamps at 100% intensity with a cooling fan to keep the temperature around 35 °C and stirred for 24 h. The reaction mixture was concentrated under reduced pressure with a rotary evaporator. The crude residue was diluted with $CDCl_3$, and the yield was determined by ^{1}H NMR spectroscopy using trichloroethylene (6.6 mg, 0.050 mmol, 1.0 equiv) as an internal standard.

$TrocN_3$ was found to be the most suitable nitrene source for intermolecular C–H amination.

Table S3. Reaction condition screening for intermolecular C–H amination.

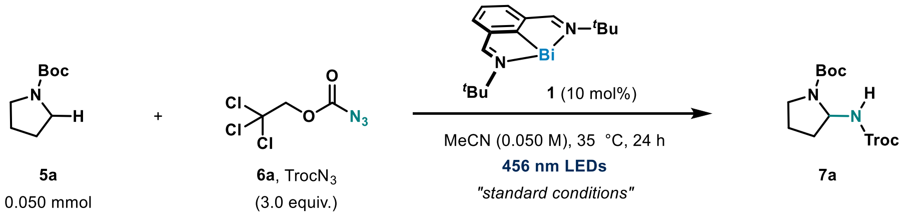

Entry	deviations from *"standard conditions"*	Yield of **7a** (%)[a]
1	---	**77**
2	TsN_3 instead of $TrocN_3$	50
3	$CbzN_3$ instead of $TrocN_3$	28
4	BnN_3 instead of $TrocN_3$	0
5	^{t}BuCN instead of MeCN	54
6	DMSO instead of MeCN	11
7	DCM instead of MeCN	46
8	Toluene instead of MeCN	25
9	PhCl instead of MeCN	30
10	390 nm LEDs instead of 456 nm LEDs	58
11	w/o **1**	0
12	w/o light (dark)	0

[a]Yields were determined by ^{1}H NMR spectroscopy with trichloroethylene as the internal standard.

4.6. Reaction optimization for intermolecular C–H amination for *N*-Boc-piperidine

Procedure: In an argon-filled glovebox, bismuth complex **1** (2.3 mg, 0.0050 mmol, 10 mol%) was dissolved in dry MeCN (1.0 mL, 0.050 M) in an oven-dried reaction vial equipped with a Teflon-coated magnetic stir bar. $TrocN_3$ (33 mg, 0.15 mmol, 3.0 equiv.) was added to the reaction mixture, and the reaction was stirred for 1 min until the dark green color faded. Then *N*-Boc-piperidine (9.3 mg, 0.050 mmol, 1.0 equiv.) and additive (0.050 mmol, 1.0 equiv.) were placed into the vial. The vial was sealed with a screw cap, taken out of the glovebox and placed between two 456 nm Kessil lamps at 100% intensity with stirring for 24 h. The reaction mixture was concentrated under reduced pressure with a rotary evaporator. The crude residue was diluted with $CDCl_3$, and the yield was determined by ^{1}H NMR spectroscopy using trichloroethylene (6.6 mg, 0.050 mmol, 1.0 equiv.) as an internal standard.

In the case of *N*-Boc-piperidine, the addition of base was found to be effective, especially K_3PO_4, NaOEt, and Cs_2CO_3. For other six or seven-membered ring substrate and less reactive substrates, these three inorganic bases were tested to determine which one provided the product in highest yield.

Table S4. Reaction condition screening for intermolecular C–H amination.

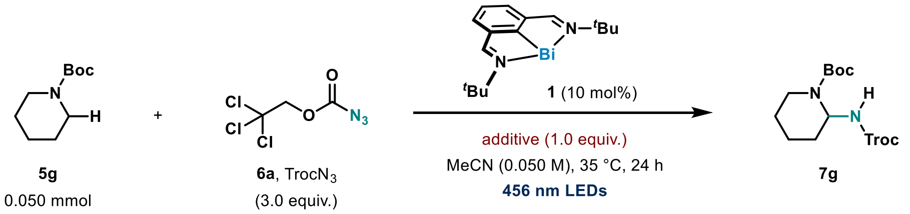

Entry	additive	Yield of **7g** (%)[a]
1	---	32
2	DMAP	0
3	Barton's base	10
4	KO^{t}Bu	0
5	K_2CO_3	52
6	K_3PO_4	**64**
7	K_2HPO_4	43
8	KH_2PO_4	20
9	KOEt	59
10	Na_3PO_4	51
11	NaOEt	**64**
12	Cs_2CO_3	**64**

[a]Yields were determined by ^{1}H NMR spectroscopy with trichloroethylene as the internal standard.

4.7. Reaction optimization for intermolecular C–H azidation

Procedure: In an argon-filled glovebox, bismuth complex **1** (2.3 mg, 0.0050 mmol, 10 mol%) was dissolved in dry MeCN (1.0 mL, 0.050 M) in an oven-dried reaction vial equipped with a Teflon-coated magnetic stir bar. Then organic azide (0.15 mmol, 3.0 equiv.) was added, followed by unprotected pyrrolidine **8a** (3.6 mg, 0.05 mmol, 1.0 equiv.) to the reaction mixture, and the reaction mixture was stirred for 1 min until the dark green color faded. The vial was sealed with a screw cap, taken out of the glovebox and placed between two 456 nm Kessil lamps at 100% intensity with a cooling fan to keep the temperature around 35 °C and stirred for 24 h. The reaction mixture was concentrated under reduced pressure with a rotary evaporator. The crude residue was diluted with $CDCl_3$, and the yield was determined by ^{1}H NMR spectroscopy using trichloroethylene (6.6 mg, 0.050 mmol, 1.0 equiv) as an internal standard.

$BocN_3$ and $CbzN_3$ gave an azidation product in high selectivity.

Table S5. Organic azides screening.

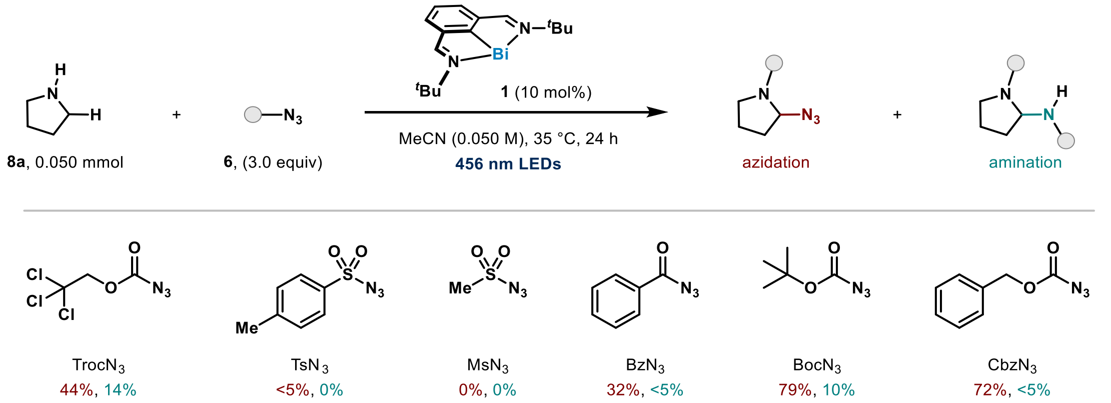

5. UV-Vis measurement

Procedure: UV-Vis spectra were recorded using 2 mm (l = 0.2 cm) Suprasil Quartz cuvettes. All measurements were done using solutions of the specified concentration in anhydrous MeCN, stored inside an argon-filled glovebox, and using the same solvent in the blank. **3b** was generated *in situ* from **1** and **2b** (1.0 equiv.).

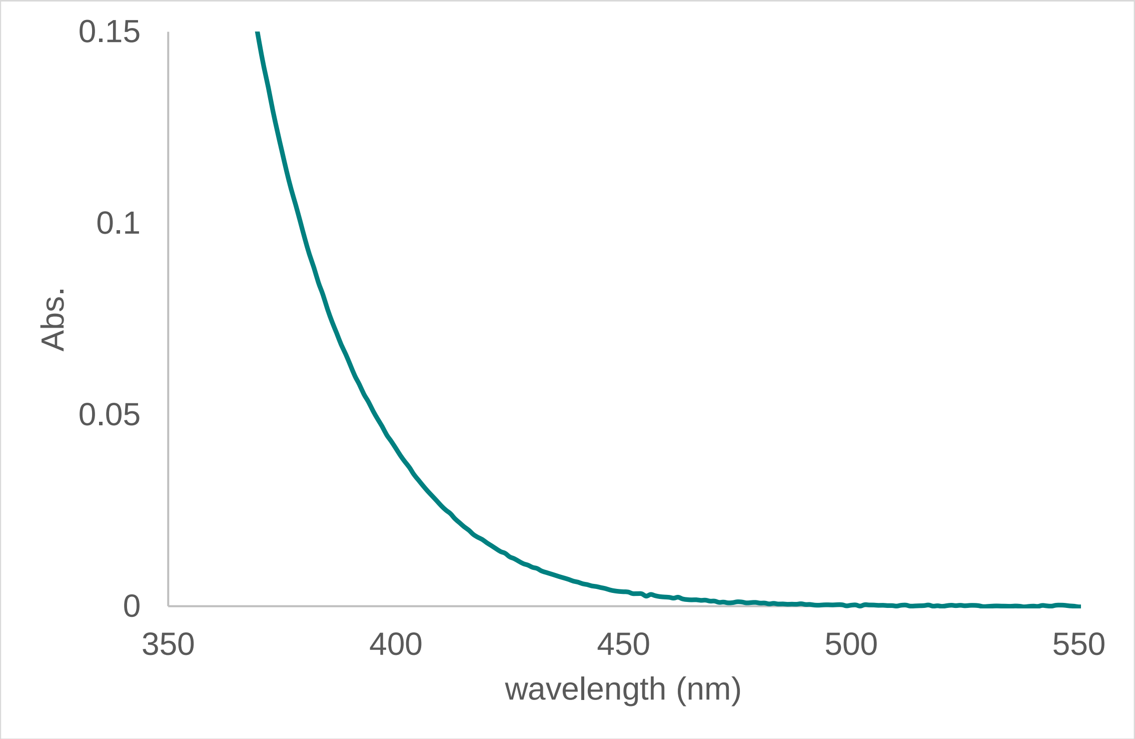

Figure. S6. UV-Vis absorbance spectrum of **3b** (1.0 mM, green trace) in MeCN.

6. Product Characterization

6.1. Characterization of intramolecular C–H amination products 4

5,7-diisopropyl-3,3-dimethyl-2,3-dihydrobenzo[*d*]isothiazole 1,1-dioxide (4a)

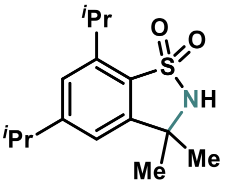

Following the general procedure, the reaction was performed with 2,4,6-triisopropylbenzenesulfonyl azide (62 mg, 0.20 mmol) and Bi complex **1** (0.90 mg, 0.0020 mmol, 1.0 mol%) in MeCN (0.050 M, 4.0 mL) for 24 h under 456 nm LEDs irradiation. The crude mixture was purified by preparative TLC (Et_2O/pentene = 1:1) to afford the product in 91% yield (51 mg, 0.18 mmol) as a white solid.

R_f = 0.40 (Et_2O/pentene = 1:1)

^{1}H NMR (600 MHz, $CDCl_3$, 298 K) δ 7.22 (d, *J* = 1.4 Hz, 1H), 6.98 (d, *J* = 1.4 Hz, 1H), 4.41 (s, 1H), 3.61 (quin, *J* = 6.8 Hz, 1H), 2.98 (quin, *J* = 6.9 Hz, 1H), 1.63 (s, 6H), 1.35 (d, *J* = 6.8 Hz, 6H), 1.28 (d, *J* = 6.9 Hz, 6H).

^{13}C NMR (151 MHz, $CDCl_3$, 298 K) δ 155.7, 146.8, 145.5, 131.0, 124.5, 117.9, 59.9, 34.8, 30.1, 29.6, 24.0, 23.7.

HRMS (ESI-positive): calc'd for $C_{15}H_{23}N_1O_2S_1Na_1$ $[M+Na]^+$: 304.13417, found: 304.13414.

The spectral data matched with those reported in the literature.[2]

7-isopropyl-3,3-dimethyl-2,3-dihydrobenzo[d]isothiazole 1,1-dioxide (4c)

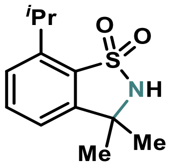

Following the general procedure, the reaction was performed with 2,6-diisopropylbenzenesulfonyl azide (54 mg, 0.20 mmol) and Bi complex **1** (9.0 mg, 0.020 mmol, 10 mol%) in MeCN (0.10 M, 2.0 mL) for 24 h under 456 nm LEDs irradiation. The crude mixture was purified by preparative TLC (Et_2O /pentene = 1:1) to afford the product in 81% yield (80.6 mg, 0.161 mmol) as a white solid.

R_f = 0.30 (Et_2O/pentene = 1:1)

^{1}H NMR (600 MHz, $CDCl_3$, 298 K) δ 7.56 (td, *J* = 7.7, 0.5 Hz, 1H), 7.39 (ddd, *J* = 7.7, 0.9, 0.5 Hz, 1H), 7.18 (dd, *J* = 7.7, 0.9 Hz, 1H), 4.49 (s, 1H), 3.64 (sept, *J* = 6.8 Hz, 1H), 1.64 (s, 6H), 1.35 (d, *J* = 6.8 Hz, 6H).

^{13}C NMR (151 MHz, $CDCl_3$, 298 K) δ 146.4, 145.7, 133.9, 133.2, 126.1, 120.2, 59.9, 30.0, 29.5, 23.7.

HRMS (ESI-positive): calc'd for $C_{12}H_{17}N_1O_2S_1Na_1$ $[M+Na]^+$: 262.08722, found: 262.08709.

6-isopropyl-3,3-dimethyl-2,3-dihydrobenzo[d]isothiazole 1,1-dioxide (4d)

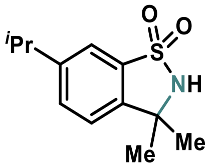

Following the general procedure, the reaction was performed with 2,5-diisopropylbenzenesulfonyl azide (54 mg, 0.20 mmol), CsI (52 mg, 0.20 mmol), and Bi complex **1** (9.0 mg, 0.020 mmol, 10 mol%) in DMSO (0.20 M, 1.0 mL) for 24 h under 456 nm LEDs irradiation. The crude mixture was purified by flash column chromatography (EtOAc/pentane = 1:9) to afford the product in 53% yield (25.6 mg, 0.107 mmol) as a white solid.

^{1}H NMR (600 MHz, $CDCl_3$, 298 K) δ 7.58 (dd, J = 1.5, 0.8 Hz, 1H), 7.48 (ddd, J = 8.0, 1.6, 0.5 Hz, 1H), 7.29 (dd, J = 8.1, 0.6 Hz, 1H), 4.56 (s, 1H), 3.01 (sept, J = 6.9 Hz, 1H), 1.64 (s, 6H), 1.28 (d, J = 7.0 Hz, 6H).

^{13}C NMR (151 MHz, $CDCl_3$, 298 K) δ 150.8, 143.7, 135.3, 132.4, 122.7, 118.7, 60.8, 34.2, 29.9, 23.9.

HRMS (ESI-positive): calc'd for $C_{12}H_{17}N_1O_2S_1Na_1$ $[M+Na]^+$: 262.08722, found: 262.08704.

The spectral data matched with those reported in the literature.[2]

6-cyclohexyl-2H-spiro[benzo[d]isothiazole-3,1'-cyclohexane] 1,1-dioxide (4e)

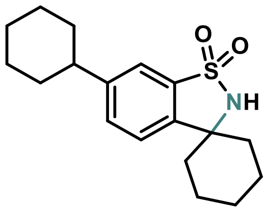

Following the general procedure, the reaction was performed with 2,5-dicyclohexyllbenzenesulfonyl azide (69.5 mg, 0.200 mmol) and Bi complex **1** (9.0 mg, 0.020 mmol, 10 mol%) in DMSO (0.10 M, 2.0 mL) for 24 h under 456 nm LEDs irradiation. The crude mixture was purified by preparative TLC (Et_2O /pentene = 1:1 and EtOAc/toluene = 2:8) to afford the product in 24% yield (15.3 mg, 0.0479 mmol) as a white solid.

R_f = 0.45 (pentene/Et_2O = 1:1)

^{1}H NMR (600 MHz, $CDCl_3$, 298 K) δ 7.56 (dt, J = 1.7, 0.6 Hz, 1H), 7.45 (ddd, J = 8.1, 1.7, 0.5 Hz, 1H), 7.28 (dd, J = 8.1, 0.6 Hz, 1H), 4.58 (s, 1H), 2.60 (m, 1H), 1.92–1.73 (m, 12H), 1.64–1.54 (m, 2H), 1.47–1.20 (m, 6H).

^{13}C NMR (151 MHz, $CDCl_3$, 298 K) δ 150.1, 143.7, 135.4, 132.6, 123.0, 119.2, 63.6, 44.4, 37.9, 34.4, 26.8, 26.1, 24.9, 22.7.

HRMS (GC-EI): calc'd for $C_{18}H_{25}N_1O_2S_1$ $[M]^+$: 319.160051, found: 319.160000.

The spectral data matched with those reported in the literature.[2]

3-(2-hydroxyethoxy)benzo[d]isothiazole 1,1-dioxide (4f)

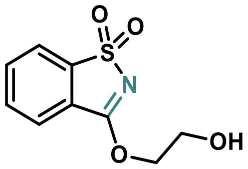

Following the general procedure, the reaction was performed with 2-(1,3-dioxolan-2-yl)benzenesulfonyl azide (51 mg, 0.20 mmol) and Bi complex **1** (9.0 mg, 0.020 mmol, 10 mol%) in MeCN (0.10 M, 2.0 mL) for 24 h under 456 nm LEDs irradiation. The crude mixture was purified by preparative TLC (DCM) to afford the ring-opened product in 72% yield (32.8 mg, 0.145 mmol) as a white solid.

R_f = 0.10 (DCM)

^{1}H NMR (600 MHz, $CDCl_3$, 298 K) δ 7.90 (dt, *J* = 7.6, 0.9 Hz, 1H), 7.81 (ddd, *J* = 7.5, 1.1, 0.7 Hz, 1H), 7.78 (td, *J* = 7.5, 1.1 Hz, 1H), 7.72 (td, *J* = 7.5, 1.0 Hz, 1H), 4.75–4.71 (m, 2H), 4.12–4.06 (m, 2H), 1.98 (s, 1H).

^{13}C NMR (151 MHz, $CDCl_3$, 298 K) δ 169.6, 143.7, 134.4, 133.7, 126.9, 123.7, 122.2, 73.3, 60.6.

HRMS (ESI-positive): calc'd for $C_9H_9N_1O_4S_1Na_1$ $[M+Na]^+$: 250.01445, found: 250.01422.

5,7-diethyl-3-methyl-2,3-dihydrobenzo[*d*]isothiazole 1,1-dioxide (4g)

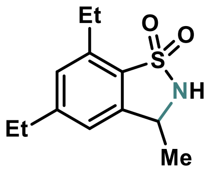

Following the general procedure, the reaction was performed with 2,4,6-triethylbenzenesulfonyl azide (54 mg, 0.20 mmol), NaI (30 mg, 0.20 mmol, 1.0 equiv.), and Bi complex **1** (0.90 mg, 0.0020 mmol, 1.0 mol%) in MeCN (0.050 M, 4.0 mL) for 24 h under 456 nm LEDs irradiation. The crude mixture was purified by preparative TLC (pentene/Et_2O = 1:1) to afford the product in 43% yield (20 mg, 0.085 mmol) as a colorless oil.

R_f = 0.20 (pentene/Et_2O = 1:1)

^{1}H NMR (600 MHz, $CDCl_3$, 298 K) δ 7.13 (dq, *J* = 1.3, 0.7 Hz, 1H), 6.98 (s, 1H), 4.72–4.66 (m, 1H), 4.51 (s, 1H), 3.03–2.96 (m, 2H), 2.74–2.66 (m, 2H), 1.59 (d, *J* = 6.7 Hz, 3H), 1.35 (t, *J* = 7.6 Hz, 3H), 1.26 (t, *J* = 7.6 Hz, 3H).

^{13}C NMR (151 MHz, $CDCl_3$, 298 K) δ 150.8, 142.6, 140.5, 131.6, 128.9, 120.4, 52.8, 29.2, 24.5, 21.8, 15.6, 14.8.

HRMS (ESI-positive): calc'd for $C_{12}H_{17}N_1O_2S_1Na_1$ $[M+Na]^+$: 262.08722, found: 262.08719.

The spectral data matched with those reported in the literature.[2]

6-ethyl-3-methyl-2,3-dihydrobenzo[d]isothiazole 1,1-dioxide (4h)

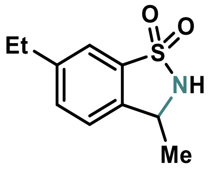

Following the general procedure, the reaction was performed with 2,5-diethylbenzenesulfonyl azide (48 mg, 0.20 mmol), CsI (52 mg, 0.20 mmol, 1.0 equiv.), and Bi complex **1** (9.0 mg, 0.020 mmol, 10 mol%) in MeCN (0.50 M, 0.40 mL) for 24 h under 456 nm LEDs irradiation. The reaction mixture was diluted with $CDCl_3$, and the yield was determined by ^{1}H NMR spectroscopy (13% yield) using trichloroethylene as an internal standard.

^{1}H NMR (600 MHz, $CDCl_3$, 298 K) δ 7.59 (s, 1H), 7.46 (m, 1H), 7.27 (m, 1H), 4.91 (brs, 1H), 4.76 (m, 1H), 4.51 (s, 1H), 2.74 (m, 2H), 1.58 (d, *J* = 6.7 Hz, 3H), 1.35 (t, *J* = 7.6 Hz, 3H), 1.26 (m, 3H).

The spectral data matched with those reported in the literature.[2]

6.2. Characterization of intermolecular C–H amination products 7

2,2,2-trichloroethyl-(1-methyl-5-(pyridin-3-yl)pyrrolidin-2-ylidene)carbamate (7a)

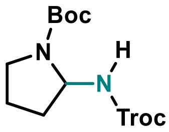

Following the general procedure, the reaction was performed with *N*-Boc-pyrrolidine (34.2 mg, 0.200 mmol), $TrocN_3$ (131 mg, 0.600 mmol, 3.00 equiv.) and **1** (9.0 mg, 0.020 mmol, 10 mol%) in MeCN (0.20 mL, 1.0 M) for 24 h under 456 nm LEDs irradiation. The crude mixture was purified by flash column chromatography (EtOAc/pentane = 1:9, with 1% of Et_3N) to afford the product in 77% yield (55.5 mg, 0.153 mmol) as a colorless oil.

R_f = 0.5 (EtOAc/Et_3N/pentane = 1:1:8, stained with Ninhydrin)

^{1}H NMR (600 MHz, $CDCl_3$, 298 K) δ 5.48 (brs, 1H), 5.18 (brs, 1H), 4.88–4.52 (m, 2H), 3.48 (s, 1H), 3.28 (s, 1H), 2.19–1.84 (m, 4H), 1.45 (s, 9H).

^{13}C NMR (151 MHz, $CDCl_3$, 298 K) δ 154.1, 153.1, 95.6, 80.6, 74.6, 65.9, 46.0, 32.6, 28.6, 22.4.

HRMS (EI): calc'd for $C_{12}H_{19}N_2O_4Cl_3Na_1$ $[M+Na]^+$: 383.030260, found: 383.030400.

The spectral data matched with those reported in the literature.[28]

benzyl 2-(((2,2,2-trichloroethoxy)carbonyl)amino)pyrrolidine-1-carboxylate (7b)

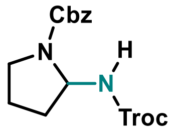

Following the general procedure, the reaction was performed with *N*-Cbz-pyrrolidine (41.1 mg, 0.200 mmol), $TrocN_3$ (0.13 g, 0.60 mmol, 3.0 equiv.) and **1** (9.0 mg, 0.020 mmol, 10 mol%) in MeCN (0.50 mL, 0.40 M) for 24 h under 456 nm LEDs irradiation. The crude mixture was purified by flash column chromatography (Et_2O/DCM = 5:95 to 10:90, with 1% of Et_3N) to afford the 2 rotamers (ratio ~ 1:0.8) in 42% yield (33 mg, 0.084 mmol) as a yellow oil.

R_f = 0.40 (Et_2O/DCM = 1:9)

HRMS (ESI positive): calc'd for $C_{15}H_{17}N_2O_4Cl_3Na_1$ $[M+Na]^+$: 417.01461, found: 417.01462.

NOTE: The rotamer exchange was almost completely frozen out at 243 K. Therefore, the sample was characterized at 243 K. The two rotamers were assigned by 2D NMR and 1D selective TOCSY spectroscopy.

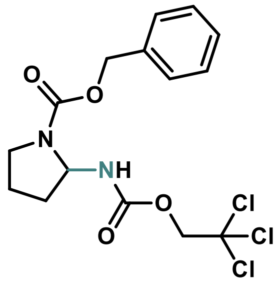

Major rotamer

^{1}H NMR (600 MHz, $CDCl_3$, 243 K) δ 7.40–7.30 (m, 5H), 5.60 (ddd, *J* = 8.1, 6.2, 1.6 Hz, 1H), 5.29 (d, *J* = 8.1 Hz, 1H), 5.19 (d, *J* = 12.3 Hz, 1H), 5.07 (d, *J* = 12.3 Hz, 1H), 4.61 (d, *J* = 12.0 Hz, 1H), 4.53 (d, *J* = 12.0 Hz, 1H), 3.59–3.51 (m, 1H), 3.33 (qd, *J* = 8.1, 2.6 Hz, 1H), 2.18–1.82 (m, 4H).

^{13}C NMR (151 MHz, $CDCl_3$, 243 K) δ 154.5, 152.85, 136.1, 128.56, 128.21, 128.16, 95.2, 74.1, 67.2, 65.4, 46.5, 33.9, 22.3.

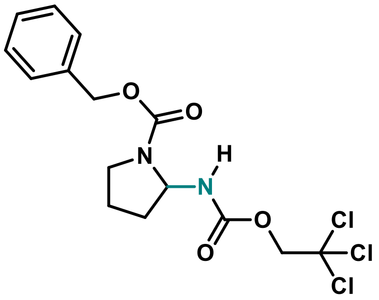

Minor rotamer

^{1}H NMR (600 MHz, $CDCl_3$, 243 K) δ 7.40–7.30 (m, 5H), 5.57 (td, *J* = 7.2, 2.3 Hz, 1H), 5.35 (d, *J* = 7.2 Hz, 1H), 5.14 (d, *J* = 12.3 Hz, 1H), 5.10 (d, *J* = 12.3 Hz, 1H), 4.83 (d, *J* = 12.0 Hz, 1H), 4.59 (d, *J* = 12.0 Hz, 1H), 3.59–3.51 (m, 1H), 3.33 (qd, *J* = 8.1, 2.6 Hz, 1H), 2.18–1.82 (m, 4H).

^{13}C NMR (151 MHz, $CDCl_3$, 243 K) δ 153.1, 152.85, 136.2, 128.61, 128.29, 128.27, 95.3, 74.2, 67.1, 66.2, 46.3, 32.6, 23.1.

2,2,2-trichloroethyl (1-acetylpyrrolidin-2-yl)carbamate (7c)

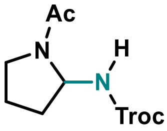

Following the general procedure, the reaction was performed with *N*-Ac-pyrrolidine (22.6 mg, 0.200 mmol), $TrocN_3$ (131 mg, 0.600 mmol, 3.00 equiv.), NaOEt (13.6 mg, 0.200 mmol, 1.00 equiv.), and **1** (9.0 mg, 0.020 mmol, 10 mol%) in MeCN (0.20 mL, 1.0 M) for 24 h under 456 nm LEDs irradiation. The crude mixture was purified by flash column chromatography (EtOAc/pentane = 1:9, with 1% of Et_3N) to afford the 2 rotamers (ratio ~ 3:1) in 55% yield (33.3 mg, 0.110 mmol) as a viscous yellow oil.

R_f = 0.3 (EtOAc/pentane = 1:1, stained with $KMnO_4$)

HRMS (ESI positive): calc'd for $C_9H_{13}N_2O_3Cl_3Na_1$ $[M+Na]^+$: 324.98840, found: 324.98838.

NOTE: The rotamer exchange was almost completely frozen out at 233 K. Therefore, the sample was characterized at 233 K. The two rotamers were assigned by 2D NMR and 1D selective TOCSY spectroscopy.

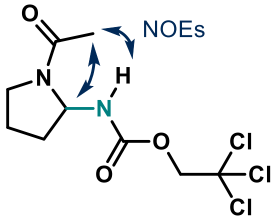

Major rotamer

^{1}H NMR (600 MHz, $CDCl_3$, 233 K) δ 6.50 (d, *J* = 8.9 Hz, 1H), 5.61 (dd, *J* = 8.9, 6.0 Hz, 1H), 4.90 (d, *J* = 12.1 Hz, 1H), 4.59 (d, *J* = 12.1 Hz, 1H), 3.60–3.55 (m, 1H), 3.34–3.27 (m, 1H), 2.17–2.11 (m, 1H), 2.14 (s, 3H), 2.17–2.09 (m, 1H), 2.04–1.93 (m, 2H).

^{13}C NMR (151 MHz, $CDCl_3$, 233 K) δ 170.9, 153.2, 95.3, 73.9, 66.1, 45.7, 34.4, 22.4, 21.5

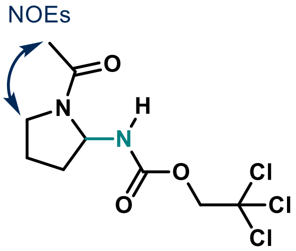

Minor rotamer

^{1}H NMR (600 MHz, $CDCl_3$, 233 K) δ 5.90 (d, *J* = 6.0 Hz, 1H), 5.53 (td, *J* = 6.5, 2.2 Hz, 1H), 4.75 (d, *J* = 12.0 Hz, 1H), 4.59 (d, *J* = 12.0 Hz, 1H), 3.64–3.60 (m, 1H), 3.43–3.37 (m, 1H), 2.17–2.11 (m, 1H), 2.07 (s, 3H), 2.04–1.93 (m, 3H).

^{13}C NMR (151 MHz, $CDCl_3$, 233 K) δ 170.5, 153.1, 95.2, 74.0, 65.0, 45.8, 31.6, 23.5, 23.2

2,2,2-trichloroethyl-2-(((2,2,2-trichloroethoxy)carbonyl)amino)pyrrolidine-1-carboxylate (7d)

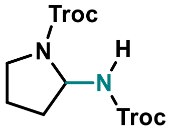

Following the general procedure, the reaction was performed with *N*-Troc-pyrrolidine (49.3 mg, 0.200 mmol), $TrocN_3$ (131 mg, 0.600 mmol, 3.00 equiv.), NaOEt (13.6 mg, 0.200 mmol, 1.00 equiv.), and **1** (9.0 mg, 0.020 mmol, 10 mol%) in MeCN (0.20 mL, 1.0 M) for 24 h under 456 nm LEDs irradiation. The crude mixture was purified by flash column chromatography (EtOAc/pentane = 5:95, with 1% of Et_3N) to afford the 2 rotamers (ratio ~ 2:1) in 24% yield (21.3 mg, 0.0487 mmol) as a colorless oil. R_f = 0.1 (EtOAc/pentane = 2:8, stained with $KMnO_4$).

HRMS (ESI positive): calc'd for $C_{10}H_{12}N_2O_4Cl_6Na_1$ $[M+Na]^+$: 456.88209, found: 456.88204.

NOTE: The rotamer exchange was almost completely frozen out at 233 K. Therefore, the sample was characterized at 233 K. The observed NOEs were not evident enough to assign the rotamers.

Major rotamer

^{1}H NMR (600 MHz, $CDCl_3$, 233 K) δ 5.66 (ddd, *J* = 7.8, 6.3, 1.3 Hz, 1H), 5.47 (d, *J* = 7.8 Hz, 1H), 4.95 (d, *J* = 12.0 Hz, 1H), 4.74 (d, *J* = 11.9 Hz, 1H), 4.57 (d, *J* = 11.8 Hz, 1H), 4.57 (d, *J* = 12.0 Hz, 1H), 3.62 (ddd, *J* = 10.4, 7.4, 2.8 Hz, 1H), 3.37 (td, *J* = 10.4, 7.1 Hz, 1H), 2.21–2.11 (m, 1H), 2.10–1.95 (m, 3H).

^{13}C NMR (151 MHz, $CDCl_3$, 233 K) δ 152.67, 152.55, 95.1, 95.0, 74.7, 74.3, 65.4, 46.8, 33.9, 22.2.

Minor rotamer

^{1}H NMR (600 MHz, $CDCl_3$, 233 K) δ 5.62 (td, *J* = 8.1, 1.9 Hz, 1H), 5.45 (d, *J* = 8.1 Hz, 1H), 4.84 (d, *J* = 12.0 Hz, 1H), 4.75 (d, *J* = 11.9 Hz, 1H), 4.69 (d, *J* = 11.9 Hz, 1H), 4.59 (d, *J* = 12.0 Hz, 1H), 3.65 (ddd, *J* = 10.2, 6.7, 4.0 Hz, 1H), 3.49–3.41 (m, 1H), 2.21–2.11 (m, 1H), 2.10–1.95 (m, 3H).

^{13}C NMR (151 MHz, $CDCl_3$, 233 K) δ 153.1, 152.74, 95.2 (two peaks are overlapped), 74.6, 74.2, 66.0, 46.3, 32.6, 23.0.

***tert*-butyl-1-(((2,2,2-trichloroethoxy)carbonyl)amino)hexahydrocyclopenta[c]pyrrole-2(1H)-carboxylate (7e)**

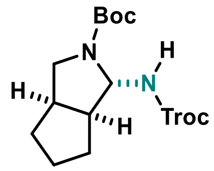

Following the general procedure, the reaction was performed with *tert*-butyl-hexahydrocyclopenta[c]pyrrole-2(1H)-carboxylate (42.3 mg, 0.200 mmol), $TrocN_3$ (131 mg, 0.600 mmol, 3.00 equiv.), and **1** (9.0 mg, 0.020 mmol, 10 mol%) in MeCN (0.20 mL, 1.0 M) for 24 h under 456 nm LEDs irradiation. The diastereomeric ratio was determined to be a >5:1 ration by ^{1}H NMR spectroscopy of the crude reaction mixture. The crude mixture was purified by flash column chromatography (EtOAc/pentane = 0:100 to 5:95, with 1% of Et_3N) to afford the product in 53% yield (42.6 mg, 0.106 mmol) as a white solid.

R_f = 0.4 (EtOAc/pentane = 2:8, stained with $KMnO_4$)

NOTE: At room temperature, the signals appeared slightly broadened; therefore, the sample was characterized at 333 K, where most signals appeared significantly sharper.

^{1}H NMR (600 MHz, $CDCl_3$, 333 K) δ 5.24 (brs, 1H), 5.19 (d, *J* = 6.5 Hz, 1H), 4.72 (s, 2H), 3.63 (dd, *J* = 11.2, 8.5 Hz, 1H), 3.23 (dd, *J* = 11.2, 5.0 Hz, 1H), 2.78 (dtdd, *J* = 8.5, 7.7, 5.0, 4.3 Hz, 1H), 2.67 (q, *J* = 7.9 Hz, 1H), 2.04–1.95 (m, 1H), 1.89–1.80 (m, 1H), 1.79–1.69 (m, 1H), 1.63–1.47 (m, 3H), 1.46 (s, 9H).

^{13}C NMR (151 MHz, $CDCl_3$, 333 K) δ 154.2, 153.4, 95.9, 80.5, 74.8, 72.5, 52.7, 52.0, 41.1, 32.6, 31.6, 28.6, 25.6.

HRMS (ESI positive): calc'd for $C_{15}H_{23}N_2O_4Cl_3Na_1$ $[M+Na]^+$: 423.061560, found: 423.061710.

The spectral data matched with those reported in the literature.[29]

Notably, NH and H1 appear to be affected by cross-relaxation, leading to shared NOEs; as a result, these NOEs could not be unambiguously interpreted. At lower temperature (233 K), two rotameric forms of the compound are observed. Although exchange cross-peaks between the two rotamers are present, the overall NOE patterns could still be distinguished at this temperature. In particular, strong NOEs from NH-101 to H6, H7′, and H2, as well as from H1 to H3′, are observed, which are consistent with the relative stereochemistry shown above.

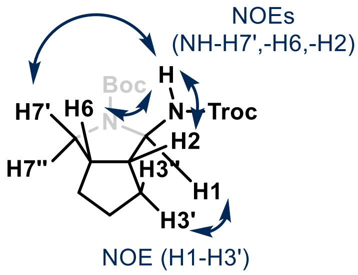

tert-butyl-1-(((2,2,2-trichloroethoxy)carbonyl)amino)octahydro-2H-isoindole-2-carboxylate (7f)

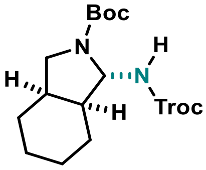

Following the general procedure, the reaction was performed with *tert*-butyl-octahydro-2H-isoindole-2-carboxylate (45.1 mg, 0.200 mmol), $TrocN_3$ (131 mg, 0.600 mmol, 3.00 equiv.), and **1** (9.0 mg, 0.020 mmol, 10 mol%) in MeCN (0.20 mL, 1.0 M) for 24 h under 456 nm LEDs irradiation. The diastereomeric ratio was determined to be a >5:1 ration by ^1H NMR spectroscopy of the crude reaction mixture. The crude mixture was purified by flash column chromatography (EtOAc/cyclohexane = 3:97 to 10:90, with 1% of Et_3N) to afford *mono*- and *bis*-aminated product (ratio ~ 7:1) as a white solid

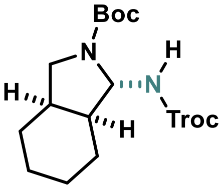

Mono-aminated product

50% yield (41.2 mg, 0.0989 mmol)

R_f = 0.3 (EtOAc/cyclohexane = 2:8, stained with $KMnO_4$)

NOTE: At room temperature, the signals appeared slightly broadened; therefore, the sample was characterized at 383 K, where most signals appeared significantly sharper.

^{1}H NMR (600 MHz, DMSO-*d*$_6$, 383 K) δ 7.63 (s, 1H), 5.08 (dd, *J* = 8.0, 3.5 Hz, 1H), 4.79 (d, *J* = 12.1 Hz, 1H), 4.74 (d, *J* = 12.3 Hz, 1H), 3.33 (dd, *J* = 10.3, 7.4 Hz, 1H), 3.16 (dd, *J* = 10.3, 7.3 Hz, 1H), 2.44–2.36 (m, 1H), 2.05–1.98 (m, 1H), 1.63–1.20 (m, 8H), 1.41 (s, 9H).

^{13}C NMR (151 MHz, DMSO-*d*$_6$, 383 K) δ 153.3, 152.5, 95.9, 78.2, 73.3, 69.3, 48.1, 44.3, 32.8, 27.7, 24.2, 23.9, 22.2, 21.1.

HRMS (ESI positive): calc'd for $C_{16}H_{25}N_2O_4Cl_3Na_1$ $[M+Na]^+$: 437.077210, found: 437.077710.

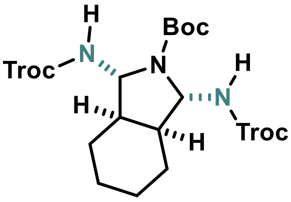

Bis-aminated product

7% yield (8.56 mg, 0.0142 mmol)

R_f = 0.3 (EtOAc/cyclohexane = 2:8, stained with $KMnO_4$)

NOTE: At room temperature, the signals appeared slightly broadened; therefore, the sample was characterized at 383 K, where most signals appeared significantly sharper.

^{1}H NMR (600 MHz, DMSO-*d*$_6$, 383 K) δ 7.49 (s, 2H), 5.14 (dd, *J* = 8.0, 3.8 Hz, 2H), 4.79 (m, 4H), 2.17–2.12 (m, 2H), 1.63–1.20 (m, 8H), 1.41 (s, 9H).

^{13}C NMR (151 MHz, DMSO-*d*$_6$, 383 K) δ 152.6, 152.5, 95.7, 79.4, 73.4, 68.6, 41.9, 27.5, 23.3, 21.5.

HRMS (ESI positive): calc'd for $C_{19}H_{27}N_3O_6Cl_6Na_1$ $[M+Na]^+$: 625.99232, found: 625.99209.

tert-butyl-2-(((2,2,2-trichloroethoxy)carbonyl)amino)piperidine-1-carboxylate (7g)

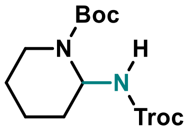

Following the general procedure, the reaction was performed with *N*-Boc-piperidine (37.1 mg, 0.200 mmol), $TrocN_3$ (131 mg, 0.600 mmol, 3.00 equiv.), NaOEt (13.6 mg, 0.200 mmol, 1.00 equiv.), and **1** (9.0 mg, 0.020 mmol, 10 mol%) in MeCN (0.20 mL, 1.0 M) for 24 h under 456 nm LEDs irradiation. The crude mixture was purified by flash column chromatography (EtOAc/pentane = 1:9, with 1% of Et_3N) to afford the product in 60% yield (45.1 mg, 0.120 mmol) as a white solid.

R_f = 0.2 (EtOAc/cyclohexane = 1:9, stained with Ninhydrin)

^{1}H NMR (600 MHz, $CDCl_3$, 298 K) δ 5.95 (dt, *J* = 7.4, 3.5 Hz, 1H), 5.49 (brs, 1H), 4.78–4.65 (m, 2H), 3.98 (d, *J* = 13.7 Hz, 1H), 2.84 (t, *J* = 13.5 Hz, 1H), 1.85 (d, *J* = 13.5 Hz, 1H), 1.78–1.70 (m, 2H), 1.68 (d, *J* = 12.2 Hz, 1H), 1.58–1.48 (m, 2H), 1.46 (s, 9H).

^{13}C NMR (151 MHz, $CDCl_3$, 298 K) δ 154.5, 153.2, 95.6, 80.6, 74.7, 59.7, 39.5, 29.9, 28.4, 24.8, 18.9.

HRMS (ESI positive): calc'd for $C_{13}H_{21}N_2O_4Cl_3Na_1$ $[M+Na]^+$: 397.045910, found: 397.045990.

The spectral data matched with those reported in the literature.[28]

benzyl-2-(((2,2,2-trichloroethoxy)carbonyl)amino)piperidine-1-carboxylate (7h)

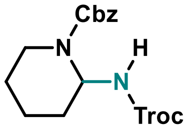

Following the general procedure, the reaction was performed with *N*-Cbz-piperidine (43.6 mg, 0.200 mmol), $TrocN_3$ (131 mg, 0.600 mmol, 3.00 equiv.), NaOEt (13.6 mg, 0.200 mmol, 1.00 equiv.), and **1** (9.0 mg, 0.020 mmol, 10 mol%) in MeCN (0.20 mL, 1.0 M) for 24 h under 456 nm LEDs irradiation. The crude mixture was purified by flash column chromatography (EtOAc/pentane = 3:97 to 10:90) to afford the product in 48% yield (39 mg, 0.096 mmol) as a viscous colorless oil.

R_f = 0.4 (EtOAc/pentane = 2:8, stained with $KMnO_4$)

NOTE: At room temperature, the signals appeared slightly broadened; therefore, the sample was characterized at 323 K, where most signals appeared significantly sharper.

^{1}H NMR (600 MHz, $CDCl_3$, 323 K) δ 7.40–7.36 (m, 2H), 7.38–7.32 (m, 2H), 7.32–7.28 (m, 1H), 6.06–5.99 (m, 1H), 5.41 (brs, 1H), 5.25–5.10 (m, 2H), 4.76–4.67 (m, 2H), 4.06 (m, *J* = 13.7 Hz, 1H), 2.97 (td, *J* = 13.7, 3.0 Hz, 1H), 1.92–1.85 (m, 1H), 1.80–1.72 (m, 2H), 1.69 (d, *J* = 13.6 Hz, 1H), 1.64–1.49 (m, 2H).

^{13}C NMR (151 MHz, $CDCl_3$, 323 K) δ 155.2, 153.3, 136.8, 128.7, 128.2, 128.1, 95.7, 74.9, 67.7, 60.0, 39.9, 29.8, 24.9, 18.9.

HRMS (ESI positive): calc'd for $C_{16}H_{19}N_2O_4Cl_3Na_1$ $[M+Na]^+$: 431.03026, found: 431.03092.

2,2,2-trichloroethyl-(1-acetylpiperidin-2-yl)carbamate (7i)

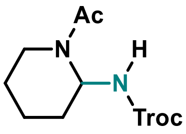

Following the general procedure, the reaction was performed with *N*-Ac-piperidine (25.4 mg, 0.200 mmol), $TrocN_3$ (131 mg, 0.600 mmol, 3.00 equiv.), NaOEt (13.6 mg, 0.200 mmol, 1.00 equiv.), and **1** (9.0 mg, 0.020 mmol, 10 mol%) in MeCN (0.20 mL, 1.0 M) for 24 h under 456 nm LEDs irradiation. The crude mixture was purified by flash column chromatography (EtOAc/pentane = 1:9 to 3:7) to afford the 2 rotamers (ratio ~ 5:1) in 36% yield (23.1 mg, 0.073 mmol) as a white solid.

R_f = 0.3 (EtOAc/pentane = 1:1, stained with $KMnO_4$)

HRMS (ESI positive): calc'd for $C_{10}H_{15}N_2O_3Cl_3Na_1$ $[M+Na]^+$: 339.004045, found: 339.004340.

NOTE: The rotamer exchange was almost completely frozen out at 233 K. Therefore, the sample was characterized at 233 K. The two rotamers were assigned by 2D NMR spectroscopy.

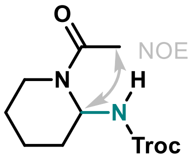

Major rotamer

^{1}H NMR (600 MHz, $CDCl_3$, 233 K) δ 5.92 (d, *J* = 8.0 Hz, 1H), 5.72–5.67 (m, 1H), 4.83 (d, *J* = 12.0 Hz, 1H), 4.60 (d, *J* = 12.0 Hz, 1H), 4.52 (m, *J* = 13.7 Hz, 1H), 2.67 (td, *J* = 13.5, 2.8 Hz, 1H), 2.34 (s, 3H), 1.95–1.69 (m, 4H), 1.65–1.54 (m, 1H), 1.49–1.38 (m, 1H).

^{13}C NMR (151 MHz, $CDCl_3$, 233 K) δ 169.8, 153.5, 94.9, 74.35, 61.1, 36.1, 30.1, 24.6, 21.8, 18.91.

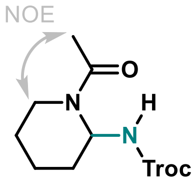

Minor rotamer

^{1}H NMR (600 MHz, $CDCl_3$, 233 K) δ 6.46–6.41 (m, 1H), 5.66 (d, *J* = 7.0 Hz, 1H), 4.79 (d, *J* = 12.0 Hz, 1H), 4.60 (d, *J* = 12.0 Hz, 1H), 3.64 (d, *J* = 13.9 Hz, 1H), 3.24 (ddd, *J* = 13.9, 12.8, 2.7 Hz, 1H), 2.11 (s, 3H), 1.95–1.69 (m, 4H), 1.65–1.54 (m, 1H), 1.49–1.38 (m, 1H).

^{13}C NMR (151 MHz, $CDCl_3$, 233 K) δ 169.3, 153.1, 95.1, 74.35, 55.8, 41.8, 28.9, 25.5, 22.4, 18.88.

2,2,2-trichloroethyl 2-(((2,2,2-trichloroethoxy)carbonyl)amino)piperidine-1-carboxylate (7j)

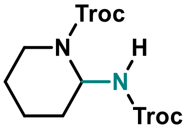

Following the general procedure, the reaction was performed with *N*-Troc-piperidine (50.1 mg, 0.200 mmol), $TrocN_3$ (131 mg, 0.600 mmol, 3.00 equiv.), Cs_2CO_3 (65.2 mg, 0.200 mmol, 1.00 equiv.), and **1** (9.0 mg, 0.020 mmol, 10 mol%) in MeCN (0.20 mL, 1.0 M) for 24 h under 456 nm LEDs irradiation. The crude mixture was purified by flash column chromatography (EtOAc/pentane = 3:97) to afford the product in 41% yield (36.5 mg, 0.0810 mmol) as a pale-yellow viscous solid.

R_f = 0.4 (EtOAc/pentane = 2:8, stained with $KMnO_4$)

NOTE: At room temperature, the signals appeared slightly broadened; therefore, the sample was characterized at 333 K, where most signals appeared significantly sharper.

^{1}H NMR (600 MHz, $CDCl_3$, 333 K) δ 6.03 (ddd, *J* = 7.3, 4.2, 2.7 Hz, 1H), 5.44 (brs, 1H), 4.84–4.69 (m, 4H), 4.11–4.04 (m, 1H), 3.11–3.03 (m, 1H), 1.96–1.90 (m, 1H), 1.87–1.70 (m, 3H), 1.68–1.53 (m, 2H).

^{13}C NMR (151 MHz, $CDCl_3$, 333 K) δ 153.4, 153.3, 95.9, 95.6, 75.6, 75.1, 60.4, 40.3, 29.7, 24.9, 18.9.

HRMS (ESI positive): calc'd for $C_{11}H_{14}N_2O_4Cl_6Na_1$ $[M+Na]^+$: 470.89769, found: 470.89800.

***tert*-butyl-2-(((2,2,2-trichloroethoxy)carbonyl)amino)azepane-1-carboxylate (7k)**

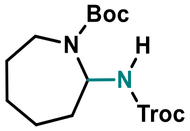

Following the general procedure, the reaction was performed with *N*-Boc-azepane (39.9 mg, 0.200 mmol), $TrocN_3$ (131 mg, 0.600 mmol, 3.00 equiv.), Cs_2CO_3 (16.3 mg, 0.200 mmol, 1.00 equiv.), and **1** (9.0 mg, 0.020 mmol, 10 mol%) in MeCN (0.40 mL, 0.50 M) for 24 h under 456 nm LEDs irradiation. The crude mixture was purified by flash column chromatography (EtOAc/pentane = 0:10 to 1:9, with 1% of Et_3N) to afford the product in 36% yield (28 mg, 0.071 mmol) as a white solid.

R_f = 0.5 (EtOAc/pentane = 2:8, stained with $KMnO_4$)

NOTE: At room temperature, two sets of broadened signals were observed, indicating the presence of rotamers. At 373 K, only a single, averaged set of signals was detected. Therefore, the sample was characterized at 373 K.

^{1}H NMR (600 MHz, DMSO-*d*$_6$, 373 K) δ 7.68 (brd, *J* = 7.9 Hz, 1H), 5.58 (dt, *J* = 10.3, 7.3 Hz, 1H), 4.79 (d, *J* = 12.3 Hz, 1H), 4.75 (d, *J* = 12.3 Hz, 1H), 3.59 (brd, *J* = 14.6 Hz, 1H), 3.08 (ddd, *J* = 14.6, 11.1, 2.1 Hz, 1H), 2.09 (dt, *J* = 14.8, 7.4 Hz, 1H), 1.75–1.64 (m, 3H), 1.66–1.58 (m, 1H), 1.45–1.39 (m, 10H), 1.36–1.27 (m, 1H), 1.22–1.13 (m, 1H).

^{13}C NMR (151 MHz, DMSO-*d*$_6$, 373 K) δ 153.8, 152.5, 95.9, 78.4, 73.3, 63.1, 40.9, 33.3, 28.0, 27.7, 27.6, 23.0.

HRMS (ESI positive): calc'd for $C_{14}H_{23}N_2O_4Cl_3Na_1$ $[M+Na]^+$: 411.06156, found: 411.06162.

***tert*-butyl-4-cyano-2-(((2,2,2-trichloroethoxy)carbonyl)amino)piperidine-1-carboxylate (7l)**

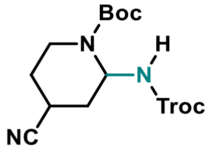

Following the general procedure, the reaction was performed with 4-cyano-*N*-Boc-piperidine (42.1 mg, 0.200 mmol), $TrocN_3$ (131 mg, 0.600 mmol, 3.00 equiv.), NaOEt (13.6 mg, 0.200 mmol, 1.00 equiv.), and **1** (9.0 mg, 0.020 mmol, 10 mol%) in MeCN (0.20 mL, 1.0 M) for 24 h under 456 nm LEDs irradiation. The diastereomeric ratio was determined to be a 2:1 ration by ^{1}H NMR spectroscopy of the crude reaction mixture. The crude mixture was purified by flash column chromatography (EtOAc/pentane = 5:95 to 20:80), and the two diastereomers were isolated separately in 53% combined yield.

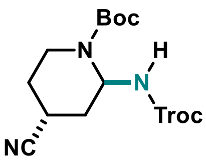

Major diastereomer

34% yield (27.6 mg, 0.069 mmol), a white solid

R_f = 0.50 (EtOAc/pentane = 3:7, stained with Ninhydrin)

NOTE: At room temperature, the signals were slightly broadened; therefore, the sample was characterized at 323 K, where most signals appeared significantly sharper.

^{1}H NMR (600 MHz, $CDCl_3$, 323 K) δ 5.95 (ddd, J = 6.1, 4.3, 2.7 Hz, 1H), 5.20 (brd, J = 6.1 Hz, 1H), 4.76 (brd, J = 12.0 Hz, 1H), 4.71 (d, J = 12.0 Hz, 1H), 4.11 (ddd, J = 14.2, 4.8, 2.7 Hz, 1H), 2.88 (td, J = 14.2, 3.2 Hz, 1H), 2.87 (tt, J = 12.4, 3.8 Hz, 1H), 2.41 (dm, J = 13.9 Hz, 1H), 2.10 (dp, J = 13.5, 3.8 Hz, 1H), 1.98 (ddd, J = 13.9, 12.7, 4.3 Hz, 1H), 1.79 (qd, J = 13.4, 4.6 Hz, 1H), 1.48 (s, 9H).

^{13}C NMR (151 MHz, $CDCl_3$, 323 K) δ 153.9, 153.4, 120.9, 95.5, 81.9, 75.0, 59.1, 38.3, 32.9, 28.5, 28.4, 22.6

HRMS (ESI positive): calc'd for $C_{14}H_{20}N_3O_4Cl_3Na_1$ $[M+Na]^+$: 422.04116, found: 422.04133.

The relative stereochemistry was established based on NOEs from NH100 H3/H5ax, consistent with all of them occupying axial positions in the ring. Additional support comes from NOEs between H1 and both H2ax and H2eq, as well as from the large coupling constant between H2ax and H3 (~12.4 Hz, both axial) and the much smaller coupling between H2ax and H1 (4.3 Hz, *cis* relationship).

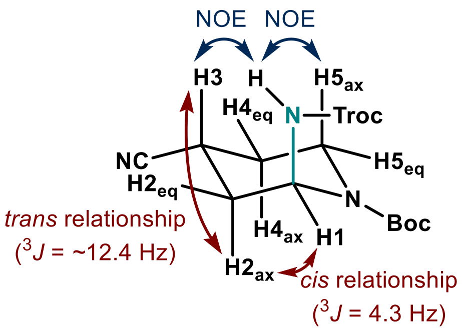

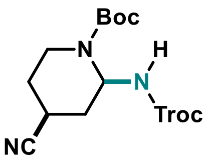

Minor diastereomer

19% yield (15.1 mg, 0.038 mmol), a viscous yellow oil

R_f = 0.25 (EtOAc/pentane = 3:7, stained with Ninhydrin)

NOTE: At room temperature, the signals were slightly broadened; therefore, the sample was characterized at 323 K, where most signals appeared significantly sharper.

^{1}H NMR (600 MHz, $CDCl_3$, 323 K) δ 5.98–5.90 (m, 2H), 4.81 (brd, J = 12.0 Hz, 1H), 4.70 (d, J = 12.0 Hz, 1H), 4.05 (dt, J = 14.4, 3.9 Hz, 1H), 3.29 (ddd, J = 14.5, 12.1, 3.0 Hz, 1H), 3.01 (tt, J = 4.8, 3.8 Hz, 1H), 2.29 (ddq, J = 14.4, 3.5, 1.7 Hz, 1H), 2.02 (dt, J = 14.4, 5.0 Hz, 1H), 2.00 (dqd, J = 13.8, 3.3, 1.7 Hz, 1H), 1.82 (ddt, J = 13.8, 12.1, 4.7 Hz, 1H), 1.47 (s, 9H).

^{13}C NMR (151 MHz, $CDCl_3$, 323 K) δ 154.1, 153.4, 121.7, 95.6, 81.7, 74.9, 59.3, 36.6, 31.5, 28.4, 27.5, 22.3.

HRMS (ESI positive): calc'd for $C_{14}H_{20}N_3O_4Cl_3Na_1$ $[M+Na]^+$: 422.04116, found: 422.04145.

Unfortunately, based on the assignment data, H1 and NH100 overlap across the measured temperature range and likely form a more complex spin system. The relative stereochemistry was therefore established by comparison to previously assigned samples and by analysis of the observed J values. The coupling constants of H3 to its neighbors are all < 6 Hz, which is indicative for a more dominantly equatorial orientation. Furthermore, the similar coupling constants of H2ax to H3 and H1 (~5 Hz) support a 1,3-*cis* configuration.

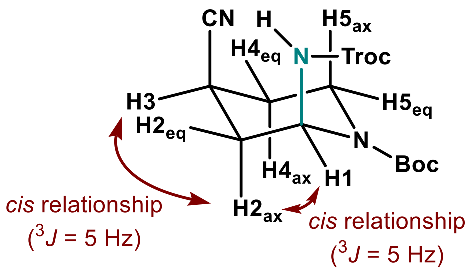

***tert*-butyl-4-hydroxy-2-(((2,2,2-trichloroethoxy)carbonyl)amino)piperidine-1-carboxylate (7m)**

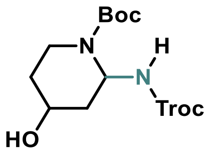

Following the general procedure, the reaction was performed with 4-hydroxy-*N*-Boc-piperidine (40.3 mg, 0.200 mmol), $TrocN_3$ (131 mg, 0.600 mmol, 3.00 equiv.), and **1** (9.0 mg, 0.020 mmol, 10 mol%) in MeCN (0.20 mL, 1.0 M) for 24 h under 456 nm LEDs irradiation. The diastereomeric ratio was determined to be a 4:1 ratio by ^{1}H NMR spectroscopy of the crude reaction mixture. The crude mixture was purified by flash column chromatography (EtOAc/pentane = 1:9 to 3:7), and the two diastereomers were isolated separately in 35% combined yield.

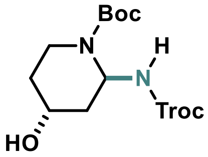

Major diastereomer

26% yield (20 mg, 0.051 mmol), a viscous oil

R_f = 0.2 (EtOAc/pentane = 4:6, stained with Ninhydrin)

NOTE: At room temperature, the signals were slightly broadened; therefore, the sample was characterized at 323 K, where most signals appeared significantly sharper.

^{1}H NMR (600 MHz, $CDCl_3$, 323 K) δ 6.05 (dtd, J = 7.4, 4.6, 1.0 Hz, 1H), 5.30 (brs, 1H), 4.79–4.67 (m, 2H), 4.10 (dt, J = 14.1, 4.0 Hz, 1H), 3.97 (tt, J = 11.4, 4.5 Hz, 1H), 2.92 (td, J = 14.1, 2.9 Hz, 1H), 2.22 (ddt, J = 13.2, 4.5, 2.4 Hz, 1H), 1.99 (ddq, J = 12.7, 4.8, 2.7 Hz, 1H), 1.66 (ddd, J = 13.2, 11.4, 4.6 Hz, 1H), 1.52 (brs, 1H), 1.48 (s, 9H), 1.47 (tdd, J = 12.7, 10.8, 4.8 Hz, 1H).

^{13}C NMR (151 MHz, $CDCl_3$, 323 K) δ 154.2, 153.3, 95.7, 81.1, 74.9, 65.0, 60.7, 39.0, 38.2, 34.5, 28.5.

HRMS (ESI positive): calc'd for $C_{13}H_{21}N_2O_5Cl_3Na_1$ $[M+Na]^+$: 413.040825, found: 413.04080.

The relative stereochemistry was established based on NOEs from NH to H3 and H5ax. Two large coupling constants of H3 to its neighbors, H4ax (10.8 Hz) and H2ax (11.4 Hz), are typical for a dominantly axial orientation. Furthermore, the significantly different coupling constants of H2ax to H1 (4.6 Hz) also support a 1,3-*trans* configuration.

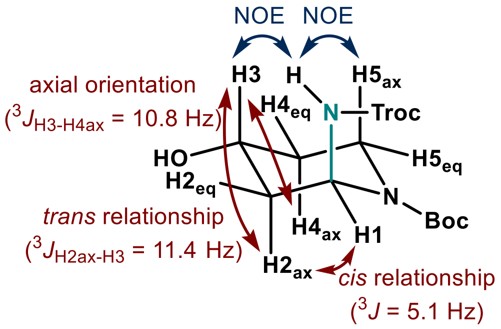

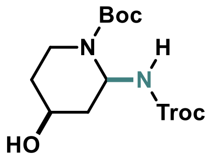

Minor diastereomer

9% yield (7.3 mg, 0.019 mmol), a viscous oil

R_f = 0.25 (EtOAc/pentane = 4:6, stained with Ninhydrin)

NOTE: At room temperature, the signals were slightly broadened; therefore, the sample was characterized at 323 K, where most signals appeared significantly sharper.

^{1}H NMR (600 MHz, CDCl$_3$, 323 K) δ 6.91 (d, J = 8.9 Hz, 1H), 6.05 (ddd, J = 8.9, 5.1, 2.4 Hz, 1H), 4.72 (s, 2H), 4.33 (m, 1H), 3.96–3.90 (m, 1H), 3.33 (td, J = 14.1, 3.8 Hz, 1H), 1.97 (ddt, J = 14.4, 3.2, 2.0 Hz, 1H), 1.89 (ddd, J = 14.4, 5.1, 2.8 Hz, 1H), 1.82–1.70 (m, 3H), 1.49 (s, 9H).

^{13}C NMR (151 MHz, CDCl$_3$, 323 K) δ 154.3, 153.4, 95.9, 80.6, 74.8, 65.4, 59.1, 35.5, 33.1, 32.5, 28.5.

HRMS (ESI positive): calc'd for $C_{13}H_{21}N_2O_5Cl_3Na_1$ [M+Na]$^+$: 413.040825, found: 413.041090.

The relative stereochemistry was established based on the ^3J coupling of H2ax to H3 and H1 (2.8 Hz / 5.1 Hz). These values are relatively small due to the cis relationship, but they likely differ slightly because of contributions from different ring conformations (e.g., chair vs. boat). Additional support comes from weak NOEs between H5ax/NH and H3′/H_2O.

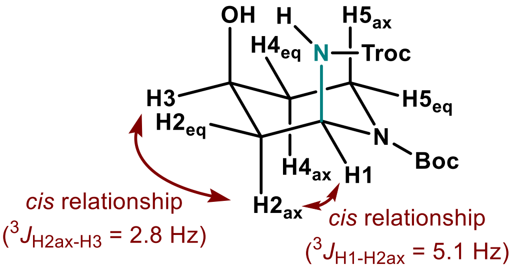

tert-butyl-4-bromo-2-(((2,2,2-trichloroethoxy)carbonyl)amino)piperidine-1-carboxylate (7n)

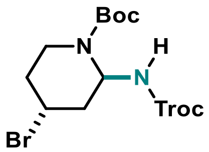

Following the general procedure, the reaction was performed 4-bromo-*N*-Boc-piperidine (42.3 mg, 0.200 mmol), $TrocN_3$ (131 mg, 0.600 mmol, 3.00 equiv.), NaOEt (13.6 mg, 0.200 mmol, 1.00 equiv.), and **1** (9.0 mg, 0.020 mmol, 10 mol%) in MeCN (0.20 mL, 1.0 M) for 24 h under 456 nm LEDs irradiation. The diastereomeric ratio was determined to be a >5:1 ratio by ^{1}H NMR spectroscopy of the crude reaction mixture. The crude mixture was purified by flash column chromatography (EtOAc/pentane = 0:10 to 5:95) to afford the major diastereomer in 32% yield (29.2 mg, 0.064 mmol) as a yellow solid.

R_f = 0.6 (EtOAc/pentane = 2:8, stained with Ninhydrin)

NOTE: At room temperature, the signals appeared slightly broadened; therefore, the sample was characterized at 323 K, where most signals appeared significantly sharper.

^{1}H NMR (600 MHz, $CDCl_3$, 323 K) δ 5.93 (ddt, *J* = 6.9, 4.6, 2.6 Hz, 1H), 5.29 (brs, 1H), 4.75 (d, *J* = 12.0 Hz, 1H), 4.72 (d, *J* = 12.0 Hz, 1H), 4.24 (ddt, *J* = 12.4, 11.7, 4.2 Hz, 1H), 4.06 (ddd, *J* = 14.2, 4.8, 1.1 Hz, 1H), 2.94 (ddd, *J* = 14.2, 12.6, 2.9 Hz, 1H), 2.54 (ddt, *J* = 13.6, 4.2, 2.0 Hz, 1H), 2.29 (dqd, *J* = 13.1, 4.2, 2.9 Hz, 1H), 2.17 (ddd, *J* = 13.6, 12.4, 4.6 Hz, 1H), 1.97 (tdd, *J* = 13.1, 11.7, 4.8 Hz, 1H), 1.48 (s, 9H).

^{13}C NMR (151 MHz, $CDCl_3$, 323 K) δ 154.1, 153.3, 95.6, 81.5, 75.0, 61.5, 42.8, 41.1, 40.3, 36.7, 28.4.

HRMS (ESI positive): calc'd for $C_{13}H_{20}N_2O_4Cl_3Br_1Na_1$ $[M+Na]^+$: 474.95642, found: 474.95659.

The relative stereochemistry was established based on NOEs from H3 to H100 and H5ax. Additional support comes from NOEs between H1 and H5eq, originating from a minor ring conformer. Two large coupling constants of H3 to its neighbors, H4ax (11.7 Hz) and H2ax (12.4 Hz), are typical for a dominantly axial orientation. The ^4J coupling (1.1 Hz) between H1 and H5eq is characteristic of a dominant equatorial orientation. Furthermore, the significantly different coupling constants of H2ax to H3 and H1 (4.6 Hz) also support a 1,3-*trans* configuration.

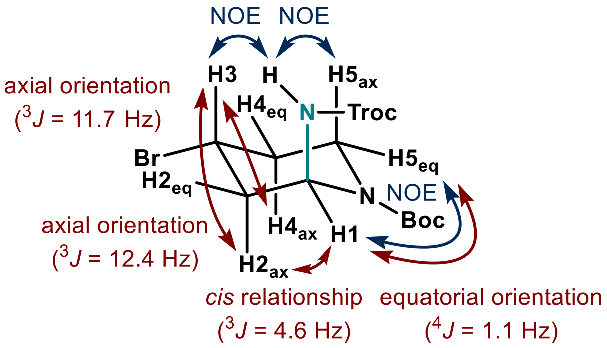

***tert*-butyl-4-benzyl-2-(((2,2,2-trichloroethoxy)carbonyl)amino)piperidine-1-carboxylate (7o)**

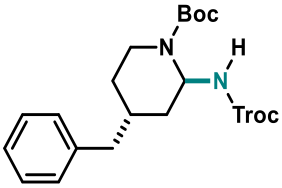

Following the general procedure, the reaction was performed 4-benzyl-*N*-Boc-piperidine (55.1 mg, 0.200 mmol), $TrocN_3$ (131 mg, 0.600 mmol, 3.00 equiv.), NaOEt (13.6 mg, 0.200 mmol, 1.00 equiv.), and **1** (9.0 mg, 0.020 mmol, 10 mol%) in MeCN (0.20 mL, 1.0 M) for 24 h under 456 nm LEDs irradiation. The diastereomeric ratio was determined to be a >5:1 ratio by ^{1}H NMR spectroscopy of the crude reaction mixture. The crude mixture was purified by flash column chromatography (EtOAc/pentane = 0:100 to 5:95) to afford the major diastereomer in 40% yield (38 mg, 0.081 mmol) as a white solid.

R_f = 0.5 (EtOAc/pentane = 2:8, stained with $KMnO_4$ and Ninhydrin)

NOTE: At room temperature, the signals appeared slightly broadened; therefore, the sample was characterized at 333 K, where most signals appeared significantly sharper.

^{1}H NMR (600 MHz, $CDCl_3$, 333 K) δ 7.30–7.26 (m, 2H), 7.22–7.19 (m, 1H), 7.14–7.11 (m, 2H), 6.01 (dt, *J* = 7.5, 4.4 Hz, 1H), 5.24 (s, 1H), 4.71 (m, 2H), 4.04 (d, *J* = 14.0 Hz, 1H), 2.79 (td, *J* = 13.5, 2.7 Hz, 1H), 2.61–2.50 (m, 2H), 1.94–1.81 (m, 2H), 1.69 (dp, *J* = 13.0, 2.7 Hz, 1H), 1.48–1.46 (m, 10H), 1.19 (tdd, *J* = 13.1, 11.9, 4.8 Hz, 1H).

^{13}C NMR (151 MHz, $CDCl_3$, 333 K) δ 154.3, 153.3, 139.5, 129.3, 128.6, 126.5, 95.8, 80.7, 74.9, 60.0, 43.3, 39.3, 36.6, 32.7, 31.4, 28.5.

HRMS (ESI positive): calc'd for $C_{20}H_{27}N_2O_4Cl_3Na_1$ $[M+Na]^+$: 487.09286, found: 487.09280.

The relative stereochemistry was established based on NOEs from NH to H3 and H5ax. Additional support comes from weak NOEs between H1 and H5eq, originating from contributions of a minor conformer. Two large coupling constants of H3 to its neighbors, H4ax (11.9 Hz) and H2ax (12.8 Hz), are typical for a dominantly axial orientation. Furthermore, the significantly different coupling constants of H2ax to H3 and H1 (4.4 Hz) also support a 1,3-*trans* configuration.

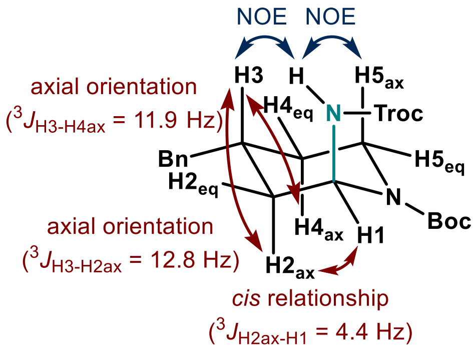

tert-butyl-4-phenyl-2-(((2,2,2-trichloroethoxy)carbonyl)amino)piperidine-1-carboxylate (7p)

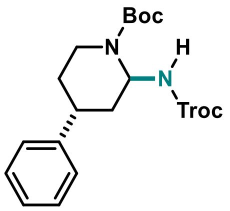

Following the general procedure, the reaction was performed 4-phenyl-*N*-Boc-piperidine (52.3 mg, 0.200 mmol), $TrocN_3$ (131 mg, 0.600 mmol, 3.00 equiv.), NaOEt (13.6 mg, 0.200 mmol, 1.00 equiv.), and **1** (9.0 mg, 0.020 mmol, 10 mol%) in MeCN (0.20 mL, 1.0 M) for 24 h under 456 nm LEDs irradiation. The diastereomeric ratio was not assigned due to the complexity of ^{1}H NMR spectrum arising from the rotameric nature of each diastereomer of the product. The crude mixture was purified by flash column chromatography (EtOAc/pentane = 5:95) to afford the major diastereomer in 38% yield (35 mg, 0.077 mmol) as a white solid.

R_f = 0.4 (EtOAc/pentane = 2:8, stained with Ninhydrin)

NOTE: At room temperature, the signals appeared slightly broadened; therefore, the sample was characterized at 333 K, where most signals appeared significantly sharper.

^{1}H NMR (600 MHz, $CDCl_3$, 333 K) δ 7.34–7.30 (m, 2H), 7.24–7.21 (m, 1H), 7.21–7.19 (m, 2H), 6.12 (ddd, *J* = 7.3, 4.3, 2.3 Hz, 1H), 5.43 (bs, 1H), 4.78 (d, *J* = 12.0 Hz, 1H), 4.74 (d, *J* = 12.0 Hz, 1H), 4.19 (d, *J* = 13.9 Hz, 1H), 3.00 (td, *J* = 13.4, 2.8 Hz, 1H), 2.87 (tt, *J* = 12.7, 3.5 Hz, 1H), 2.13 (d, *J* = 13.5 Hz, 1H), 1.96–1.88 (m, 2H), 1.69 (qd, *J* = 12.9, 4.6 Hz, 1H), 1.51 (s, 9H).

^{13}C NMR (151 MHz, $CDCl_3$, 333 K) δ 154.4, 153.4, 144.6, 128.9, 126.9, 126.9, 95.8, 81.0, 75.0, 60.3, 39.7, 37.4, 36.9, 32.7, 28.5.

HRMS (ESI positive): calc'd for $C_{19}H_{25}N_2O_4Cl_3Na_1$ $[M+Na]^+$: 473.07721, found: 473.07745.

The relative stereochemistry was established based on NOEs from H3 to NH and H5ax. Two large coupling constants of H3 (~12.7 Hz) to its neighbors, H4ax and H2ax, are typical for a dominantly axial orientation. The significantly different coupling constants of H2ax to H3 and H1 (4.2 Hz) also support a 1,3- *trans* configuration.

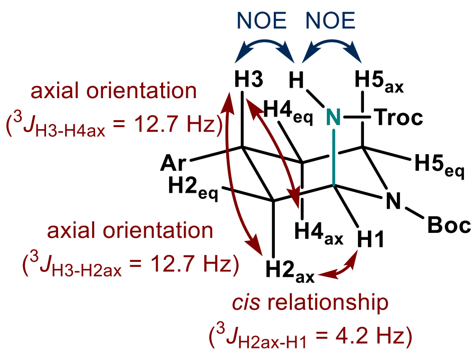

tert-butyl-4-(methoxy(methyl)carbamoyl)-2-(((2,2,2-trichloroethoxy)carbonyl)amino)piperidine-1-carboxylate (7q)

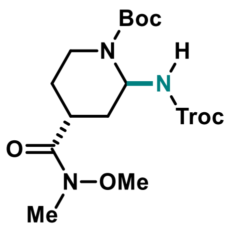

Following the general procedure, the reaction was performed with 4-(*N*-methoxy-*N*-methylcarbamoyl)-*N*-Boc-piperidine (54.5 mg, 0.200 mmol), $TrocN_3$ (131 mg, 0.600 mmol, 3.00 equiv.), NaOEt (13.6 mg, 0.200 mmol, 1.00 equiv.), and **1** (9.0 mg, 0.020 mmol, 10 mol%) in MeCN (0.20 mL, 1.0 M) for 24 h under 456 nm LEDs irradiation. The diastereomeric ratio was determined to be a 3.6:1 ratio by ^{1}H NMR spectroscopy of the crude reaction mixture. The crude mixture was purified by flash column chromatography (EtOAc/pentane = 1:9 to 3:7) to afford the major diastereomer (2 rotamers, ratio ~ 3:1) in 36% yield (33.5 mg, 0.072 mmol) as a white solid.

R_f = 0.6 (EtOAc/pentane = 6:4, stained with Ninhydrin)

HRMS (ESI positive): calc'd for $C_{16}H_{26}N_3O_6Cl_3Na_1$ $[M+Na]^+$: 484.07794, found: 484.07822.

NOTE: The rotamer exchange was almost completely frozen out at 233 K. Therefore, the sample was characterized at 233 K. The two rotamers were assigned by 2D NMR spectroscopy.

Major rotamer

^{1}H NMR (600 MHz, $CDCl_3$, 233 K) δ 6.07 (ddd, *J* = 8.0, 4.6, 2.3 Hz, 1H), 5.60 (d, *J* = 8.0 Hz, 1H), 4.75 (d, *J* = 12.1 Hz, 1H), 4.71 (d, *J* = 12.1 Hz, 1H), 4.13 (dt, *J* = 13.7, 4.6 Hz, 1H), 3.74 (s, 3H), 3.20 (s, 3H,), 3.02 (tt, *J* = 12.6, 3.7 Hz, 1H), 2.85 (td, *J* = 13.7, 3.1 Hz, 1H), 1.99 (td, *J* = 13.6, 4.4 Hz, 1H), 1.90 (d, *J* = 13.8 Hz, 1H), 1.82 (bd, *J* = 13.5 Hz, 1H), 1.66 (qd, *J* = 13.2, 4.8 Hz, 1H), 1.45 (s, 9H).

^{13}C NMR (151 MHz, $CDCl_3$, 233 K) δ 174.5, 154.2, 152.9, 95.3, 81.1, 74.16, 61.91, 58.9, 37.8, 32.36, 32.13, 32.05, 28.2, 27.3.

Minor rotamer

^{1}H NMR (600 MHz, $CDCl_3$, 233 K) δ 6.09 (ddd, *J* = 6.2, 4.6, 2.3 Hz, 1H, H1), 5.40 (d, *J* = 6.3 Hz, 1H), 4.87 (d, *J* = 12.1 Hz, 1H), 4.56 (d, *J* = 12.1 Hz, 1H), 4.04 (ddd, *J* = 13.7, 4.5, 2.4 Hz, 1H), 3.71 (s, 3H), 3.19 (s, 3H), 3.07 (tt, *J* = 12.4, 3.7 Hz, 1H), 2.95 (td, *J* = 13.4, 3.3 Hz, 1H), 2.07 (d, *J* = 13.8 Hz, 1H), 1.89 (td, *J* = 13.4, 4.1 Hz, 1H), 1.77–1.64 (m, 2H), 1.44 (s, 9H).

^{13}C NMR (151 MHz, $CDCl_3$, 233 K) δ 174.7, 153.9, 153.0, 95.2, 80.9, 74.24, 61.85, 58.3, 39.0, 32.35, 32.12, 31.7, 28.3, 27.1.

The relative stereochemistry was established based on observed NOEs of NH to H3 and H5ax as well the couplings of H2ax to H3 (12.6 Hz) and to H1 (4.6 Hz).

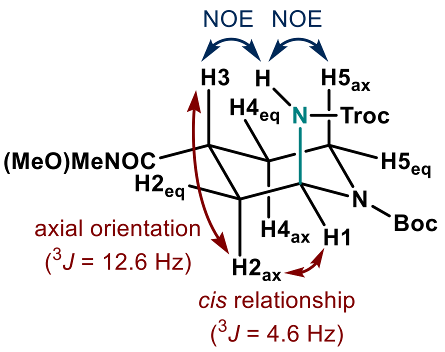

***tert*-butyls-4-(4-bromo-1H-pyrazol-1-yl)-2-(((2,2,2-trichloroethoxy)carbonyl)amino)piperidine-1-carboxylate (7r)**

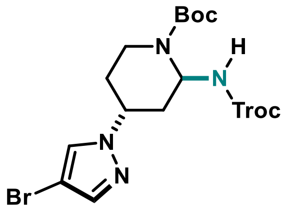

Following the general procedure, the reaction was performed with *tert*-butyl-4-(4-bromopyrazol-1-yl)piperidin-1-carboxylat (66.0 mg, 0.200 mmol), $TrocN_3$ (131 mg, 0.600 mmol, 3.00 equiv.), NaOEt (13.6 mg, 0.200 mmol, 1.00 equiv.), and **1** (9.0 mg, 0.020 mmol, 10 mol%) in MeCN (0.20 mL, 1.0 M) for 24 h under 456 nm LEDs irradiation. The diastereomeric ratio was determined to be a >5:1 ratio by ^{1}H NMR spectroscopy of the crude reaction mixture. The crude mixture was purified by flash column chromatography (EtOAc/pentane = 3:97 to 15:85) to afford the product in 54% yield (56 mg, 0.11 mmol) as a white solid.

R_f = 0.25 (EtOAc/pentane = 3:7, stained with Ninhydrin)

NOTE: At room temperature, the signals appeared slightly broadened; therefore, the sample was characterized at 333 K, where the ^{13}C NMR signals appeared significantly sharper.

^{1}H NMR (600 MHz, $CDCl_3$, 333 K) δ 7.46 (s, 1H), 7.42 (s, 1H), 6.12 (td, *J* = 6.7, 2.3 Hz, 1H), 5.35 (d, *J* = 6.7 Hz, 1H), 4.77 (d, *J* = 12.0 Hz, 1H), 4.72 (d, *J* = 12.0 Hz, 1H), 4.50 (tt, *J* = 12.2, 3.9 Hz, 1H), 4.24 (d, *J* = 13.9 Hz, 1H), 3.05 (td, *J* = 13.4, 3.0 Hz, 1H), 2.46 (ddt, *J* = 13.5, 3.9, 2.3 Hz, 1H), 2.20–2.13 (m, 2H), 1.96 (tdd, *J* = 12.8, 12.2, 4.8 Hz, 1H), 1.50 (s, 9H).

^{13}C NMR (151 MHz, $CDCl_3$, 333 K) δ 154.0, 153.4, 140.1, 127.3, 95.6, 93.4, 81.6, 75.0, 60.3, 55.8, 38.6, 36.4, 32.0, 28.5.

HRMS (ESI positive): calc'd for $C_{16}H_{22}N_4O_4Cl_3Br_1Na_1$ $[M+Na]^+$: 540.97822, found: 540.97873.

tert-butyl-7-(((2,2,2-trichloroethoxy)carbonyl)amino)-1,4-dioxa-8-azaspiro[4.5]decane-8-carboxylate (7s)

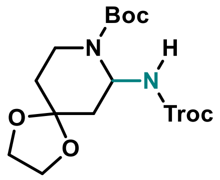

Following the general procedure, the reaction was performed with *tert*-butyl-1,4-dioxa-8-azaspiro[4.5]decane-8-carboxylate (48.7 mg, 0.200 mmol), $TrocN_3$ (131 mg, 0.600 mmol, 3.00 equiv.), NaOEt (13.6 mg, 0.200 mmol, 1.00 equiv.), and **1** (9.0 mg, 0.020 mmol, 10 mol%) in MeCN (0.20 mL, 1.0 M) for 24 h under 456 nm LEDs irradiation. The crude mixture was purified by flash column chromatography (EtOAc/pentane = 0:10 to 1:9, with 1% of Et_3N) to afford the product in 40% yield (35 mg, 0.081 mmol) as a white solid.

R_f = 0.1 (EtOAc/pentane = 2:8, stained with Ninhydrin)

NOTE: At room temperature, the signals appeared slightly broadened; therefore, the sample was characterized at 323 K, where the ^{13}C NMR signals appeared significantly sharper.

^1H NMR (600 MHz, $CDCl_3$, 323 K) δ 6.60 (brs, 1H), 6.21–6.14 (m, 1H), 4.73 (brs, 2H), 4.13–3.96 (m, 5H), 3.16–3.04 (m, 1H), 1.98 (dd, *J* = 13.8, 5.0 Hz, 1H), 1.82 (dt, *J* = 13.8, 1.9 Hz, 1H), 1.75–1.65 (m, 2H), 1.49 (s, 9H).

^{13}C NMR (151 MHz, $CDCl_3$, 323 K) δ 154.0, 153.3, 107.4, 95.9, 80.8, 74.8, 65.0, 64.5, 59.7, 38.4, 36.7, 34.4, 28.5.

HRMS (ESI positive): calc'd for $C_{15}H_{23}N_2O_6Cl_3Na_1$ $[M+Na]^+$: 455.05139, found: 455.05141.

tert-butyl-5-(((2,2,2-trichloroethoxy)carbonyl)amino)-6-azaspiro[2.5]octane-6-carboxylate (7t)

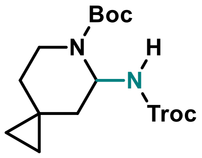

Following the general procedure, the reaction was performed with *tert*-butyl 6-azaspiro[2.5]octane-6-carboxylate (42.3 mg, 0.200 mmol), $TrocN_3$ (131 mg, 0.600 mmol, 3.00 equiv.), Cs_2CO_3 (65.2 mg, 0.200 mmol, 1.00 equiv.), and **1** (9.0 mg, 0.020 mmol, 10 mol%) in MeCN (0.20 mL, 1.0 M) for 24 h under 456 nm LEDs irradiation. The crude mixture was purified by flash column chromatography (EtOAc/pentane = 0:10 to 1:9) to afford the product in 40% yield (32 mg, 0.079 mmol) as a viscous oil.

R_f = 0.6 (EtOAc/pentane = 2:8, stained with Ninhydrin)

^1H NMR (600 MHz, $CDCl_3$, 298 K) δ 6.05 (dt, *J* = 8.8, 4.5 Hz, 1H), 5.82 (d, *J* = 8.8 Hz, 1H), 4.75 (d, *J* = 12.0 Hz, 1H), 4.69 (d, *J* = 12.0 Hz, 1H), 4.03 (d, *J* = 13.6 Hz, 1H), 2.99 (t, *J* = 13.3 Hz, 1H), 2.21 (dd, *J* = 13.9, 4.5 Hz, 1H), 1.93 (td, *J* = 13.3, 4.6 Hz, 1H), 1.47 (s, 9H), 1.10 (d, *J* = 13.9 Hz, 1H), 0.87 (d, *J* = 13.7 Hz, 1H), 0.55–0.44 (m, 2H), 0.39–0.28 (m, 2H).

^{13}C NMR (151 MHz, $CDCl_3$, 298 K) δ 154.5, 153.1, 95.7, 80.6, 74.6, 60.4, 38.54, 38.48, 34.1, 28.4, 13.6, 12.1, 9.3.

HRMS (ESI positive): calc'd for $C_{15}H_{23}N_2O_4Cl_3Na_1$ $[M+Na]^+$: 423.061561, found: 423.061460.

tert-butyl-ethyl(1-(((2,2,2-trichloroethoxy)carbonyl)amino)ethyl)carbamate (7u)

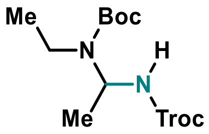

Following the general procedure, the reaction was performed with *tert*-butyl diethylcarbamate (42.3 mg, 0.200 mmol), $TrocN_3$ (131 mg, 0.600 mmol, 3.00 equiv.), Cs_2CO_3 (65.2 mg, 0.200 mmol, 1.00 equiv.), and **1** (9.0 mg, 0.020 mmol, 10 mol%) in MeCN (0.20 mL, 1.0 M) for 24 h under 456 nm LEDs irradiation. The crude mixture was purified by flash column chromatography (EtOAc/pentane = 0:10 to 1:9) to afford the product in 33% yield (24 mg, 0.066 mmol) as a colorless oil.

R_f = 0.6 (EtOAc/pentane = 2:8, stained with Ninhydrin)

NOTE: At room temperature, the signals appeared broadened; therefore, the sample was characterized at 333 K, where the ^1H and ^{13}C NMR spectra showed significantly sharper signals.

^{1}H NMR (600 MHz, $CDCl_3$, 333 K) δ 6.10 (brs, 1H), 5.13 (m, 1H), 4.73 (AB system, *J* = 12.3 Hz, 2H), 3.32 (dq, *J* = 14.3, 7.1 Hz, 1H), 3.24 (dq, *J* = 14.3, 7.1 Hz, 1H), 1.53 (d, *J* = 6.7 Hz, 3H), 1.48 (s, 9H), 1.14 (t, *J* = 7.1 Hz, 3H).

^{13}C NMR (151 MHz, $CDCl_3$, 333 K) δ 154.8, 153.7, 95.8, 80.4, 74.9, 63.4, 42.8, 28.7, 20.5, 14.7.

HRMS (ESI positive): calc'd for $C_{12}H_{21}N_2O_4Cl_3Na_1$ $[M+Na]^+$: 385.045910, found: 385.046090.

tert-butyl-4-(methoxy(methyl)carbamoyl)-2-(((2,2,2-trichloroethoxy)carbonyl)amino)piperidine-1-carboxylate (7v)

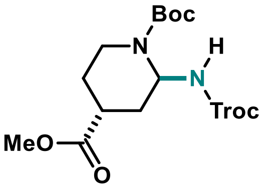

Following the general procedure, the reaction was performed with 1-*tert*-butyl-4-methylpiperidin-1,4-dicarboxylate (54.5 mg, 0.200 mmol), $TrocN_3$ (131 mg, 0.600 mmol, 3.00 equiv.), NaOEt (13.6 mg, 0.200 mmol, 1.00 equiv.), and **1** (9.0 mg, 0.020 mmol, 10 mol%) in MeCN (0.20 mL, 1.0 M) for 24 h under 456 nm LEDs irradiation. The diastereomeric ratio was determined to be a >5:1 ratio by ^{1}H NMR spectroscopy of the crude reaction mixture. The crude mixture was purified by flash column chromatography (EtOAc/pentane = 1:9 to 2:8) to afford the major diastereomer in 36% yield (31 mg, 0.072 mmol) as a white solid.

R_f = 0.25 (EtOAc/pentane = 2:8, stained with Ninhydrin)

NOTE: At room temperature, the signals appeared slightly broadened. Therefore, the sample was characterized at 323 K, where the signals appeared significantly sharper.

^{1}H NMR (600 MHz, $CDCl_3$, 323 K) δ 6.04 (ddd, *J* = 6.9, 4.3, 2.5 Hz, 1H), 5.24 (brs, 1H), 4.76 (d, *J* = 12.0 Hz, 1H), 4.71 (d, *J* = 12.0 Hz, 1H), 4.11 (ddd, *J* = 14.1, 4.7, 2.4 Hz, 1H), 3.70 (s, 3H), 2.87 (td, *J* = 14.1, 3.0 Hz, 1H), 2.63 (tt, *J* = 12.6, 3.7 Hz, 1H), 2.21 (brd, *J* = 13.9 Hz, 1H), 1.99 (dp, *J* = 13.4, 3.7 Hz, 1H), 1.87 (ddd, *J* = 13.9, 13.0, 4.3 Hz, 1H), 1.64 (dddd, *J* = 13.4, 13.0, 12.3, 4.7 Hz, 1H), 1.48 (s, 9H).

^{13}C NMR (151 MHz, $CDCl_3$, 323 K) δ 174.5, 154.2, 153.3, 95.7, 81.2, 74.9, 59.5, 52.1, 38.6, 36.4, 32.4, 28.5, 27.7.

HRMS (ESI positive): calc'd for $C_{15}H_{23}N_2O_6Cl_3Na_1$ $[M+Na]^+$: 455.05139, found: 455.05122.

The relative stereochemistry was established based on NOEs from H3 to NH and H5ax. Two large coupling constants of H3 to its neighbors, H4ax (12.4 Hz) and H2ax (13.0 Hz), are typical for a dominantly axial orientation. Furthermore, the significantly different coupling constants of H2ax to H3 and H1 (4.3 Hz) also support a 1,3-*trans* configuration.

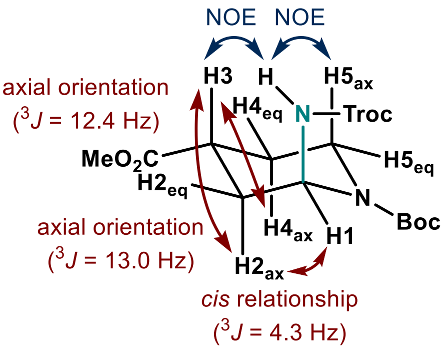

tert-butyl-4-(6-fluorobenzo[d]isoxazol-3-yl)-2-(((2,2,2-trichloroethoxy)carbonyl)amino)piperidine-1-carboxylate (7w)

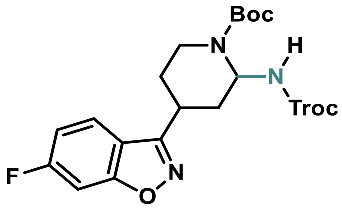

Following the general procedure, the reaction was performed with *tert*-butyl 4-(6-fluorobenzo[d]isoxazol-3-yl)piperidine-1-carboxylate (64.1 mg, 0.200 mmol), $TrocN_3$ (131 mg, 0.600 mmol, 3.00 equiv.), NaOEt (13.6 mg, 0.200 mmol, 1.00 equiv.), and **1** (9.0 mg, 0.020 mmol, 10 mol%) in MeCN (0.20 mL, 1.0 M) for 24 h under 456 nm LEDs irradiation. The diastereomeric ratio was determined to be a >5:1 ratio by ^{1}H NMR spectroscopy of the crude reaction mixture. The crude mixture was purified by flash column chromatography (EtOAc/pentane = 5:95 to 20:80), and the two diastereomers were isolated separately in 44% combined yield.

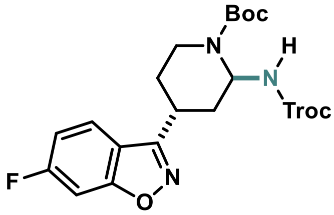

Major diastereomer

37% yield (36.5 mg, 0.0715 mmol), a white solid

R_f = 0.4 (EtOAc/pentane = 2:8, stained with Ninhydrin)

NOTE: At room temperature, the signals appeared slightly broadened. Therefore, the sample was characterized at 323 K, where the signals appeared significantly sharper.

^{1}H NMR (600 MHz, $CDCl_3$, 323 K) δ 7.64 (ddd, *J* = 8.7, 5.0, 0.5 Hz, 1H), 7.25 (ddd, *J* = 8.4, 2.1, 0.5 Hz, 1H), 7.08 (td, *J* = 8.8, 2.1 Hz, 1H), 6.13 (dt, *J* = 6.6, 4.1 Hz, 1H), 5.44 (d, *J* = 6.6 Hz, 1H), 4.81–4.74 (m, 2H), 4.25 (d, *J* = 14.1 Hz, 1H), 3.46 (tt, *J* = 12.7, 3.5 Hz, 1H), 3.09 (td, *J* = 13.4, 2.9 Hz, 1H), 2.48 (d, *J* = 13.7 Hz, 1H), 2.22–2.12 (m, 2H), 1.98 (qd, *J* = 12.9, 4.7 Hz, 1H), 1.51 (s, 9H).

^{13}C NMR (151 MHz, $CDCl_3$, 323 K) δ 164.5 (d, *J* = 251.5 Hz), 164.4 (d, *J* = 13.6 Hz), 160.1, 154.2, 153.4, 122.3 (d, *J* = 11.1 Hz), 117.2, 112.9 (d, *J* = 25.5 Hz), 97.8 (d, *J* = 26.8 Hz), 95.7, 81.4, 74.9, 59.9, 39.2, 34.4, 29.8, 29.6, 28.5.

^{19}F NMR (565 MHz, $CDCl_3$, 323 K) δ −109.1 (td, *J* = 8.6, 5.0 Hz),

HRMS (ESI positive): calc'd for $C_{20}H_{23}N_3O_5F_1Cl_3Na_1$ $[M+Na]^+$: 532.05795, found: 532.05791.

The relative stereochemistry was established based on NOEs from H3 to NH and H5ax, consistent with all of them occupying axial positions in the ring. Additional support comes from NOEs between H1 and both H2ax and H2eq, as well as from the large coupling constant between H2ax and H3 (12.7 Hz, both axial) and the much smaller coupling between H2ax and H1 (4.1 Hz, *cis* relationship).

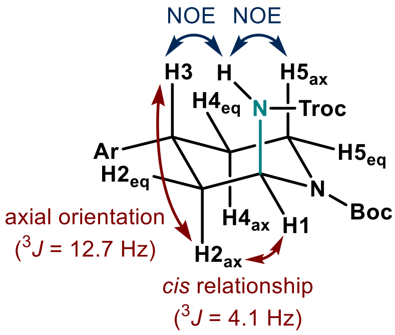

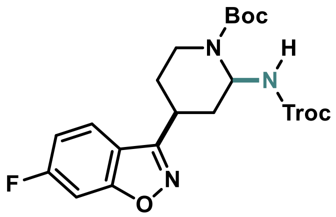

Minor diastereomer

7% yield (7.1 mg, 0.014 mmol), a white solid

R_f = 0.4 (EtOAc/pentane = 2:8, stained with Ninhydrin)

NOTE: At room temperature, the signals appeared slightly broadened. Therefore, the sample was characterized at 323 K, where the signals appeared significantly sharper.

^{1}H NMR (600 MHz, $CDCl_3$, 323 K) δ 7.59 (dd, *J* = 8.7, 4.9 Hz, 1H), 7.28 (ddd, *J* = 8.3, 2.1, 0.5 Hz, 1H), 7.26 (m, 1H), 7.10 (td, *J* = 8.8, 2.1 Hz, 1H), 5.93 (ddd, *J* = 9.0, 5.4, 4.0 Hz, 1H), 4.72–4.64 (m, 2H), 3.89 (dt, *J* = 14.2, 4.3 Hz, 1H), 3.62 (tt, *J* = 5.8, 4.3 Hz, 1H), 3.30 (ddd, *J* = 14.3, 11.2, 3.2 Hz, 1H), 2.40 (dt, *J* = 14.5, 5.8 Hz, 1H), 2.32 (dtd, *J* = 14.5, 4.4, 1.1 Hz, 1H), 2.13 (ddt, *J* = 13.8, 11.3, 5.8 Hz, 1H), 2.03 (dqd, J = 13.8, 4.3, 1.1 Hz, 1H), 1.51 (s, 9H).

^{13}C NMR (151 MHz, $CDCl_3$, 323 K) δ 164.8 (d, *J* = 252.1 Hz), 164.0 (d, *J* = 13.7 Hz), 160.7, 154.7, 153.6, 122.2 (d, *J* = 11.1 Hz), 117.4, 113.2 (d, *J* = 25.5 Hz), 97.9 (d, *J* = 26.9 Hz), 95.9, 81.0, 74.7, 59.6, 37.1, 31.9, 29.2, 28.5, 28.2.

^{19}F NMR (565 MHz, $CDCl_3$, 323 K) δ −108.3 (td, *J* = 8.6, 5.1 Hz)

HRMS (ESI positive): calc'd for $C_{20}H_{23}N_3O_5F_1Cl_3Na_1$ $[M+Na]^+$: 532.05795, found: 532.05844.

The relative stereochemistry was established based on NOEs from H3 to H1 and H5eq, originating from a minor ring conformer. Additional support comes from NOEs between NH and H5ax, as well as NOEs of H3 to both the equatorial and axial protons at positions 2 and 3. The coupling constants of H3 to its neighbors are all < 6 Hz, which is typical for a dominantly equatorial orientation. Furthermore, the similar coupling constants of H2ax to H3 and H1 support a 1,3-*cis* configuration.

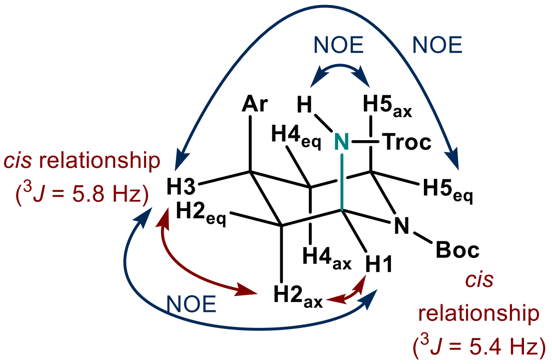

tert-butyl-2-(((2,2,2-trichloroethoxy)carbonyl)amino)-5-(3,4,5-trimethoxybenzamido)piperidine-1-carboxylate (7x)

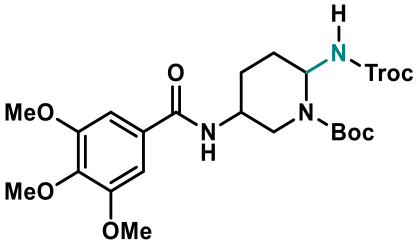

Following the general procedure, the reaction was performed with *N*-Boc-troxipide (78.9 mg, 0.200 mmol), $TrocN_3$ (131 mg, 0.600 mmol, 3.00 equiv.), NaOEt (13.6 mg, 0.200 mmol, 1.00 equiv.), and **1** (9.0 mg, 0.020 mmol, 10 mol%) in MeCN (0.20 mL, 1.0 M) for 24 h under 456 nm LEDs irradiation. The diastereomeric ratio was not assigned due to the complexity of ^{1}H NMR spectrum arising from the rotameric nature of each diastereomer of the product. The crude mixture was purified by flash column chromatography (EtOAc/pentane = 2:8 to 4:6) to afford the mixture of two diastereomers (4:1) in 35% combined yield (40.6 mg, 0.0694 mmol) as a white solid.

HRMS (ESI positive): calc'd for $C_{23}H_{32}N_3O_8Cl_3Na_1$ $[M+Na]^+$: 606.1147, found: 606.11522.

NOTE: At room temperature, the signals appeared slightly broadened. Therefore, the sample was characterized at 323 K, where the signals appeared significantly sharper.

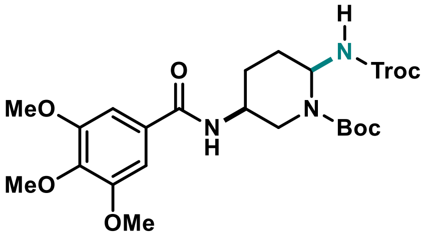

Major diastereomer

^{1}H NMR (600 MHz, $CDCl_3$, 323 K) δ 6.99 (s, 2H), 5.98 (d, *J* = 7.6 Hz, 1H), 5.95 (dt, *J* = 7.0, 4.4 Hz, 1H), 5.50 (d, *J* = 7.0 Hz, 1H), 4.74 (s, 2H), 4.30 (ddd, *J* = 13.0, 5.0, 1.8 Hz, 1H), 4.04 (tdt, *J* = 11.2, 7.6, 5.0 Hz, 1H), 3.90 (s, 6H), 3.88 (s, 3H), 2.80 (dd, *J* = 13.0, 11.0 Hz, 1H), 2.11 (dp, *J* = 13.0, 4.1 Hz, 1H), 2.05 (dq, *J* = 14.2, 3.6 Hz, 1H), 1.93 (tt, *J* = 13.7, 4.1 Hz, 1H), 1.65 (tdd, *J* = 13.2, 11.4, 4.1 Hz, 1H), 1.49 (s, 9H).

^{13}C NMR (151 MHz, $CDCl_3$, 323 K) δ 166.9, 154.2, 153.58, 153.41, 141.92, 129.89, 105.17, 95.69, 81.4, 74.89, 61.04, 59.0, 56.73, 46.5, 43.8, 28.7, 28.47, 25.9.

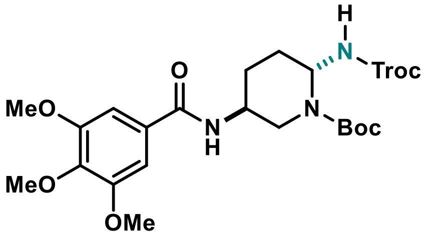

Minor diastereomer

^{1}H NMR (600 MHz, CDCl$_3$, 323 K) δ 6.99 (s, 2H), 6.49 (d, J = 6.5 Hz, 1H), 5.95 (dt, J = 7.0, 4.4 Hz, 1H), 5.50 (d, J = 7.0 Hz, 1H), 4.75 (s, 2H), 4.25 (qt, J = 6.5, 4.1 Hz, 1H), 4.12 (dt, J = 14.6, 2.3 Hz, 1H), 3.90 (s, 6H), 3.88 (s, 3H), 3.26 (dd, J = 14.6, 2.7 Hz, 1H), 2.11 (dp, J = 13.0, 4.1 Hz, 1H), 2.05 (dq, J = 14.2, 3.6 Hz, 1H), 1.93 (tt, J = 13.7, 4.1 Hz, 1H), 1.79 (dq, J = 13.8, 3.6 Hz, 1H), 1.48 (s, 9H). **^{13}C NMR (151 MHz, CDCl$_3$, 323 K) δ** 167.0, 155.6, 153.58, 153.42, 141.92, 129.87, 105.18, 95.58, 81.6, 75.0, 61.05, 60.2, 56.69, 45.2, 43.1, 28.47, 25.2, 23.5.

NH moiety is in the axial position in both diastereomers as a cross peak to H3ax and H5ax is observed in both cases. In the major diastereomer H5ax has a large coupling ($^{3}J_{HH}$ = 11 Hz) to H4 indicating that H4 is also in axial position. This is further supported by a NOESY cross peak of H4 to H2ax. In the minor diastereomer, H4 is in the equatorial position as the $^{3}J_{HH}$ coupling of H5ax is small (2.7 Hz)

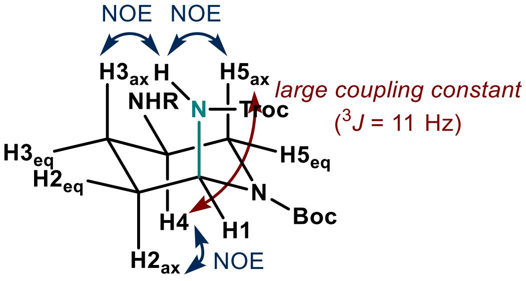

major diastereomer

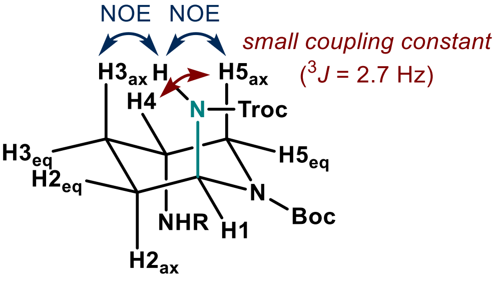

minor diastereomer

2,2,2-trichloroethyl (*S*)-(1-methyl-5-(pyridin-3-yl)pyrrolidin-2-ylidene)carbamate (7y)

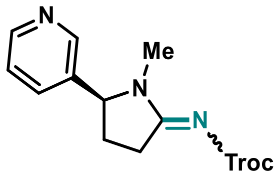

Following the general procedure, the reaction was performed with (–)-nicotine (32.5 mg, 0.200 mmol), $TrocN_3$ (131 mg, 0.600 mmol, 3.00 equiv.) and **1** (9.0 mg, 0.020 mmol, 10 mol%) in MeCN (0.20 mL, 1.0 M) for 24 h under 456 nm LEDs irradiation. The crude mixture was purified by flash column chromatography (EtOAc/cyclohexane = 3:7 to 7:3, with 1% of Et_3N) to afford the product in 53% yield (37 mg, 0.11 mmol) as a pink oil.

R_f = 0.25 (EtOAc)

^{1}H NMR (600 MHz, $CDCl_3$, 298 K) δ 8.63 (dd, *J* = 4.9, 1.6 Hz, 1H), 8.52 (d, *J* = 2.3 Hz, 1H), 7.51 (dddd, *J* = 7.9, 2.3, 1.6, 0.4 Hz, 1H), 7.36 (ddd, *J* = 7.9, 4.8, 0.9 Hz, 1H), 4.83 (s, 2H), 4.68 (dd, *J* = 8.5, 6.0 Hz, 1H), 3.34–3.25 (m, 1H), 3.19 (dddd, *J* = 18.4, 9.7, 6.8, 1.0 Hz, 1H), 2.89 (s, 3H), 2.61 (dddd, *J* = 13.3, 9.7, 8.4, 5.8 Hz, 1H), 1.98 (dddd, *J* = 13.4, 9.6, 6.8, 6.0 Hz, 1H).

^{13}C NMR (151 MHz, $CDCl_3$, 298 K) δ 175.1, 161.6, 150.4, 148.8, 135.5, 133.9, 124.3, 96.1, 75.5, 64.8, 31.2, 30.7, 29.9.

HRMS (EI): calc'd for $C_{13}H_{14}N_3O_2Cl_3$ $[M]^+$: 349.014610, found: 349.014730.

The spectral data matched with those reported in the literature.[29]

(2S,5S)-1-(*tert*-butoxycarbonyl)-5-(((2,2,2-trichloroethoxy)carbonyl)amino)pyrrolidine-2-carboxylic acid (7z)

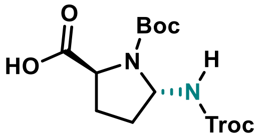

Following the general procedure, the reaction was performed with (*L*)-*N*-Boc-proline (43.0 mg, 0.200 mmol), $TrocN_3$ (131 mg, 0.600 mmol, 3.00 equiv.) and **1** (9.0 mg, 0.020 mmol, 10 mol%) in MeCN (0.50 mL, 0.40 M) for 72 h under 456 nm LEDs irradiation. The diastereomeric ratio was determined to be a >5:1 ratio by ^{1}H NMR spectroscopy of the crude reaction mixture. The crude mixture was purified by flash column chromatography (Et_2O/DCM = 0:10 to 1:9, with 1% of AcOH) to afford the product in 23% yield (19 mg, 0.046 mmol) as a colorless oil.

R_f = 0.25 (Et_2O/DCM = 2:8)

NOTE: At room temperature, the signals appeared slightly broadened. Therefore, the sample was characterized at 383 K.

^{1}H NMR (600 MHz, DMSO-d_6, 383 K) δ 11.93 (s, 1H), 7.74 (brs, 1H), 5.49 (td, *J* = 7.7, 0.9 Hz, 1H), 4.77 (qff, *J* = 12.3 Hz, 2H), 4.17 (dd, *J* = 9.3, 1.5 Hz, 1H), 2.45 (tdd, *J* = 13.2, 9.3, 6.8 Hz, 1H), 2.12 (tt, *J* = 13.2, 7.2 Hz, 1H), 1.91–1.83 (m, 1H), 1.74 (ddt, *J* = 12.8, 6.8, 0.9 Hz, 1H), 1.39 (s, 9H).

^{13}C NMR (151 MHz, DMSO-d_6, 383 K) δ 172.6, 152.23, 152.15, 95.8, 78.9, 73.3, 65.4, 58.4, 31.1, 27.5, 26.0

HRMS (ESI negative): calc'd for $C_{13}H_{18}N_2O_6Cl_3$ $[M-H]^-$: 403.023596, found: 403.023840.

The relative stereochemistry was determined based on an NOE.

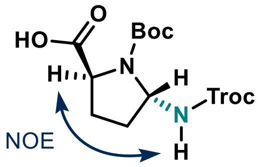

6.3. Characterization of intermolecular C–H azidation products 9

2,2,2-trichloroethyl1-2-azidopyrrolidine-1-carboxylate (9a)

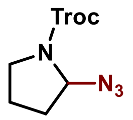

Following the general procedure, the reaction was performed with pyrrolidine (14.2 mg, 0.200 mmol), $TrocN_3$ (131 mg, 0.600 mmol, 3.00 equiv.) and **1** (9.0 mg, 0.020 mmol, 10 mol%) in MeCN (1.0 mL, 0.20 M) for 24 h under 456 nm LEDs irradiation. The crude mixture was purified by flash column chromatography (Et_2O/DCM = 1:99 to 3:97) to afford the 2 rotamers (ratio ~ 1.1:1) in 37% yield (21 mg, 0.074 mmol) as a pale-yellow viscous oil.

R_f = 0.7 (Et_2O/DCM = 5:95, stained with Ninhydrin)

HRMS (ESI positive): calc'd for $C_7H_9N_4O_2Cl_3Na_1$ $[M+Na]^+$: 308.9683, found: 308.96829.

Major rotamer

^{1}H NMR (600 MHz, $CDCl_3$, 298 K) δ 5.62–5.58 (m, 1H), 4.79 (AB, *J* = 1.9 Hz, 2H), 3.69 (ddd, *J* = 10.4, 8.4, 2.0 Hz, 1H), 3.49–3.39 (m, 1H), 2.10–1.88 (m, 4H).

^{13}C NMR (151 MHz, $CDCl_3$, 298 K) δ 152.6, 95.5, 75.22, 75.1, 46.4, 32.5, 23.1.

Minor rotamer

^{1}H NMR (600 MHz, $CDCl_3$, 298 K) δ 5.57 (d, *J* = 4.7 Hz, 1H), 4.99 (d, *J* = 11.9 Hz, 1H), 4.65 (d, *J* = 11.9 Hz, 1H), 3.64–3.60 (m, 1H), 3.49–3.39 (m, 1H), 2.10–1.88 (m, 4H).

^{13}C NMR (151 MHz, $CDCl_3$, 298 K) δ 153.9, 95.3, 75.24, 74.5, 46.8, 33.6, 22.2.

tert-butyl 2-azidopyrrolidine-1-carboxylate (9b)

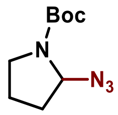

Following the general procedure, the reaction was performed with pyrrolidine (14.2 mg, 0.200 mmol), $BocN_3$ (85.9 mg, 0.600 mmol, 3.00 equiv.) and **1** (9.0 mg, 0.020 mmol, 10 mol%) in MeCN (1.0 mL, 0.20 M) for 24 h under 456 nm LEDs irradiation. The reaction mixture was diluted with $CDCl_3$, and the yield was determined by ^{1}H NMR spectroscopy (78% yield) using trichloroethylene as an internal standard. The crude mixture was purified by flash column chromatography (EtOAc/pentane = 3:97) to afford the 2 rotamers (ratio ~ 1.8:1) in 51% yield (21.5 mg, 0.101 mmol) as a colorless oil. The discrepancy between the yield determined by ^{1}H NMR spectroscopy and isolated yield was a result of the volatility and instability of the compound during flash column chromatography.

R_f = 0.6 (EtOAc/pentane = 1:9, stained with Ninhydrin)

HRMS (ESI positive): calc'd for $C_9H_{16}N_4O_2Na_1$ $[M+Na]^+$: 235.11655, found: 235.11663.

Major rotamer

^{1}H NMR (600 MHz, $CDCl_3$, 298 K) δ 5.45 (d, *J* = 4.6 Hz, 1H), 3.55–3.45 (m, 1H), 3.38–3.21 (m, 1H), 2.06–1.81 (m, 4H), 1.51 (s, 9H).

^{13}C NMR (151 MHz, $CDCl_3$, 298 K) δ 154.8, 81.3, 74.63, 46.4, 33.5, 28.4, 22.2.

Minor rotamer

^{1}H NMR (600 MHz, $CDCl_3$, 298 K) δ 5.53 (d, *J* = 4.1 Hz, 1 H), 3.55–3.45 (m, 1H), 3.38–3.21 (m, 1H), 2.06–1.81 (m, 4H), 1.48 (s, 9H).

^{13}C NMR (151 MHz, $CDCl_3$, 298 K) δ 153.8, 80.6, 74.62, 46.0, 32.6, 28.4, 23.2.

The spectral data matched with those reported in the literature.[30]

benzyl 2-azidopyrrolidine-1-carboxylate (9c)

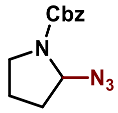

Following the general procedure, the reaction was performed with pyrrolidine (14.2 mg, 0.200 mmol), $CbzN_3$ (106 mg, 0.600 mmol, 3.00 equiv.) and **1** (9.0 mg, 0.020 mmol, 10 mol%) in MeCN (1.0 mL, 0.20 M) for 24 h under 456 nm LEDs irradiation. The crude mixture was purified by flash column chromatography (EtOAc/pentane = 2:98) to afford the 2 rotamers (ratio ~ 1.1:1) in 66% yield (32.6 mg, 0.132 mmol) as a colorless oil.

R_f = 0.3 (EtOAc/pentane = 1:9, stained with Ninhydrin)

HRMS (ESI positive): calc'd for $C_{12}H_{14}N_4O_2Na_1$ $[M+Na]^+$: 269.10090, found: 269.10066.

NOTE: The rotamer exchange was almost completely frozen out at 253 K. Therefore, the sample was characterized at 253 K. The two rotamers were assigned by 2D NMR and 1D selective TOCSY spectroscopy.

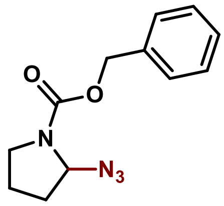

Major rotamer

^{1}H NMR (600 MHz, $CDCl_3$, 253 K) δ 7.44–7.31 (m, 5H), 5.52–5.48 (m, 1H), 5.23 (d, J = 12.0 Hz, 1H), 5.12 (d, J = 12.0 Hz, 1H), 3.64–3.54 (m, 1H), 3.42–3.28 (m, 1H), 2.08–1.84 (m, 4H).

^{13}C NMR (151 MHz, $CDCl_3$, 253 K) δ 154.4, 135.8, 128.8, 128.62, 128.5, 74.2, 67.8, 46.4, 33.3, 22.1.

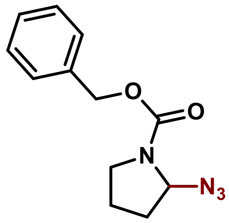

Minor rotamer

^{1}H NMR (600 MHz, $CDCl_3$, 253 K) δ 7.44–7.31 (m, 5H), 5.62–5.58 (m, 1H), 5.20 (d, J = 12.3 Hz, 1H), 5.15 (d, J = 12.3 Hz, 1H), 3.64–3.54 (m, 1H), 3.42–3.28 (m, 1H), 2.08–1.84 (m, 4H).

^{13}C NMR (151 MHz, $CDCl_3$, 253 K) δ 155.5, 136.1, 128.63, 128.3, 128.2, 75.0, 67.4, 46.2, 32.4, 23.1.

The spectral data matched with those reported in the literature.[31]

Large scale synthesis

Following the procedure mentioned above, the reaction was conducted in 2.0 mmol scale to afford the product in 43% yield (209 mg, 0.850 mmol) as a colorless oil.

NOTE: $CbzN_3$ should be added slowly to the reaction mixture.

benzyl 2-azido-5-methylpyrrolidine-1-carboxylate (9d)

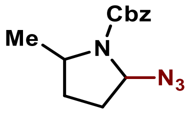

Following the general procedure, the reaction was performed with 2-methyl-pyrrolidine (17.0 mg, 0.200 mmol), $CbzN_3$ (106 mg, 0.600 mmol, 3.00 equiv..) and **1** (9.0 mg, 0.020 mmol, 10 mol%) in MeCN (1.0 mL, 0.20 M) for 24 h under 456 nm LEDs irradiation. The diastereomeric ratio was determined to be a 1.3:1 ratio by ^{1}H NMR spectroscopy of the crude reaction mixture. The crude mixture was purified by flash column chromatography (EtOAc/pentane = 2:98) to afford the two diastereomers were isolated separately in 33% combined yield.

NOTE: The rotamer exchange was almost completely frozen out at 253 K. Therefore, the sample was characterized at 253 K. The two rotamers were assigned by 2D NMR and 1D selective TOCSY spectroscopy.

***Trans diastereomer** (two rotamers ~ 1:0.95)*

18% yield (9.5 mg, 0.037 mmol), a colorless oil.

R_f = 0.5 (EtOAc/pentane = 1:9, stained with Ninhydrin)

HRMS (ESI positive): calc'd for $C_{13}H_{16}N_4O_2Na_1$ $[M+Na]^+$: 283.11655, found: 283.11640.

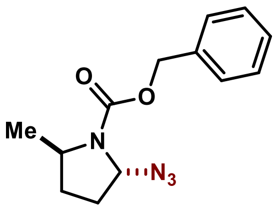

Major rotamer

^{1}H NMR (600 MHz, CDCl$_3$, 253 K) δ 7.43–7.40 (m, 2H), 7.39–7.32 (m, 3H), 5.42 (d, J = 5.1 Hz, 1H), 5.23 (d, J = 12.0 Hz, 1H), 5.09 (d, J = 12.0 Hz, 1H), 4.09 (dq, J = 7.8, 6.4 Hz, 1H), 2.22–2.04 (m, 2H), 1.89–1.80 (m, 1H), 1.57 (dd, J = 11.4, 5.3, 1H), 1.18 (d, J = 6.4 Hz, 3H).

^{13}C NMR (151 MHz, CDCl$_3$, 253 K) δ 153.9, 135.8, 128.8, 128.6, 128.4, 74.8, 67.5, 53.8, 31.2, 29.2, 19.4.

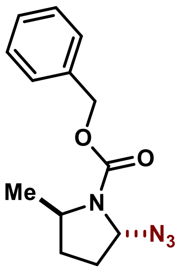

Minor rotamer

^{1}H NMR (600 MHz, CDCl$_3$, 253 K) δ 7.39–7.32 (m, 5H), 5.53 (d, J = 5.6 Hz, 1H), 5.24 (d, J = 12.3 Hz, 1H), 5.14 (d, J = 12.3 Hz, 1H), 4.05 (dq, J = 7.9, 6.4 Hz, 1H), 2.17 (dddd, J = 13.9, 12.1, 6.1, 7.8 Hz, 1H), 2.09 (dddd, J = 13.9, 12.9, 6.2, 5.6 Hz, 1H), 1.85 (dd, J = 12.9, 6.1 Hz, 1H), 1.59 (dd, J = 12.1, 6.2 Hz, 1H), 1.12 (d, J = 6.4 Hz, 3H).

^{13}C NMR (151 MHz, CDCl$_3$, 253 K) δ 155.4, 136.1, 128.7, 128.3, 128.1, 75.6, 67.4, 53.3, 30.2, 30.1, 20.4.

The ^{3}J coupling between H2'' and H3'' is rather large (~13.9 Hz), which is most consistent with a *trans* relationship. H1 shows an intense NOE to H2'', whereas H4 shows an intense NOE to H3''. Additionally, Me group exhibits strong NOEs to H2'' and H3, and even a weak NOE to H1. These observations support the trans configuration shown here.

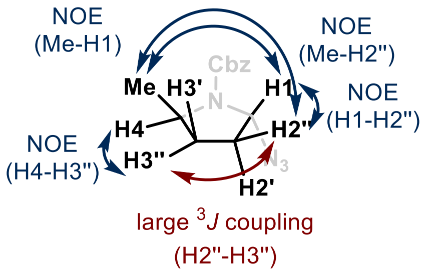

Cis diastereomer *(two rotamers ~ 1:0.75)*

15% yield (7.8 mg, 0.030 mmol), a colorless oil.

R_f = 0.4 (EtOAc/pentane = 1:9, stained with Ninhydrin)

HRMS (ESI positive): calc'd for $C_{13}H_{16}N_4O_2Na_1$ $[M+Na]^+$: 283.11655, found: 283.11632.

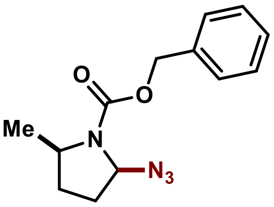

Major rotamer

^{1}H NMR (600 MHz, CDCl$_3$, 253 K) δ 7.43–7.32 (m, 5H), 5.55 (dd, *J* = 5.9, 1.4 Hz, 1H), 5.20 (d, *J* = 12.0 Hz, 1H), 5.10 (d, *J* = 12.0 Hz, 1H), 3.90 (tq, *J* = 8.7, 7.1 Hz, 1H), 2.12 (dtd, *J* = 12.8, 7.2, 2.3 Hz, 1H), 1.87 (dddd, *J* = 13.2, 12.0, 7.3, 5.9 Hz, 1H), 1.79 (ddt, *J* = 13.2, 6.9, 2.3 Hz, 1H), 1.69 (tdd, *J* = 12.8, 8.7, 6.8 Hz, 1H), 1.38 (d, *J* = 6.1 Hz, 3H).

^{13}C NMR (151 MHz, CDCl$_3$, 253 K) δ 154.3, 135.8, 128.6, 128.4, 128.3, 75.1, 67.6, 54.8, 32.0, 31.0, 21.1.

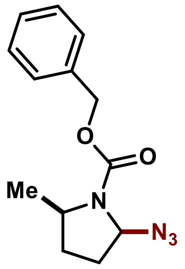

Minor rotamer

^{1}H NMR (600 MHz, CDCl$_3$, 253 K) δ 7.43–7.32 (m, 5H), 5.64 (dd, *J* = 6.1, 1.9 Hz, 1H), 5.18 (s, 2H), 3.94–3.83 (m, 1H), 2.11 (dtd, *J* = 12.7, 7.5, 3.2 Hz, 1H), 1.92 (dddd, *J* = 13.2, 10.9, 7.5, 6.1 Hz, 1H), 1.81 (dddd, *J* = 13.2, 7.0, 3.2, 1.9 Hz, 1H), 1.72 (dddd, *J* = 12.7, 10.9, 7.4, 7.0 Hz, 1H).1.32 (d, *J* = 6.1 Hz, 3H).

^{13}C NMR (151 MHz, CDCl$_3$, 253 K) δ 155.8, 136.0, 128.7, 128.6, 128.2, 76.1, 67.6, 54.2, 31.8, 31.3, 22.0.

The ^{3}J coupling between H2'' and H3'' is rather large (~10.9/12.0 Hz), which is most consistent with a trans relationship. H1 shows an intense NOE to H2'' and slightly weaker to H2'. H4 shows an intense NOE to H3' and very weak NOE to H1. Additionally, Me group shows a strong NOE to H3". These observations support the cis configuration shown here.

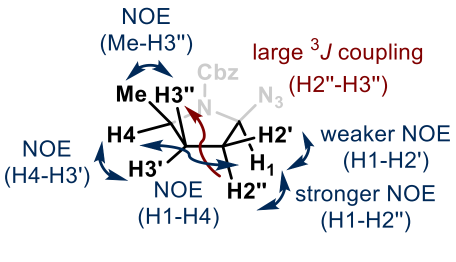

benzyl 2-azido-5-phenylpyrrolidine-1-carboxylate (9e)

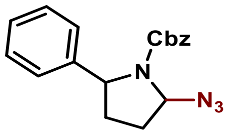

Following the general procedure, the reaction was performed with 2-phenyl-pyrrolidine (29.4 mg, 0.200 mmol), $CbzN_3$ (106 mg, 0.600 mmol, 3.00 equiv.) and **1** (9.0 mg, 0.020 mmol, 10 mol%) in MeCN (1.0 mL, 0.20 M) for 24 h under 456 nm LEDs irradiation. The diastereomeric ratio was determined to be a 1.1:1 ratio by ^{1}H NMR spectroscopy of the crude reaction mixture. The crude mixture was purified by flash column chromatography (EtOAc/pentane = 3:97 to 5:95) to afford the two diastereomers were isolated separately in 32% combined yield.

*NOTE: The rotamer exchange was almost completely frozen out at 233 K. Therefore, the sample was characterized at 233 K. The relative stereochemistry was established by comparison with the product **9d**. The two rotamers were assigned by 2D NMR and 1D selective TOCSY spectroscopy.*

Trans diastereomer *(two rotamers ~ 1.7:1)*

24% yield (15 mg, 0.047 mmol), a colorless oil.

R_f = 0.6 (EtOAc/pentane = 1:9, stained with Ninhydrin)

HRMS (ESI positive): calc'd for $C_{18}H_{18}N_4O_2Na_1$ $[M+Na]^+$: 345.13220, found: 345.13217.

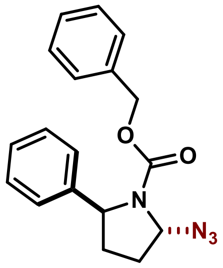

Major rotamer

^{1}H NMR (600 MHz, $CDCl_3$, 233 K) δ 7.35–7.28 (m, 3H), 7.22–7.18 (m, 1H), 7.17–7.13 (m, 2H), 7.10 – 7.05 (m, 2H), 6.71 (d, *J* = 7.2 Hz, 2H), 5.87 (d, *J* = 5.9 Hz, 1H), 5.14 (d, *J* = 12.7 Hz, 1H), 5.07 (d, *J* = 8.6 Hz, 1H), 4.91 (d, *J* = 12.7 Hz, 1H), 2.56–2.40 (m, 1H), 2.19–2.08 (m, 1H), 1.89–1.75 (m, 2H).

^{13}C NMR (151 MHz, $CDCl_3$, 233 K) δ 155.3, 142.7, 135.8, 128.63, 128.3, 127.7, 127.11, 127.08, 125.1, 75.8, 67.0, 61.0, 32.4, 29.5.

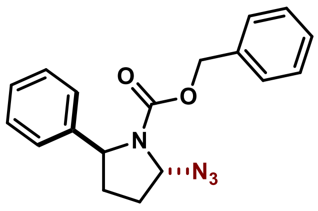

Minor rotamer

^{1}H NMR (600 MHz, $CDCl_3$, 233 K) δ 7.45–7.41 (m, 2H), 7.41–7.35 (m, 3H), 7.35–7.28 (m, 2H), 7.28–7.22 (m, 1H), 7.12–7.10 (m, 2H), 5.73 (d, *J* = 5.7 Hz, 1H), 5.20 (d, *J* = 11.9 Hz, 1H), 5.13 (d, *J* = 11.9 Hz, 1H), 5.09 (d, *J* = 8.7 Hz, 1H), 2.56–2.40 (m, 1H), 2.19–2.08 (m, 1H), 1.89–1.75 (m, 2H).

^{13}C NMR (151 MHz, $CDCl_3$, 233 K) δ 153.9, 141.8, 135.5, 129.1, 128.7, 128.59, 128.5, 127.2, 125.0, 75.2, 67.9, 61.1, 31.6, 30.7.

Cis diastereomer *(two rotamers ~ 1.2:1)*

8% yield (5.1 mg, 0.016 mmol), a colorless oil.

R_f = 0.4 (EtOAc/pentane = 1:9, stained with Ninhydrin)

HRMS (ESI positive): calc'd for $C_{18}H_{18}N_4O_2Na_1$ $[M+Na]^+$: 345.13220, found: 345.13195.

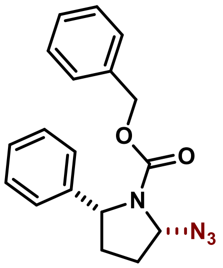

Major rotamer

^{1}H NMR (600 MHz, CDCl$_3$, 233 K) δ 7.41–7.28 (m, 5H), 7.26–7.20 (m, 1H), 7.21–7.14 (m, 2H), 6.75–6.69 (m, 2H), 5.91–5.87 (m, 1H), 5.03 (d, *J* = 12.6 Hz, 1H), 4.90 (d, *J* = 12.6 Hz, 1H), 4.79–4.71 (m, 1H), 2.42–2.32 (m, 1H), 2.08–1.84 (m, 3H).

^{13}C NMR (151 MHz, CDCl$_3$, 233 K) δ 155.7, 143.0, 135.48, 128.7, 128.3, 127.8, 127.27, 127.26, 126.2, 76.5, 67.3, 62.8, 34.7, 31.7.

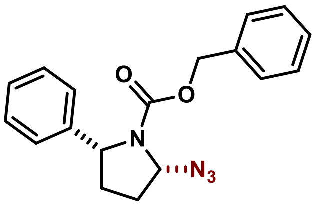

Minor rotamer

^{1}H NMR (600 MHz, CDCl$_3$, 233 K) δ 7.41–7.28 (m, 10H), 5.80–5.76 (m, 1H), 5.18 (d, *J* = 12.0 Hz, 1H), 4.97 (d, *J* = 12.0 Hz, 1H), 4.79–4.71 (m, 1H), 2.42–2.32 (m, 1H), 2.08–1.84 (m, 3H).

^{13}C NMR (151 MHz, CDCl$_3$, 233 K) δ 154.1, 142.2, 135.51, 129.0, 128.8, 128.6, 128.5, 127.6, 126.3, 75.4, 67.8, 63.2, 33.7, 32.3.

1-benzyl 2-methyl (2S,5R)-5-azidopyrrolidine-1,2-dicarboxylate (9f)

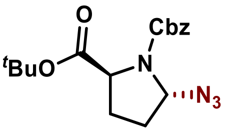

Following the general procedure, the reaction was performed with (*L*)-proline *tert*-butyl ester (34.2 mg, 0.200 mmol), $CbzN_3$ (106 mg, 0.600 mmol, 3.00 equiv.) and **1** (9.0 mg, 0.020 mmol, 10 mol%) in MeCN (1.0 mL, 0.20 M) for 24 h under 456 nm LEDs irradiation. The diastereomeric ratio was determined to be a >5:1 ratio by ^{1}H NMR spectroscopy of the crude reaction mixture The crude mixture was purified by flash column chromatography (EtOAc/pentane = 2:98) to afford the major diastereomer as 2 rotamers (ratio ~ 1.25:1) in 31% yield (21.5 mg, 0.0621 mmol) as a colorless oil.

R_f = 0.6 (EtOAc/pentane = 1:9, visualized under UV light)

HRMS (ESI positive): calc'd for $C_{17}H_{22}N_4O_4Na_1$ $[M+Na]^+$: 369.153324, found: 369.153290.

NOTE: The relative stereochemistry was assigned based on the comparison to ***9u****.*

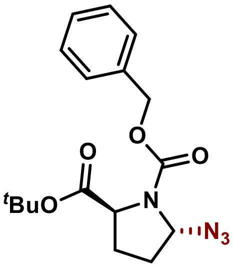

Major rotamer

^{1}H NMR (600 MHz, $CDCl_3$, 298 K) δ 7.38–7.28 (m, 5H), 5.72 (d, *J* = 6.2 Hz, 1H), 5.21 (d, *J* = 12.4 Hz, 1H), 5.12 (d, *J* = 12.4 Hz, 1H), 4.30 (d, *J* = 9.1 Hz, 1H), 2.41–2.26 (m, 1H), 2.21–2.08 (m, 1H), 2.00–1.92 (m, 1H), 1.89–1.80 (m, 1H), 1.32 (s, 9H).

^{13}C NMR (151 MHz, $CDCl_3$, 298 K) δ 171.02, 154.9, 135.9, 128.6, 128.3, 128.2, 81.89, 75.7, 67.8, 59.9, 30.8, 28.5, 27.9.

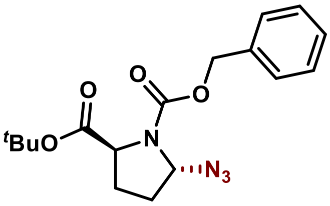

Minor rotamer

^{1}H NMR (600 MHz, $CDCl_3$, 298 K) δ 7.43–7.38 (m, 2H), 7.38–7.28 (m, 3H), 5.59 (d, *J* = 6.0 Hz, 1H), 5.22 (d, *J* = 12.1 Hz, 1H), 5.18 (d, *J* = 12.1 Hz, 1H), 4.33 (d, *J* = 9.1 Hz, 1H), 2.41–2.26 (m, 1H), 2.21–2.08 (m, 1H), 2.00–1.92 (m, 1H), 1.89–1.80 (m, 1H), 1.44 (s, 9H),

^{13}C NMR (151 MHz, $CDCl_3$, 298 K) δ 170.96, 154.1, 135.8, 128.67, 128.64, 128.5, 81.94, 74.9, 68.1, 60.2, 31.8, 28.1, 27.3.

tert-butyl 2-azidopiperidine-1-carboxylate (9g)

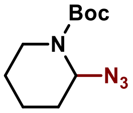

Following the general procedure, the reaction was performed with piperidine (17.0 mg, 0.200 mmol), $BocN_3$ (85.9 mg, 0.600 mmol, 3.00 equiv.) and **1** (9.0 mg, 0.020 mmol, 10 mol%) in MeCN (1.0 mL, 0.20 M) for 24 h under 456 nm LEDs irradiation. The reaction mixture was diluted with $CDCl_3$, and the yield was determined by ^1H NMR spectroscopy (37% yield) using trichloroethylene as an internal standard. The discrepancy between the yield between determined by ^1H NMR spectroscopy and isolated yield was a result of the volatility and instability of the compound during flash column chromatography.

NOTE: Isolation of the azidation product was not possible as it decomposed to the corresponding alkene via elimination during a purification on silica gel. Thus, the yield was calculated using NMR spectroscopy.

HRMS (ESI positive): calc'd for $C_{10}H_{18}N_4O_2Na_1$ $[M+Na]^+$: 249.13220, found: 249.13218.

The spectral data matched with those reported in the literature.[31]

benzyl 2-azidopiperidine-1-carboxylate (9h)

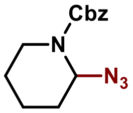

Following the general procedure, the reaction was performed with piperidine (17.0 mg, 0.200 mmol), $CbzN_3$ (106 mg, 0.600 mmol, 3.00 equiv.) and **1** (9.0 mg, 0.020 mmol, 10 mol%) in MeCN (1.0 mL, 0.20 M) for 24 h under 456 nm LEDs irradiation. The crude mixture was purified by flash column chromatography (EtOAc/pentane = 5:95) to afford the 2 rotamers (ratio ~ 1.1:1) in 41% yield (21.1 mg, 0.0811 mmol) as a colorless oil.

R_f = 0.5 (EtOAc/pentane = 1:9, visualized under UV)

NOTE: At room temperature, the ^1H NMR signals appeared slightly broadened. At lower temperatures, two sets of rotamers were observed, while most signals sharpened at 333 K. Therefore, the sample was fully characterized at 333K.

^{1}H NMR (600 MHz, $CDCl_3$, 333 K) δ 7.37 (d, *J* = 4.5 Hz, 4H), 7.35–7.30 (m, 1H), 5.97 (brs, 1H), 5.18 (AB system, *J* = 12.3 Hz, 2H), 4.04 (d, *J* = 13.0 Hz, 1H), 3.07 (td, *J* = 13.0, 3.0 Hz, 1H), 1.81 (m, *J* = 12.3 Hz, 1H), 1.74–1.57 (m, 4H), 1.52–1.41 (m, 1H).

^{13}C NMR (151 MHz, $CDCl_3$, 333 K) δ 155.7, 136.5, 128.7, 128.4, 128.3, 69.4, 68.0, 40.3, 29.8, 24.7, 18.5.

HRMS (ESI positive): calc'd for $C_{13}H_{16}N_4O_2Na_1$ $[M+Na]^+$: 283.11655, found: 283.11645.

The spectral data matched with those reported in the literature.[31]

benzyl-2-azido-4-phenylpiperidine-1-carboxylate (9i)

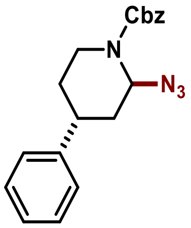

Following the general procedure, the reaction was performed with morpholine (17.4 mg, 0.200 mmol), $CbzN_3$ (106 mg, 0.600 mmol, 3.00 equiv.) and **1** (9.0 mg, 0.020 mmol, 10 mol%) in MeCN (1.0 mL, 0.20 M) for 24 h under 456 nm LEDs irradiation. The diastereomeric ratio was determined to be a >5:1 ratio by ^{1}H NMR spectroscopy of the crude reaction mixture. The crude mixture was purified by flash column chromatography (EtOAc/pentane = 3:97) to afford the 2 rotamers (ratio ~ 1:0.9) in 25% yield (17 mg, 0.049 mmol) as a colorless oil.

R_f = 0.70 (EtOAc/pentane = 1:9, stained with Ninhydrin, visualized under UV light)

HRMS (ESI positive): calc'd for $C_{19}H_{20}N_4O_2Na_1$ $[M+Na]^+$: 359.14785, found: 359.14778.

NOTE: At room temperature, the signals appeared slightly broadened. Therefore, the sample was characterized at 253 K, where the signals appeared significantly sharper. Due to overlaps of several signals, the individual rotamers are not assigned.

^{1}H NMR (600 MHz, $CDCl_3$, 253 K) δ 7.43–7.39 (m, 4H), 7.40–7.36 (m, 1H), 7.36–7.30 (m, 2H), 7.27–7.21 (m, 1H), 7.22–7.17 (m, 2H), 6.24–6.19 (m, 0.47H), 6.09 (s, 0.52H), 5.23–5.15 (m, 2H), 4.25 (ddd, J = 13.2, 4.6, 2.2 Hz, 0.52H), 4.15 (ddd, J = 13.3, 4.6, 2.2 Hz, 0.47H), 3.28–3.17 (m, 1H), 3.03–2.92 (m, 1H), 2.01 (dq, J = 13.7, 2.2 Hz, 0.47H), 1.96–1.90 (m, 1.05H), 1.86 (dp, J = 13.2, 2.6 Hz, 0.47H), 1.83 – 1.61 (m, 2H).

^{13}C NMR (151 MHz, $CDCl_3$, 253 K) δ 156.0, 155.0, 144.4, 128.74, 128.73, 128.70, 128.69, 128.6, 128.5, 128.3, 126.88, 126.87, 126.8, 69.2, 69.0, 68.2, 68.0, 40.2, 40.0, 37.1, 36.8, 36.0, 35.9, 31.9, 31.8.

The 1,3-*trans* configuration is supported by the *J*-values of H2ax to H1 (3.9 Hz, *cis*) and to H3 (12.7 Hz, *trans*). Based on the magnitude of the observed coupling constants, H3 adopts an axial orientation within the ring, whereas H1 occupies an equatorial position.

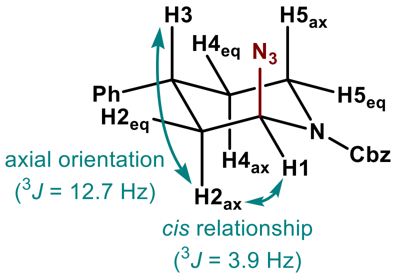

benzyl 3-azidomorpholine-4-carboxylate (9j)

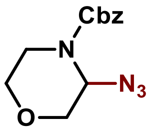

Following the general procedure, the reaction was performed with morpholine (17.4 mg, 0.200 mmol), $CbzN_3$ (106 mg, 0.600 mmol, 3.00 equiv.) and **1** (9.0 mg, 0.020 mmol, 10 mol%) in MeCN (1.0 mL, 0.20 M) for 24 h under 456 nm LEDs irradiation. The crude mixture was purified by flash column chromatography (EtOAc/pentane = 3:97) to afford the 2 rotamers (ratio ~ 1:0.9) in 52% yield (27.3 mg, 0.104 mmol) as a colorless oil.

R_f = 0.5 (EtOAc/pentane = 4:6, stained with Ninhydrin)

HRMS (ESI positive): calc'd for $C_{12}H_{14}N_4O_3Na_1$ $[M+Na]^+$: 285.095809, found: 285.095850.

NOTE: The rotamer exchange was almost completely frozen out at 233 K. Therefore, the sample was characterized at 233 K. The two rotamers were assigned by 2D NMR spectroscopy.

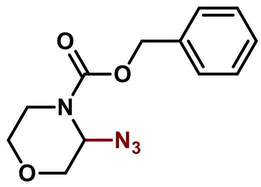

Major Rotamer

^{1}H NMR (600 MHz, CDCl$_3$, 233 K) δ 7.43–7.34 (m, 5H), 5.62 (s, 1H), 5.19 (d, *J* = 11.8 Hz, 1H), 5.15 (d, *J* = 11.8 Hz, 1H), 4.00 (dd, *J* = 11.7, 3.6 Hz, 1H), 3.88 (dd, *J* = 13.4, 2.8 Hz, 1H), 3.84 (d, *J* = 12.2 Hz, 1H), 3.55 (dd, *J* = 12.2, 2.4 Hz, 1H), 3.54 (td, *J* = 12.3, 2.8 Hz, 1H), 3.37 (td, *J* = 13.6, 3.7 Hz, 1H).

^{13}C NMR (151 MHz, CDCl$_3$, 233 K) δ 154.7, 135.0, 128.9, 128.8, 128.7, 68.6, 68.5, 67.1, 66.4, 39.2.

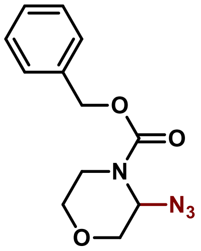

Minor Rotamer

^{1}H NMR (600 MHz, CDCl$_3$, 233 K) δ 7.43–7.34 (m, 5H), 5.74 (s, 1H), 5.20 (d, *J* = 12.1 Hz, 1H), 5.15 (d, *J* = 12.1 Hz, 1H), 3.94 (d, *J* = 12.3 Hz, 1H), 3.92 (dd, *J* = 11.7, 3.6 Hz, 1H), 3.79 (dd, *J* = 13.2, 2.6 Hz, 1H), 3.60 (dd, *J* = 12.3, 2.4 Hz, 1H), 3.51 (td, *J* = 12.3, 2.6 Hz, 1H), 3.42 (ddd, *J* = 13.2, 12.3, 3.6 Hz, 1H),

^{13}C NMR (151 MHz, CDCl$_3$, 233 K) δ 155.8, 135.3, 128.7, 128.6, 128.5, 68.9, 68.2, 66.7, 66.0, 39.9.

benzyl 2-azidoazepane-1-carboxylate (9k)

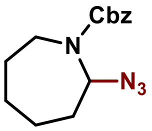

Following the general procedure, the reaction was performed with azepane (19.8 mg, 0.200 mmol), $CbzN_3$ (106 mg, 0.600 mmol, 3.00 equiv.) and **1** (9.0 mg, 0.020 mmol, 10 mol%) in MeCN (1.0 mL, 0.20 M) for 24 h under 456 nm LEDs irradiation. The crude mixture was purified by flash column chromatography (EtOAc/pentane = 5:95) to afford the 2 rotamers (ratio ~ 1.2:1) in 31% yield (16.8 mg, 0.611 mmol) as a colorless oil.

R_f = 0.55 (EtOAc/pentane = 1:9, stained with $KMnO_4$ and visualized under UV light)

HRMS (ESI positive): calc'd for $C_{14}H_{18}N_4O_2Na_1$ $[M+Na]^+$: 297.132194, found: 297.132100.

NOTE: The two rotamers were assigned by 2D NMR spectroscopy.

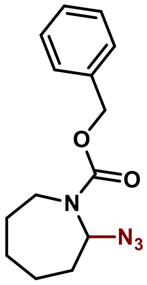

Major Rotamer

^{1}H NMR (600 MHz, $CDCl_3$, 298 K) δ 7.41–7.31 (m, 5H), 5.82 (dd, *J* = 10.8, 6.7 Hz, 1H), 5.21 (s, 2H), 3.88–3.80 (m, 1H), 3.05–2.96 (m, 1H), 2.28–2.20 (m, 1H), 1.87–1.78 (m, 1H), 1.78–1.69 (m, 2H), 1.57–1.42 (m, 2H), 1.37–1.16 (m, 2H).

^{13}C NMR (151 MHz, $CDCl_3$, 298 K) δ 157.1, 136.4, 128.7, 128.3, 127.9, 73.2, 67.8, 42.1, 33.9, 29.57, 29.1, 23.2.

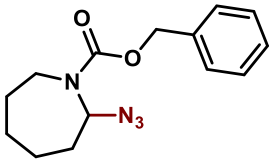

Minor Rotamer

^{1}H NMR (600 MHz, $CDCl_3$, 298 K) δ 7.41–7.31 (m, 5H), 5.65 (dd, *J* = 10.6, 6.7 Hz, 1H), 5.18 (ABq, *J* = 12.1 Hz, 2H), 3.97–3.90 (m, 1H), 3.05–2.96 (m, 1H), 2.21–2.13 (m, 1H), 1.87–1.78 (m, 1H), 1.78–1.69 (m, 2H), 1.57–1.42 (m, 2H), 1.37–1.16 (m, 2H).

^{13}C NMR (151 MHz, $CDCl_3$, 298 K) δ 155.7, 136.1, 128.7, 128.5, 128.4, 73.1, 68.1, 42.4, 34.2, 29.63, 28.6, 23.4,

(9l)

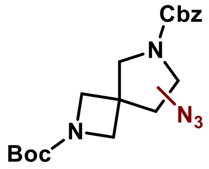

Following the general procedure, the reaction was performed with *tert*-butyl-2,6-diazaspiro[3.4]octan-2-carboxylate (42.5 mg, 0.200 mmol), $CbzN_3$ (106 mg, 0.600 mmol, 3.00 equiv.) and **1** (9.0 mg, 0.020 mmol, 10 mol%) in MeCN (1.0 mL, 0.20 M) for 24 h under 456 nm LEDs irradiation. The regioisomeric ratio was determined to be a 1.5:1 ratio by ^{1}H NMR spectroscopy of the crude reaction mixture The crude mixture was purified by flash column chromatography (EtOAc/pentane = 2:8) to afford the two regioisomers were isolated separately in 30% combined yield.

***Major regioisomer*: (6-benzyl 2-(*tert*-butyl) 5-azido-2,6-diazaspiro[3.4]octane-2,6-dicarboxylate)**

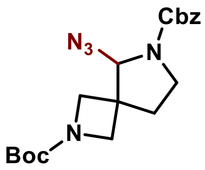

14% yield (10.9 mg, 0.0281 mmol), a colorless oil.

R_f = 0.5 (EtOAc/pentane = 1:1, stained with Ninhydrin)

HRMS (ESI positive): calc'd for $C_{19}H_{25}N_5O_4Na_1$ $[M+Na]^+$: 410.17988, found: 410.17958.

NOTE: The sample was characterized at 273 K, where the most signals were reasonably sharper. Due to extensive spectral overlap, individual rotamers were not assigned.

^{1}H NMR (600 MHz, $CDCl_3$, 273 K) δ 7.41–7.32 (m, 5H), 5.56 (s, 0.4H), 5.45 (s, 0.6H), 5.23–5.11 (m, 2H), 4.06 (d, J = 9.5 Hz, 0.4H), 3.98 (d, J = 9.5 Hz, 0.6H), 3.75 (d, J = 9.5 Hz, 1.4H), 3.70 (d, J = 8.6 Hz, 0.6H), 3.64 (d, J = 8.4 Hz, 1H), 3.62–3.54 (m, 1H), 3.39–3.24 (m, 1H), 2.20–2.07 (m, 2H), 1.45–1.39 (m, 9H).

^{13}C NMR (151 MHz, $CDCl_3$, 273 K) δ 156.12, 156.08, 155.5, 154.3, 135.9, 135.5, 128.9, 128.72, 128.70, 128.65, 128.5, 128.3, 80.2, 80.1, 79.4, 68.2, 67.8, 59.5, 58.2, 53.8, 52.5, 44.2, 43.94, 43.93, 43.2, 32.9, 31.8, 28.41, 28.40.

The regioselectivity is supported by the characteristic ^{15}N HMBC cross-peak, as well as two signlets peak at 5.56 and 5.45 ppm.

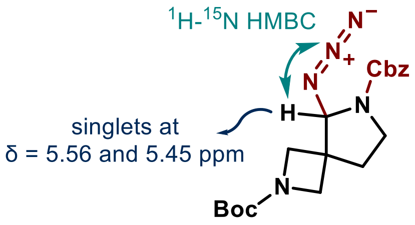

***Minor regioisomer*: (6-benzyl 2-(*tert*-butyl) 7-azido-2,6-diazaspiro[3.4]octane-2,6-dicarboxylate**

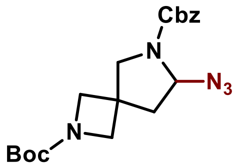

16% yield (12.7 mg, 0.0328 mmol), a colorless oil.

R_f = 0.35 (EtOAc/pentane = 1:1, stained with Ninhydrin)

HRMS (ESI positive): calc'd for $C_{19}H_{25}N_5O_4Na_1$ $[M+Na]^+$: 410.17988, found: 410.17967.

NOTE: Due to extensive spectral overlap, individual rotamers were not assigned.

^{1}H NMR (600 MHz, CDCl$_3$, 298 K) δ 7.41–7.30 (m, 5H), 5.64 (d, *J* = 5.5 Hz, 0.5H), 5.52 (d, *J* = 5.6 Hz, 0.5H), 5.26–5.10 (m, 2H), 3.99 (dd, *J* = 8.9, 2.5 Hz, 1H), 3.93–3.78 (m, 4H), 3.55–3.41 (m, 1H), 2.21–2.08 (m, 2H), 1.43 (s, 9H).

^{13}C NMR (151 MHz, CDCl$_3$, 298 K) δ 156.2, 155.2, 154.1, 136.1, 135.8, 128.82, 128.75, 128.7, 128.6, 128.5, 128.3, 80.0, 75.1, 74.4, 68.1, 67.8, 62.0, 56.5, 56.3, 56.1, 43.0, 42.1, 38.8, 37.9, 28.5.

The regioselectivity is supported by the COSY and NOESY correlation data and well as the ^{15}N-HMBC cross peak.

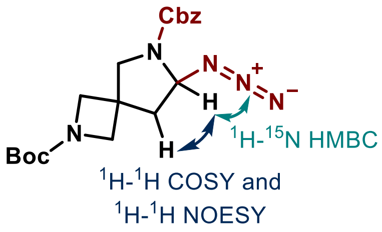

2-(trimethylsilyl)ethyl 2-azidopyrrolidine-1-carboxylate (9m)

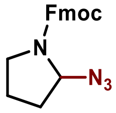

Following the general procedure, the reaction was performed with pyrrolidine (14.2 mg, 0.200 mmol), $FmocN_3$ (159 mg, 0.600 mmol, 3.00 equiv.) and **1** (9.0 mg, 0.020 mmol, 10 mol%) in MeCN (1.0 mL, 0.20 M) for 24 h under 456 nm LEDs irradiation. The crude mixture was purified by flash column chromatography (EtOAc/pentane = 5:95) to afford the product in 20% yield (13.5 mg, 0.0404 mmol) as a viscous oil.

R_f = 0.3 (EtOAc/pentane = 1:9, stained with KMnO4, UV)

NOTE: Due to extensive signal overlap and the diastereotopic nature of Fmoc group, the rotamers are not assigned individually.

^{1}H NMR (600 MHz, $CDCl_3$, 298 K) δ 7.77 (d, *J* = 7.6 Hz, 2H), 7.67–7.57 (m, 2H), 7.41 (t, *J* = 7.5 Hz, 2H), 7.36–7.30 (m, 2H), 5.58 (d, *J* = 4.7 Hz, 0.5H), 5.39–5.09 (m, 0.5H), 4.55–4.47 (m, 1.5H),4.45–4.39 (m, 0.5H), 4.32–4.23 (m, 1H), 3.62–3.56 (m, 1H), 3.42–3.32 (m, 1H), 2.12–1.86 (m, 4H).

^{13}C NMR (151 MHz, $CDCl_3$, 298 K) δ 155.5, 154.5, 144.2, 144.0, 143.9, 143.7, 141.6, 141.51, 141.48, 141.45, 127.9, 127.3, 127.2, 125.21, 125.15, 125.10, 120.2, 75.1, 74.2, 67.9, 67.8, 47.4, 47.3, 46.6, 46.2, 33.6, 32.5, 23.22, 22.17.

HRMS (ESI positive): calc'd for $C_{19}H_{18}N_4O_2Na_1$ $[M+Na]^+$: 357.132194, found: 357.132370.

2-(trimethylsilyl)ethyl 2-azidopyrrolidine-1-carboxylate (9n)

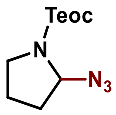

Following the general procedure, the reaction was performed with pyrrolidine (14.2 mg, 0.200 mmol), $TeocN_3$ (112 mg, 0.600 mmol, 3.00 equiv.) and **1** (9.0 mg, 0.020 mmol, 10 mol%) in MeCN (1.0 mL, 0.20 M) for 24 h under 456 nm LEDs irradiation. The crude mixture was purified by flash column chromatography (EtOAc/pentane = 5:95) to afford the 2 rotamers (ratio ~ 1:1) in 57% yield (29.0 mg, 0.113 mmol) as a colorless oil.

R_f = 0.5 (EtOAc/pentane = 1:9, stained with Ninhydrin and $KMnO_4$)

HRMS (ESI positive): calc'd for $C_{10}H_{20}N_4O_2Si_1Na_1$ $[M+Na]^+$: 279.124772, found: 279.124820.

Rotamer 1

^{1}H NMR (600 MHz, CDCl$_3$, 298 K) δ 5.58–5.55 (m, 1H), 4.26–4.19 (m, 2H), 3.54–3.49 (m, 1H), 3.30 (td, *J* = 10.1, 6.6 Hz, 1H), 2.07–1.83 (m, 4H), 1.10–0.96 (m, 2H), 0.04 (s, 9H).

^{13}C NMR (151 MHz, CDCl$_3$, 298 K) δ 155.9, 75.1, 64.2, 46.1, 32.6, 23.2, 17.8, −1.3.

Rotamer 2

^{1}H NMR (600 MHz, CDCl$_3$, 298 K) δ 5.46 (d, *J* = 4.7 Hz, 1H), 4.26–4.19 (m, 2H), 3.56 (td, *J* = 8.1, 4.0 Hz, 1H), 3.40–3.34 (m, 1H), 2.07–1.83 (m, 4H), 1.10–0.96 (m, 2H), 0.04 (s, 9H).

^{13}C NMR (151 MHz, CDCl$_3$, 298 K) δ 154.9, 74.4, 64.3, 46.4, 33.5, 22.2, 17.9, −1.4.

neopentyl 2-azidopyrrolidine-1-carboxylate (9o)

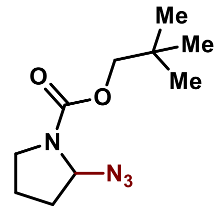

Following the general procedure, the reaction was performed with pyrrolidine (14.2 mg, 0.200 mmol), neopentyl carbonazidate (94.3 mg, 0.600 mmol, 3.00 equiv.) and **1** (9.0 mg, 0.020 mmol, 10 mol%) in MeCN (1.0 mL, 0.20 M) for 24 h under 456 nm LEDs irradiation. The crude mixture was purified by flash column chromatography (EtOAc/pentane = 5:95) to afford the 2 rotamers (ratio ~ 1.1:1) in 46% yield (21 mg, 0.091 mmol) as a colorless oil.

R_f = 0.5 (EtOAc/pentane = 1:9, stained with Ninhydrin)

HRMS (ESI positive): calc'd for $C_{10}H_{18}N_4O_2Na_1$ $[M+Na]^+$: 249.132194, found: 249.132490.

Major rotamer

^{1}H NMR (600 MHz, CDCl$_3$, 298 K) δ 5.58–5.53 (m, 1H), 3.85 (d, *J* = 10.3 Hz, 1H), 3.80 (d, *J* = 10.1 Hz, 1H), 3.58 (t, *J* = 9.2 Hz, 1H), 3.37–3.32 (m, 1H), 2.08–1.85 (m, 4H), 0.95 (s, 9H).

^{13}C NMR (151 MHz, CDCl$_3$, 298 K) δ 155.9, 75.2, 75.0, 46.1, 32.5, 31.7, 26.6, 23.1.

Minor rotamer

^{1}H NMR (600 MHz, CDCl$_3$, 298 K) δ 5.45 (d, *J* = 4.3 Hz, 1H), 3.91 (d, *J* = 10.2 Hz, 1H), 3.81 (d, *J* = 10.2 Hz, 1H), 3.58 (t, *J* = 9.2 Hz, 1H), 3.44–3.36 (m, 1H), 2.08–1.85 (m, 4H), 0.98 (s, 9H),

^{13}C NMR (151 MHz, CDCl$_3$, 298 K) δ 154.8, 75.5, 74.3, 46.5, 33.6, 31.6, 26.7, 22.1.

prop-2-yn-1-yl 2-azidopyrrolidine-1-carboxylate (9p)

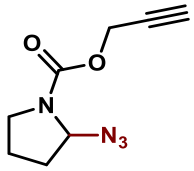

Following the general procedure, the reaction was performed with pyrrolidine (14.2 mg, 0.200 mmol), prop-2-yn-1-yl carbonazidate (75.1 mg, 0.600 mmol, 3.00 equiv.) and **1** (9.0 mg, 0.020 mmol, 10 mol%) in MeCN (1.0 mL, 0.20 M) for 24 h under 456 nm LEDs irradiation. The reaction mixture was diluted with $CDCl_3$, and the yield was determined by ^{1}H NMR spectroscopy (41% yield) using trichloroethylene as an internal standard. The crude mixture was purified by flash column chromatography (Et_2O/DCM = 2:98) to afford the 2 rotamers (ratio ~ 1:1) in 26% yield (9.9 mg, 0.051 mmol) as a yellow oil. The discrepancy between the yield between determined by ^{1}H NMR spectroscopy and isolated yield was a result of the volatility and instability of the compound during flash column chromatography.

R_f = 0.6 (Et_2O/DCM = 5:95, stained with Ninhydrin)

NOTE: We did not attempt to assign the signals to individual rotamers due to the exchange observed in the 1D selective TOCSY experiments, as well as extensive signal overlap.

^{1}H NMR (600 MHz, $CDCl_3$, 298 K) δ 5.57 (d, *J* = 4.6 Hz, 0.5H), 5.50 (d, *J* = 4.7 Hz, 0.5H), 4.84–4.76 (m, 1H), 4.76–4.69 (m, 1H), 3.63–3.54 (m, 1H), 3.45–3.33 (m, 1H), 2.49 (t, *J* = 2.5 Hz, 1H), 2.09–1.85 (m, 4H).

^{13}C NMR (151 MHz, $CDCl_3$, 298 K) δ 154.7, 153.5, 78.1, 77.9, 75.17, 75.10, 75.0, 74.3, 53.3, 46.6, 46.2, 33.5, 32.5, 23.2, 22.2.

HRMS (ESI positive): calc'd for $C_8H_{10}N_4O_2Na_1$ $[M+Na]^+$: 217.06960, found: 217.06951.

(1S,2R,5S)-2-isopropyl-5-methylcyclohexyl 2-azidopyrrolidine-1-carboxylate (9q)

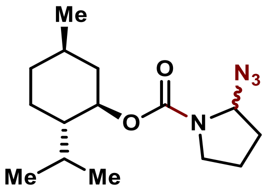

Following the general procedure, the reaction was performed with pyrrolidine (14.2 mg, 0.200 mmol), (1S,2R,5S)-2-isopropyl-5-methylcyclohexyl carbonazidate (135 mg, 0.600 mmol, 3.00 equiv.) and **1** (9.0 mg, 0.020 mmol, 10 mol%) in MeCN (1.0 mL, 0.20 M) for 24 h under 456 nm LEDs irradiation. The diastereomeric ratio was not assigned due to the complexity of ^{1}H NMR spectrum arising from the rotameric nature of each diastereomer of the product. The crude mixture was purified by flash column chromatography (EtOAc/pentane = 3:97) to afford a mixture of both diastereomers (ratio ~ 1.2:1) in 38% yield (22 mg, 0.075 mmol) as colorless oil.

R_f = 0.8 (EtOAc/pentane = 1:9, stained with $KMnO_4$)

NOTE: At room temperature, the signals appeared slightly broadened. Therefore, the sample was characterized at 253 K, where the signals appeared significantly sharper. At this temperature, the major diastereomer consists of two rotamers in a ratio of ~1:0.6, and the minor diastereomer shows two rotamers in a ratio of ~1:0.8. Due to the high complexity of the molecule, the signals—particularly the ^{13}C shifts—were assigned only to their respective positions.

^{1}H NMR (600 MHz, $CDCl_3$, 253 K) δ 5.61–5.57 (m, 0.2H, minor diastereomer), 5.59–5.54 (m, 0.2H, major diastereomer), 5.48–5.44 (m, 0.3H, major diastereomer), 5.40–5.36 (m, 0.3H, minor diastereomer), 4.72 (td, *J* = 11.0, 4.5 Hz, 0.3H), 4.63–4.55 (m, 0.7H), 3.57–3.50 (m, 1H), 3.39–3.23 (m, 1H), 2.11–2.06 (m, 0.5 H), 2.06–1.82 (m, 5.5H), 1.65 (dq, *J* = 9.2, 5.3 Hz, 2H), 1.54–1.32 (m, 2H), 1.11–0.94 (m, 2H), 0.91–0.80 (m, 7H), 0.79 – 0.72 (m, 3H).

^{13}C NMR (151 MHz, $CDCl_3$, 253 K) δ 155.5, 155.4, 154.5, 154.4, 75.9, 75.7, 75.5, 75.4, 74.94, 74.93, 74.7, 74.5, 47.2, 47.1, 47.0, 46.9, 46.4, 46.3, 46.1, 41.7, 41.4, 41.1, 40.3, 34.2, 34.14, 34.05, 34.02, 33.7, 33.5, 32.43, 32.39, 31.5, 31.4, 31.3, 26.22, 26.17, 26.1, 25.7, 23.24, 23.15, 23.1, 22.6, 22.3, 22.22, 22.21, 22.17, 22.16, 21.1, 21.04, 21.00, 20.99, 16.4, 15.9.

HRMS (ESI positive): calc'd for $C_{15}H_{26}N_4O_2Na_1$ $[M+Na]^+$: 317.194794, found: 317.195170.

(S)-1-(benzyloxy)-1-oxopropan-2-yl 2-azidopyrrolidine-1-carboxylate (9r)

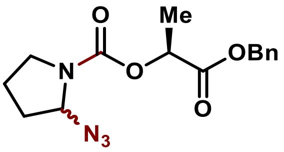

Following the general procedure, the reaction was performed with pyrrolidine (14.2 mg, 0.200 mmol), benzyl (S)-2-((azidocarbonyl)oxy)propanoate **8r** (150 mg, 0.600 mmol, 3.00 equiv.) and Bi complex **1** (9.0 mg, 0.020 mmol, 10 mol%) in MeCN (1.0 mL, 0.20 M) for 24 h under 456 nm LEDs irradiation. The diastereomeric ratio was not assigned due to the complexity of ^{1}H NMR spectrum arising from the rotameric nature of each diastereomer of the product. The crude mixture was purified by flash column chromatography (EtOAc/pentane = 15:85) to afford a mixture of both diastereomers, each having two rotamers in 39% yield (25 mg, 0.077 mmol) as a colorless oil.

R_f = 0.3 (EtOAc/pentane = 2:8, stained with $KMnO_4$ and visualized under UV light)

NOTE: The sample contains a mixture of 2 diastereomers and 2 rotamers of the title compound. Due to extensive overlap in the ^{1}H NMR spectrum, we were unable to resolve the signals corresponding to the individual diastereomers and rotamers of the compound. The NMR experiments at higher temperature to attempt to average out the rotamers resulted in the decomposition of the compound. Based on the acquired data, the rotamer ratios of the two diastereomers are not identical. The presence of the azide group is supported by the characteristic ^{15}N HMBC cross-peak.

^{1}H NMR (600 MHz, $CDCl_3$, 298 K) δ 7.39–7.30 (m, 5H), 5.62–5.45 (m, 1H), 5.27–5.09 (m, 3H), 3.68–3.52 (m, 1H), 3.48–3.32 (m, 1H), 2.10–1.82 (m, 4H), 1.57–1.49 (m, 3H).

^{13}C NMR (151 MHz, $CDCl_3$, 298 K) δ 171.34, 171.31, 171.0, 170.8, 154.84, 154.75, 153.68, 153.62, 135.60, 135.59, 135.50, 135.47, 128.75, 128.72, 128.70, 128.65, 128.56, 128.52, 128.44, 128.41, 128.25, 128.21, 75.14, 74.85, 74.77, 74.64, 70.10, 69.86, 69.83, 69.67, 67.17, 67.11, 67.08, 67.07, 46.50, 46.47, 46.27, 46.23, 33.66, 33.43, 32.56, 32.46, 23.13, 23.09, 22.26, 22.22, 17.36, 17.35, 17.19, 17.07.

HRMS (ESI positive): calc'd for $C_{15}H_{18}N_4O_4Na_1$ $[M+Na]^+$: 341.1220, found: 341.12206.

(1S,2R,4S)-1,7,7-trimethylbicyclo[2.2.1]heptan-2-yl 2-azidopyrrolidine-1-carboxylate (9s)

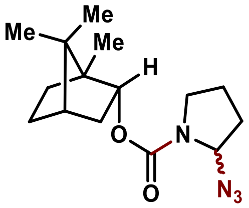

Following the general procedure, the reaction was performed with pyrrolidine (14.2 mg, 0.200 mmol), benzyl (S)-2-((azidocarbonyl)oxy)propanoate **8s** (150 mg, 0.600 mmol, 3.00 equiv.) and Bi complex **1** (9.0 mg, 0.020 mmol, 10 mol%) in MeCN (1.0 mL, 0.20 M) for 24 h under 456 nm LEDs irradiation. The diastereomeric ratio was not assigned due to the complexity of ^{1}H NMR spectrum arising from the rotameric nature of each diastereomer of the product. The crude mixture was purified by flash column chromatography (EtOAc/pentane = 7:93) to afford a mixture of both diastereomers, each having two rotamers in 61% yield (35.5 mg, 0.121 mmol) as a colorless oil.

R_f = 0.3 (EtOAc/pentane = 1:9, stained with $KMnO_4$)

NOTE: The sample contains a mixture of 2 diastereomers and 2 rotamers of the title compound. Due to extensive overlap in the ^{1}H NMR spectrum, we were unable to resolve the signals corresponding to the individual diastereomers and rotamers of the compound. The NMR experiments at higher temperature to attempt to average out the rotamers resulted in the decomposition of the compound. Based on the acquired data, the rotamer ratios of the two diastereomers are not identical. The presence of the azide group is supported by the characteristic ^{15}N HMBC cross-peak.

^{1}H NMR (600 MHz, $CDCl_3$, 298 K) δ 5.61–5.40 (m, 1H), 5.01–4.82 (m, 1H), 3.63–3.52 (m, 1H), 3.44–3.31 (m, 1H), 2.42–2.30 (m, 1H), 2.10–1.85 (m, 5H), 1.81–1.70 (m, 1H), 1.69 (s, 1H), 1.38–1.20 (m, 2H), 1.20–0.99 (m, 1H), 0.94–0.82 (m, 9H).

^{13}C NMR (151 MHz, $CDCl_3$, 298 K) δ 156.1, 156.0, 155.0, 154.9, 82.0, 81.8, 81.4, 81.3, 75.08, 75.05, 74.3, 74.2, 49.13, 49.07, 49.0, 48.9, 48.1, 48.00, 47.94, 47.90, 46.53, 46.51, 46.12, 46.08, 45.04, 45.02, 44.9, 37.4, 37.2, 36.9, 36.0, 33.7, 33.6, 32.6, 32.5, 28.28, 28.23, 28.17, 27.9, 27.7, 27.42, 27.40, 27.35, 23.13, 23.10, 22.11, 22.09, 19.87, 19.03, 19.01, 13.8, 13.67, 13.65, 13.55.

HRMS (ESI positive): calc'd for $C_{15}H_{24}N_4O_2Na_1$ $[M+Na]^+$: 315.17915, found: 315.17903.

Less reactive compounds

1-(*tert*-butyl) 2-methyl (2S,5R)-5-azidopyrrolidine-1,2-dicarboxylate (9t)

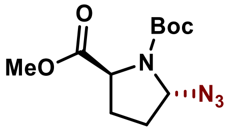

Following the general procedure, the reaction was performed with (*L*)-proline methyl ester (25.8 mg, 0.200 mmol), $BocN_3$ (85.9 mg, 0.600 mmol, 3.00 equiv.) and **1** (9.0 mg, 0.020 mmol, 10 mol%) in MeCN (1.0 mL, 0.20 M) for 24 h under 456 nm LEDs irradiation. The diastereomeric ratio was determined to be a >5:1 ratio by ^{1}H NMR spectroscopy of the crude reaction mixture. The crude mixture was purified by flash column chromatography (EtOAc/pentane = 2:98) to afford the major diastereomer as 2 rotamers (ratio ~ 1:0.8) in 17% yield (10.4 mg, 0.0334 mmol) as a colorless oil.

R_f = 0.6 (EtOAc/pentane = 1:9, visualized under UV light)

HRMS (ESI positive): calc'd for $C_{11}H_{18}N_4O_4Na_1$ $[M+Na]^+$: 293.12203, found: 293.12188.

*NOTE: The relative stereochemistry was assigned based on the comparison to **9u**.*

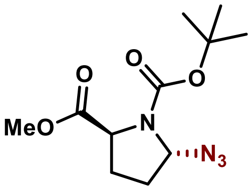

Major rotamer

^{1}H NMR (600 MHz, CDCl$_3$, 298 K) δ 5.60 (d, J = 5.9 Hz, 1H), 4.39 (d, J = 9.2 Hz, 1H), 3.73 (s, 3H), 2.31 (tdd, J = 13.3, 9.3, 6.4 Hz, 1H), 2.15 (dddd, J = 13.7, 13.0, 6.9, 6.0 Hz, 1H), 1.95 (dd, J = 13.0, 6.8 Hz, 1H), 1.82 (dd, J = 12.9, 6.4 Hz, 1H), 1.51 (s, 9H).

^{13}C NMR (151 MHz, CDCl$_3$, 298 K) δ 172.6, 153.4, 82.3, 75.0, 58.9, 52.5, 31.9, 28.31, 27.2

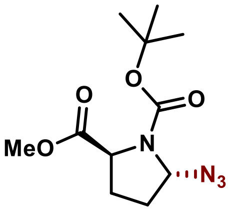

Minor rotamer

^{1}H NMR (600 MHz, CDCl$_3$, 298 K) δ 5.69 (d, J = 6.1 Hz, 1H), 4.33 (d, J = 9.1 Hz, 1H), 3.72 (s, 3H), 2.38 (tdd, J = 13.4, 9.1, 6.7 Hz, 1H), 2.12 (tt, J = 13.3, 6.7 Hz, 1H), 1.98 (dd, J = 13.1, 7.0 Hz, 1H), 1.83 (dd, J = 12.9, 6.5 Hz, 1H), 1.42 (s, 9H).

^{13}C NMR (151 MHz, CDCl$_3$, 298 K) δ 172.9, 153.8, 81.5, 75.1, 59.4, 52.3, 30.9, 28.27, 28.2.

1-benzyl 2-methyl (2S,5R)-5-azidopyrrolidine-1,2-dicarboxylate (9u)

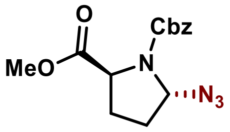

Following the general procedure, the reaction was performed with (*L*)-proline methyl ester (25.8 mg, 0.200 mmol), $CbzN_3$ (106 mg, 0.600 mmol, 3.00 equiv.) and **1** (9.0 mg, 0.020 mmol, 10 mol%) in MeCN (1.0 mL, 0.20 M) for 24 h under 456 nm LEDs irradiation. The diastereomeric ratio was determined to be a >5:1 ratio by ^{1}H NMR spectroscopy of the crude reaction mixture. The crude mixture was purified by flash column chromatography (EtOAc/pentane = 2:98) to afford the major diastereomer as 2 rotamers (ratio ~ 1:1) in 17% yield (10.4 mg, 0.0341 mmol) as a colorless oil.

R_f = 0.6 (EtOAc/pentane = 1:9, visualized under UV light)

HRMS (ESI positive): calc'd for $C_{14}H_{16}N_4O_4Na_1$ $[M+Na]^+$: 327.106374, found: 327.106680.

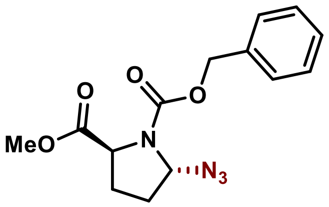

Rotamer A

^{1}H NMR (600 MHz, $CDCl_3$, 298 K) δ 7.42–7.28 (m, 5H), 5.62 (d, J = 6.0 Hz, 1H), 5.22 (d, J = 12.1 Hz, 1H), 5.18 (d, J = 12.1 Hz, 1H), 4.46 (d, J = 9.3 Hz, 1H), 3.74 (s, 3H), 2.34 (tdd, J = 13.5, 9.3, 6.5 Hz, 1H), 2.17 (tt, J = 13.5, 6.5 Hz, 1H), 2.03–1.96 (m, 1H), 1.91–1.83 (m, 1H).

^{13}C NMR (151 MHz, $CDCl_3$, 298 K) δ 172.3, 154.1, 135.7, 128.70, 128.67, 128.5, 74.8, 68.3, 59.3, 52.6, 31.9, 27.3.

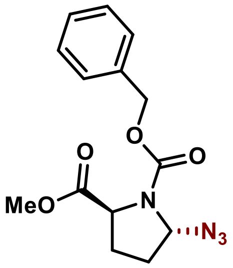

Rotamer B

^{1}H NMR (600 MHz, $CDCl_3$, 298 K) δ 7.42–7.28 (m, 5H), 5.75 (d, J = 6.1 Hz, 1H), 5.26 (d, J = 12.3 Hz, 1H), 5.06 (d, J = 12.3 Hz, 1H), 4.42 (d, J = 9.2 Hz, 1H), 3.55 (s, 3H), 2.39 (tdd, J = 13.5, 9.3, 6.5 Hz, 1H), 2.13 (tt, J = 13.5, 6.5 Hz, 1H), 2.03–1.96 (m, 1H), 1.91–1.83 (m, 1H).

^{13}C NMR (151 MHz, $CDCl_3$, 298 K) δ 172.4, 154.7, 136.0, 128.6, 128.4, 128.1, 75.6, 67.8, 59.1, 52.4, 30.8, 28.4.

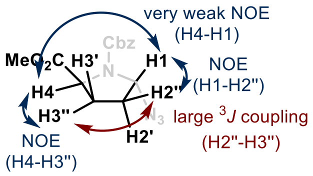

A very weak NOE is observed between H1 and H4, and a stronger NOE would generally be expected for a *cis* isomer. Due to the absence of protons at the adjacent positions to C1 and C4, no additional NOEs across the ring could be observed. The ^3J coupling between H2″ and H3″ is rather large (~13.5 Hz), which is most likely a *trans* coupling, as no NOE between these two atoms is observed in the 2D NOESY —only a COSY-type cross peak. H1 shows an intense NOE to H2″, whereas H4 shows an intense NOE to H3″. Taken together, these observations support the proposed relative configuration shown above.

7. Derivatization of α-azido product 9c

7.1. [3+2]-cycloaddition

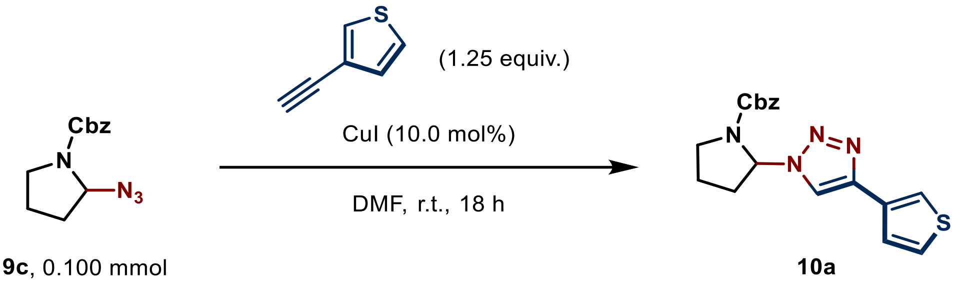

9c, 0.100 mmol **10a**

CuI (1.90 mg, 0.0100 mmol, 10.0 mol%) was added to a solution of 3-ethynl thiophene (12.0 µL, 0.125 mmol, 1.25 equiv.) and **9c** (24.6 mg, 0.100 mmol, 1.00 equiv.) in anhydrous DMF (0.5 mL, 0.2 M) under a positive counterflow of an argon atmosphere. The reaction mixture was then stirred at °C for 18 h after which the reaction was diluted with EtOAc and washed 3 times with 10% w/v LiCl solution. The organic layer was then dried over anhydrous $MgSO_4$, filtered and concentrated under reduced pressure on a rotary evaporator. The crude residue was purified by flash column chromatography (EtOAc/pentane = 0:10 to 4:6) to afford the product in 31% yield (10.9 mg, 0.031 mmol) as a colorless oil.

R_f = 0.16 (EtOAc/pentane = 4:6, visualized under UV light).

NOTE: ^{1}H and ^{13}C NMR spectra are complicated due to the presence of rotamers at room temperature.

^{1}H NMR (400 MHz, $CDCl_3$, 298 K) δ 7.74–7.67 (m, 1H), 7.59 (d, *J* = 7.6 Hz, 2H), 7.47–7.28 (m, 3H), 7.24–7.13 (m, 3H), 6.54–6.36 (m, 1H), 5.26–4.98 (m, 2H), 3.90 (q, *J* = 10.2 Hz, 1H), 3.61 (dq, *J* = 17.0, 8.6 Hz, 1H), 2.53–2.24 (m, 3H), 2.08–1.97 (m, 1H).

^{13}C NMR (151 MHz, Acetone-d_6, 298K) 154.9, 154.4, 144.6, 137.9, 137.74, 133.02, 132.97, 132.1, 131.9, 129.3, 129.0, 128.7, 128.6, 128.4, 127.9, 127.5, 126.8, 122.4, 122.4, 77.4, 76.7, 67.4, 67.1, 47.8, 47.5, 34.3, 33.5, 23.5, 22.6.

HRMS (ESI): calc'd for $C_{18}H_{18}N_4O_2S_1Na_1$ $[M+Na]^+$: 377.10427, found: 377.10392.

7.2. Phosphoroamidation

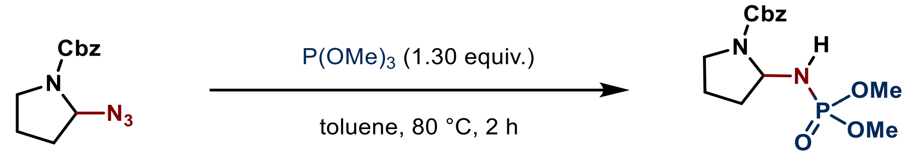

9c, 0.100 mmol **10b**

$P(OMe)_3$ (15 μL, 0.13 mmol, 1.3 equiv.) was added to a stirred solution of benzyl 2-azidopyrrolidine-1-carboxylate (**9c**) (24.6 mg, 0.100 mmol, 1.00 equiv.) in toluene (1.0 mL, 0.10 M) under an argon atmosphere. The reaction was heated to 80 °C and stirred for a further 2 h. Upon completion the solvent was removed under a constant stream of argon and the crude residue was purified by flash column chromatography (EtOH/EtOAc = 0:10 to 8:2) to afford the two rotamers (ratio ~ 1.2:1) in yield 76% yield (25.0 mg, 0.0730 mmol) as a colorless oil.

R_f = 0.1 (EtOAc, stained with $KMnO_4$).

NOTE: The sample was characterized at 233 K, where the most signals were reasonably sharper. We did not attempt to assign the signals to individual rotamers

^{1}H NMR (400 MHz, $CDCl_3$, 233 K) δ 7.47–7.30 (m, 5H), 5.22 (d, *J* = 12.1, 0.45H), 5.12–5.05 (m, 5H), 3.76 (dd, *J* = 11.3, 1.4 Hz, 1.65H), 3.71 (dd, *J* = 11.2, 0.9 Hz, 1.65H), 3.56 (d, *J* = 11.2 Hz, 1.35H), 3.47 (dd, *J* = 11.3, 1.4 Hz, 1.35H), 2.02–1.89 (m, 4H).

^{13}C NMR (101 MHz, $CDCl_3$, 233 K) δ 154.6, 154.5, 136.3, 136.0, 128.6, 128.6, 128.4, 128.3, 128.2, 128.0, 67.3, 66.8, 66.3 (dd, *J* = 14.6, 2.2 Hz), 65.8 (dd, *J* = 15.2, 2.5 Hz), 53.5 (d, *J* = 2.3 Hz), 53.4 (d, *J* = 1.9 Hz), 53.2 (d, *J* = 5.7 Hz), 53.0 (d, *J* = 5.2 Hz), 46.1, 45.8, 34.3 (dd, *J* = 11.7, 2.3 Hz), 33.1 (dd, *J* = 13.6, 2.2 Hz), 22.7 (d, *J* = 2.8 Hz), 21.7 (d, *J* = 2.9 Hz).

^{31}P {^{1}H} NMR (243 MHz, $CDCl_3$, 233 K) δ 8.42, 8.12

HRMS (ESI): calc'd for $C_{14}H_{21}N_2O_5P_1Na_1$ $[M+Na]^+$: 351.10803, found: 351.10768.

.

7.3. Methoxylation

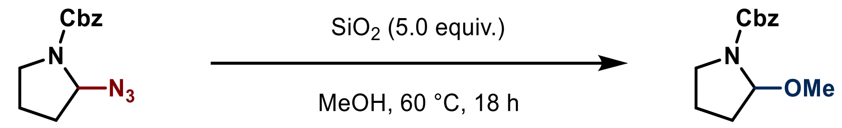

9c, 0.10 mmol **10c**

The reaction was carried out under air. **9c** (25 mg, 0.10 mmol) and SiO_2 (30 mg, 0.50 mmol, 5.0 equiv.) were dissolved in MeOH (2.0 mL, 0.050 M) in a reaction vial equipped with a Teflon-coated magnetic stir, and it was stirred at 60 °C for 18 h. It was quenched by adding water. The organic phase was extracted by Et_2O, and concentrated under reduced pressure. The resulting crude material was then purified by flash column chromatography (Et_2O/DCM = 5:95) to afford the product in 78% yield (18.3 mg, 0.0778 mmol) as colorless oil

R_f = 0.5 (Et_2O/DCM = 1:9, stained with $KMnO_4$)

^{1}H NMR (600 MHz, $CDCl_3$, 298 K) δ 7.39–7.29 (m, 5H), 5.25–5.12 (m, 3H), 3.52 (t, *J* = 9.5 Hz, 1H), 3.49–3.33 (m, 3H), 3.26 (s, 1 H), 2.13–2.00 (m, 1H), 1.97–1.86 (m, 2H), 1.81–1.71 (m, 1H).

^{13}C NMR (151 MHz, $CDCl_3$, 298 K) δ 156.0, 155.1, 136.8, 136.7, 128.6, 128.2, 128.0, 89.3, 88.7, 67.3, 67.0, 56.1, 55.5, 46.1, 45.9, 32.7, 32.1, 22.8, 21.9.

HRMS (ESI positive): calc'd for $C_{13}H_{17}N_1O_3Na_1$ $[M+Na]^+$: 258.110063, found: 258.109950.

The spectral data matched with those reported in the literature.[32]

7.4. Hydroxylation

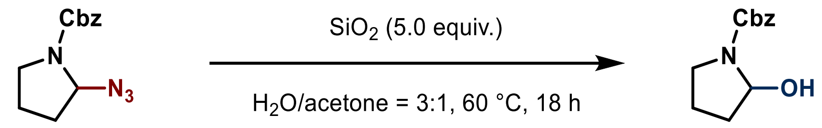

9c, 0.10 mmol **10d**

The reaction was carried out under air. **9c** (25 mg, 0.10 mmol) and SiO_2 (30 mg, 0.50 mmol, 5.0 equiv.) were dissolved in the mixture of H_2O/acetone (2.0 mL, 0.050 M, 3:1) in a reaction vial equipped with a Teflon-coated magnetic stir, and it was stirred at 60 °C for 18 h. The organic phase was extracted by Et_2O, and concentrated under reduced pressure. The resulting crude material was then purified by flash column chromatography (EtOAc/pentane = 1:1) to afford the 2 rotamers (ratio ~ 2.1:1) 84% yield (18.6 mg, 0.0841 mmol) as colorless oil

R_f = 0.3 (EtOAc/pentane = 1:1, stained with $KMnO_4$)

HRMS (ESI positive): calc'd for $C_{12}H_{15}N_1O_3Na_1$ $[M+Na]^+$: 244.094413, found: 244.094410.

NOTE: The sample was characterized at 233 K, where the most signals were reasonably sharper. The two rotamers were assigned by 2D NMR and 1D selective TOCSY spectroscopy.

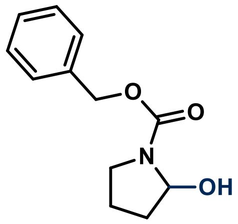

Major rotamer

^{1}H NMR (600 MHz, $CDCl_3$, 233 K) δ 7.41–7.31 (m, 5H), 5.53 (ddd, *J* = 5.6, 2.6, 1.9 Hz, 1H), 5.12 (s, 2H), 4.02 (dd, *J* = 2.6, 1.3 Hz, 1H), 3.65–3.55 (m, 1H), 3.40–3.26 (m, 1H), 2.14–2.01 (m, 1H), 2.01–1.82 (m, 3H).

^{13}C NMR (151 MHz, $CDCl_3$, 233 K) δ 155.4, 136.1, 128.6, 128.2, 128.0, 81.9, 66.9, 45.9, 32.6, 22.9.

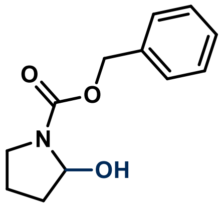

Minor rotamer

^{1}H NMR (600 MHz, $CDCl_3$, 233 K) δ 7.41–7.31 (m, 5H), 5.48 (dt, *J* = 4.7, 2.4 Hz, 1H), 5.19 (d, *J* = 12.2 Hz, 1H), 5.13 (d, *J* = 12.2 Hz, 1H), 3.65–3.55 (m, 1H), 3.40–3.26 (m, 1H), 3.11 (d, *J* = 2.7 Hz, 1H), 2.14–2.01 (m, 1H), 2.01–1.82 (m, 3H).

^{13}C NMR (151 MHz, $CDCl_3$, 233 K) δ 154.1, 136.0, 128.7, 128.4, 128.3, 81.2, 67.2, 46.3, 33.4, 22.1.

7.5. Allylation

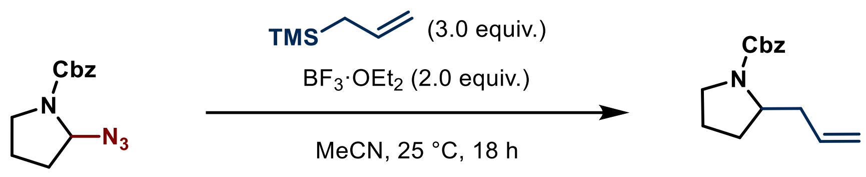

9c, 0.100 mmol **10e**

BF_3·OEt_2 (25 µL, 0.20 mmol, 2.0 equiv.) and allyltrimethylsilane (48 µL, 0.30 mmol, 3.0 equiv.) were added sequentially to a solution of **9c** (24.6 mg, 0.100 mmol, 1.00 equiv.) in anhydrous MeCN (1.0 mL, 0.10 M) under an argon atmosphere. The reaction mixture was then stirred at 25 °C after which the reaction mixture was partitioned between brine and EtOAc. The organic fraction was retained and then sequentially dried over anhydrous $MgSO_4$, filtered and concentrated under reduced pressure on a rotary evaporator. The crude residue was purified by flash column chromatography (EtOAc/pentane = 0:10 to 1:9) to afford the product in 87% yield (21.4 mg, 0.087 mmol) as a yellow oil.

NOTE: ^{1}H and ^{13}C NMR spectra are complicated due to the presence of rotamers at room temperature.

R_f = 0.36 (EtOAc/Pentane = 1:9, stained with $KMnO_4$).

^{1}H NMR (400 MHz, $CDCl_3$, 298 K) δ 7.42–7.27 (m, 5H), 5.74 (tq, *J* = 24.7, 7.5 Hz, 1H), 5.22–5.07 (m, 2H), 5.07–4.97 (m, 2H), 3.92 (s, 1H), 3.54–3.35 (m, 2H), 2.65–2.37 (m, 1H), 2.15 (dq, *J* = 15.0, 7.7 Hz, 1H), 1.98–1.67 (m, 4H).

^{13}C NMR (101 MHz, $CDCl_3$, 298 K) δ 155.1, 154.9, 154.9, 137.3, 137.1, 135.2, 135.0, 128.6, 128.0, 127.9, 117.41, 117.35, 66.8, 66.6, 57.4, 56.9, 39.1, 38.2, 30.1, 29.3, 23.8, 23.0.

HRMS (ESI): calc'd for $C_{15}H_{19}N_1O_2Na_1$ $[M+Na]^+$: 268.13080, found: 268.13098.

The spectral data matched with those reported in the literature.[33]

7.6. Arylation

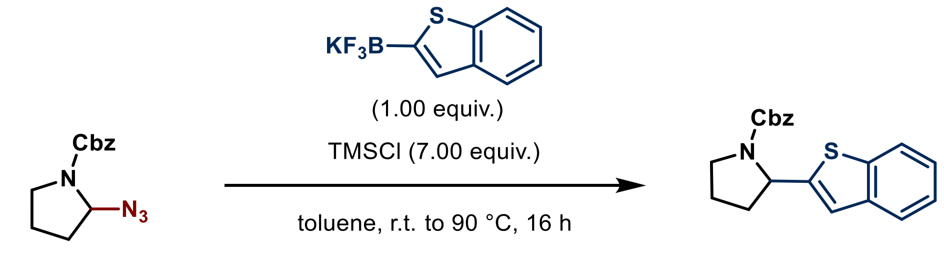

9c, 0.100 mmol **10f**

TMSCl (89.0 µL, 0.700 mmol, 7.00 equiv.) was added dropwise to a solution of potassium benzo[b]thiophen-2-yltrifluoroborate (24.0 mg, 0.100 mmol, 1.00 equiv.) in anhydrous toluene (0.71 mL, 0.14 M) under an argon atmosphere at 25 °C. The reaction was stirred for 10 min, after which the **9c** (32.0 mg, 0.130 mmol, 1.30 equiv.) was added neat and the reaction mixture was then stirred at 90 °C for 16 h. The reaction was then cooled down to 0 °C and quenched by the dropwise addition of water, followed by the addition of aq. sat. $NaHCO_3$. The biphasic mixture was then extracted with EtOAc and aqueous phase was extracted twice more with EtOAc. The combined organic layers were dried over anhydrous $MgSO_4$, filtered and concentrated under reduced pressure on a rotary evaporator. The crude residue was purified by flash column chromatography (EtOAc/pentane = 0:100 to 15:85) to afford the two rotamers (ratio ~ 1.5:1) in 48% yield (16.2 mg, 0.048 mmol) as a viscous yellow-brown oil.

R_f = 0.3 (EtOAc/pentane = 1:9, visualized under UV light).

NOTE: The sample was characterized at 233 K, where the most signals were reasonably sharper. We did not attempt to assign the signals to individual rotamers

^{1}H NMR (600 MHz, $CDCl_3$, 233 K) δ 7.93–7.89 (m, 1.5H, major), 7.85 (ddd, *J* = 7.8, 1.3, 0.7 Hz, 1H, minor), 7.77–7.69 (m, 2.5H, major+minor), 7.44–7.38 (m, 8H, major+minor), 7.38–7.34 (m, 2H, minor), 7.23–7.14 (m, 4.5H, major+minor), 7.07 (d, *J* = 1.1 Hz, 1H, minor), 7.03 (d, *J* = 1.1 Hz, 1H, major), 7.01–6.97 (m, 3H, major+minor), 5.42 (dt, *J* = 8.0, 1.5 Hz, 1H, minor), 5.36 (dt, *J* = 7.9, 1.6 Hz, 1H, major), 5.19 (d, *J* = 12.4 Hz, 1H, minor), 5.12 (d, *J* = 12.5 Hz, 1H, minor), 5.06 (d, *J* = 12.8 Hz, 1H, major), 5.01 (d, *J* = 12.8 Hz, 1H, major), 3.77 (tdd, *J* = 10.6, 8.0, 2.5 Hz, 2.5H, major+minor), 3.61–3.51 (m, 2.5H, major+minor), 2.33–2.27 (m, 1.5H, major), 2.24 (ddt, *J* = 12.1, 8.1, 5.6 Hz, 1H, minor), 2.05–1.98 (m, 2.5H, major+minor), 1.98–1.85 (m, 5H, major+minor).

^{13}C NMR (151 MHz, $CDCl_3$, 233 K) δ 155.0, 154.9, 141.1, 141.0, 137.9, 137.3, 136.81, 136.76, 136.64, 136.58, 128.6, 128.3, 128.1, 128.1, 127.7, 127.4, 124.4, 124.3, 124.0, 123.9, 123.1, 123.0, 121.8, 121.7, 121.2, 120.9, 66.9, 66.5, 57.0, 56.5, 46.9, 46.6, 32.6, 31.7, 23.4, 22.7.

HRMS (EI): calc'd for $C_{20}H_{19}N_1O_2S_1$ $[M]^+$: 337.113101, found: 337.113060.

8. TD-DFT calculations

All calculations were performed using the development version of ORCA 5.0 program suite employing the scalar relativistic zero order regular approximation (ZORA).[34,35] Geometry optimizations were carried out using the BP86 density functional[36] conjunction with the ZORA-Def2-TZVP basis set for H, C, N, O, and S atoms,[37] as well as the SARC-ZORA-TZVPP basis for Bi atom, which features a contraction optimized for the ZORA Hamiltonian at 298.15 K.[38] The RI approximation with SARC/J fitting basis set was employed to accelerate the calculations.[38–41] Furthermore, the atom-pairwise D3 dispersion correction with Becke-Johnson (D3BJ) damping was considered.[42,43] Subsequent frequency calculations revealed that all optimized geometries were local minima with no imaginary frequencies. Single point energy calculations were carried out at the same level of theory. TD-DFT calculations was performed with the optimized geometries at gas phase using the PBE0 functional and the same basis set with 100 roots in total. The Chemcraft 1.8, and Avogadro 1.2 software were used to display molecular geometries.

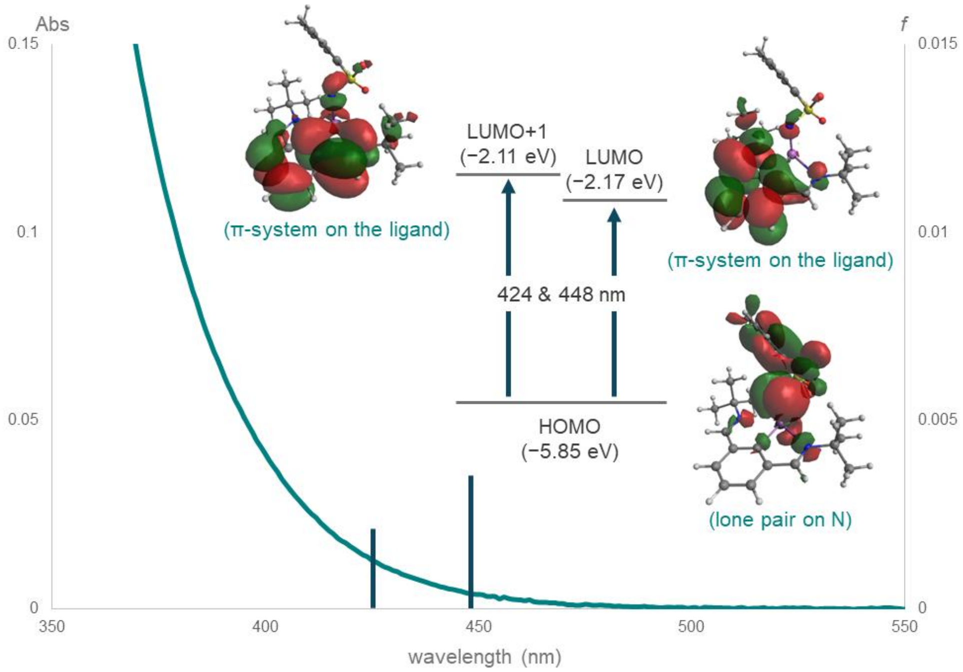

Figure. S7. (A) Experimental UV-vis spectrum for **3b** (green trace) and TD-DFT excited transitions at 448 and 424 nm (blue bars).

The computed UV/vis absorption spectra showed good agreement with the measured excitation spectra. There are two absorptions at 448 and 424 nm, which were assigned as transitions from HOMO to LUMO and HOMO to LUMO+1. While the HOMO corresponds to the lone pair on the anionic nitrogen, the LUMO and LUMO+1 are mainly localized on the π system of the *N,C,N*-pincer ligand. Therefore, absorptions in blue light regime are associated with ligand-to-ligand charge transfer (LLCT) that ultimately forms *N*-centered radical.

XYZ coordinates of DFT optimized structure of **3b**

83	17.262292000	8.702729000	-0.959681000
7	18.408791000	8.380136000	-3.243759000
7	15.035642000	8.112390000	0.213987000
6	15.860789000	7.651661000	-2.388261000
6	16.266256000	7.419730000	-3.704799000
6	15.384976000	6.805372000	-4.613643000
1	15.704350000	6.611703000	-5.640574000
6	14.102698000	6.436969000	-4.198504000
6	13.691685000	6.677643000	-2.884896000
1	12.689051000	6.385621000	-2.563097000
6	14.574520000	7.289508000	-1.976442000
6	17.622825000	7.820474000	-4.089829000
1	17.939095000	7.614566000	-5.122938000
6	14.206558000	7.539974000	-0.580084000
1	13.216492000	7.200101000	-0.240235000
1	13.421315000	5.957089000	-4.901464000
7	18.101472000	6.927371000	-0.184734000
16	19.437846000	7.330359000	0.583091000
8	20.492513000	6.342380000	0.345148000
8	19.814840000	8.762072000	0.377275000
6	19.808371000	8.743584000	-3.566538000
6	14.791349000	8.272211000	1.662916000
6	13.304094000	8.405807000	2.013858000
1	12.842024000	9.248515000	1.478620000
1	12.737757000	7.491228000	1.788499000
1	13.203208000	8.588759000	3.092077000
6	15.406261000	7.029377000	2.332715000
1	14.803569000	6.137701000	2.108150000
1	16.422608000	6.855974000	1.959139000
1	15.450198000	7.166591000	3.421729000
6	15.546732000	9.532583000	2.103565000
1	15.418943000	9.694633000	3.182174000
1	16.624132000	9.423347000	1.916094000
1	15.179160000	10.420621000	1.569286000
6	20.694150000	7.595292000	-3.049240000

1	20.476098000	6.666987000	-3.595939000
1	21.751401000	7.854270000	-3.202244000
1	20.535979000	7.409142000	-1.980817000
6	20.036767000	8.955727000	-5.069453000
1	21.066660000	9.301454000	-5.228237000
1	19.916172000	8.027725000	-5.646334000
1	19.354657000	9.717408000	-5.476113000
6	20.120655000	10.036651000	-2.799336000
1	21.148647000	10.358599000	-3.015449000
1	19.437159000	10.844512000	-3.100124000
1	20.042346000	9.872260000	-1.715230000
6	19.031238000	7.202034000	2.328650000
6	18.811765000	5.937620000	2.887357000
6	18.854491000	8.348280000	3.102487000
6	18.391866000	5.832442000	4.209873000
6	18.432830000	8.228914000	4.429507000
6	18.188105000	6.975416000	5.002788000
1	18.217867000	4.843687000	4.641398000
1	18.298455000	9.129217000	5.034057000
1	18.967754000	5.049164000	2.275216000
1	19.060612000	9.322430000	2.658505000
6	17.738755000	6.845770000	6.435145000
1	16.827754000	6.233530000	6.516165000
1	18.509588000	6.357770000	7.051840000
1	17.529191000	7.828434000	6.878458000

9. EPR experiments

Procedure: In an argon-filled glovebox, **3a** or **3b** (0.010 mmol) was dissolved in dry and degassed MeCN (0.20 mL, 0.050 M) in a vial, which was transferred to an EPR tube. The tube was sealed with a parafilm, and taken out of the glovebox. The sample was frozen in liquid nitrogen and placed at EPR instrument. A fiber-coupled ultra-high power LED from Prizmatix Ltd. (LED head: UHP-T-450 SR, peak wavelength λ = 450 nm) was connected to the optical port of the resonator using a 1 mm optical fiber and a 5 mm collimator and turned on for the experiments under illumination. Unfortunately, we were not able to detect any signals, even at very low temperatures (e.g., 10 K). We believe the lifetime of these putative radical species is extremely short, thus escaping detection at present in this system.

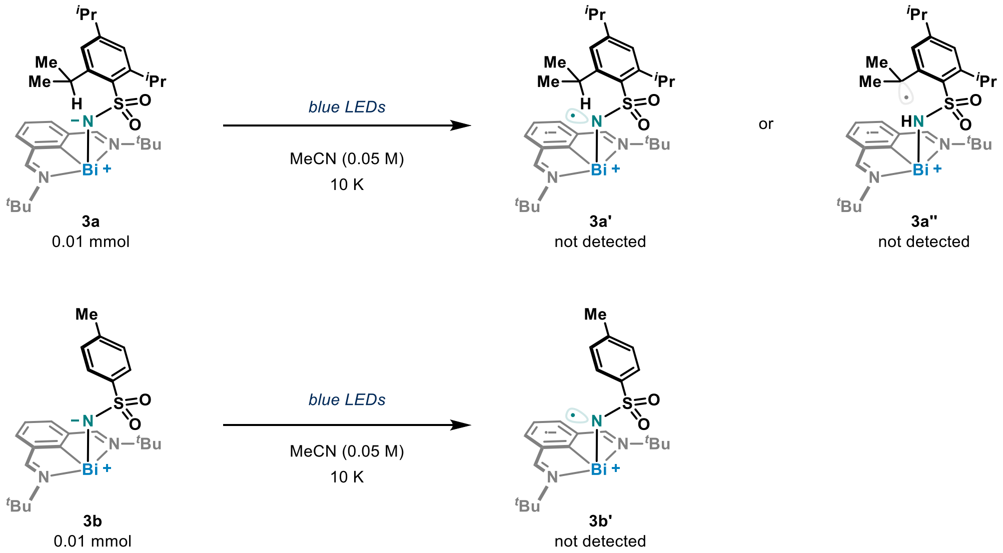

Figure S8. Attempts for direct detection of either nitrogen- or carbon-centered radical.

Additionally, we have tried to trap such short-lived radicals with spin trapping agents, such as 5,5-dimethyl-1-pyrroline-*N*-oxide (DMPO) and *N*-tert-butyl-α-phenylnitrone (PBN). However, the iminobismuthanes underwent a polar reaction, such as the one depicted below.

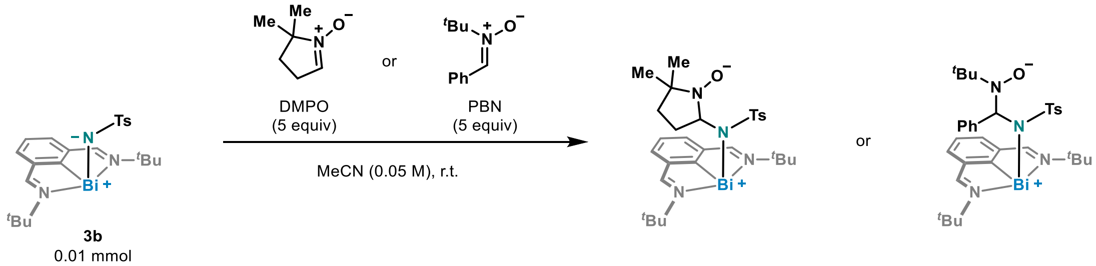

Figure S9. Attempts for spin trapping with DMPO and PBN

10. NMR Spectra

Compound **2c**: ^{1}H NMR (600 MHz, $CDCl_3$, 298 K)

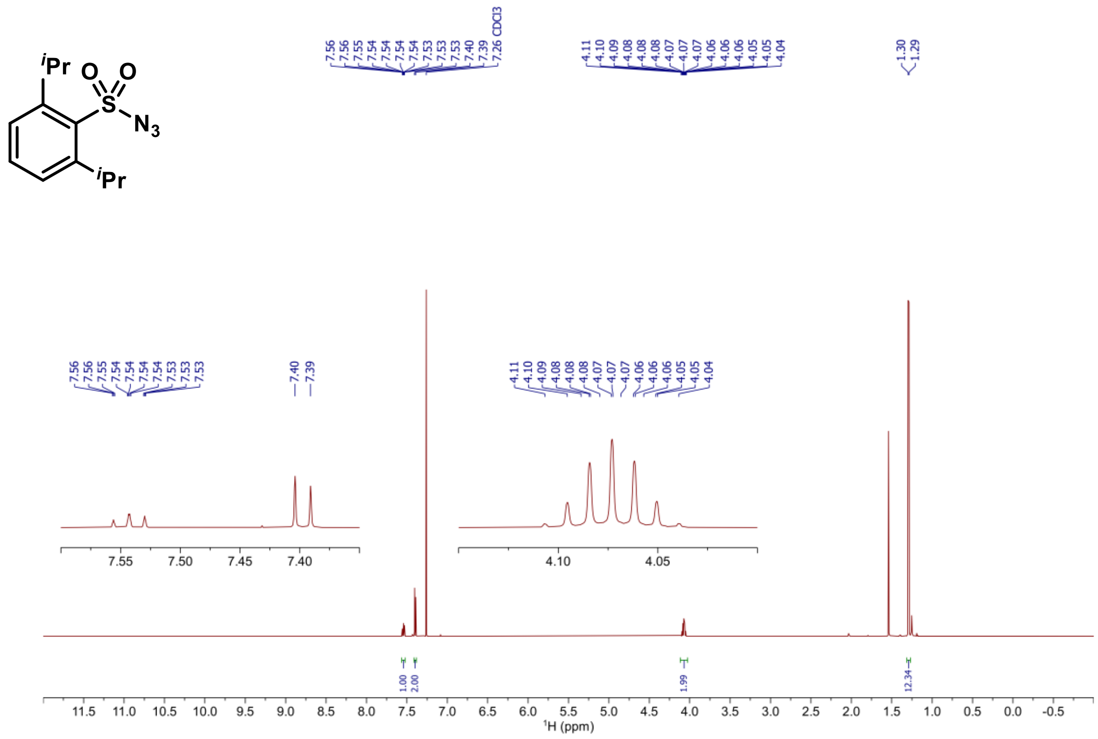

Compound **2c**: ^{13}C NMR (151 MHz, $CDCl_3$, 298 K)

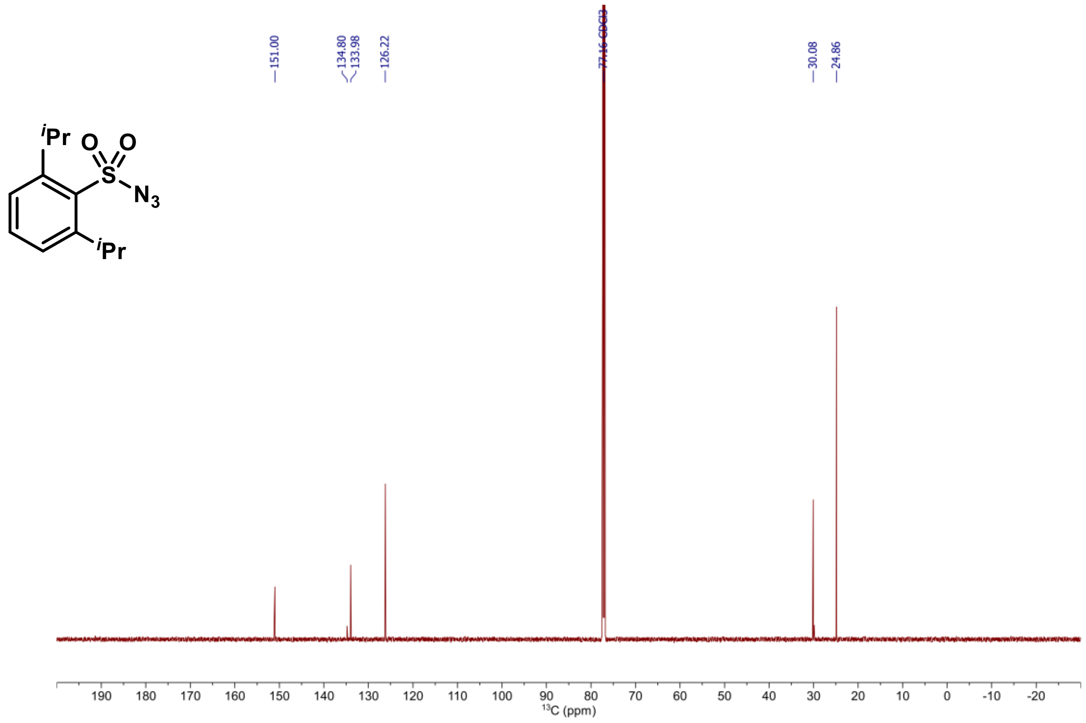

Compound **2c**: ^{1}H-^{13}C HSQC

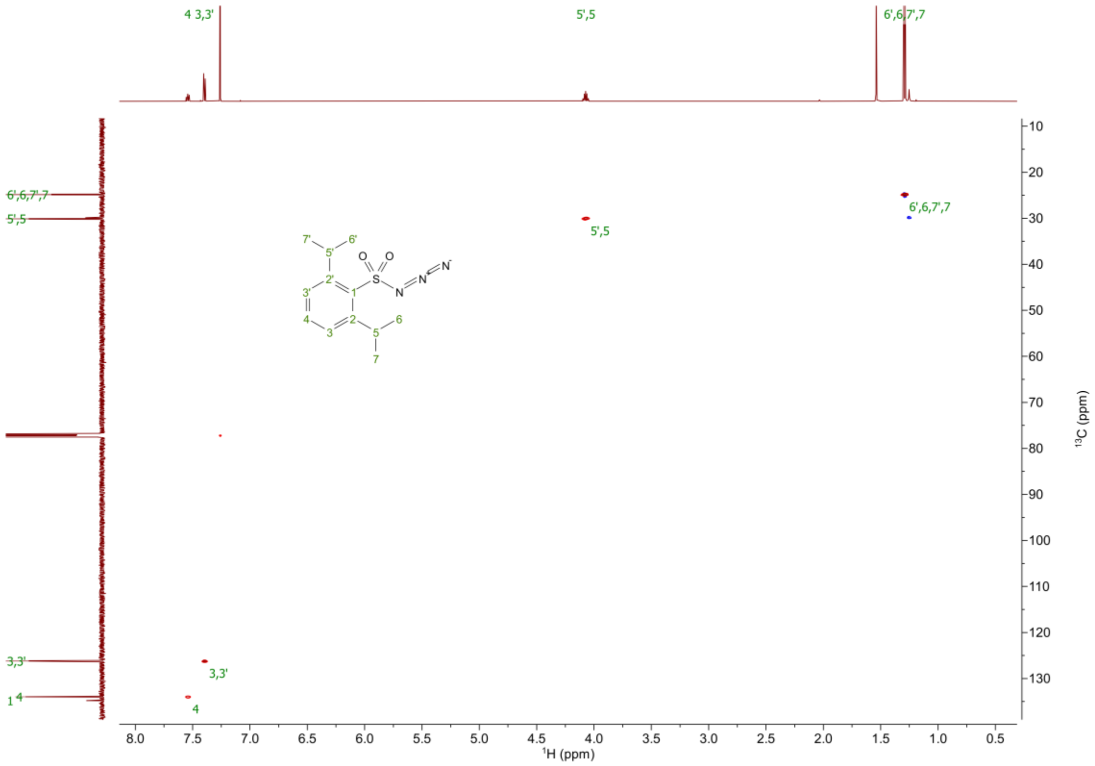

Compound **2c**: ^{1}H-^{13}C HMBC

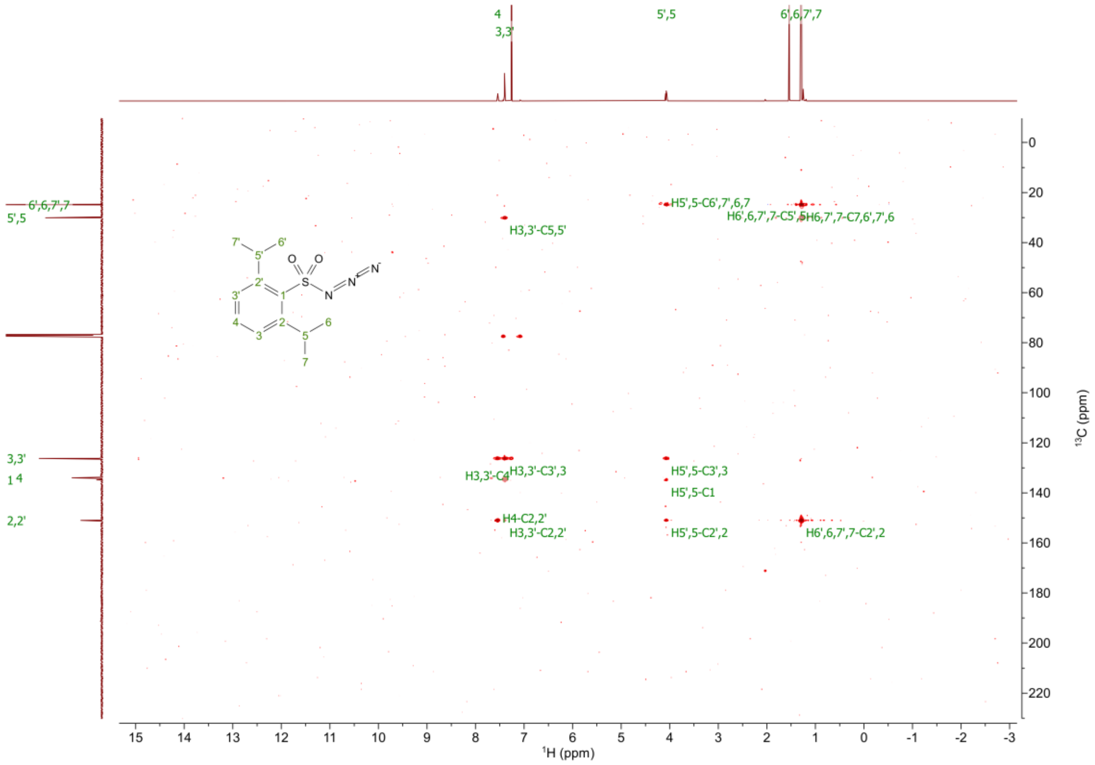

Compound **2c**: ^{1}H-^{1}H COSY

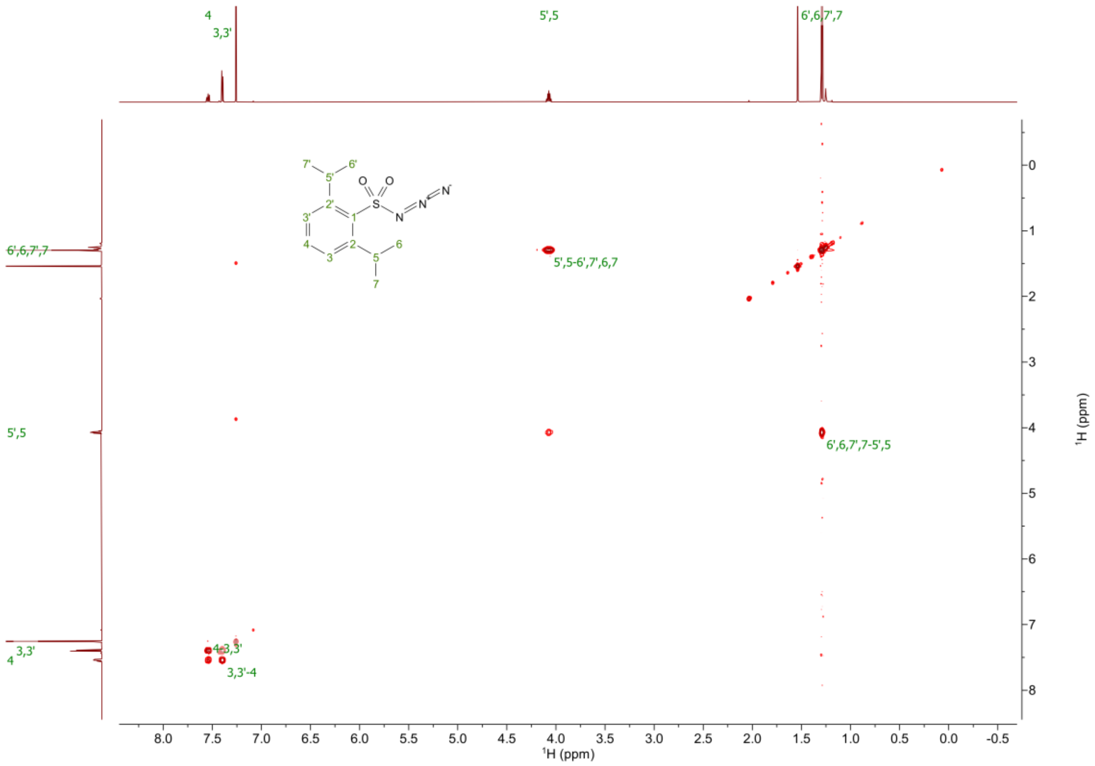

Compound **2f**: ^{1}H NMR (600 MHz, $CDCl_3$, 298 K)

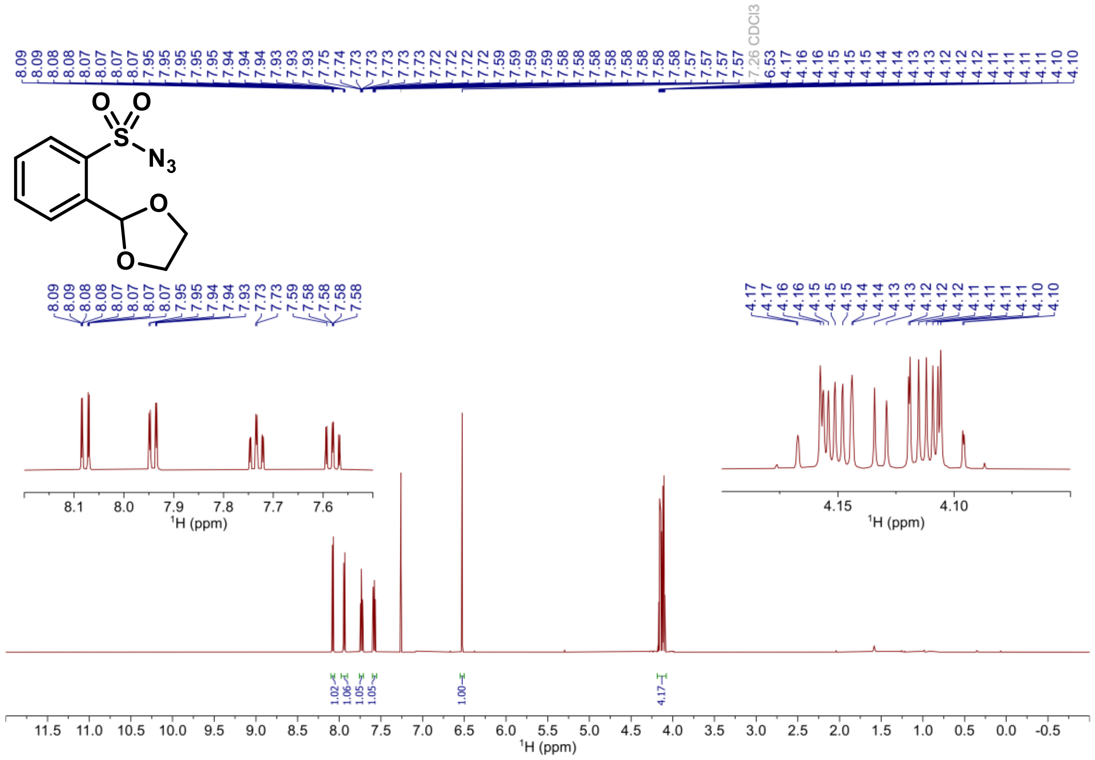

Compound **2f**: ^{13}C NMR (151 MHz, $CDCl_3$, 298 K)

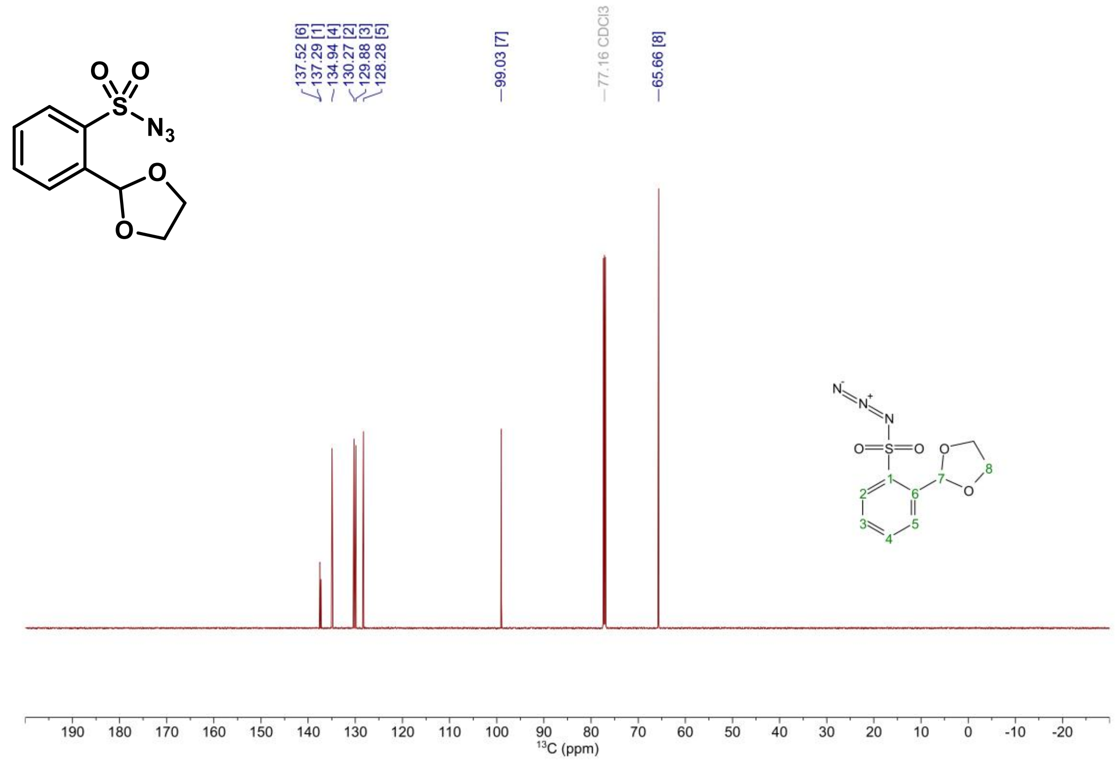

Compound **2f**: ^{1}H-^{13}C HSQC

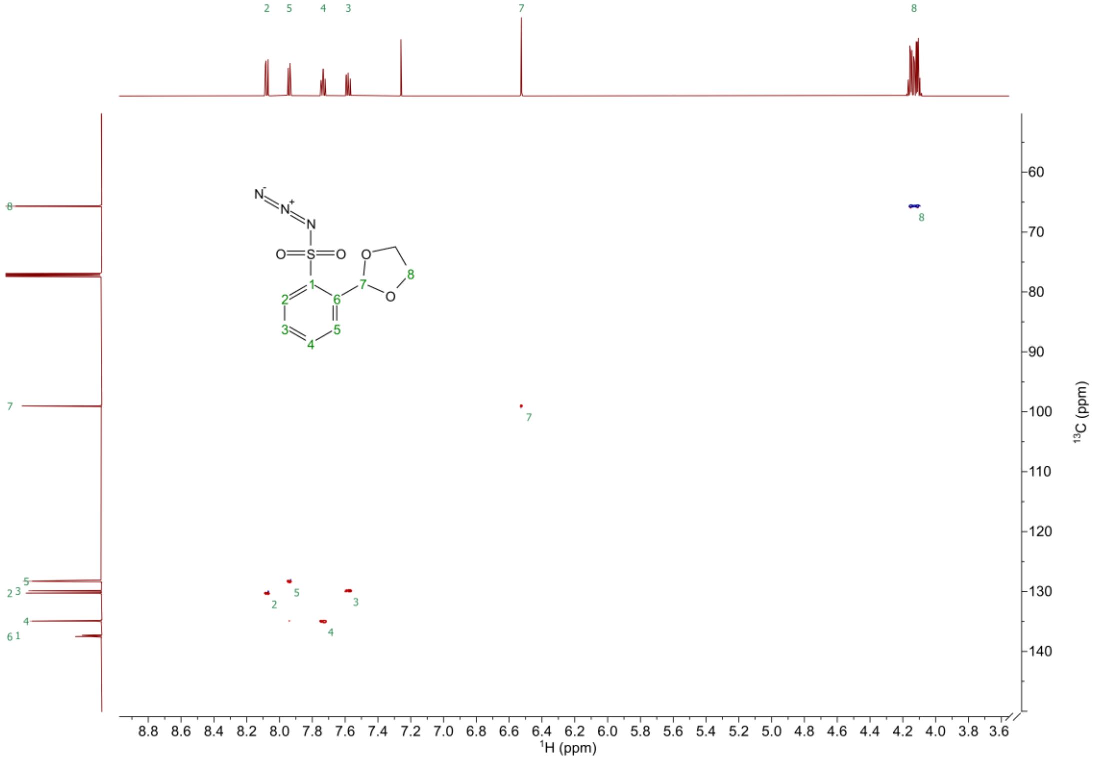

Compound **2f**: ^{1}H-^{13}C HMBC

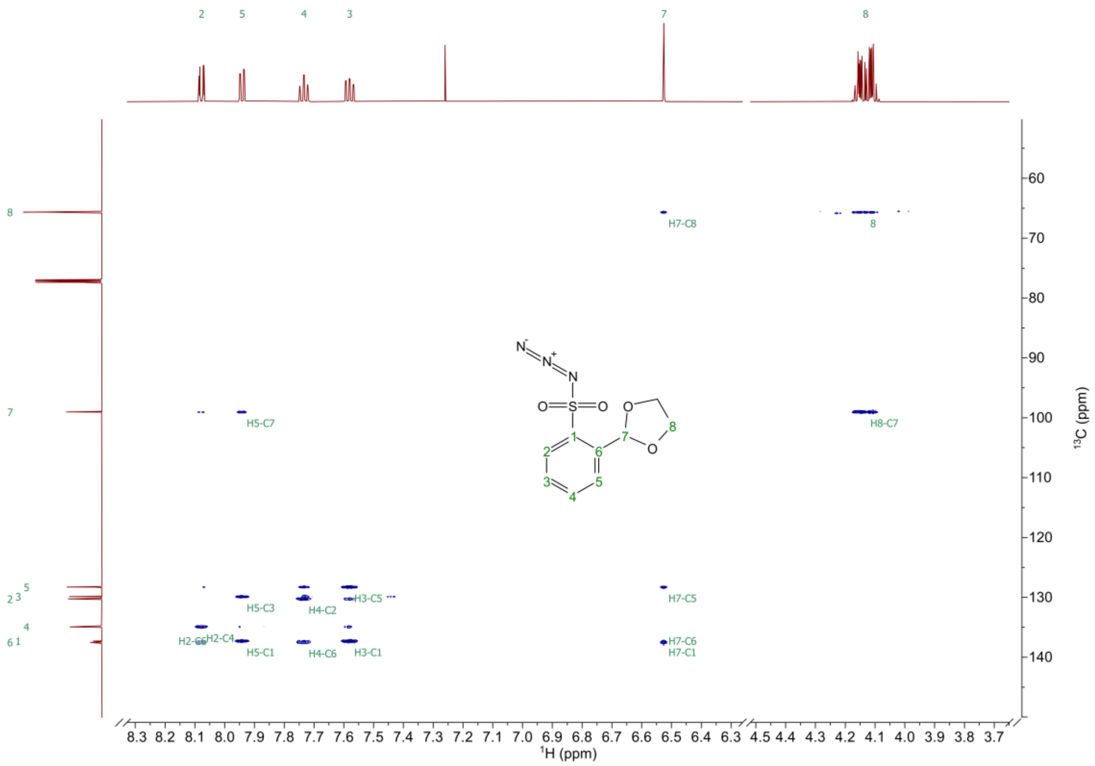

Compound **2f**: ^{1}H-^{1}H COSY

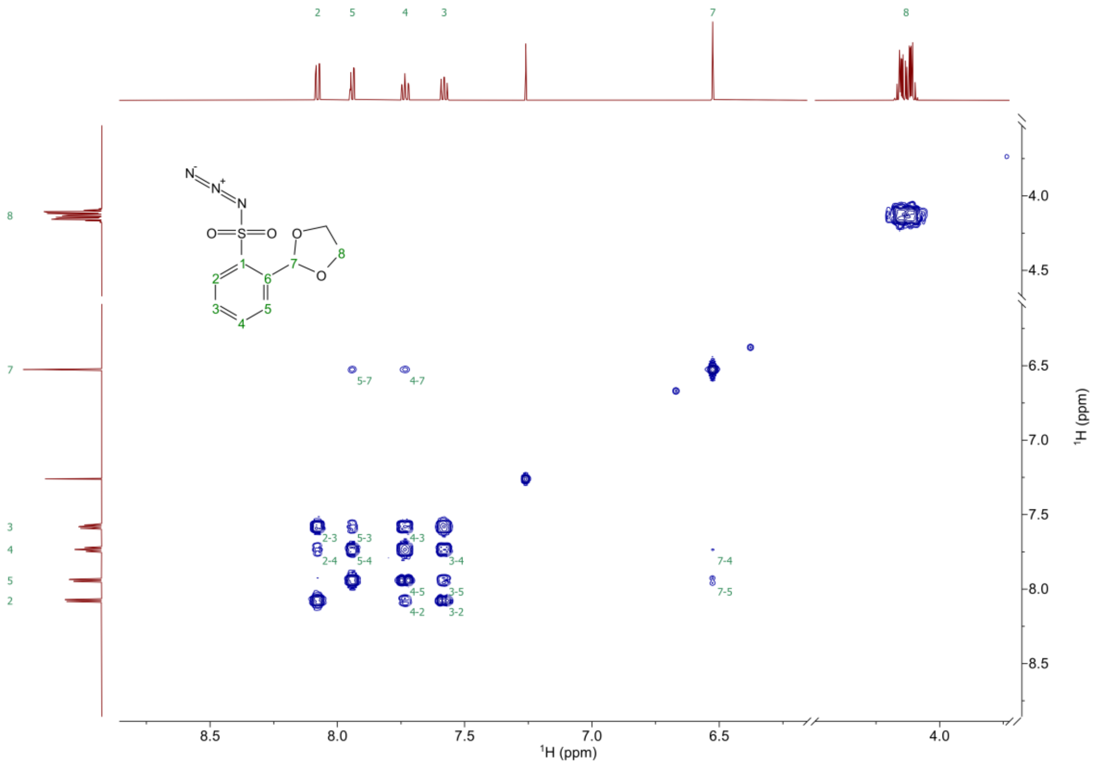

Compound **2f**: ^{1}H-^{1}H NOESY

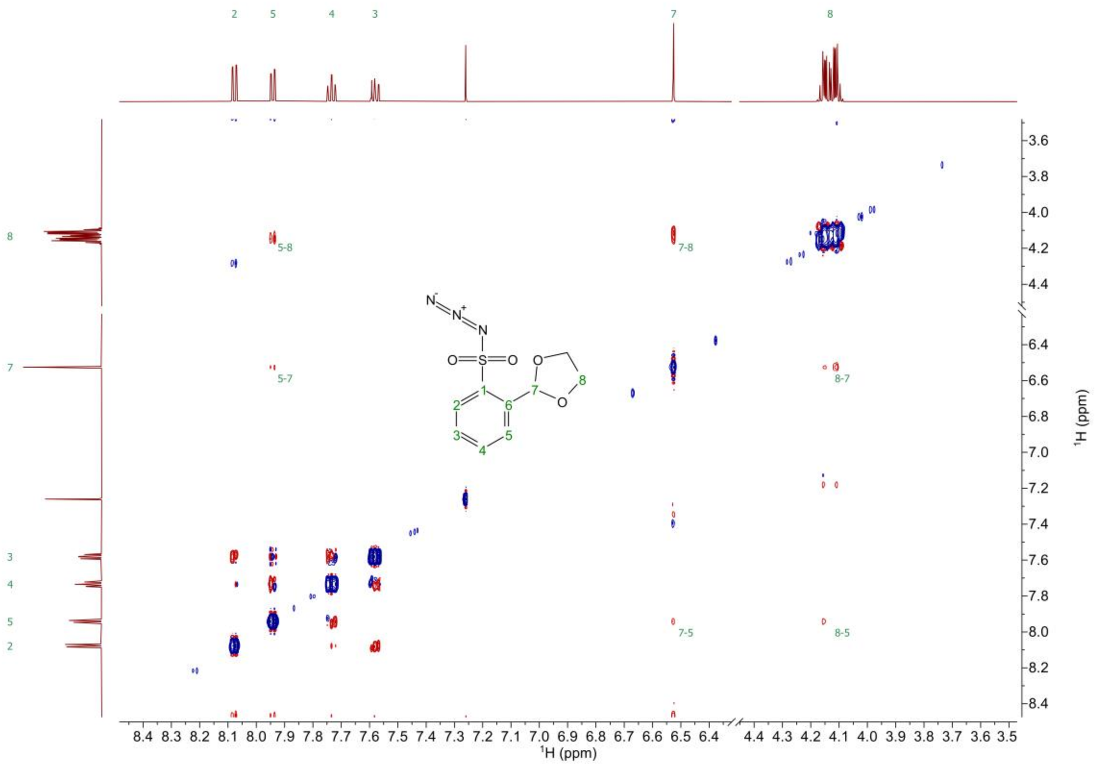

Compound **6n**: ^{1}H NMR (600 MHz, $CDCl_3$, 298 K)

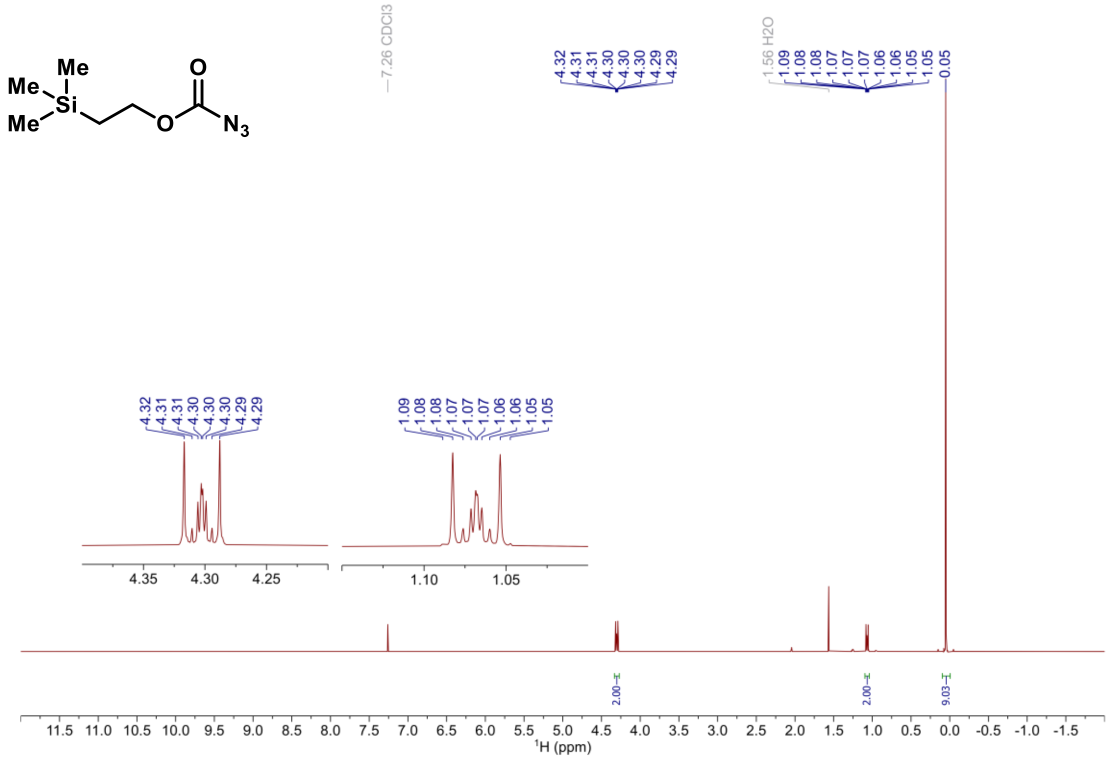

Compound **6n**: ^{13}C NMR (151 MHz, $CDCl_3$, 298 K)

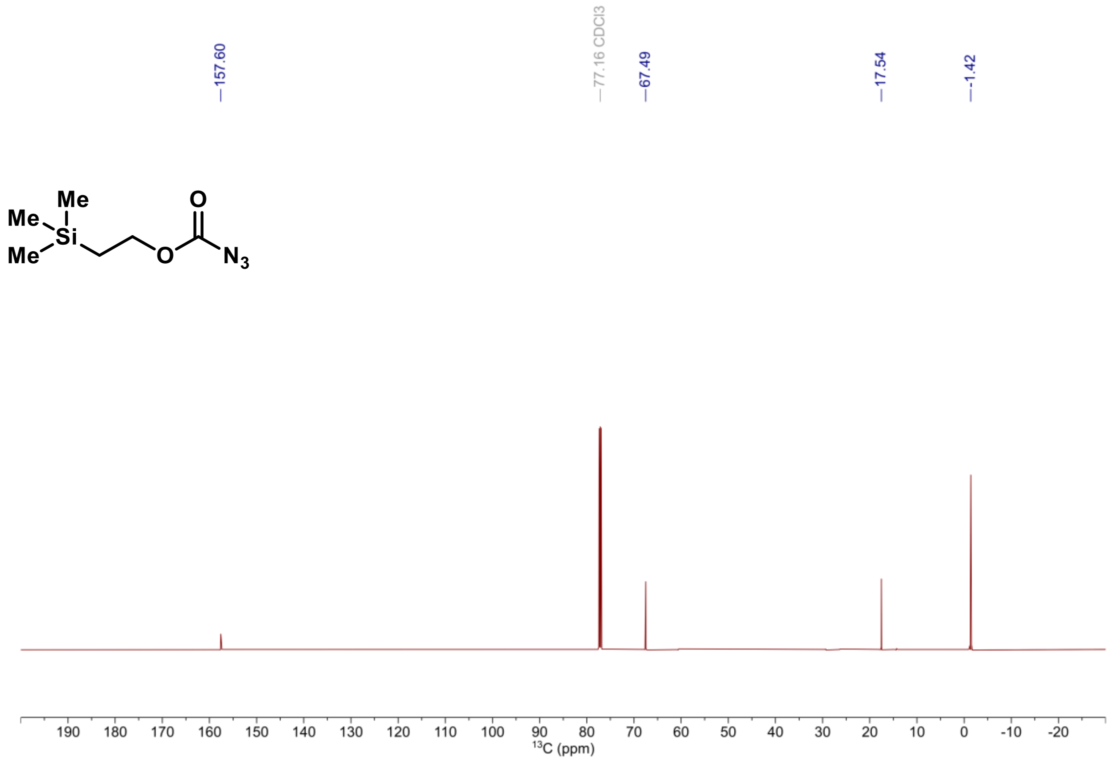

Compound **6n**: ^{1}H-^{13}C HSQC

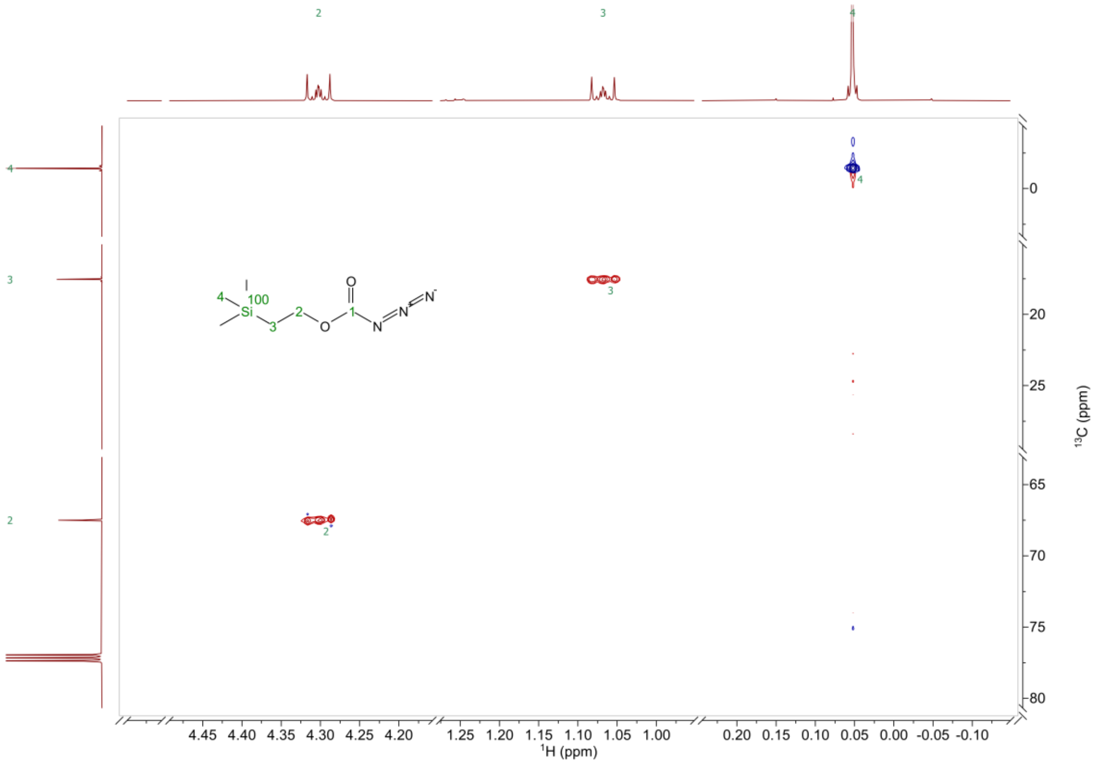

Compound **6n**: ^{1}H-^{13}C HMBC

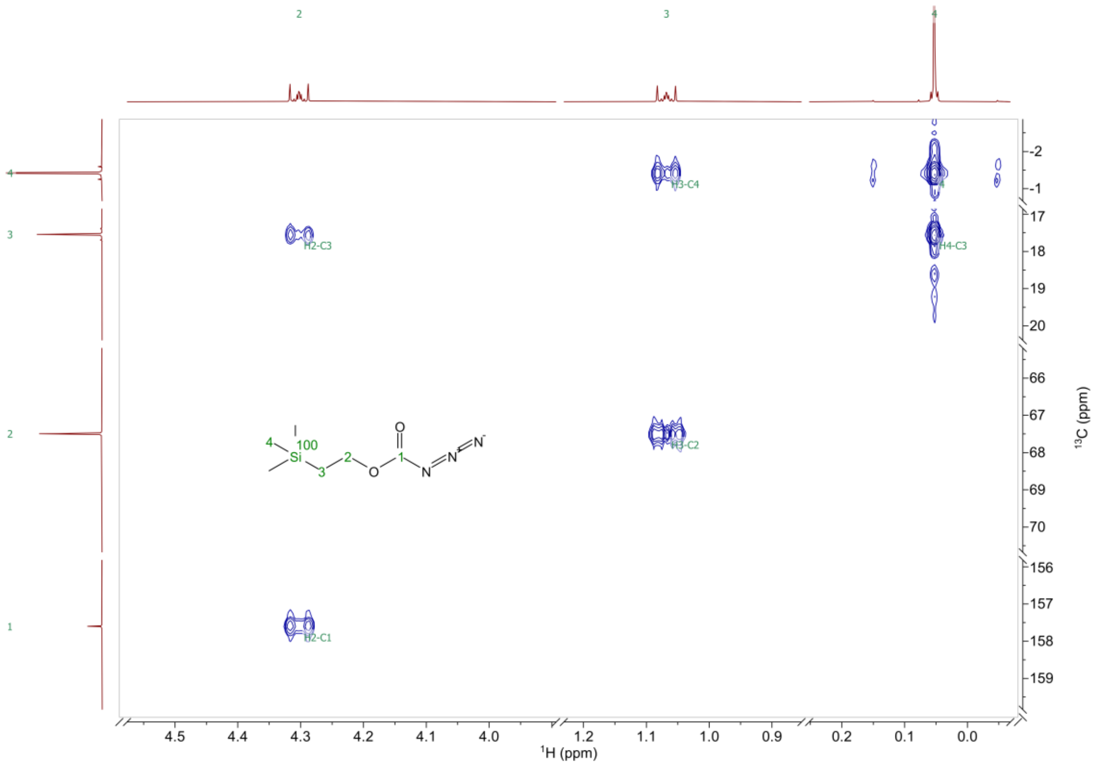

Compound **6n**: ^{1}H-^{1}H COSY

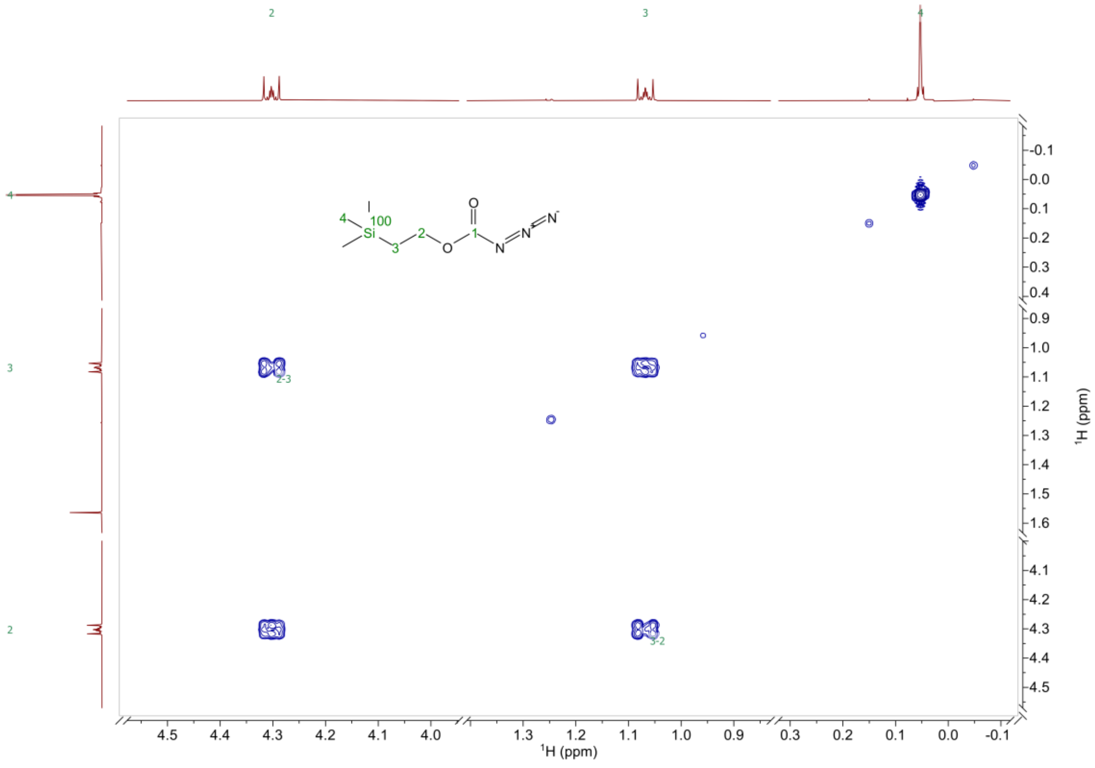

Compound **6n**: $^{29}Si\{^{1}H\}$ NMR (119 MHz, $CDCl_3$)

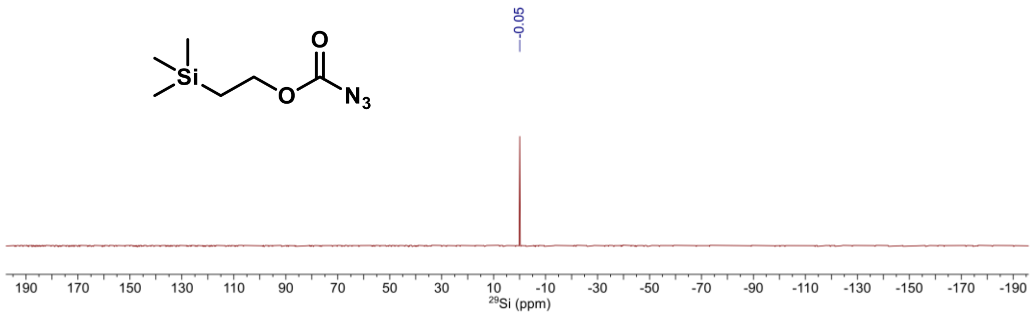

Compound **6n**: ^{1}H-^{29}Si HMBC

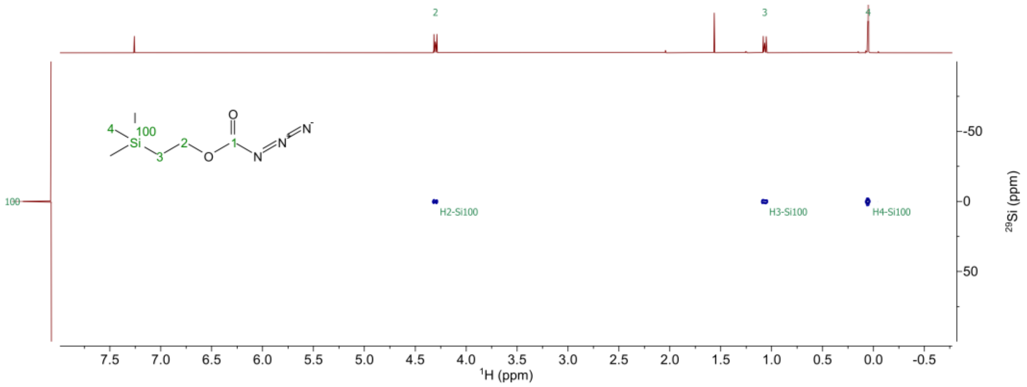

Compound **6o**: ^{1}H NMR (600 MHz, $CDCl_3$, 298 K)

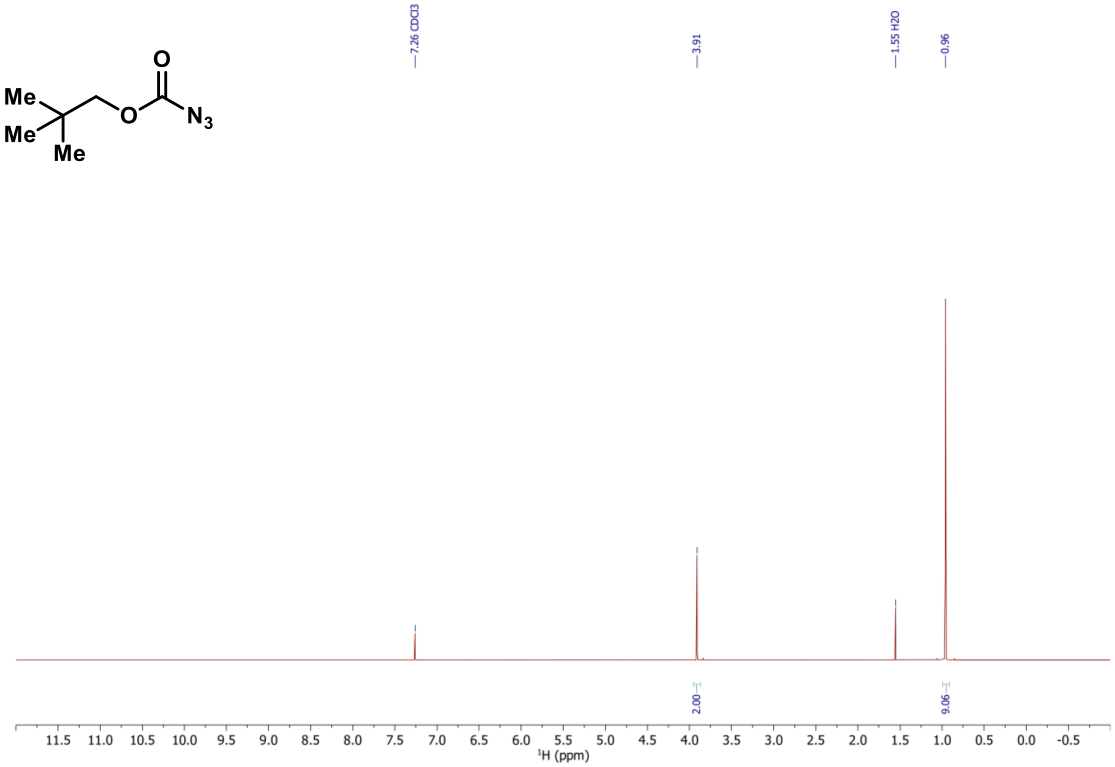

Compound **6o**: ^{13}C NMR (151 MHz, $CDCl_3$, 298 K)

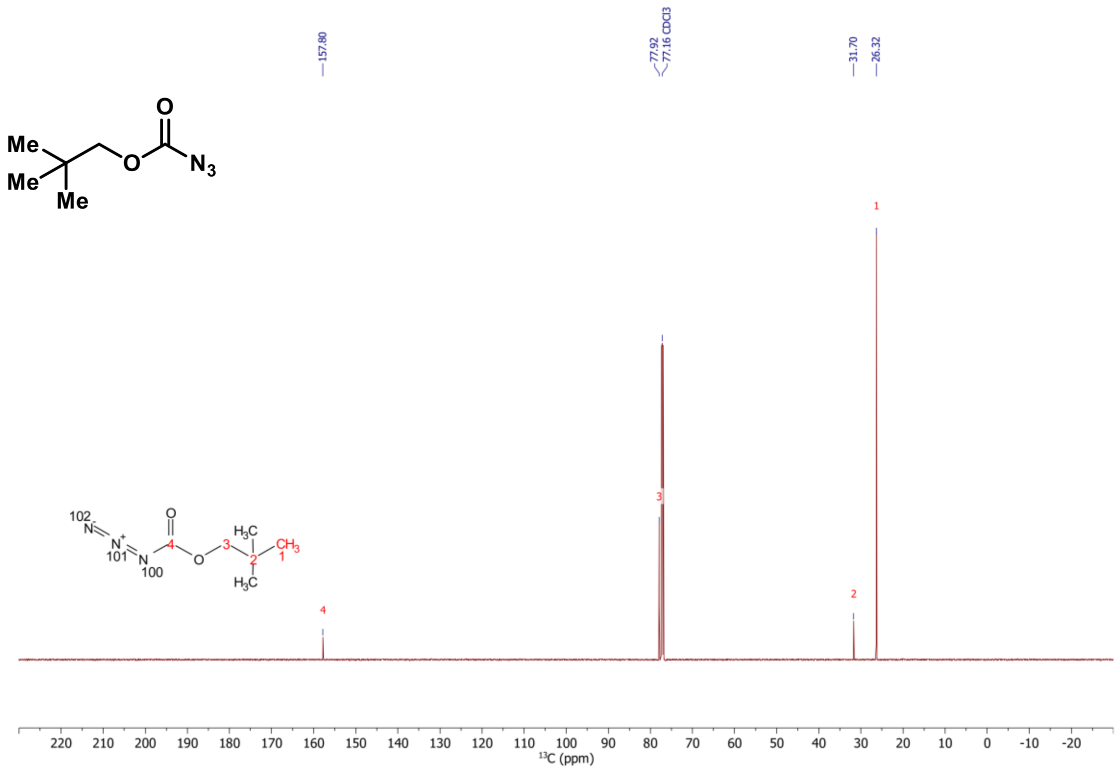

Compound **6o**: ^{1}H-^{13}C HSQC

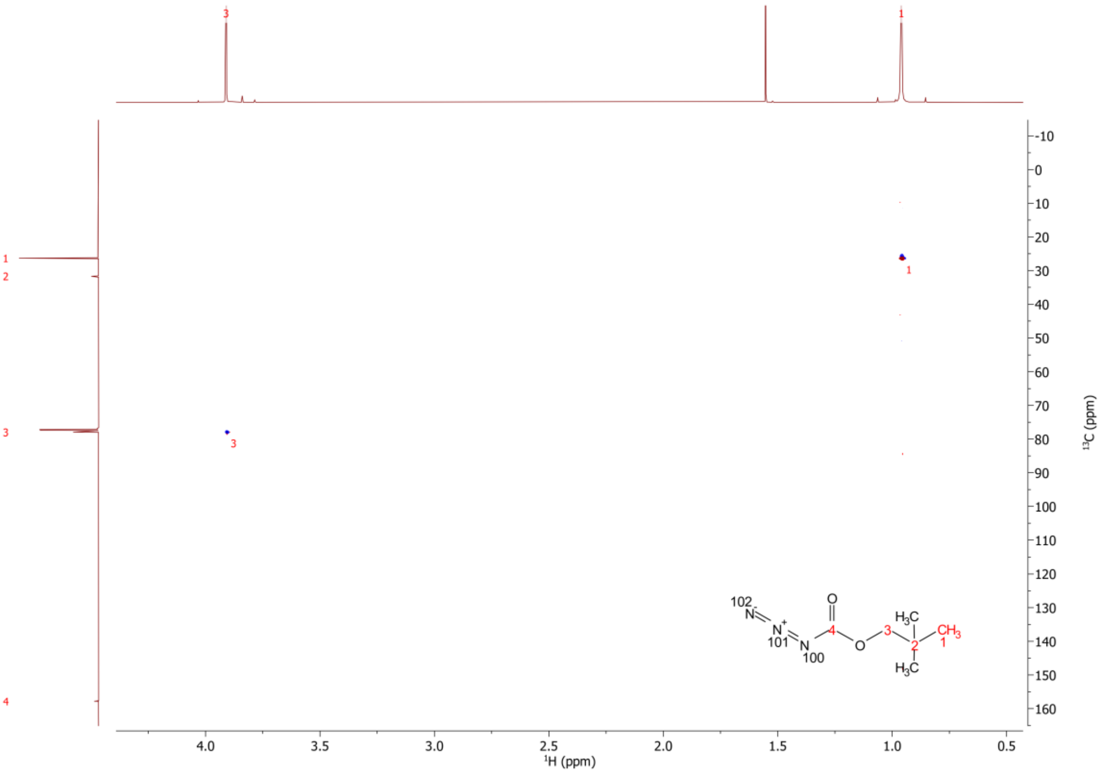

Compound **6o**: ^{1}H-^{13}C HMBC

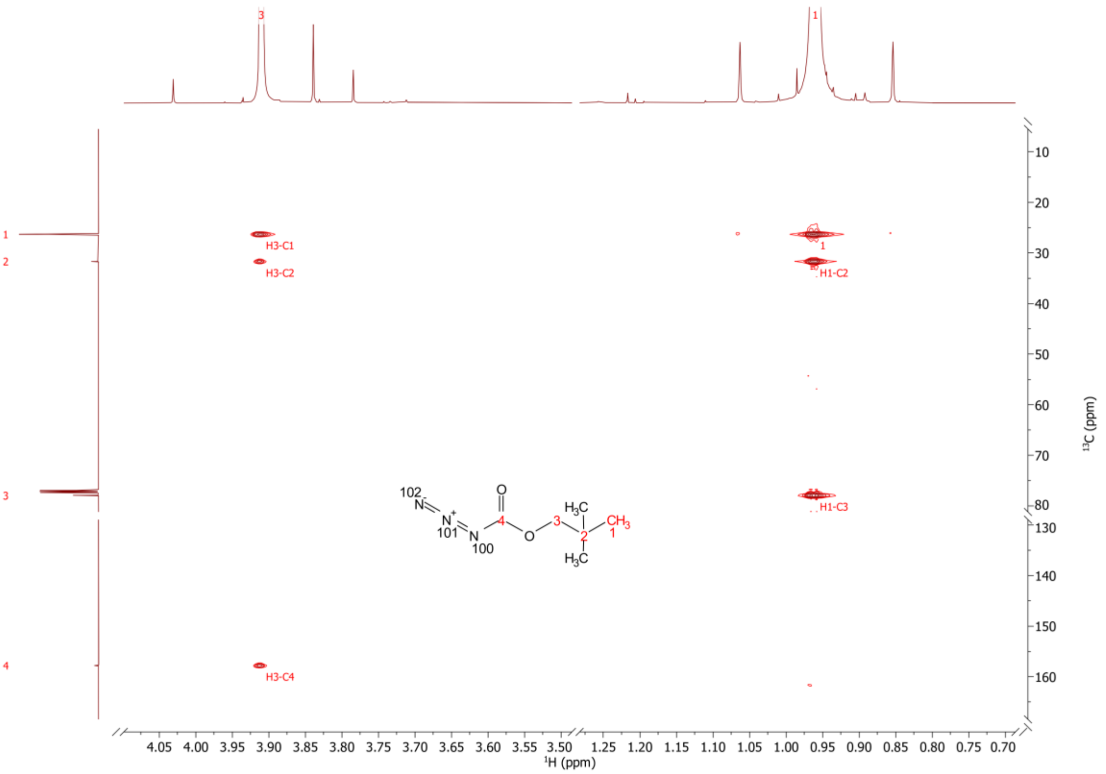

Compound **6p**: ^{1}H NMR (600 MHz, $CDCl_3$, 298 K)

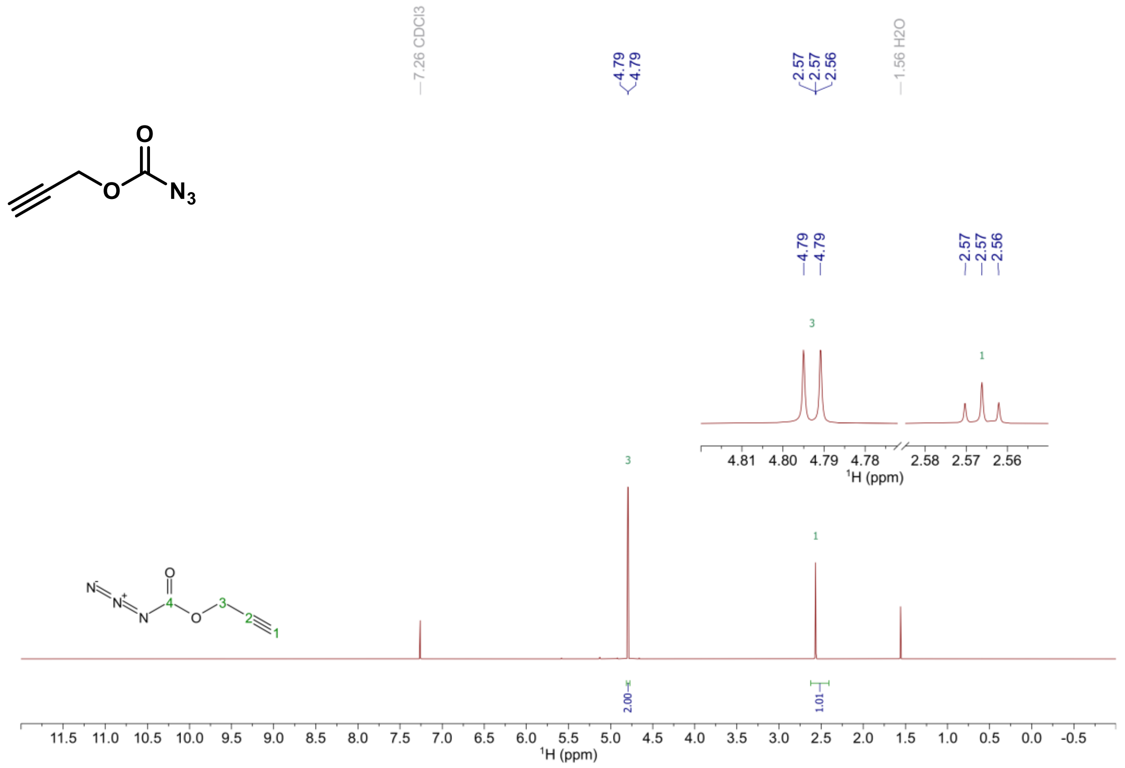

Compound **6p**: ^{13}C NMR (151 MHz, $CDCl_3$, 298 K)

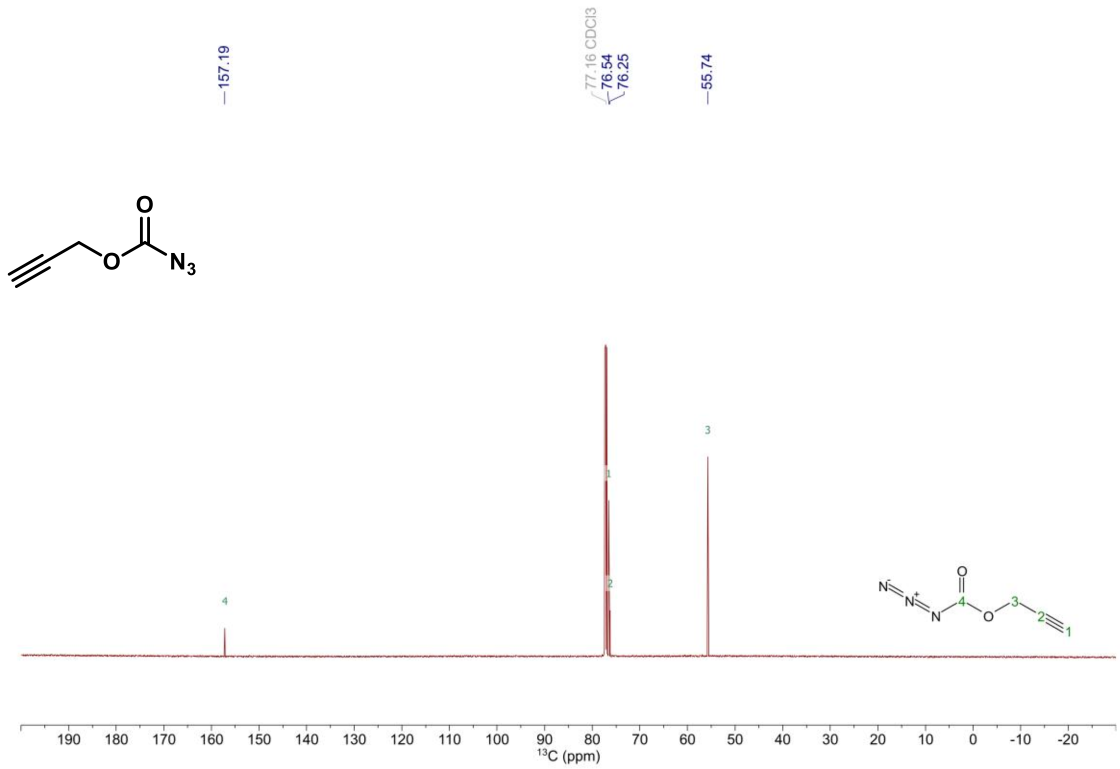

Compound **6p**: ^{1}H-^{13}C HSQC

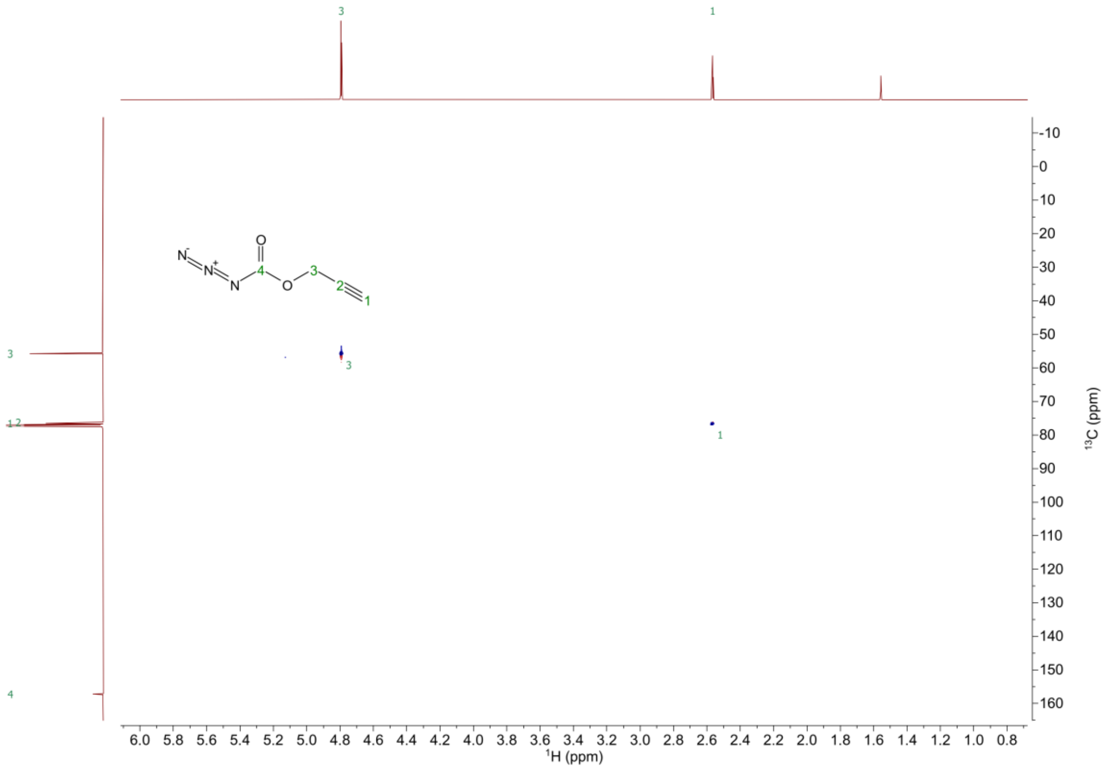

Compound **6p**: ^{1}H-^{13}C HMBC

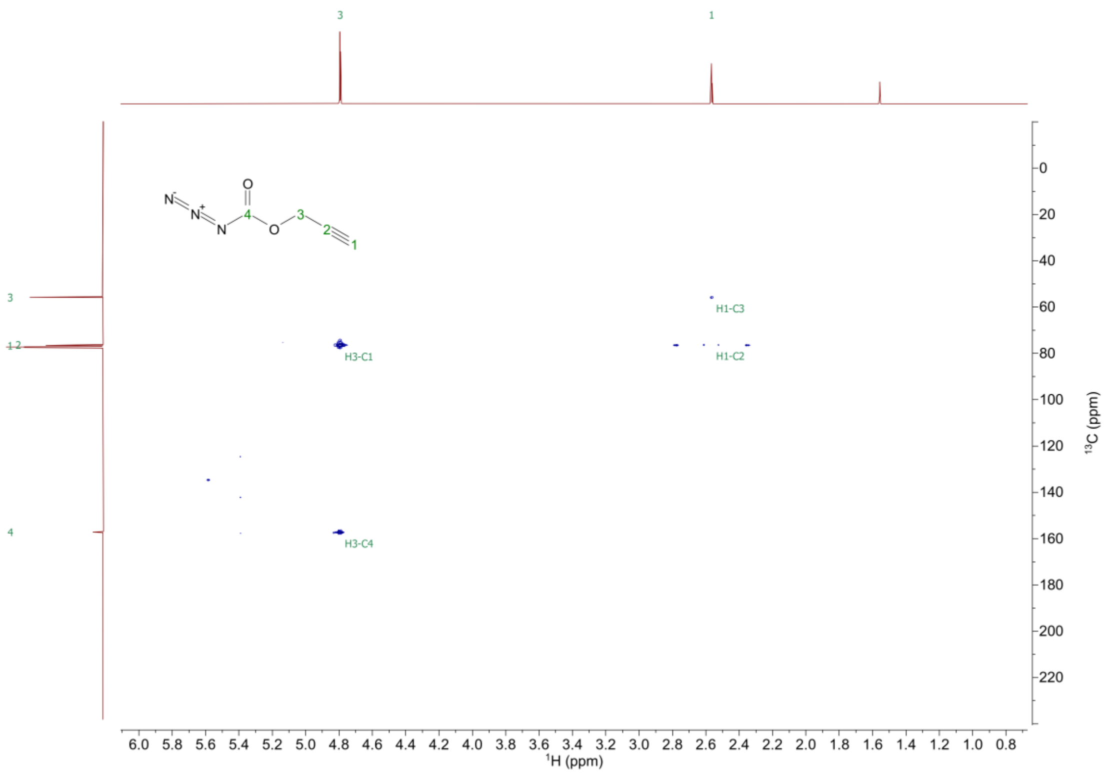

Compound **6p**: ^{1}H-^{1}H COSY

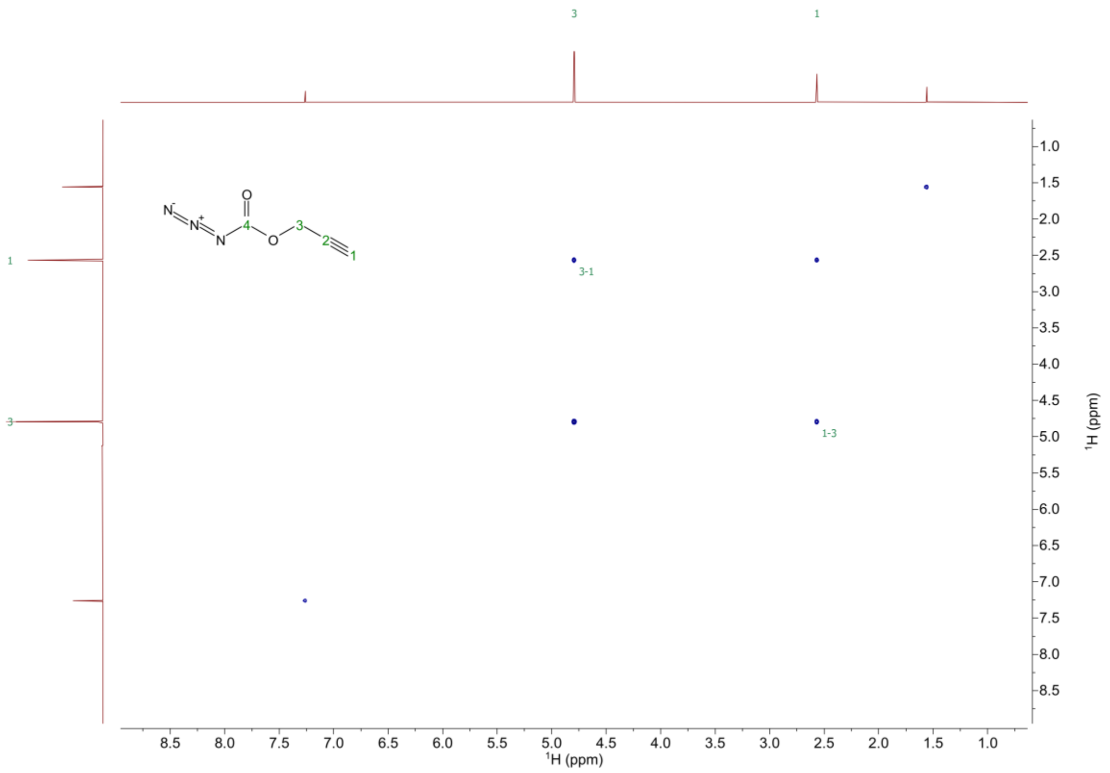

Compound **6q**: ^{1}H NMR (600 MHz, $CDCl_3$)

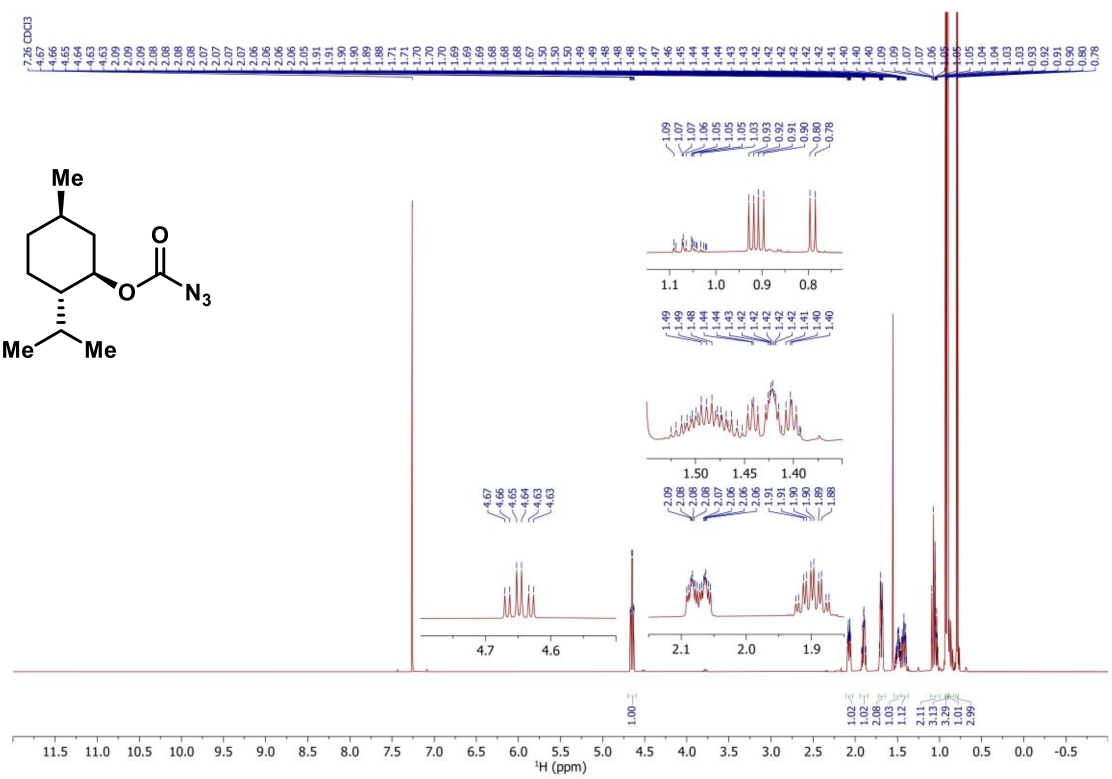

Compound **6q**: ^{13}C NMR (151 MHz, $CDCl_3$)

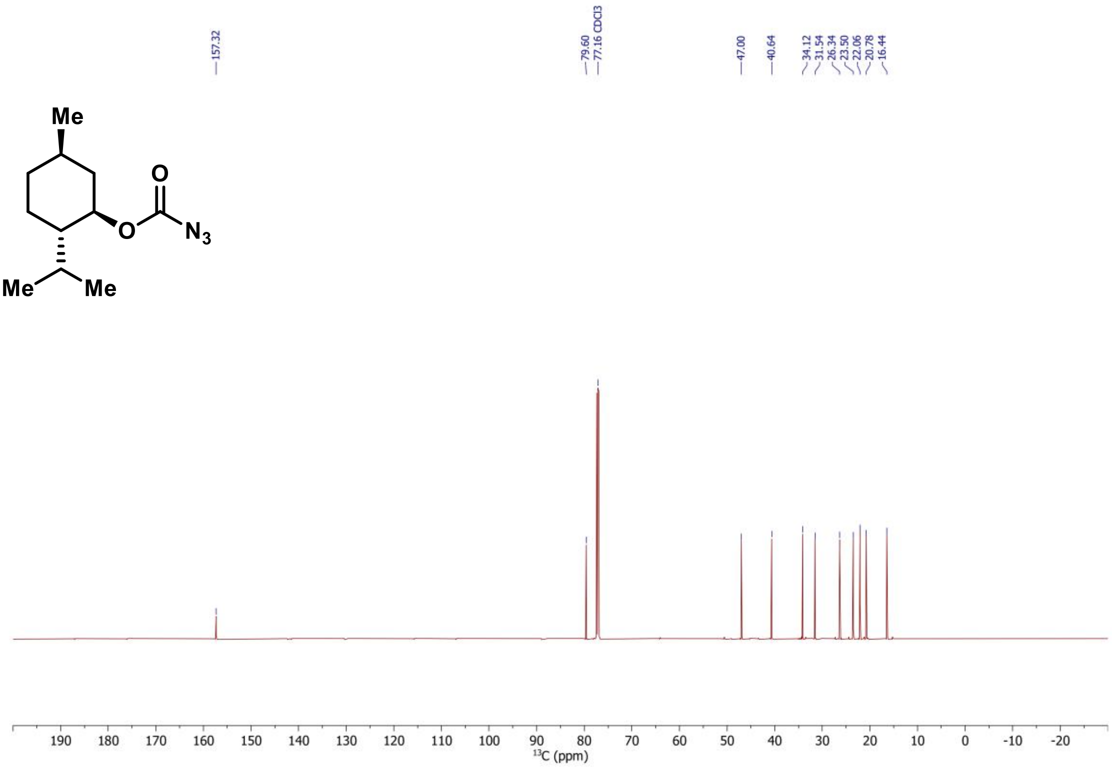

Compound **6q**: ^{1}H-^{13}C HSQC

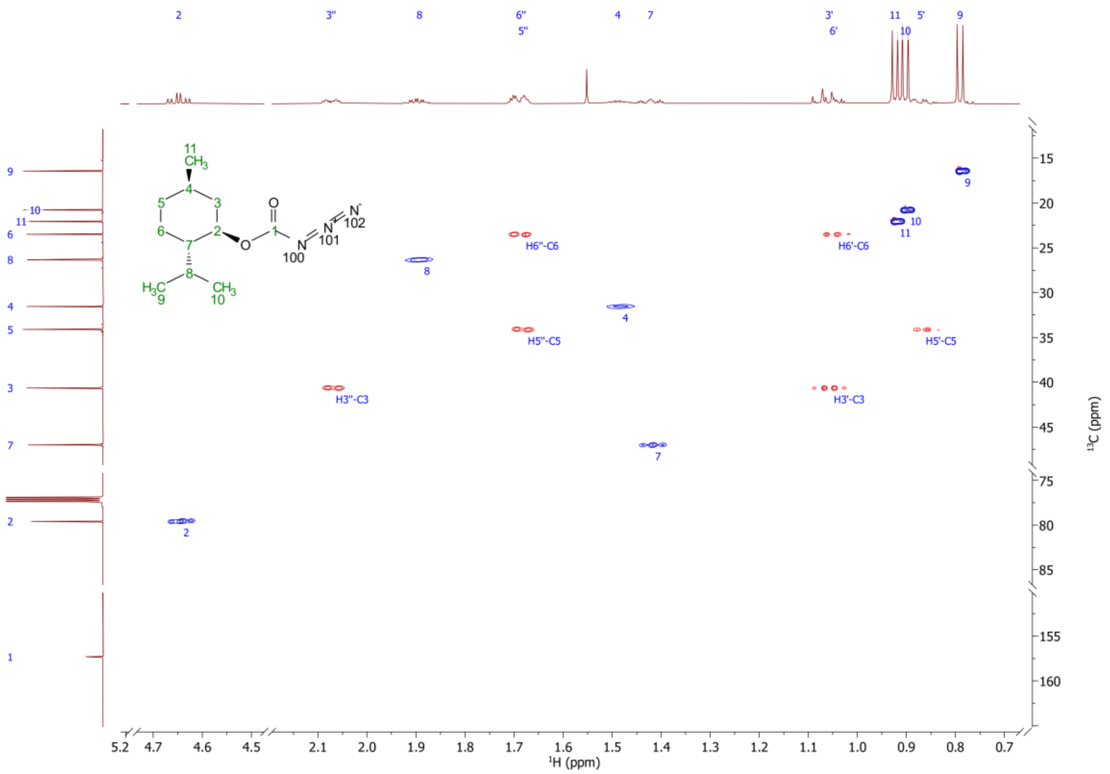

Compound **6q**: ^{1}H-^{13}C HMBC

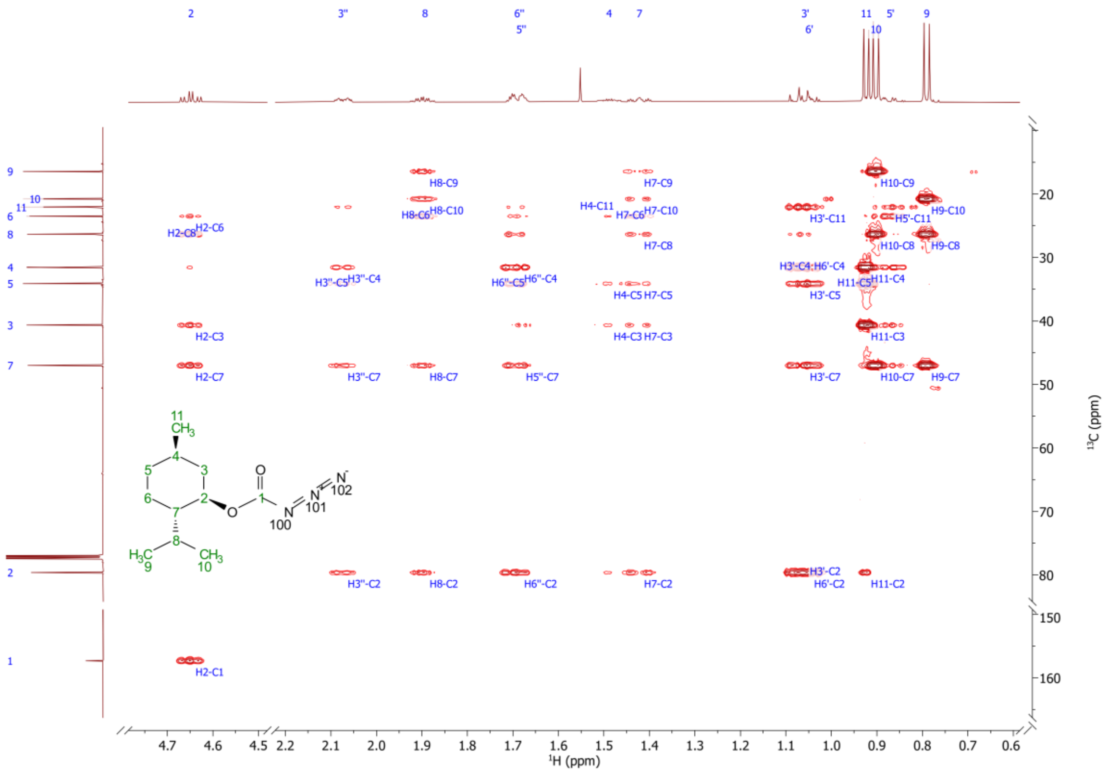

Compound **6q**: ^{1}H-^{1}H COSY

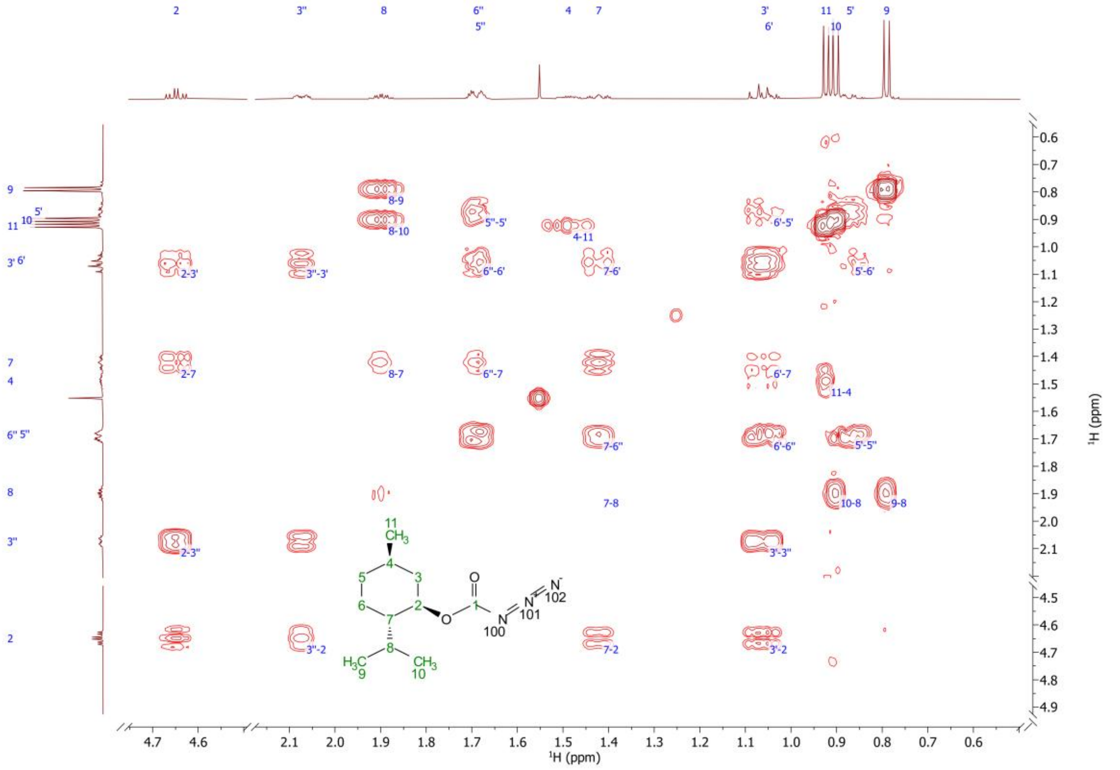

Compound **6q**: ^{1}H-^{1}H NOESY

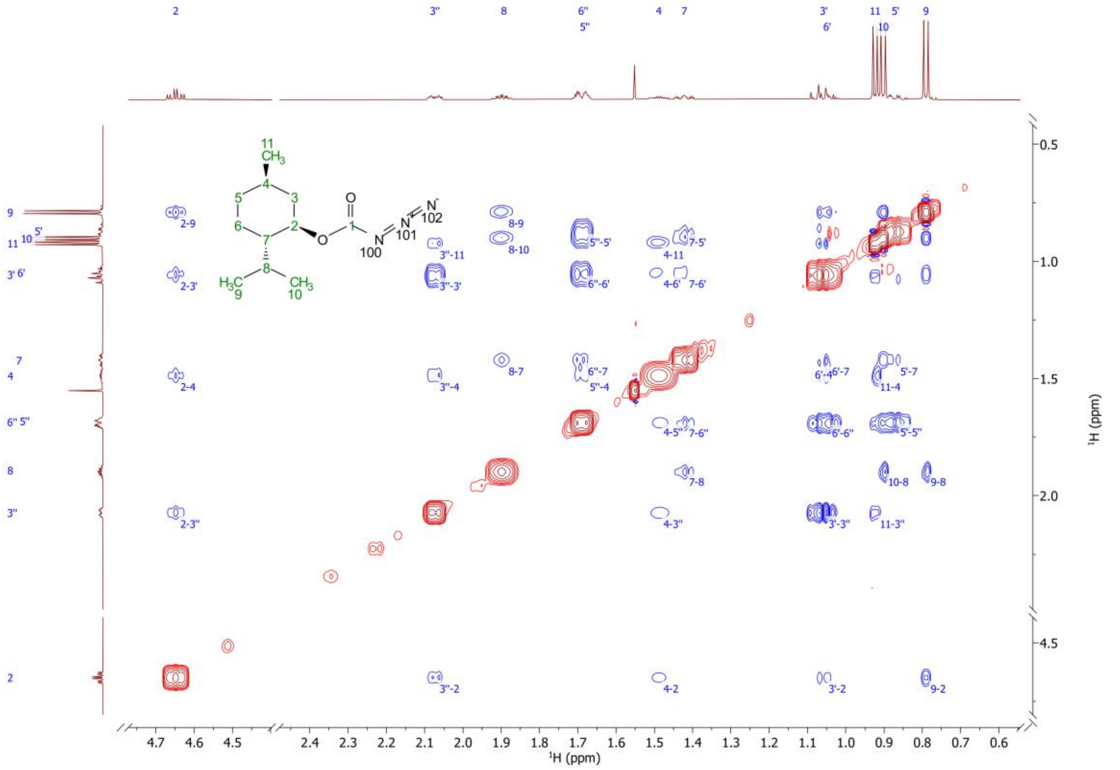

Compound **6r**: ^{1}H NMR (600 MHz, $CDCl_3$, 298 K)

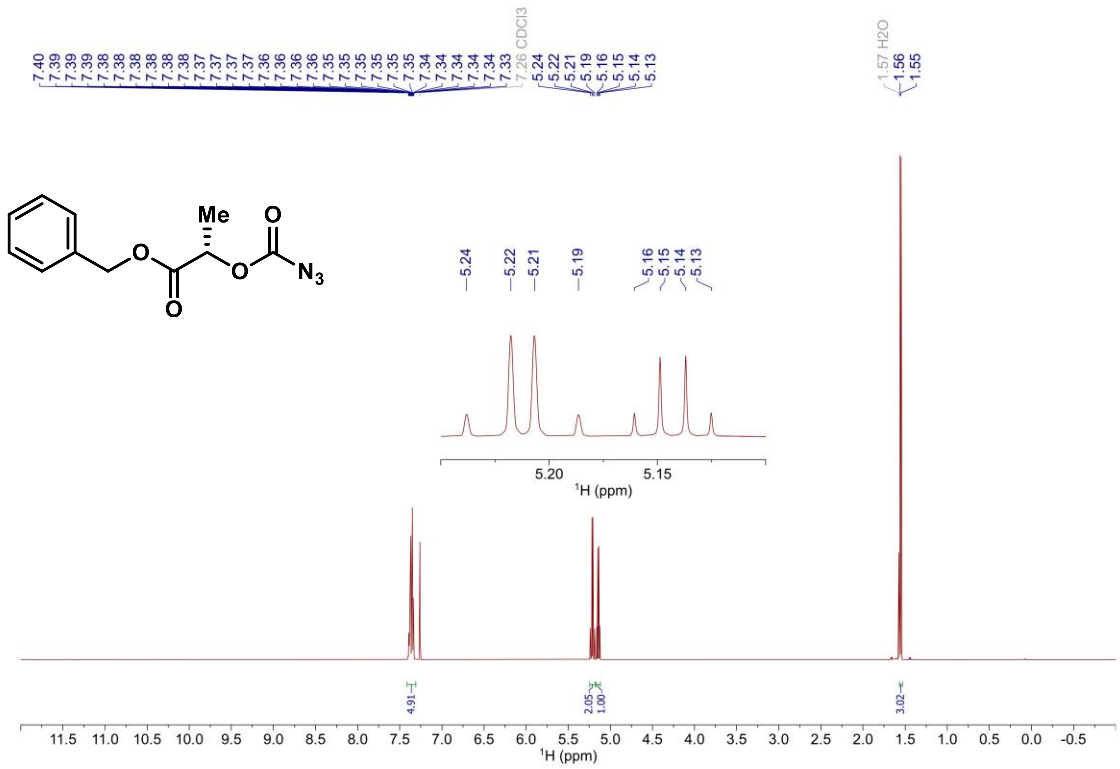

Compound **6r**: ^{13}C NMR (151 MHz, $CDCl_3$, 298 K)

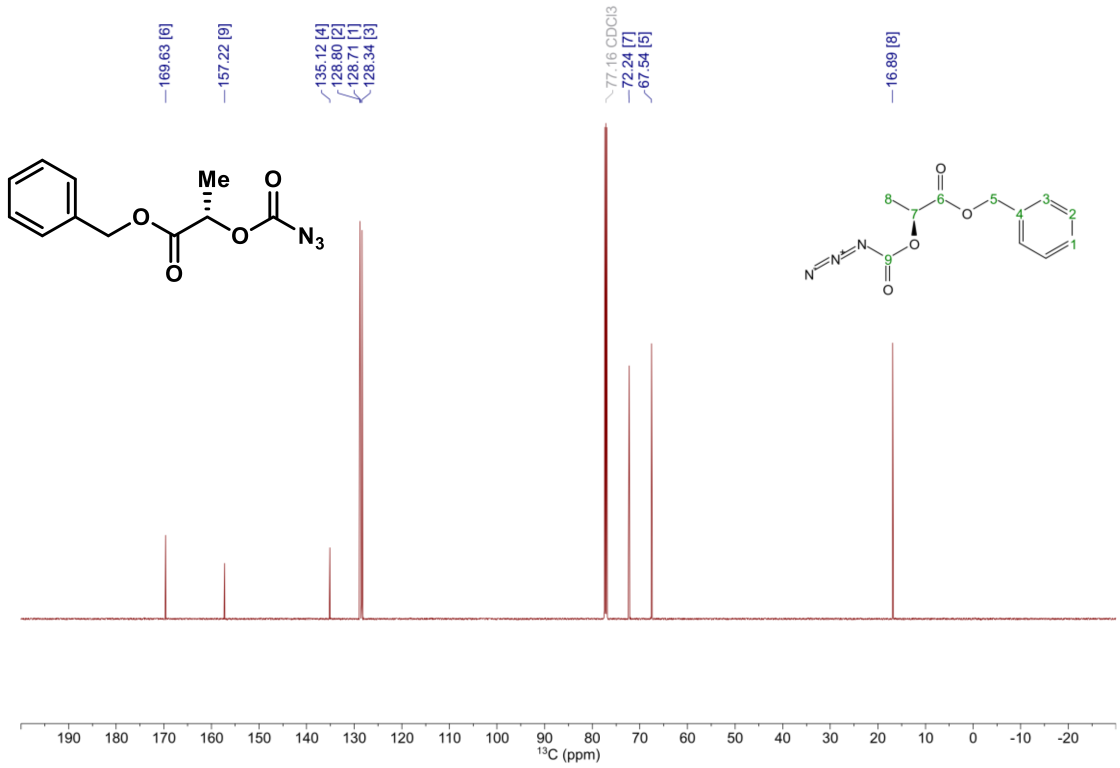

Compound **6r**: ^{1}H-^{13}C HSQC

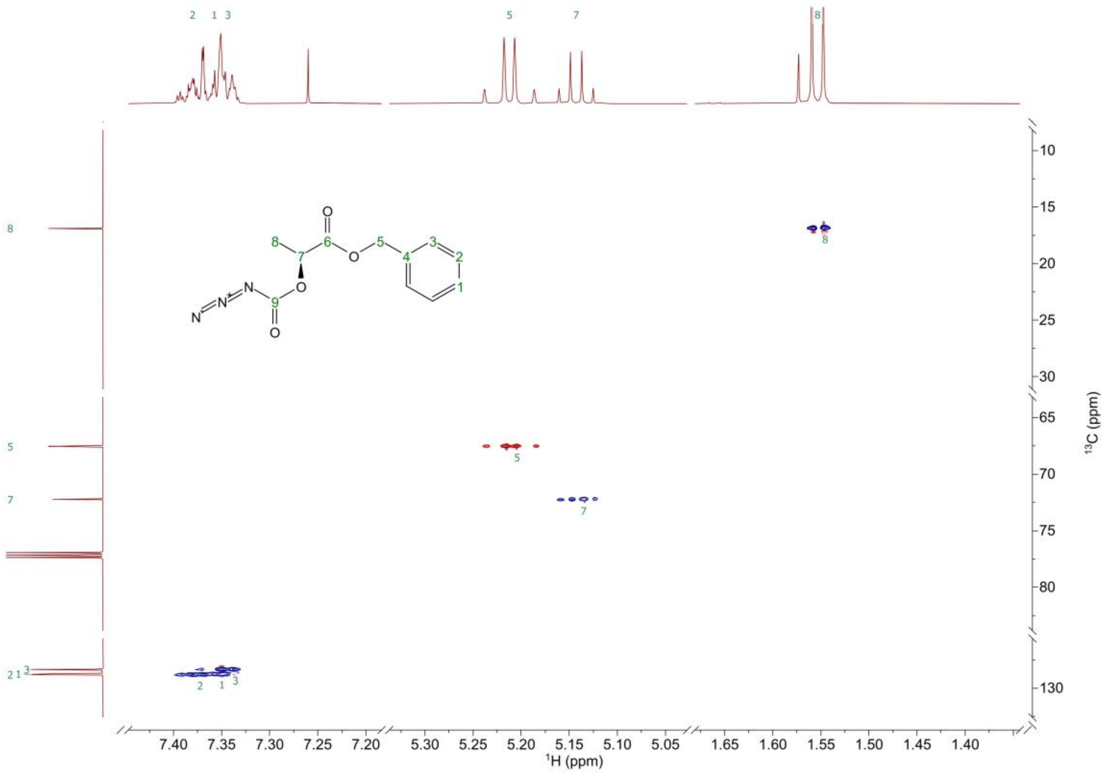

Compound **6r**: ^{1}H-^{13}C HMBC

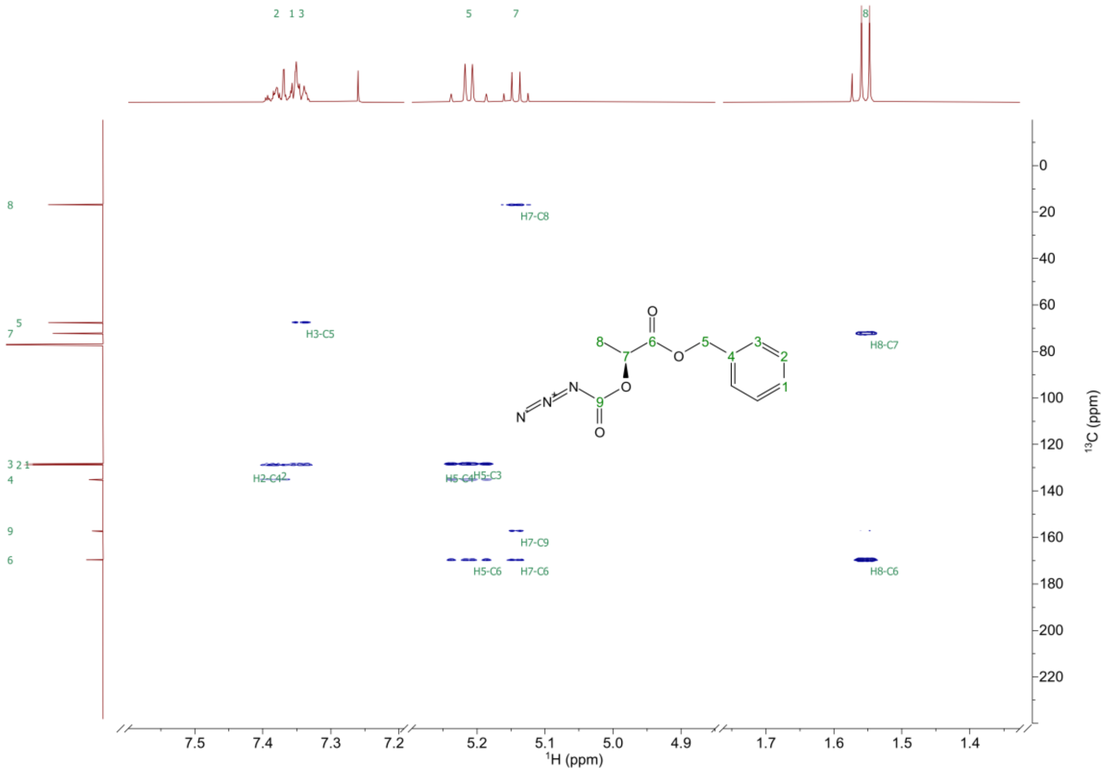

Compound **6r**: ^{1}H-^{1}H COSY

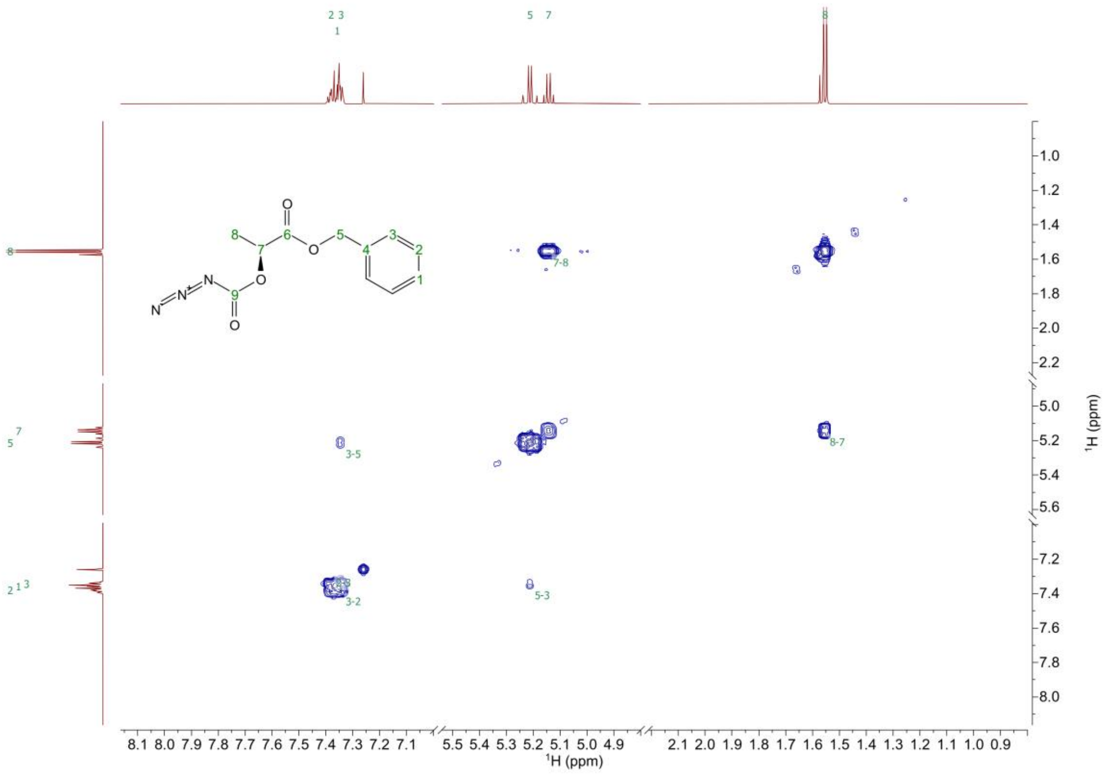

Compound **6r**: ^{1}H-^{1}H NOESY

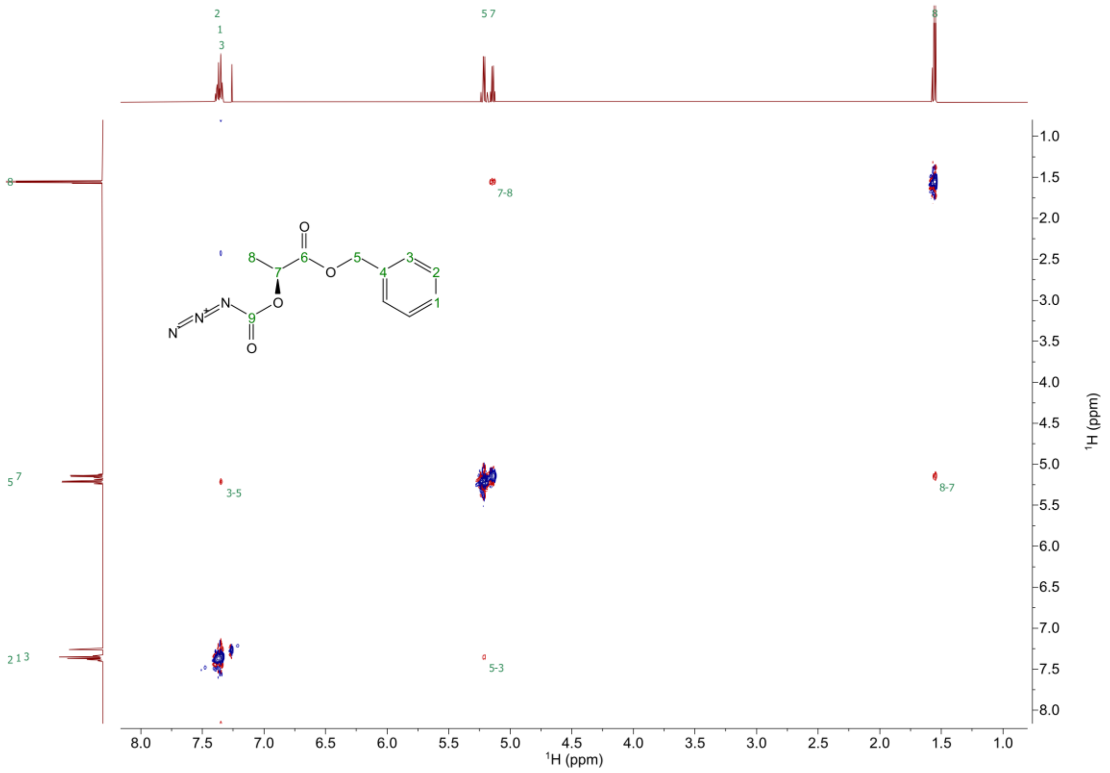

Compound **6s**: ^{1}H NMR (600 MHz, $CDCl_3$, 298 K)

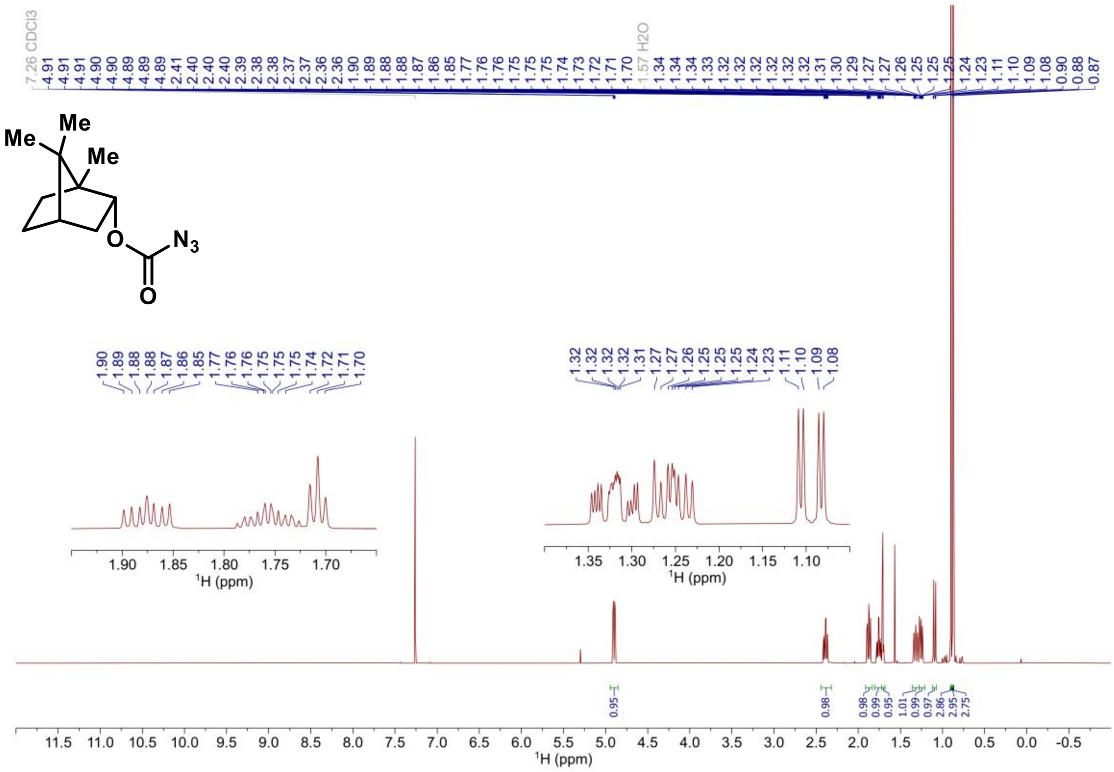

Compound **6s**: ^{13}C NMR (151 MHz, $CDCl_3$, 298 K)

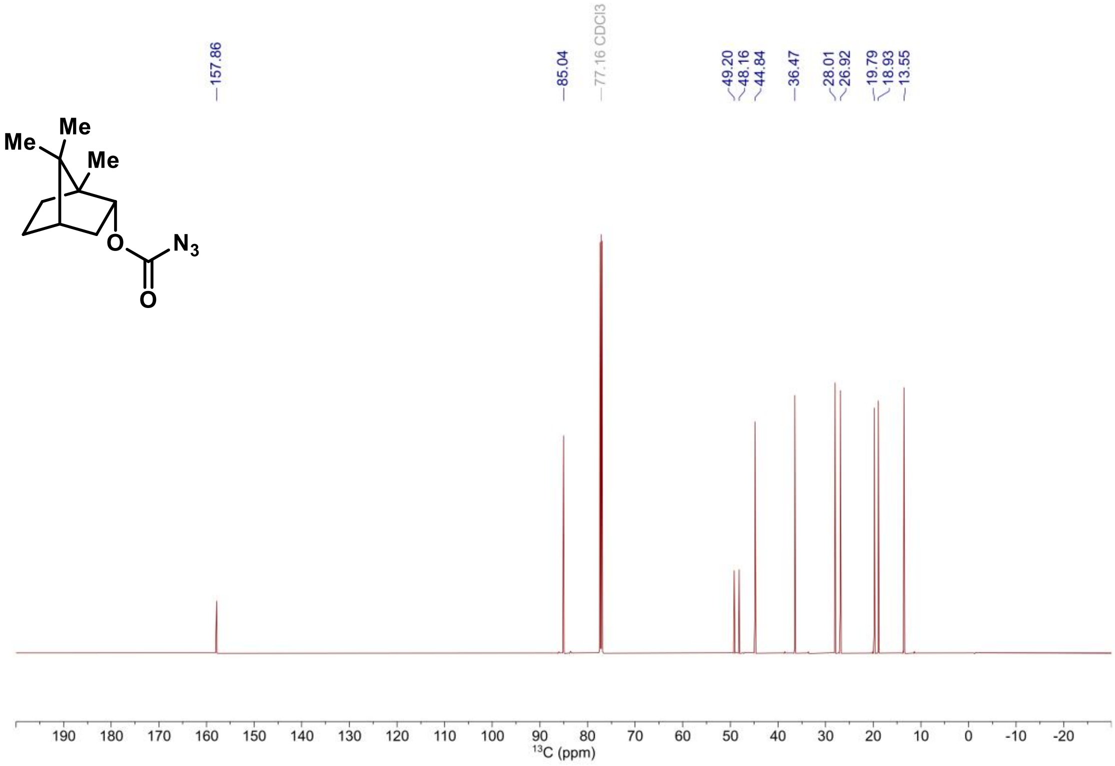

Compound **6s**: ^{1}H-^{13}C HSQC

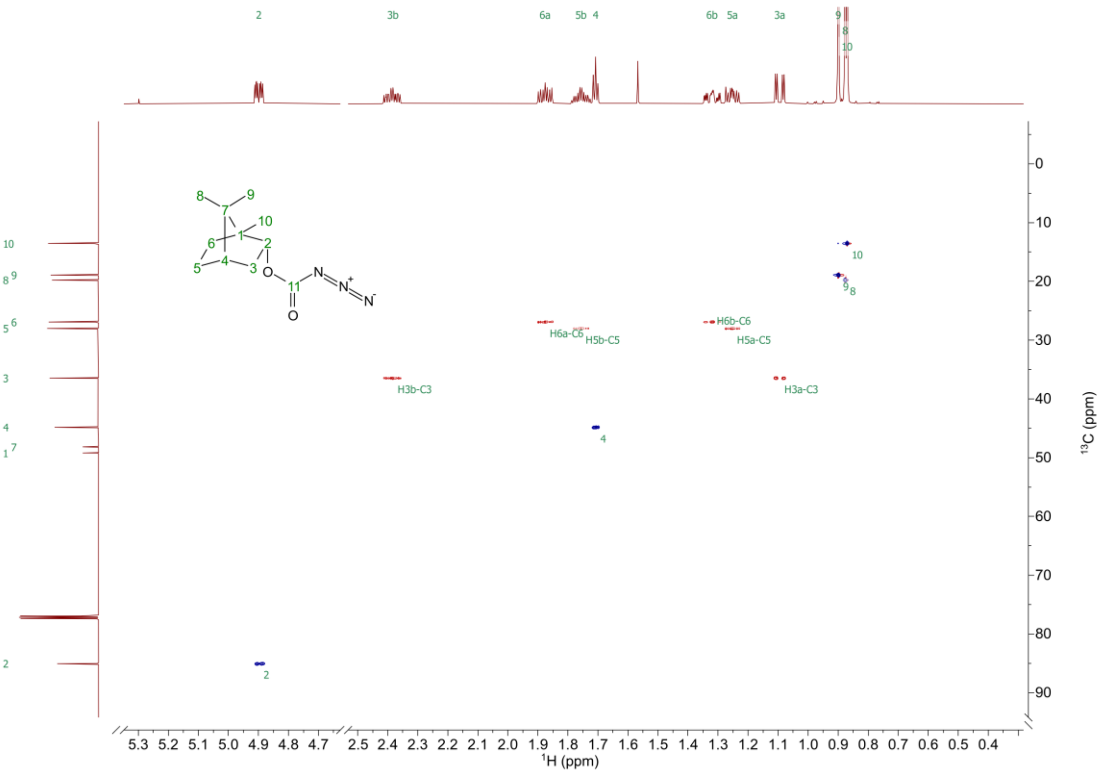

Compound **6s**: ^{1}H-^{13}C HMBC

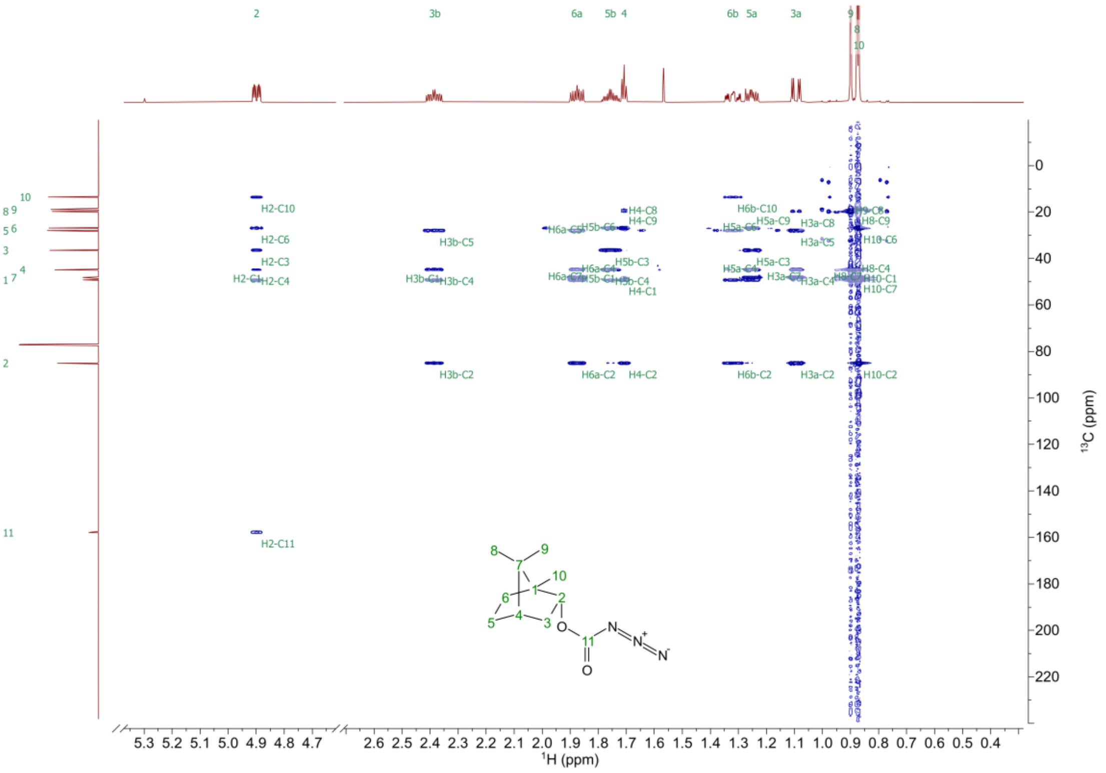

Compound **6s**: ^{1}H-^{1}H COSY

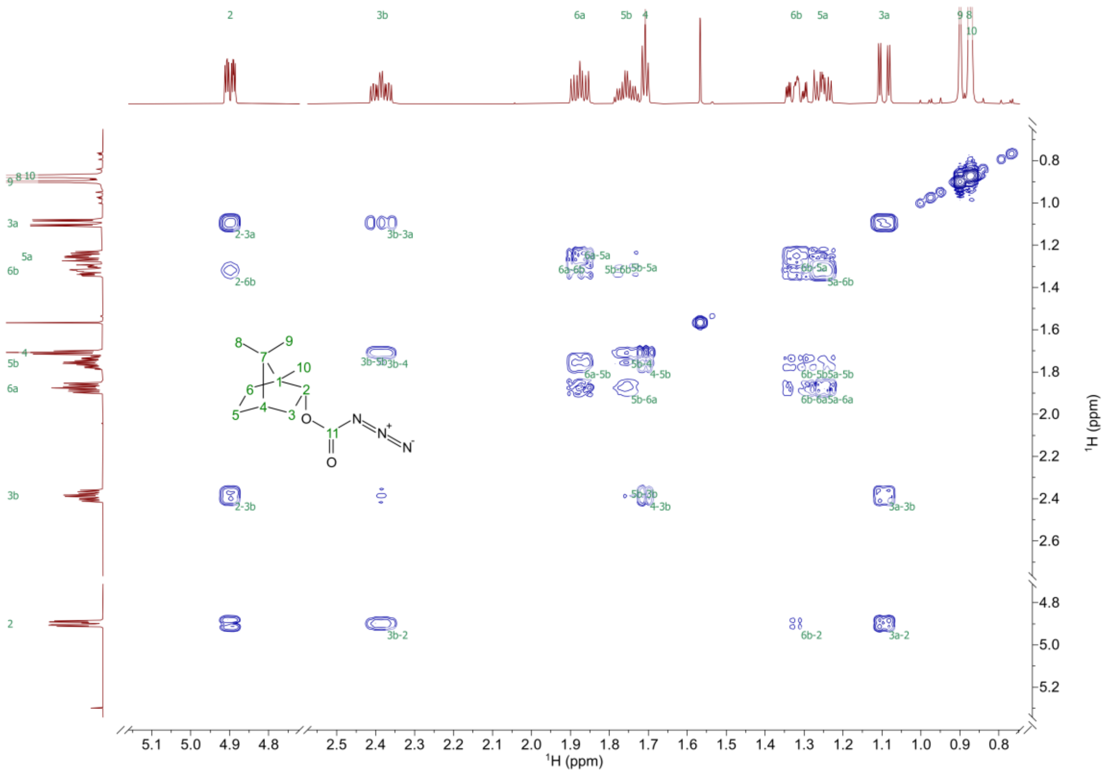

Compound **6s**: ^{1}H-^{1}H NOESY

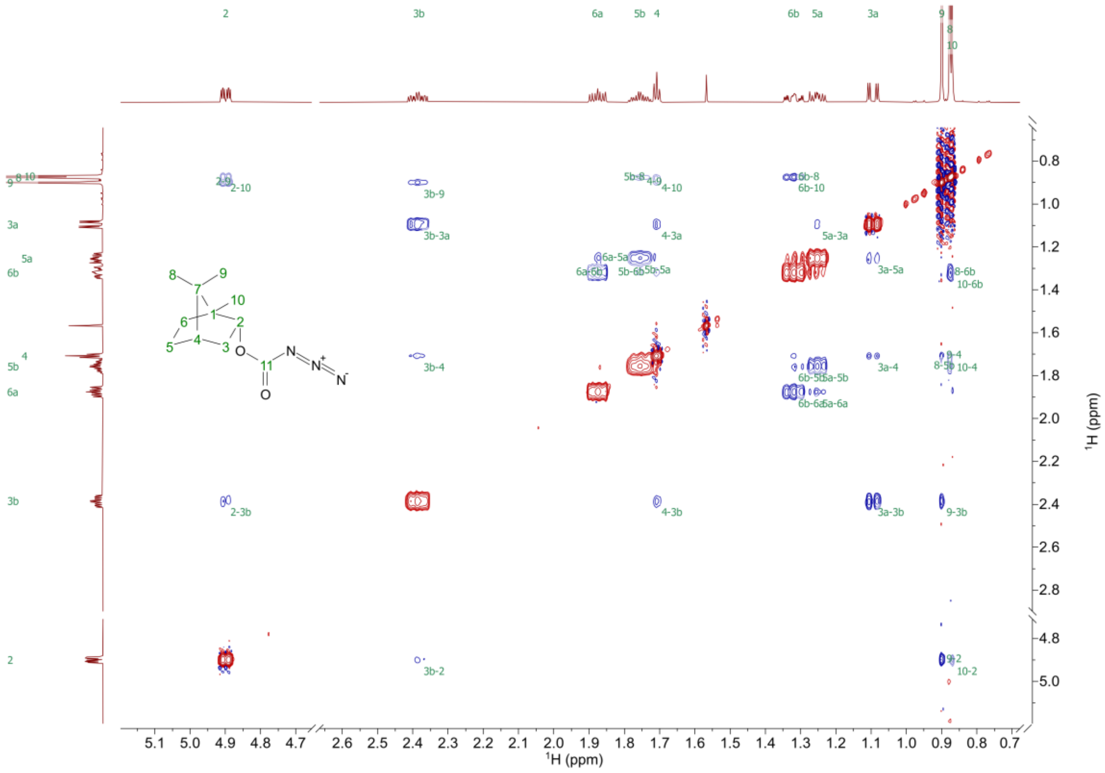

Compound **5e**: ^{1}H NMR (600 MHz, $CDCl_3$, 298 K)

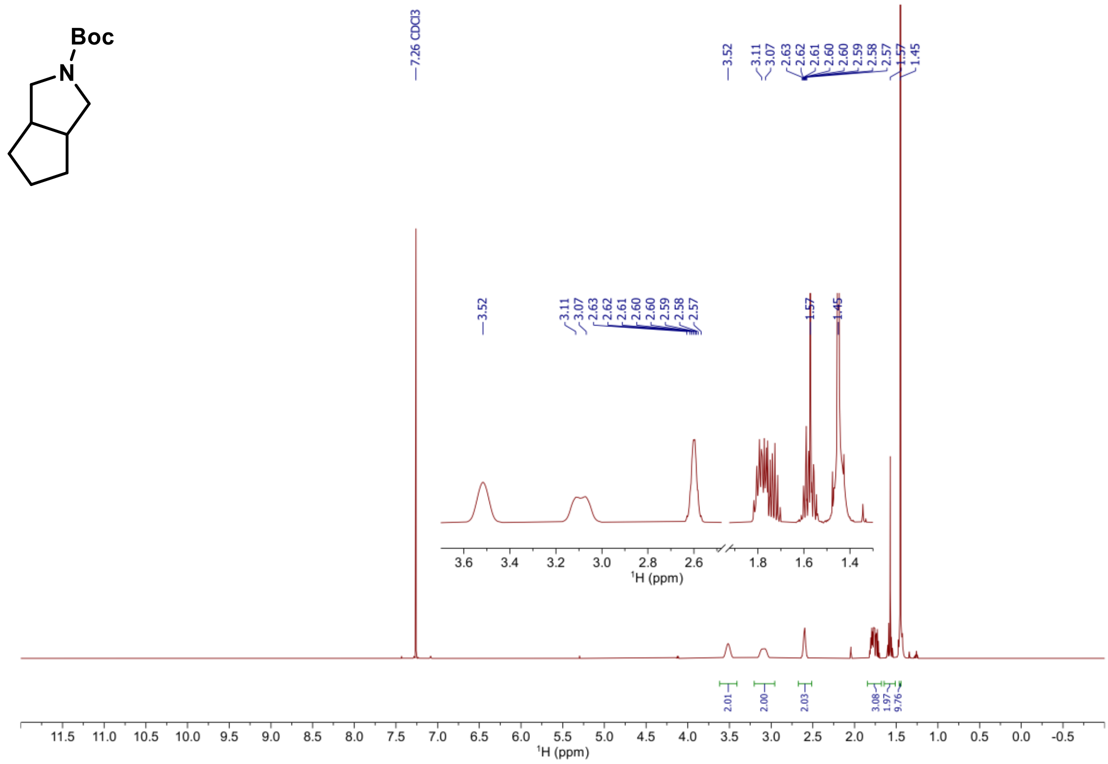

Compound **5e**: ^{13}C NMR (151 MHz, $CDCl_3$, 298 K)

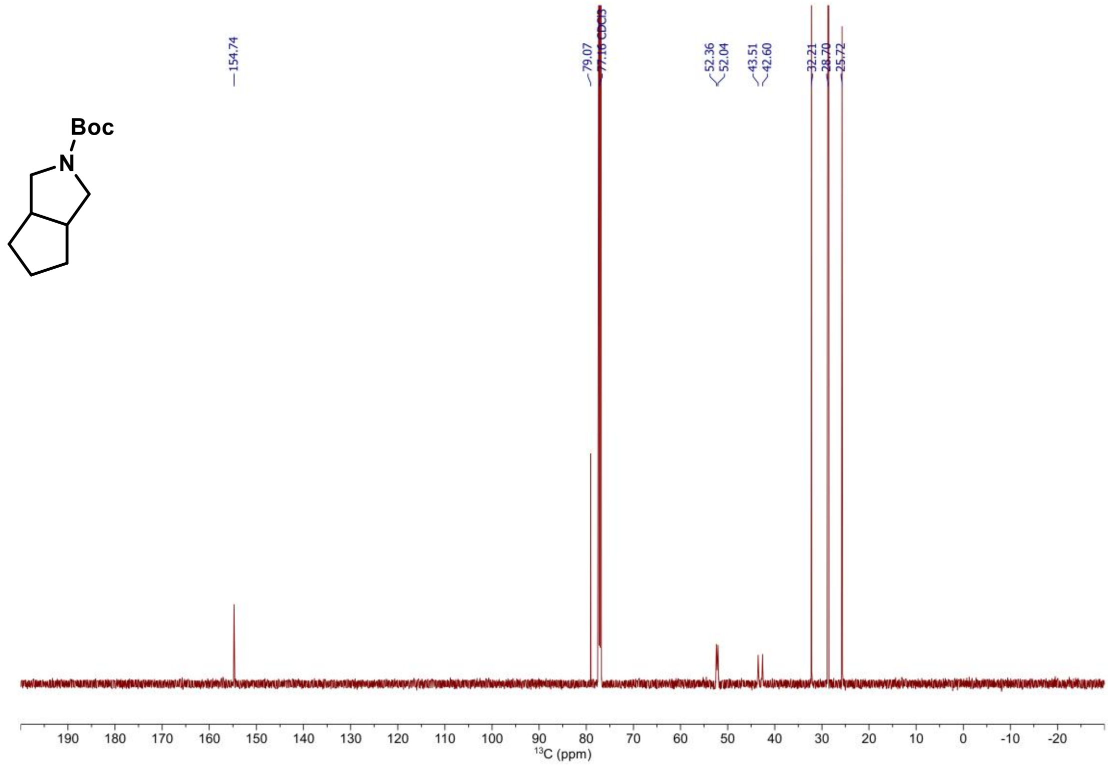

Compound **5j**: ^{1}H NMR (600 MHz, $CDCl_3$, 298 K)

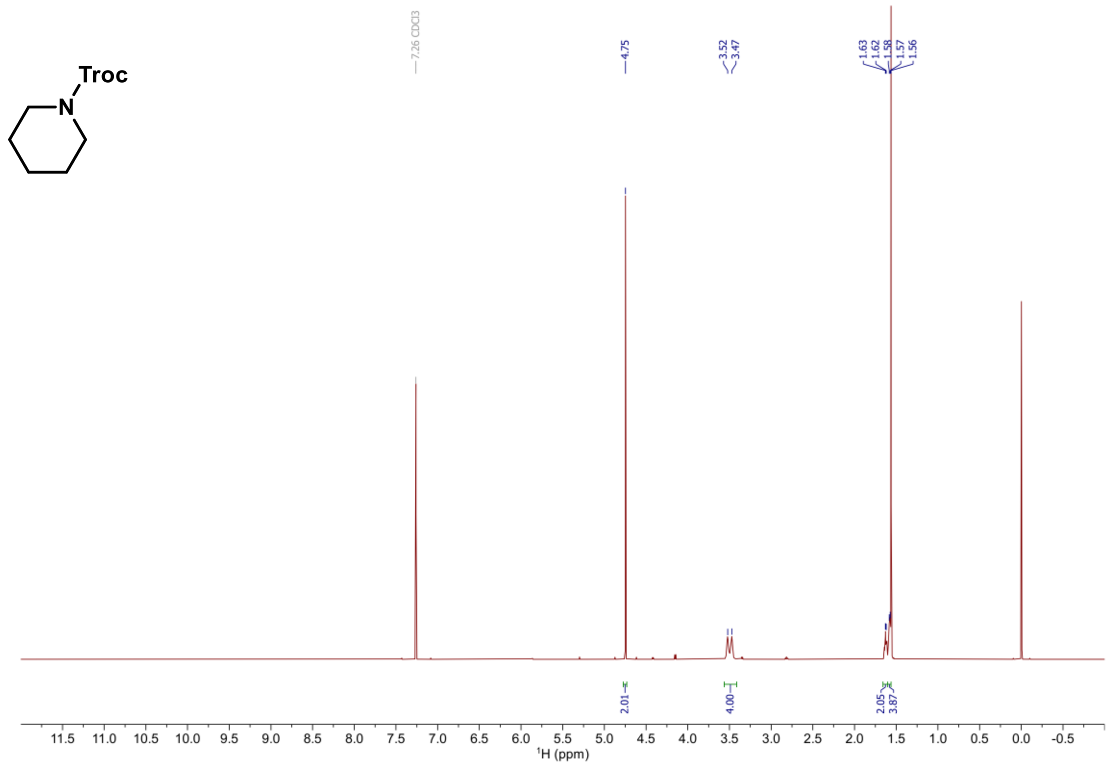

Compound **5j**: ^{13}C NMR (151 MHz, $CDCl_3$, 298 K)

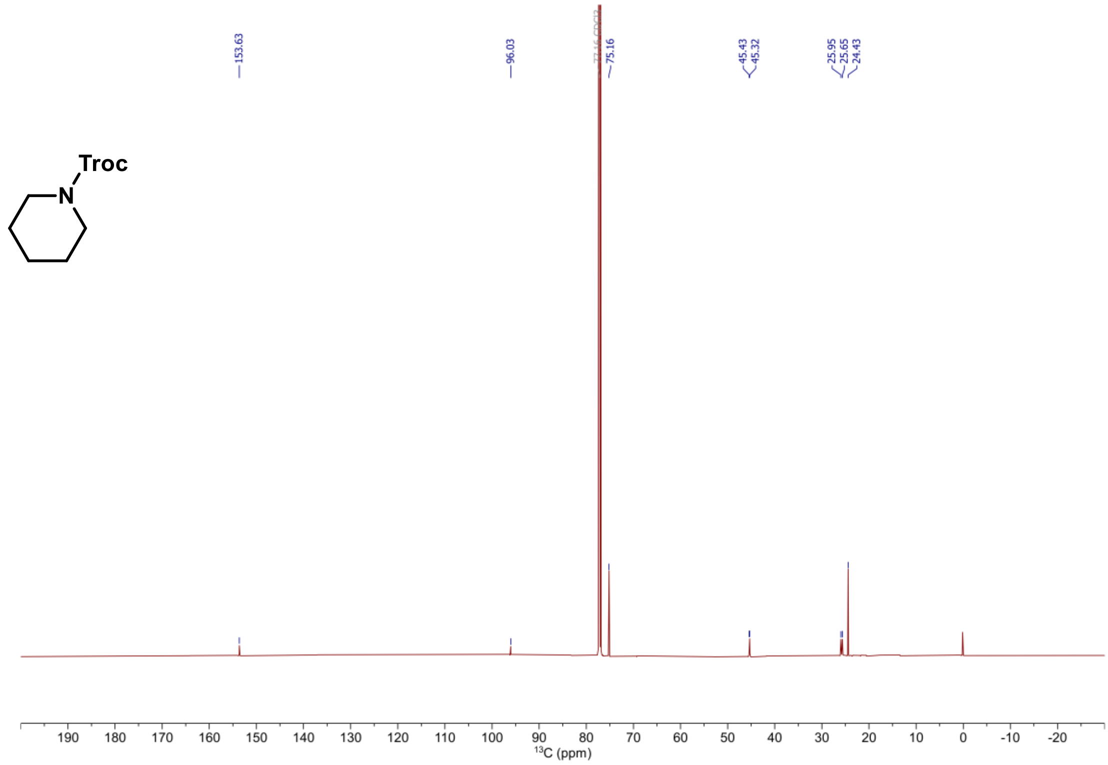

Compound **4a**: ^{1}H NMR (600 MHz, $CDCl_3$, 298 K)

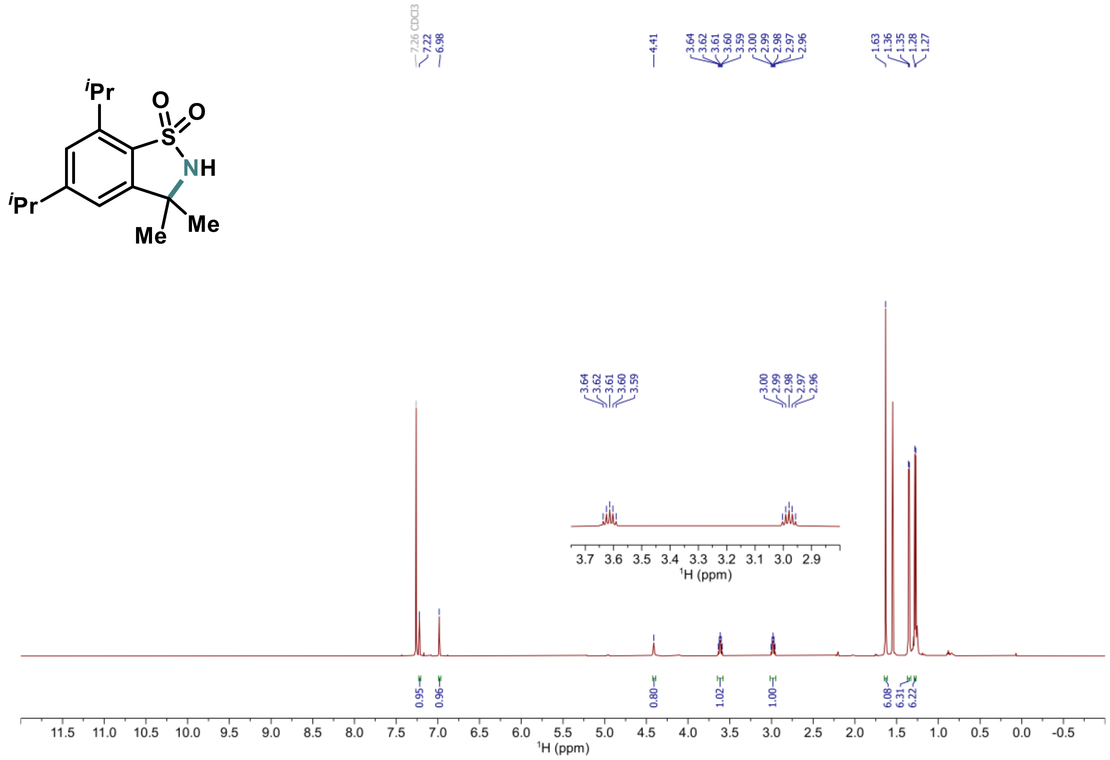

Compound **4a**: ^{13}C NMR (151 MHz, $CDCl_3$, 298 K)

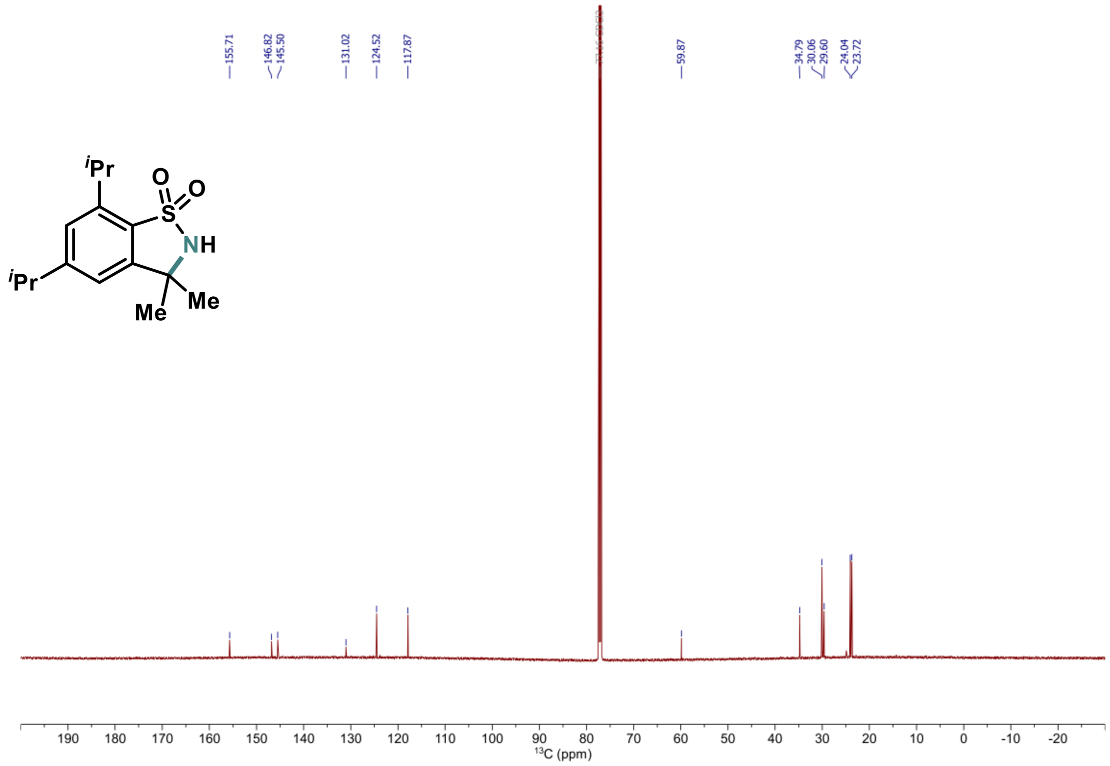

Compound **4c**: ^{1}H NMR (600 MHz, $CDCl_3$, 298 K)

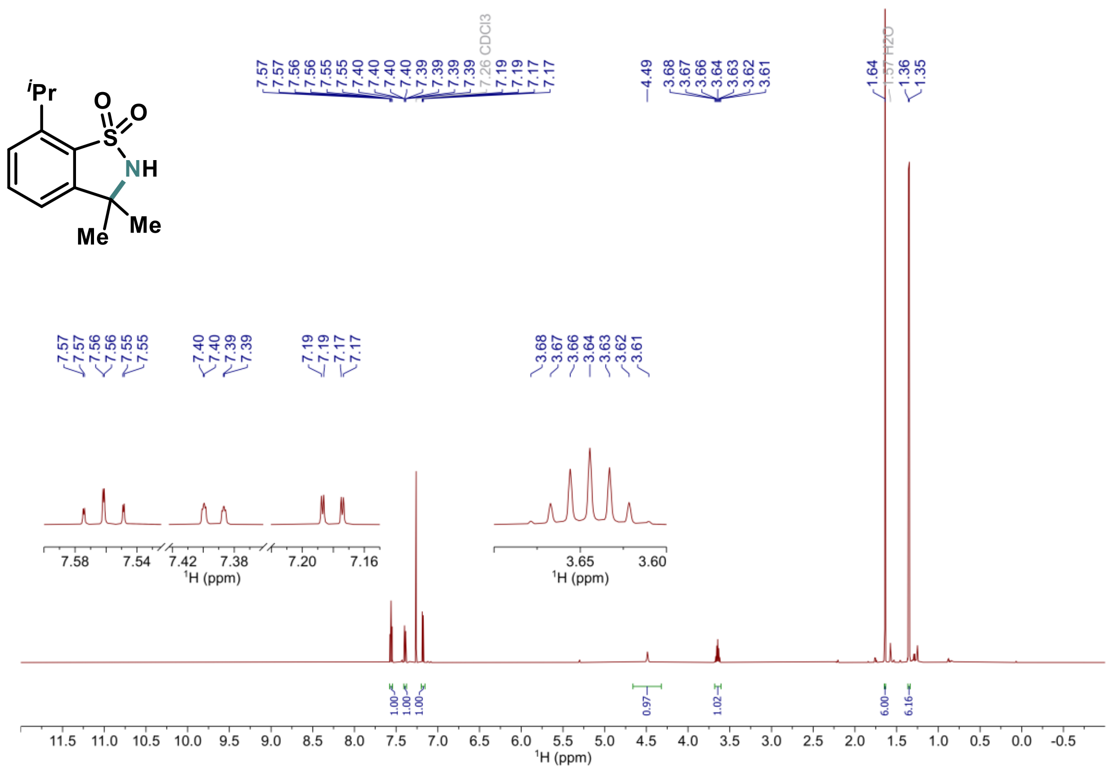

Compound **4c**: ^{13}C NMR (151 MHz, $CDCl_3$, 298 K)

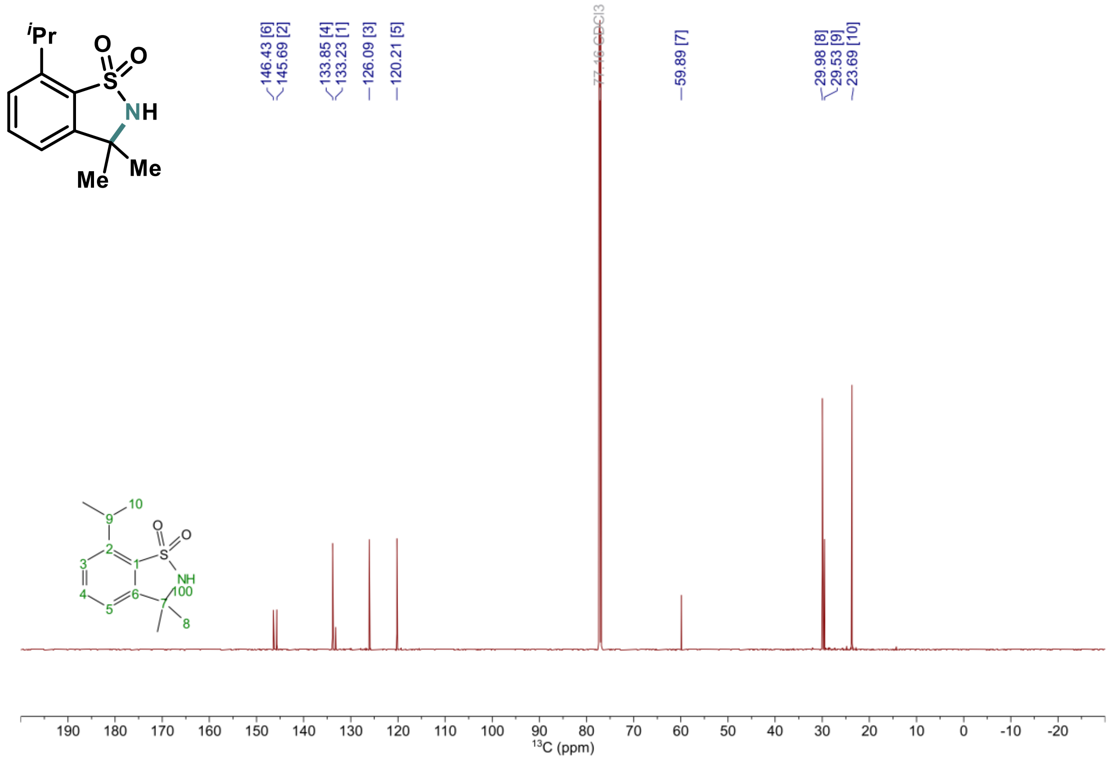

Compound **4c**: ^{1}H-^{13}C HSQC

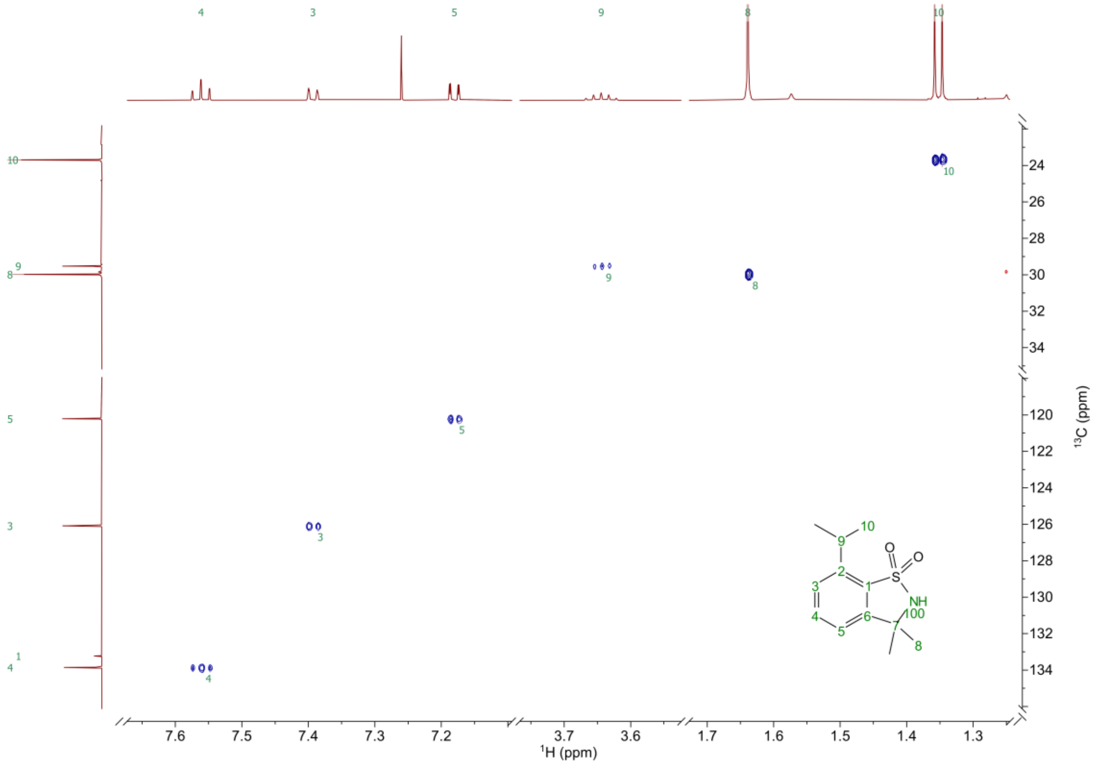

Compound **4c**: ^{1}H-^{13}C HMBC

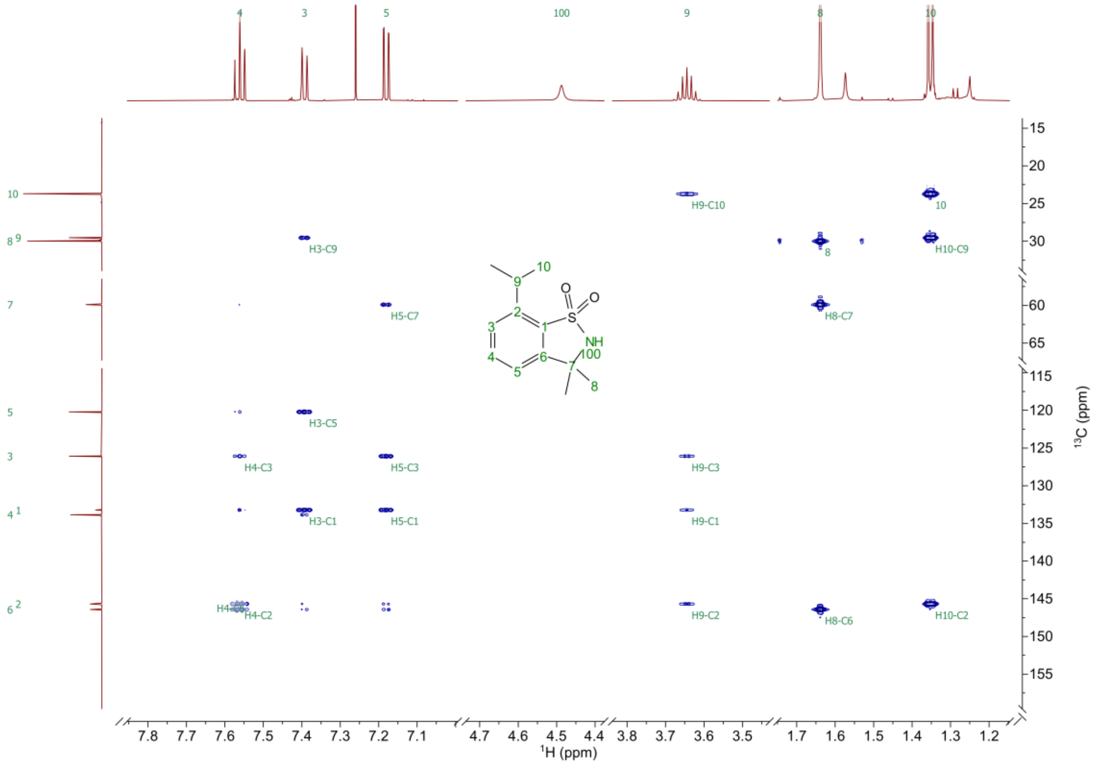

Compound **4c**: ^{1}H-^{1}H COSY

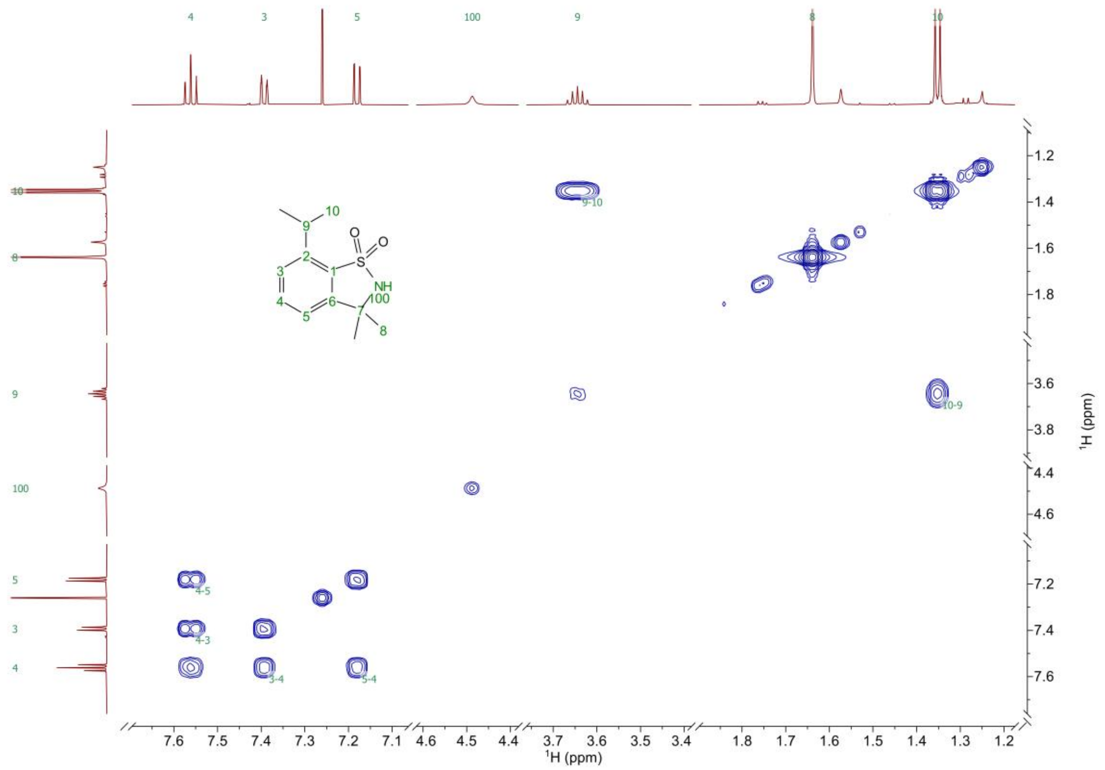

Compound **4c**: ^{1}H-^{1}H NOESY

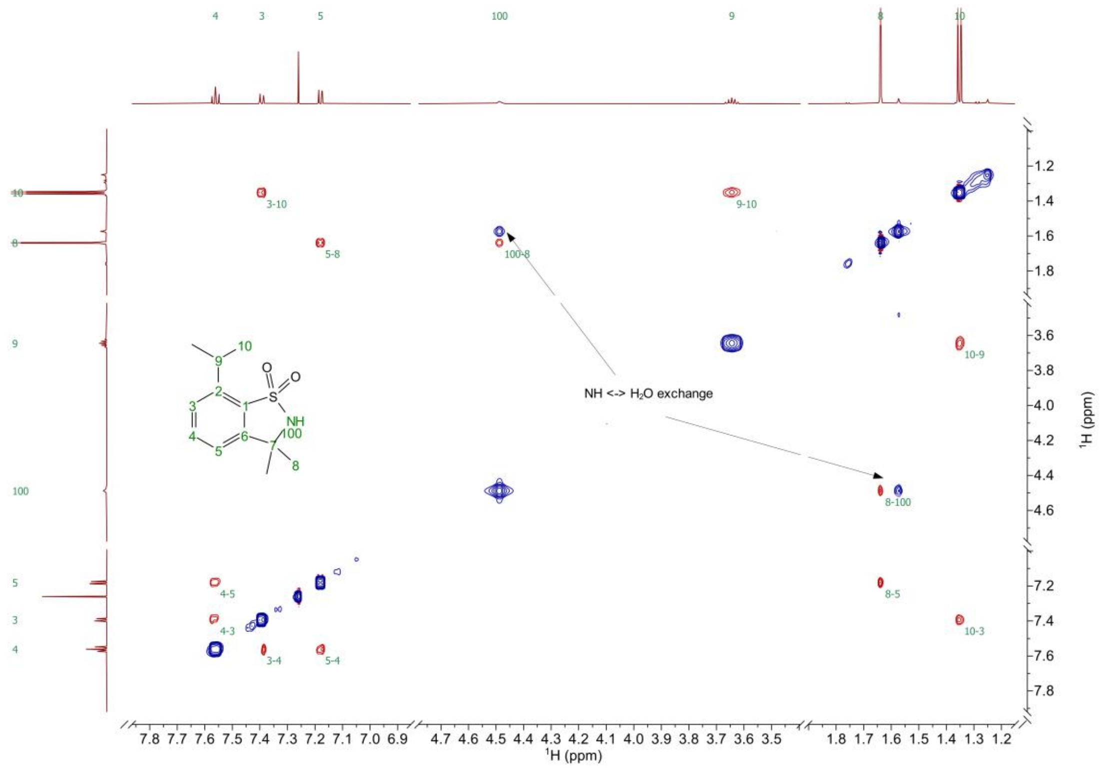

Compound **4c**: ^{1}H-^{15}N HMBC

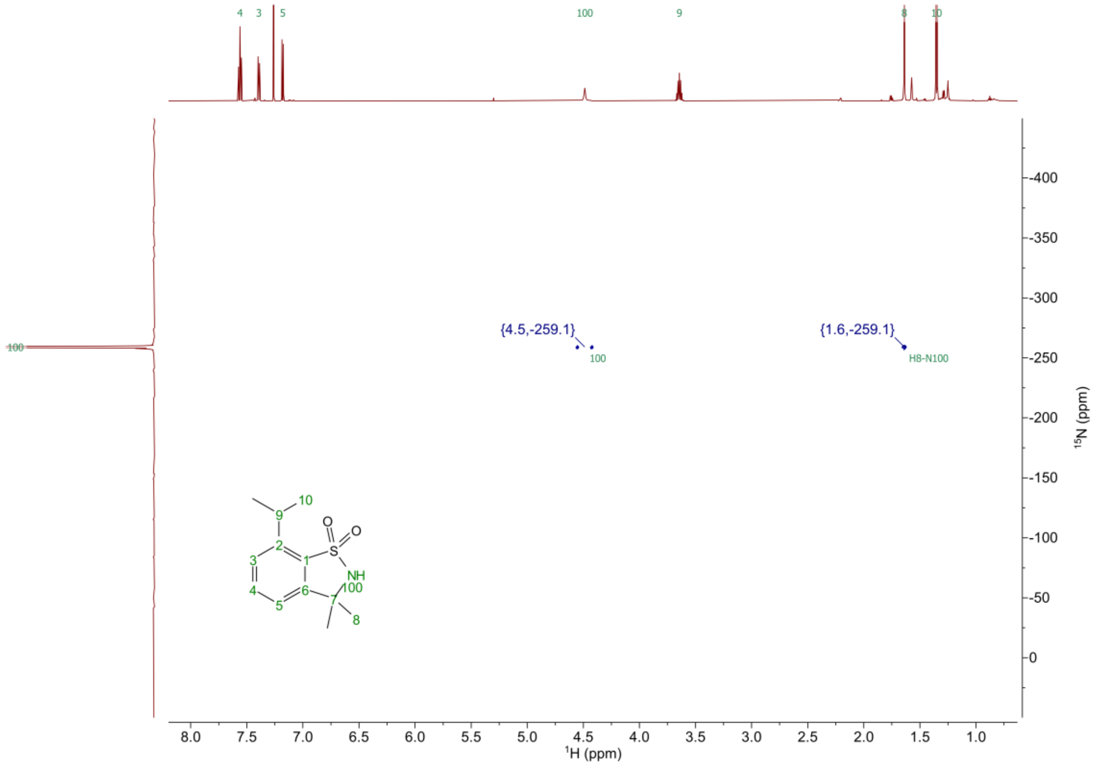

Compound **4d**: ^{1}H NMR (600 MHz, $CDCl_3$, 298 K)

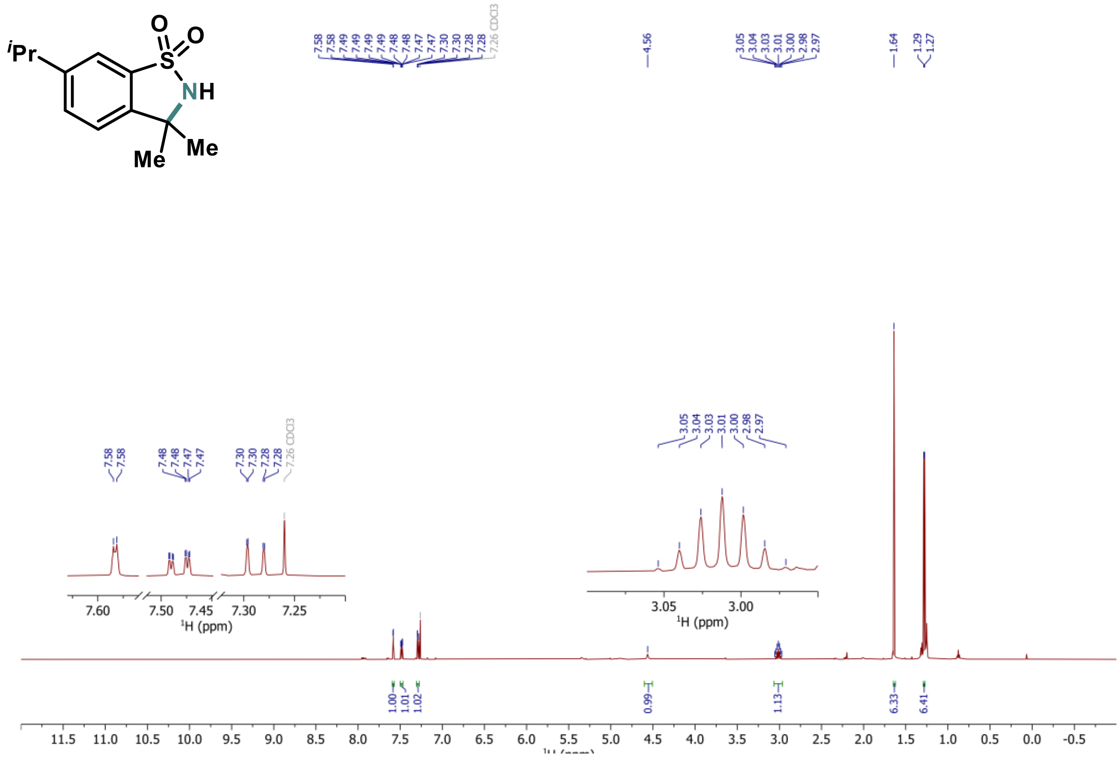

Compound **4d**: ^{13}C NMR (151 MHz, $CDCl_3$, 298 K)

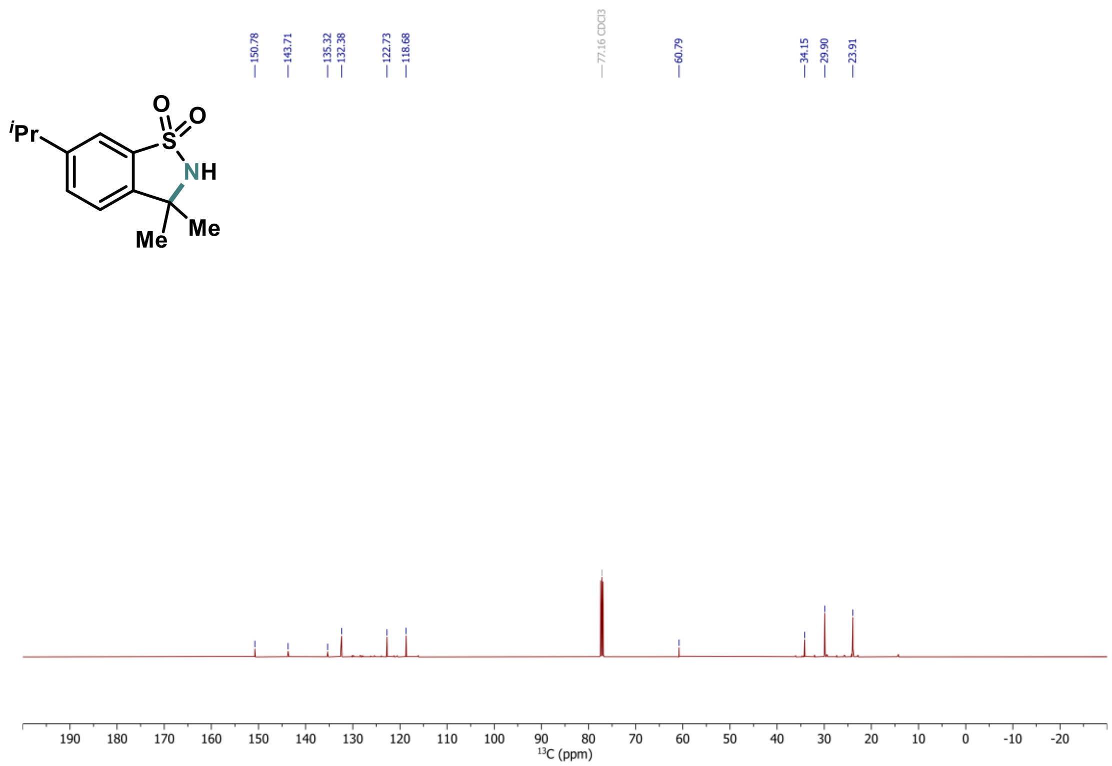

Compound **4e**: ^{1}H NMR (600 MHz, $CDCl_3$, 298 K)

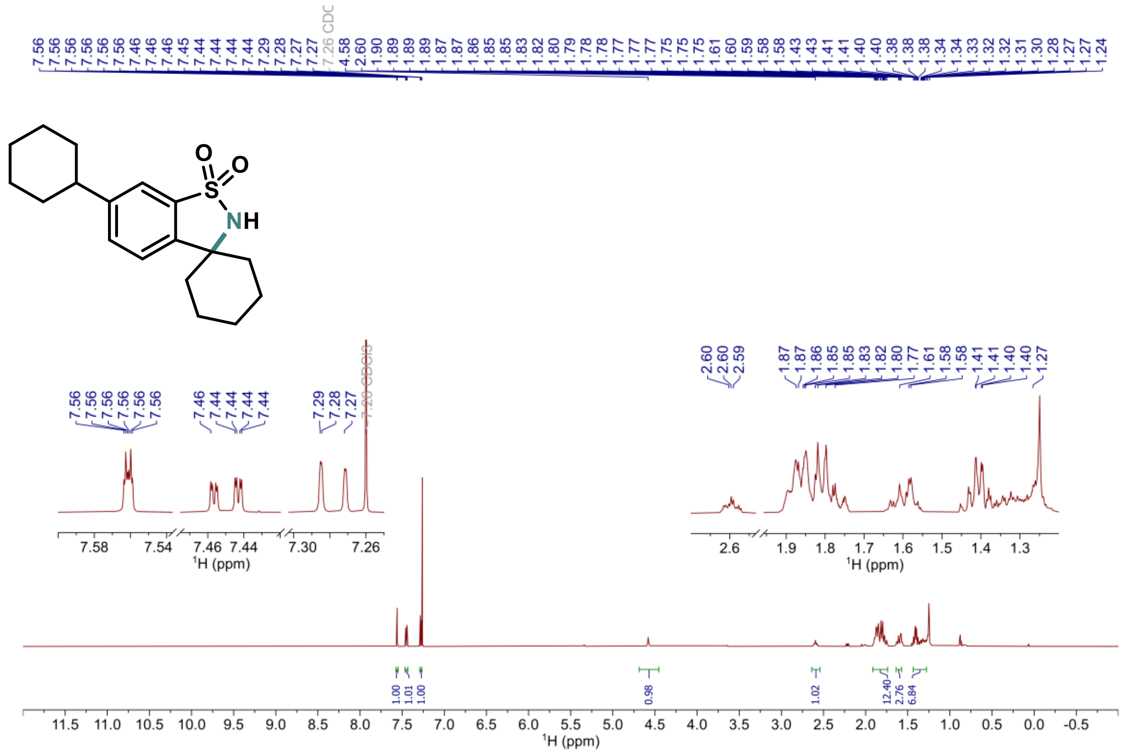

Compound **4e**: ^{13}C NMR (151 MHz, $CDCl_3$, 298 K)

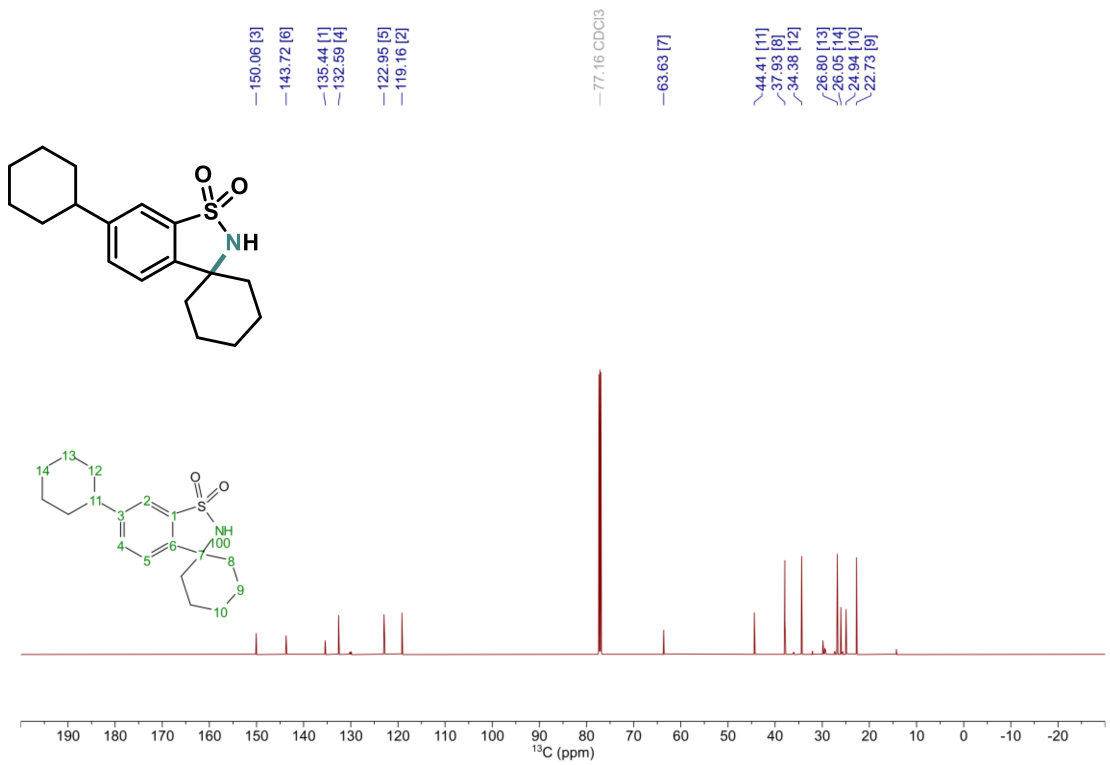

Compound **4e**: ^{1}H-^{13}C HSQC

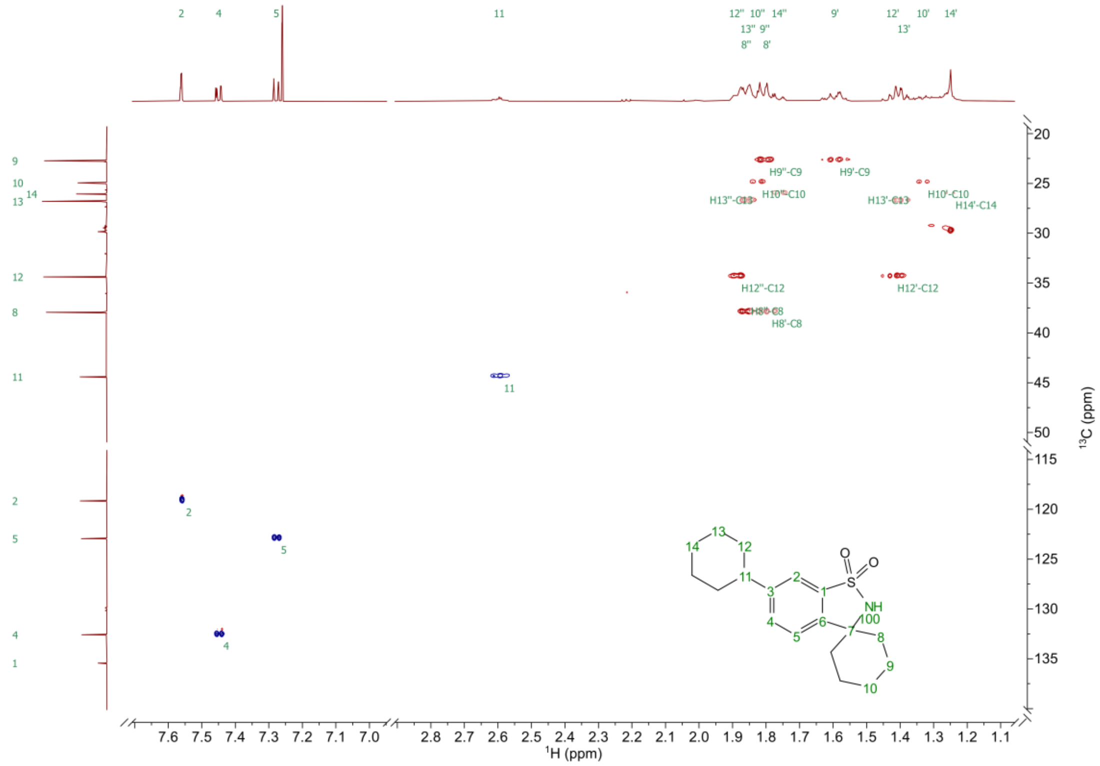

Compound **4e**: ^{1}H-^{13}C HMBC

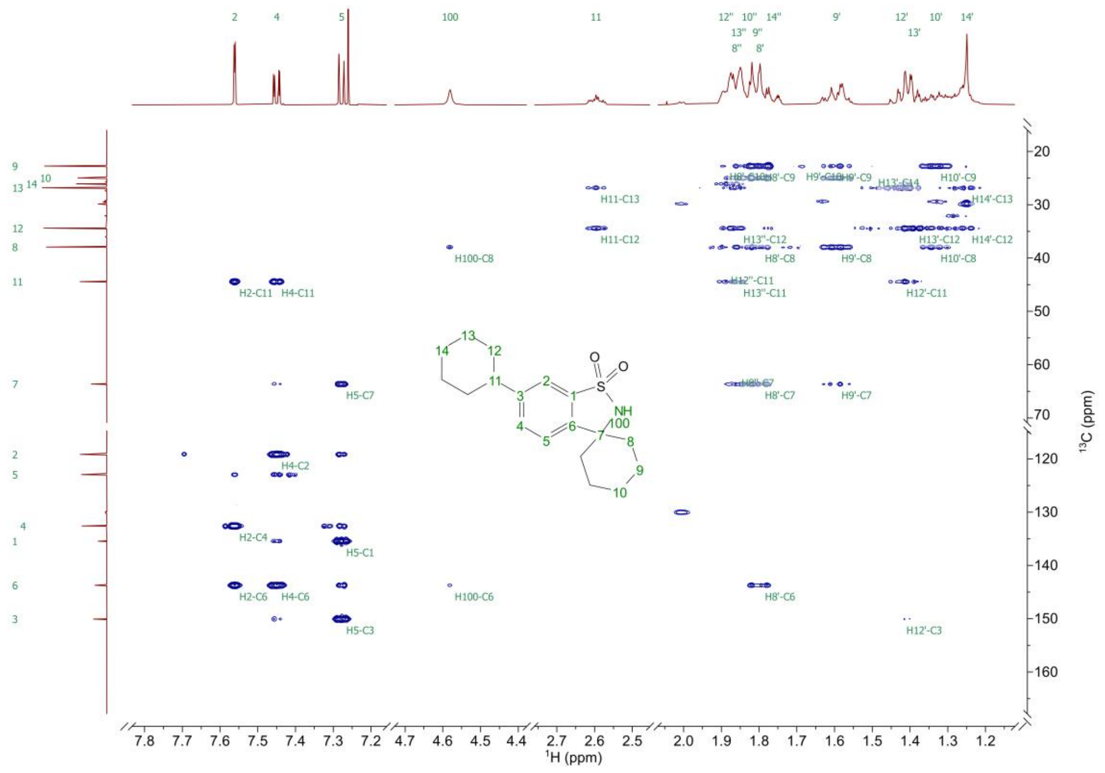

Compound **4e**: ^{1}H-^{1}H COSY

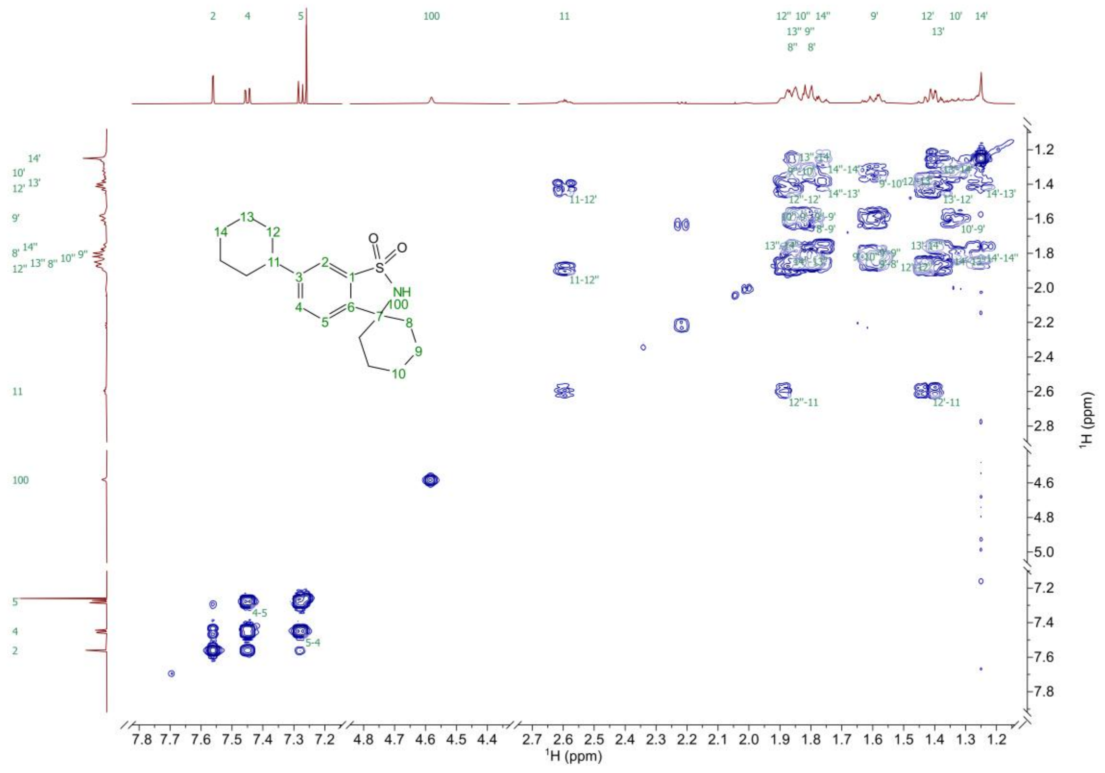

Compound **4e**: ^{1}H-^{1}H NOESY

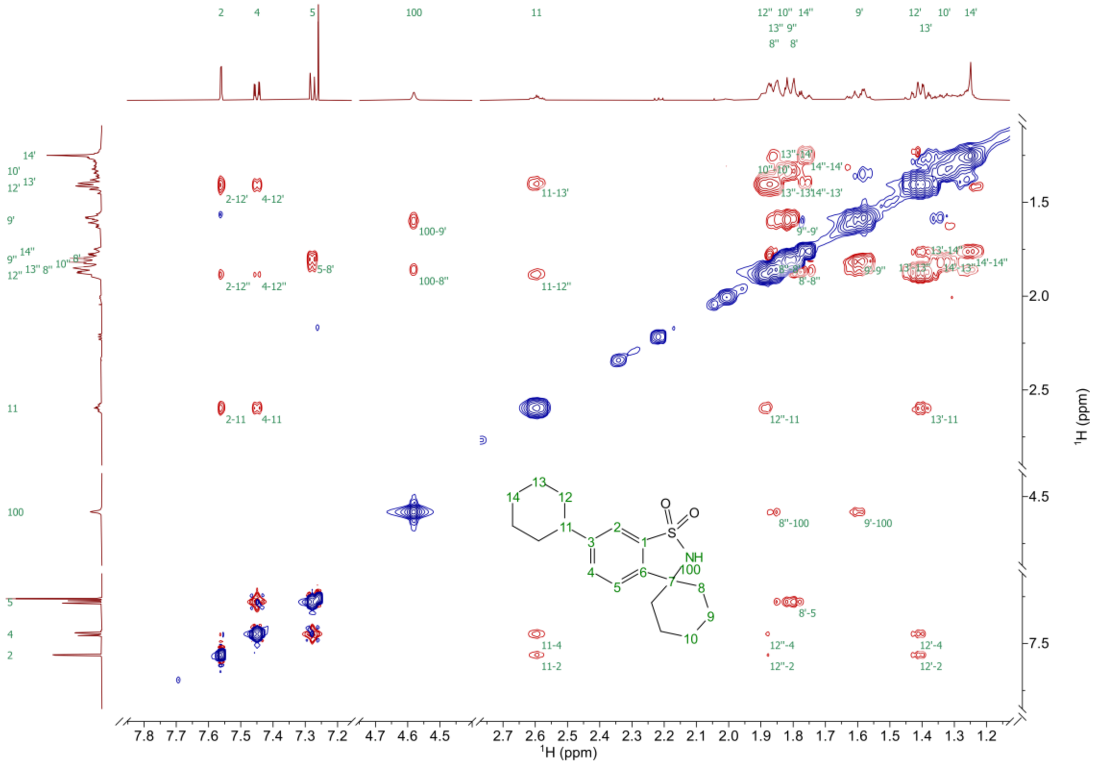

Compound **4e**: ^{1}H-^{15}N HMBC

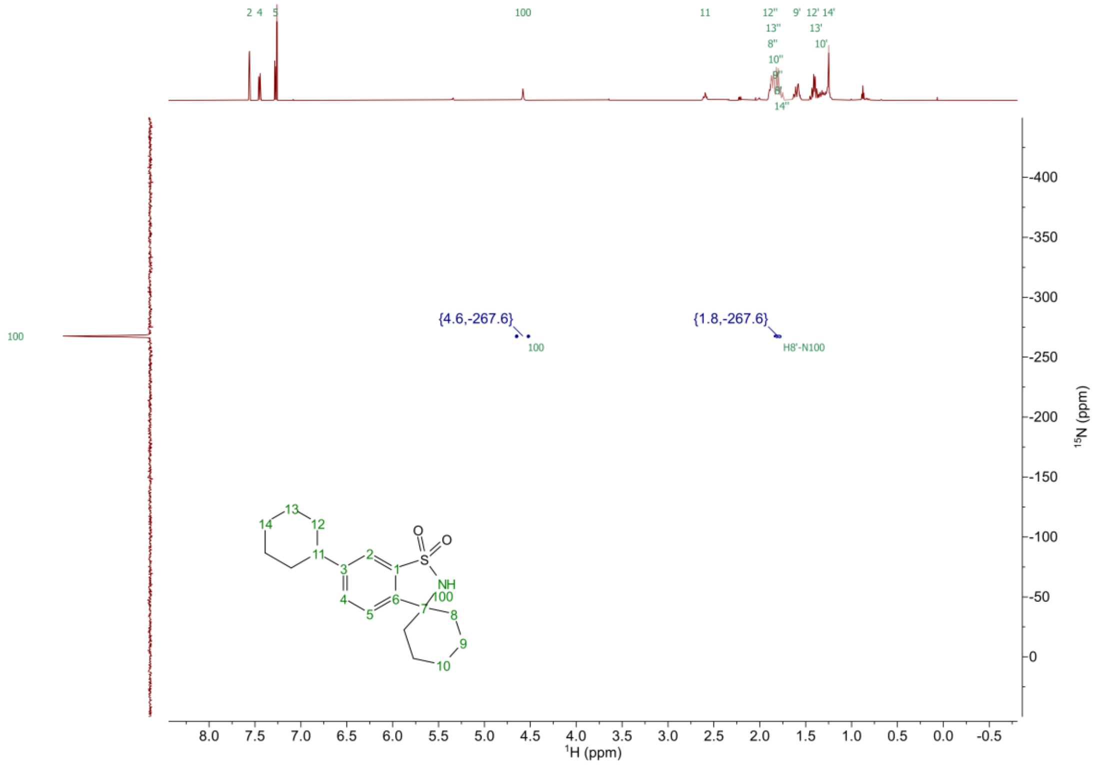

Compound **4f**: ^{1}H NMR (600 MHz, $CDCl_3$, 298 K)

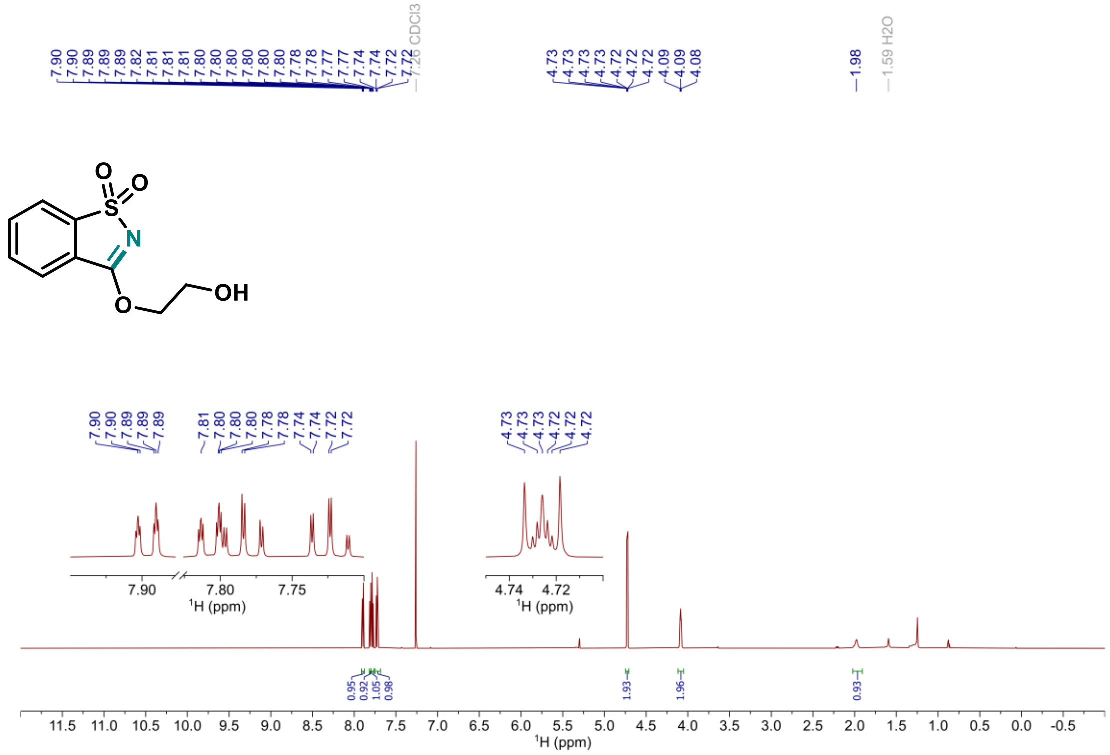

Compound **4f**: ^{13}C NMR (151 MHz, $CDCl_3$, 298 K)

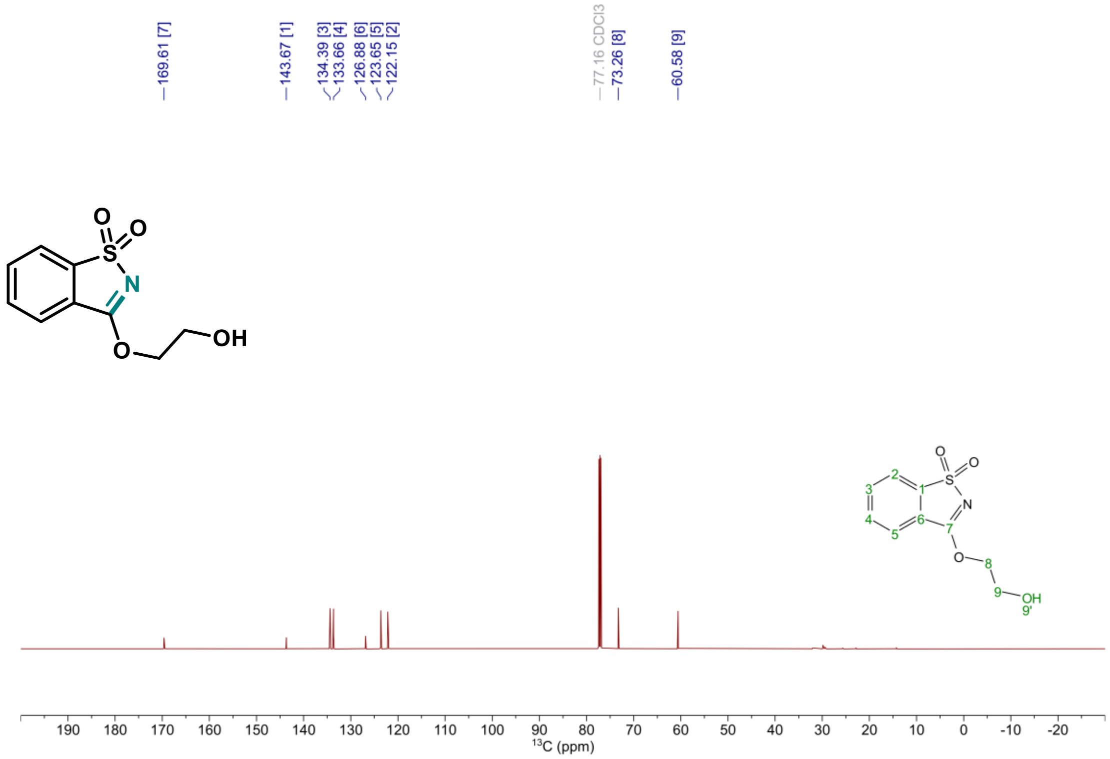

Compound **4f**: ^{1}H-^{13}C HSQC

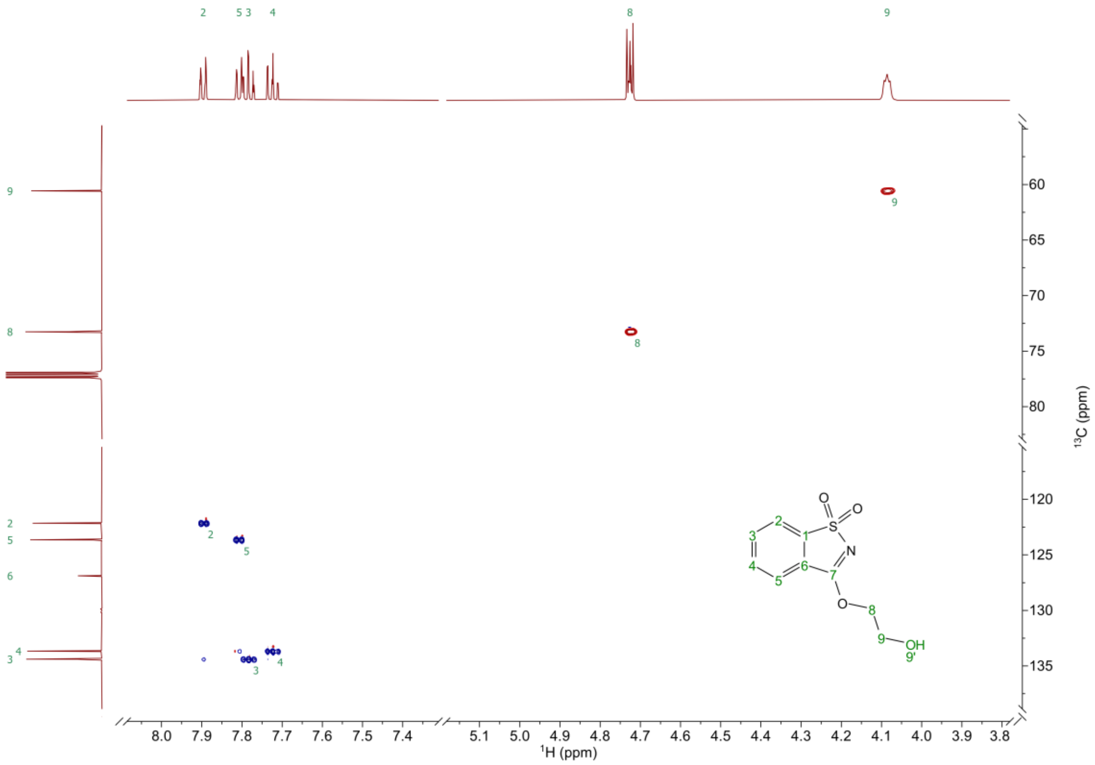

Compound **4f**: ^{1}H-^{13}C HMBC

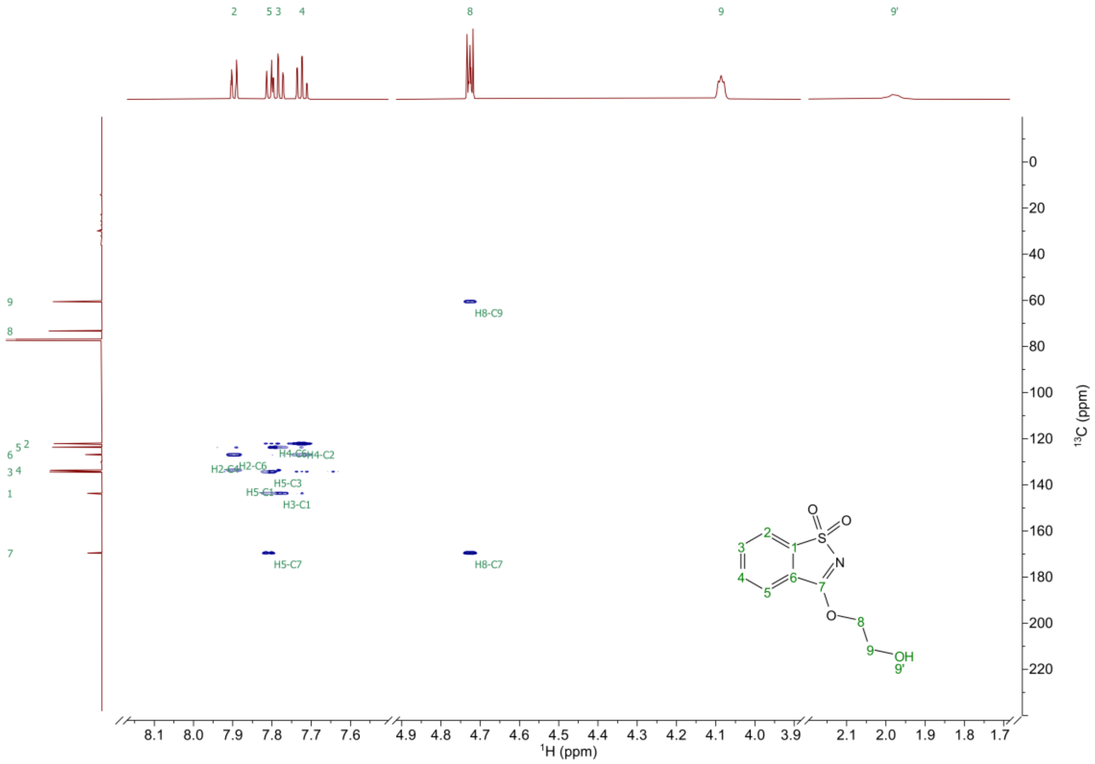

Compound **4f**: ^1H-^1H COSY

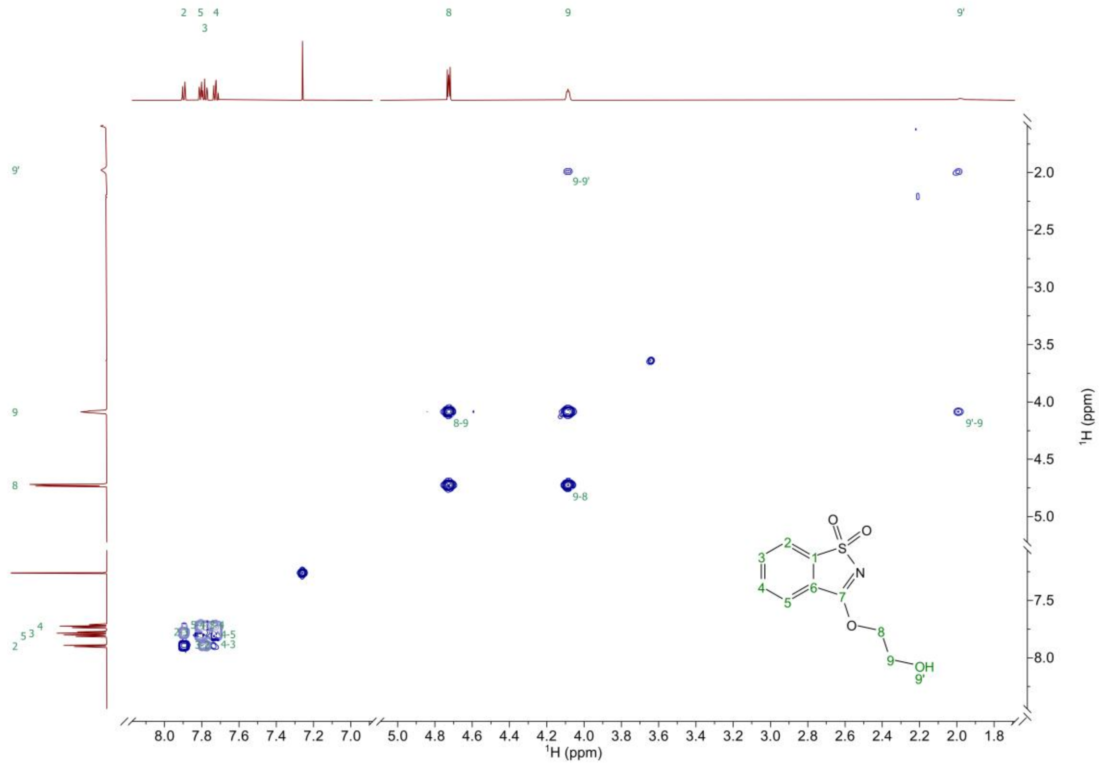

Compound **4f**: ^1H-^1H NOESY

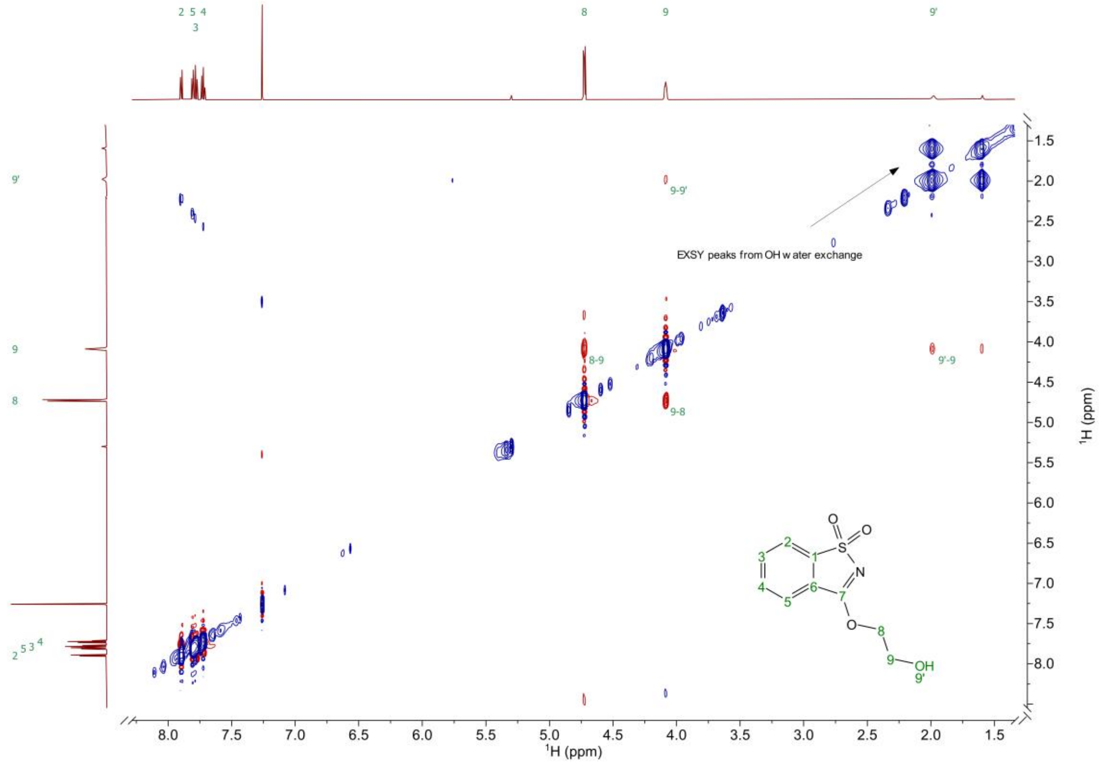

Compound **4g**: ^{1}H NMR (600 MHz, $CDCl_3$, 298 K)

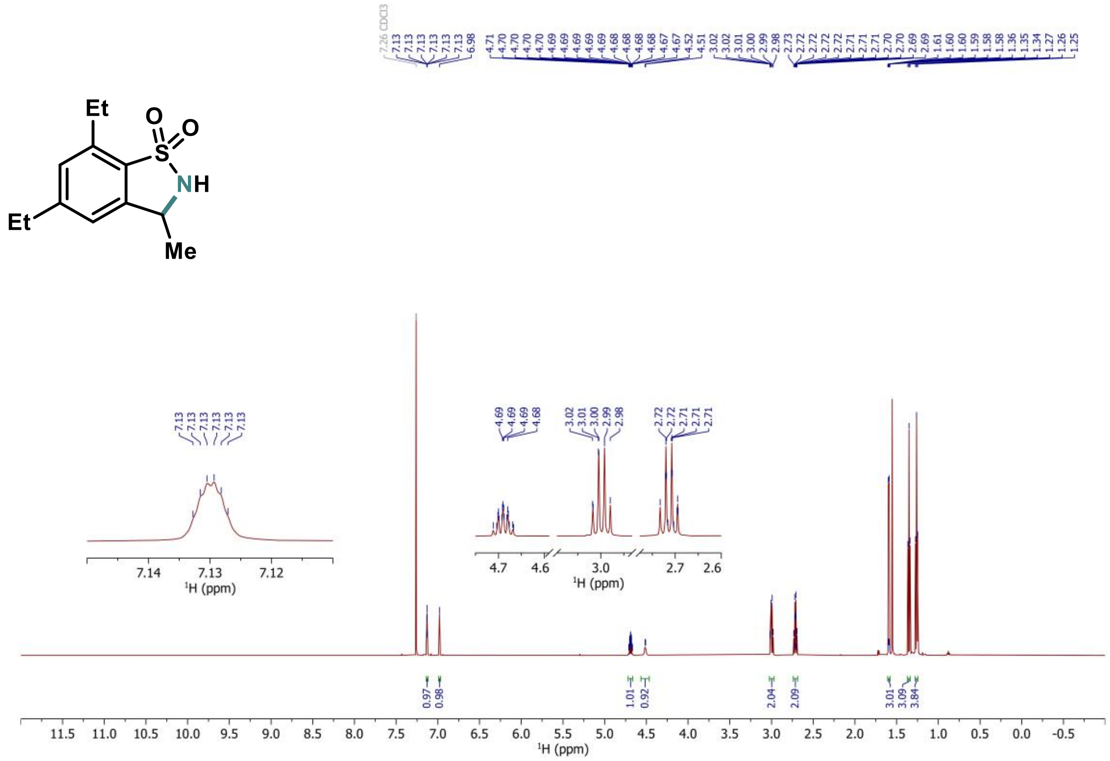

Compound **4g**: ^{13}C NMR (151 MHz, $CDCl_3$, 298 K)

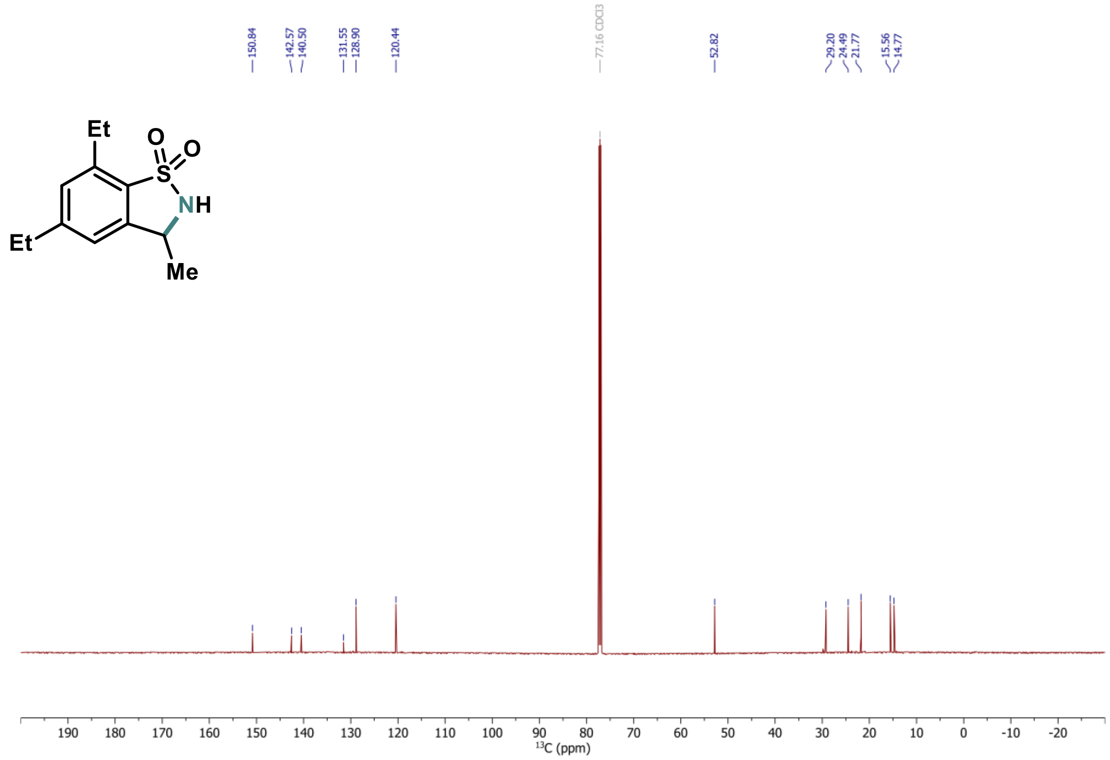

Compound **7a**: ^{1}H NMR (600 MHz, $CDCl_3$, 298 K)

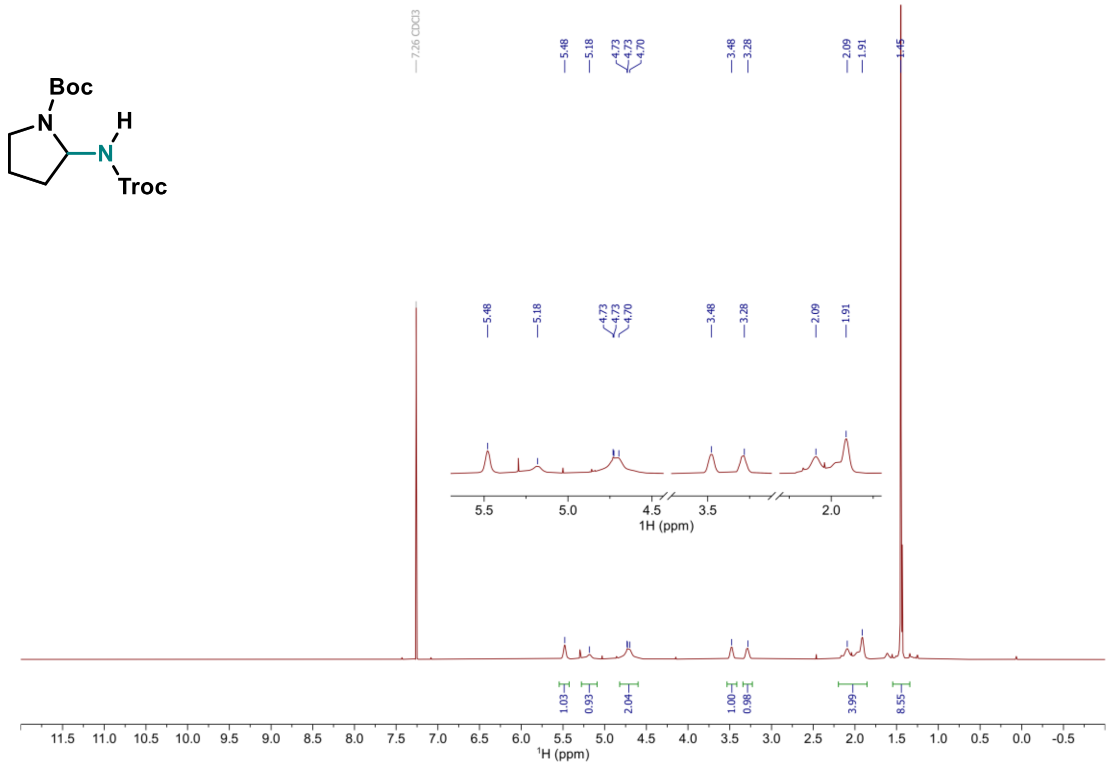

Compound **7a**: ^{13}C NMR (151 MHz, $CDCl_3$, 298 K)

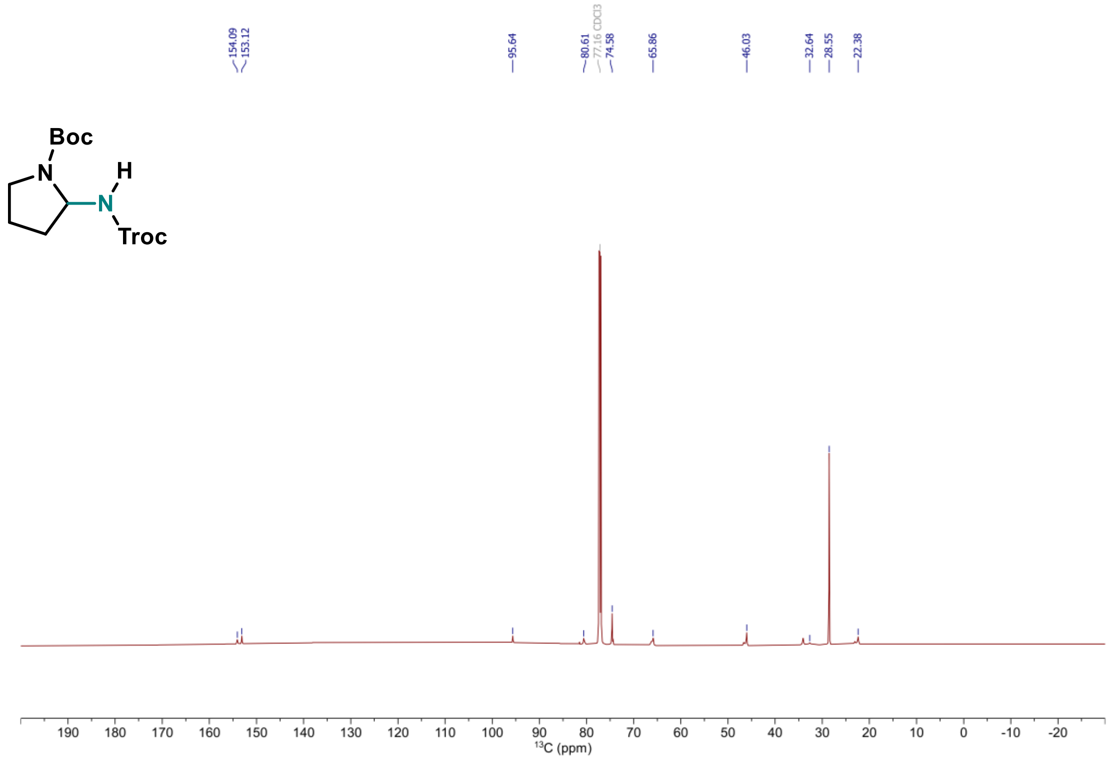

Compound **7b**: ^{1}H NMR (600 MHz, $CDCl_3$, 243 K)

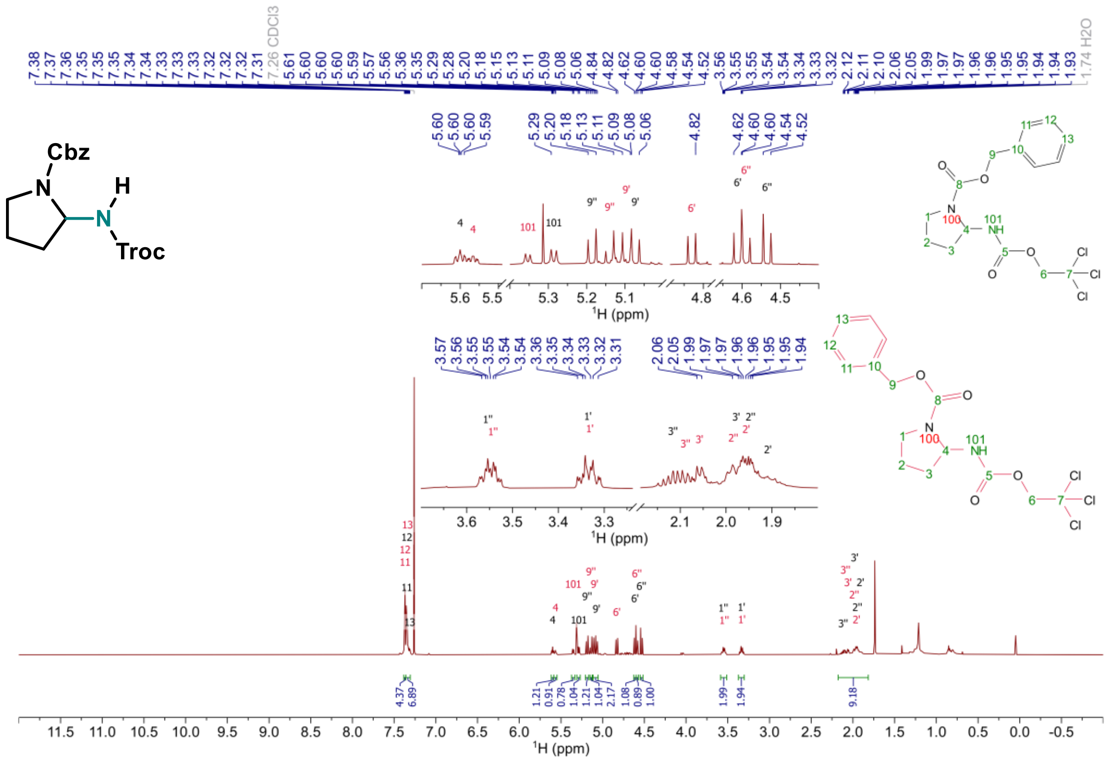

Compound **7b**: ^{13}C NMR (151 MHz, $CDCl_3$, 243 K)

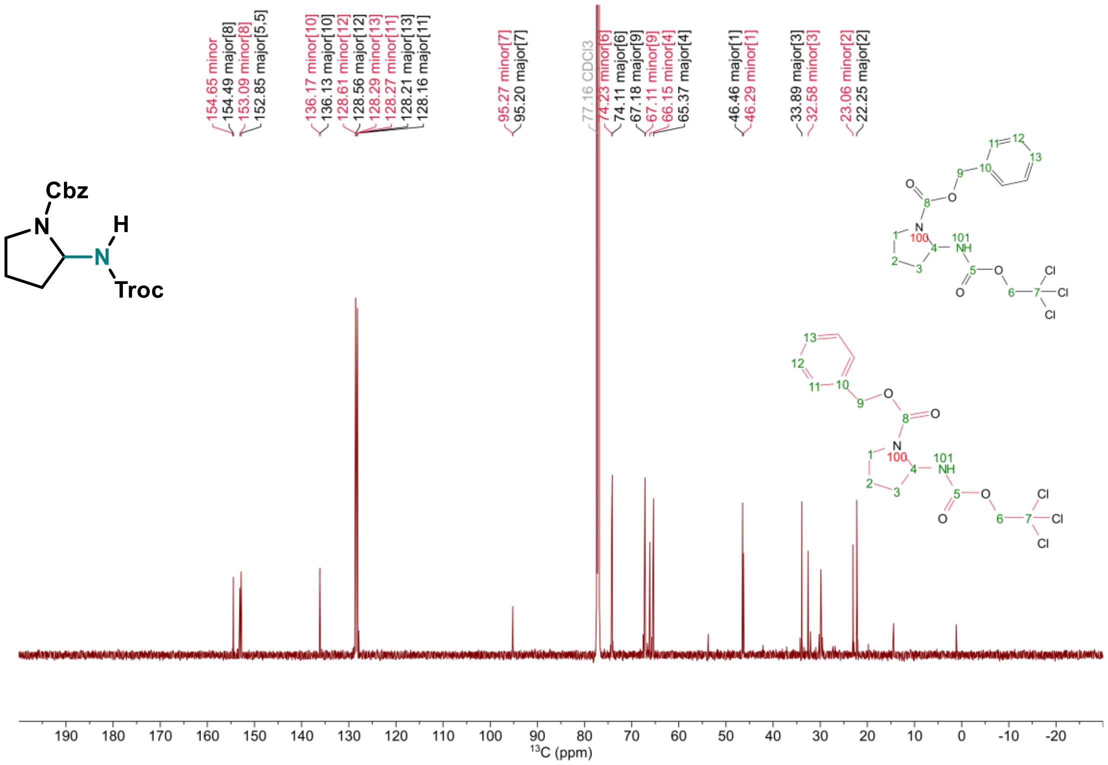

Compound **7b**: variable temperature ^{1}H NMR (600 MHz, $CDCl_3$)

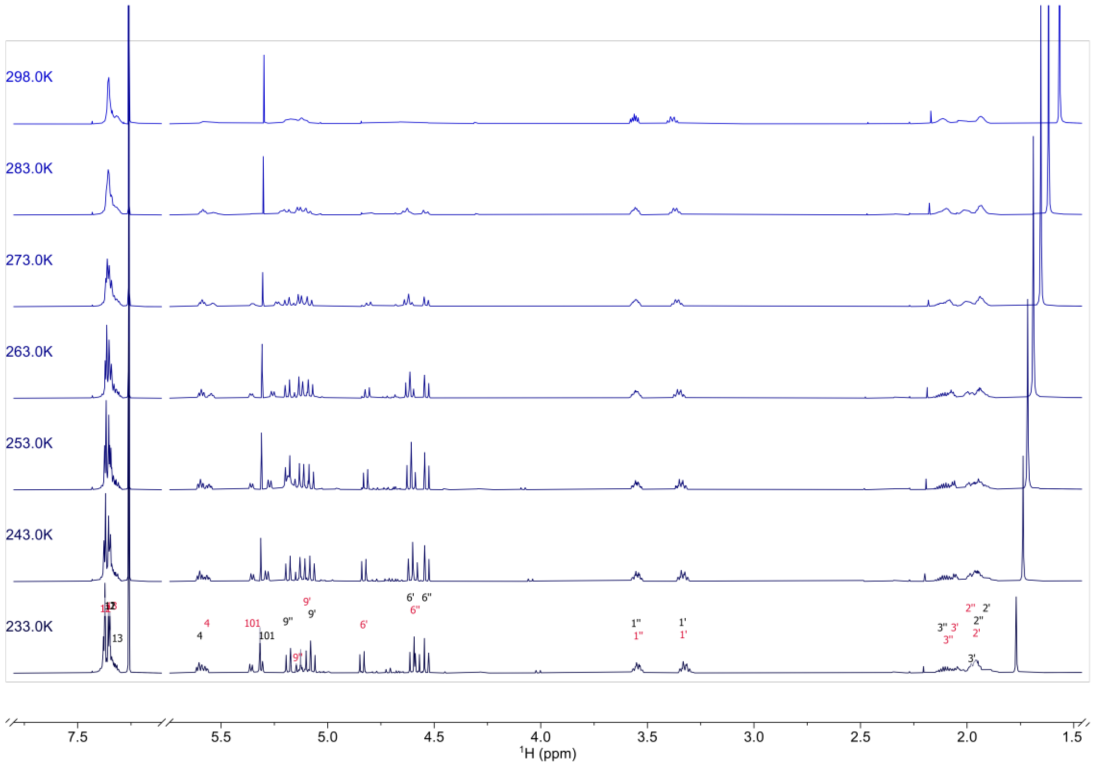

Compound **7b**: ^{1}H-^{13}C HSQC ($CDCl_3$, 243 K)

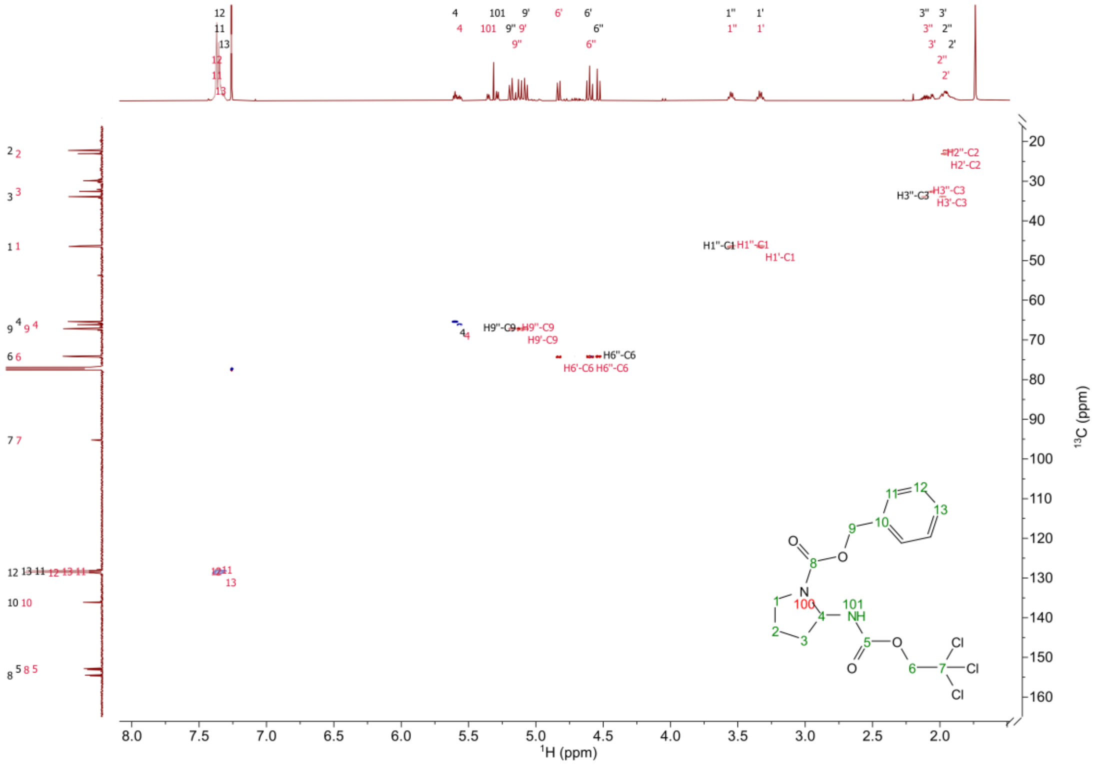

Compound **7b**: ^{1}H-^{13}C HMBC (CDCl$_3$, 243 K)

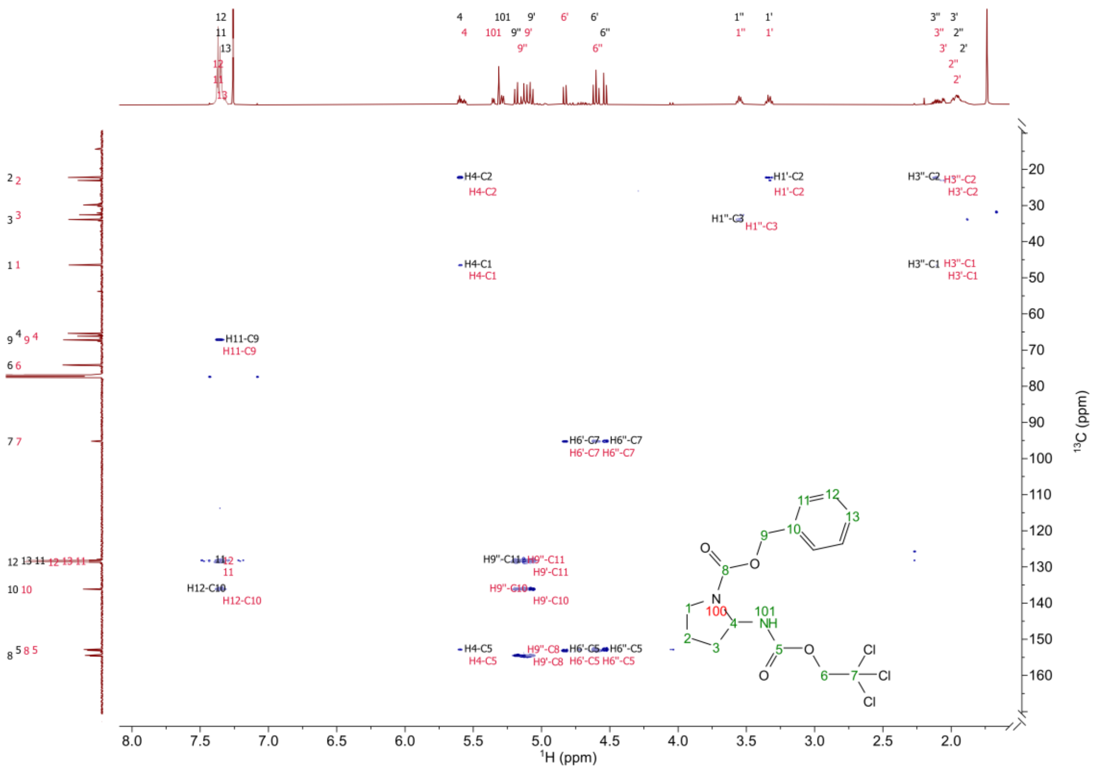

Compound **7b**: ^{1}H-^{1}H COSY (CDCl$_3$, 243 K)

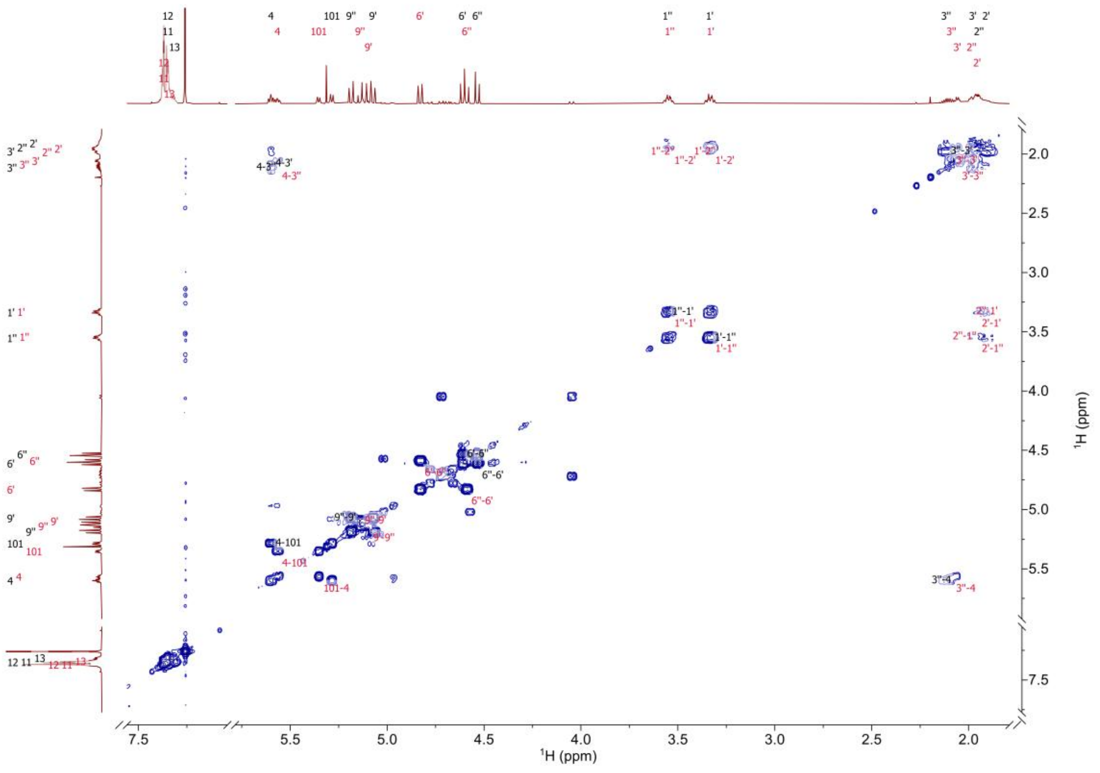

Compound **7b**: ^{1}H-^{1}H NOESY ($CDCl_3$, 243 K)

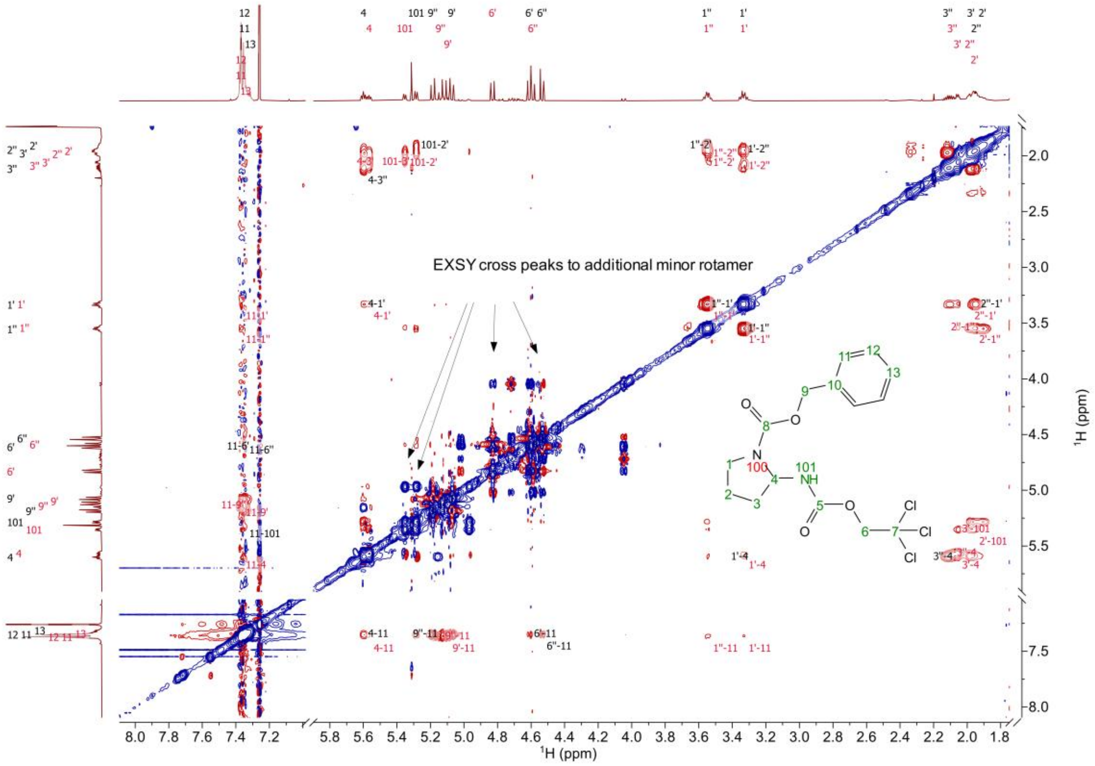

Compound **7b**: ^{1}H-^{15}N HMBC ($CDCl_3$, 243 K)

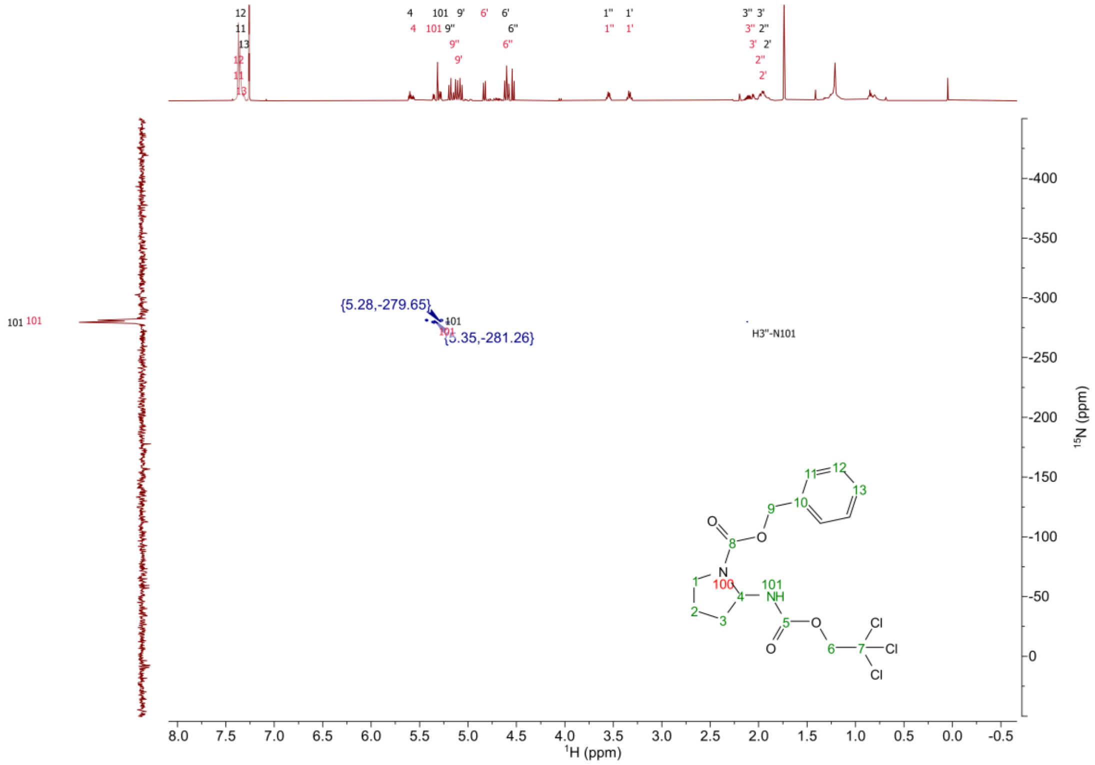

Compound **7b**: 1D selective TOCSY with excitation of H101

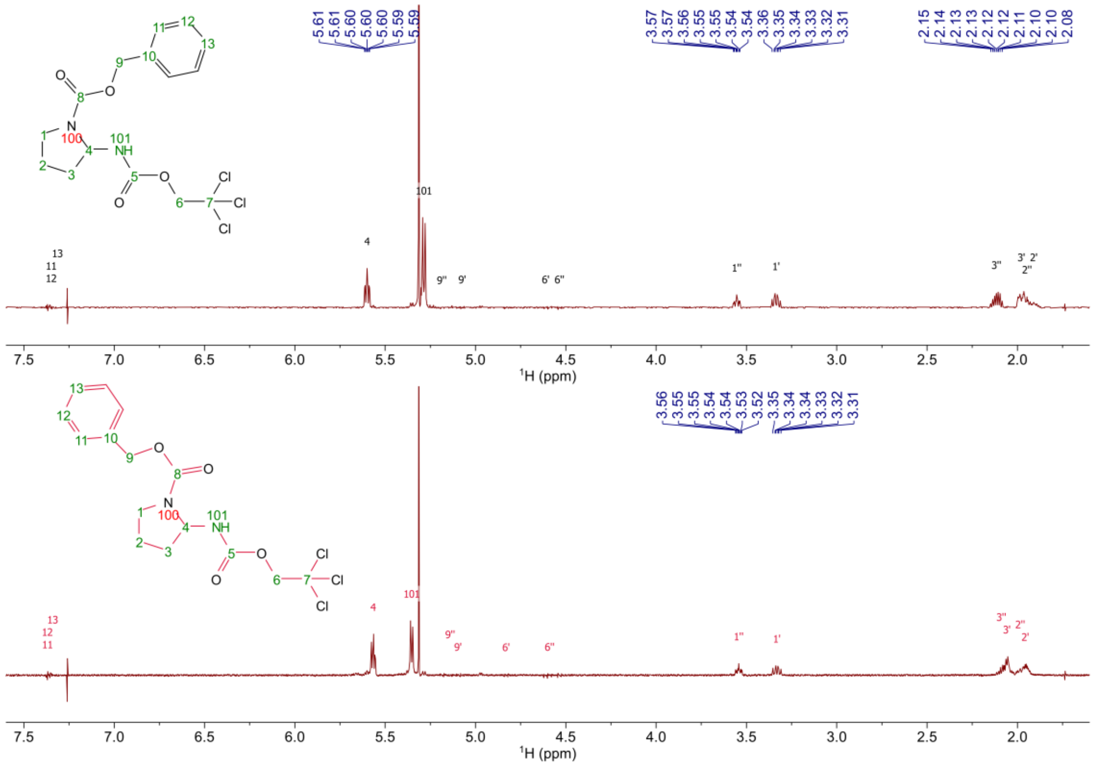

Compound **7c**: ^{1}H NMR (600 MHz, $CDCl_3$, 233 K)

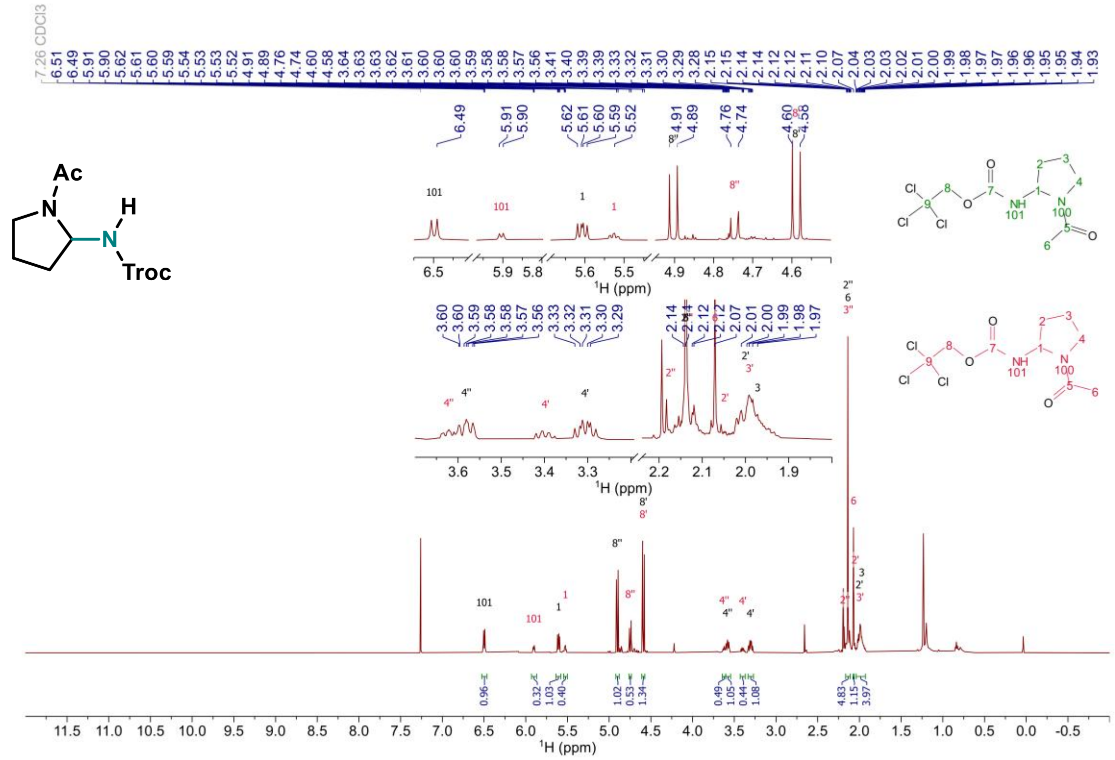

Compound **7c**: ^{13}C NMR (151 MHz, $CDCl_3$, 233 K)

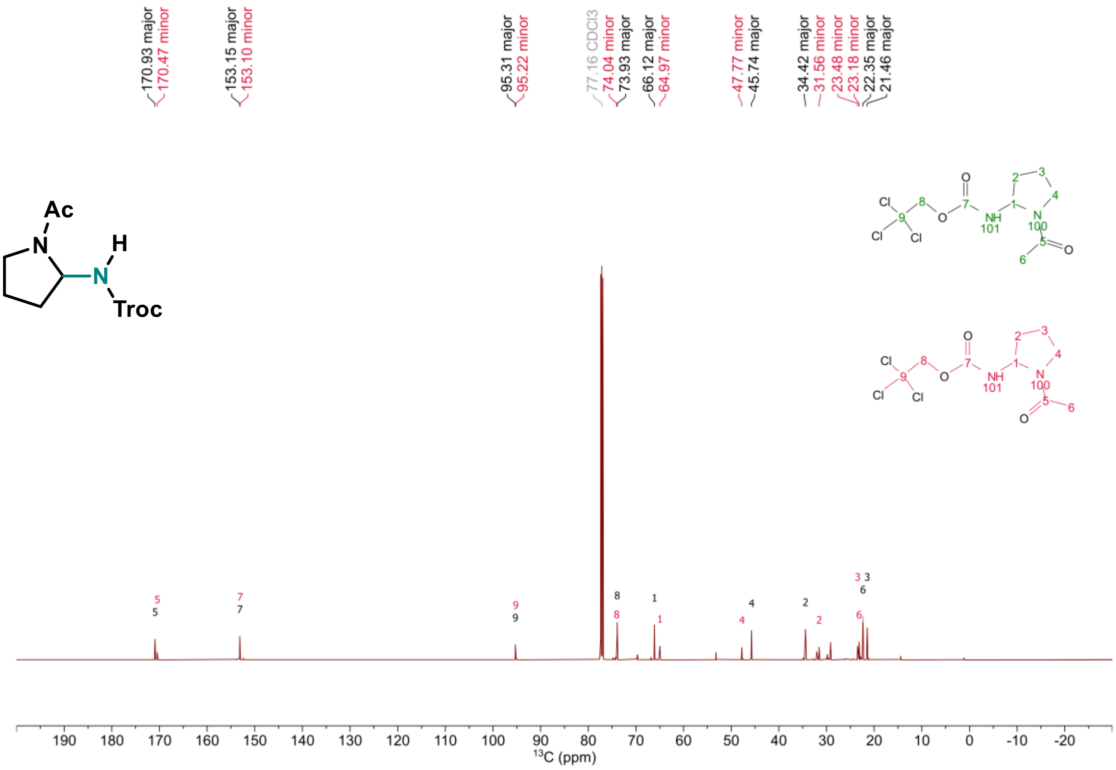

Compound **7c**: ^{1}H-^{13}C HSQC ($CDCl_3$, 233 K)

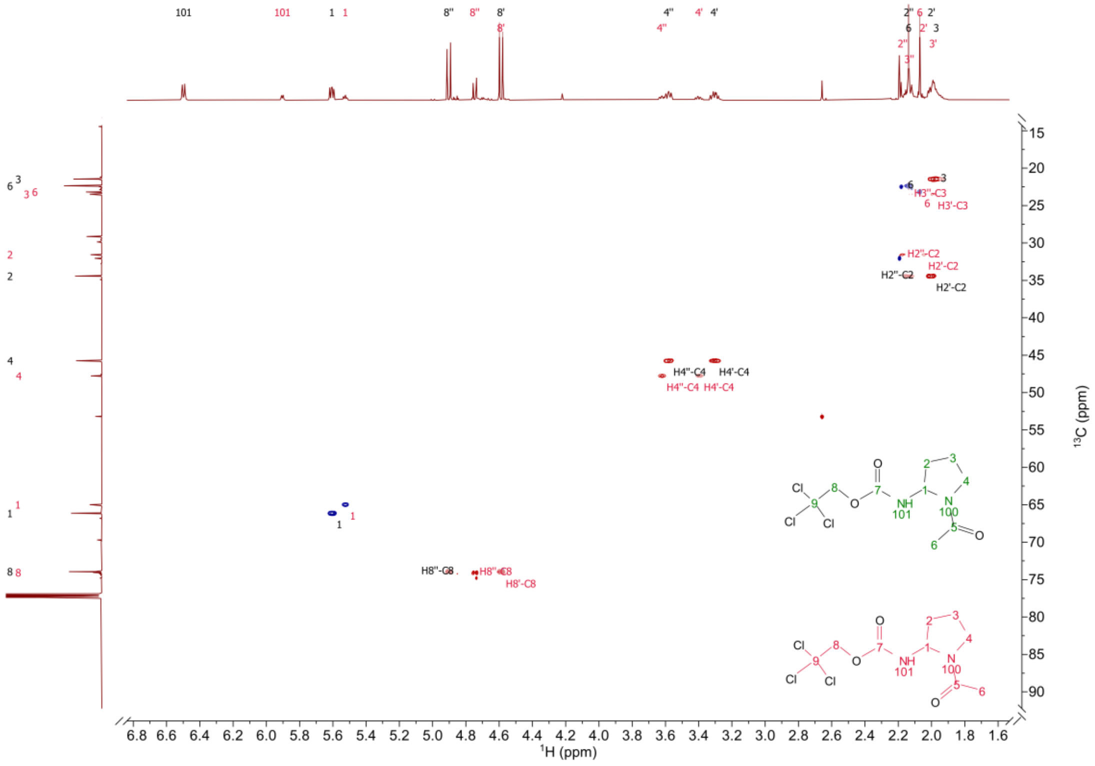

Compound **7c**: ^{1}H-^{13}C HMBC ($CDCl_3$, 233 K)

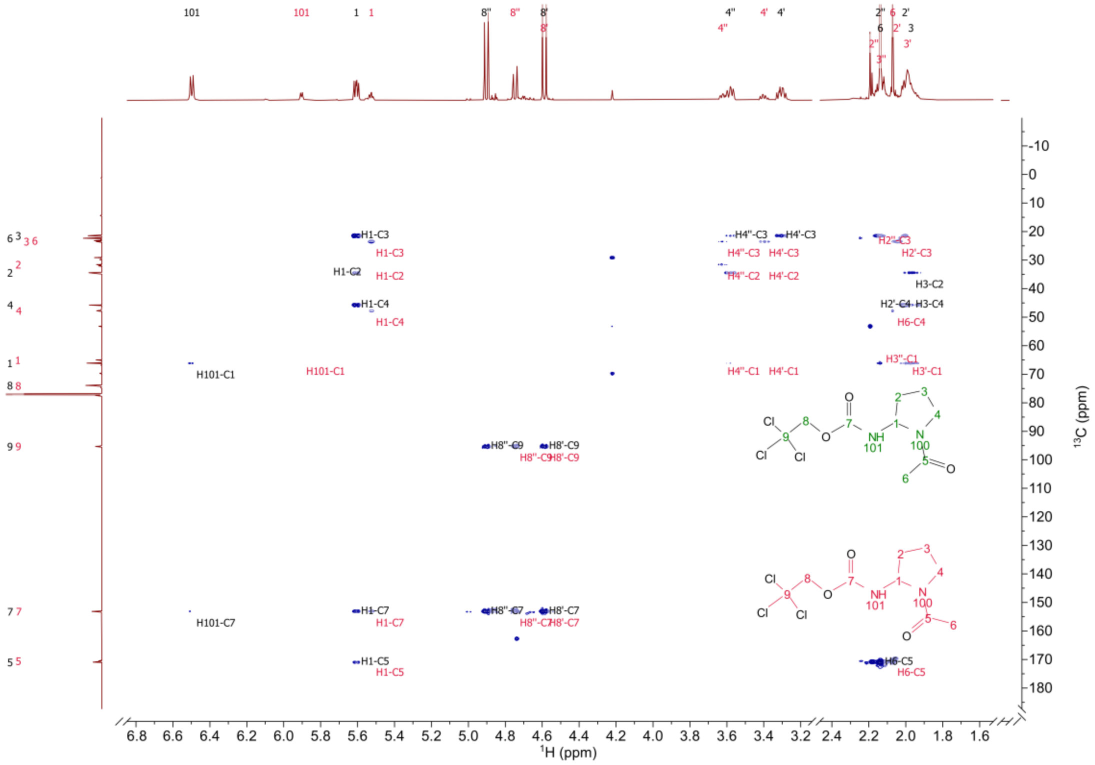

Compound **7c**: ^{1}H-^{1}H COSY ($CDCl_3$, 233 K)

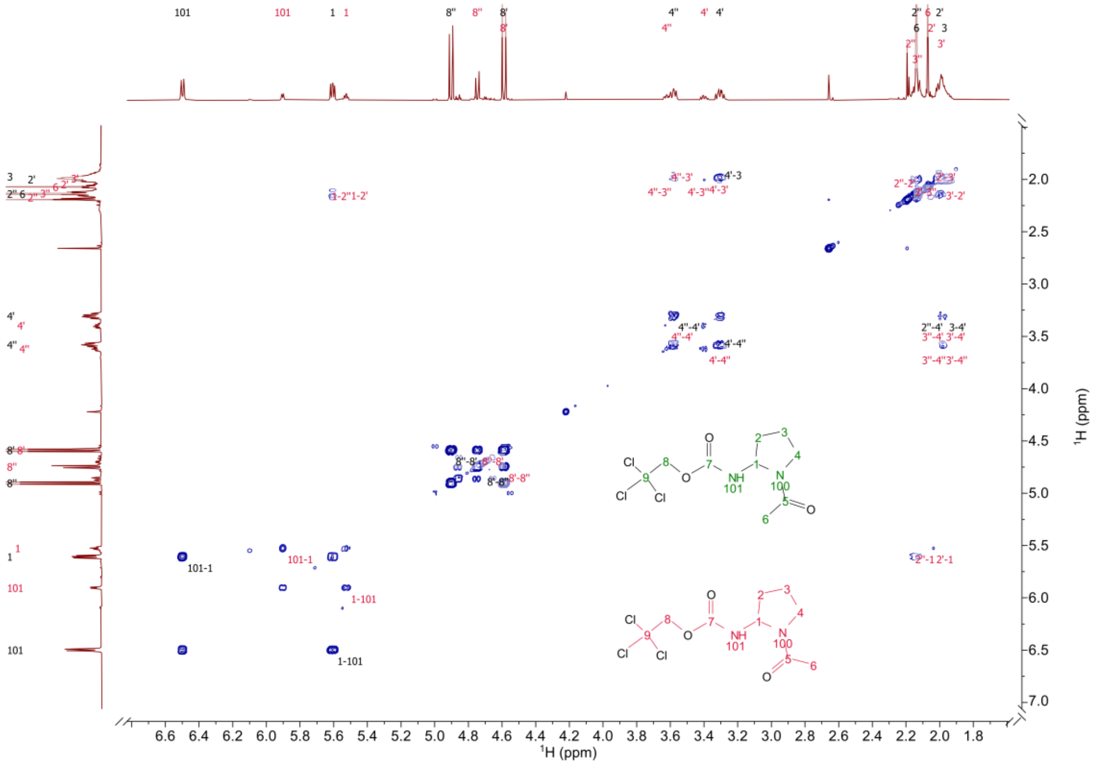

Compound **7c**: ^{1}H-^{1}H NOESY ($CDCl_3$, 233 K)

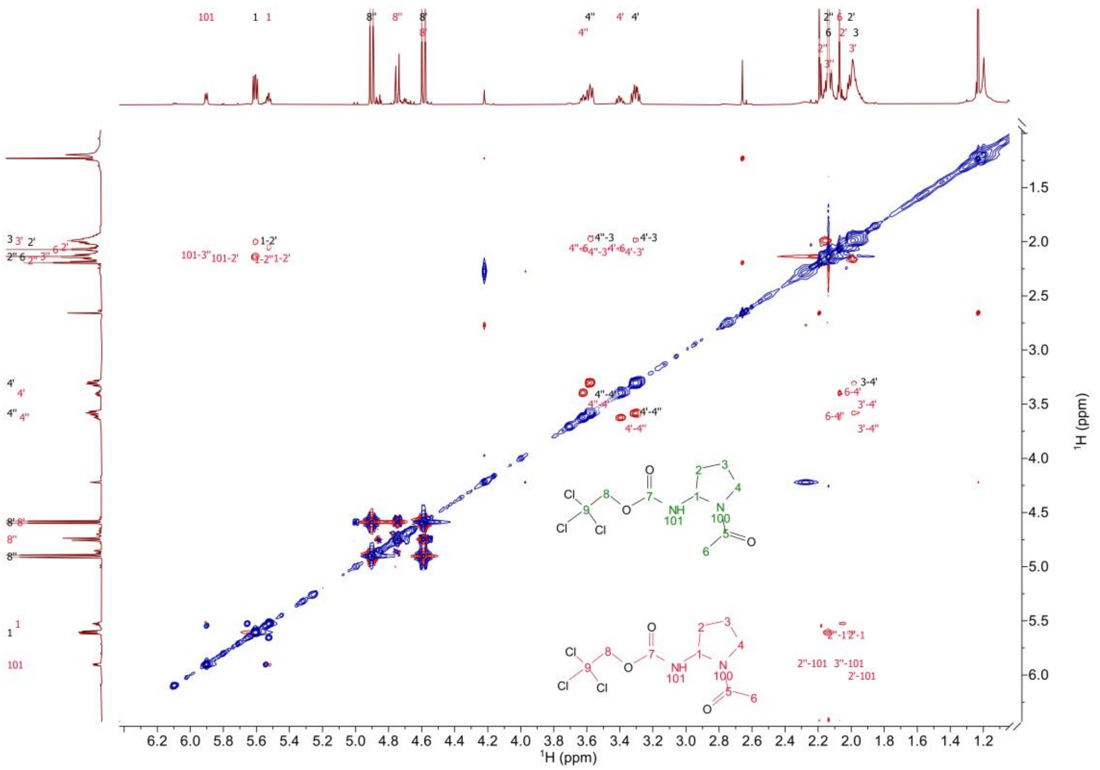

Compound **7c**: ^{1}H-^{15}N HMBC ($CDCl_3$, 233 K)

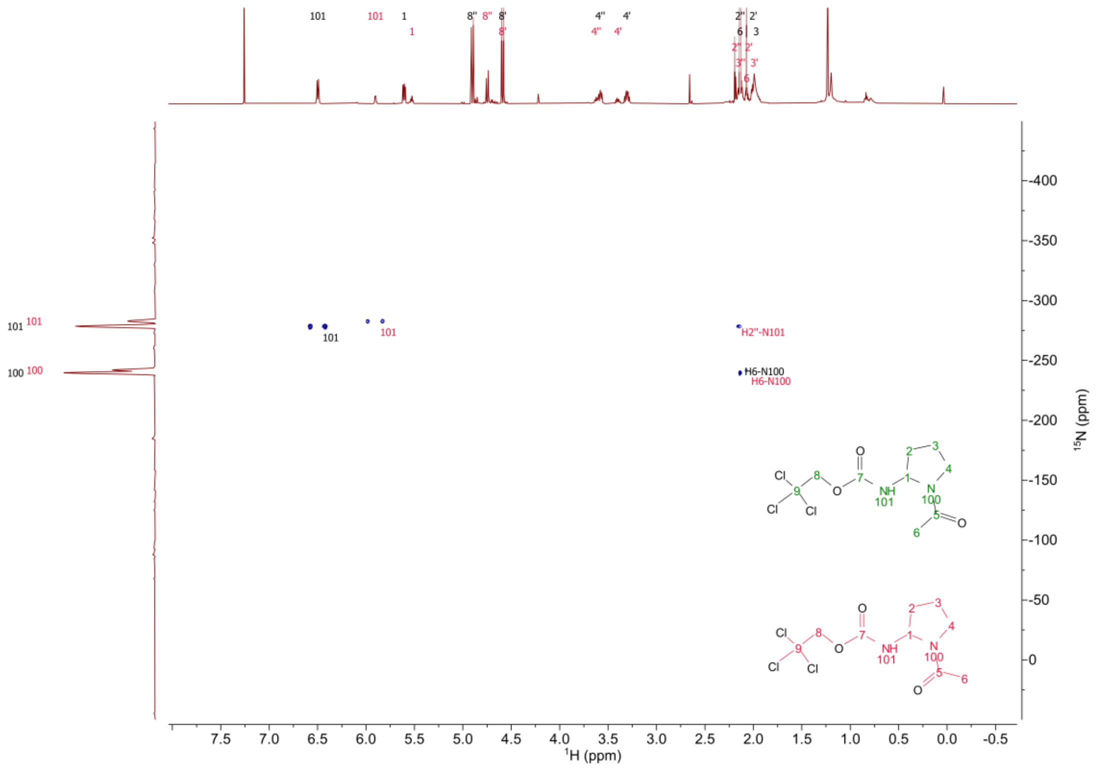

Compound **7c**: 1D selective TOCSY with excitation of H101

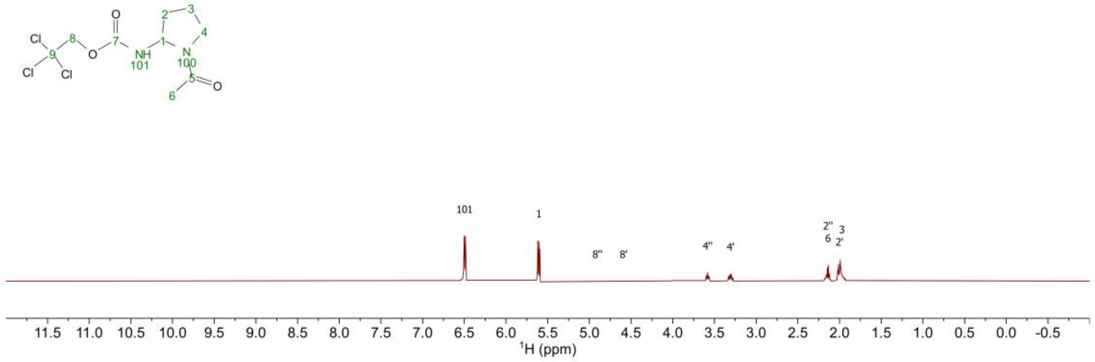

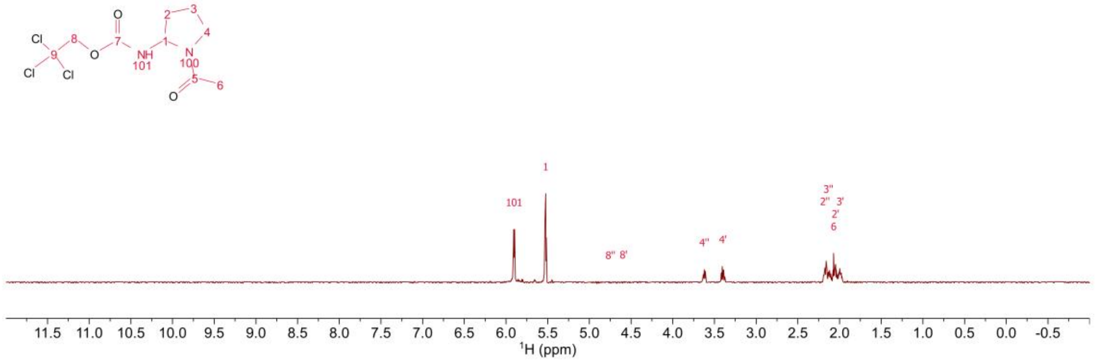

Compound **7d**: ^{1}H NMR (600 MHz, $CDCl_3$, 233 K)

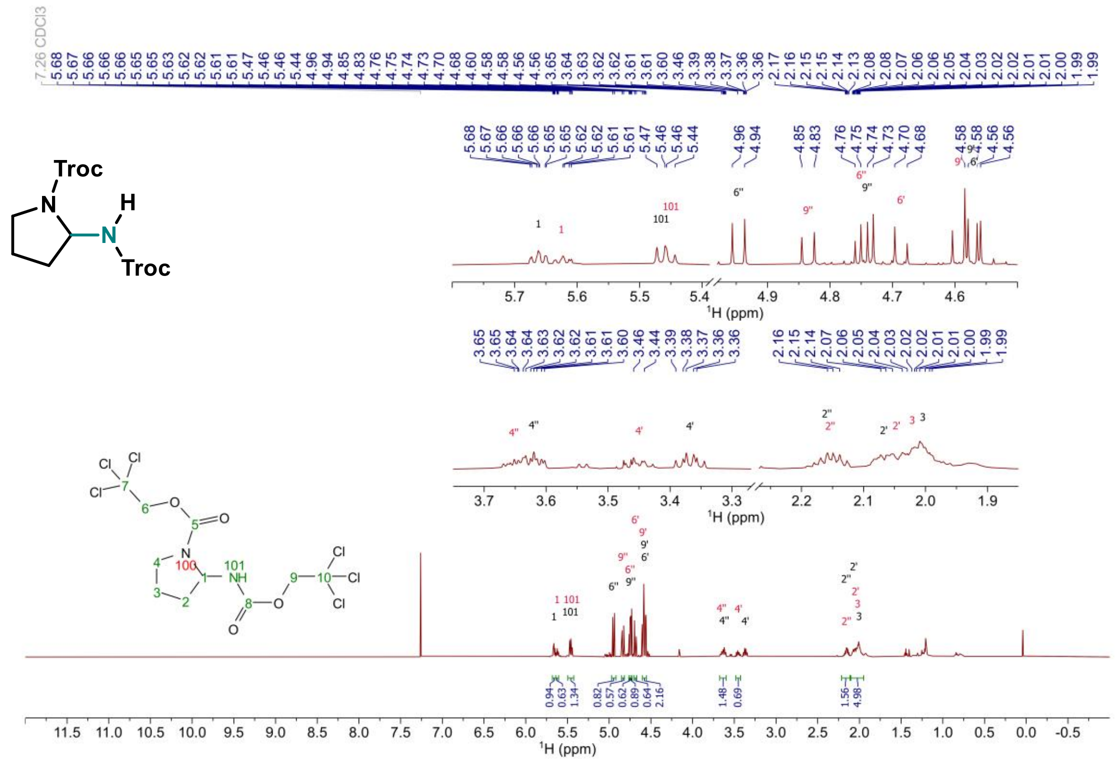

Compound **7d**: ^{13}C NMR (151 MHz, $CDCl_3$, 233 K)

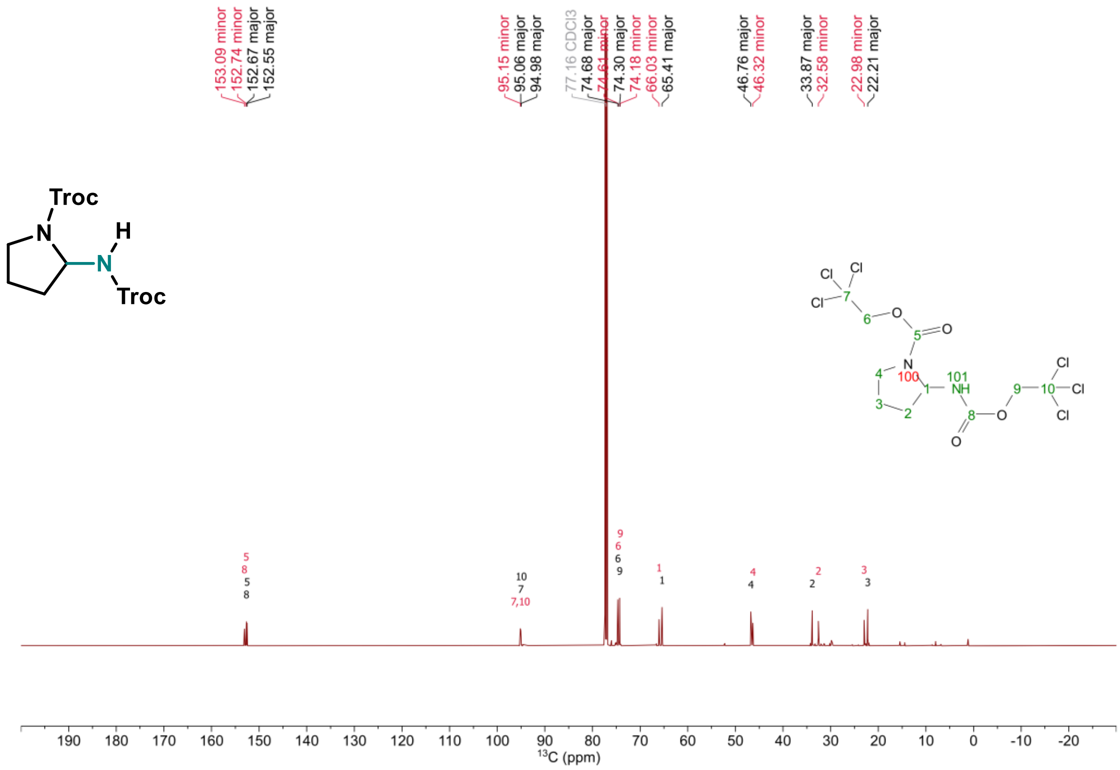

Compound **7d**: variable temperature ^{1}H NMR (600 MHz, $CDCl_3$)

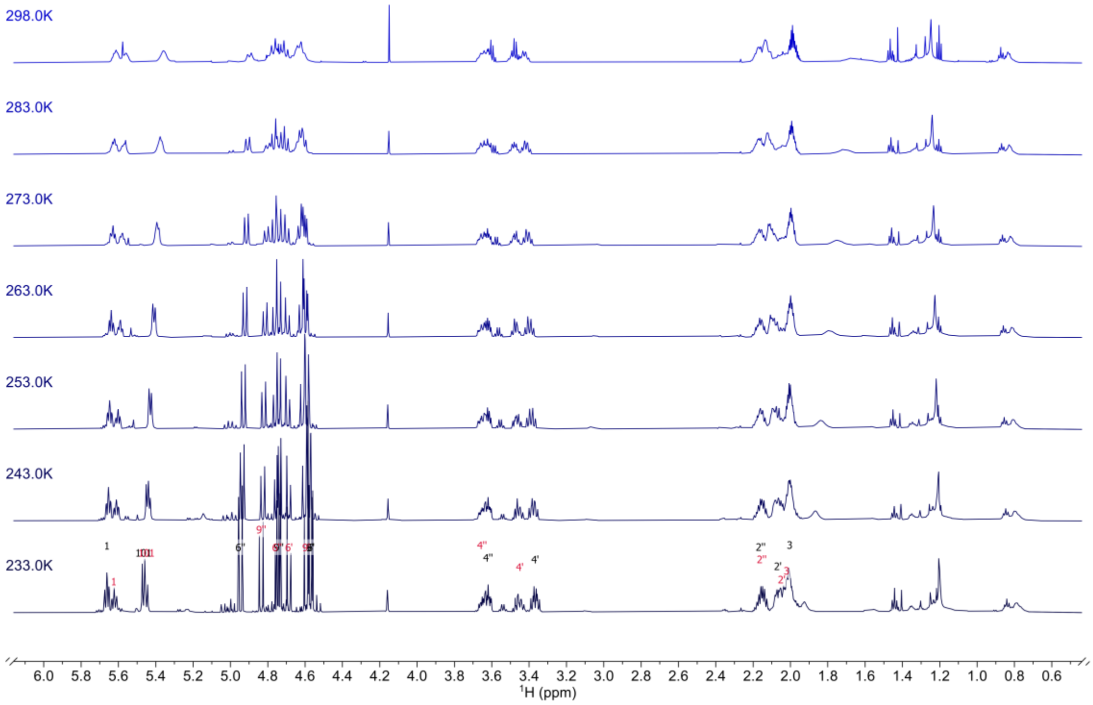

Compound **7d**: ^{1}H-^{13}C HSQC ($CDCl_3$, 233 K)

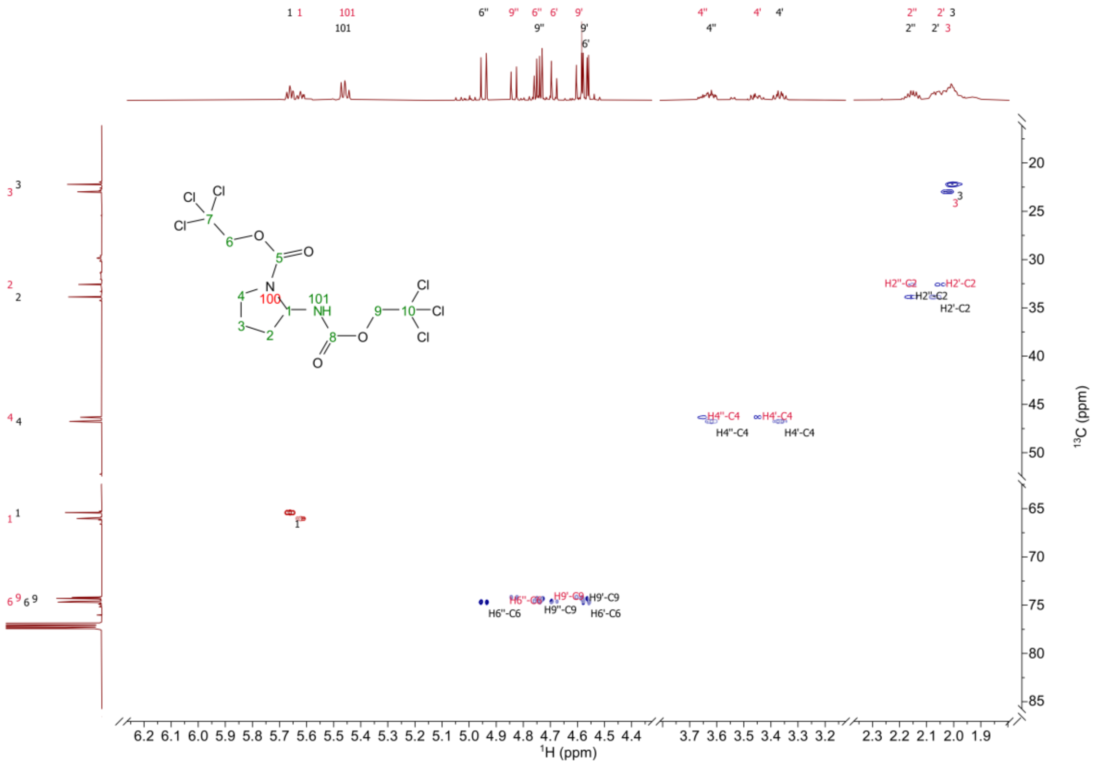

Compound **7d**: ^{1}H-^{13}C HMBC ($CDCl_3$, 233 K)

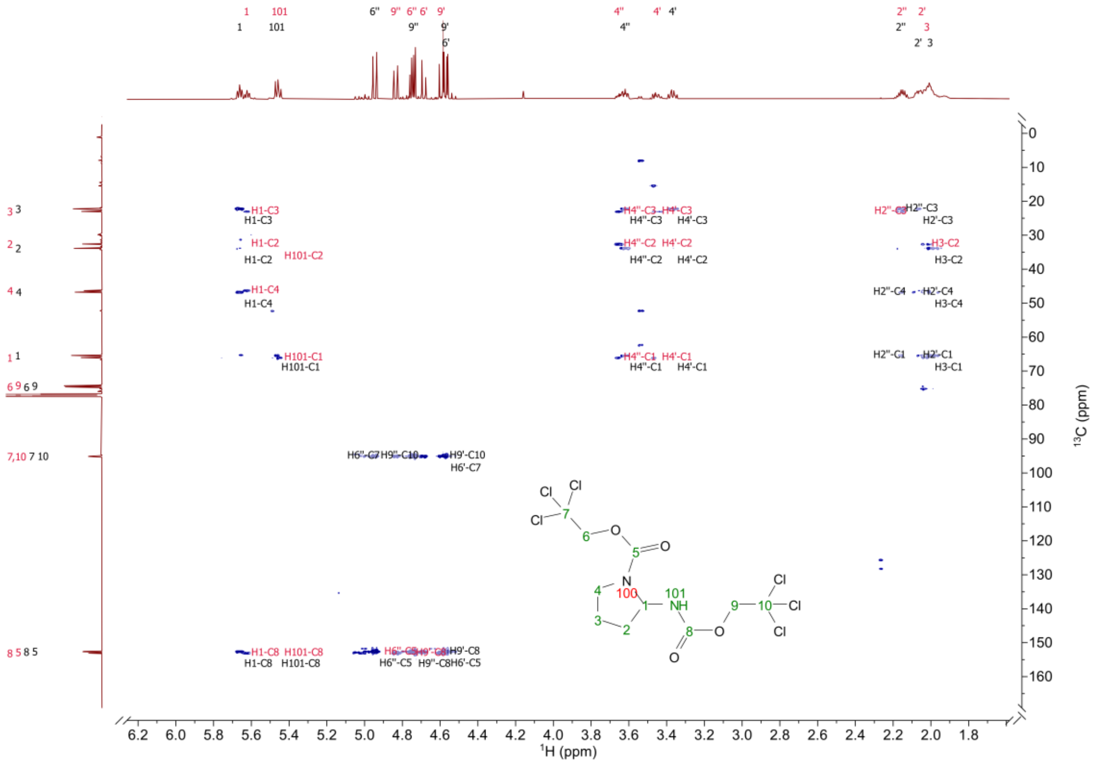

Compound **7d**: ^{1}H-^{1}H COSY ($CDCl_3$, 233 K)

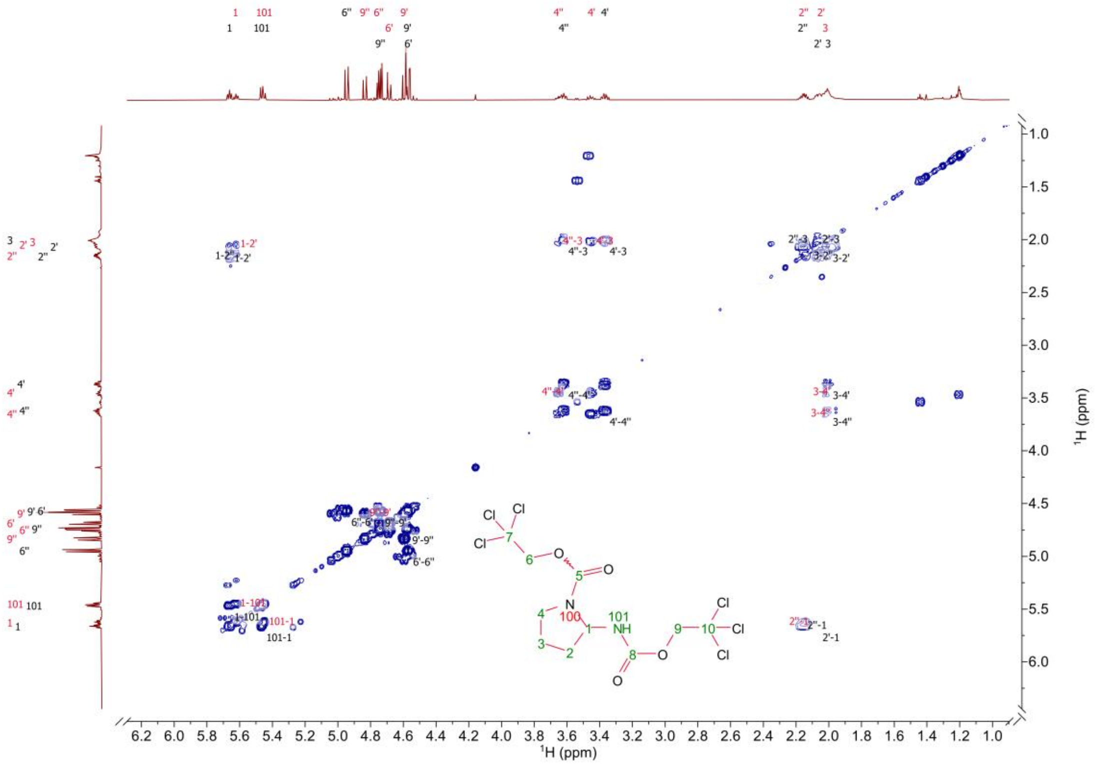

Compound **7d**: ^{1}H-^{1}H NOESY ($CDCl_3$, 233 K)

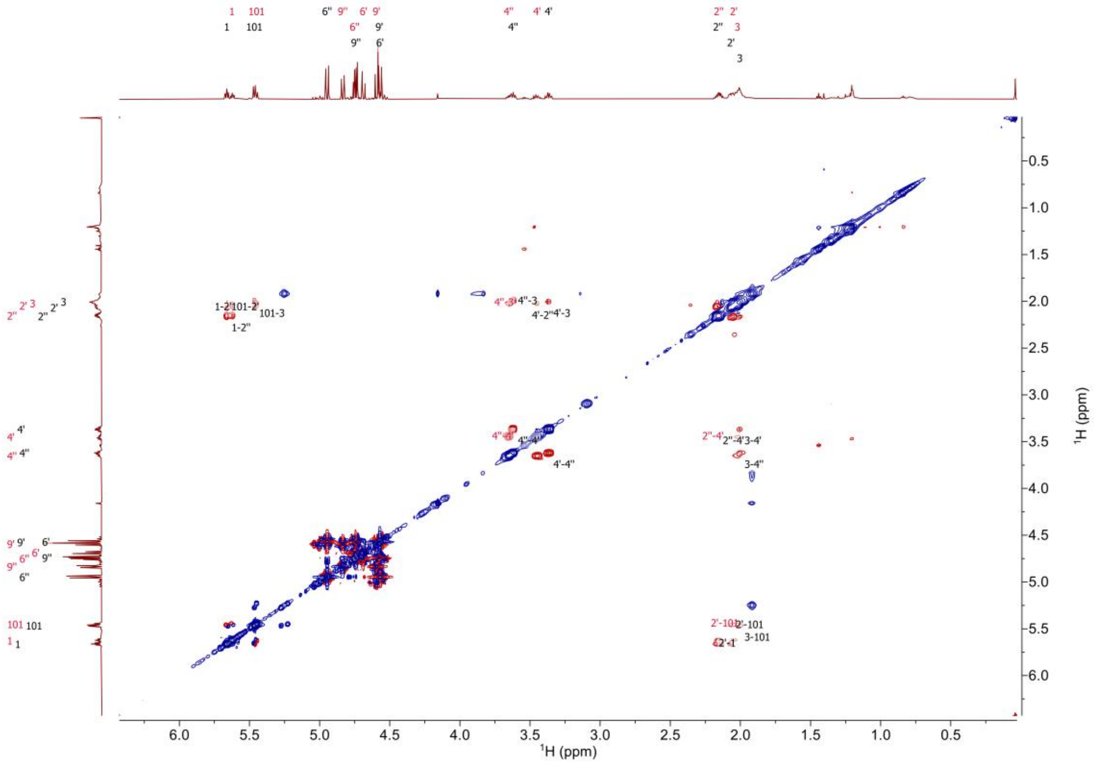

Compound **7d**: ^{1}H-^{15}N HMBC ($CDCl_3$, 233 K)

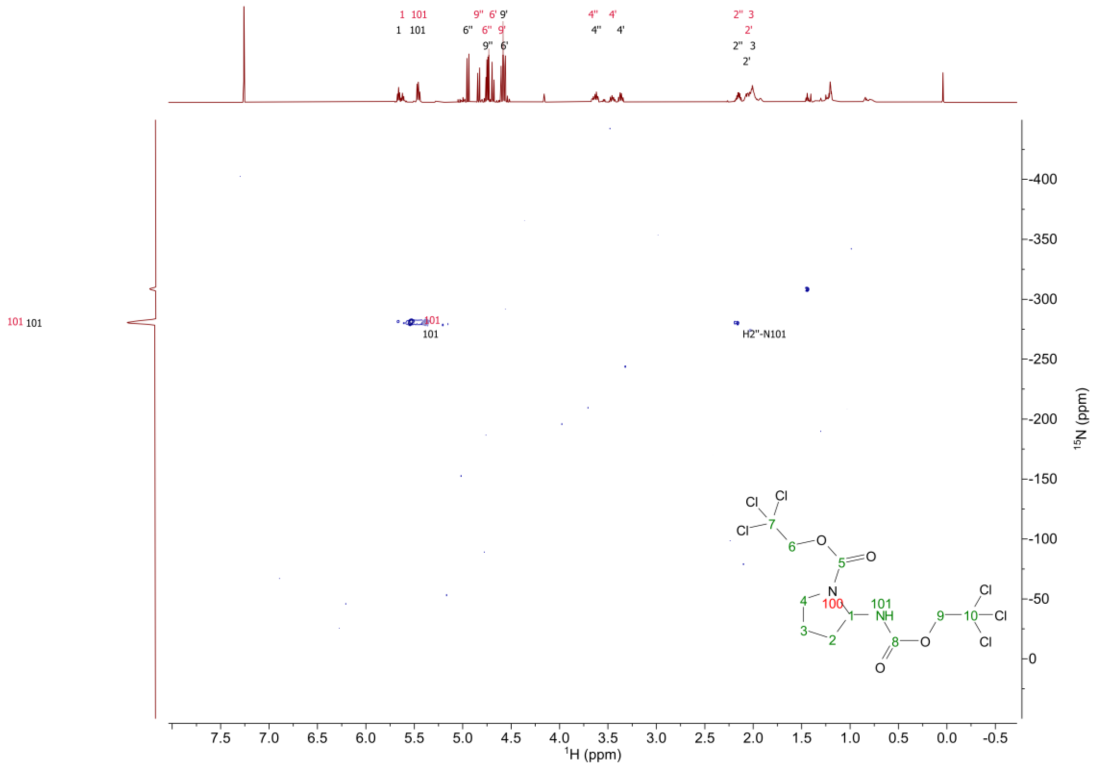

Compound **7d**: 1D selective TOCSY with excitation of H1

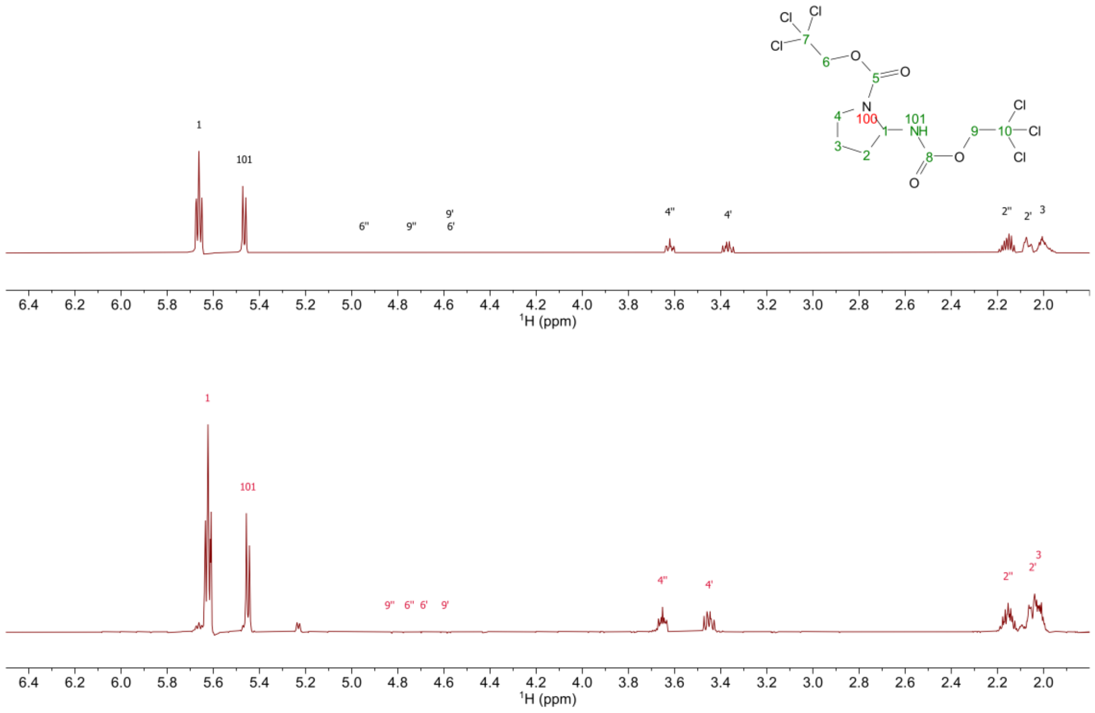

Compound **7e**: ^{1}H NMR (600 MHz, $CDCl_3$, 333 K)

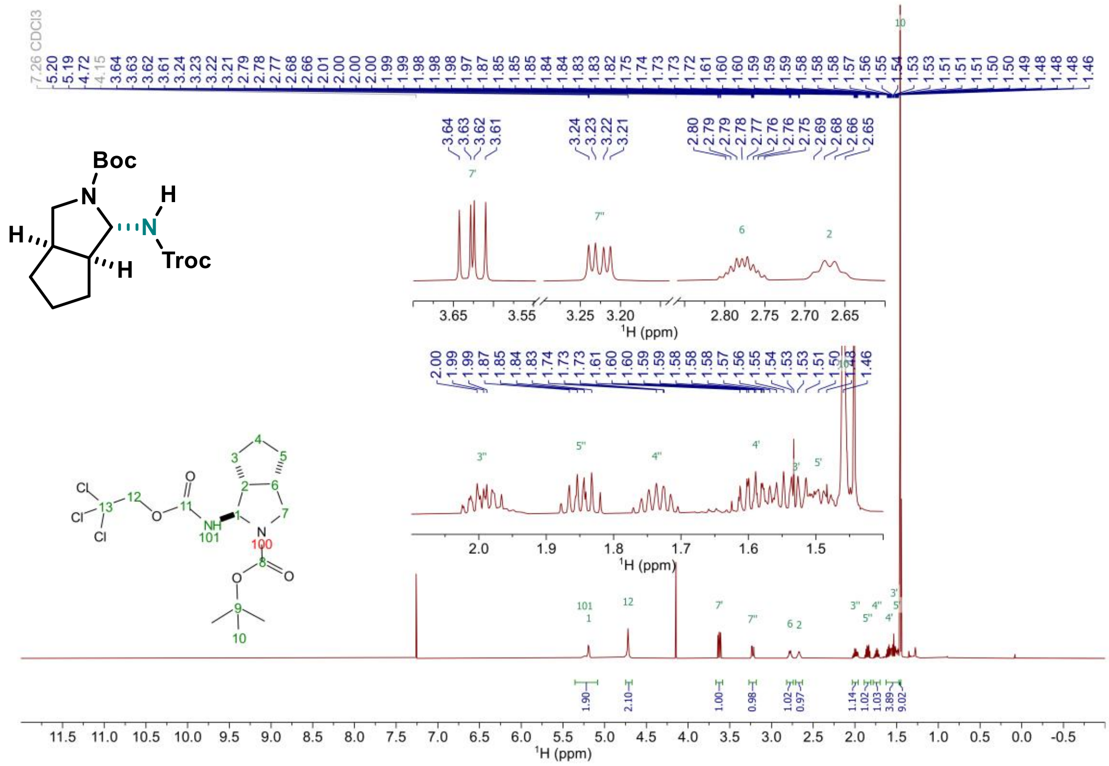

Compound **7e**: ^{13}C NMR (151 MHz, $CDCl_3$, 333 K)

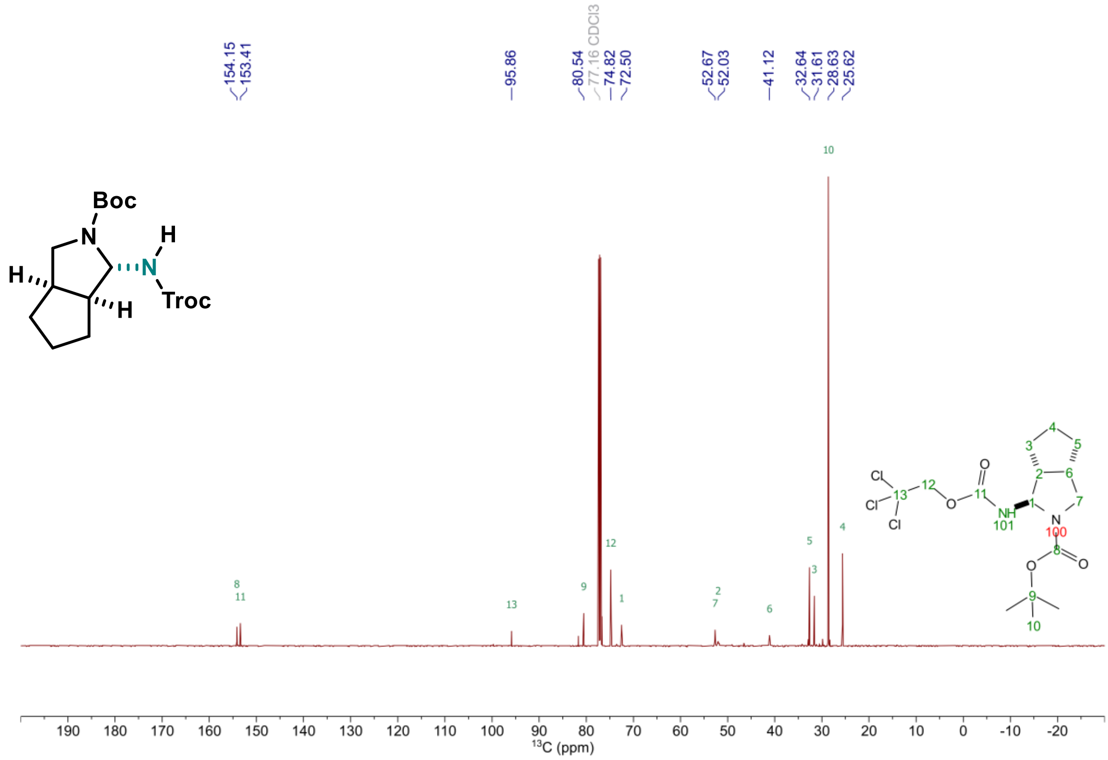

Compound **7e**: variable temperature ^{1}H NMR (600 MHz, $CDCl_3$)

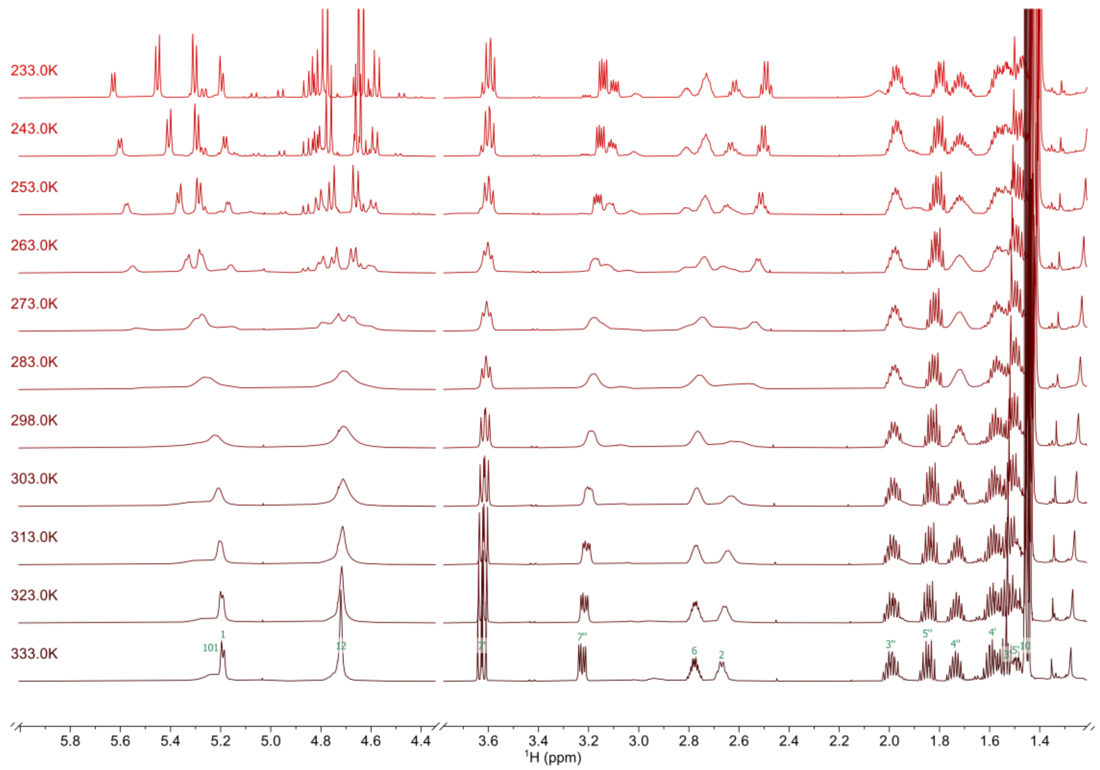

Compound **7e**: ^{1}H-^{13}C HSQC ($CDCl_3$, 333 K)

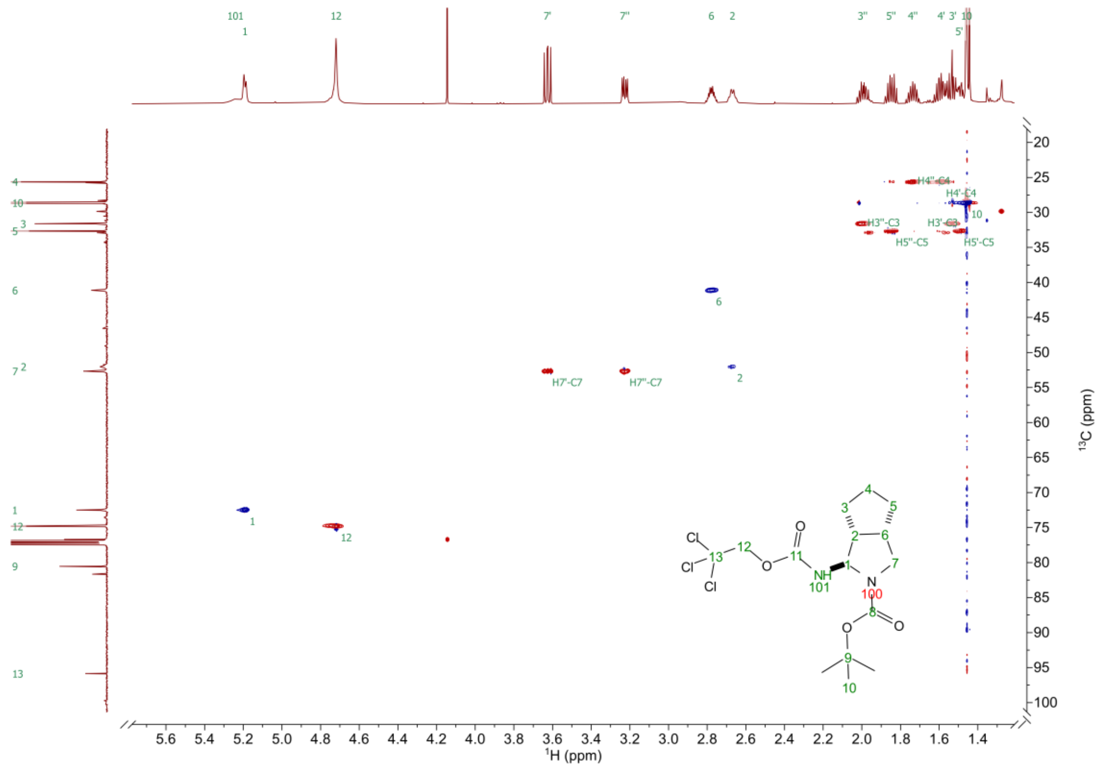

Compound **7e**: ^{1}H-^{13}C HMBC ($CDCl_3$, 333 K)

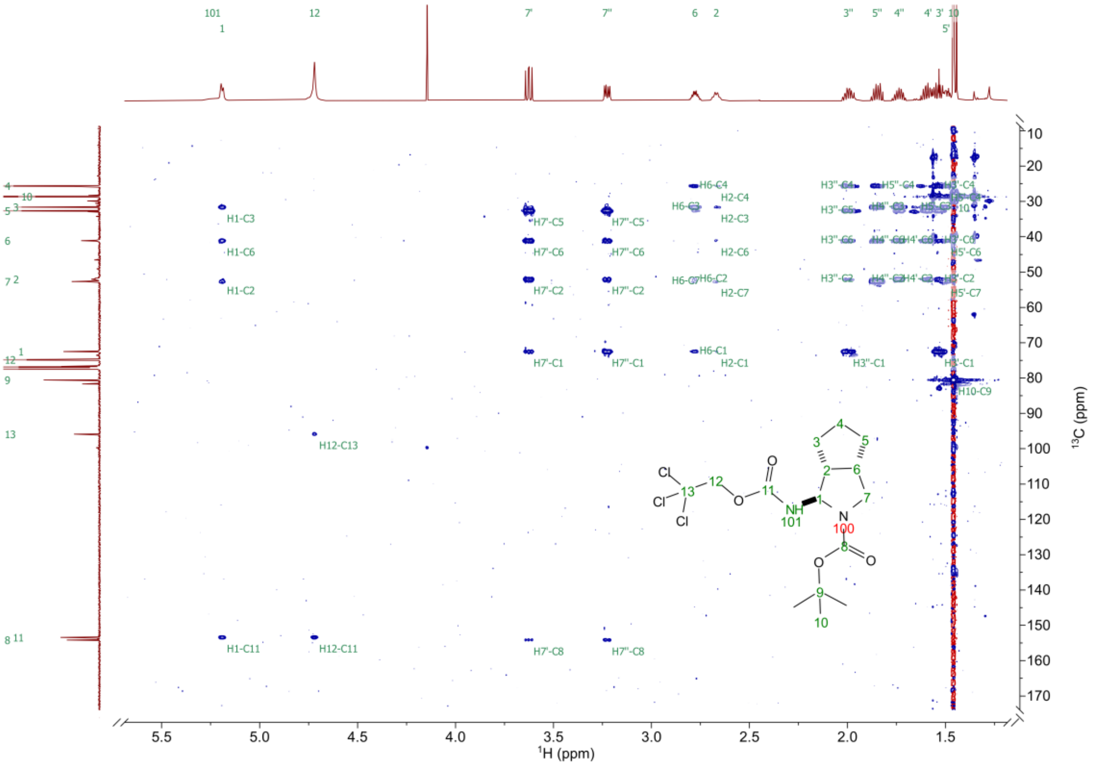

Compound **7e**: ^{1}H-^{1}H COSY ($CDCl_3$, 333 K)

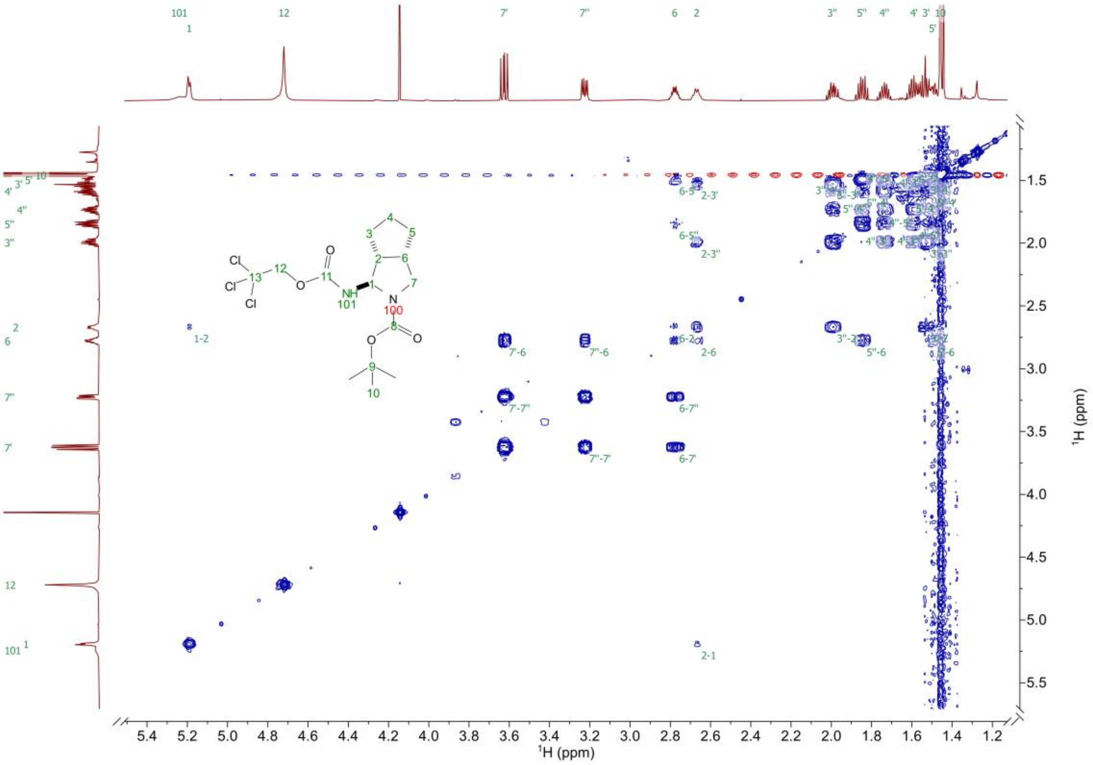

Compound **7e**: ^{1}H-^{1}H NOESY ($CDCl_3$, 333 K)

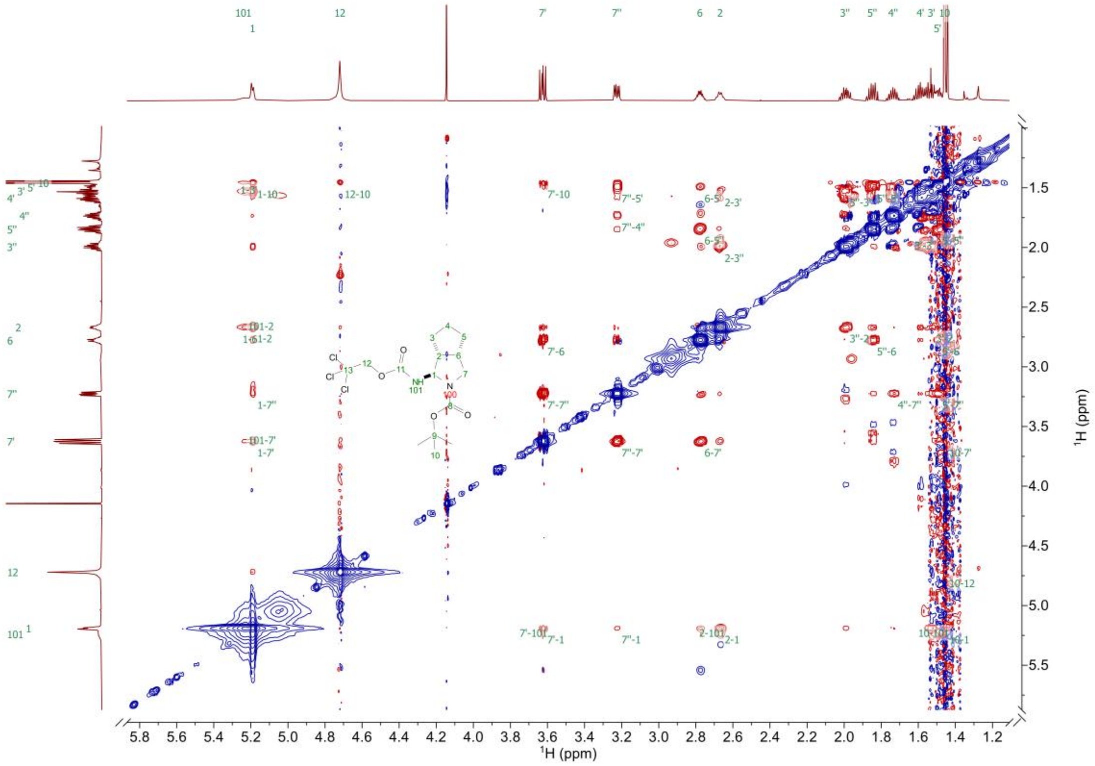

Compound **7e**: ^{1}H-^{1}H NOESY ($CDCl_3$, 233 K)

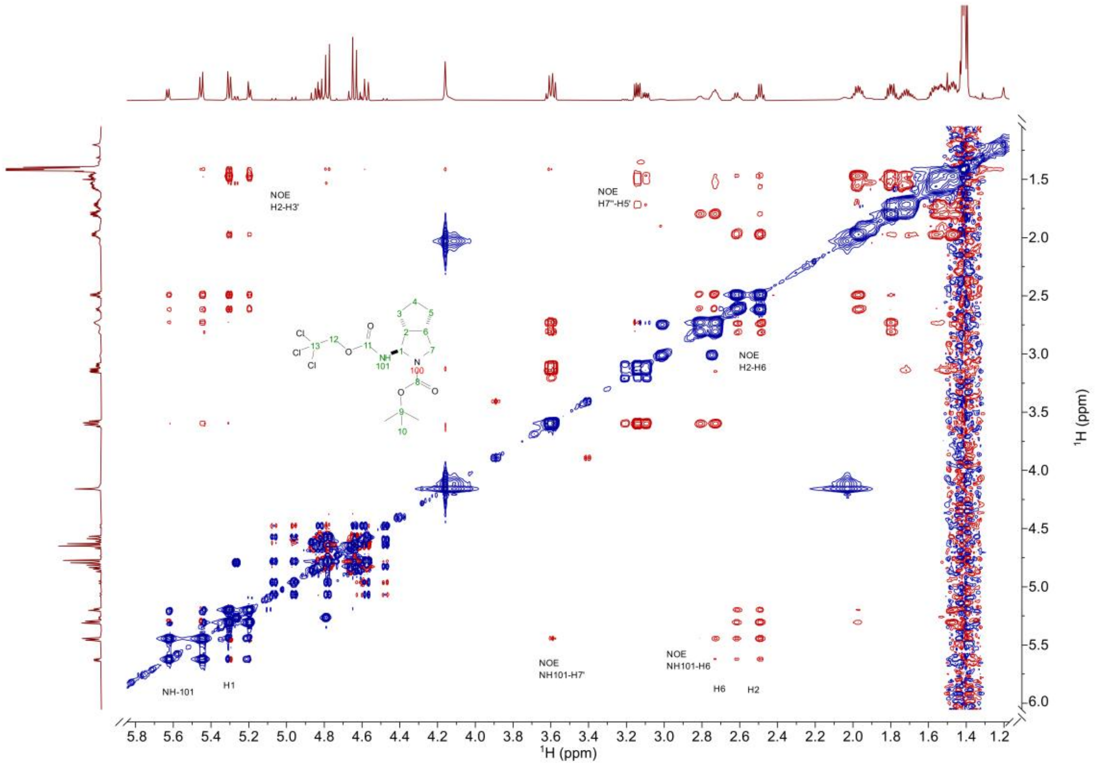

Compound **7f**: ^{1}H NMR (600 MHz, DMSO-d_6, 383 K)

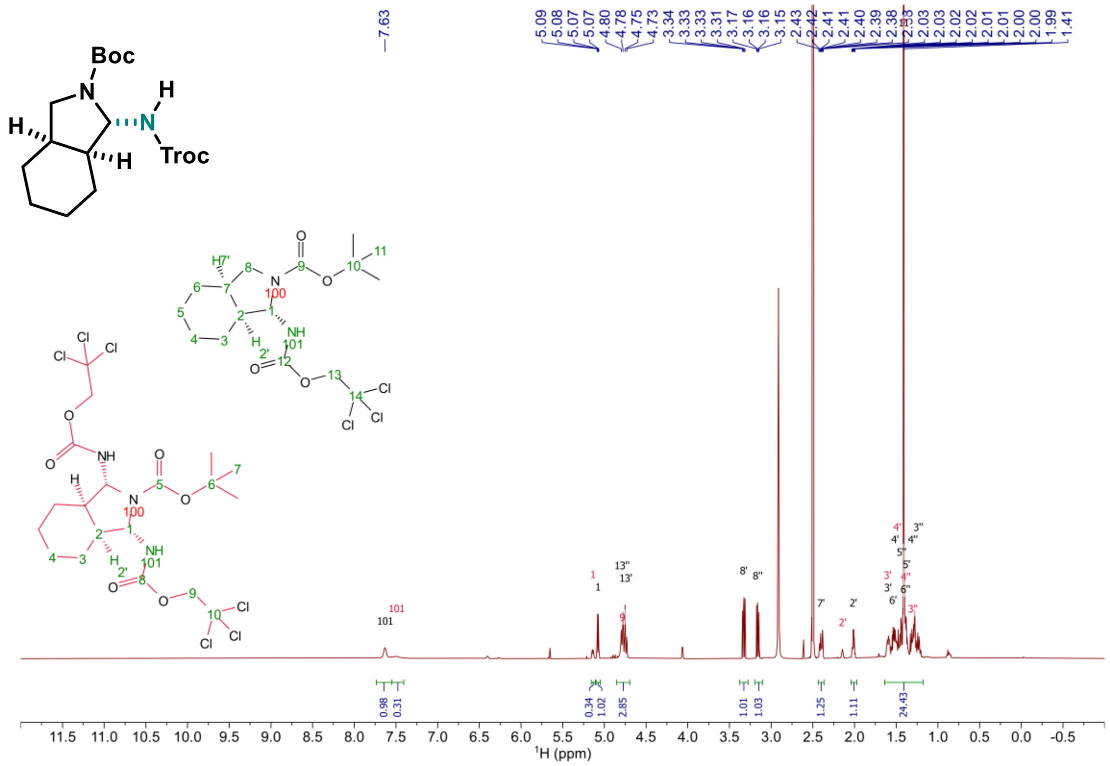

Compound **7f**: ^{13}C NMR (151 MHz, DMSO-d_6, 383 K)

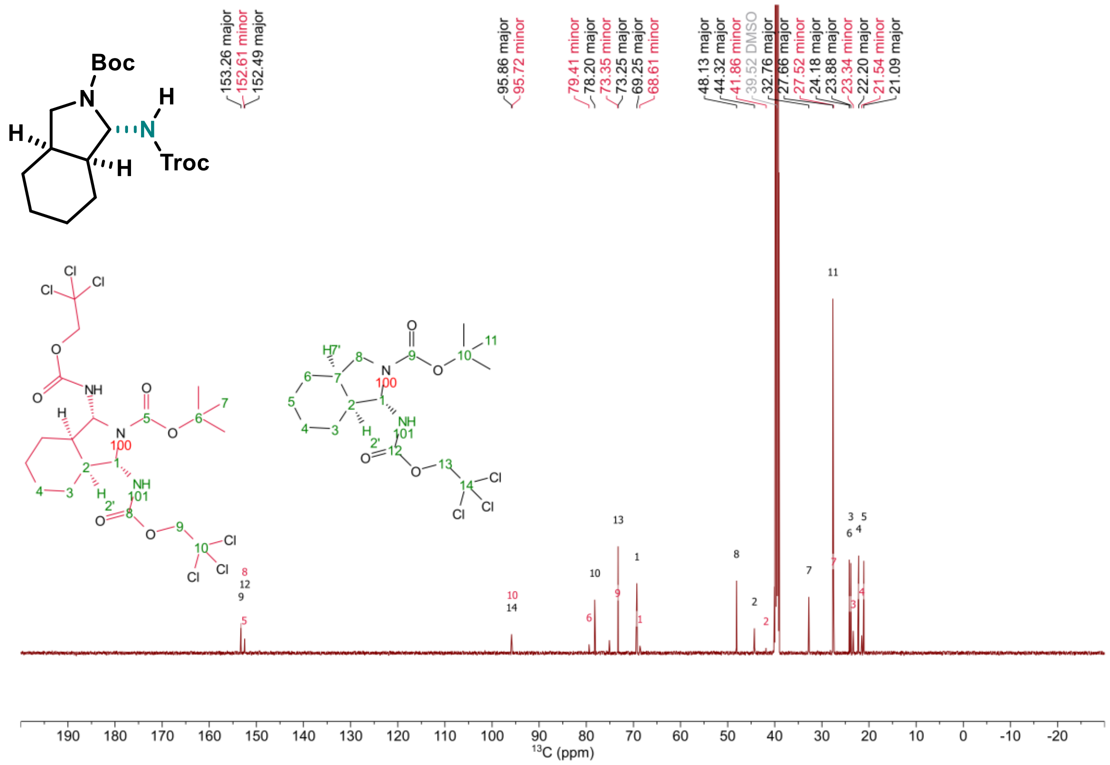

Compound **7f**: variable temperature ^{1}H NMR (600 MHz, DMSO-d_6)

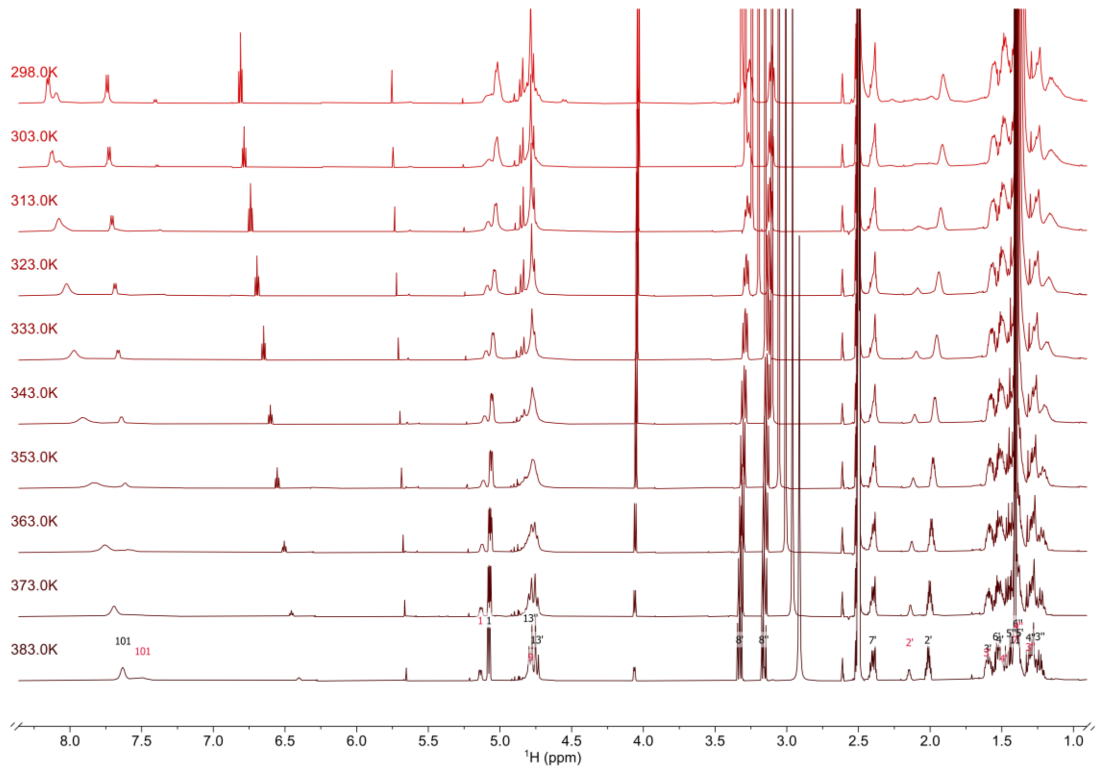

Compound **7f**: ^{1}H-^{13}C HSQC (DMSO-d_6, 383 K)

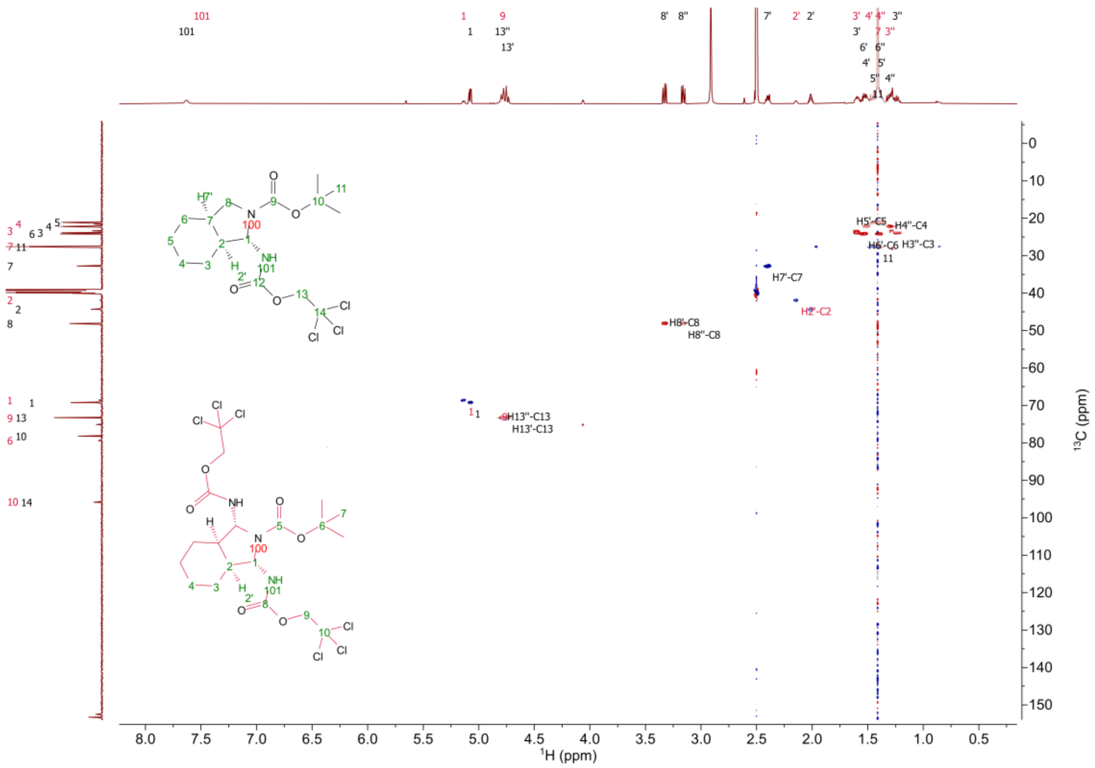

Compound **7f**: ^{1}H-^{13}C HMBC (DMSO-d_6, 383 K)

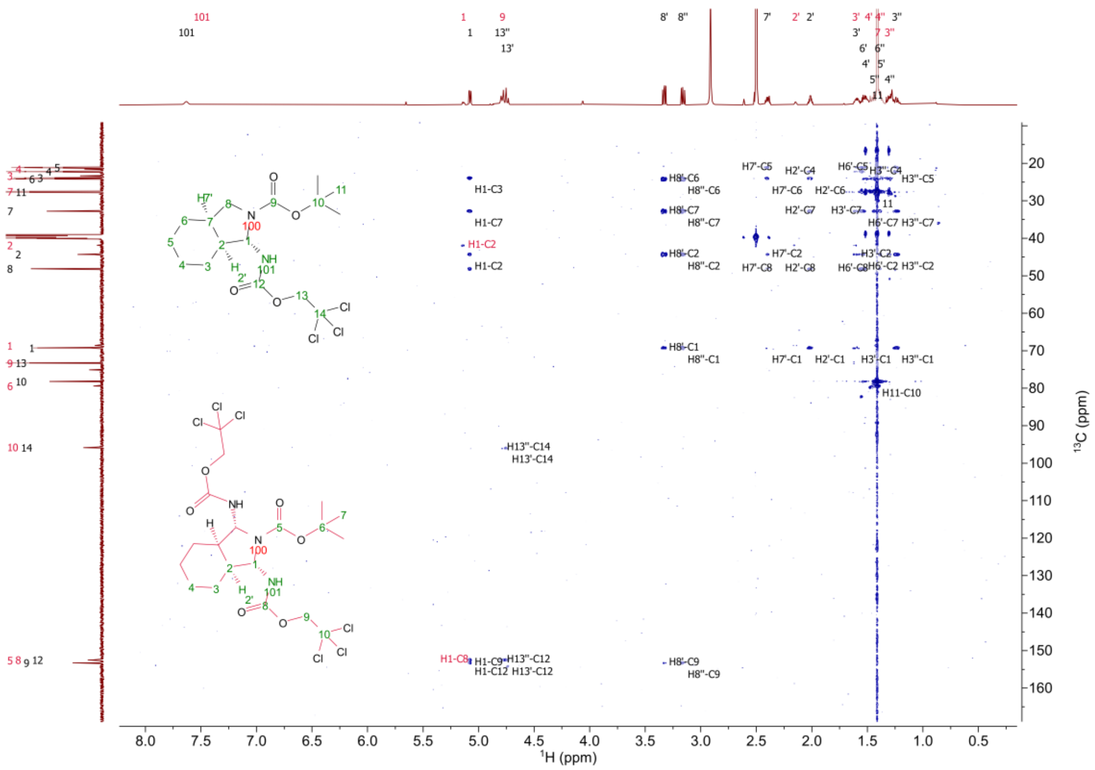

Compound **7f**: ^{1}H-^{1}H COSY (DMSO-d_6, 383 K)

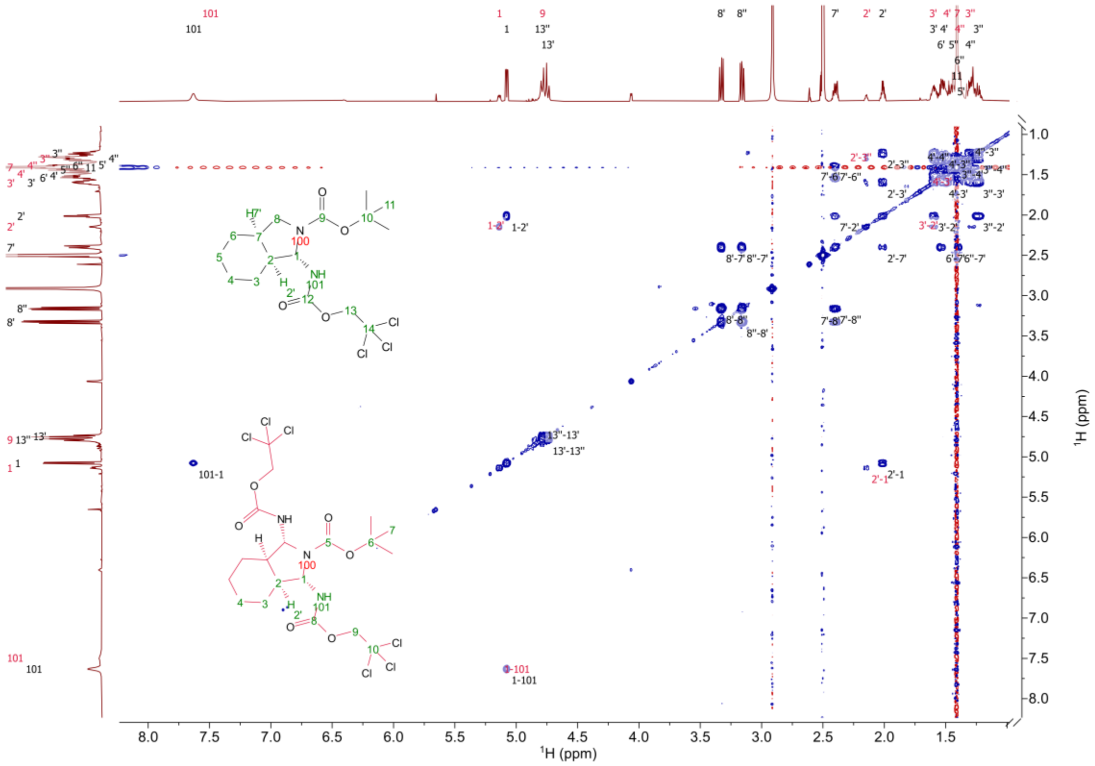

Compound **7f**: ^{1}H-^{1}H NOESY (DMSO-d_6, 383 K)

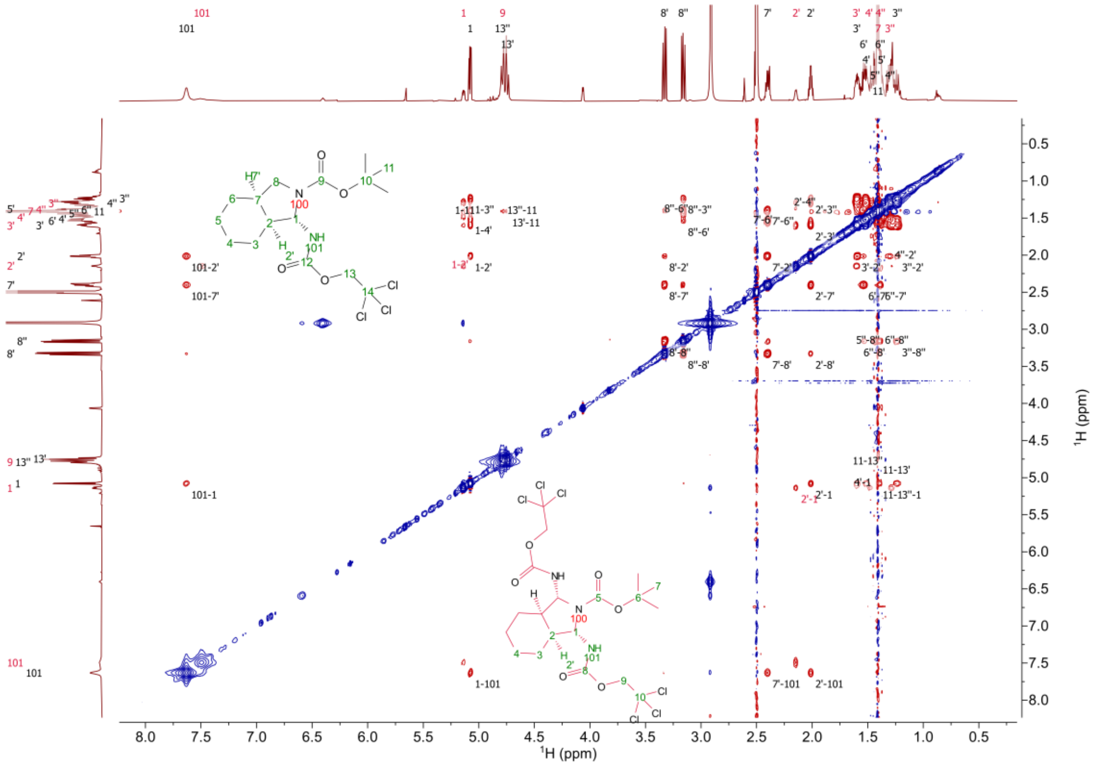

Compound **7g**: ^{1}H NMR (600 MHz, $CDCl_3$, 298 K)

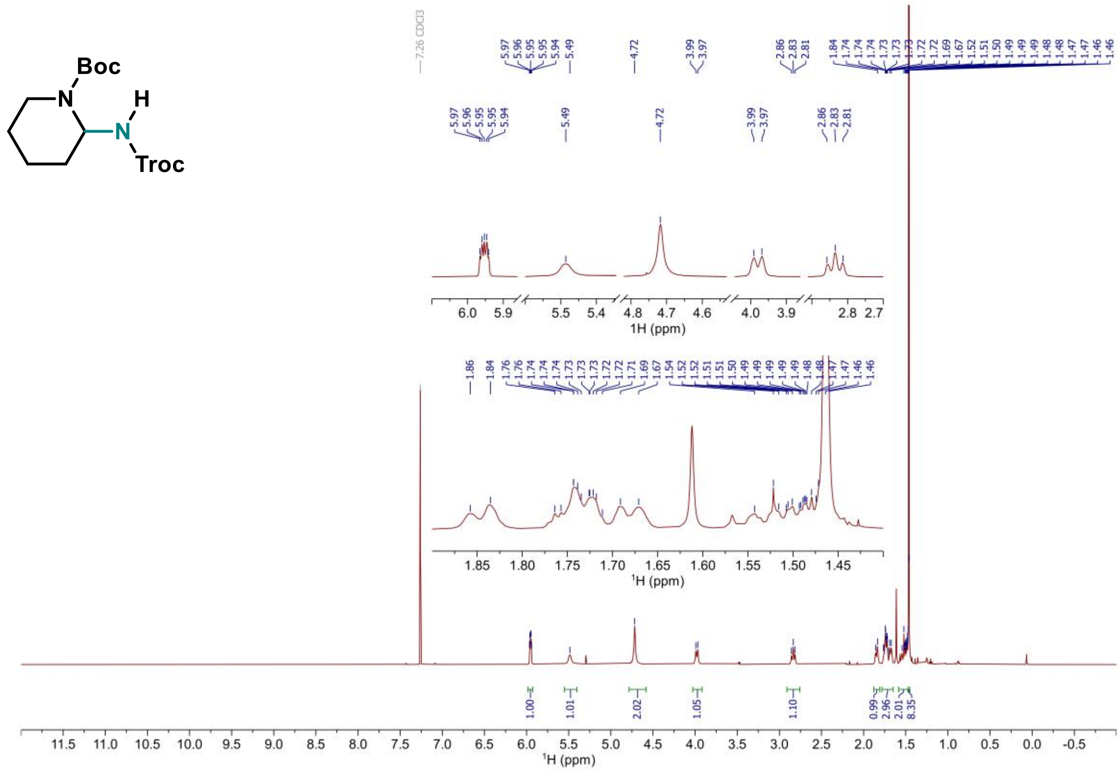

Compound **7g**: ^{13}C NMR (151 MHz, $CDCl_3$, 298 K)

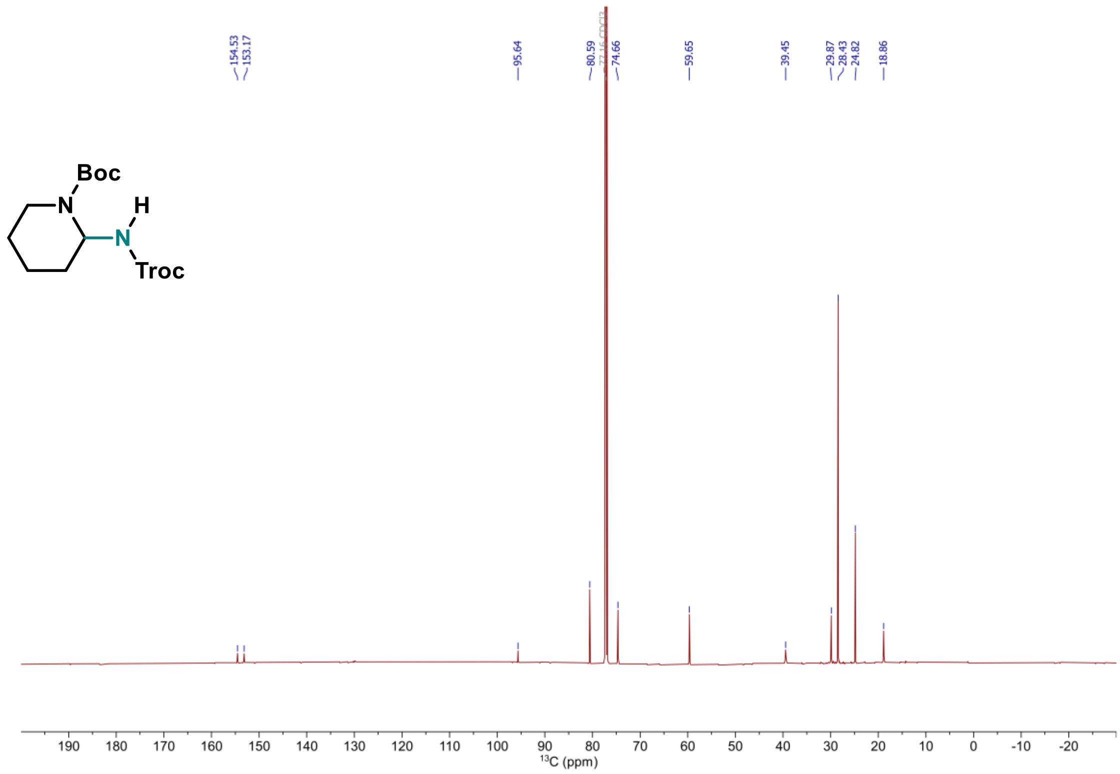

Compound **7h**: ^{1}H NMR (600 MHz, $CDCl_3$, 323 K)

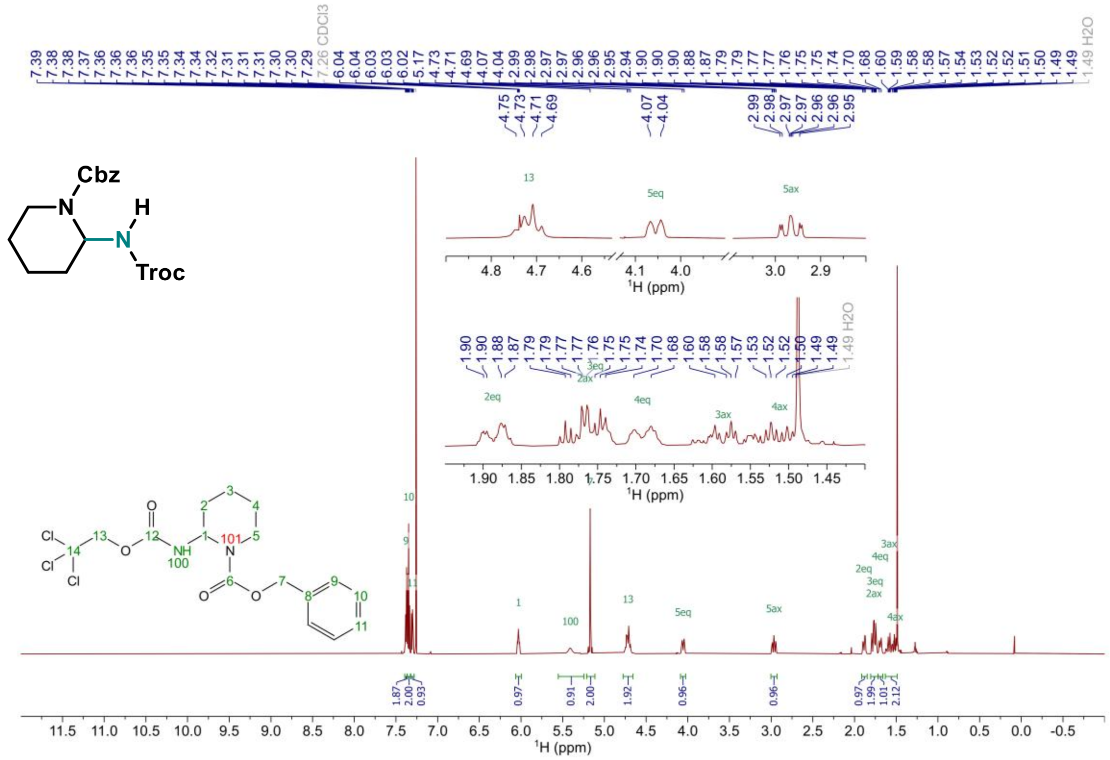

Compound **7h**: ^{13}C NMR (151 MHz, $CDCl_3$, 323 K)

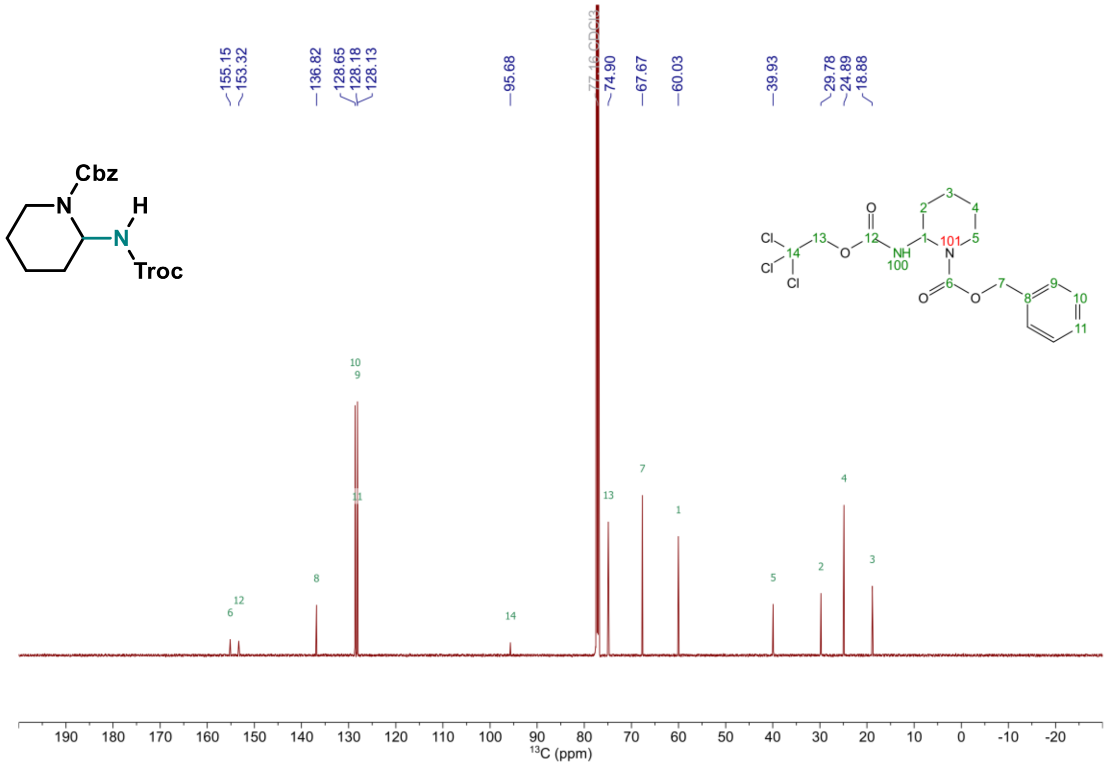

Compound **7h**: variable temperature ^{1}H NMR (600 MHz, $CDCl_3$)

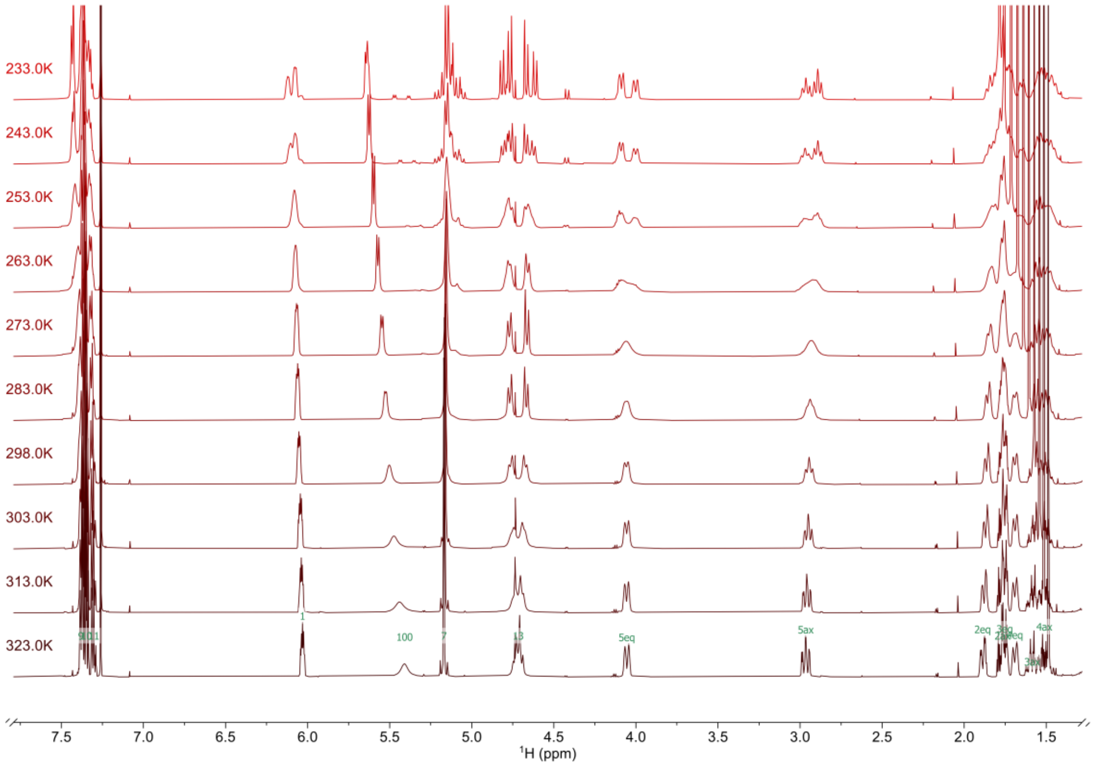

Compound **7h**: ^{1}H-^{13}C HSQC ($CDCl_3$, 323 K)

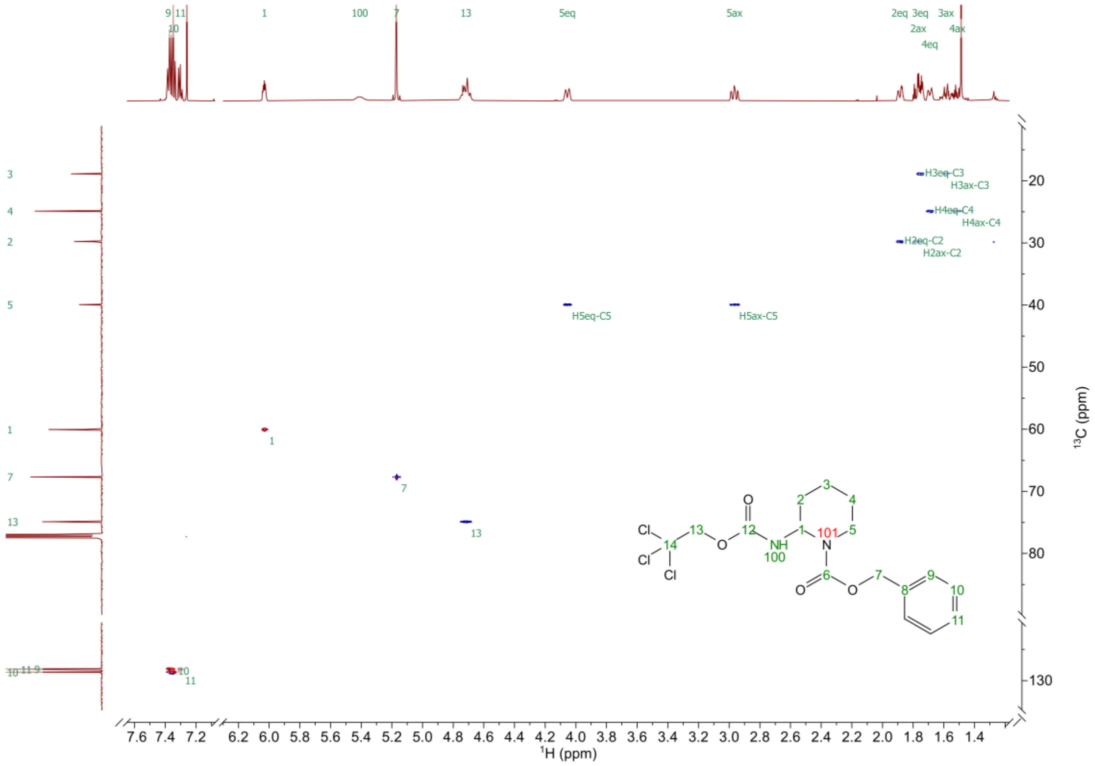

Compound **7h**: ^{1}H-^{13}C HMBC ($CDCl_3$, 323 K)

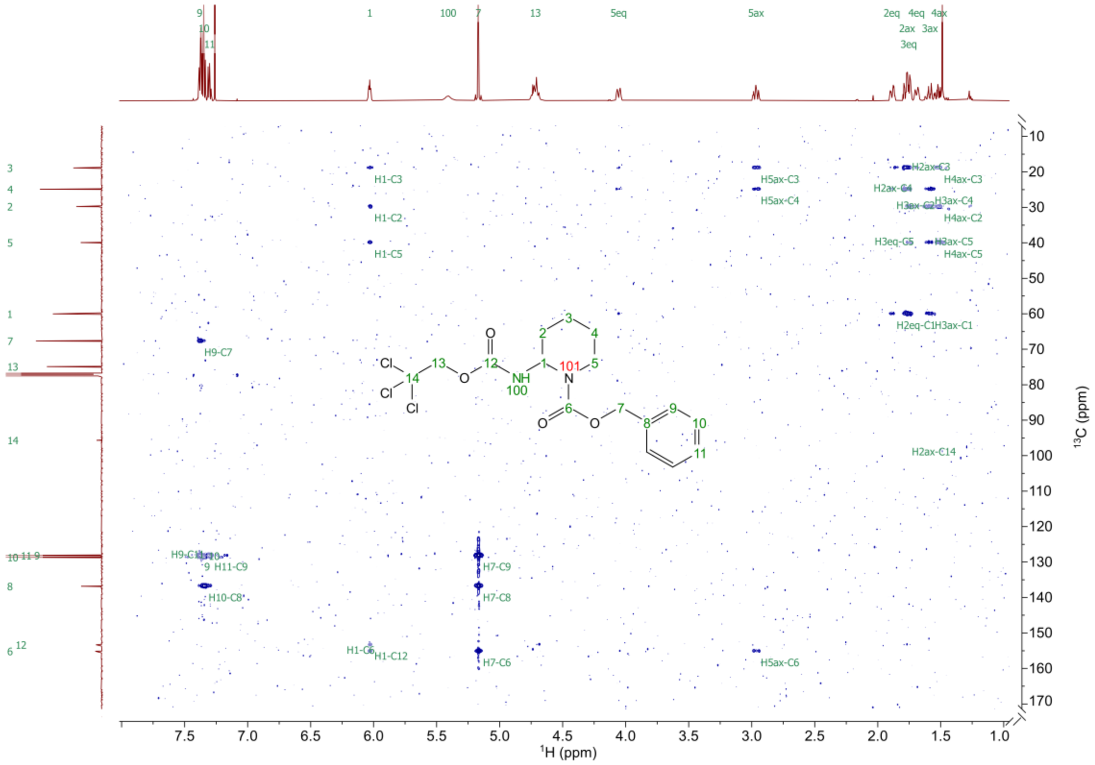

Compound **7h**: ^{1}H-^{1}H COSY ($CDCl_3$, 323 K)

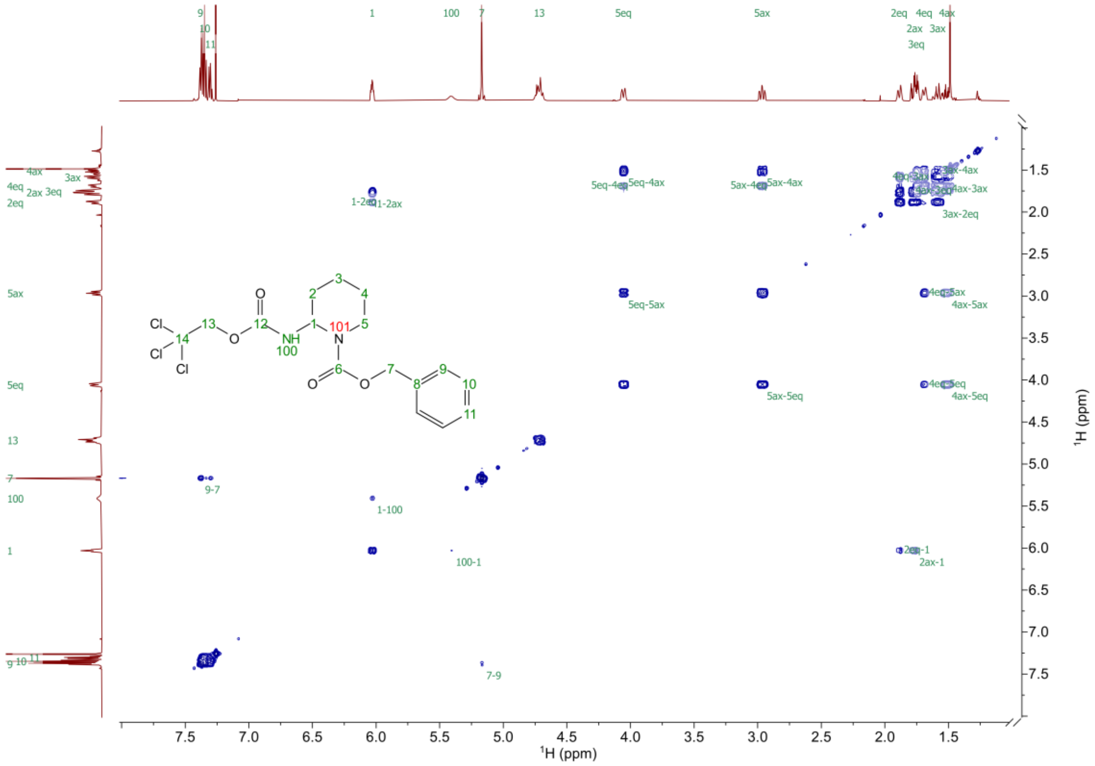

Compound **7h**: ^{1}H-^{1}H NOESY ($CDCl_3$, 323 K)

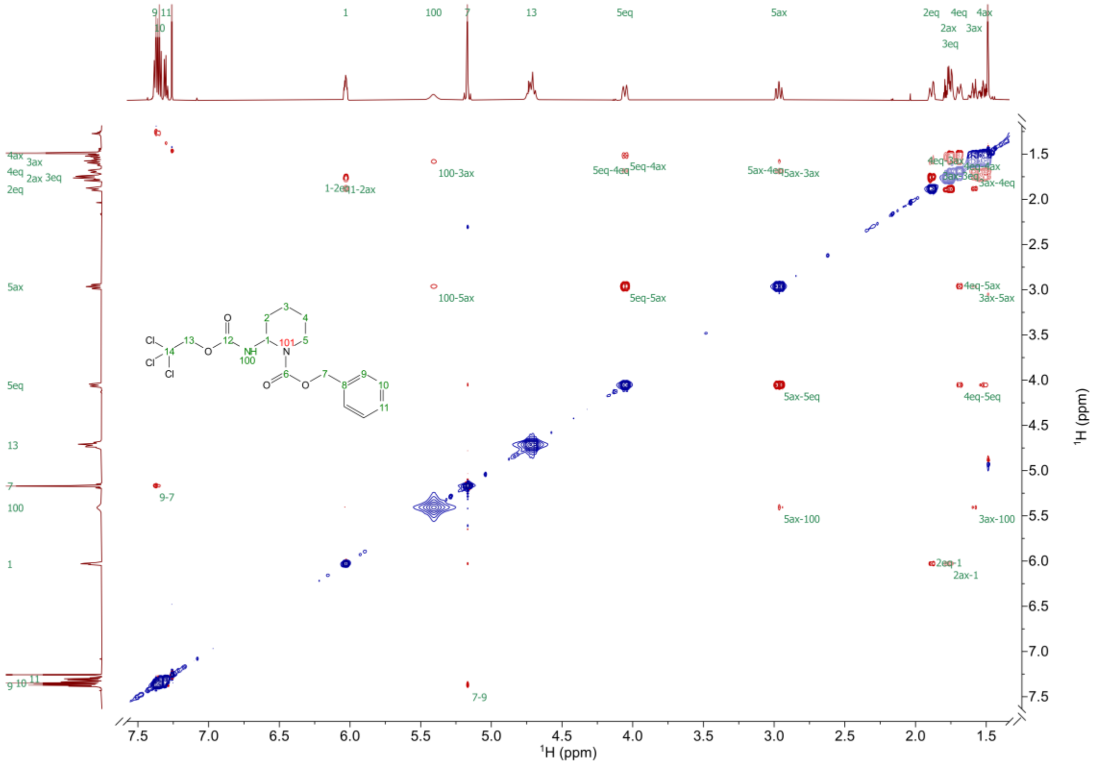

Compound **7h**: ^{1}H-^{15}N HMBC ($CDCl_3$, 323 K)

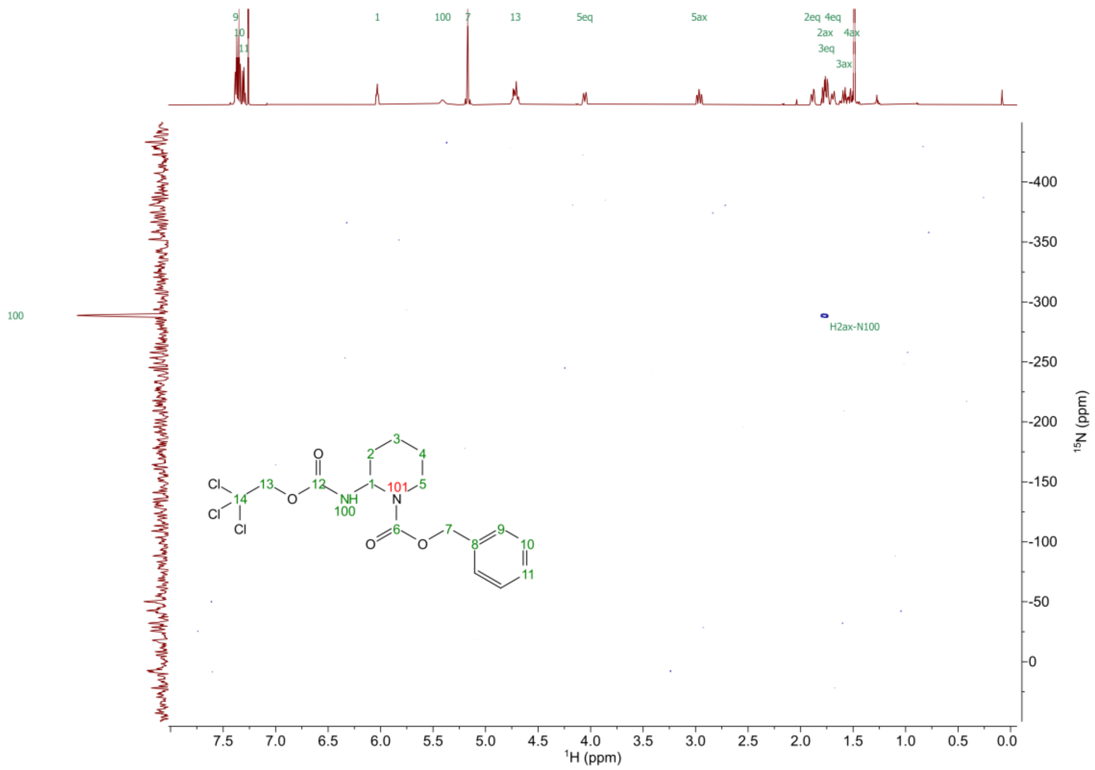

Compound **7i**: ^{1}H NMR (600 MHz, $CDCl_3$, 233 K)

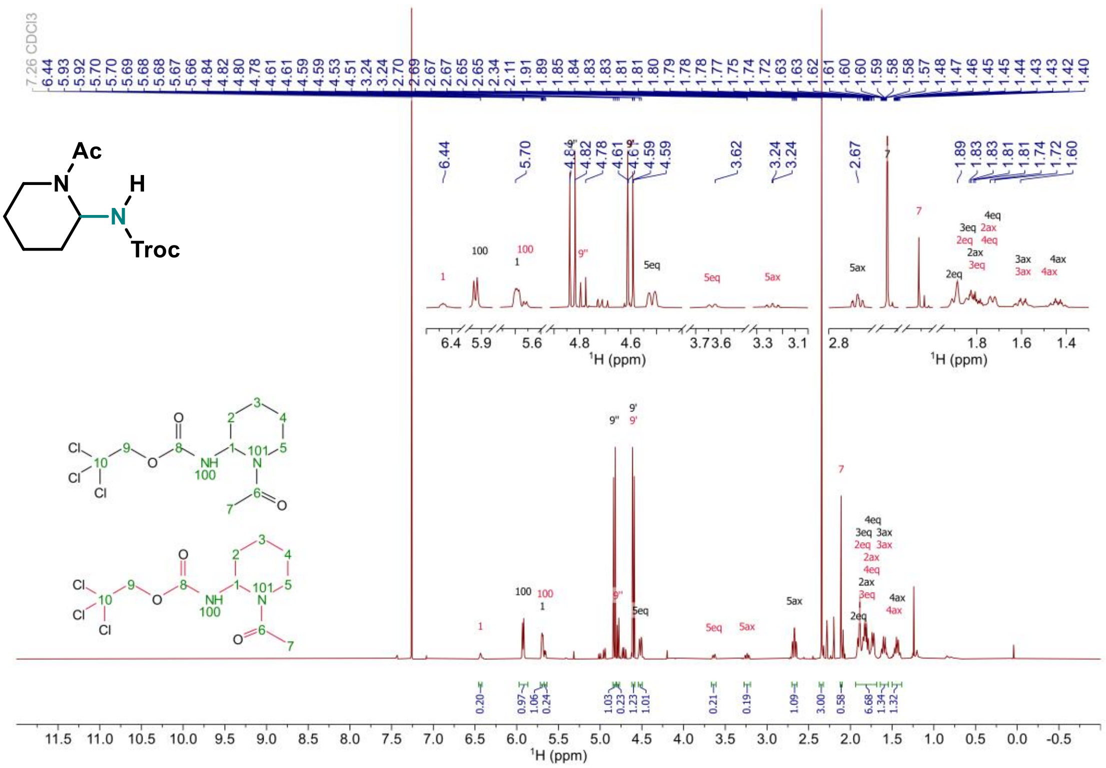

Compound **7i**: ^{13}C NMR (151 MHz, $CDCl_3$, 233 K)

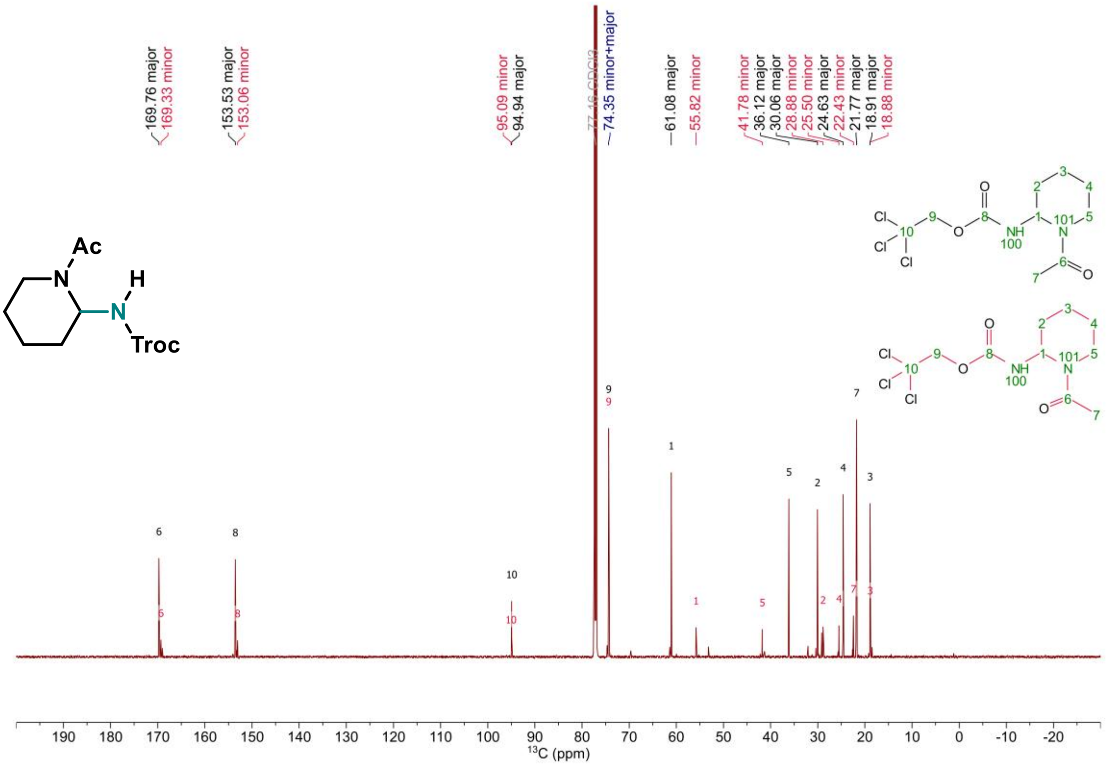

Compound **7i**: variable temperature ^{1}H NMR (600 MHz, $CDCl_3$)

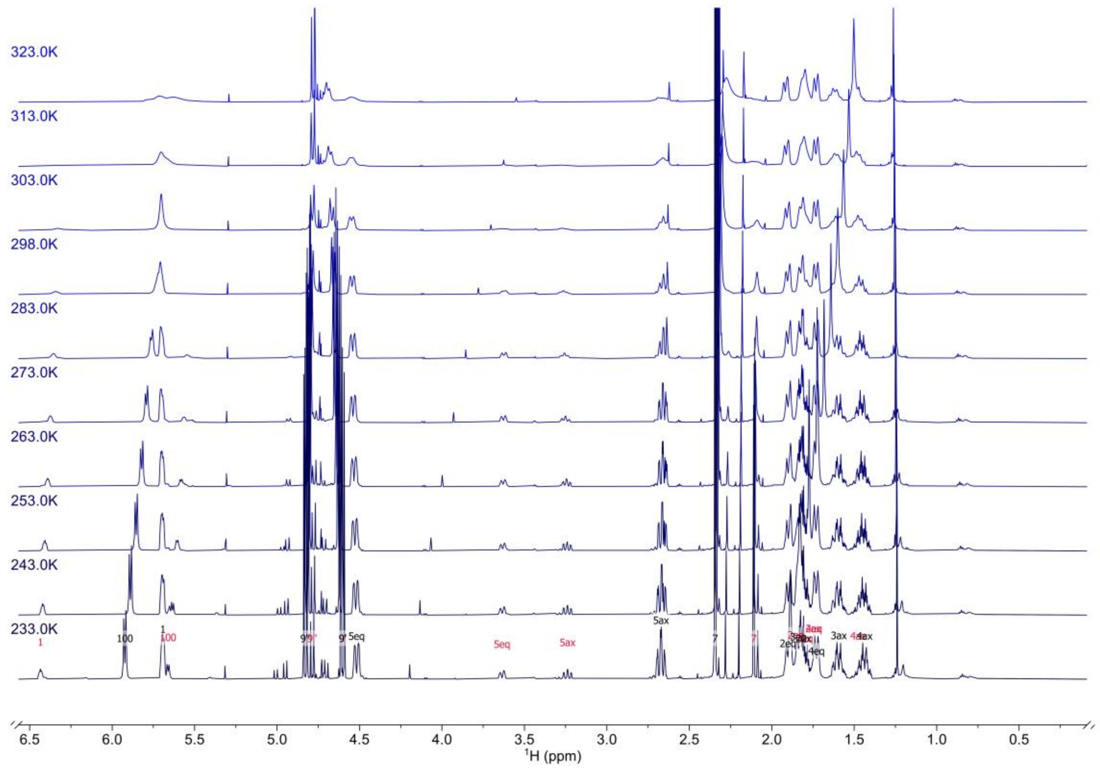

Compound **7i**: ^{1}H-^{13}C HSQC ($CDCl_3$, 233 K)

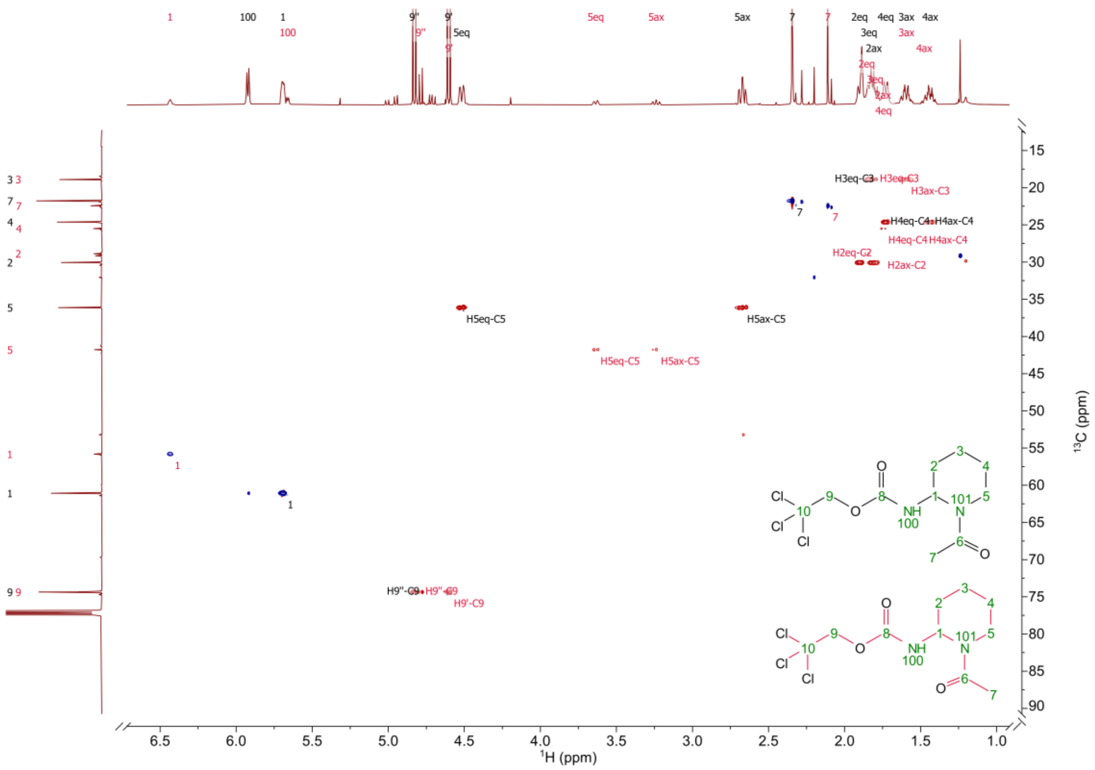

Compound **7i**: ^{1}H-^{13}C HMBC ($CDCl_3$, 233 K)

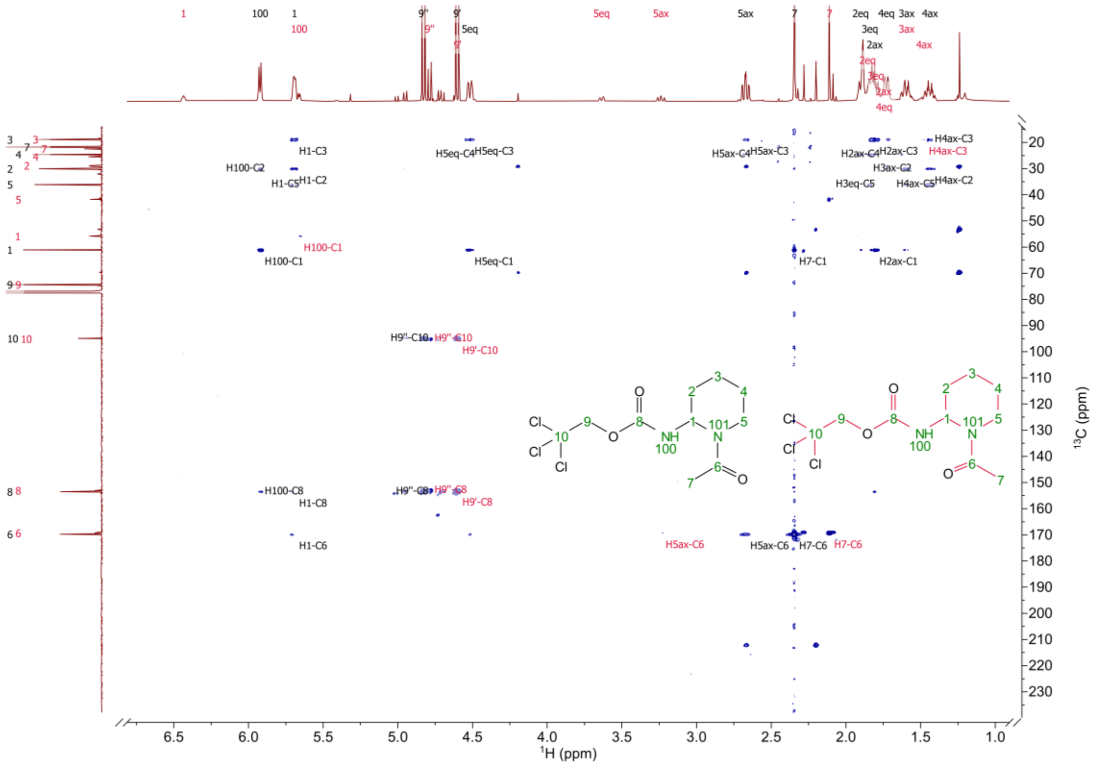

Compound **7i**: ^{1}H-^{1}H COSY ($CDCl_3$, 233 K)

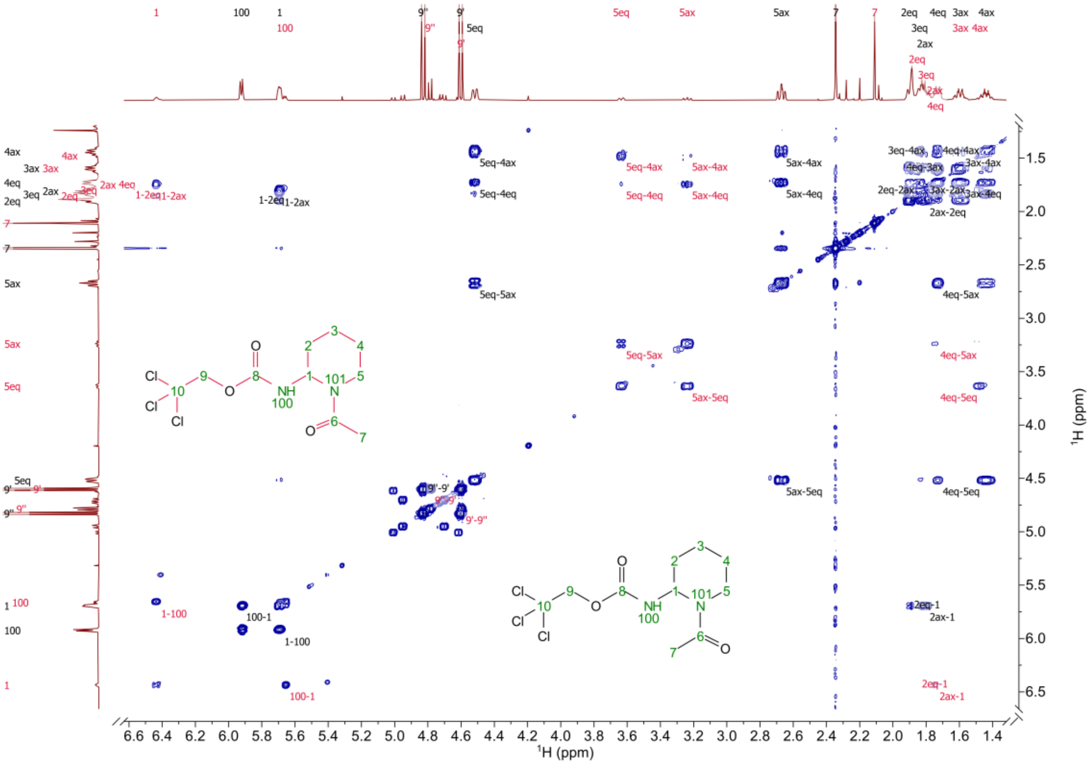

Compound **7i**: ^{1}H-^{1}H NOESY ($CDCl_3$, 233 K)

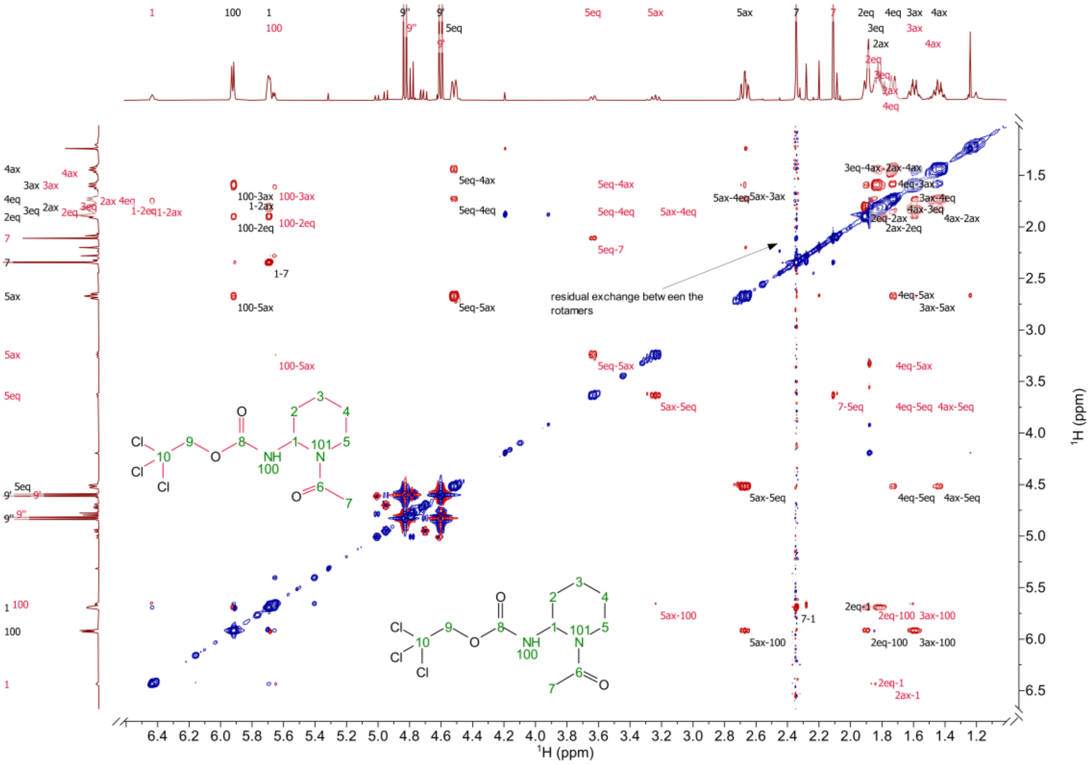

Compound **7i**: ^{1}H-^{15}N HMBC ($CDCl_3$, 233 K)

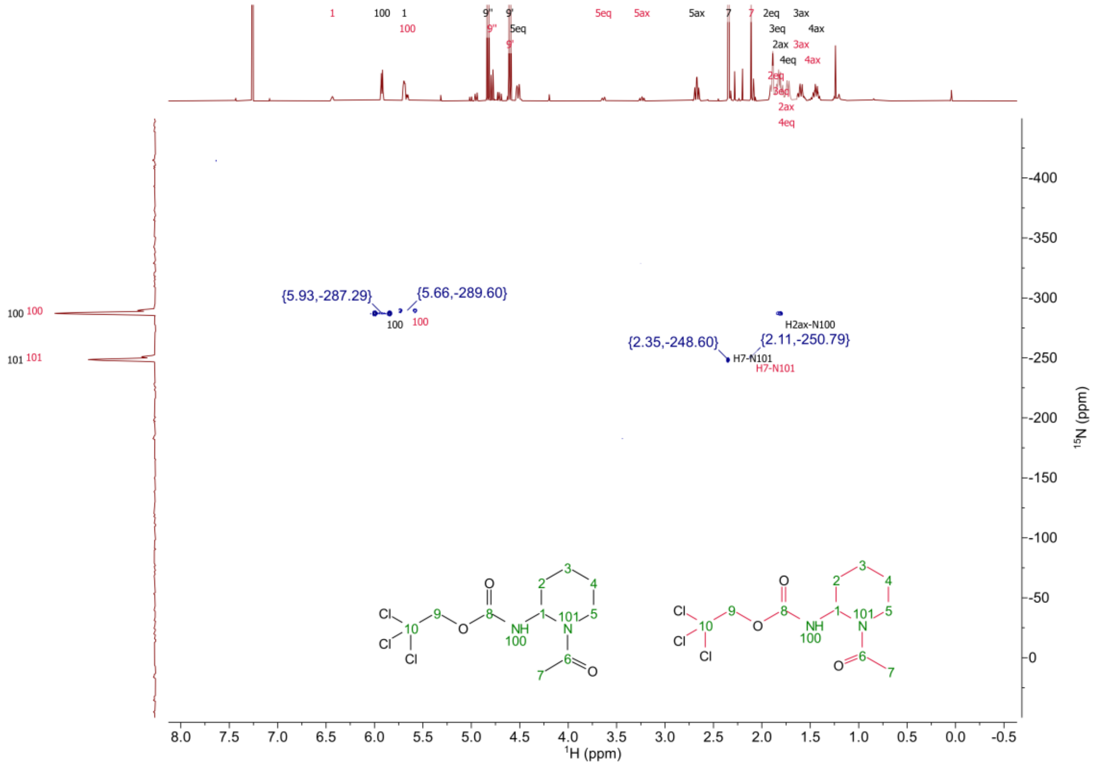

Compound **7j**: ^{1}H NMR ($CDCl_3$, 333 K)

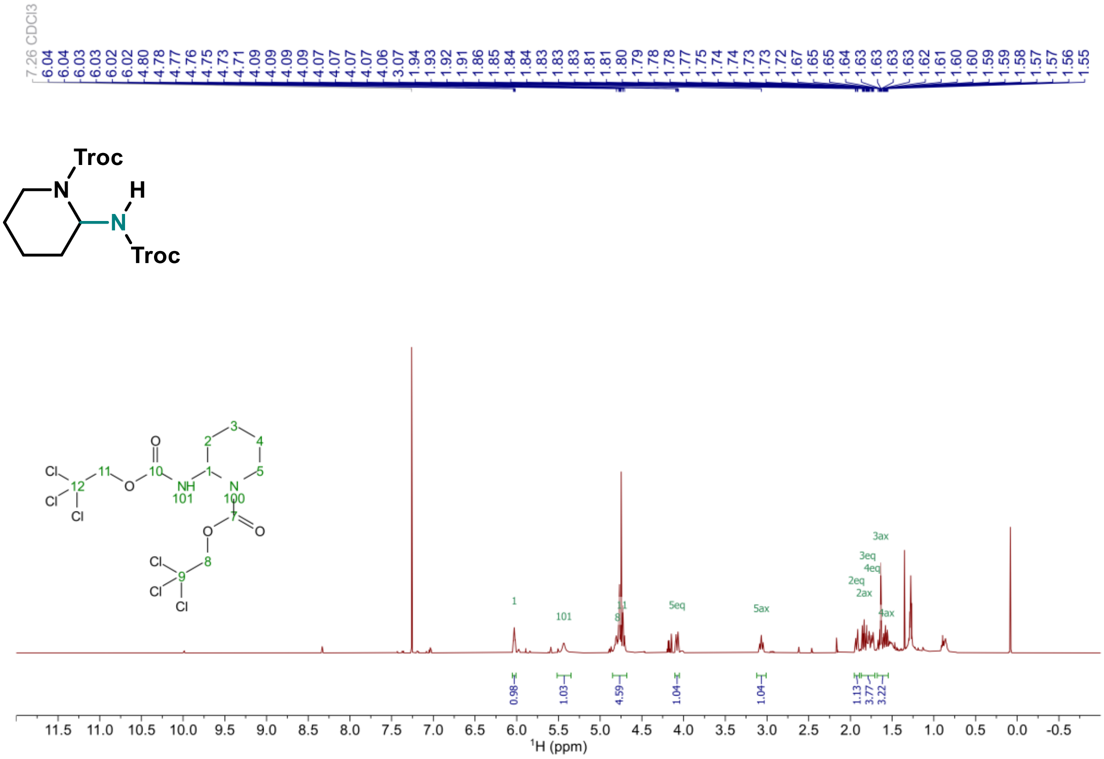

Compound **7j**: ^{13}C NMR ($CDCl_3$, 333 K)

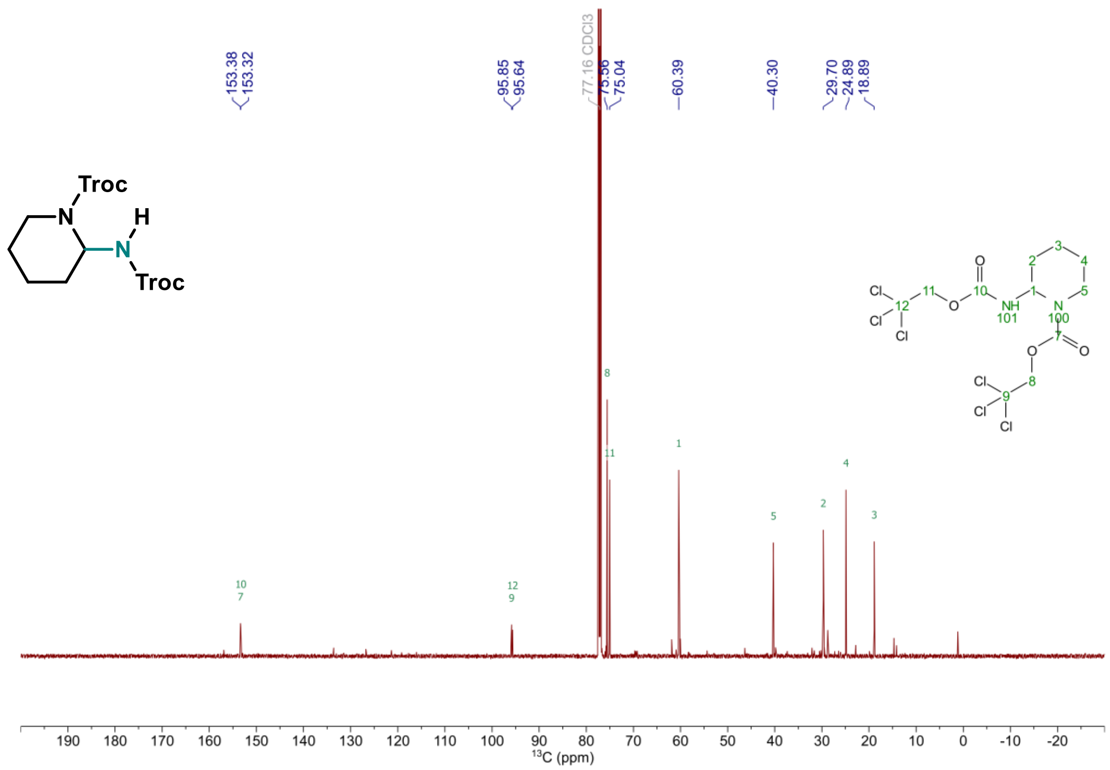

Compound **7j**: ^{1}H-^{13}C HSQC ($CDCl_3$, 333 K)

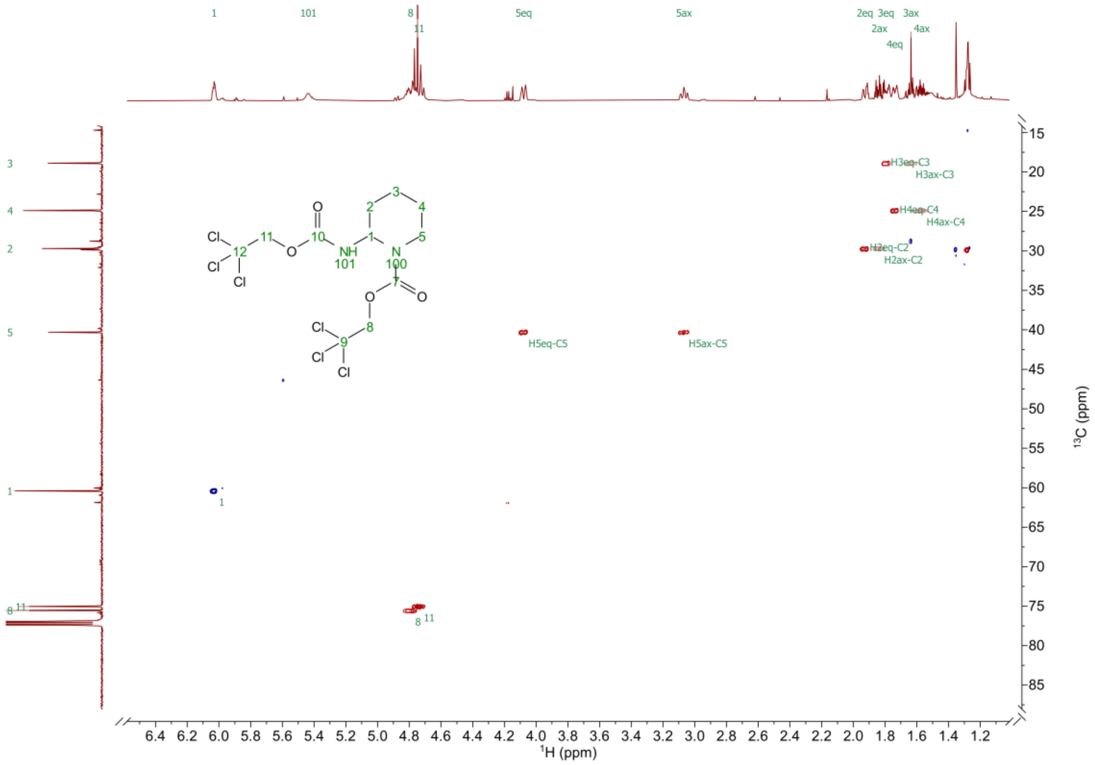

Compound **7j**: ^{1}H-^{13}C HMBC ($CDCl_3$, 333 K)

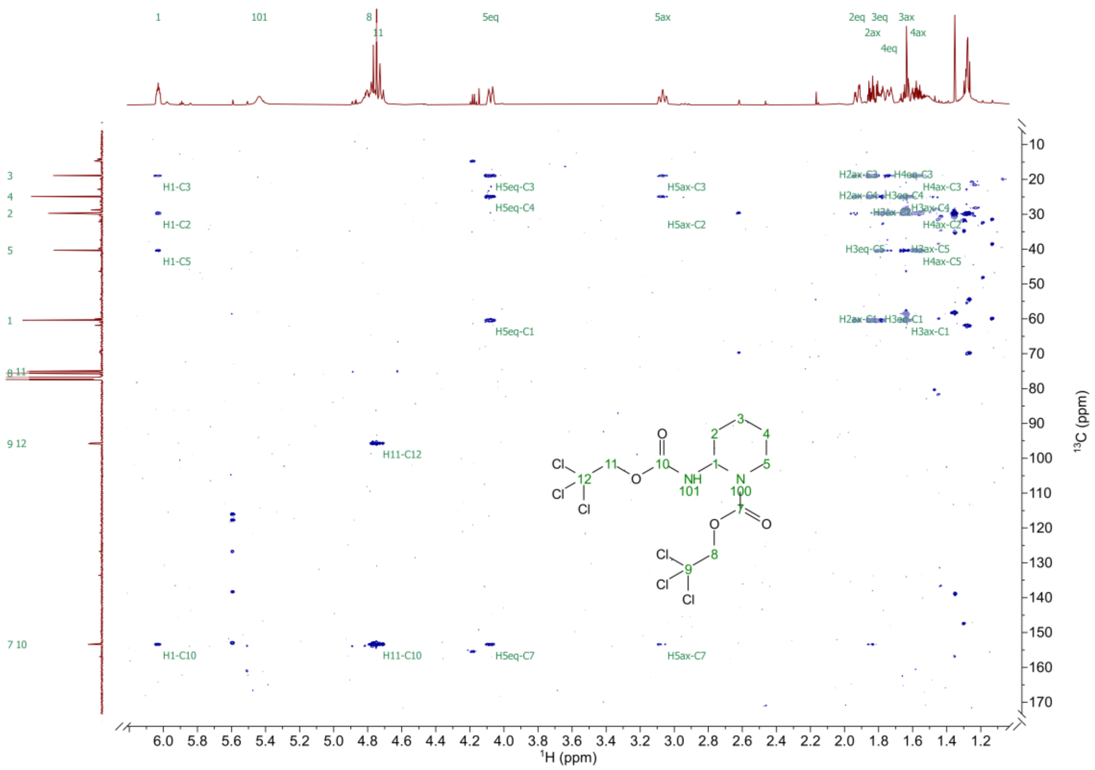

Compound **7j**: ^{1}H-^{1}H COSY ($CDCl_3$, 333 K)

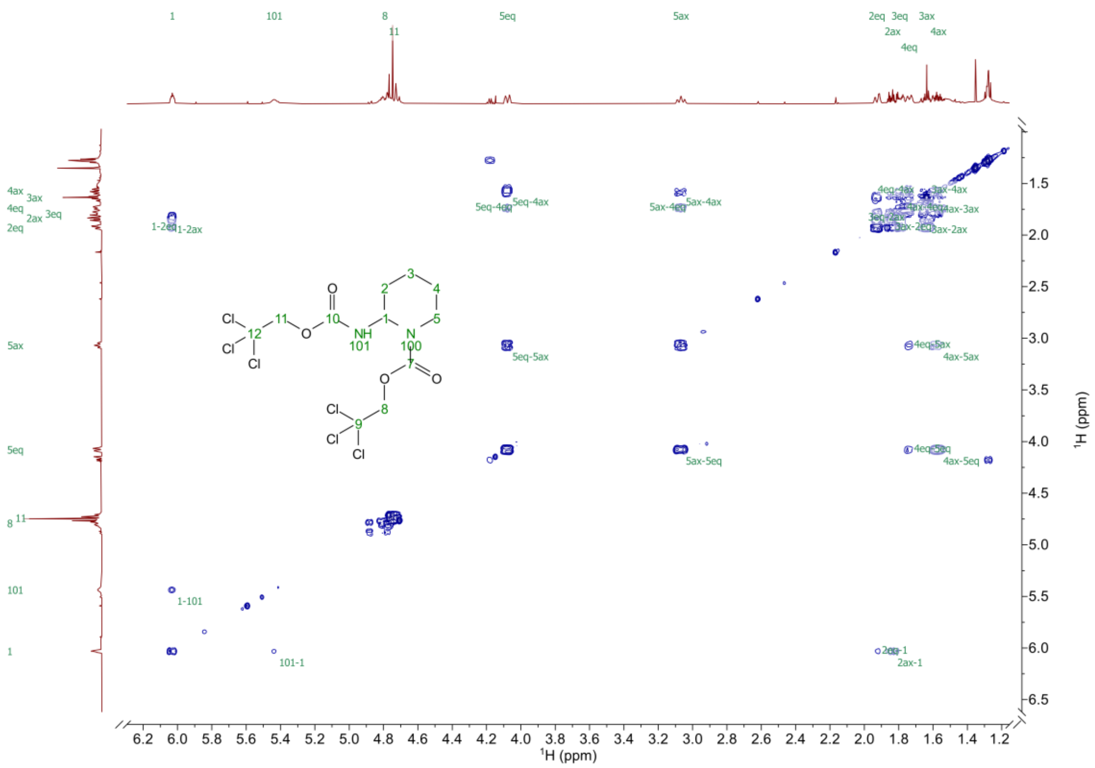

Compound **7j**: ^{1}H-^{1}H NOESY ($CDCl_3$, 333 K)

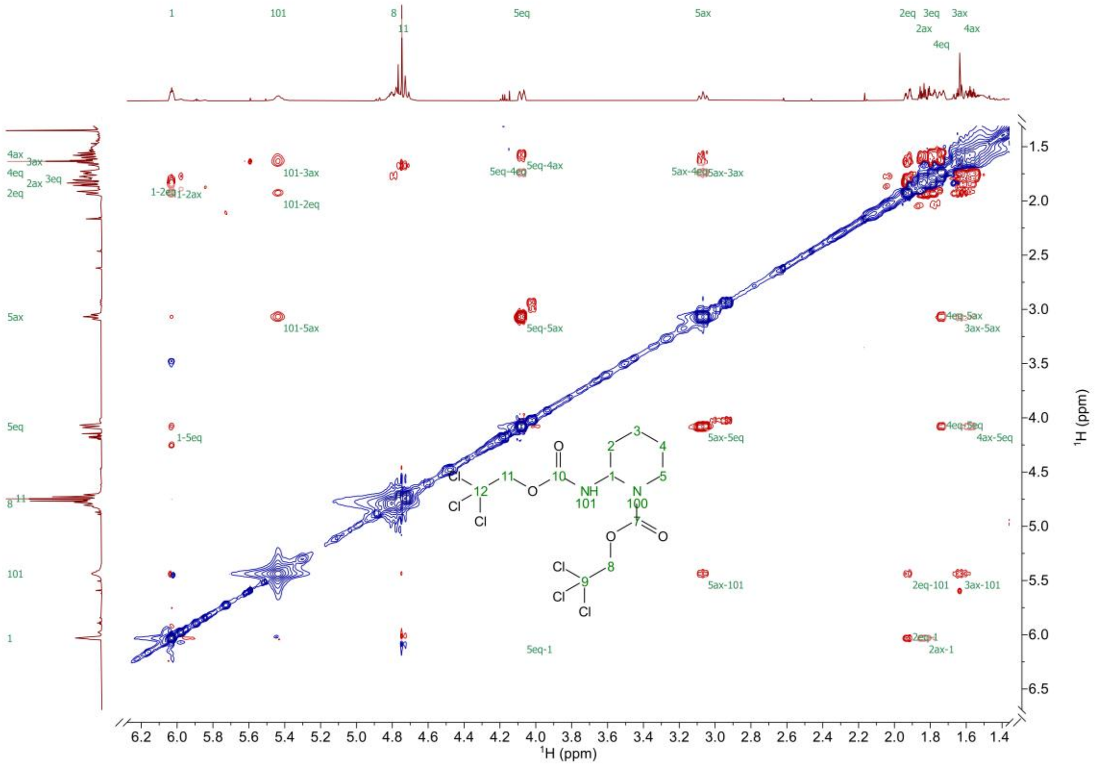

Compound **7j**: ^{1}H-^{15}N HMBC ($CDCl_3$, 333 K)

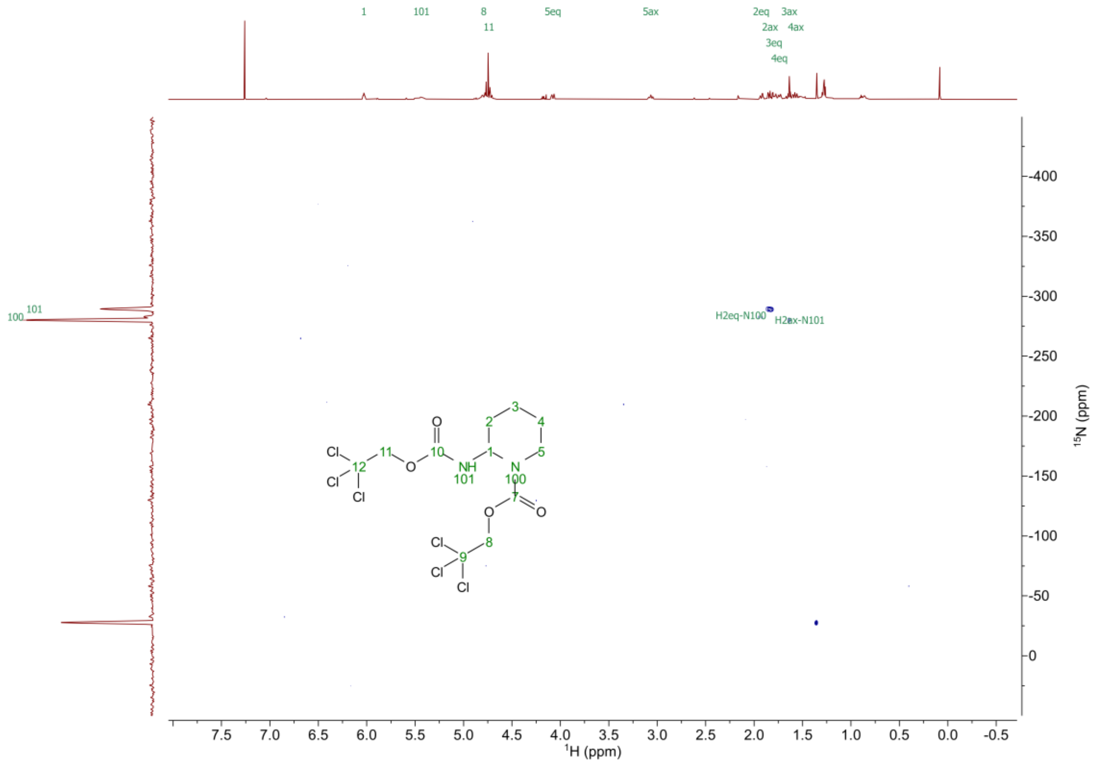

Compound **7k**: ^{1}H NMR (600 MHz, DMSO-d_6, 373 K)

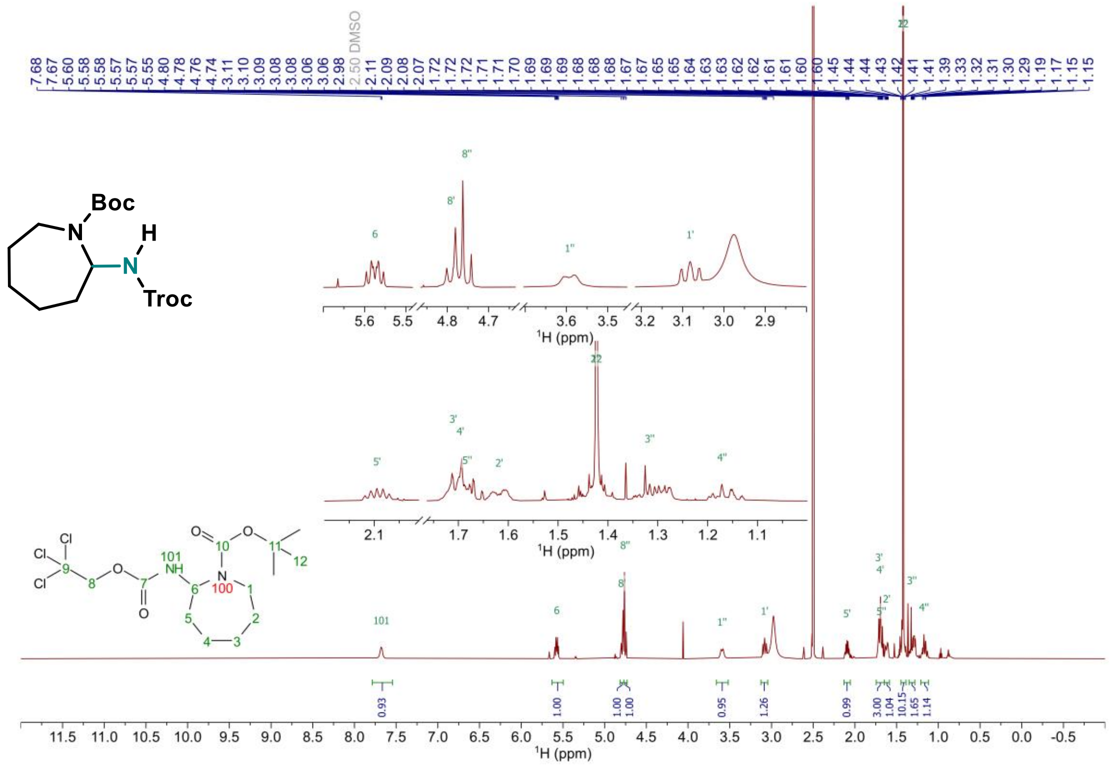

Compound **7k**: ^{13}C NMR (151 MHz, DMSO-d_6, 373 K)

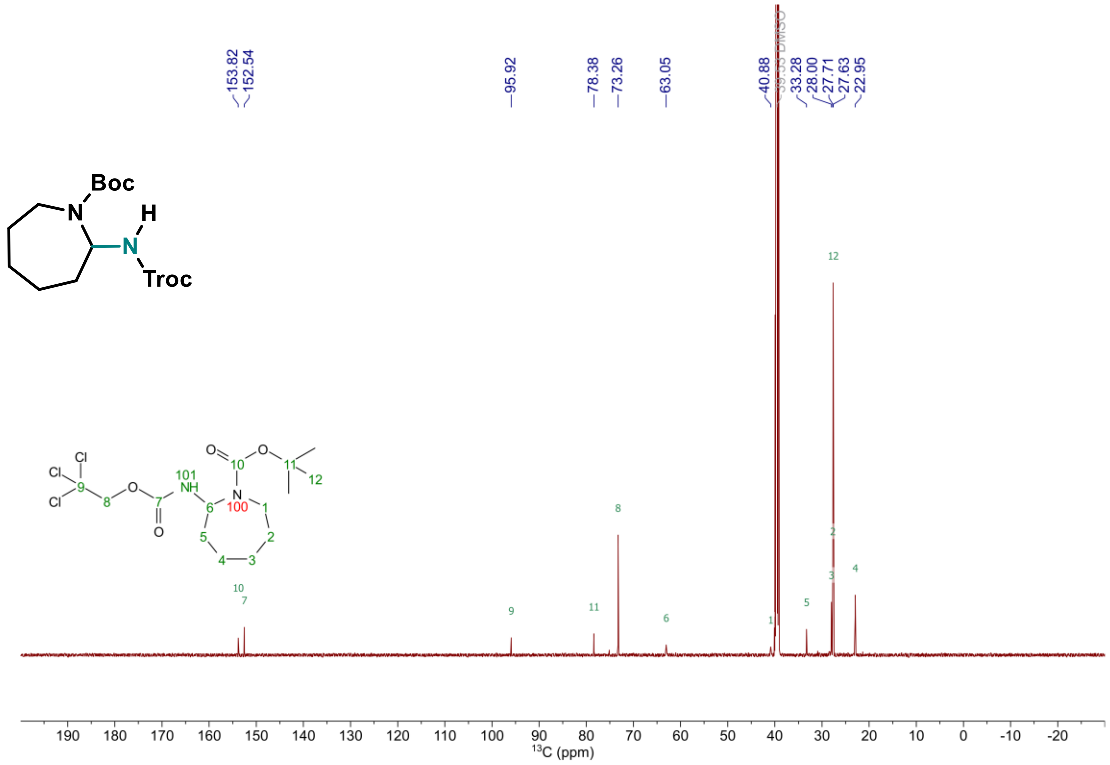

Compound **7k**: variable temperature ^{1}H NMR (600 MHz, DMSO-d_6)

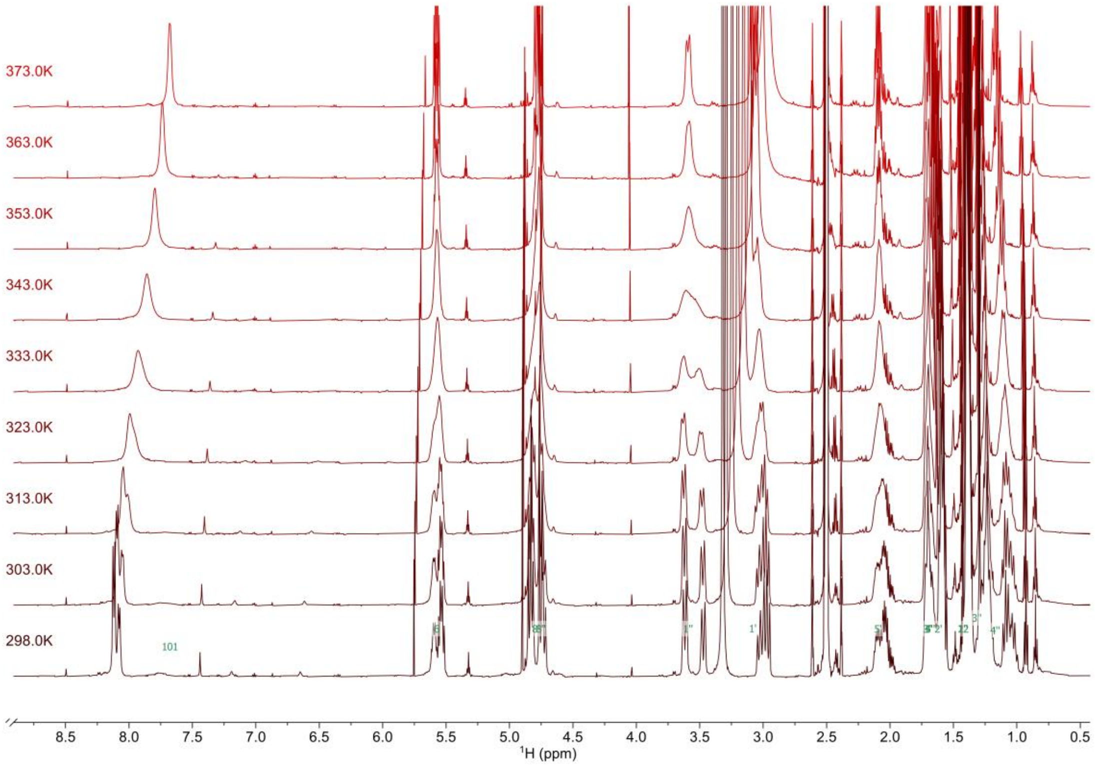

Compound **7k**: ^{1}H-^{13}C HSQC (DMSO-d_6, 373 K)

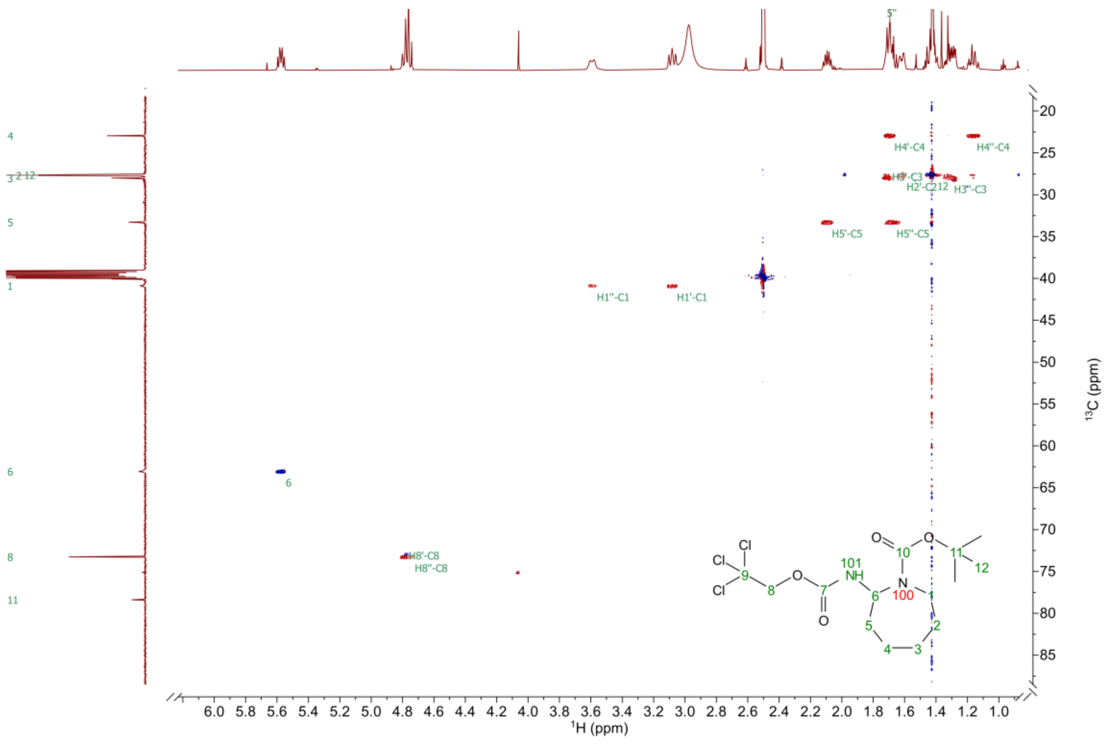

Compound **7k**: ^{1}H-^{13}C HMBC (DMSO-d_6, 373 K)

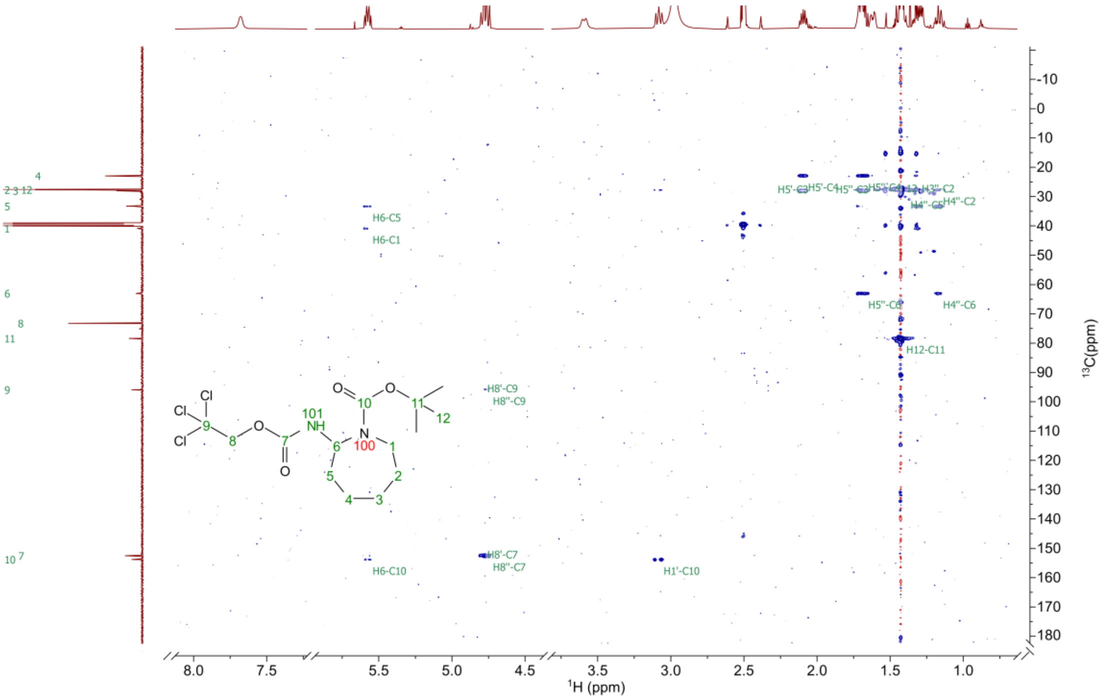

Compound **7k**: ^{1}H-^{1}H COSY (DMSO-d_6, 373 K)

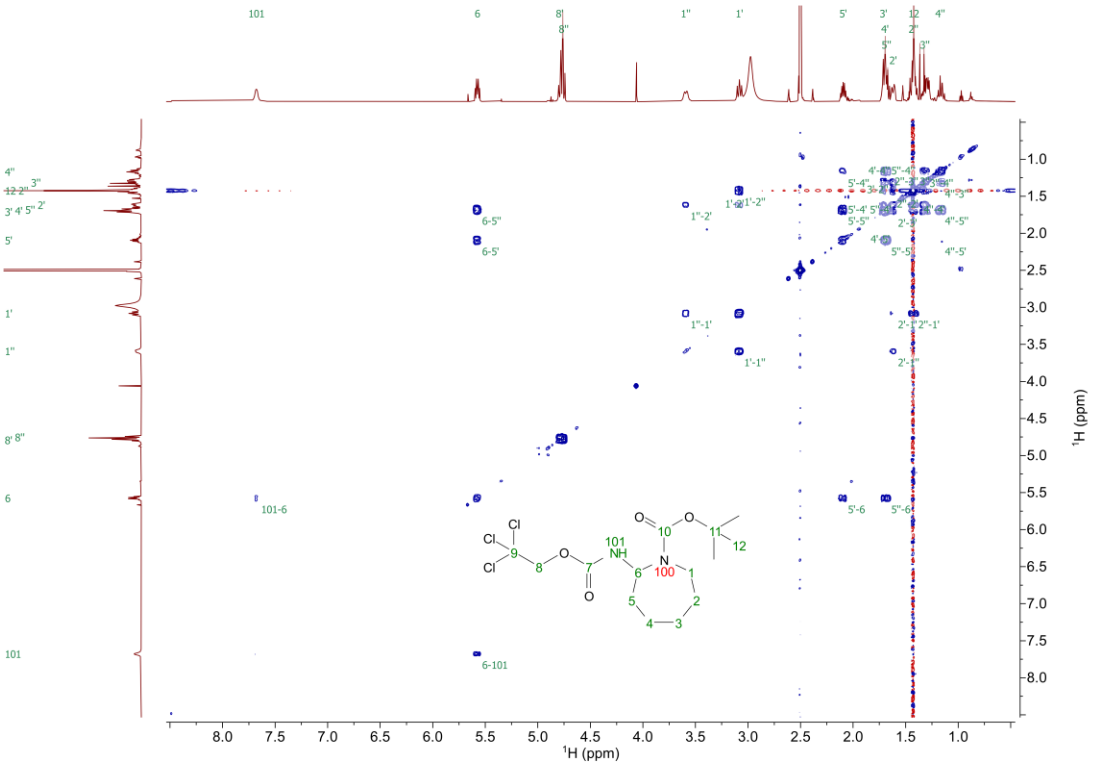

Compound **7k**: ^{1}H-^{1}H NOESY (DMSO-d_6, 373 K)

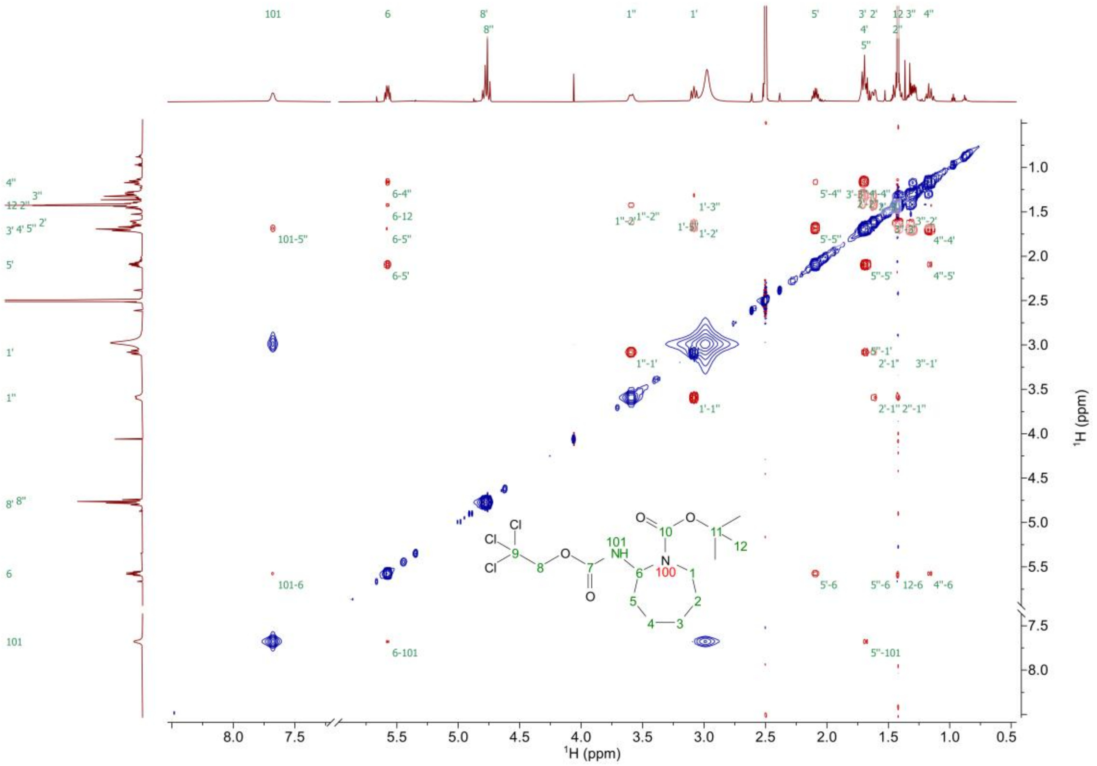

Compound **7l**, major diastereomer: ^{1}H NMR (600 MHz, $CDCl_3$, 323 K)

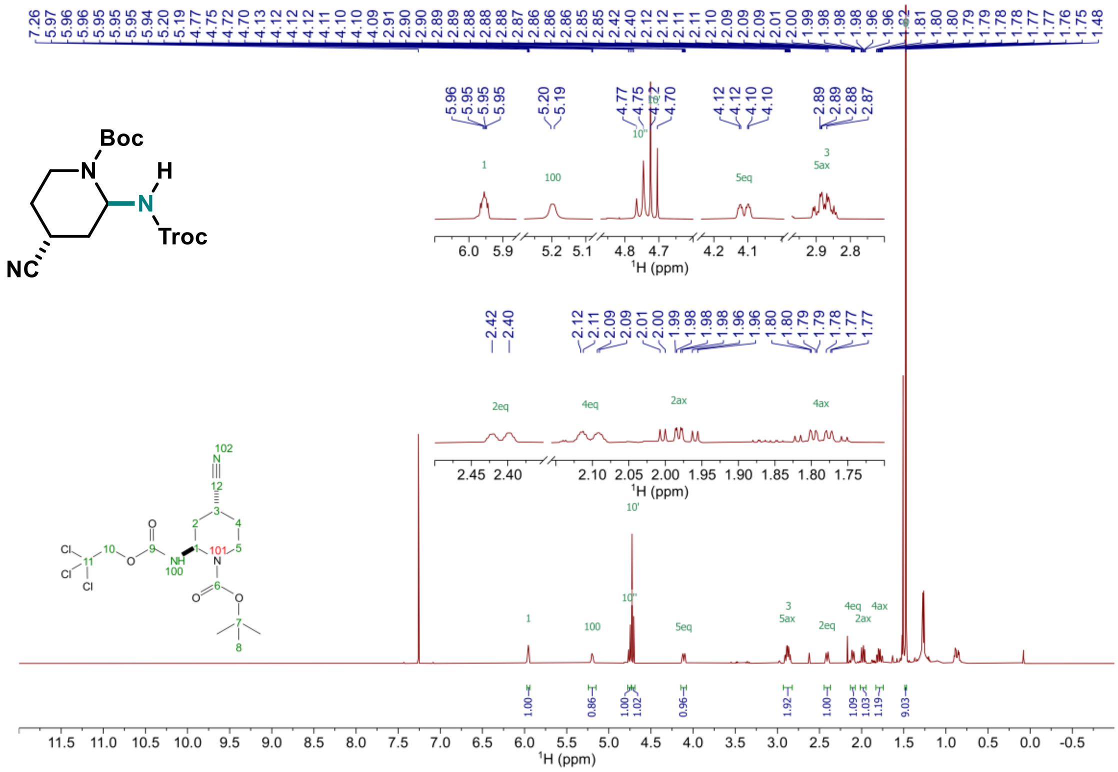

Compound **7l**, major diastereomer: ^{13}C NMR (151 MHz, $CDCl_3$, 323 K)

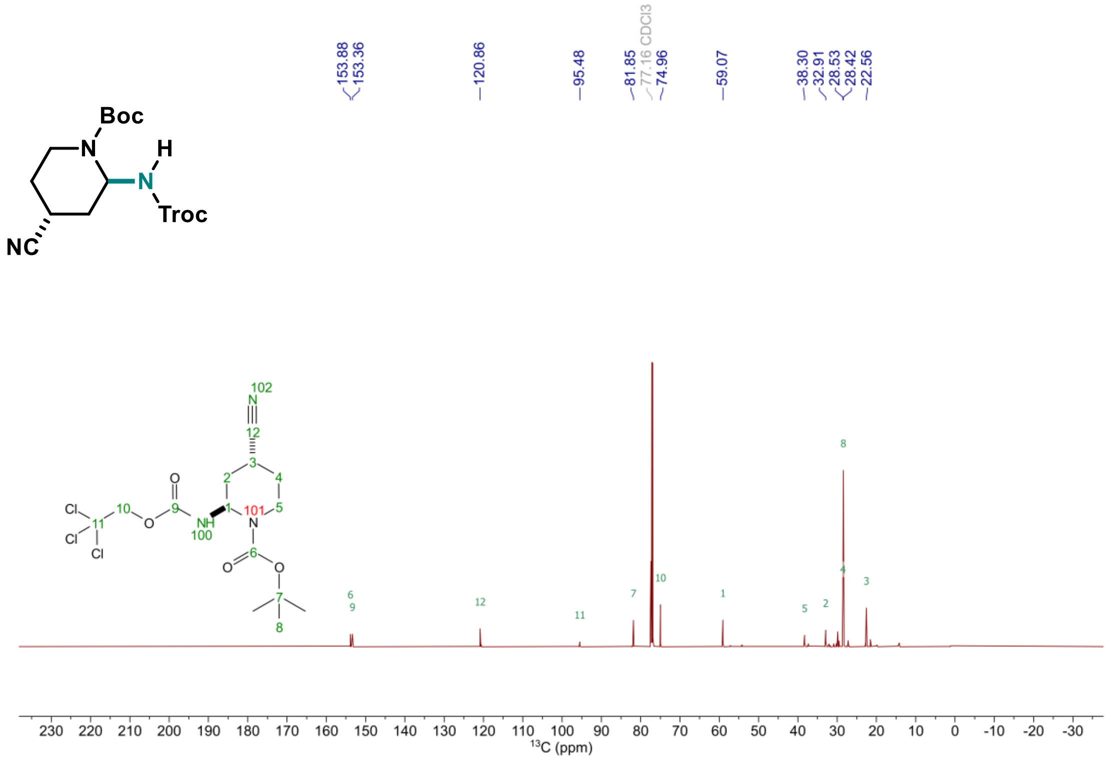

Compound **7l**, major diastereomer: variable temperature ^{1}H NMR (600 MHz, $CDCl_3$)

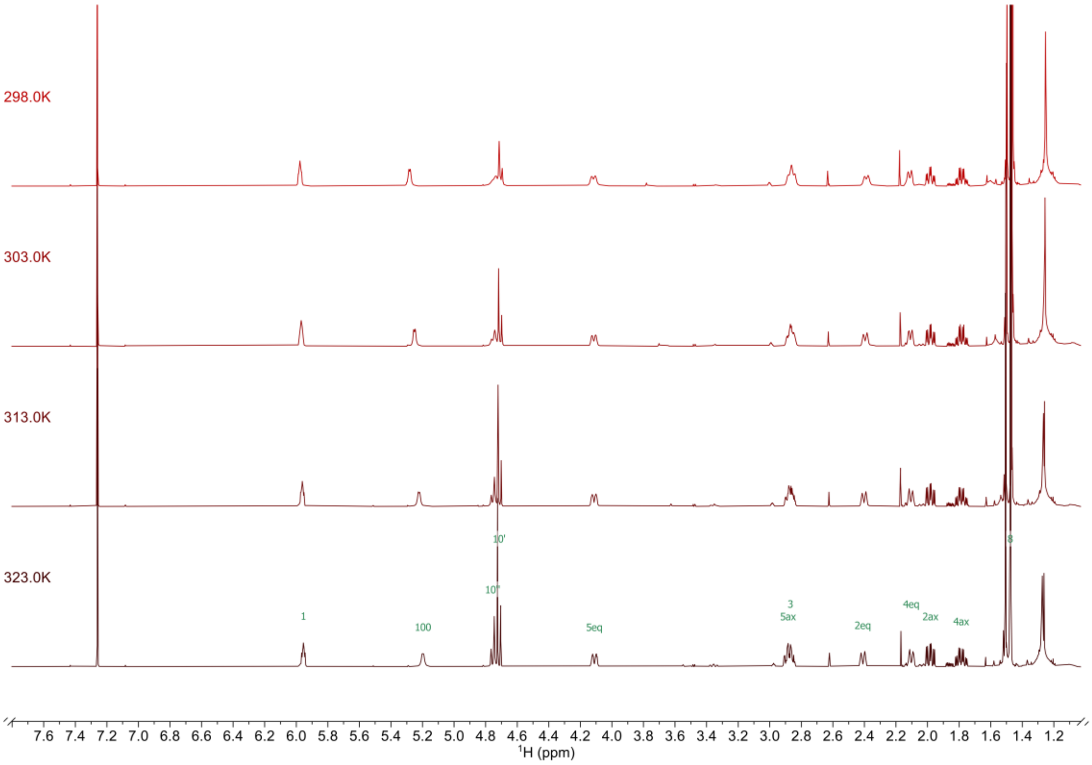

Compound **7l**, major diastereomer: ^{1}H-^{13}C HSQC ($CDCl_3$, 323 K)

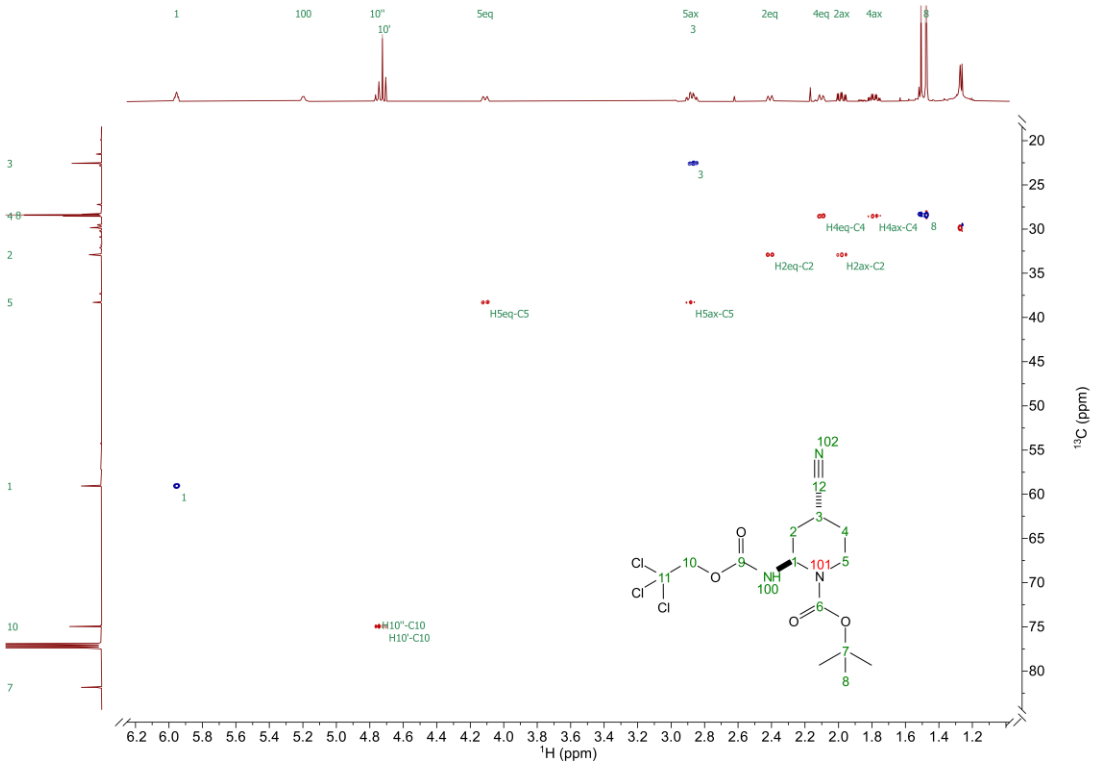

Compound **7l**, major diastereomer: ^{1}H-^{13}C HMBC ($CDCl_3$, 323 K)

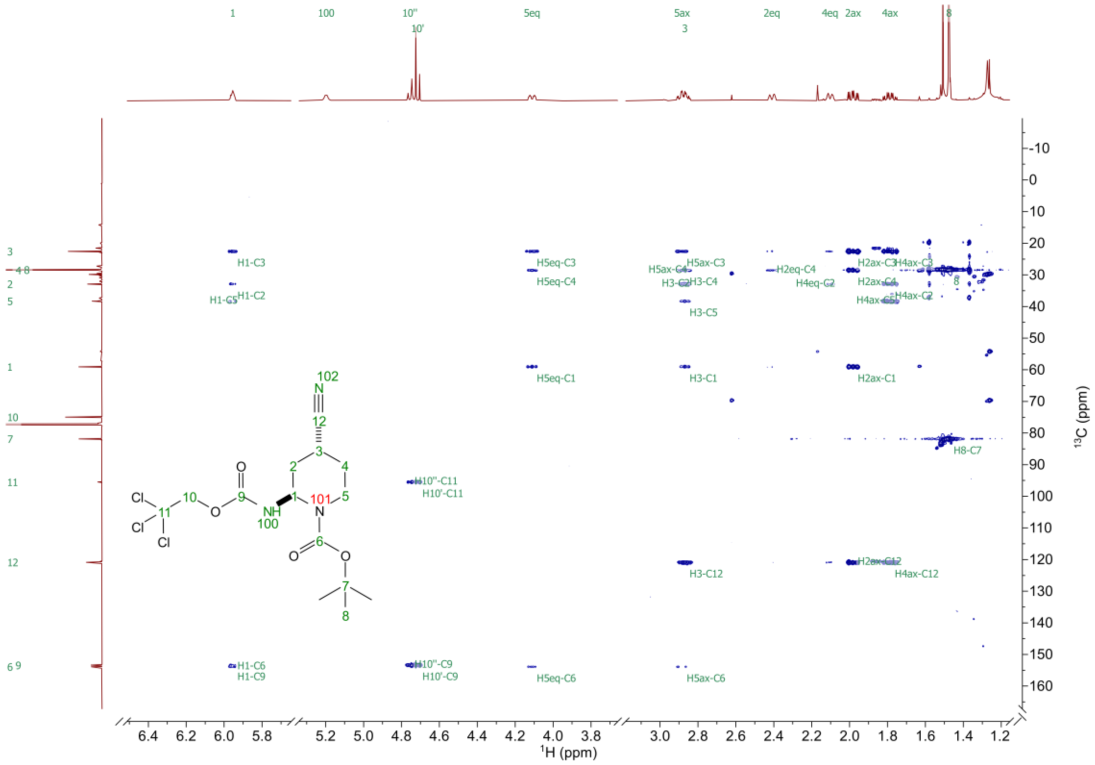

Compound **7l**, major diastereomer: ^{1}H-^{1}H COSY ($CDCl_3$, 323 K)

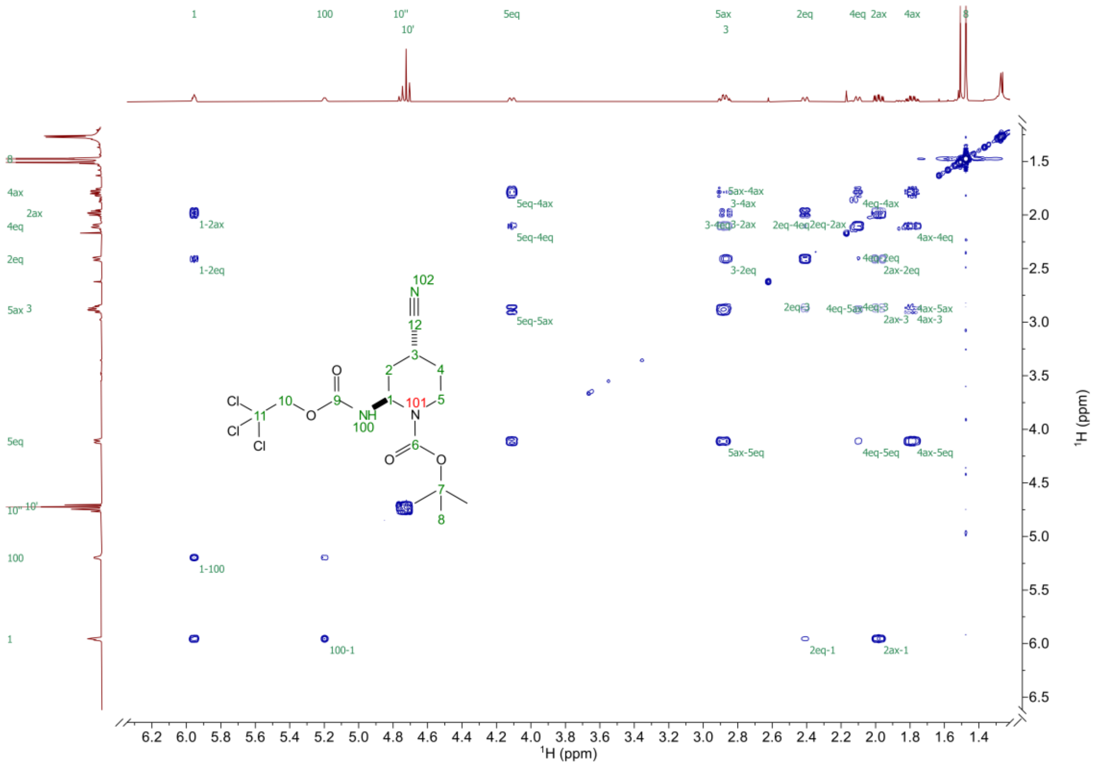

Compound **7l**, major diastereomer: ^{1}H-^{1}H NOESY ($CDCl_3$, 323 K)

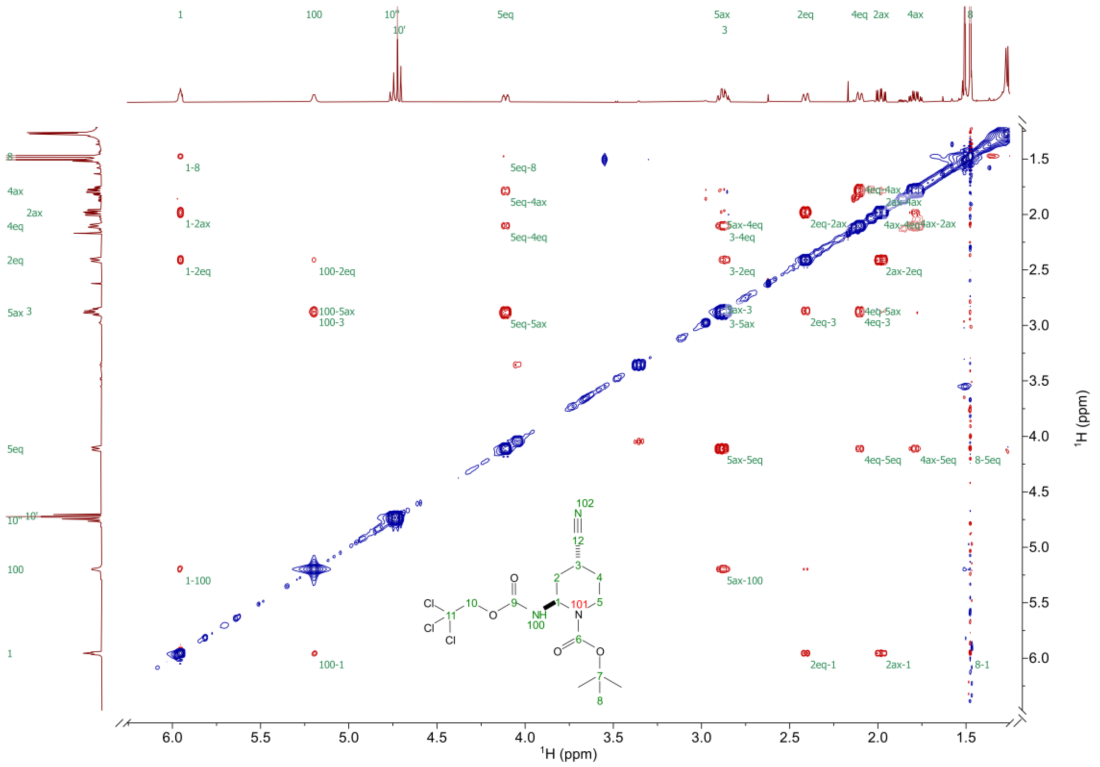

Compound **7l**, major diastereomer: ^{1}H-^{15}N HMBC ($CDCl_3$, 323 K)

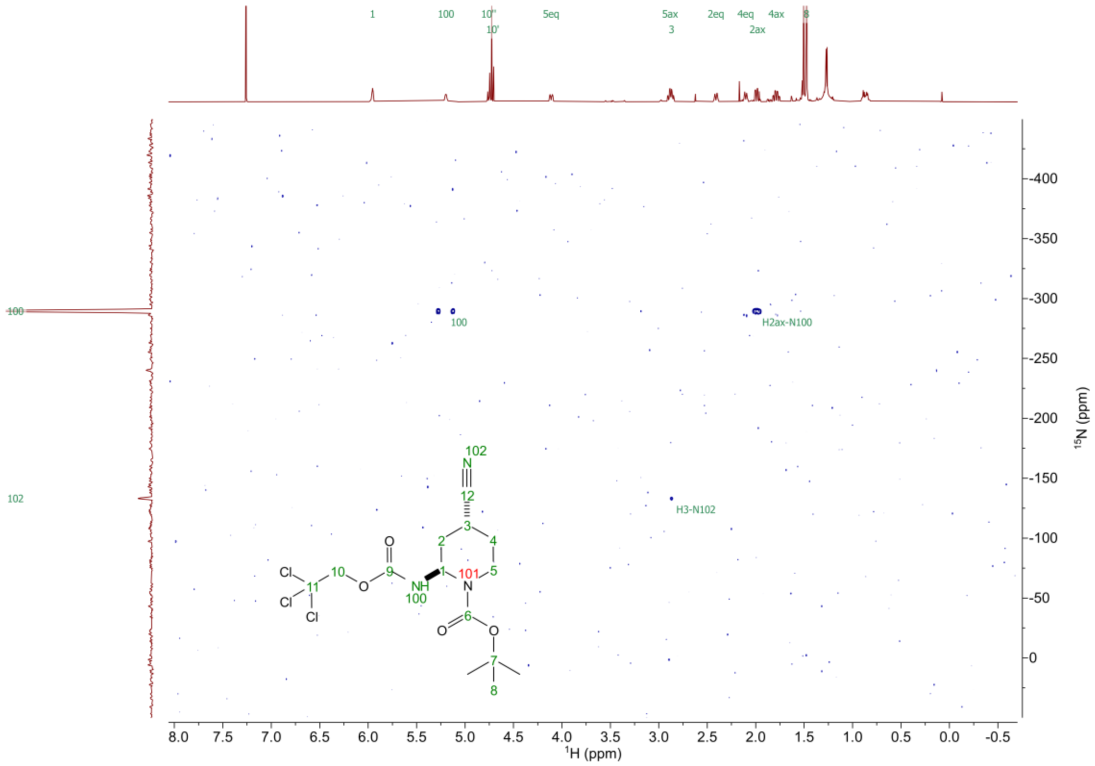

Compound **7l**, minor diastereomer: ^{1}H NMR (600 MHz, $CDCl_3$, 323 K)

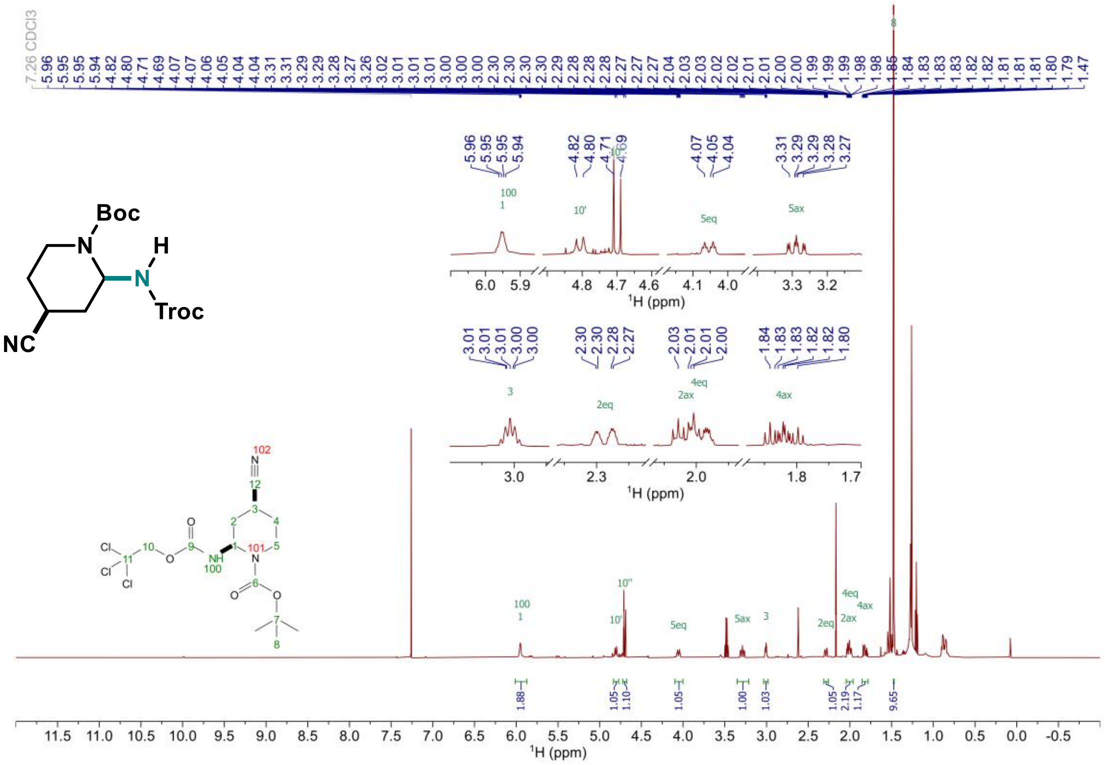

Compound **7l**, minor diastereomer: ^{13}C NMR (151 MHz, $CDCl_3$, 323 K)

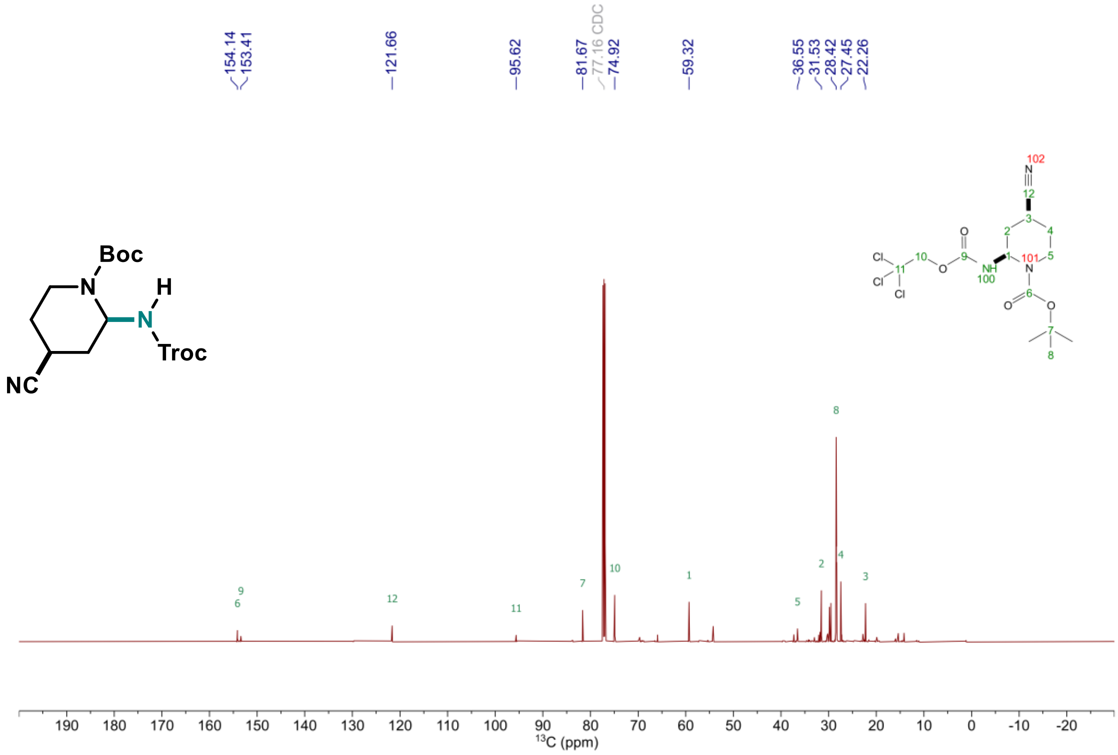

Compound **7l**, minor diastereomer: variable temperature ^{1}H NMR (600 MHz, $CDCl_3$)

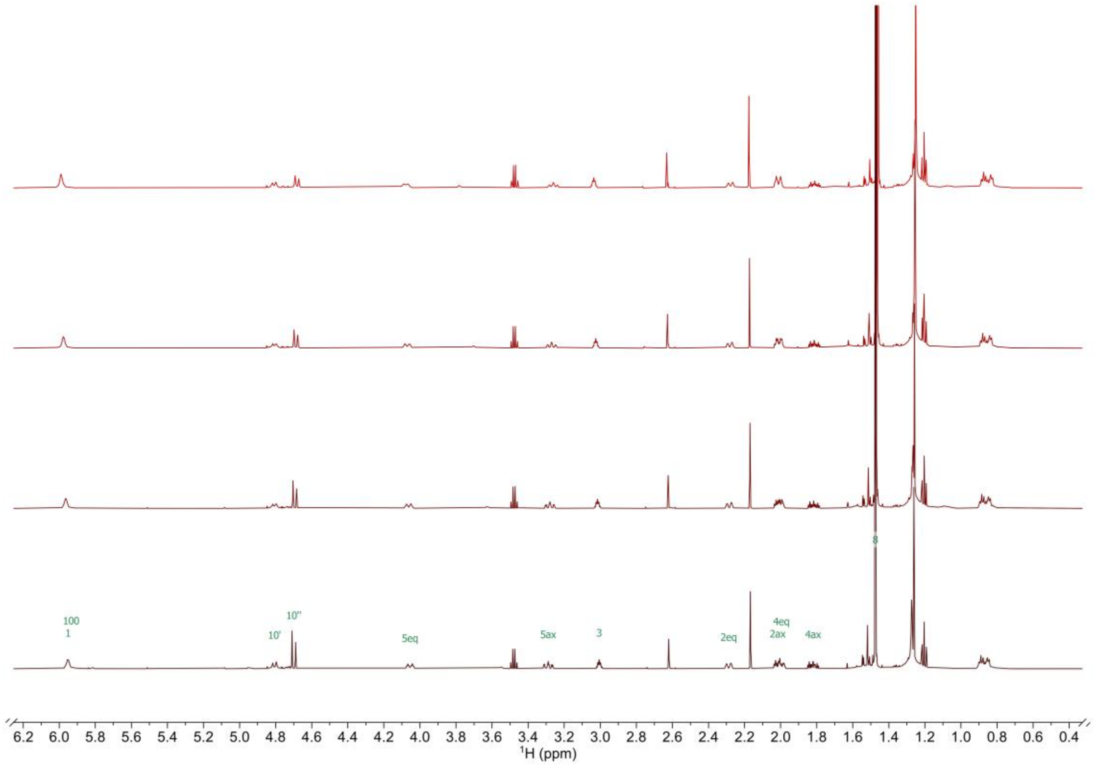

Compound **7l**, minor diastereomer: ^{1}H-^{13}C HSQC ($CDCl_3$, 323 K)

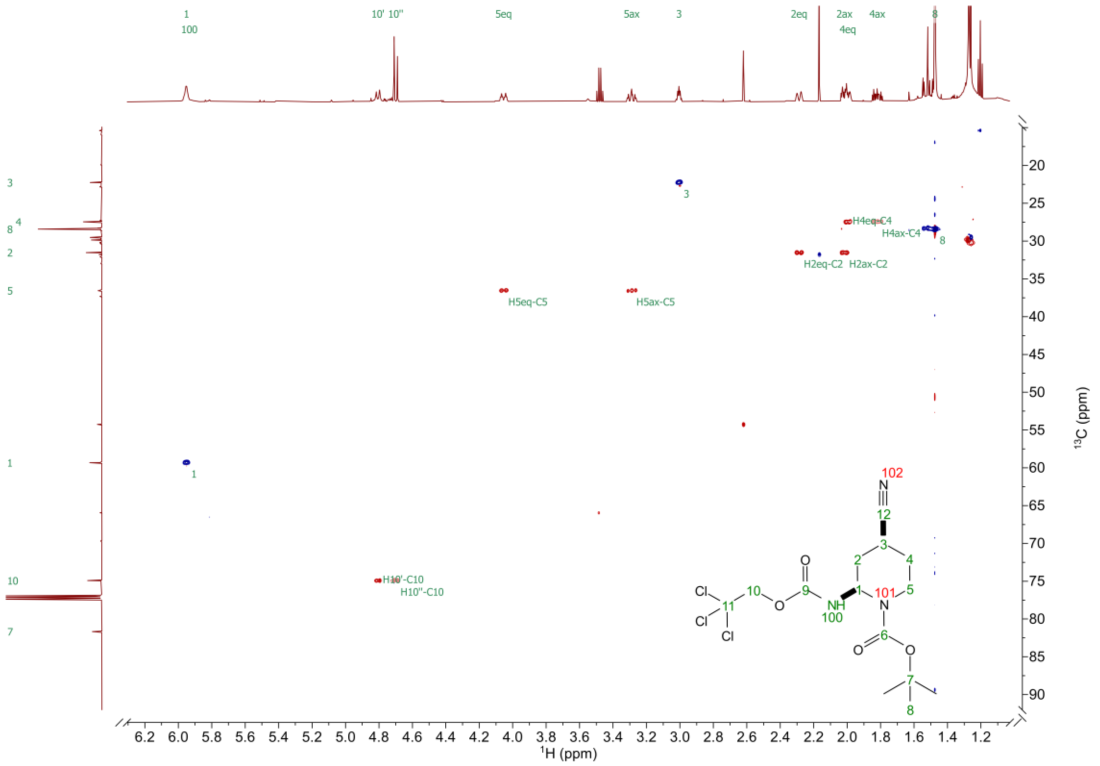

Compound **7l**, minor diastereomer: ^{1}H-^{13}C HMBC ($CDCl_3$, 323 K)

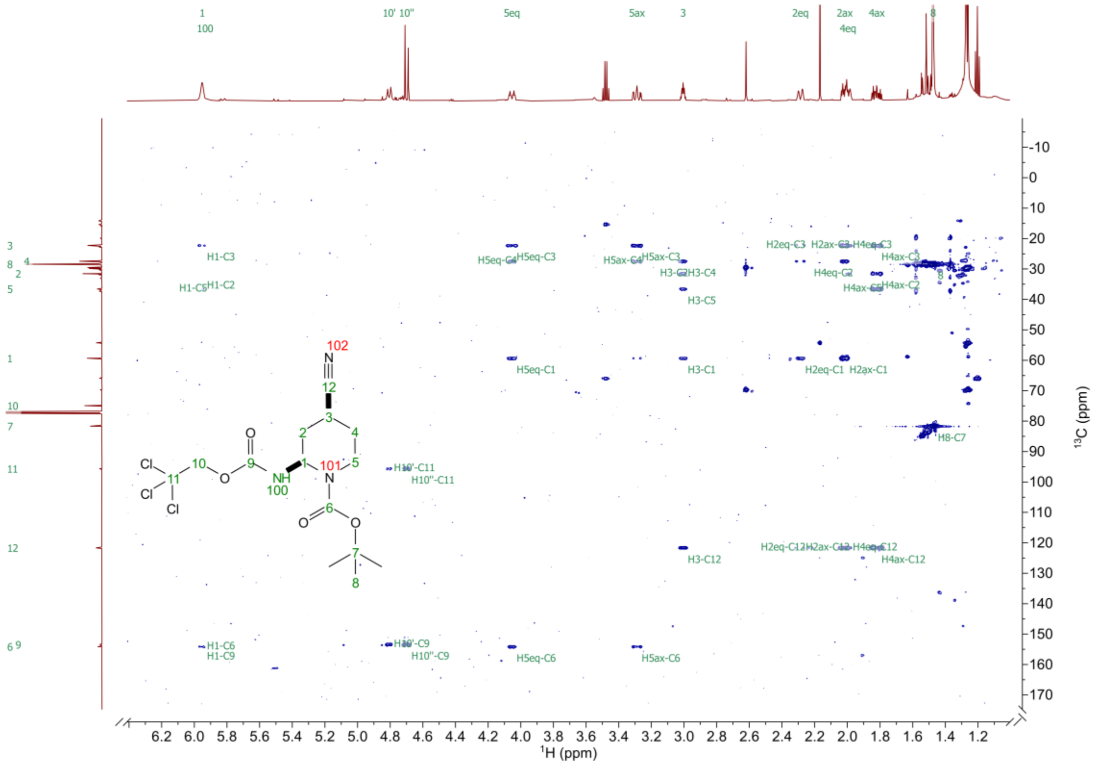

Compound **7l**, minor diastereomer: ^{1}H-^{1}H COSY ($CDCl_3$, 323 K)

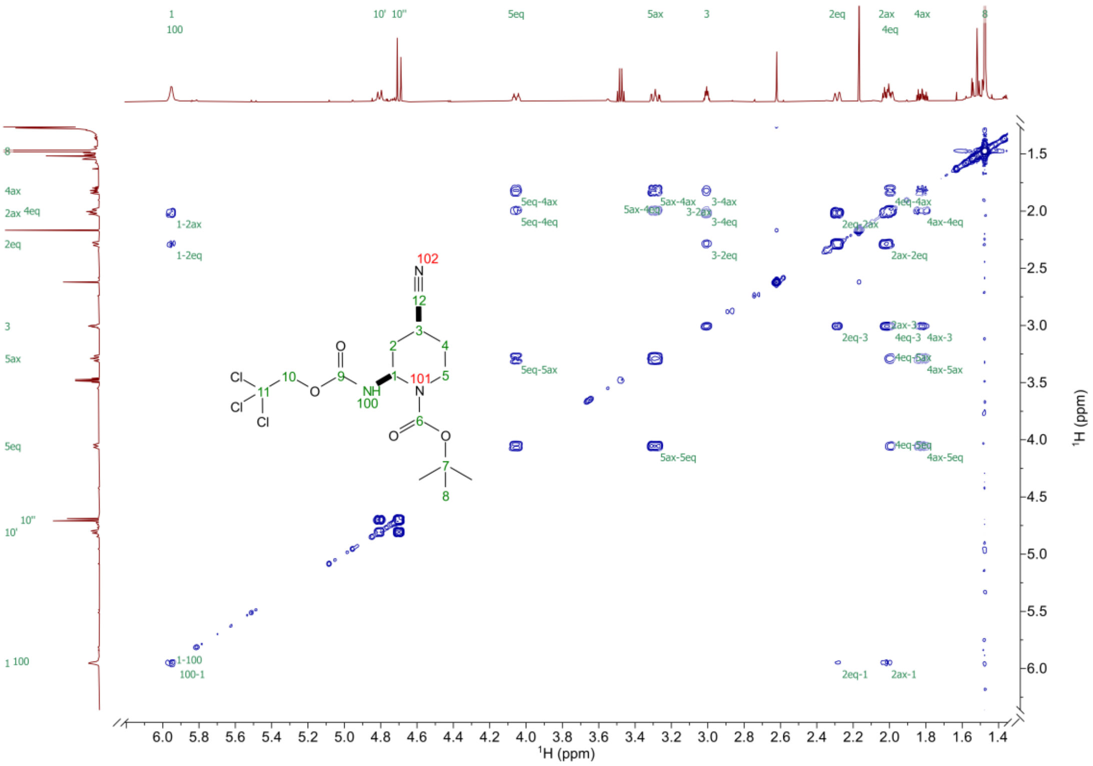

Compound **7l**, minor diastereomer: ^{1}H-^{1}H NOESY ($CDCl_3$, 323 K)

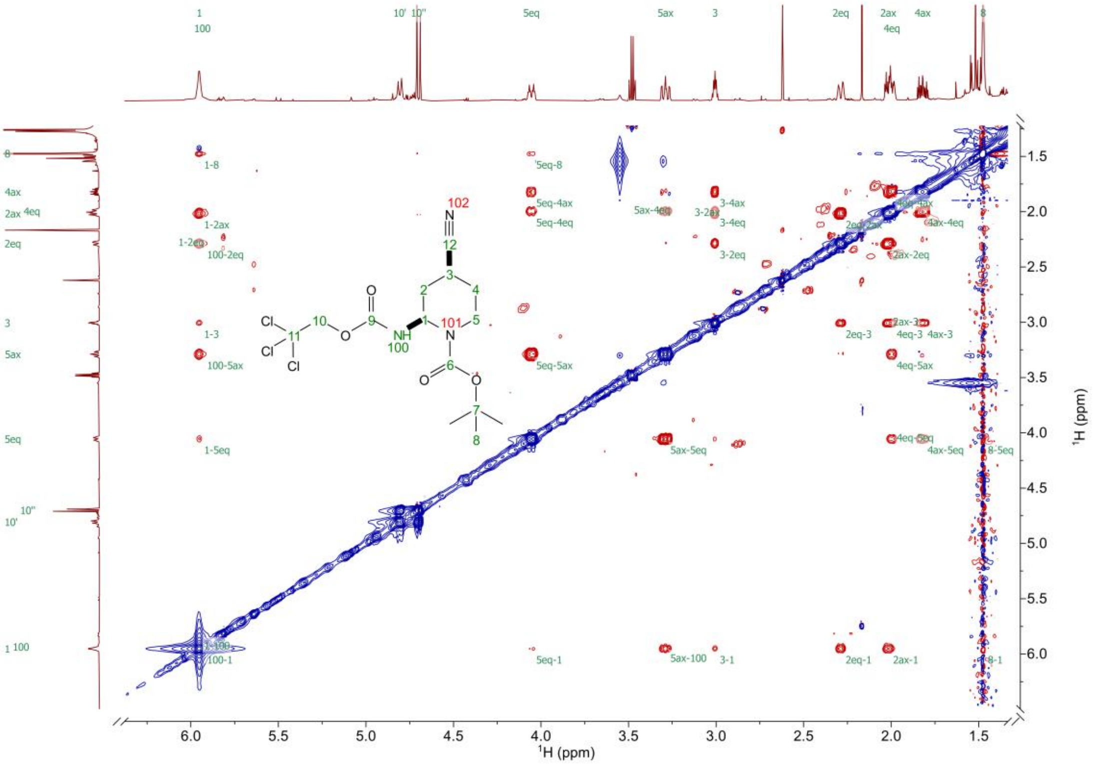

Compound **7l**, minor diastereomer: ^{1}H-^{15}N HMBC ($CDCl_3$, 323 K)

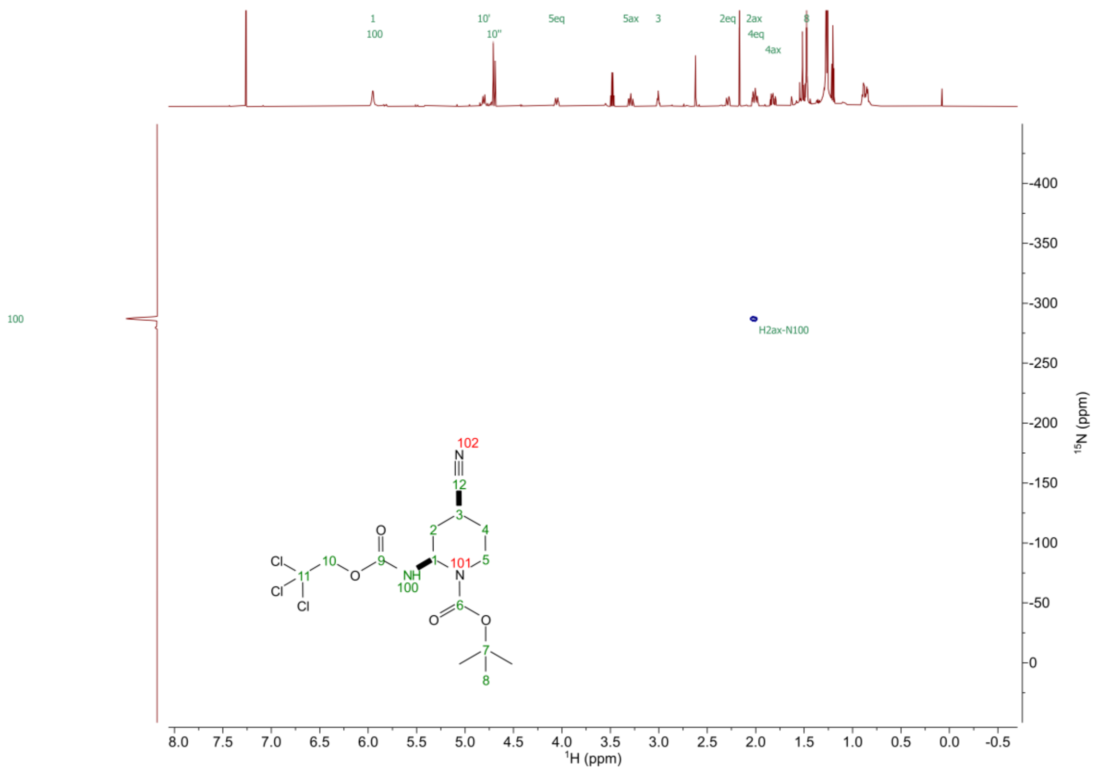

Compound **7m**, major diastereomer: ^{1}H NMR (600 MHz, $CDCl_3$, 323 K)

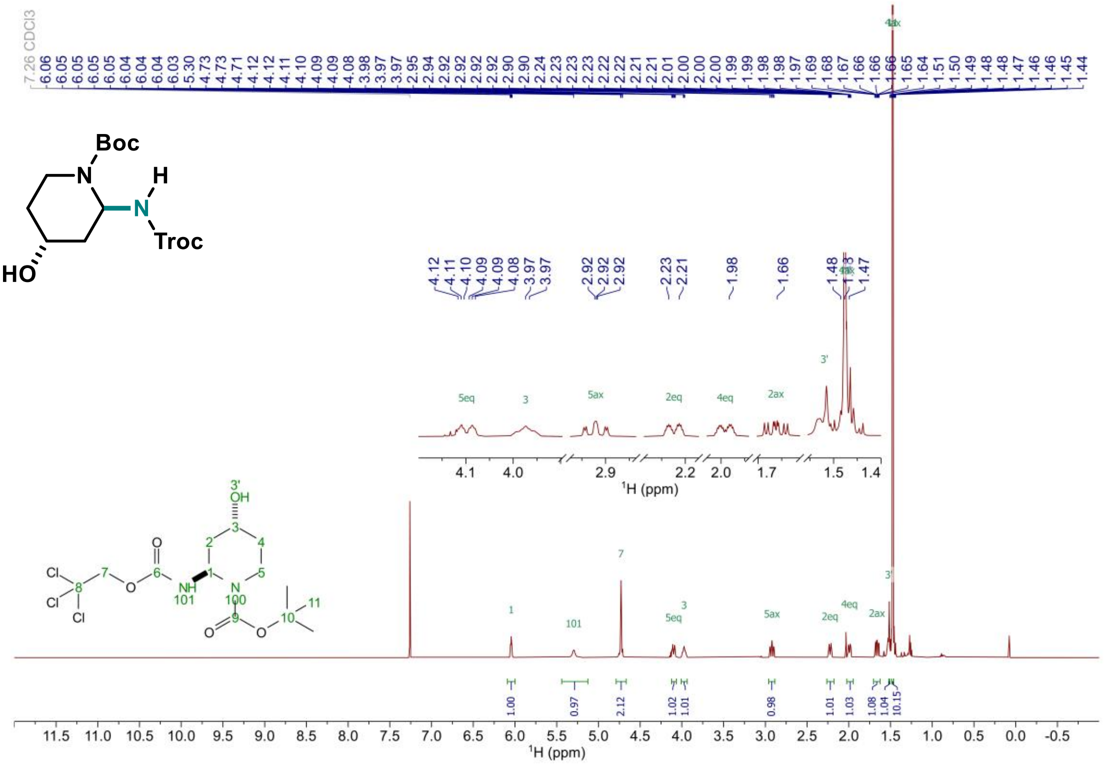

Compound **7m**, major diastereomer: ^{13}C NMR (151 MHz, $CDCl_3$, 323 K)

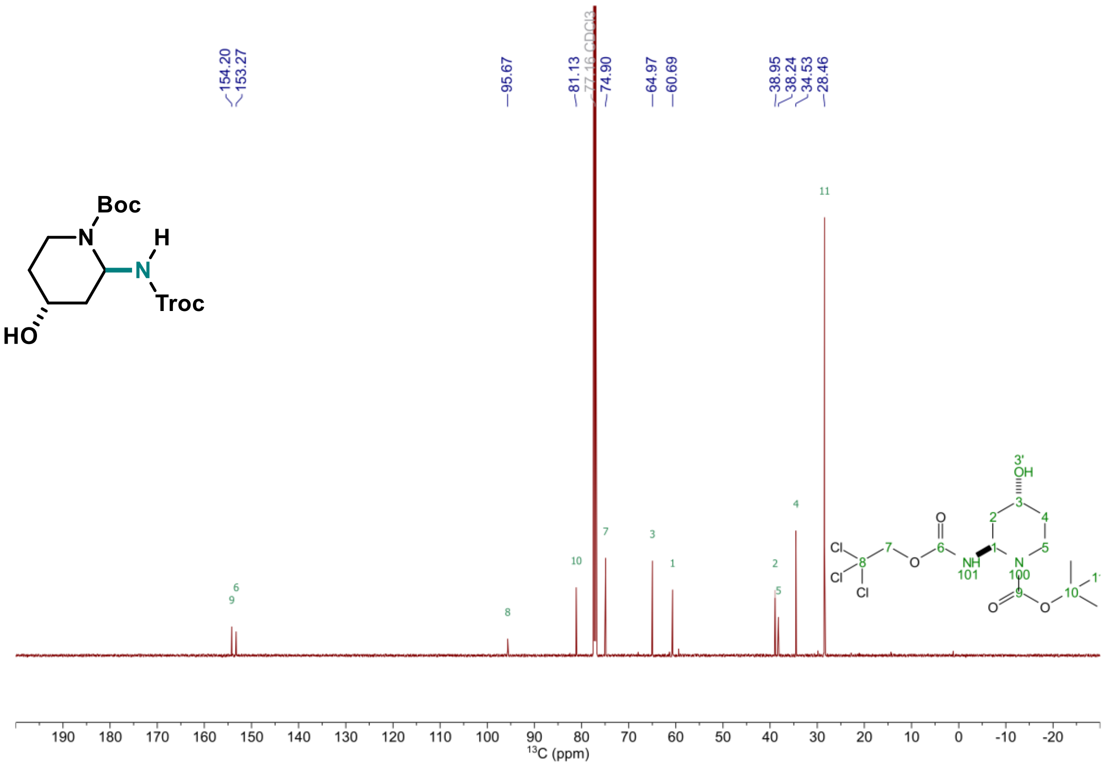

Compound **7m**, major diastereomer: variable temperature ^{1}H NMR (600 MHz, $CDCl_3$)

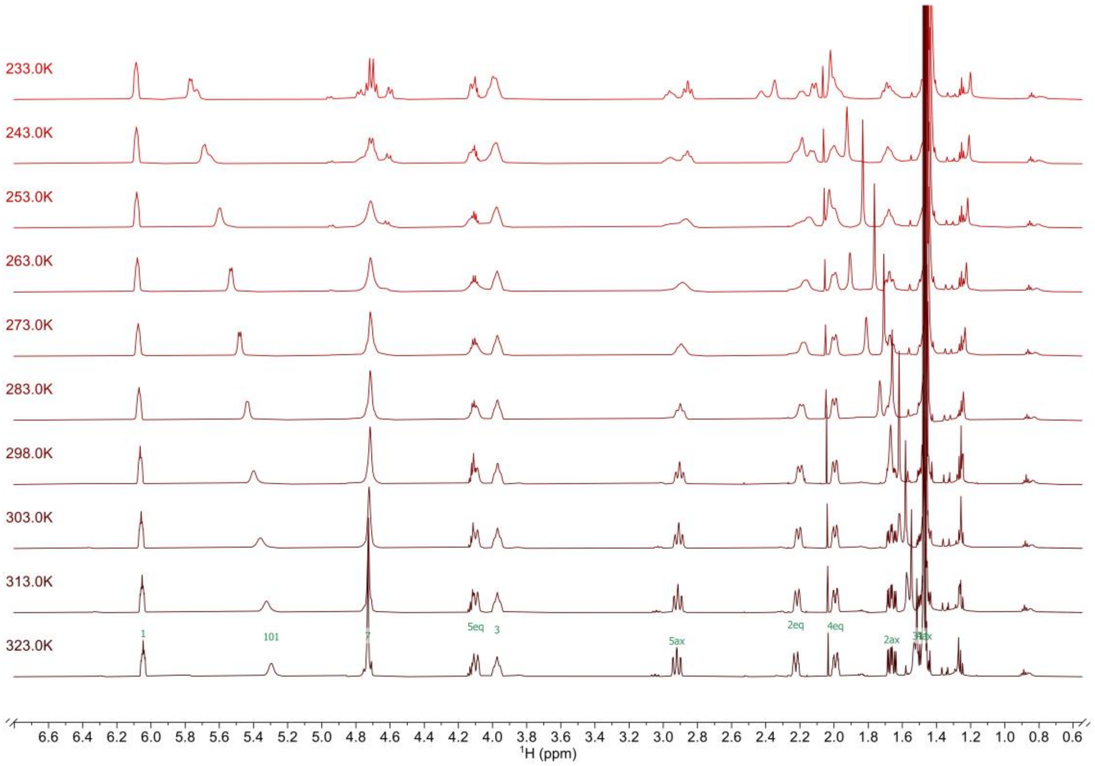

Compound **7m**, major diastereomer: ^{1}H-^{13}C HSQC ($CDCl_3$, 323 K)

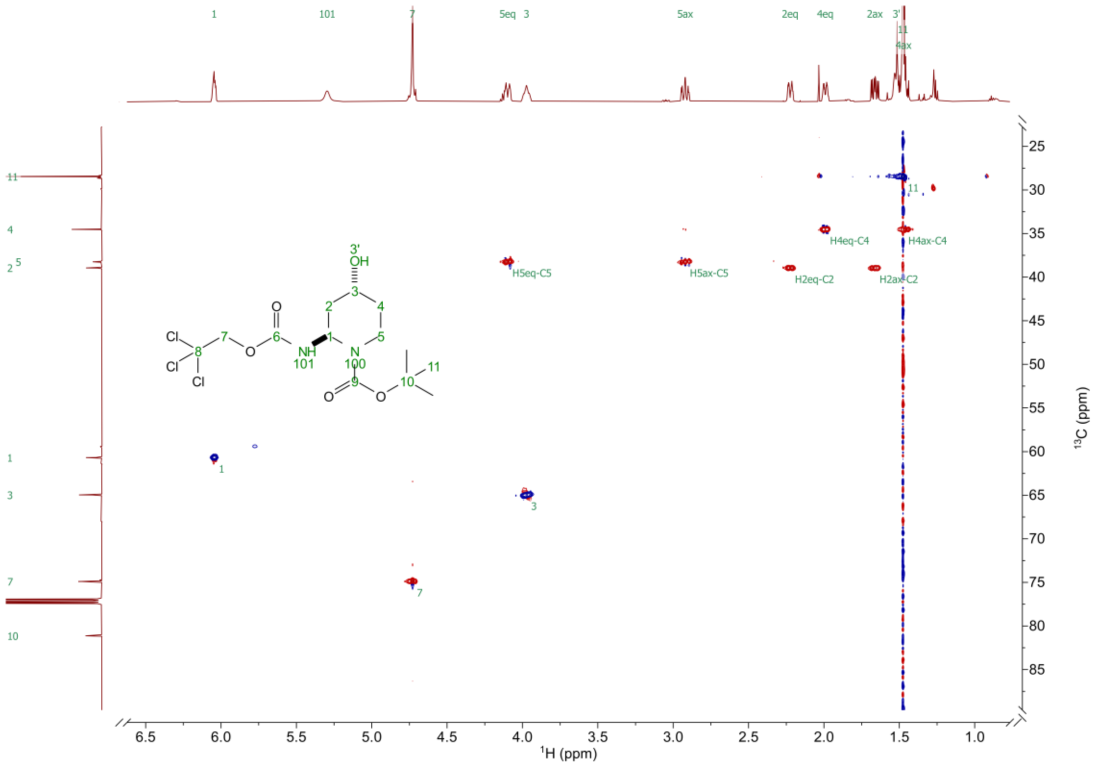

Compound **7m**, major diastereomer: ^{1}H-^{13}C HMBC ($CDCl_3$, 323 K)

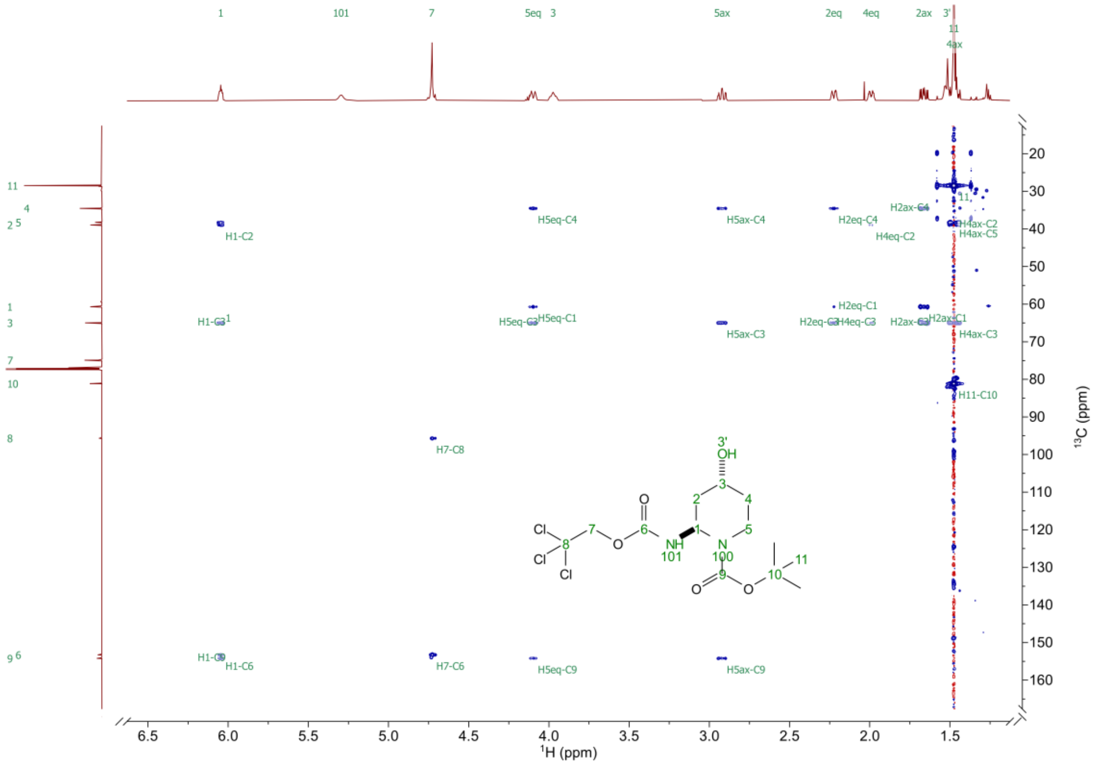

Compound **7m**, major diastereomer: ^{1}H-^{1}H COSY ($CDCl_3$, 323 K)

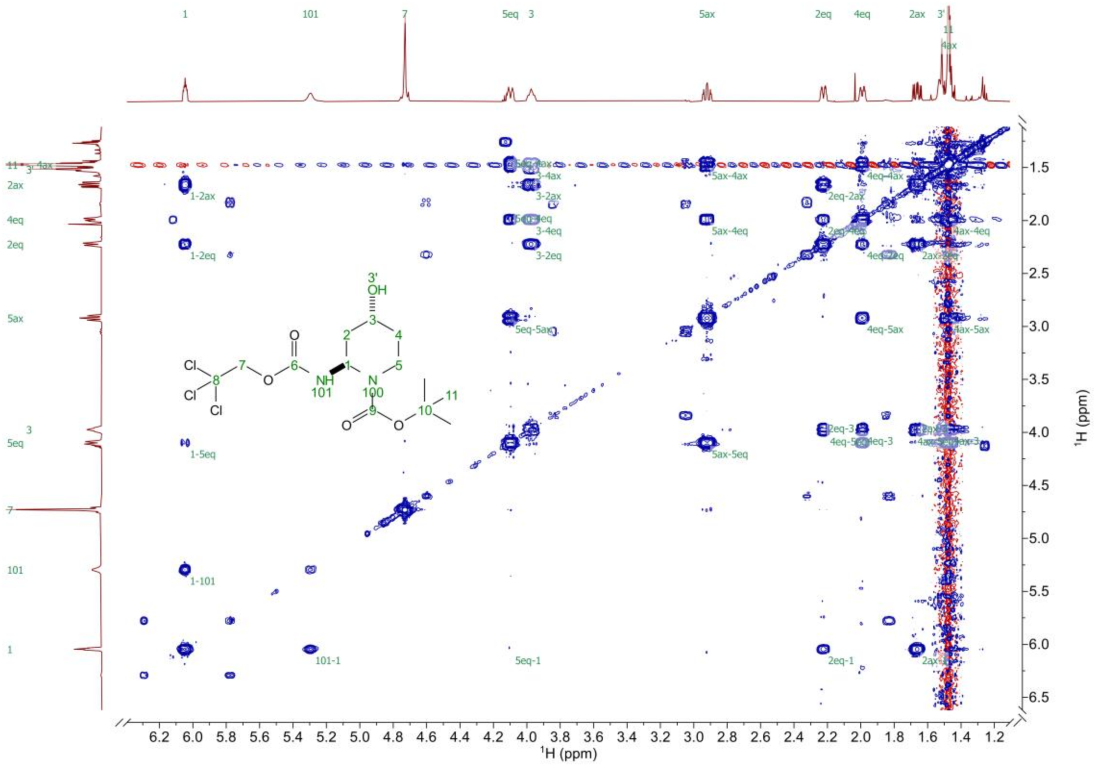

Compound **7m**, major diastereomer: ^{1}H-^{1}H NOESY ($CDCl_3$, 323 K)

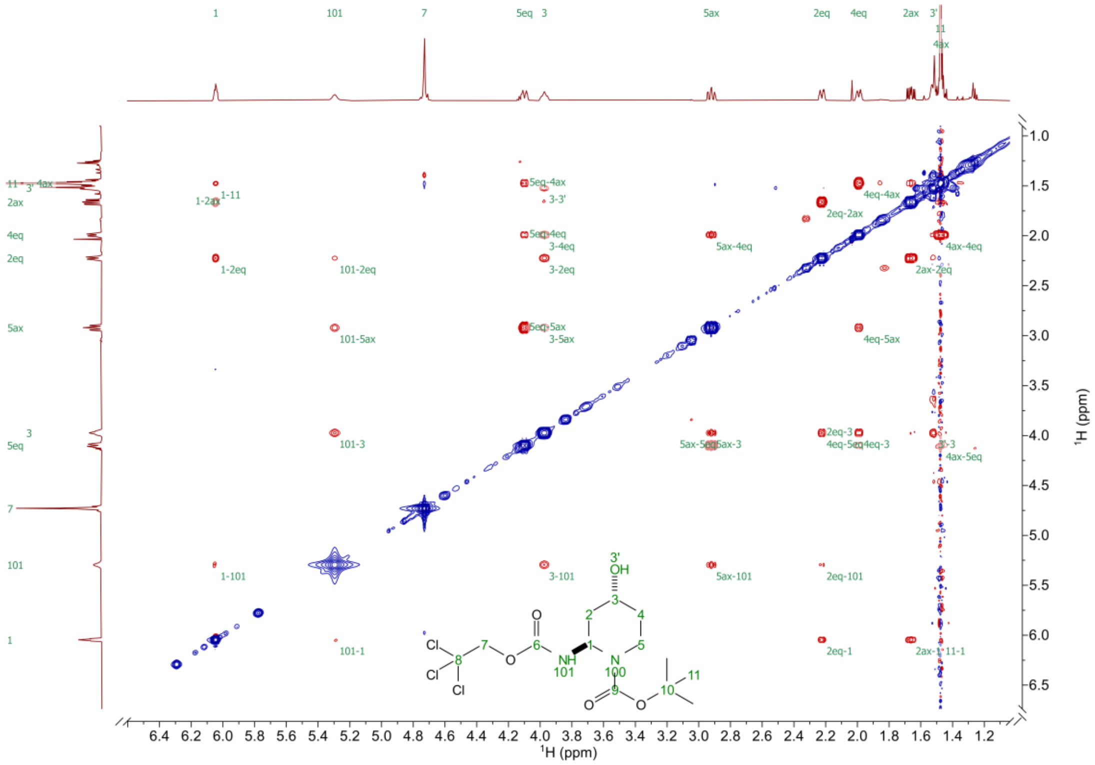

Compound **7m**, major diastereomer: ^{1}H-^{15}N HMBC ($CDCl_3$, 323 K)

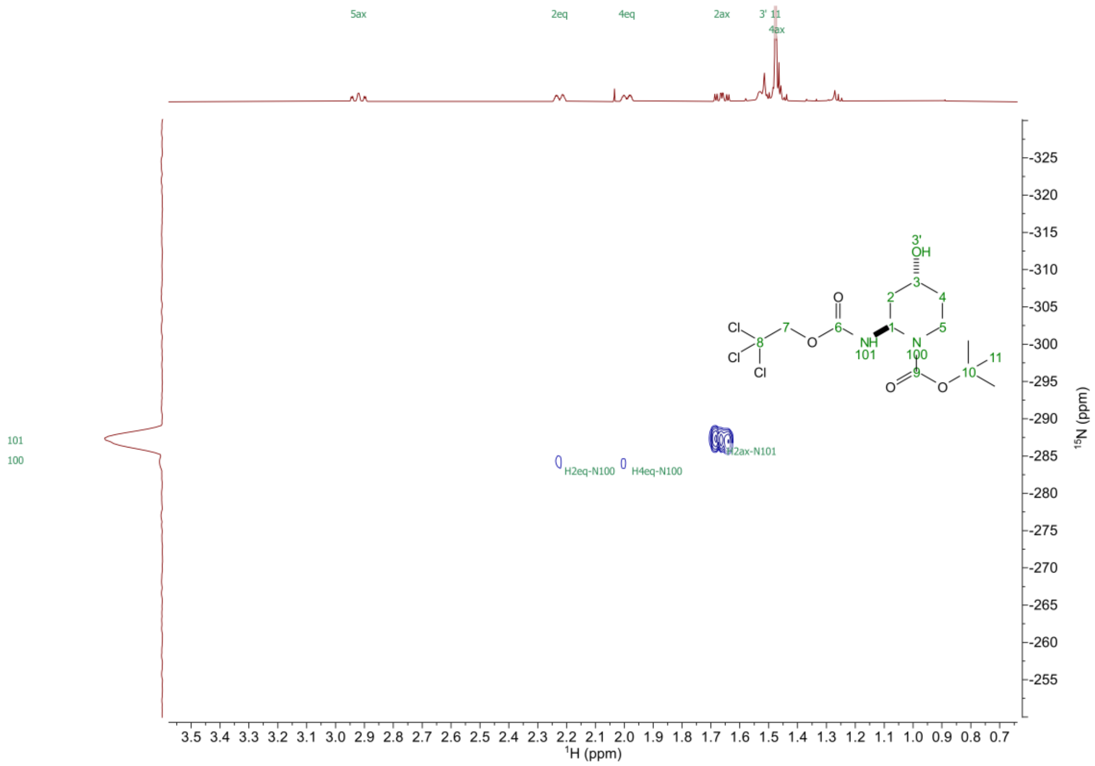

Compound **7m**, minor diastereomer: ^{1}H NMR (600 MHz, $CDCl_3$, 323 K)

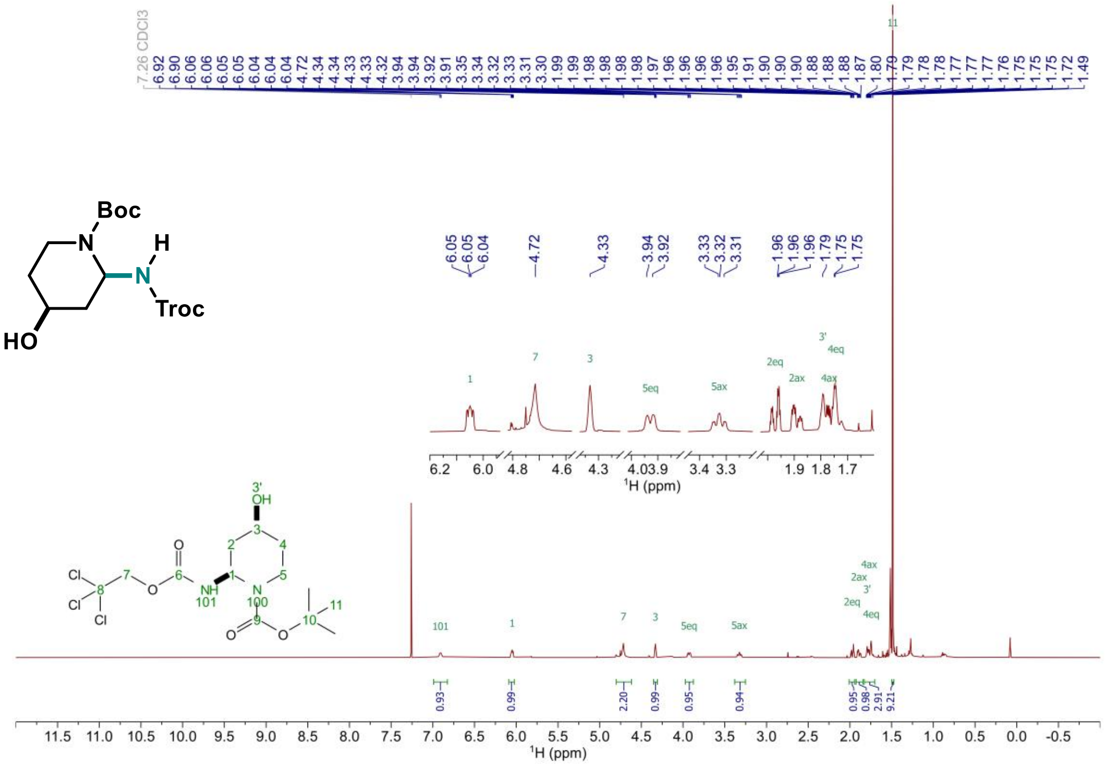

Compound **7m**, minor diastereomer: ^{13}C NMR (151 MHz, $CDCl_3$, 323 K)

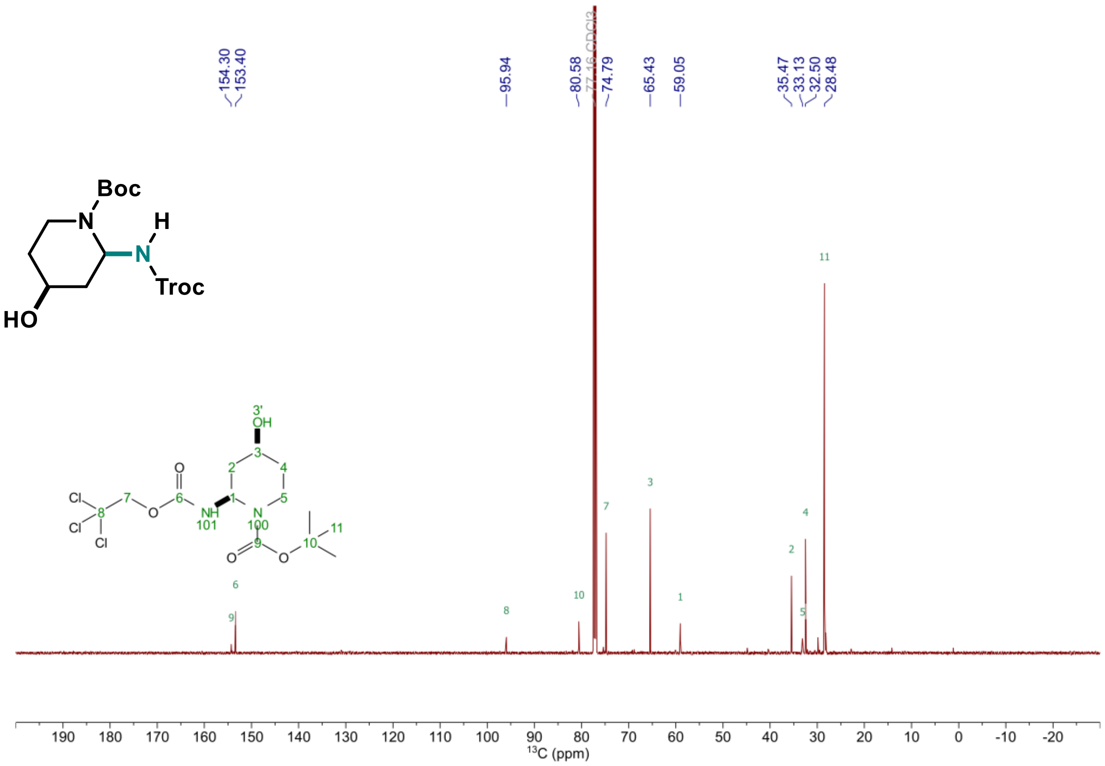

Compound **7m**, minor diastereomer: variable temperature ^{1}H NMR (600 MHz, $CDCl_3$)

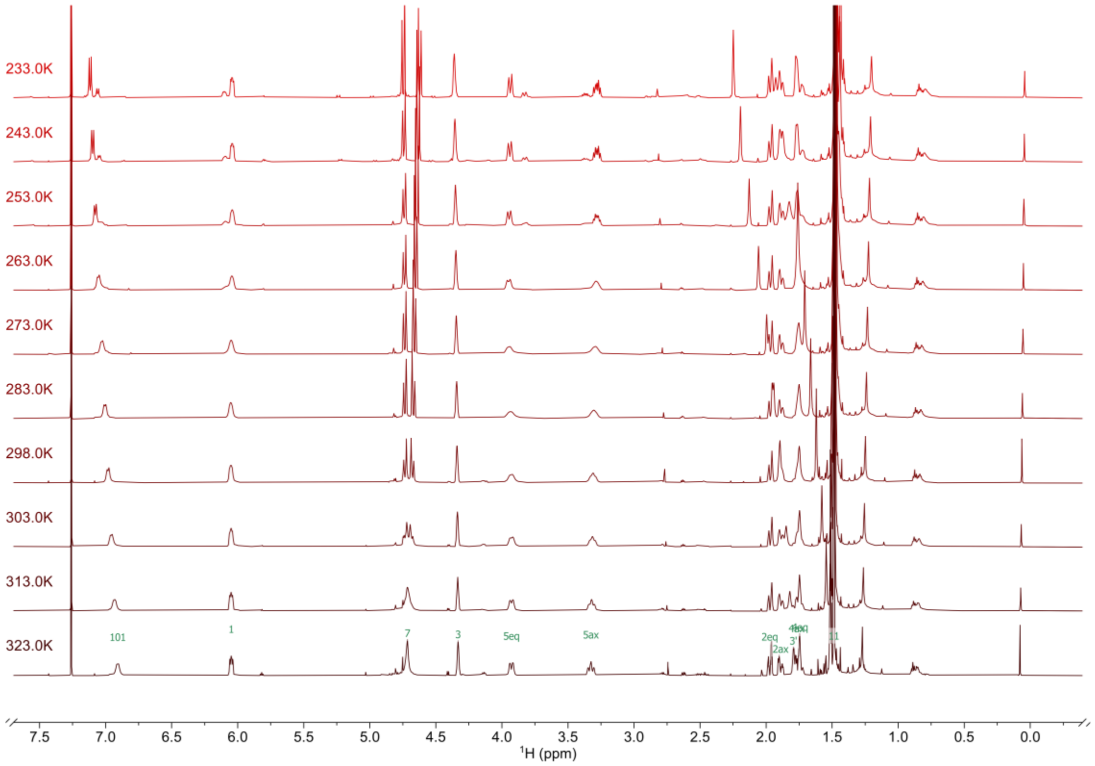

Compound **7m**, minor diastereomer: ^{1}H-^{13}C HSQC ($CDCl_3$, 323 K)

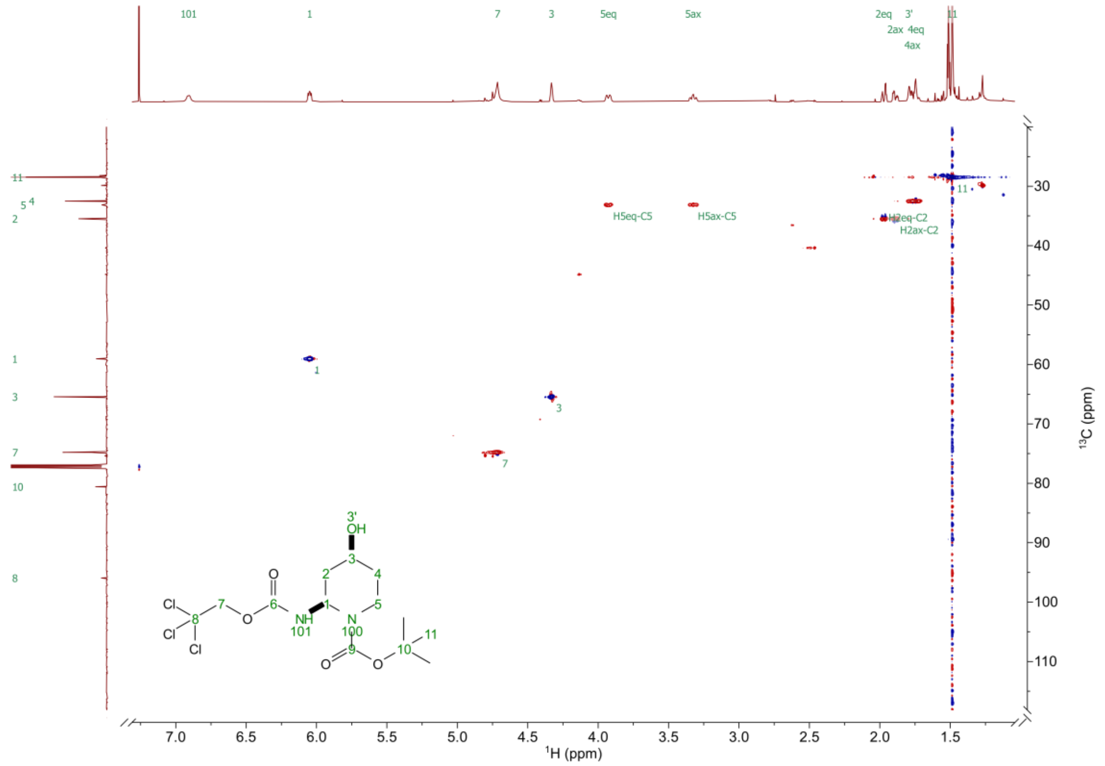

Compound **7m**, minor diastereomer: ^{1}H-^{13}C HMBC ($CDCl_3$, 323 K)

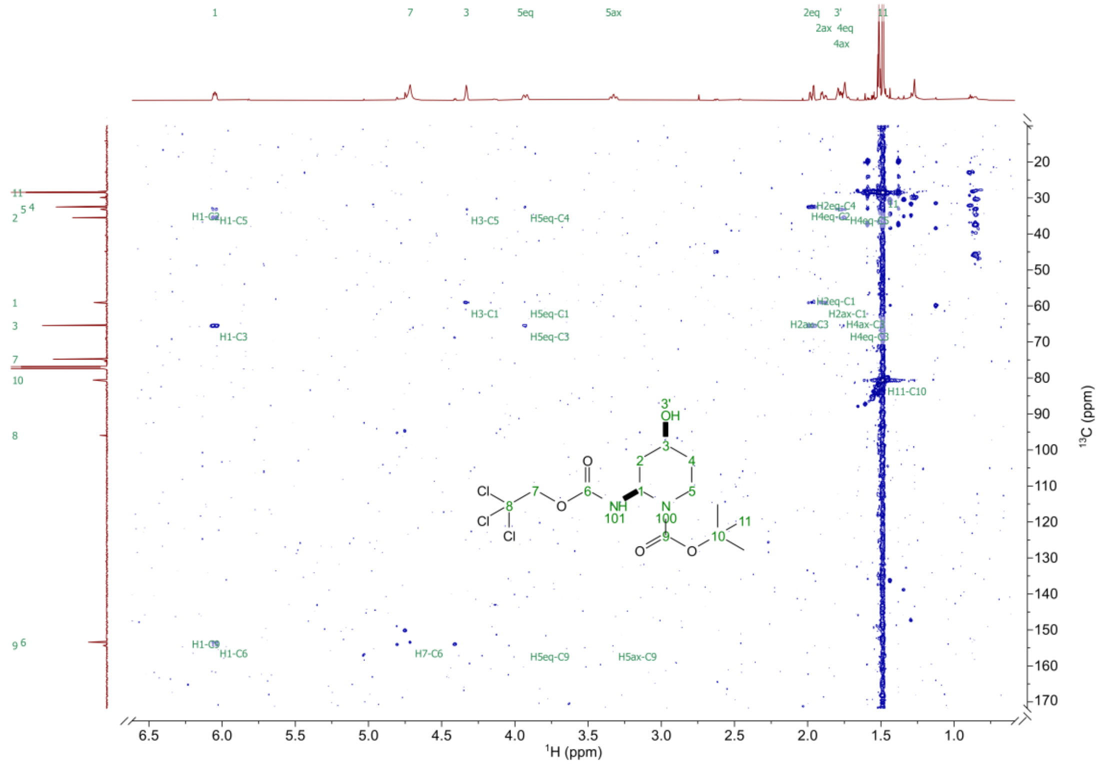

Compound **7m**, minor diastereomer: ^{1}H-^{1}H COSY ($CDCl_3$, 323 K)

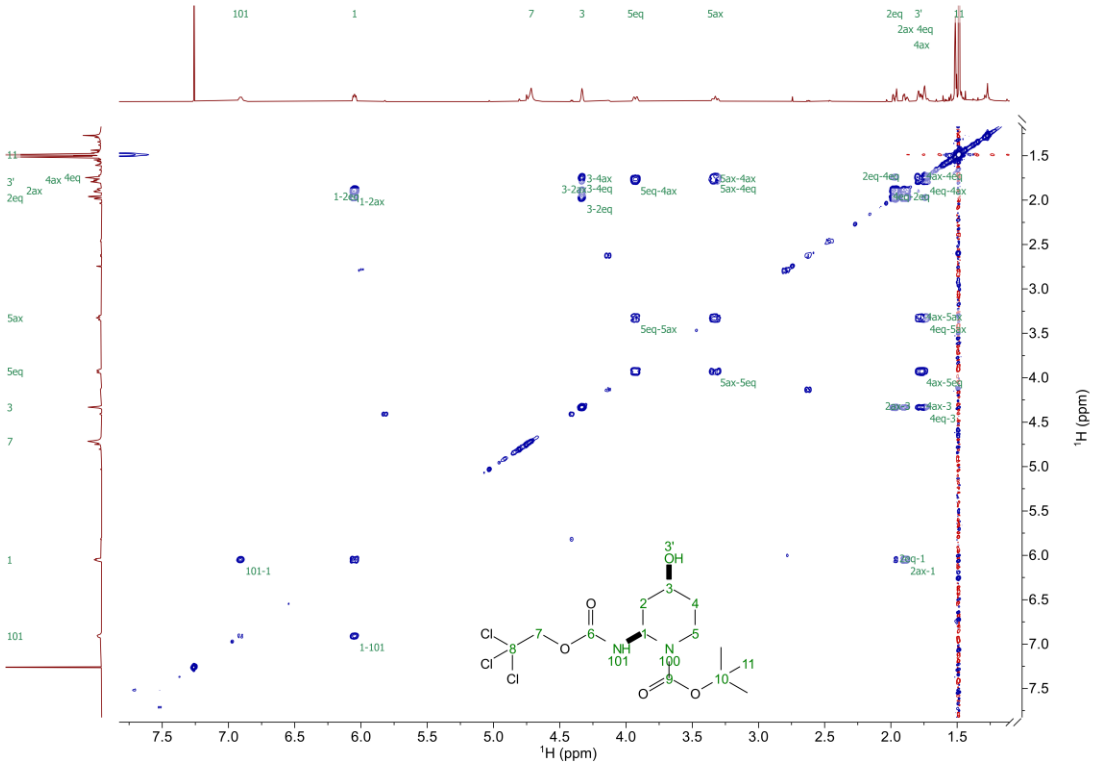

Compound **7m**, minor diastereomer: ^{1}H-^{1}H NOESY ($CDCl_3$, 323 K)

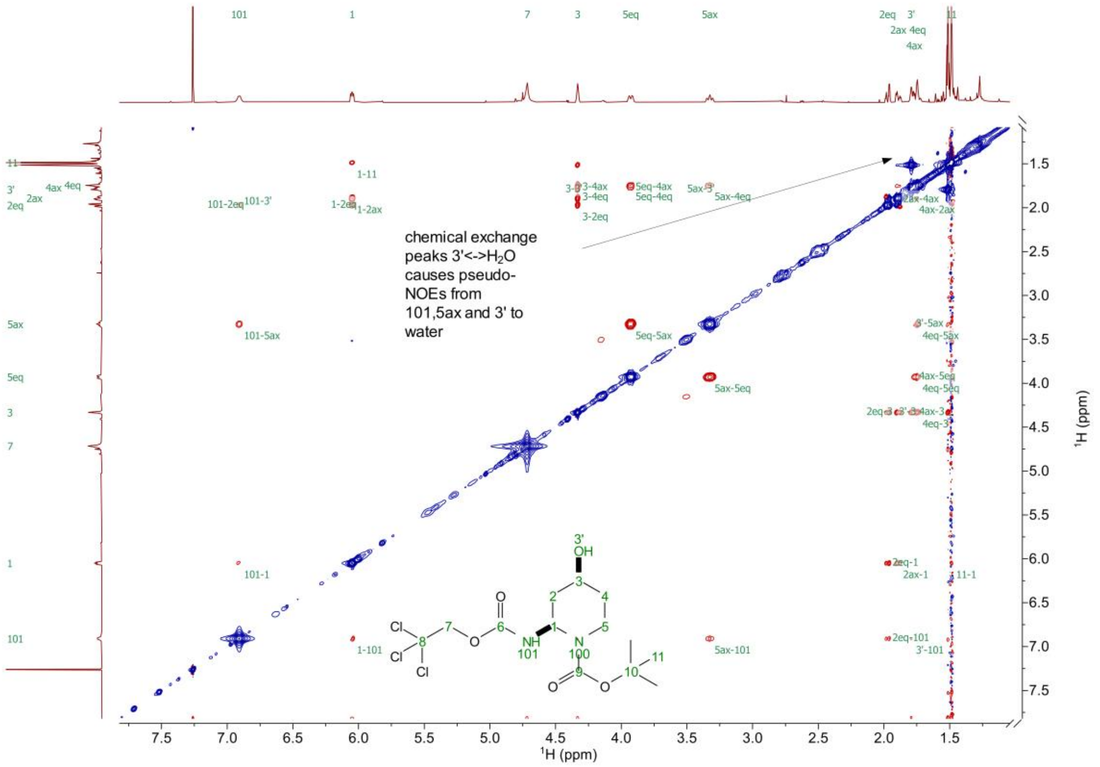

Compound **7m**, minor diastereomer: ^1H-^{15}N HMBC ($CDCl_3$, 323 K)

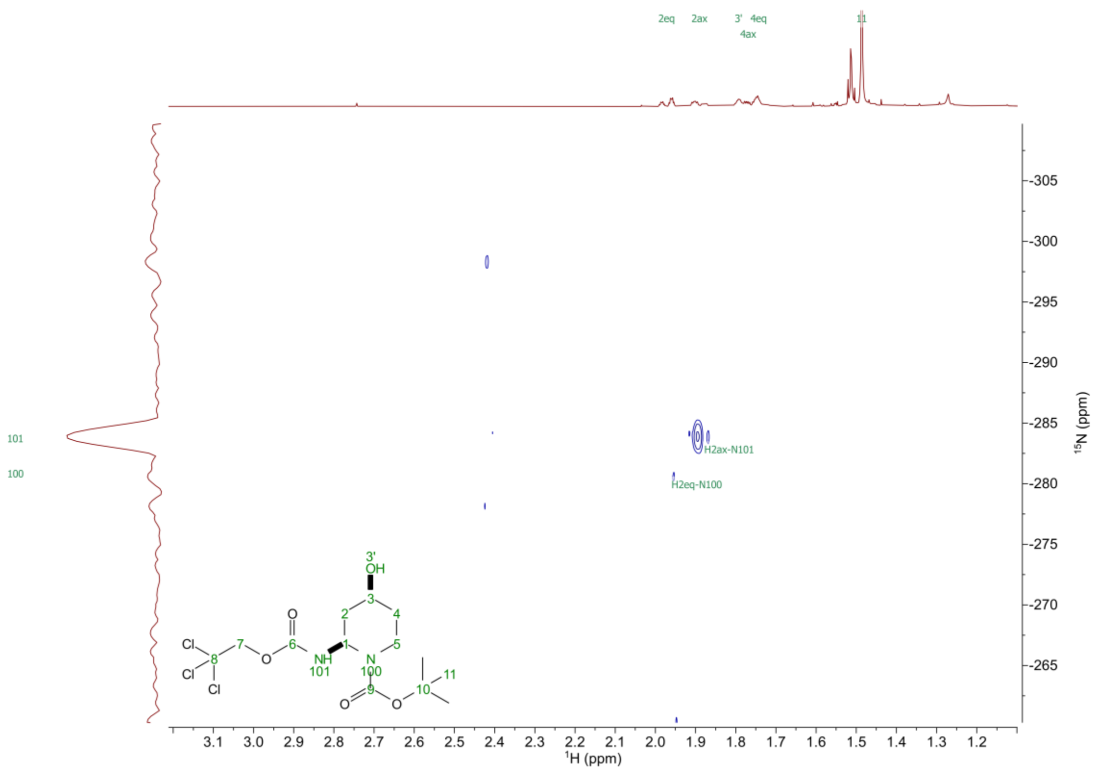

Compound **7n**: ^1H NMR (600 MHz, $CDCl_3$, 323 K)

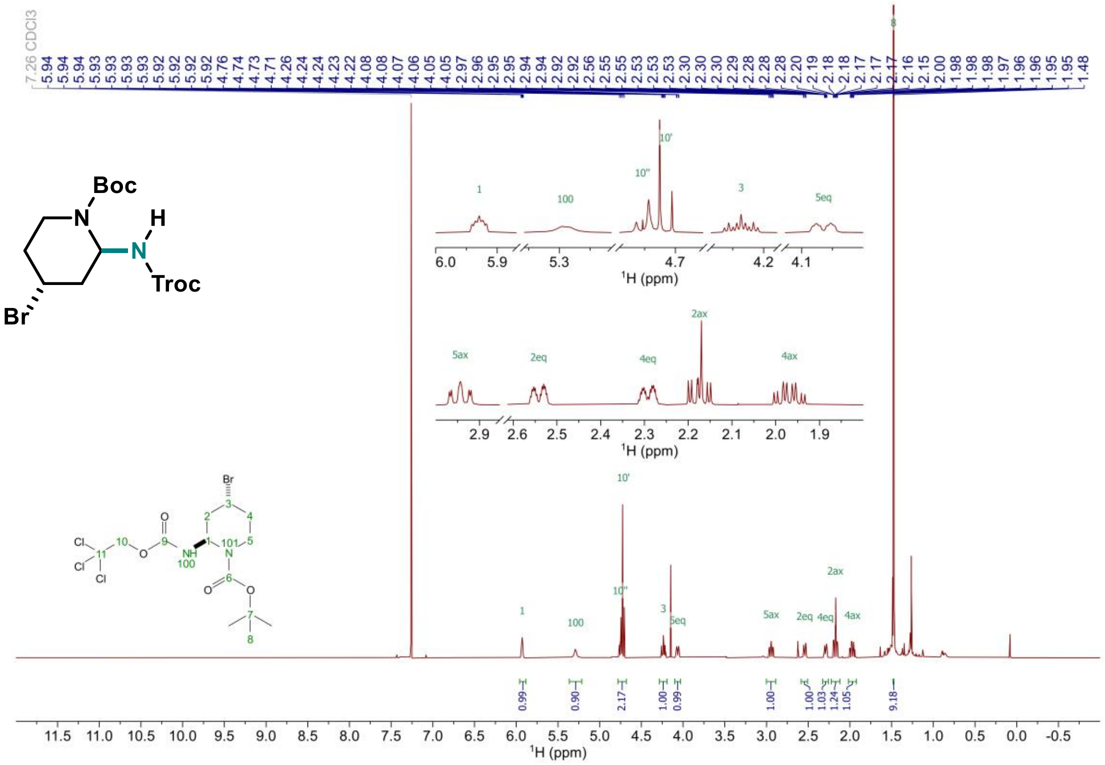

Compound **7n**: ^{13}C NMR (151 MHz, $CDCl_3$, 323 K)

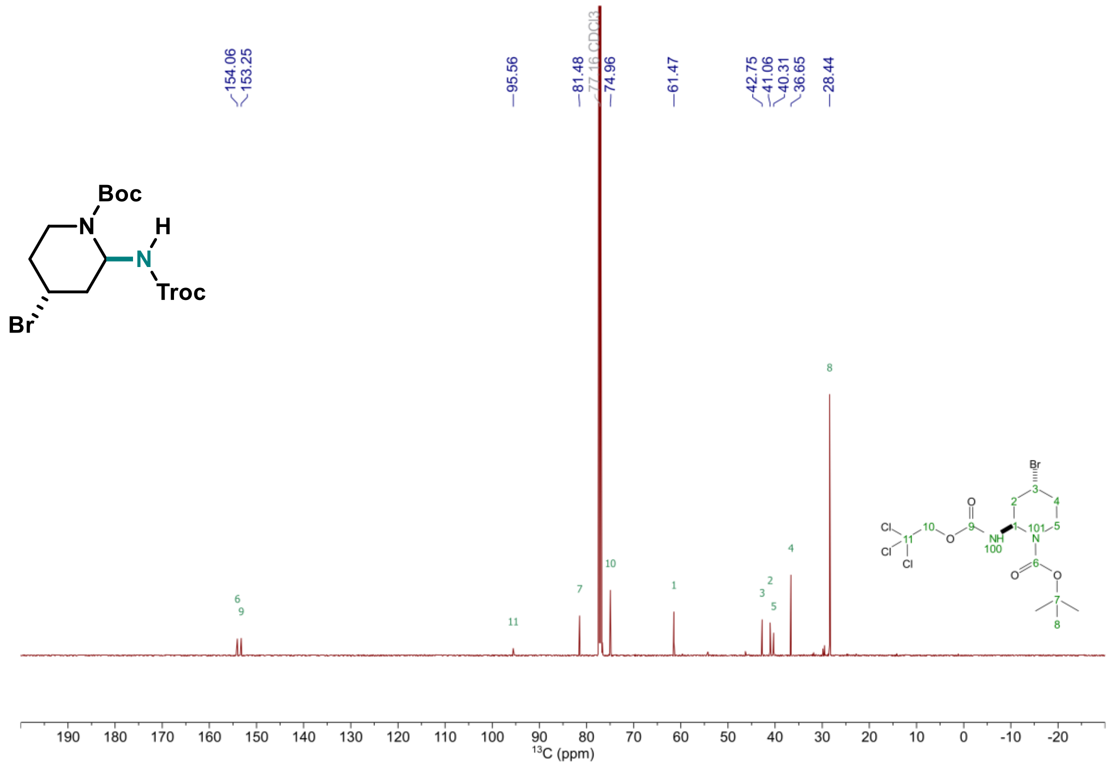

Compound **7n**: variable temperature ^1H NMR (600 MHz, $CDCl_3$)

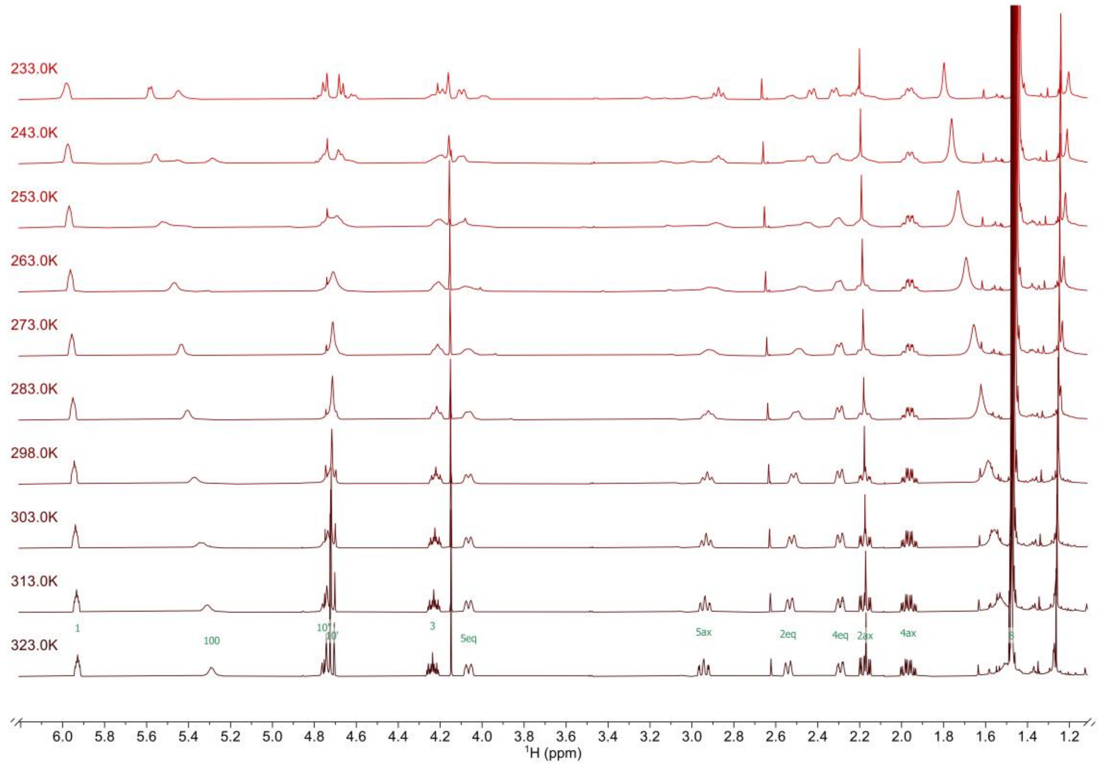

Compound **7n**: ^{1}H-^{13}C HSQC ($CDCl_3$, 323 K)

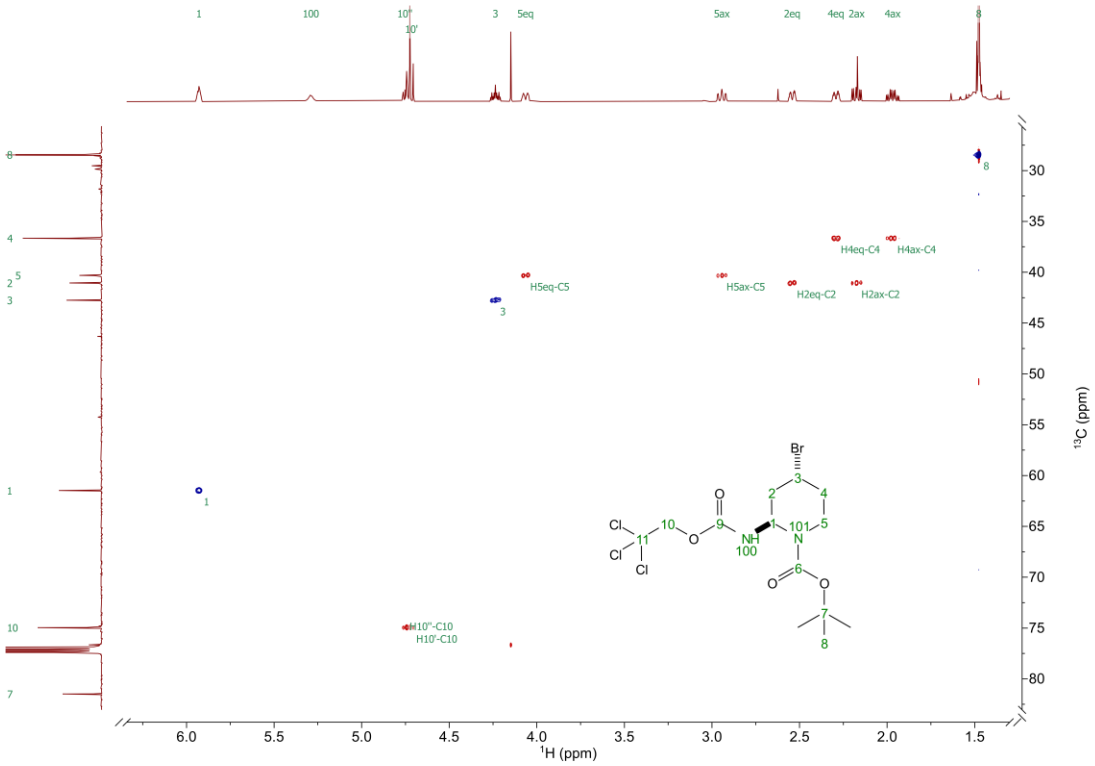

Compound **7n**: ^{1}H-^{13}C HMBC ($CDCl_3$, 323 K)

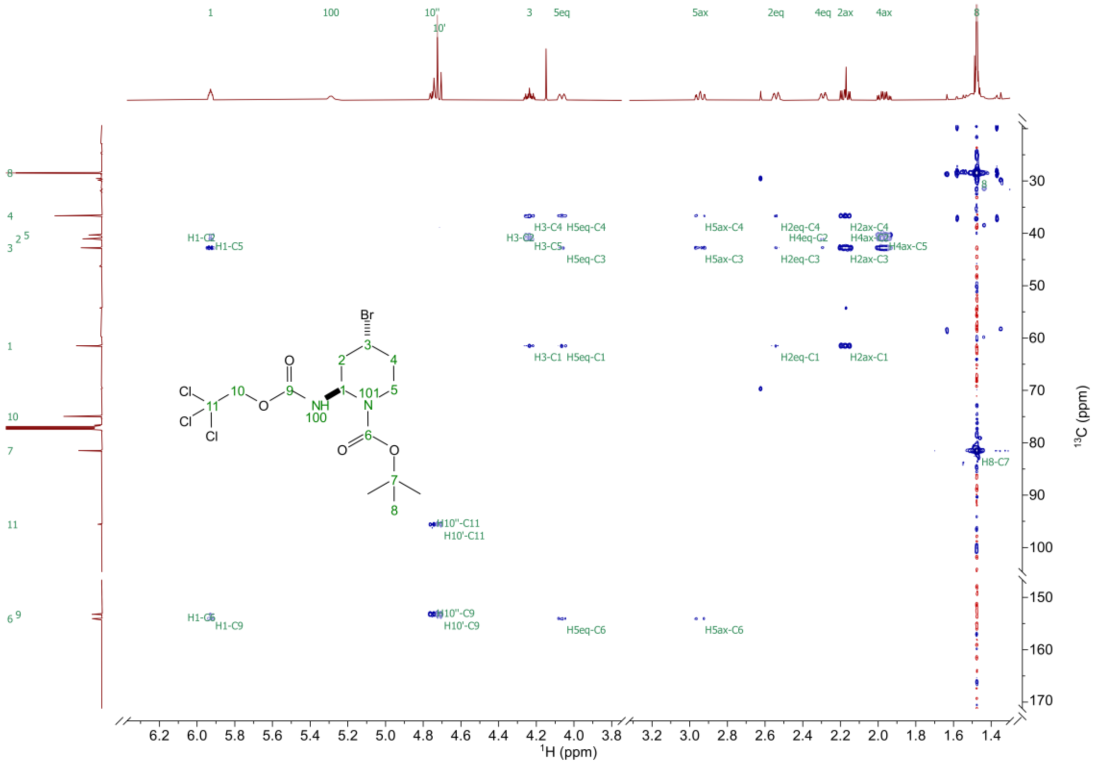

Compound **7n**: ^{1}H-^{1}H COSY (CDCl$_3$, 323 K)

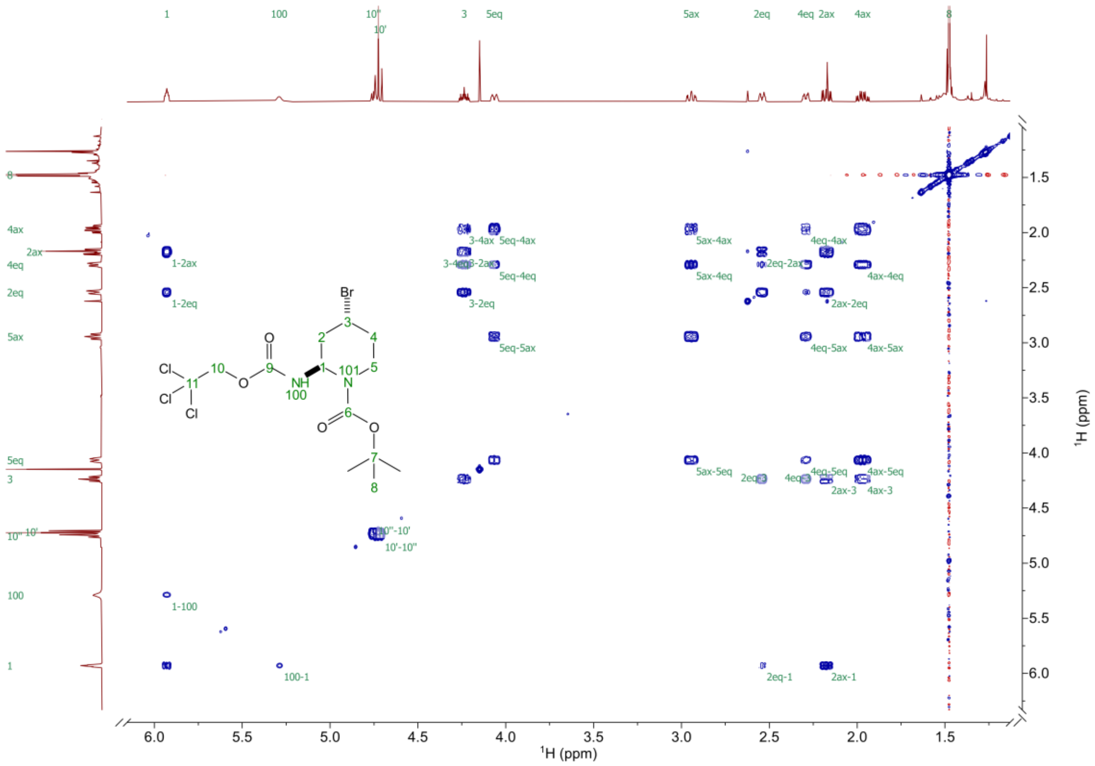

Compound **7n**: ^{1}H-^{1}H NOESY (CDCl$_3$, 323 K)

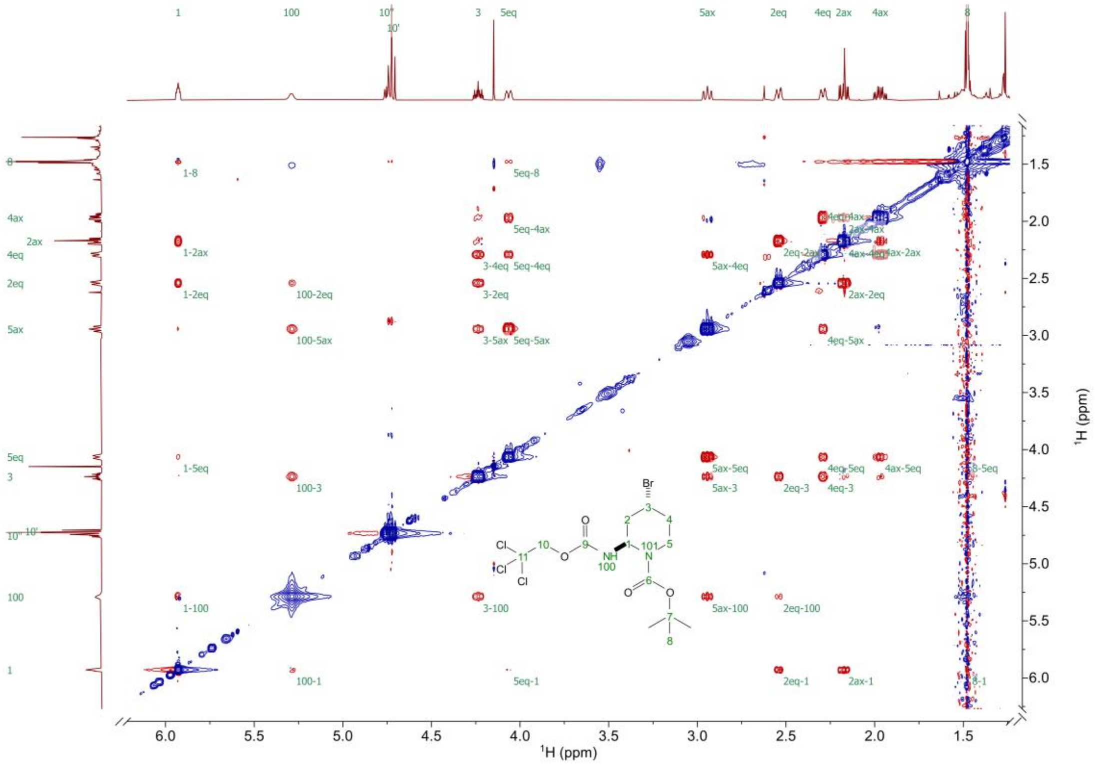

Compound **7n**: ^{1}H-^{15}N HMBC ($CDCl_3$, 323 K)

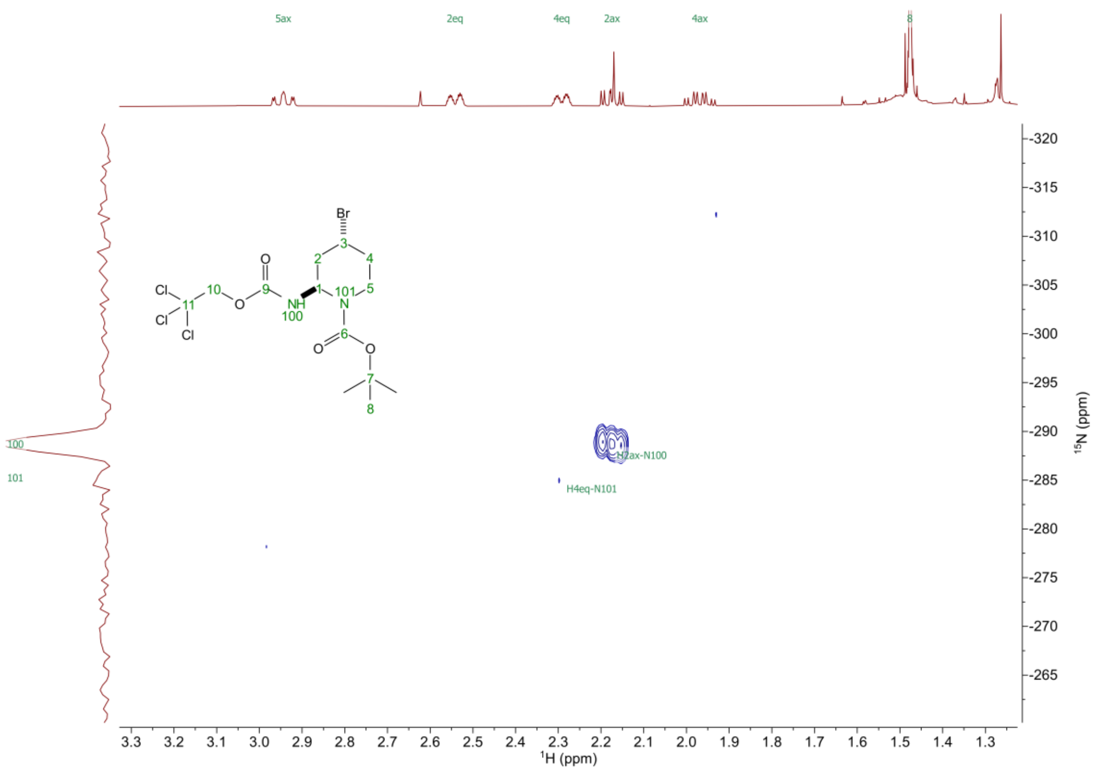

Compound **7o**: ^{1}H NMR (600 MHz, $CDCl_3$, 333 K)

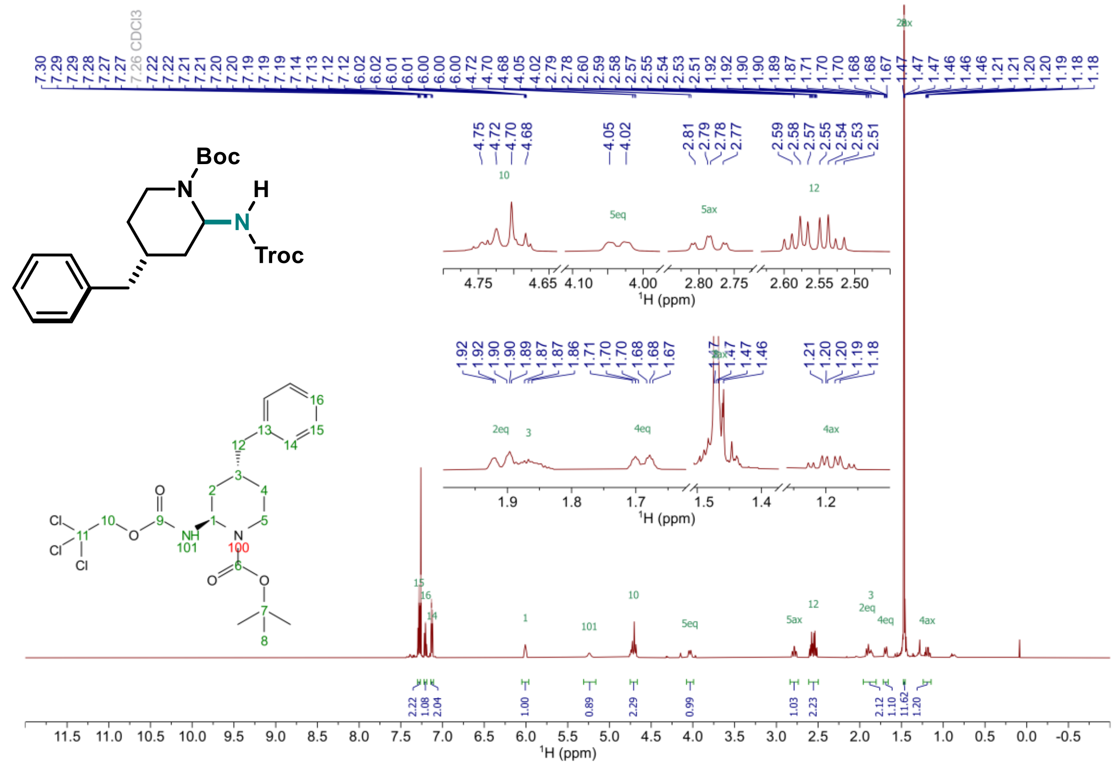

Compound **7o**: ^{13}C NMR (151 MHz, $CDCl_3$, 333 K)

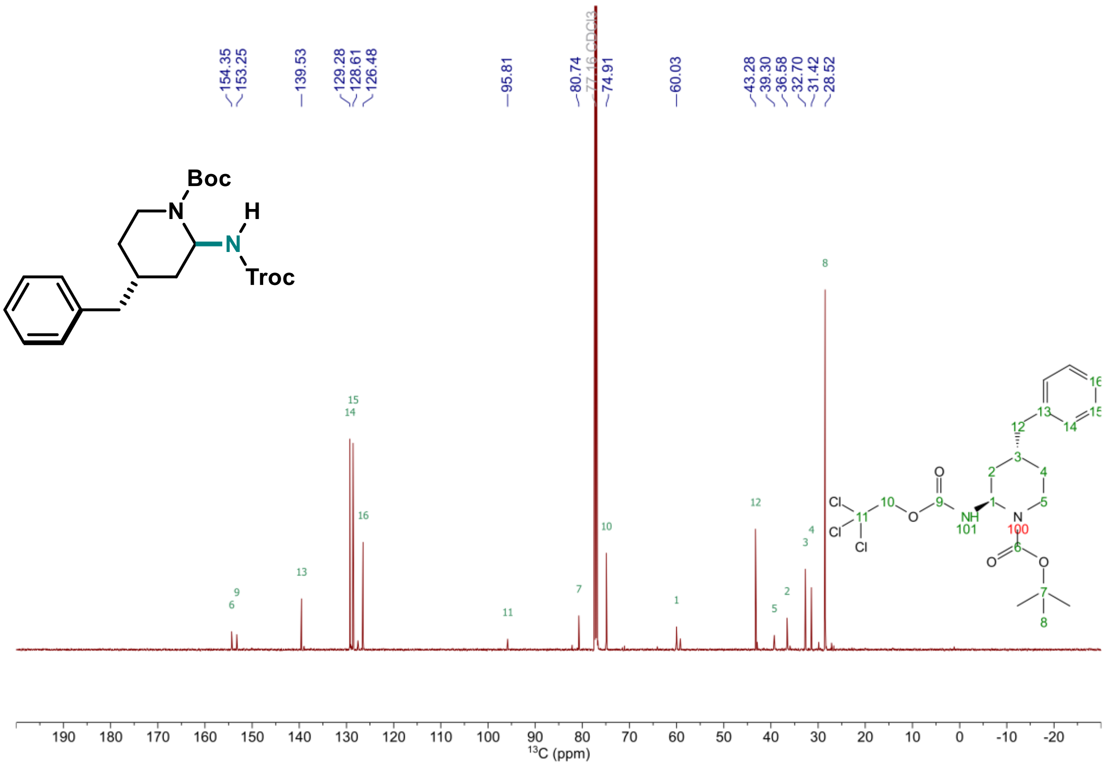

Compound **7o**: variable temperature ^{1}H NMR (600 MHz, $CDCl_3$)

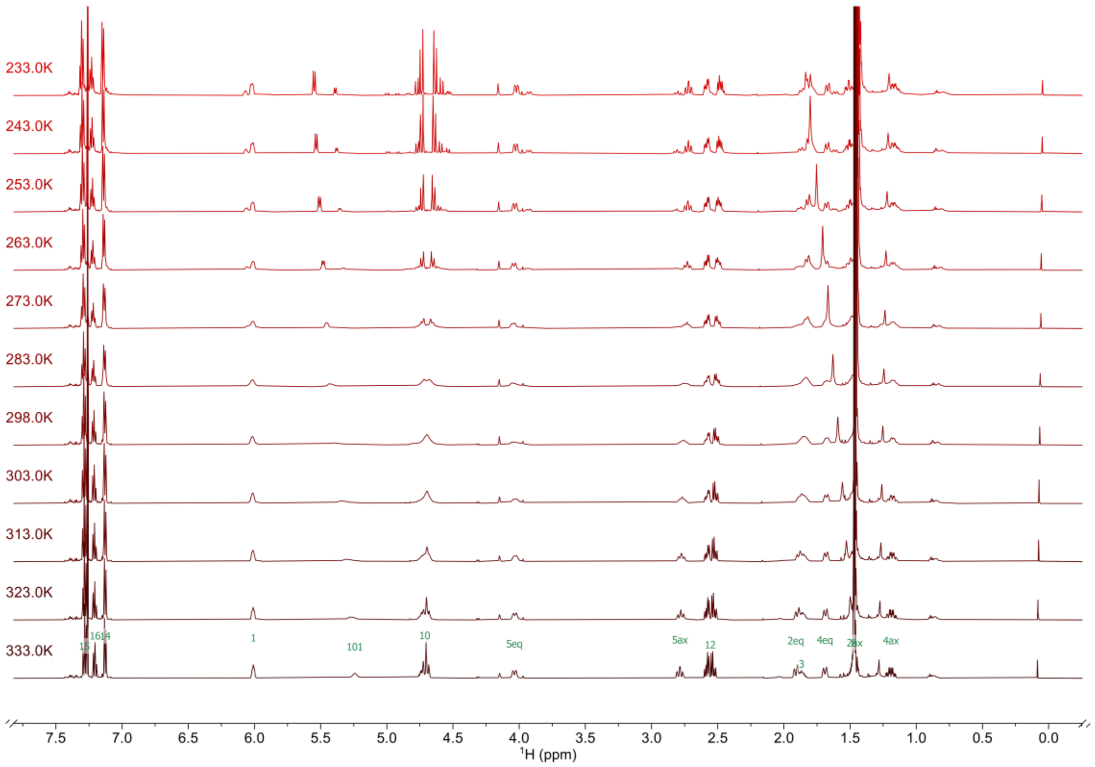

Compound **7o**: ^{1}H-^{13}C HSQC ($CDCl_3$, 333 K)

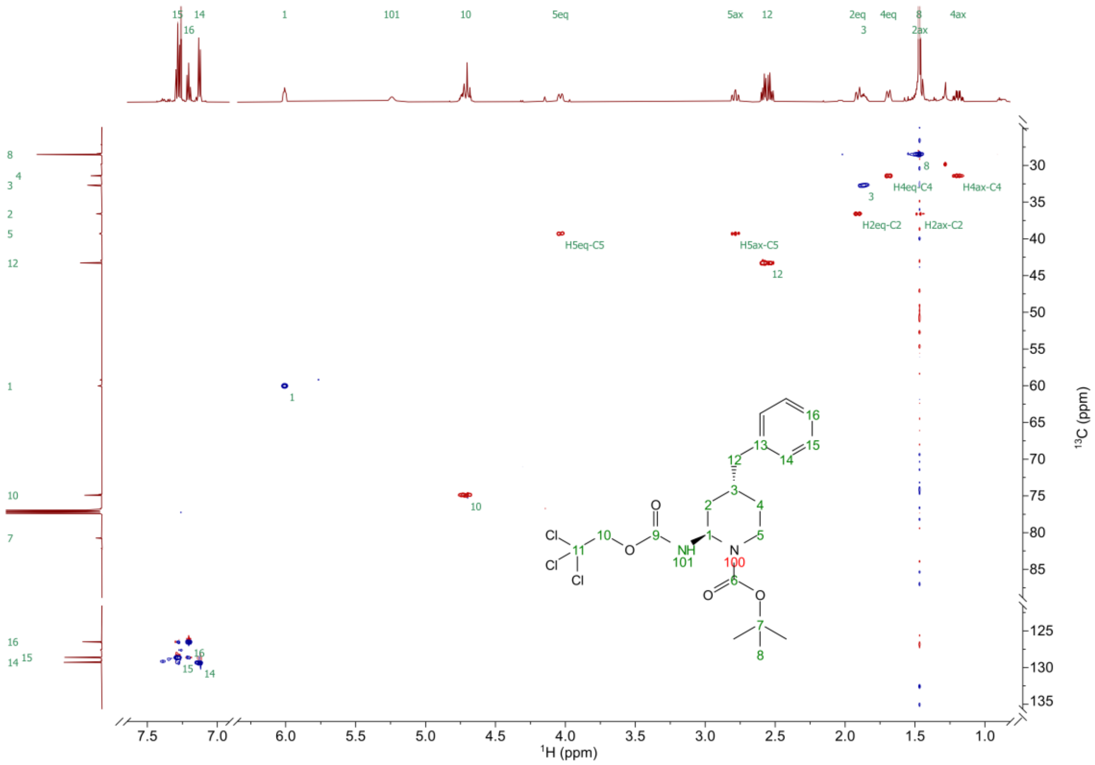

Compound **7o**: ^{1}H-^{13}C HMBC ($CDCl_3$, 333 K)

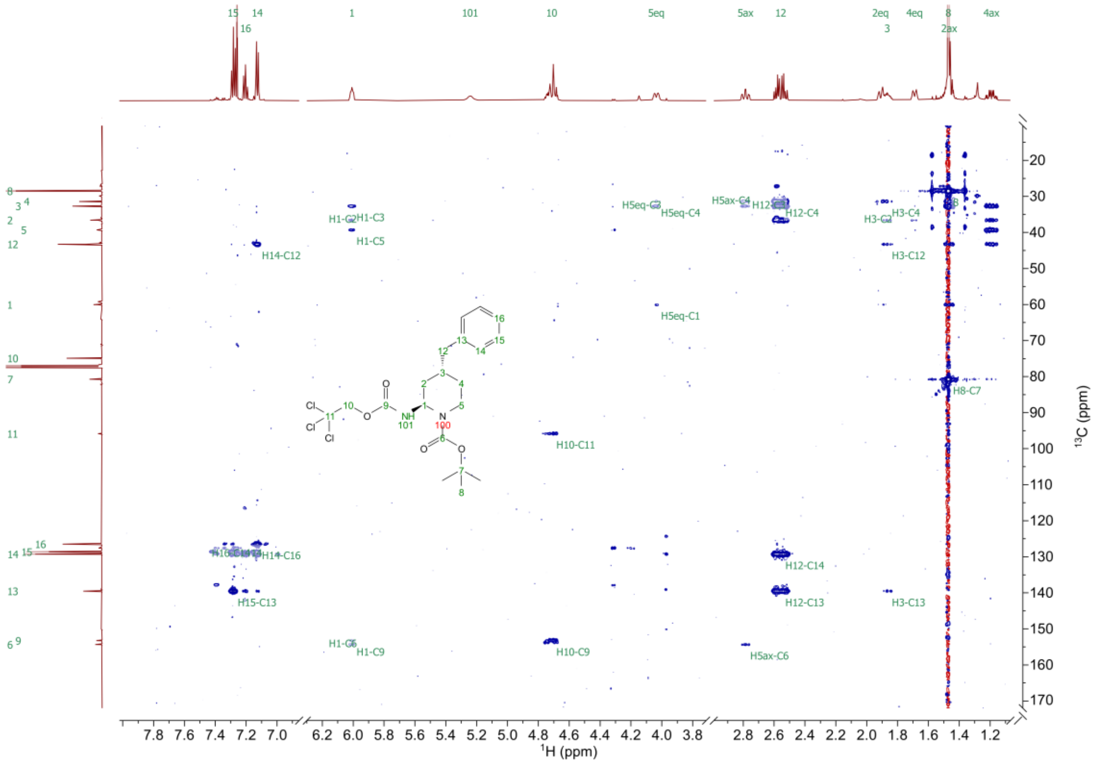

Compound **7o**: ^{1}H-^{1}H COSY ($CDCl_3$, 333 K)

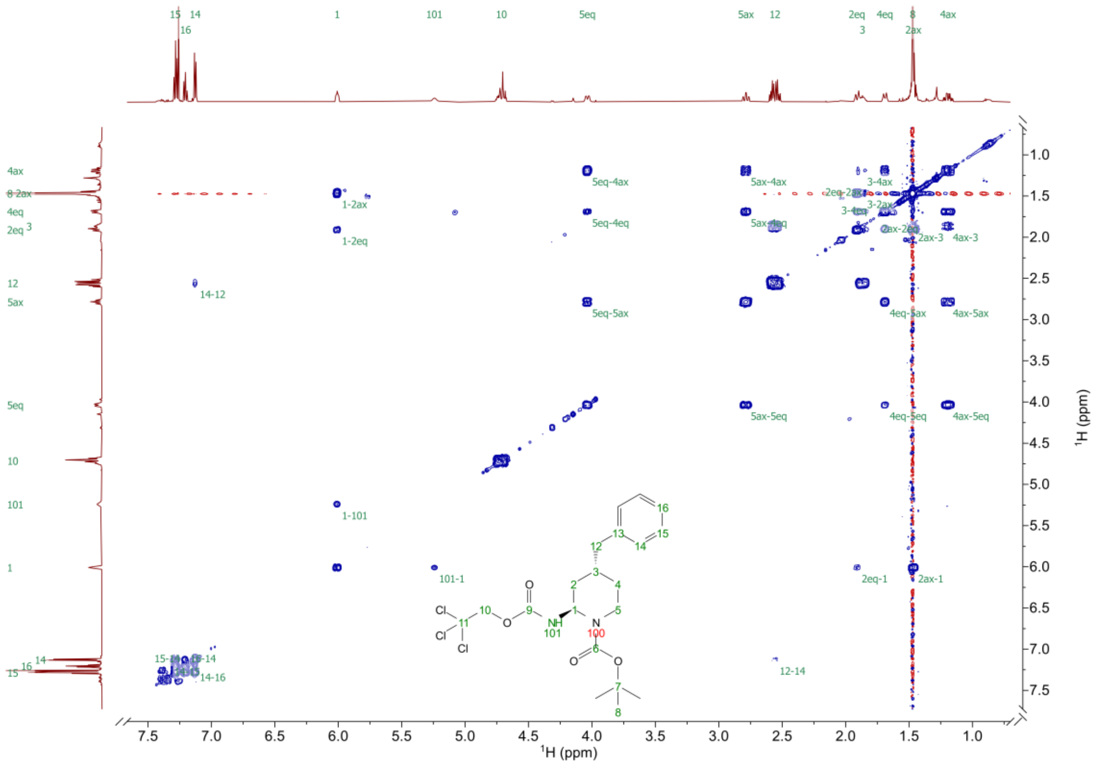

Compound **7o**: ^{1}H-^{1}H NOESY ($CDCl_3$, 333 K)

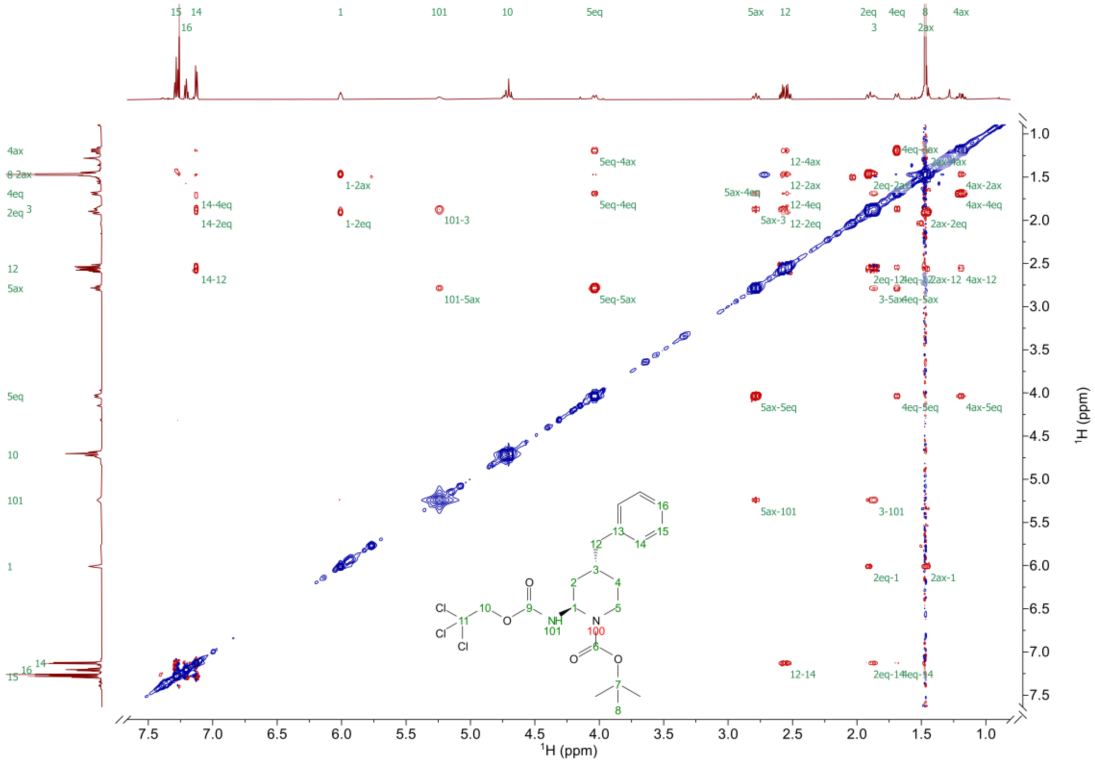

Compound **7o**: ^{1}H-^{15}N HMBC ($CDCl_3$, 333 K)

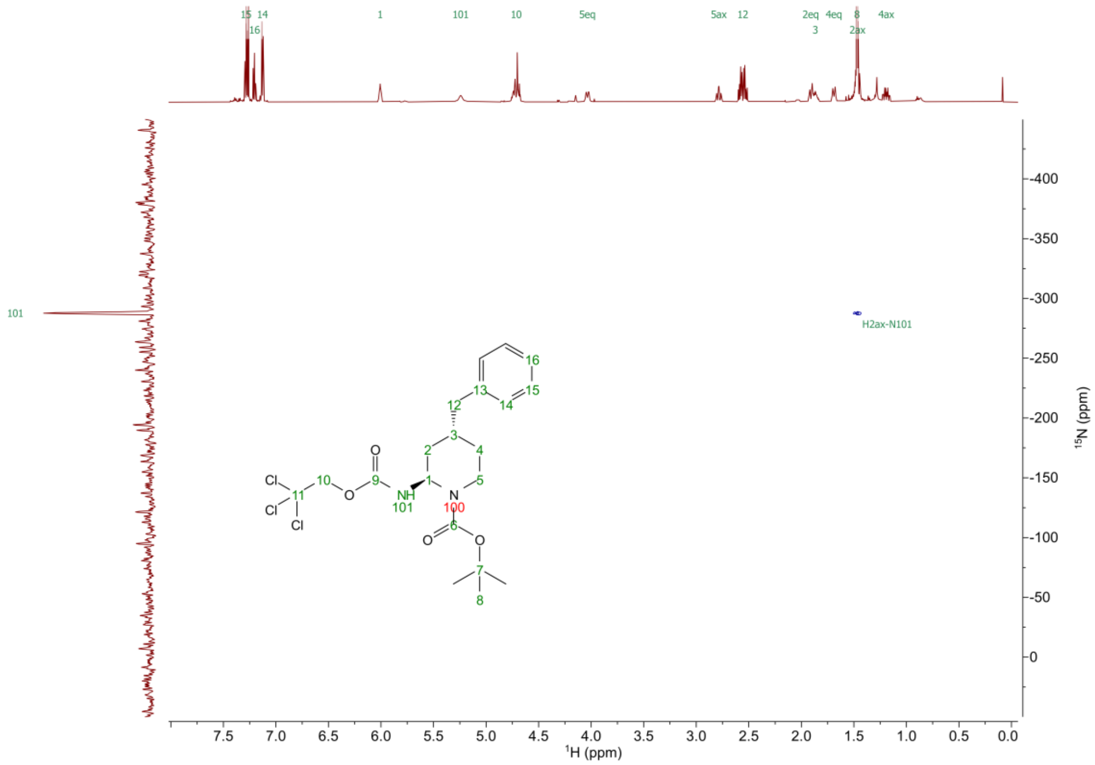

Compound **7p**: ^{1}H NMR (600 MHz, $CDCl_3$, 333 K)

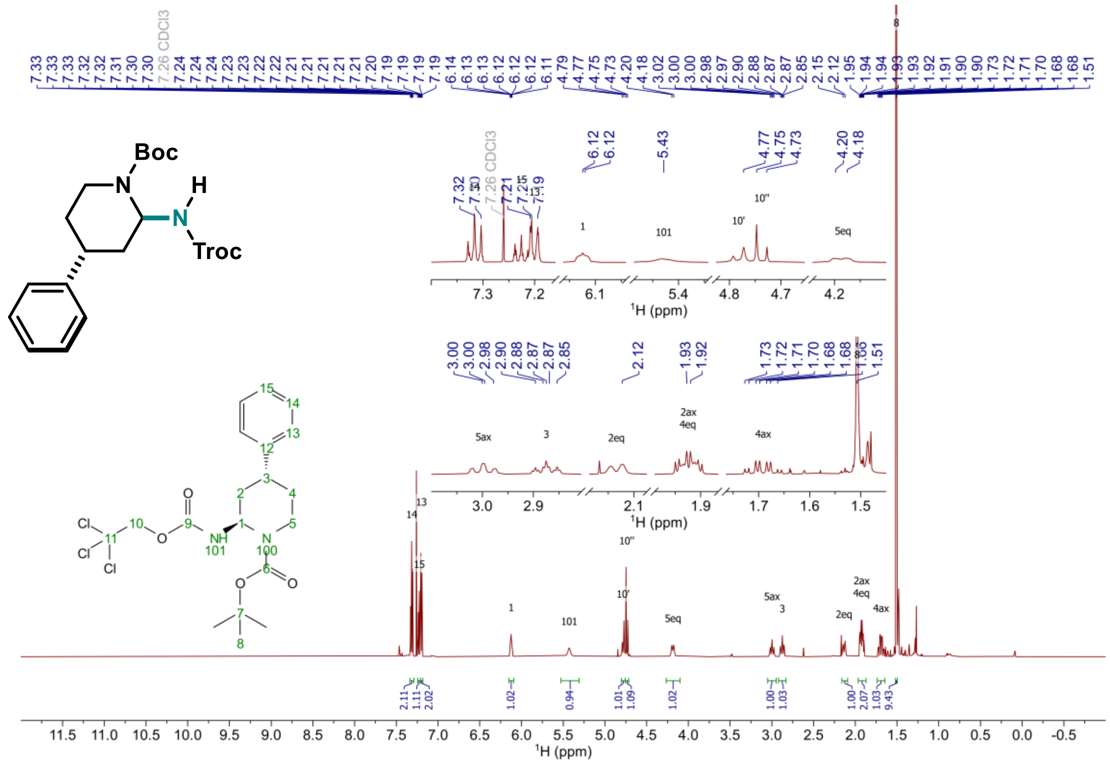

Compound **7p**: ^{13}C NMR (151 MHz, $CDCl_3$, 333 K)

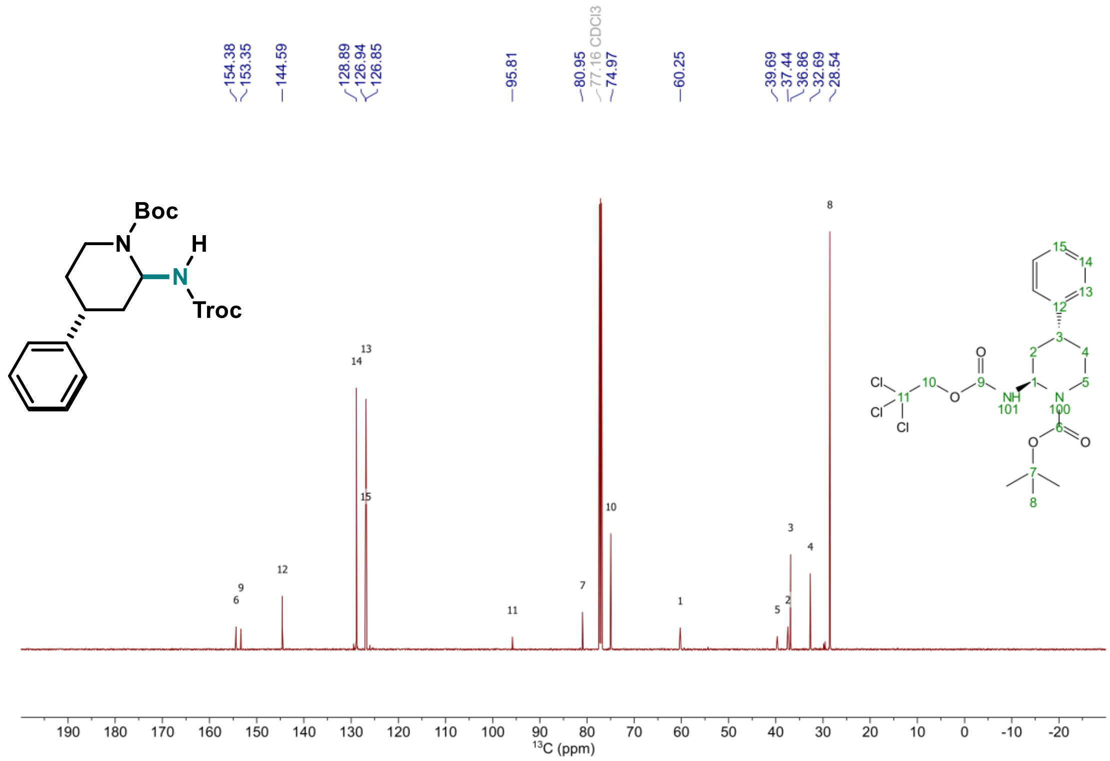

Compound **7p**: variable temperature ^{1}H NMR (600 MHz, $CDCl_3$)

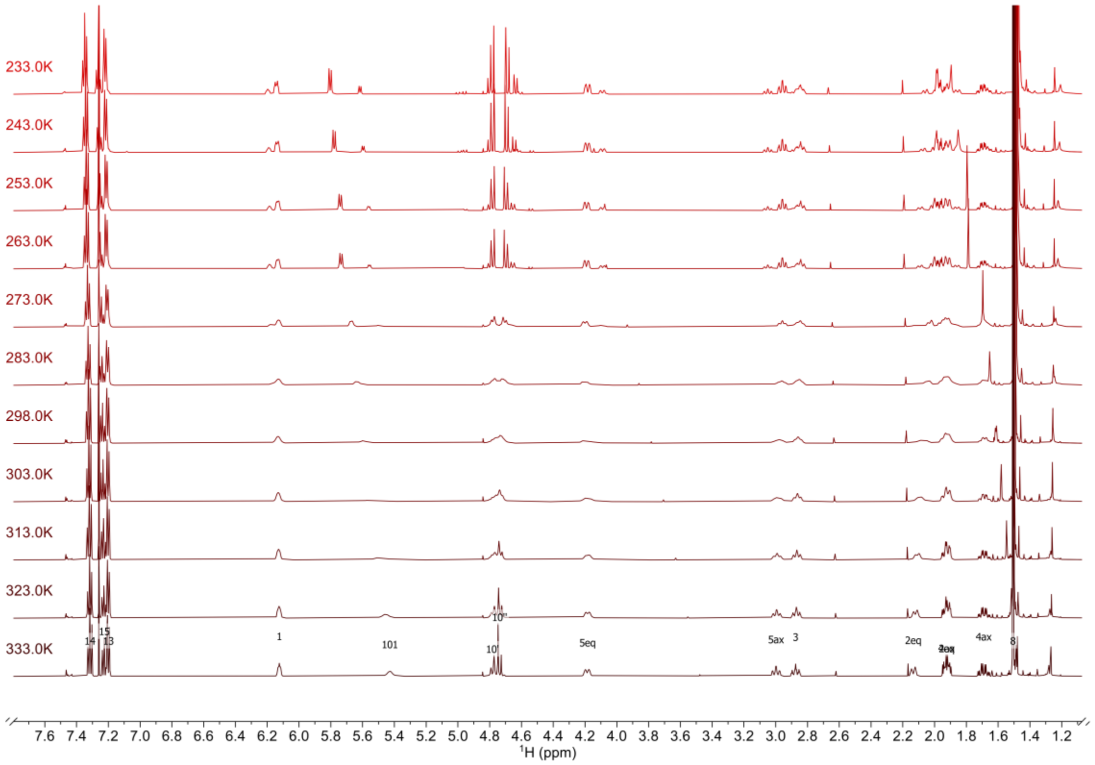

Compound **7p**: ^{1}H-^{13}C HSQC ($CDCl_3$, 333 K)

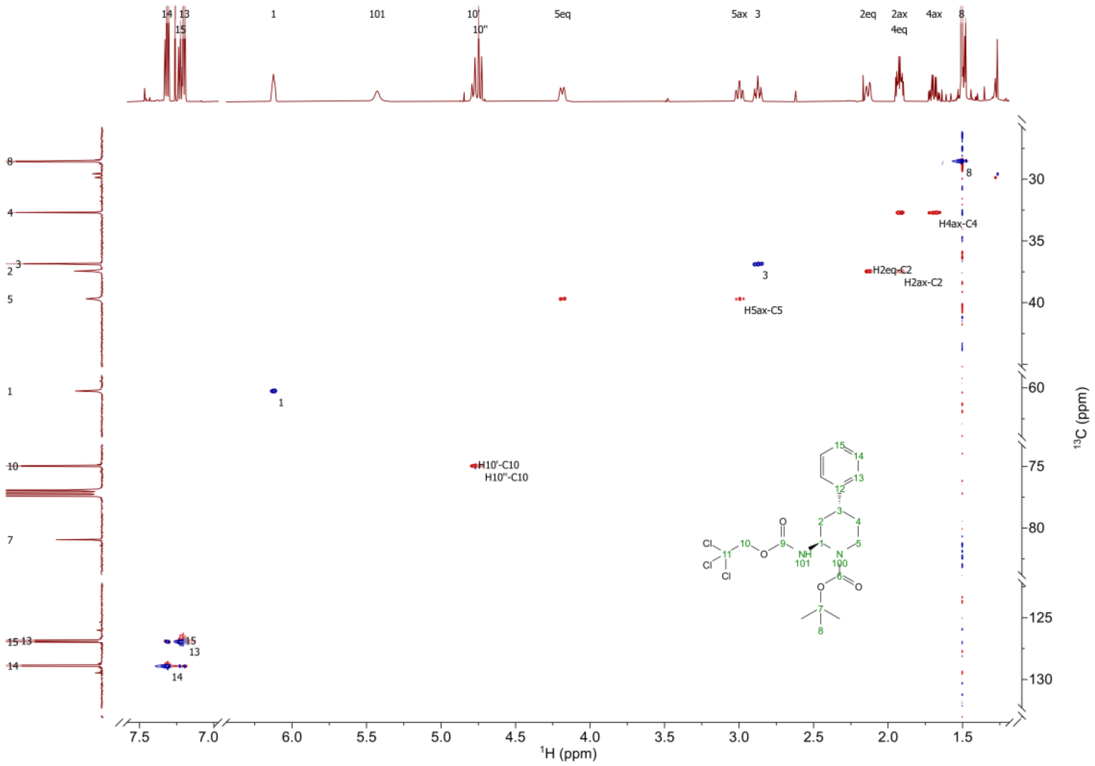

Compound **7p**: ^{1}H-^{13}C HMBC ($CDCl_3$, 333 K)

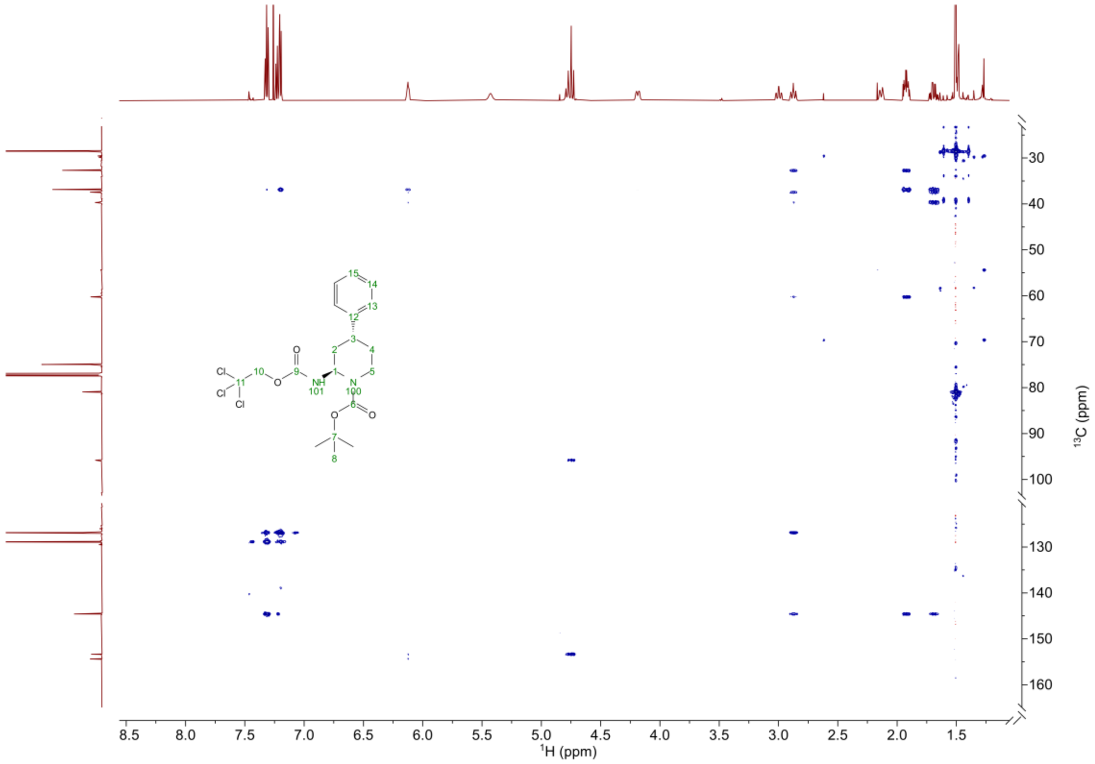

Compound **7p**: ^{1}H-^{1}H COSY ($CDCl_3$, 333 K)

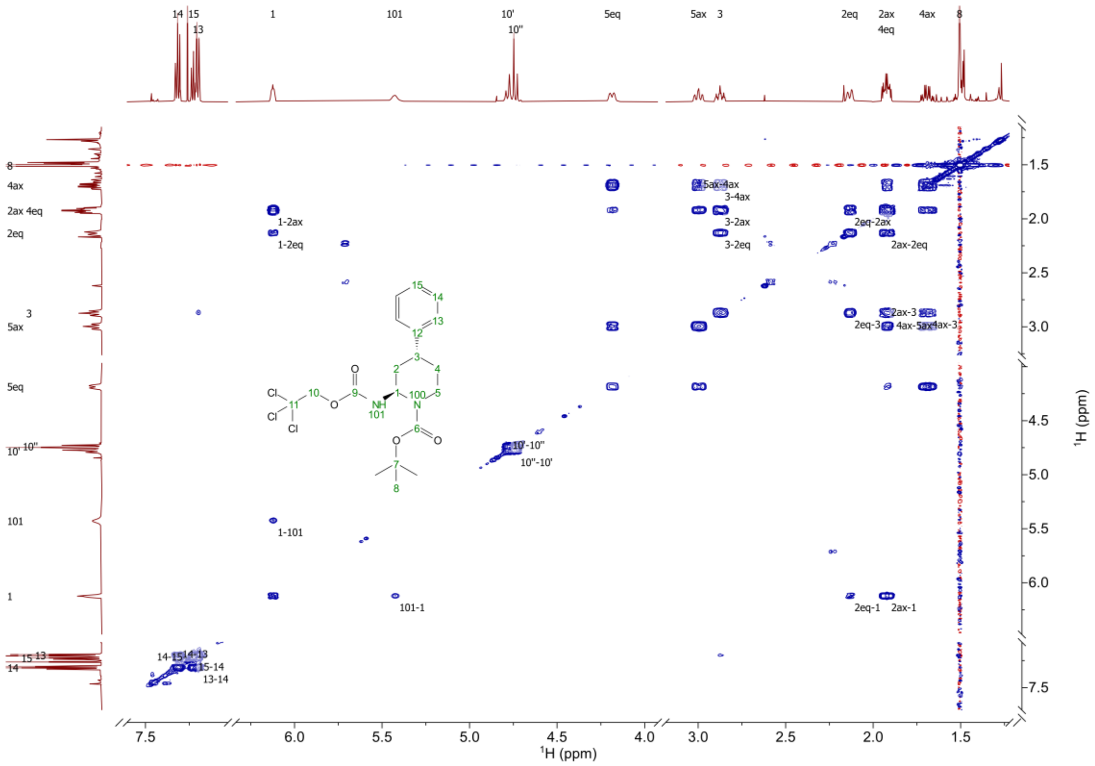

Compound **7p**: ^{1}H-^{1}H NOESY ($CDCl_3$, 333 K)

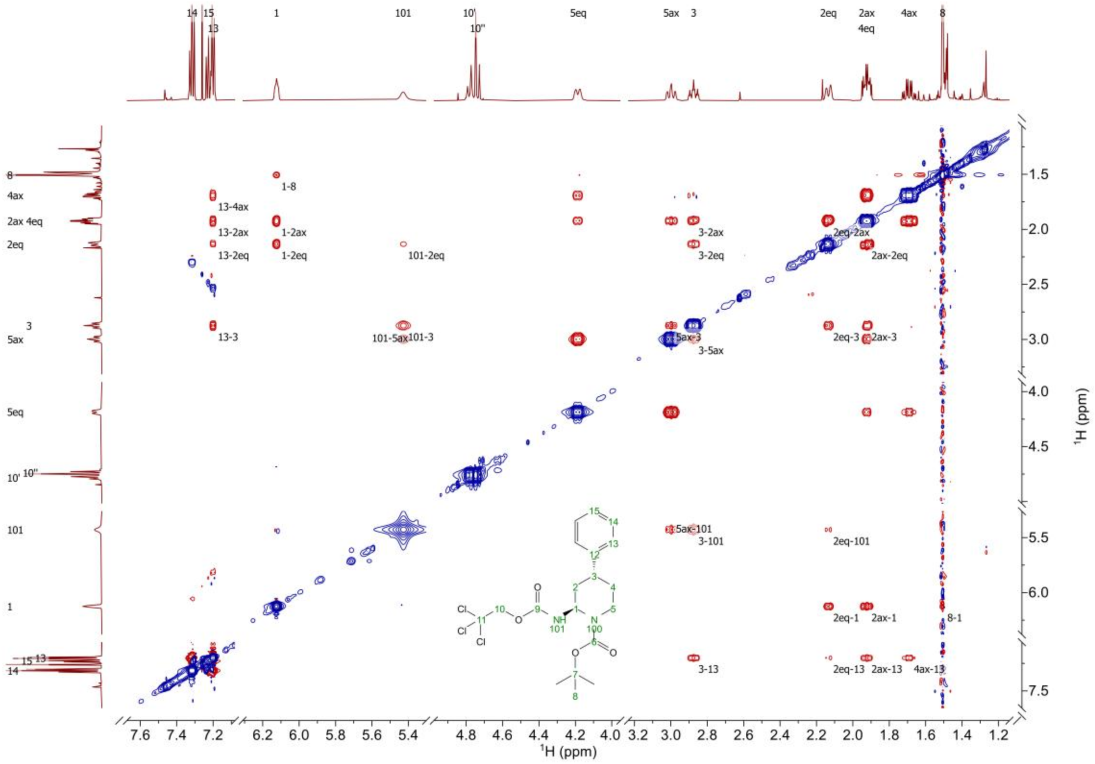

Compound **7p**: ^{1}H-^{15}N HMBC ($CDCl_3$, 333 K)

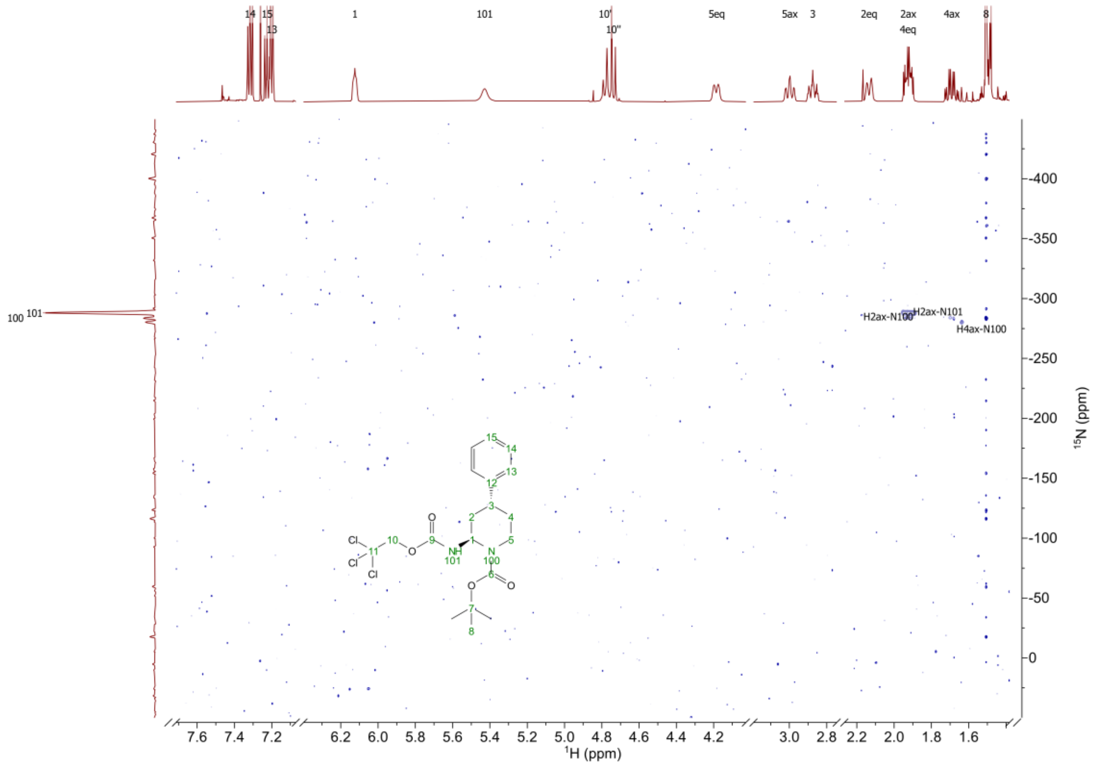

Compound **7q**: ^{1}H NMR (600 MHz, $CDCl_3$, 233 K)

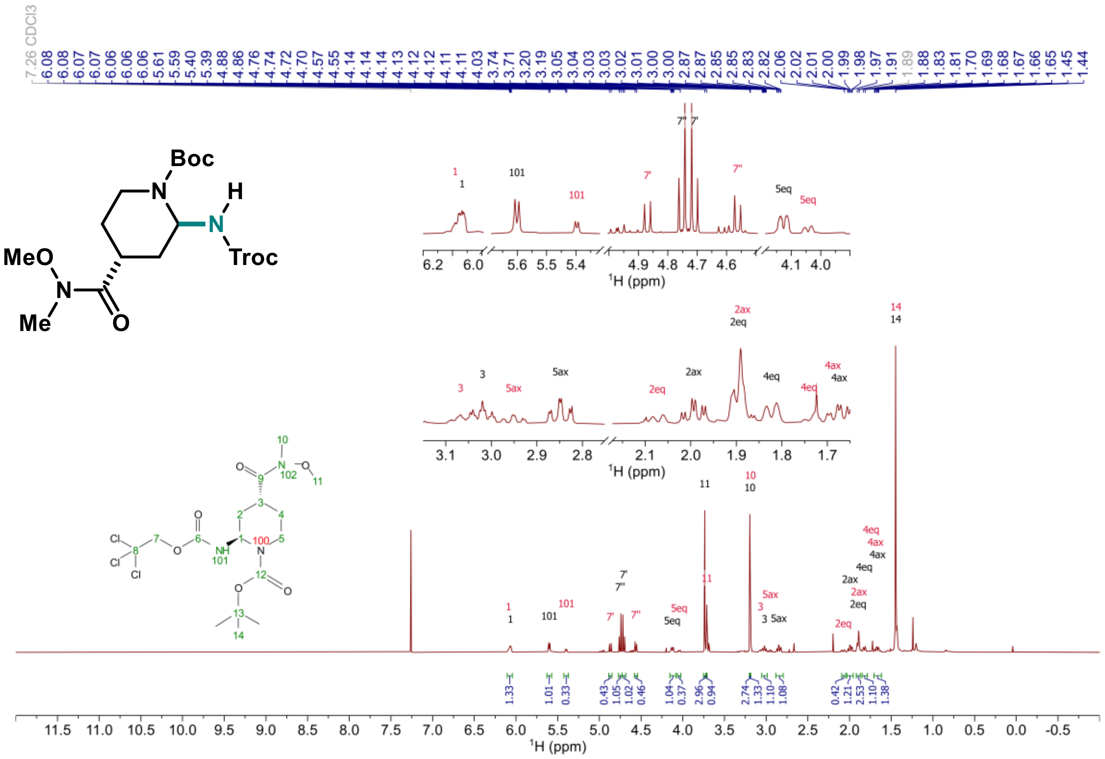

Compound **7q**: ^{13}C NMR (151 MHz, $CDCl_3$, 233 K)

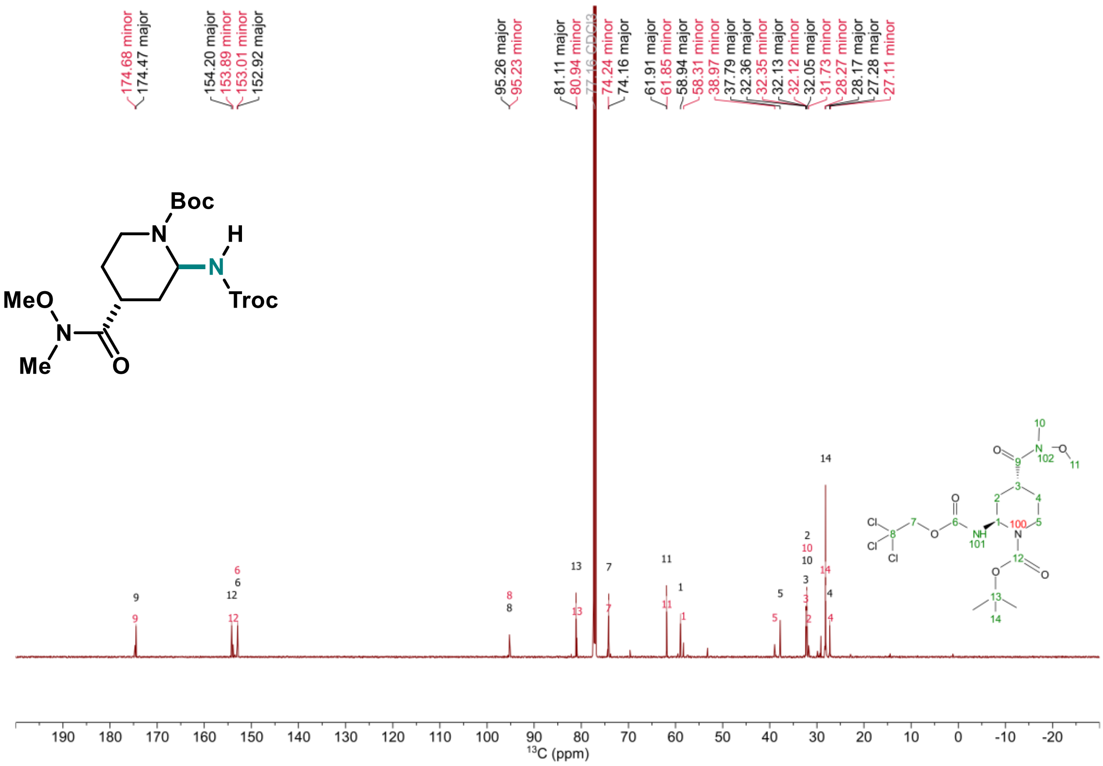

Compound **7q**: variable temperature ^{1}H NMR (600 MHz, $CDCl_3$)

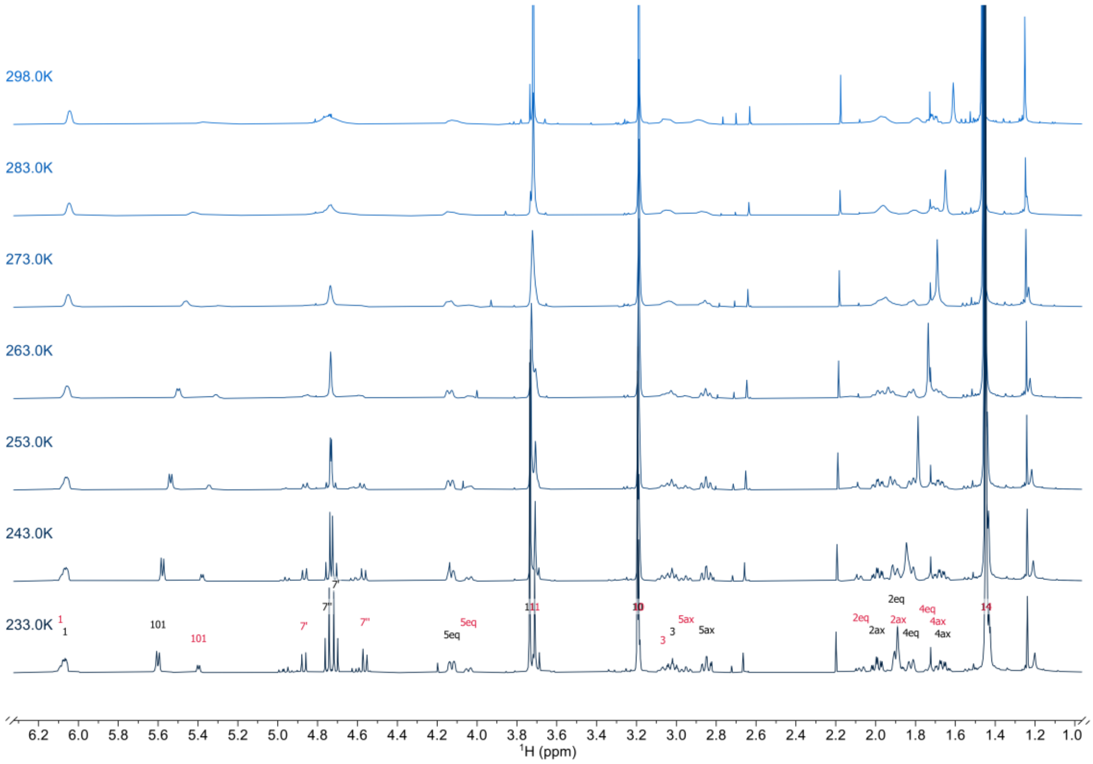

Compound **7q**: ^{1}H-^{13}C HSQC ($CDCl_3$, 233 K)

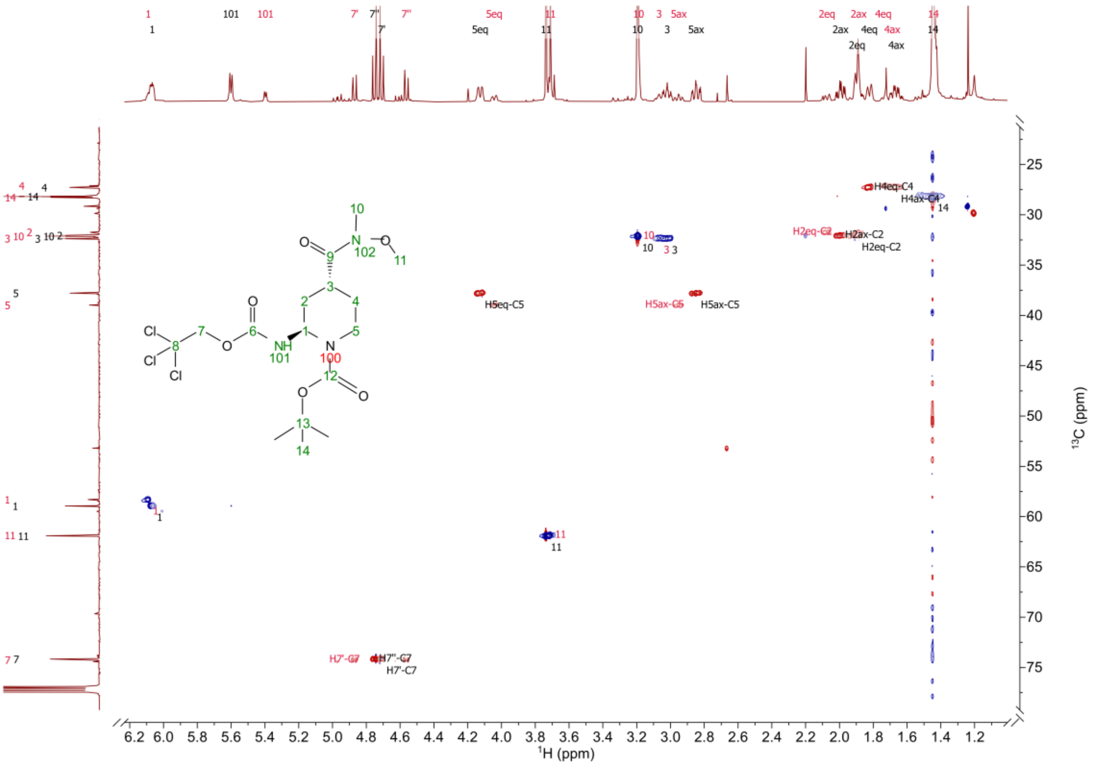

Compound **7q**: ^{1}H-^{13}C HMBC ($CDCl_3$, 233 K)

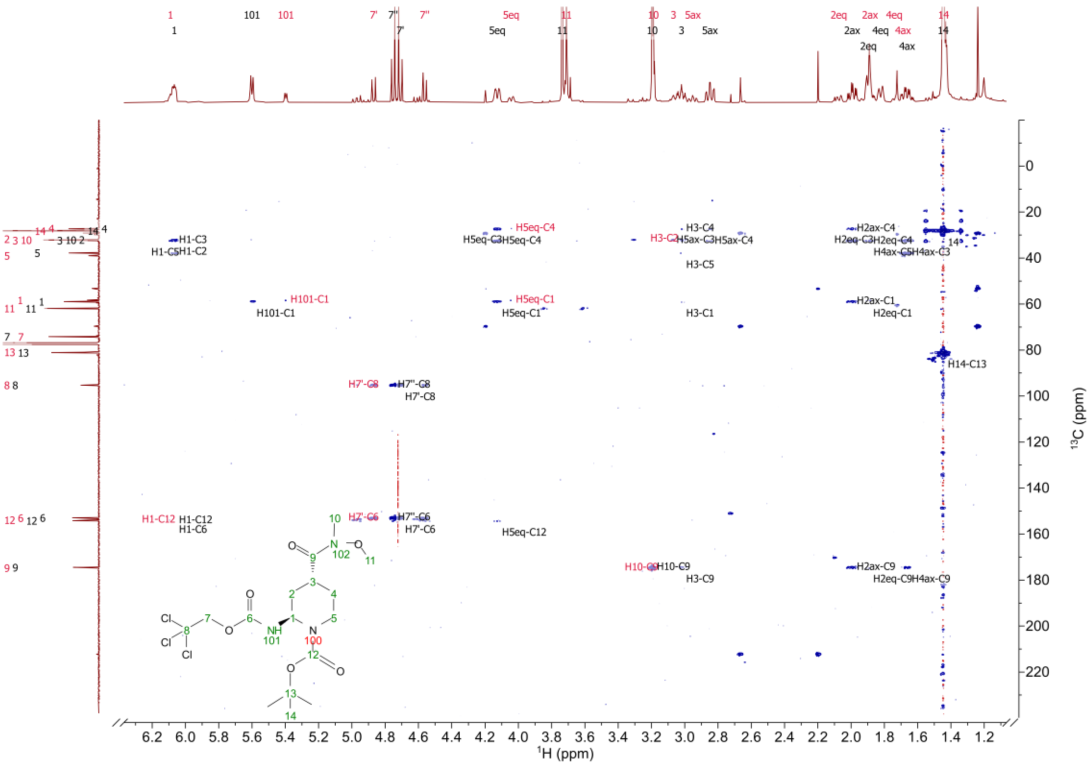

Compound **7q**: ^{1}H-^{1}H COSY ($CDCl_3$, 233 K)

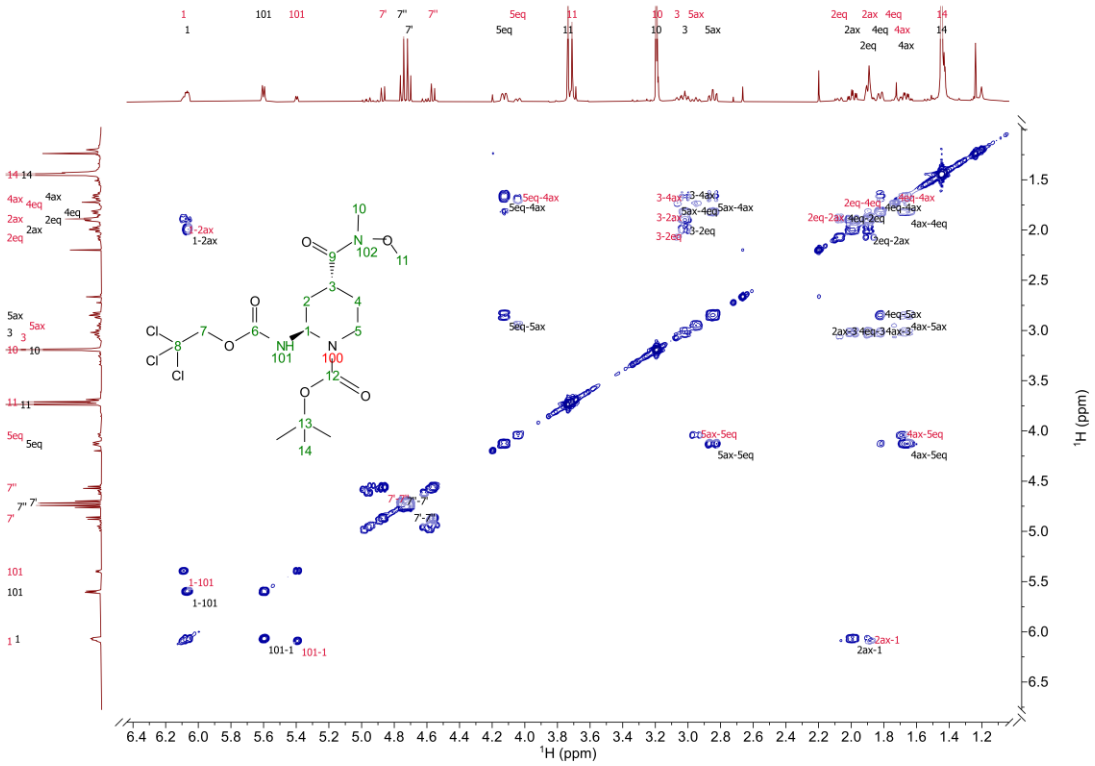

Compound **7q**: ^{1}H-^{1}H NOESY ($CDCl_3$, 233 K)

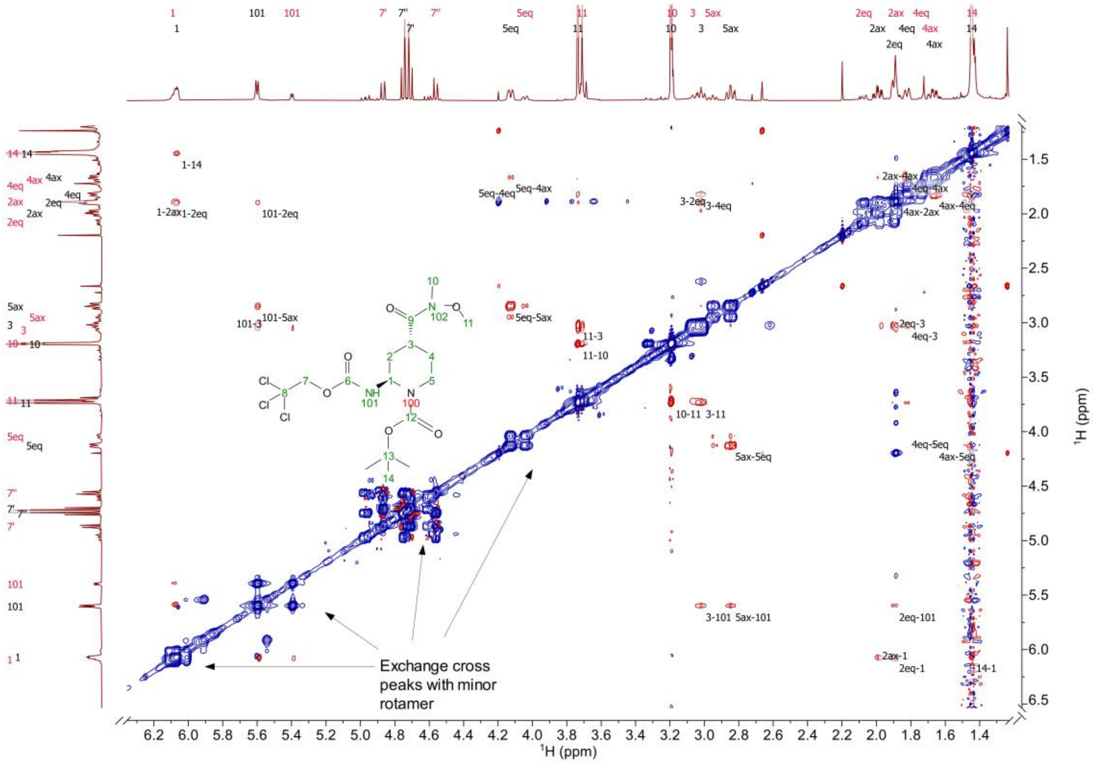

Compound **7q**: ^{1}H-^{15}N HMBC ($CDCl_3$, 233 K)

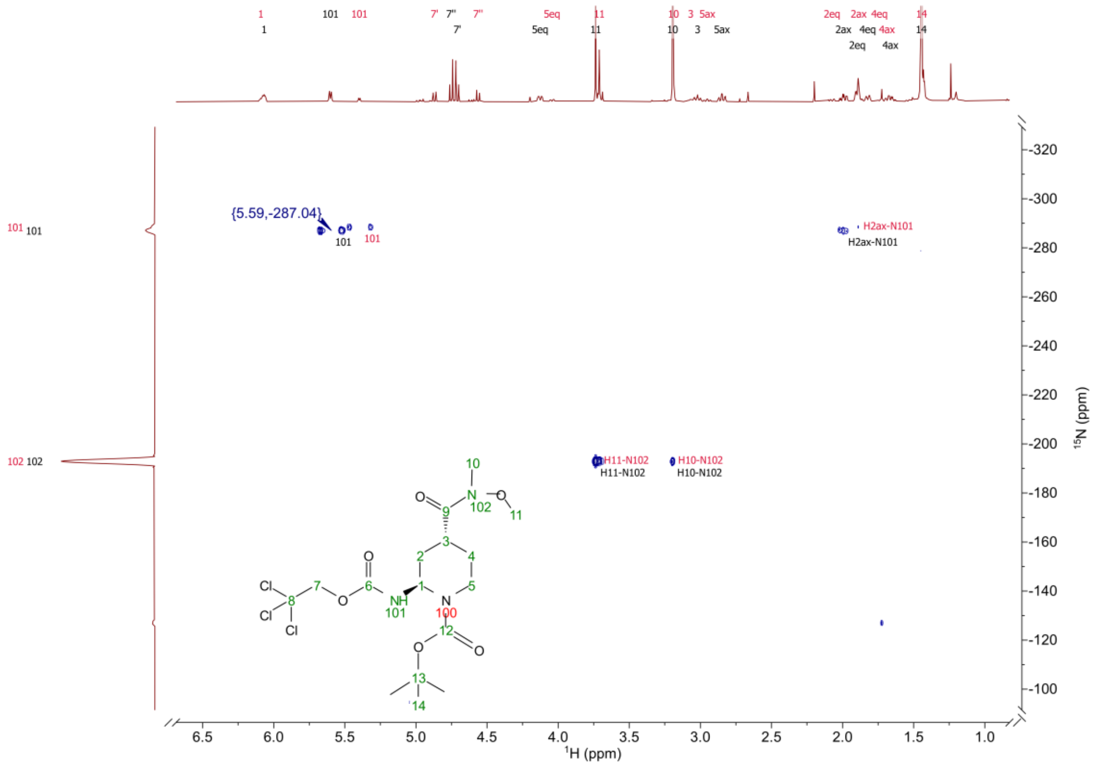

Compound **7q**: 1D selective TOCSY from excitation of H101

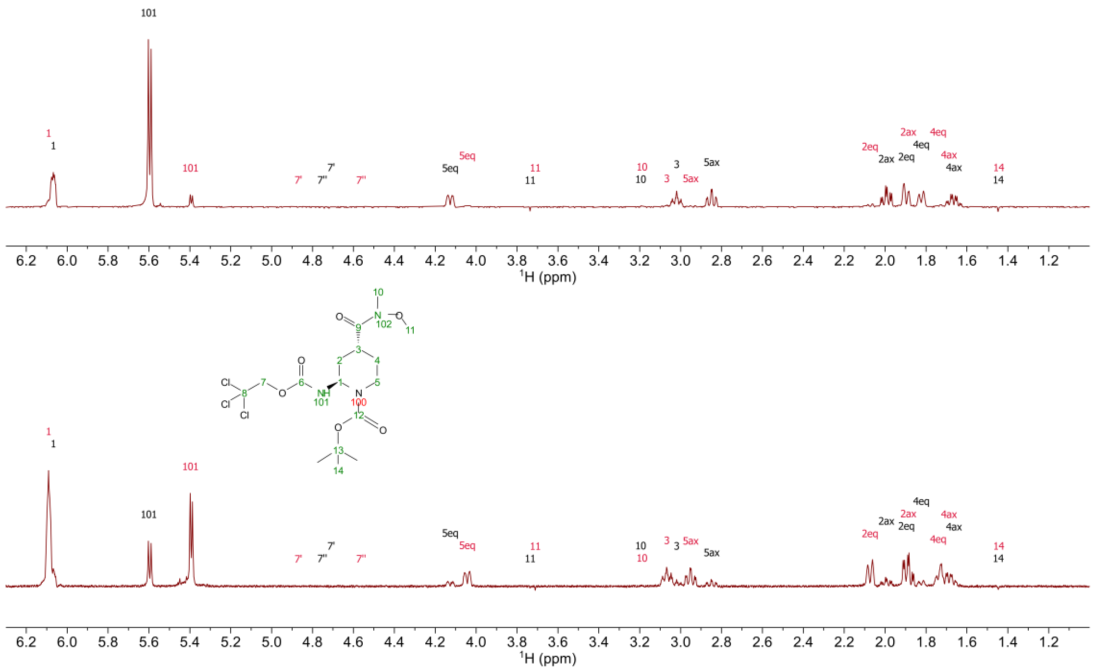

Compound **7r**: ^{1}H NMR (600 MHz, $CDCl_3$, 333 K)

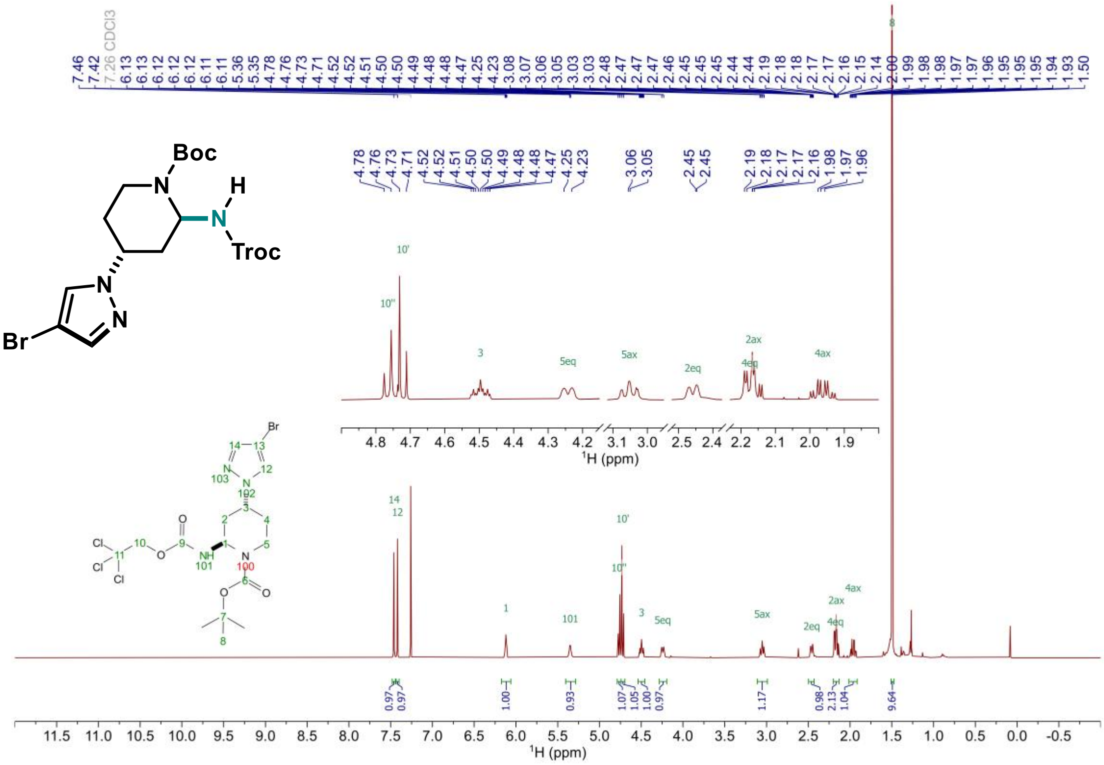

Compound **7r**: ^{13}C NMR (151 MHz, $CDCl_3$, 333 K)

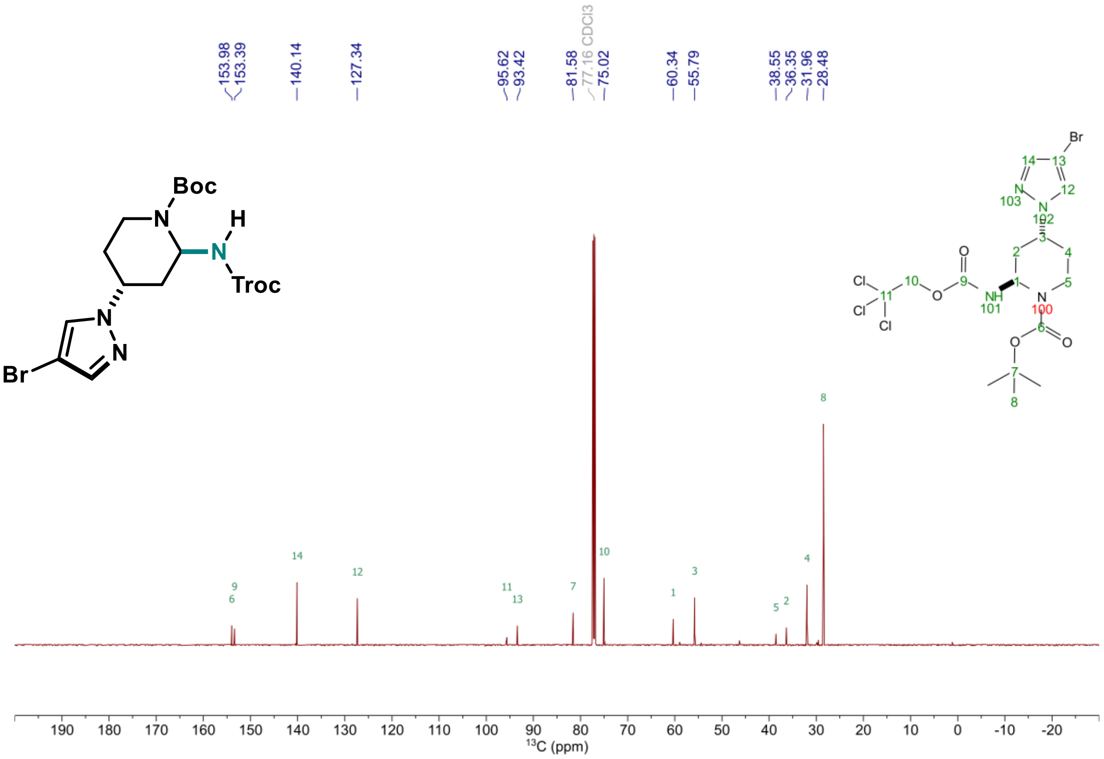

Compound **7r**: variable temperature ^{1}H NMR (600 MHz, $CDCl_3$)

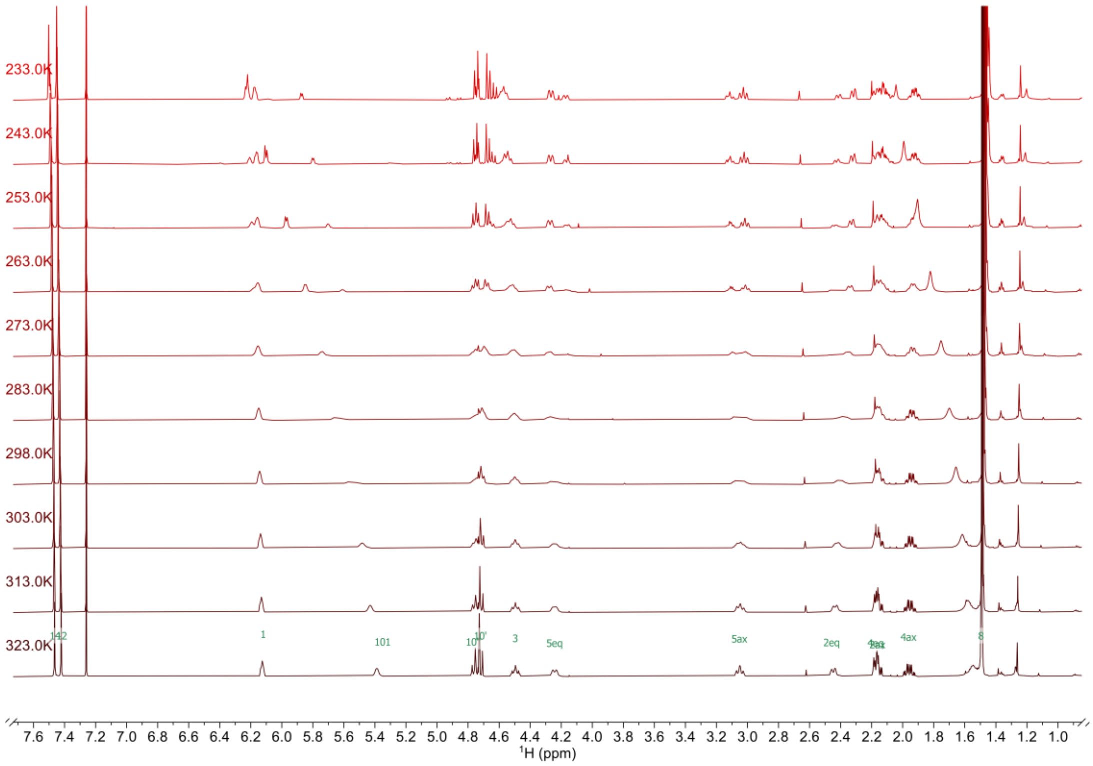

Compound **7r**: ^{1}H-^{13}C HSQC ($CDCl_3$, 333 K)

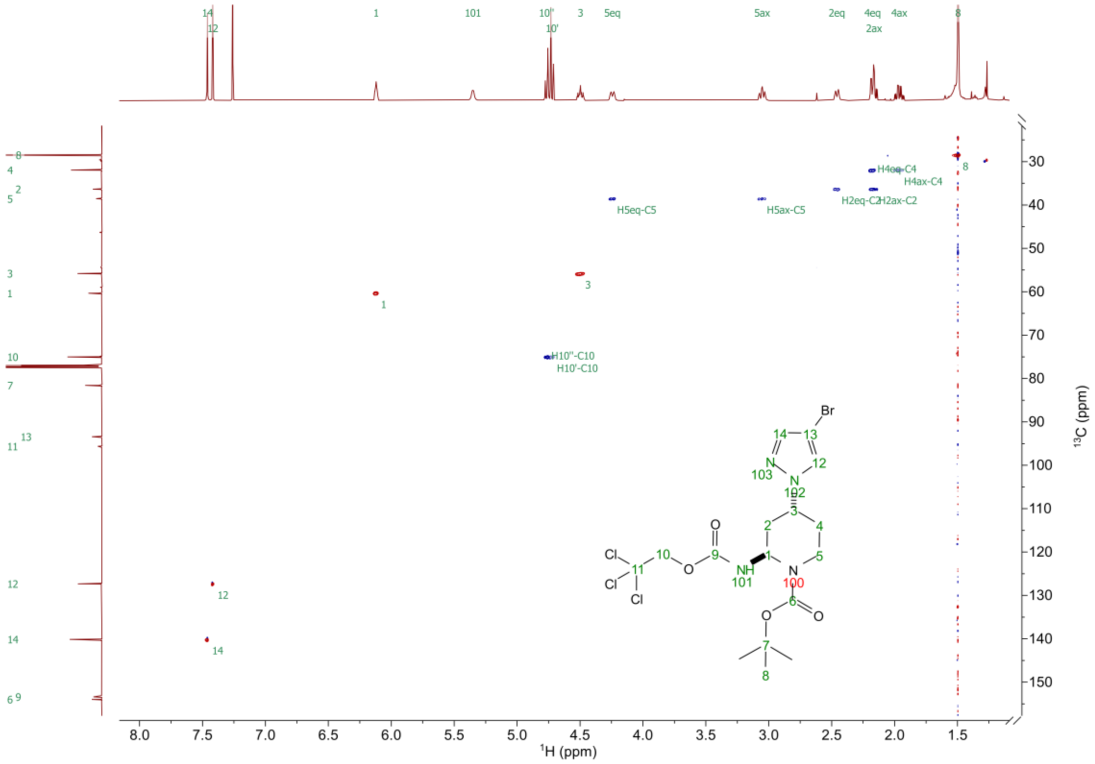

Compound **7r**: ^{1}H-^{13}C HMBC (CDCl$_3$, 333 K)

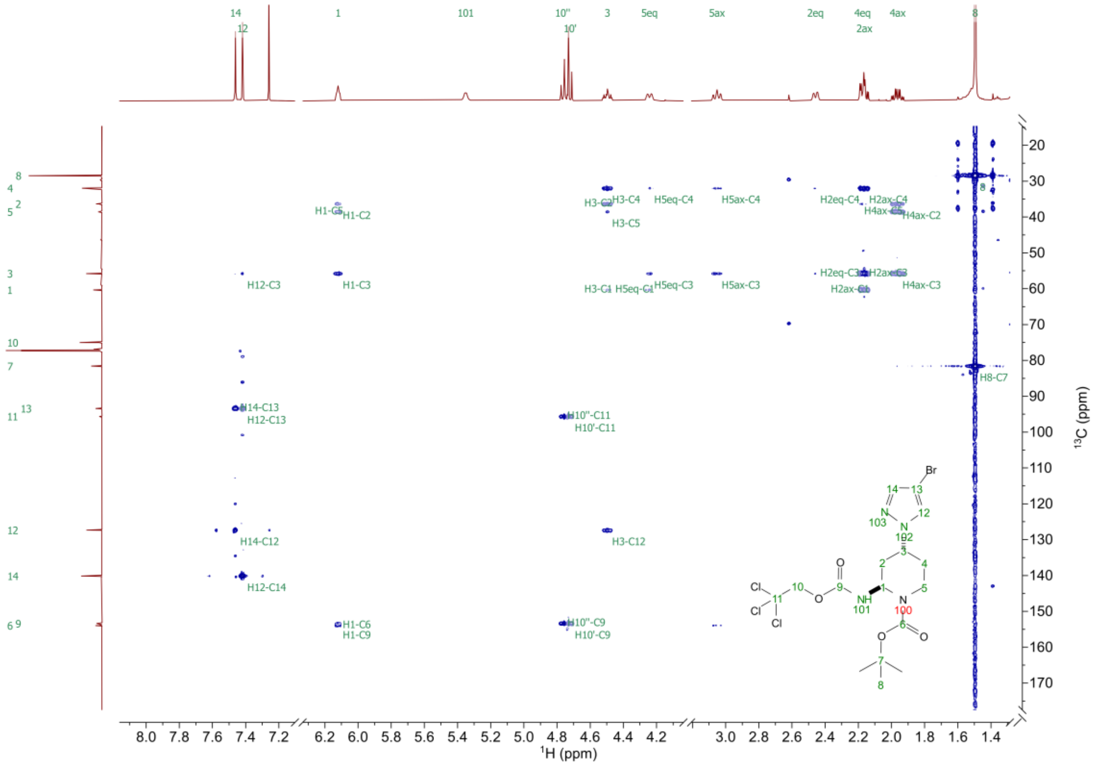

Compound **7r**: ^{1}H-^{1}H COSY (CDCl$_3$, 333 K)

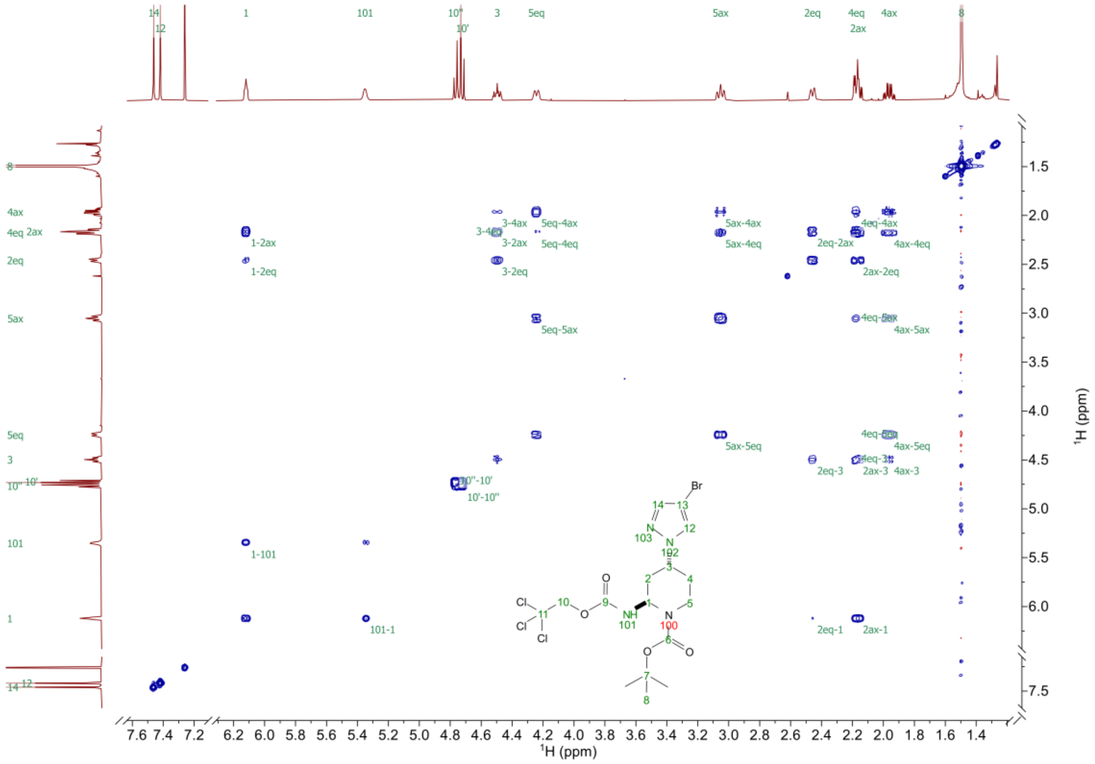

Compound **7r**: ^{1}H-^{1}H NOESY (CDCl$_3$, 333 K)

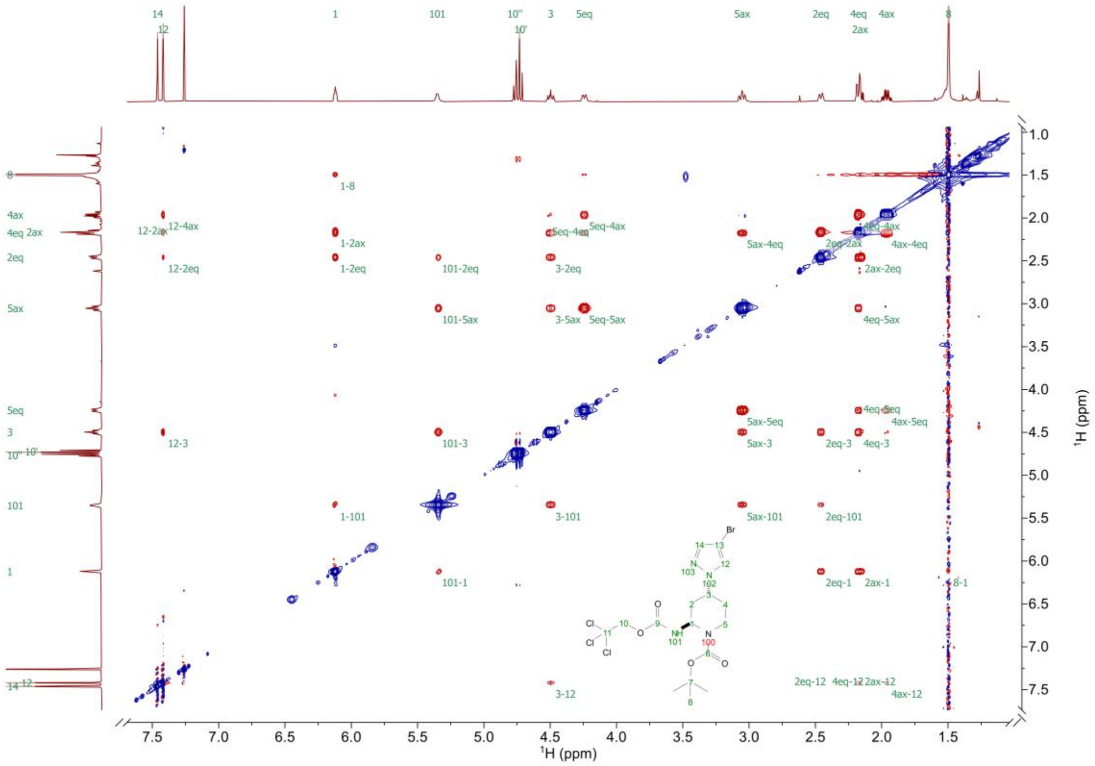

Compound **7r**: ^{1}H-^{15}N HMBC (CDCl$_3$, 333 K)

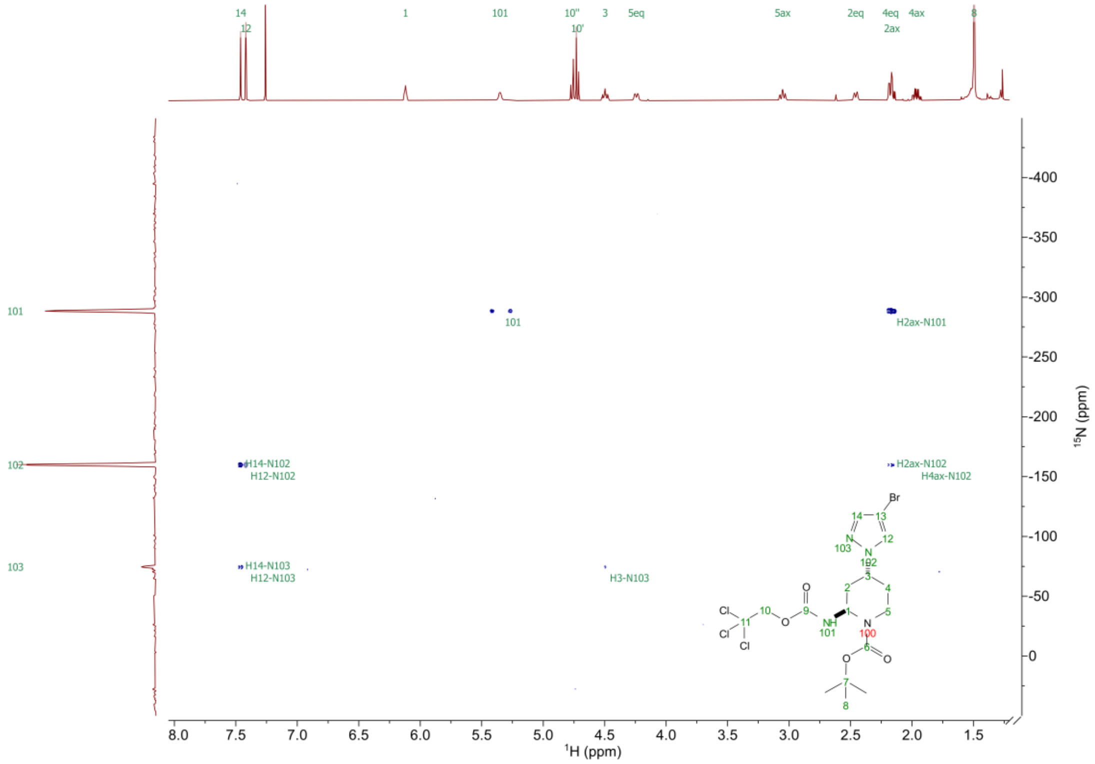

Compound **7s**: ^{1}H NMR (600 MHz, $CDCl_3$, 323 K)

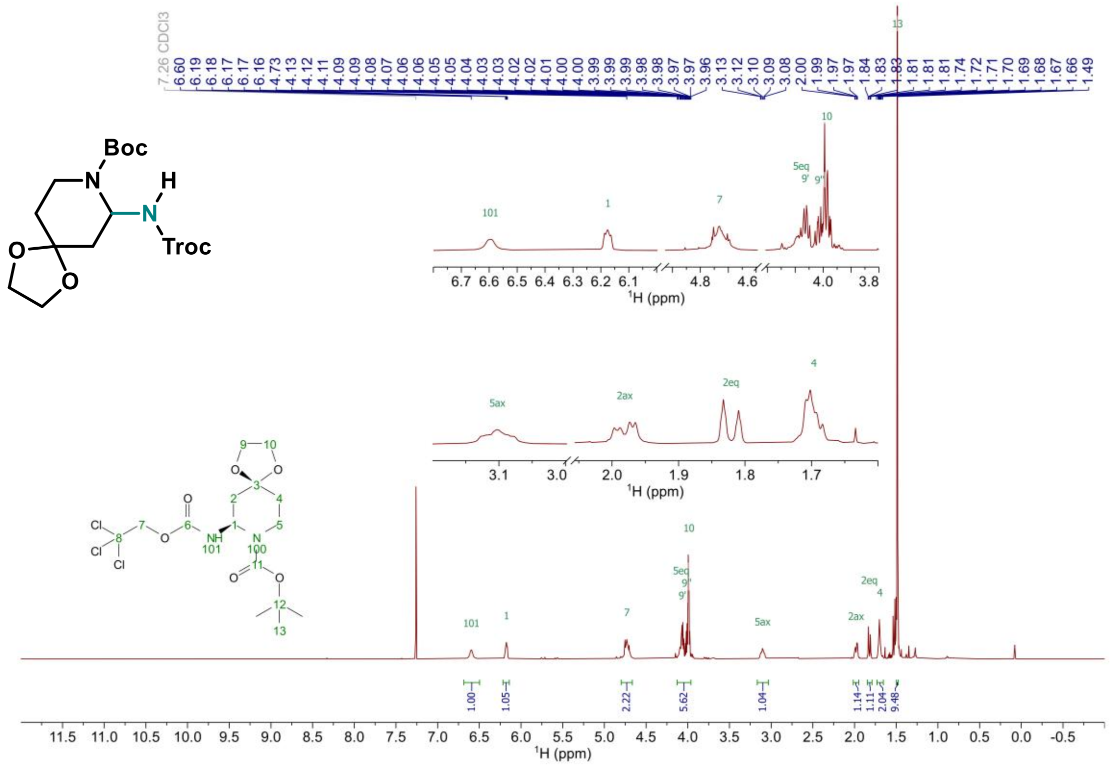

Compound **7s**: ^{13}C NMR (151 MHz, $CDCl_3$, 323 K)

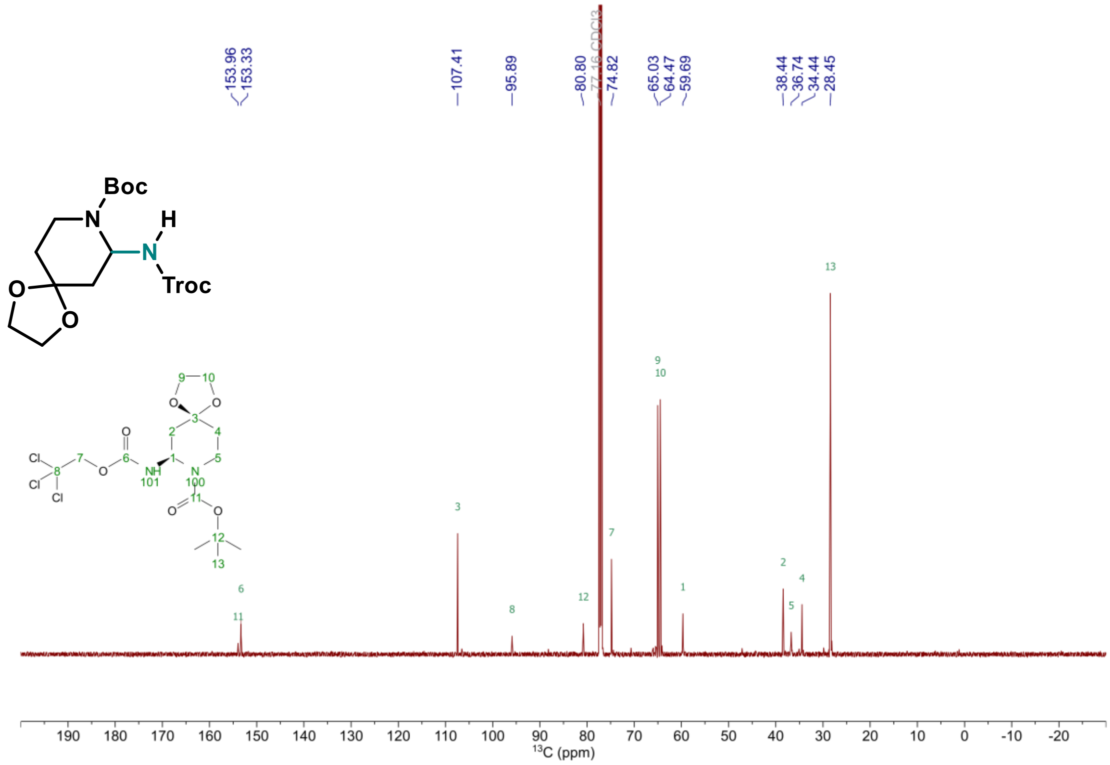

Compound **7s**: variable temperature ^{1}H NMR (600 MHz, $CDCl_3$)

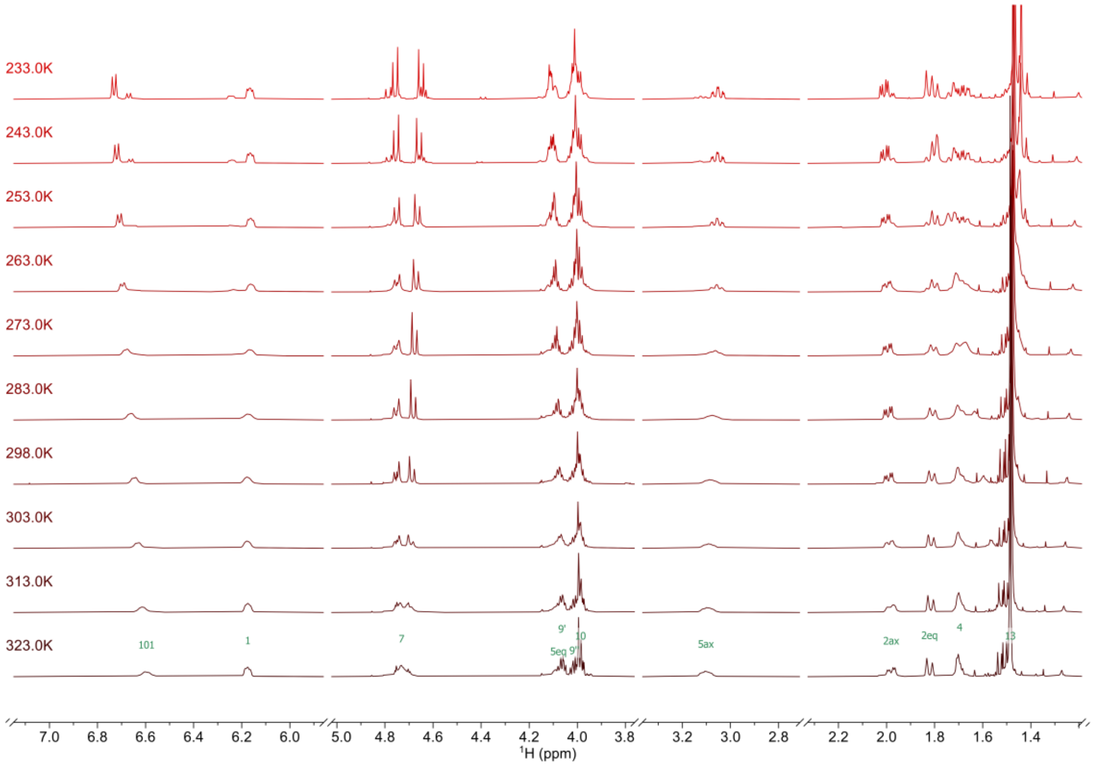

Compound **7s**: ^{1}H-^{13}C HSQC ($CDCl_3$, 323 K)

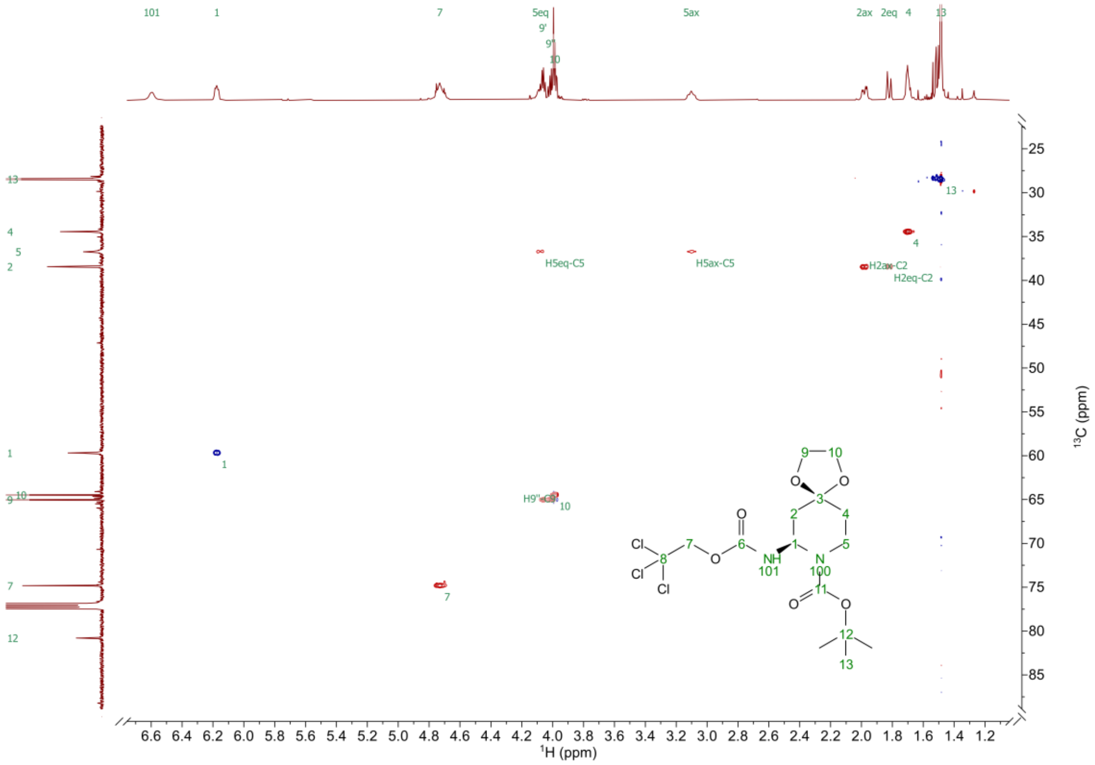

Compound **7s**: ^{1}H-^{13}C HMBC ($CDCl_3$, 323 K)

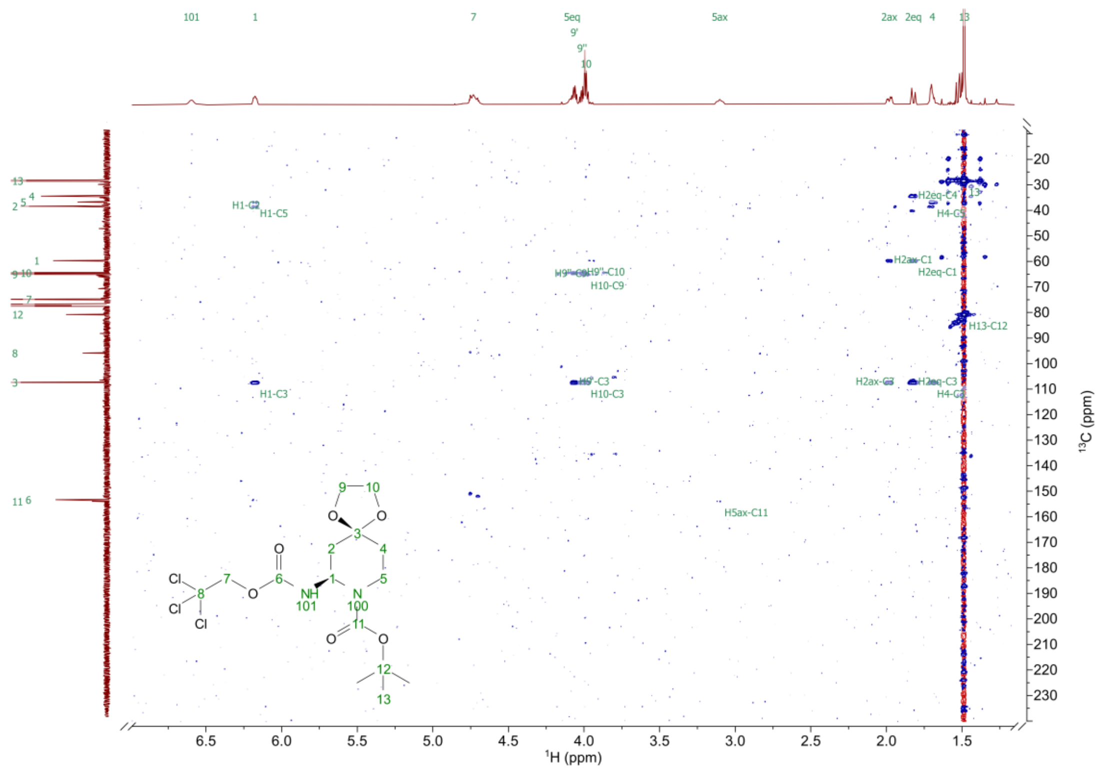

Compound **7s**: ^{1}H-^{1}H COSY ($CDCl_3$, 323 K)

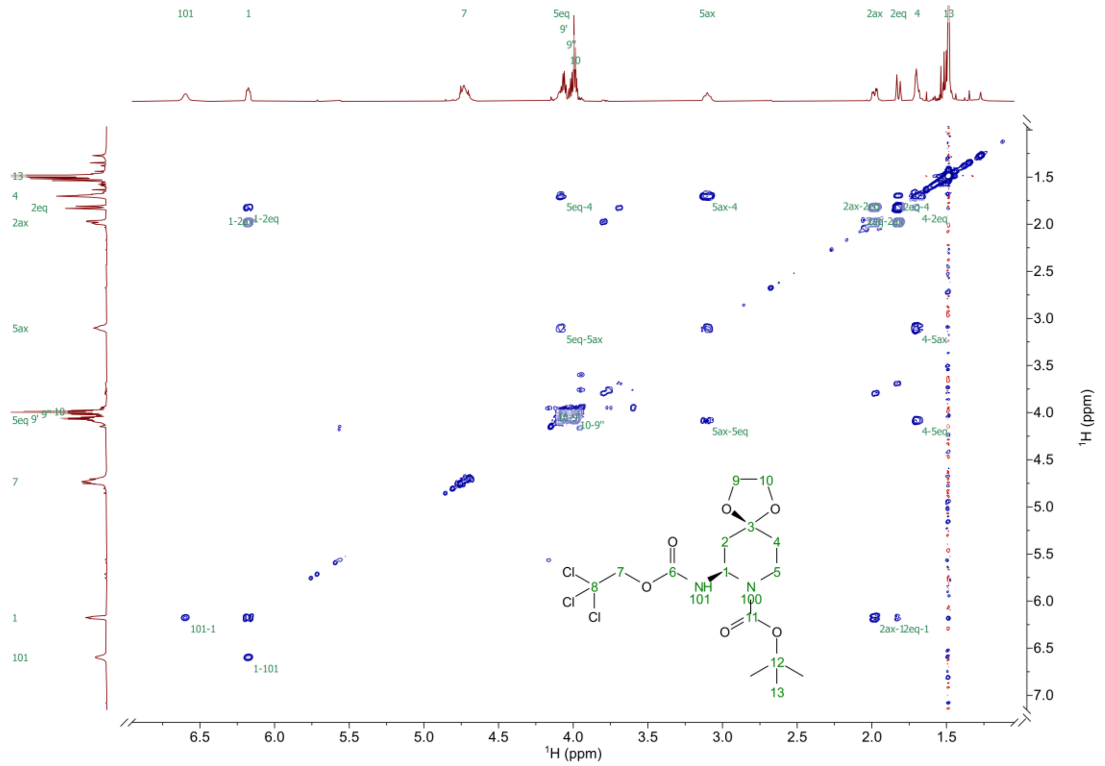

Compound **7s**: ^{1}H-^{1}H NOESY ($CDCl_3$, 323 K)

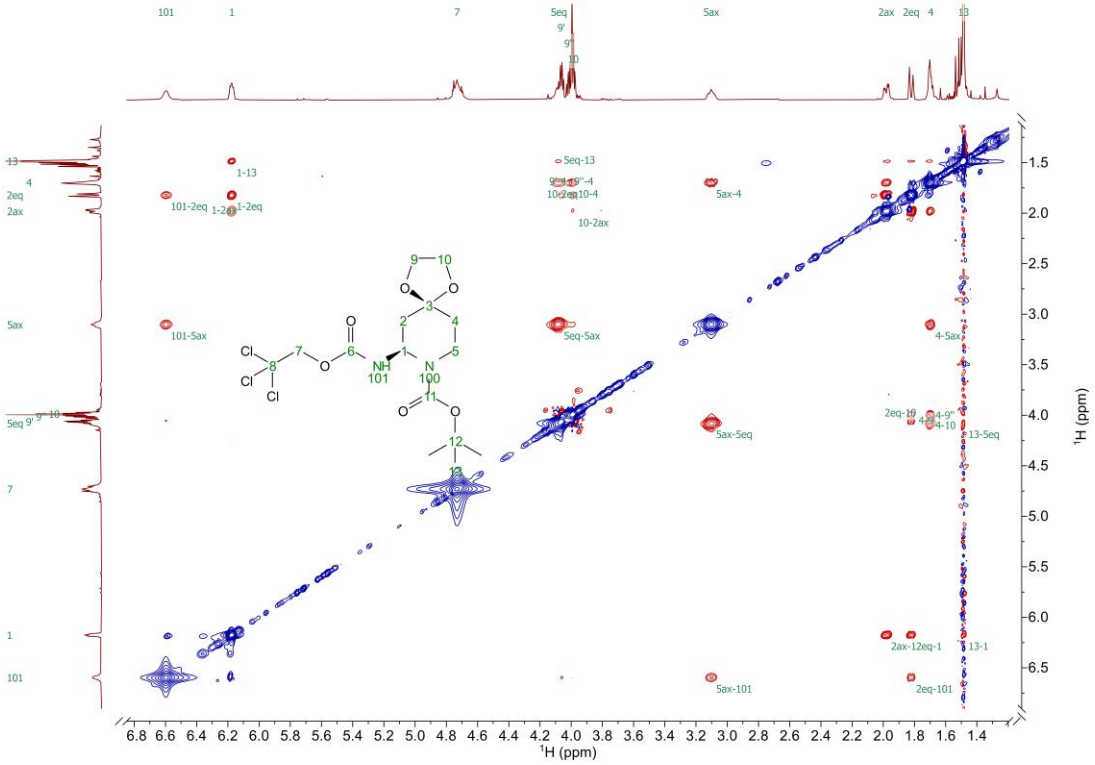

Compound **7s**: ^{1}H-^{15}N HMBC ($CDCl_3$, 323 K)

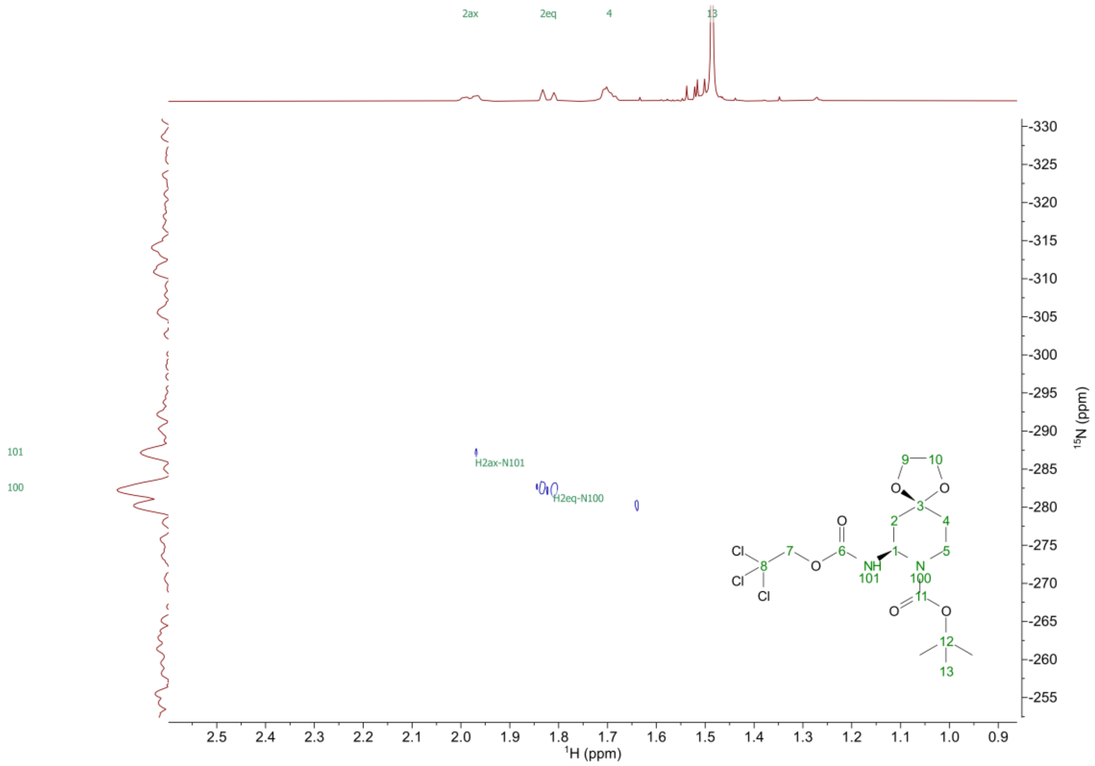

Compound **7t**: ^{1}H NMR (600 MHz, $CDCl_3$, 298 K)

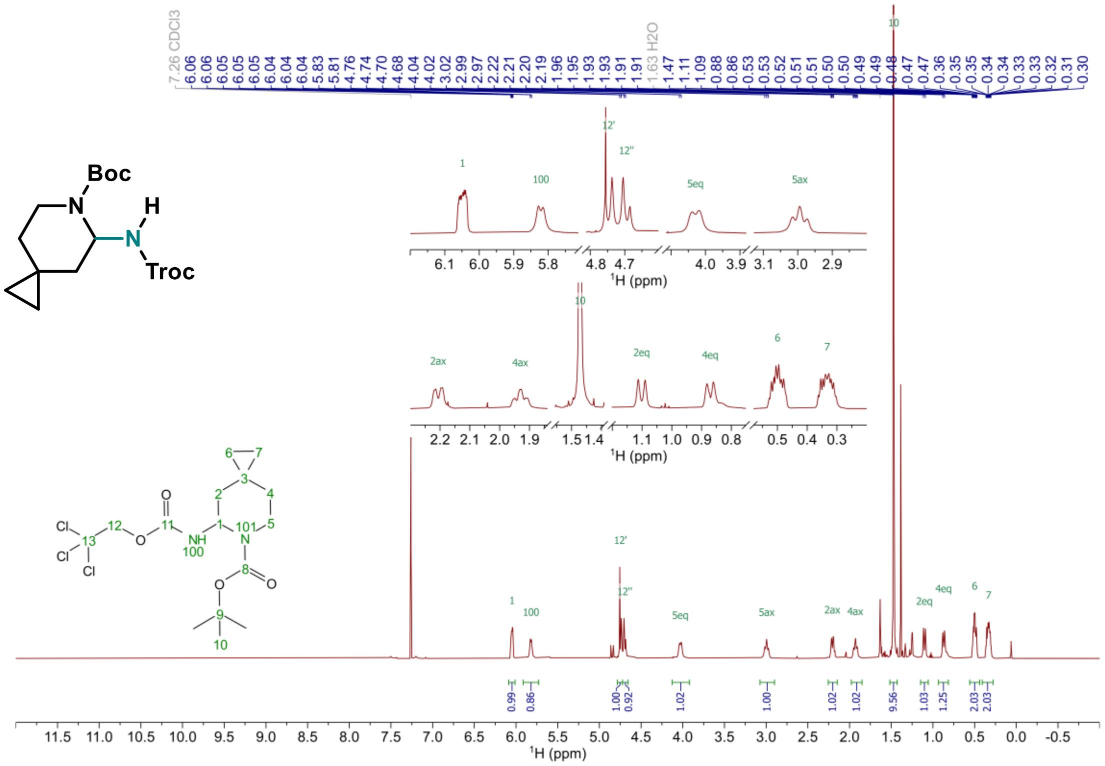

Compound **7t**: ^{13}C NMR (151 MHz, $CDCl_3$, 298 K)

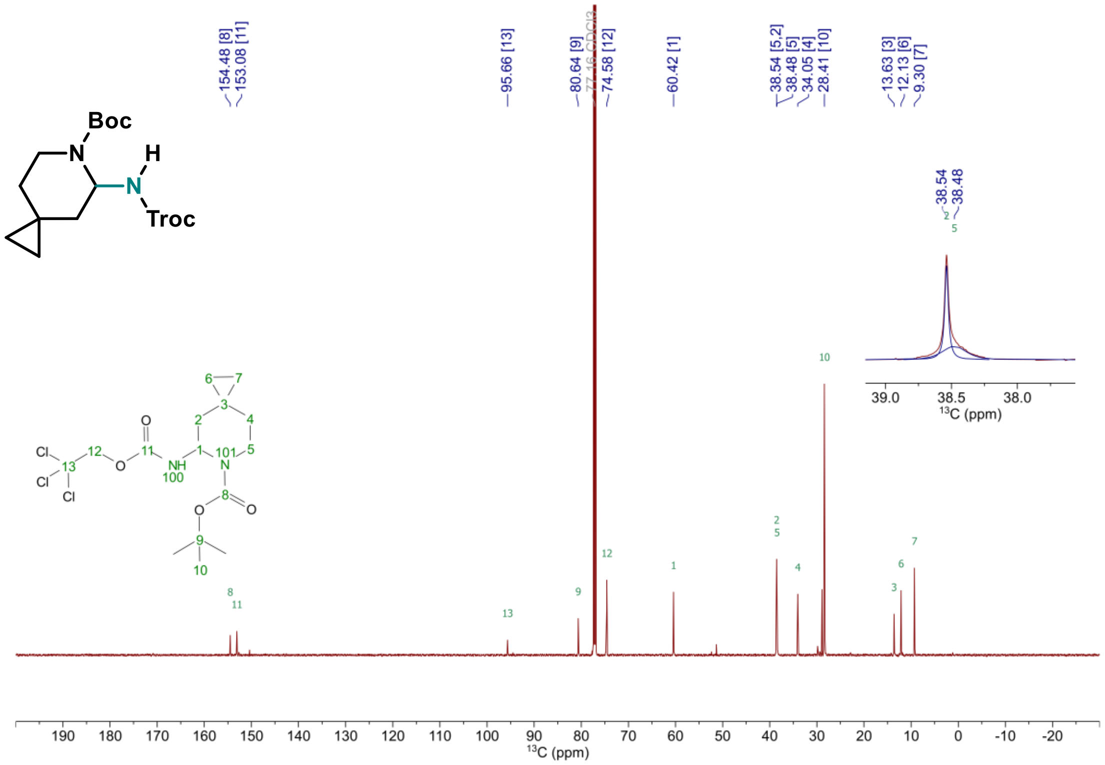

Compound **7t**: variable temperature ^{1}H NMR (600 MHz, $CDCl_3$)

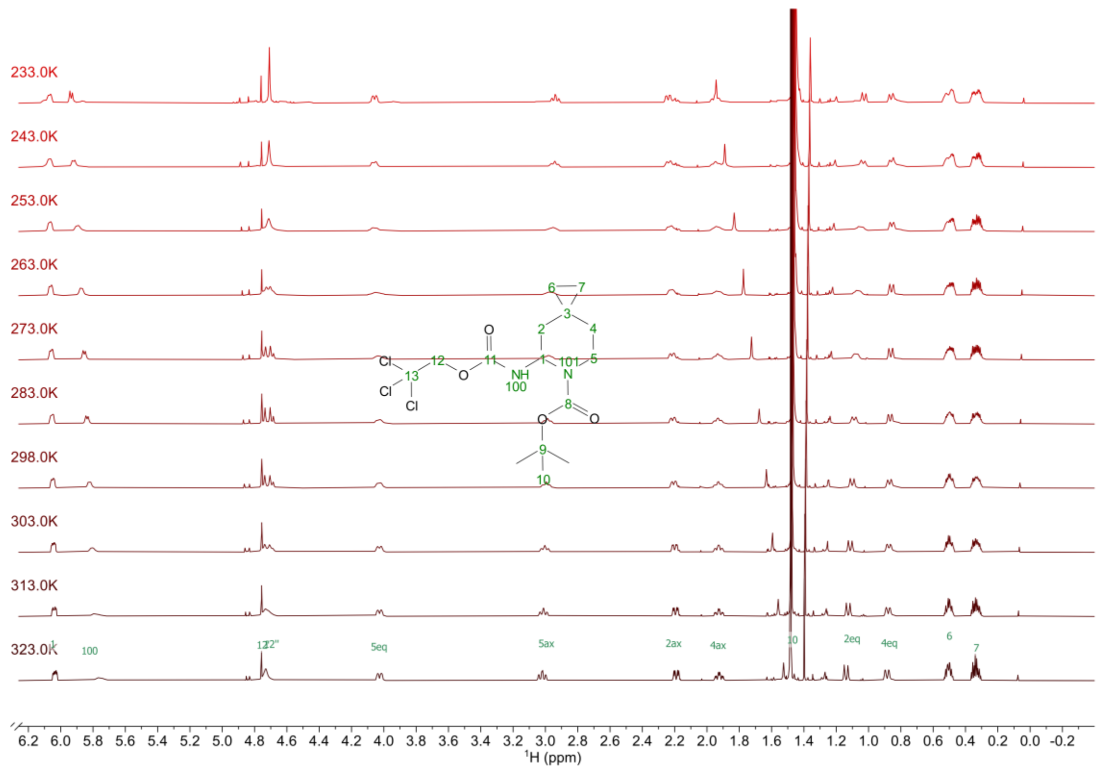

Compound **7t**: ^{1}H-^{13}C HSQC ($CDCl_3$, 298 K)

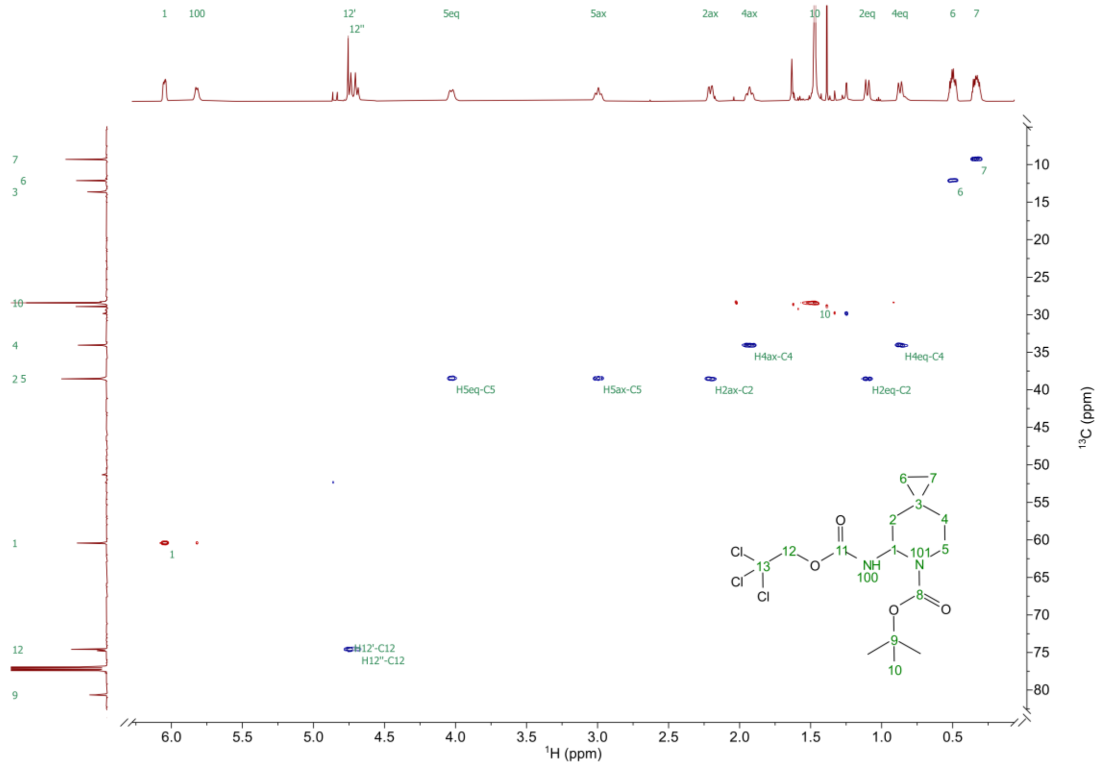

Compound **7t**: ^1H-^{13}C HMBC ($CDCl_3$, 298 K)

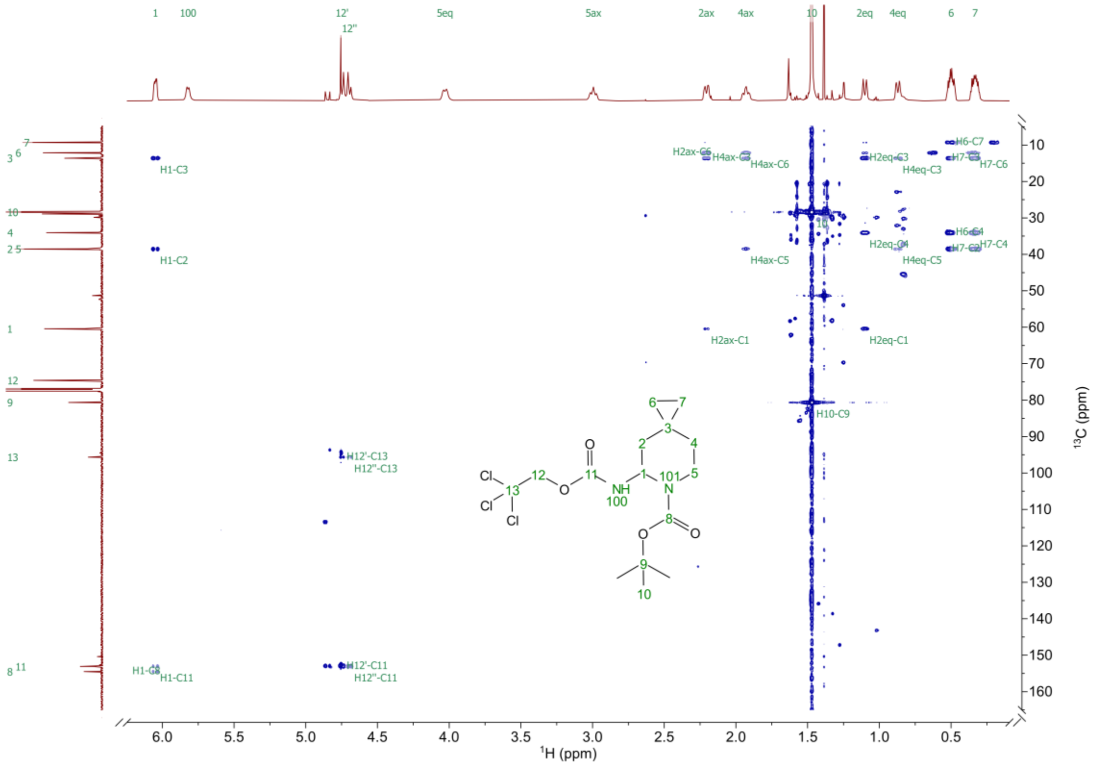

Compound **7t**: ^1H-^1H COSY ($CDCl_3$, 298 K)

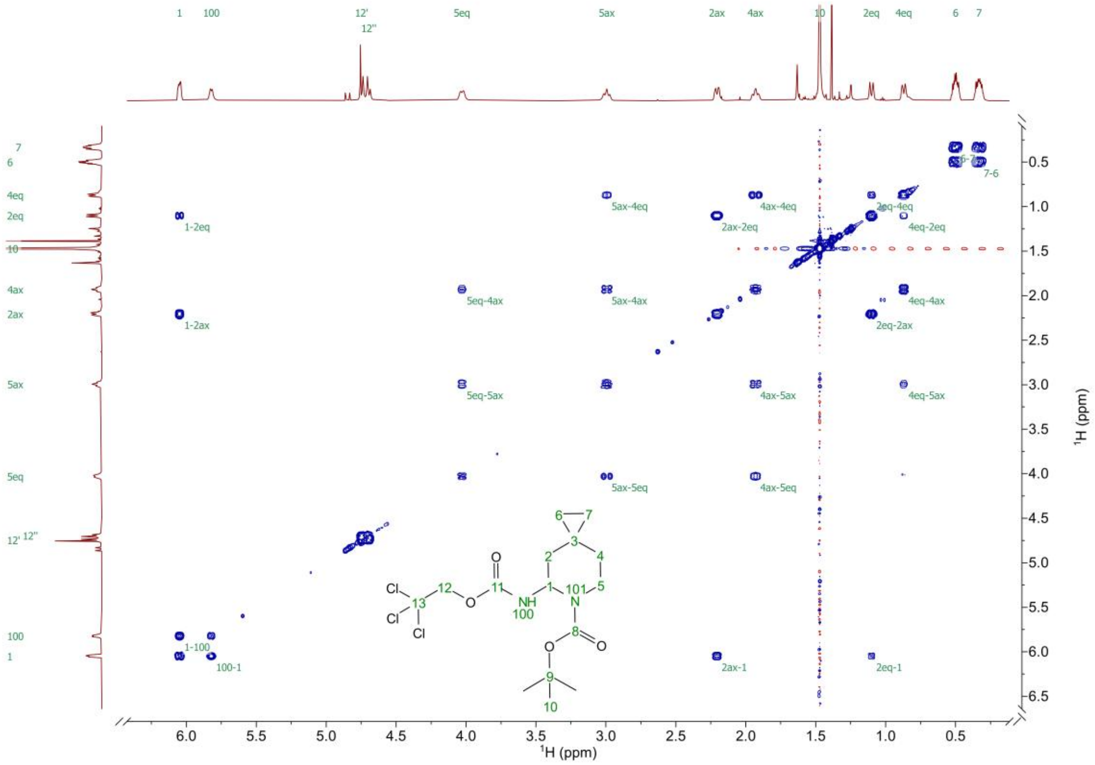

Compound **7t**: ^{1}H-^{1}H NOESY ($CDCl_3$, 298 K)

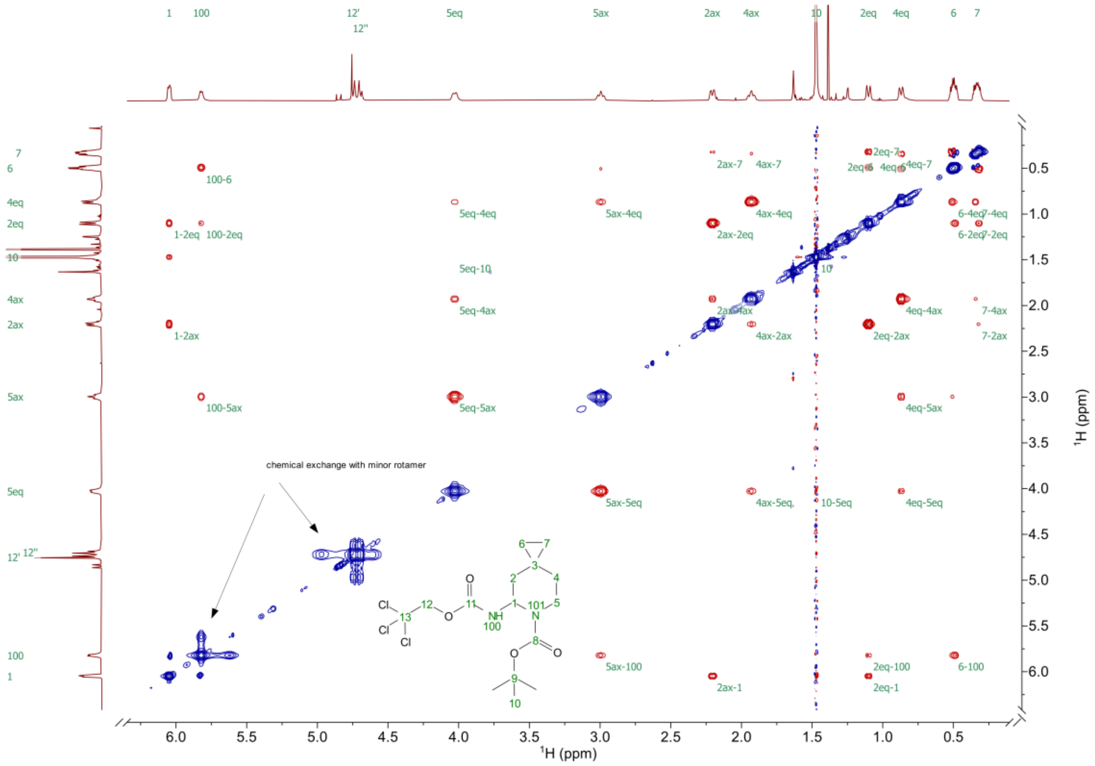

Compound **7t**: ^{1}H-^{15}N HMBC ($CDCl_3$, 298 K)

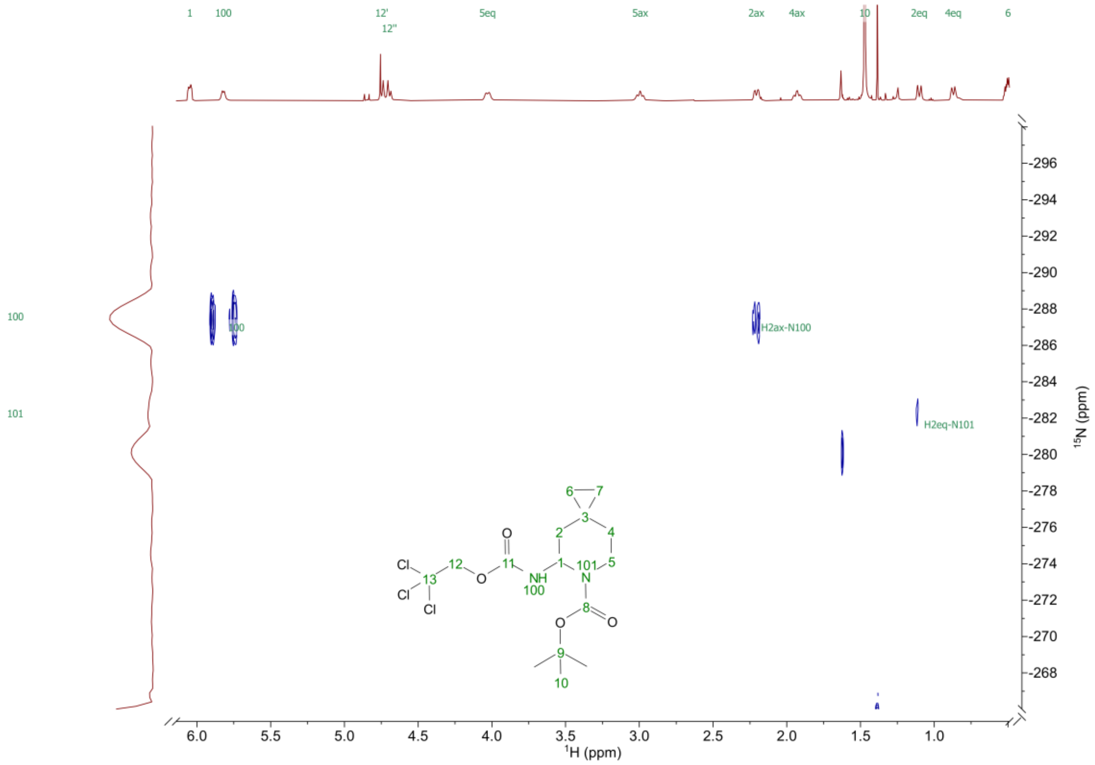

Compound **7u**: ^{1}H NMR (600 MHz, $CDCl_3$, 333 K)

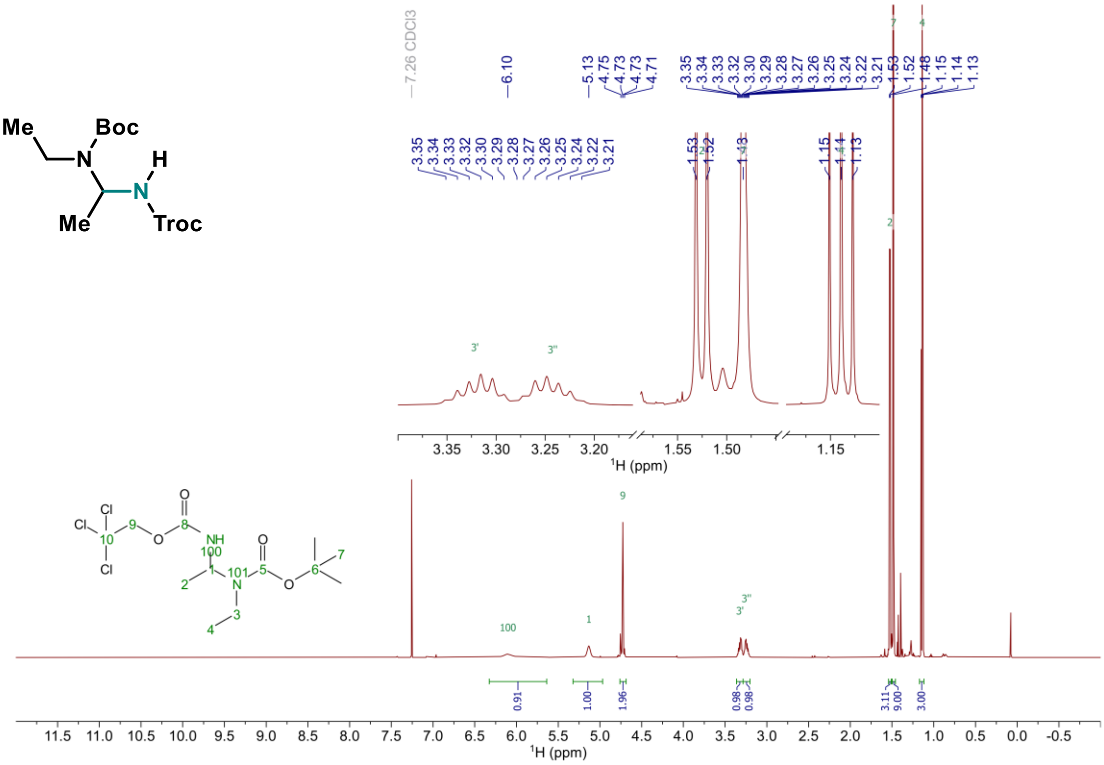

Compound **7u**: ^{13}C NMR (600 MHz, $CDCl_3$, 333 K)

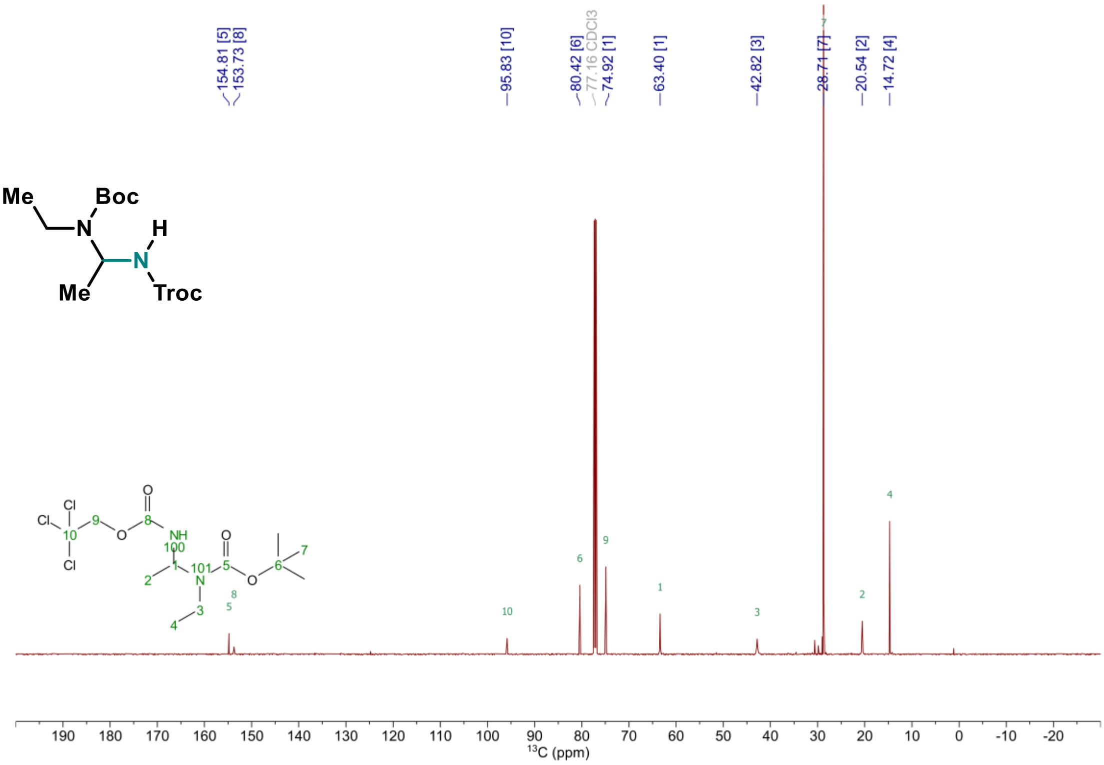

Compound **7u**: variable temperature ^{1}H NMR (600 MHz, $CDCl_3$)

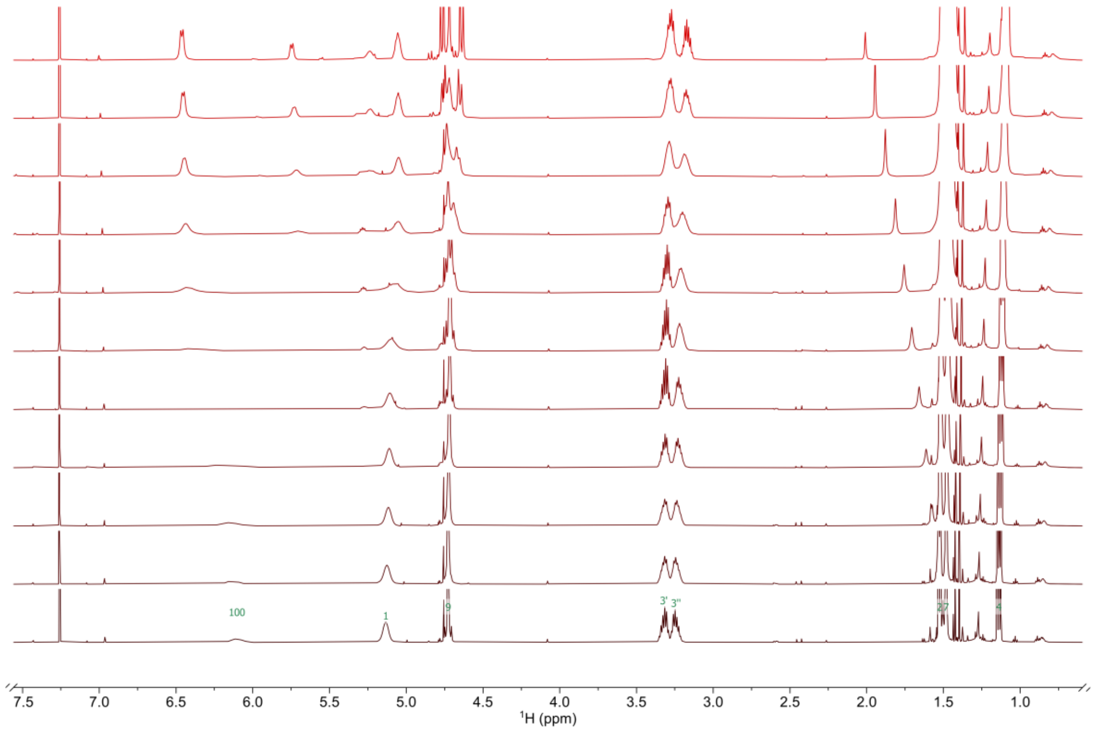

Compound **7u**: ^{1}H-^{13}C HSQC ($CDCl_3$, 333 K)

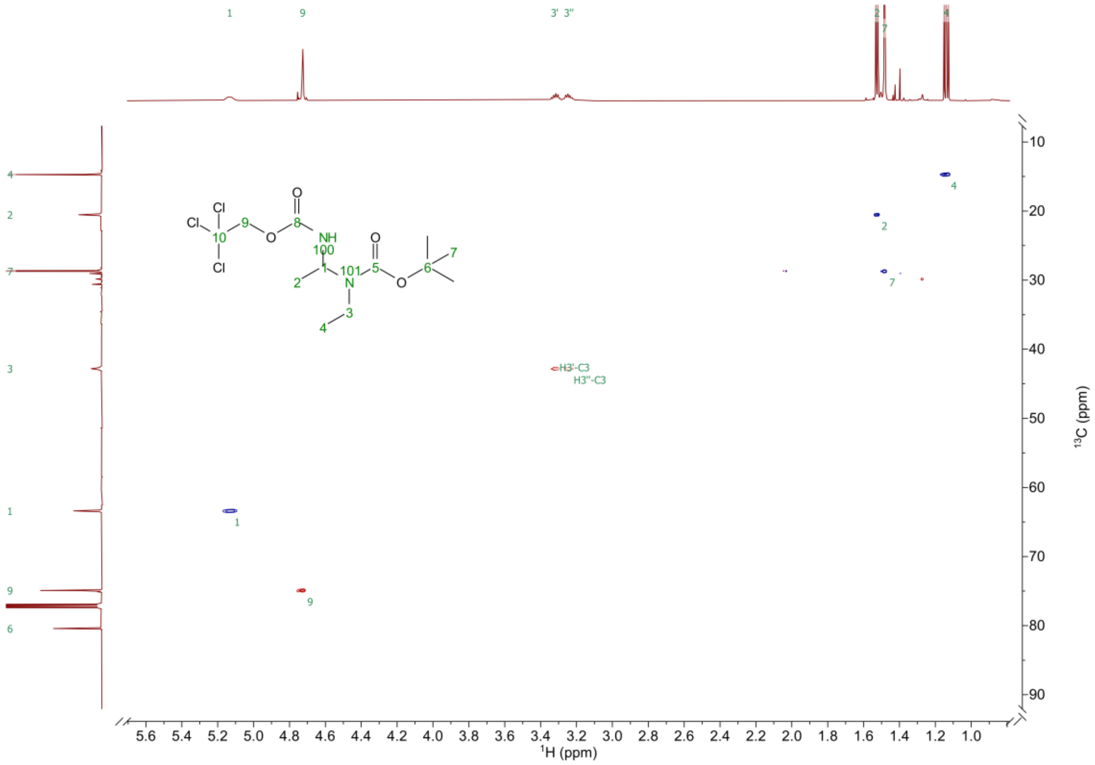

Compound **7u**: ^{1}H-^{13}C HMBC ($CDCl_3$, 333 K)

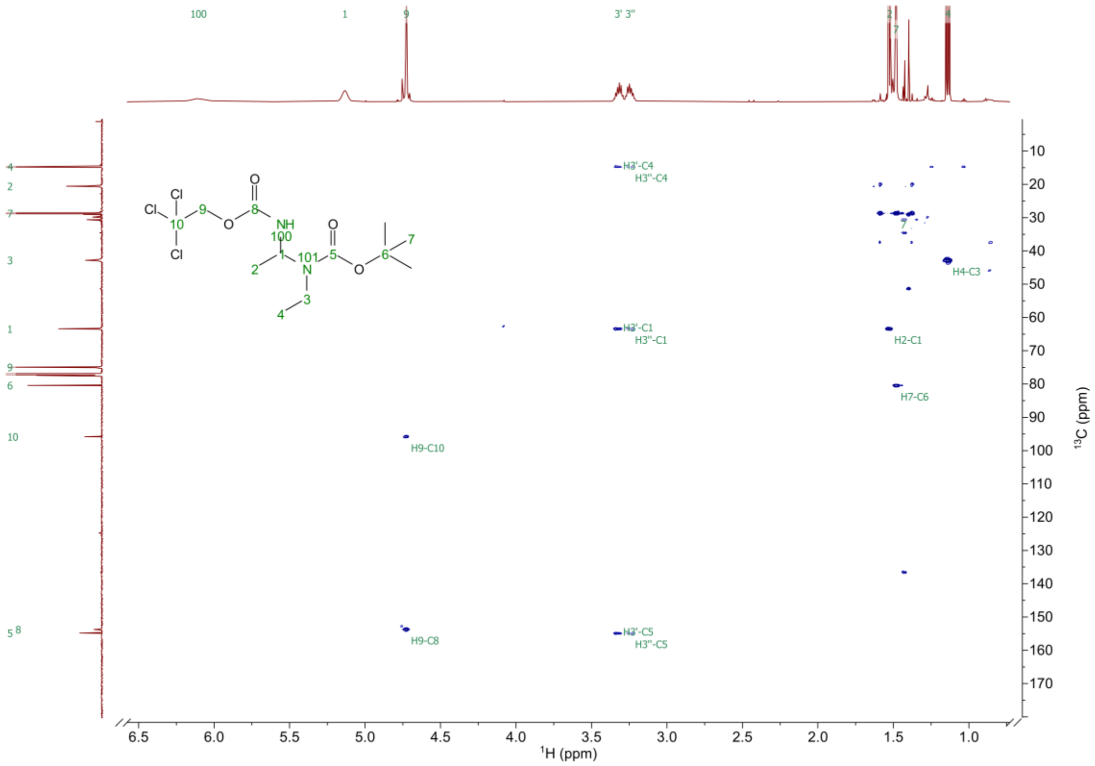

Compound **7u**: ^{1}H-^{1}H COSY ($CDCl_3$, 333 K)

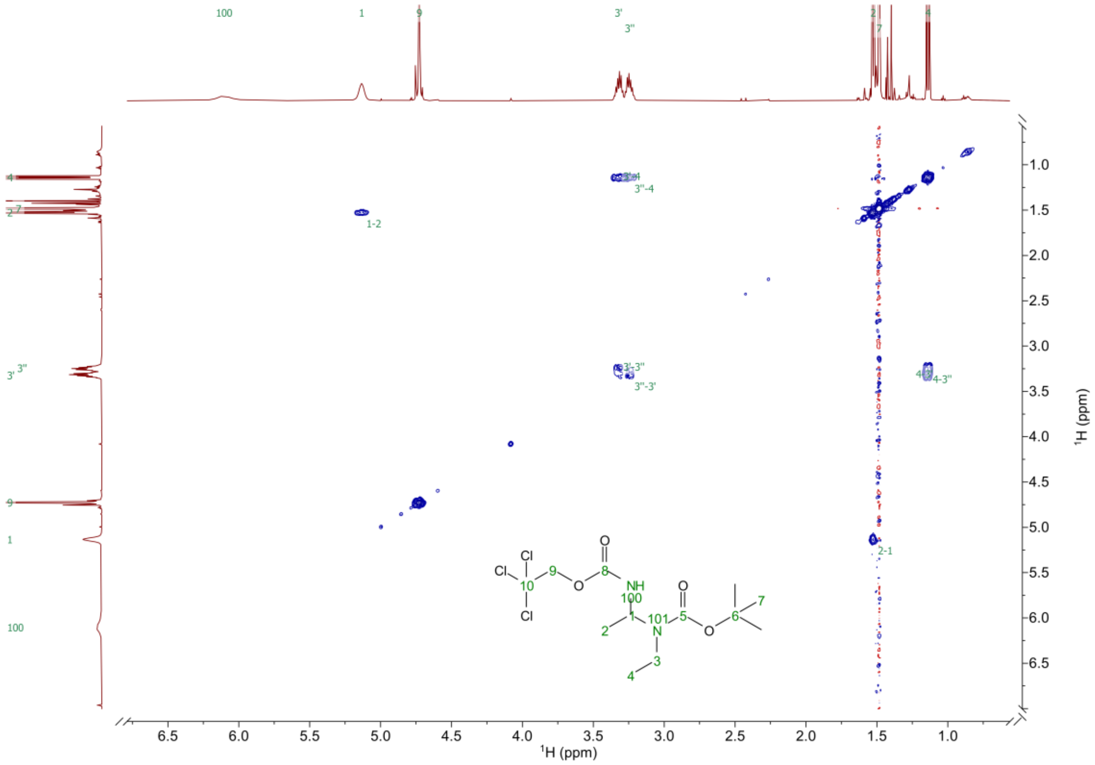

Compound **7u**: ^{1}H-^{1}H NOESY ($CDCl_3$, 333 K)

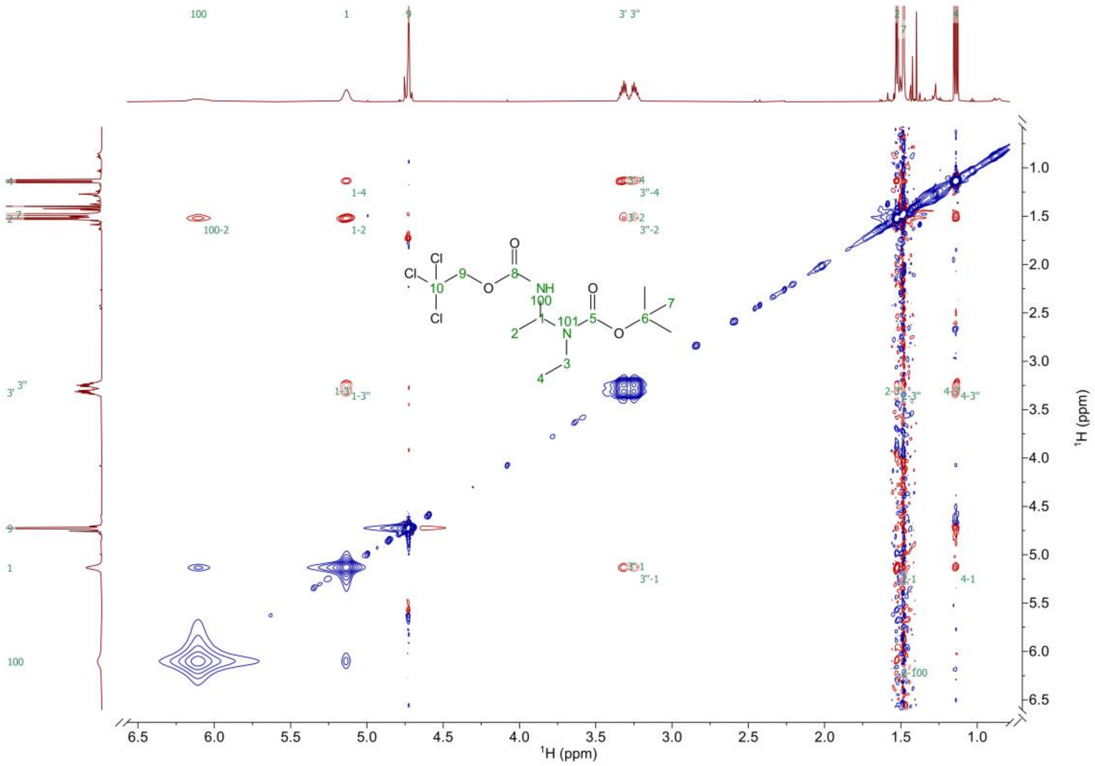

Compound **7u**: ^{1}H-^{15}N HMBC ($CDCl_3$, 333 K)

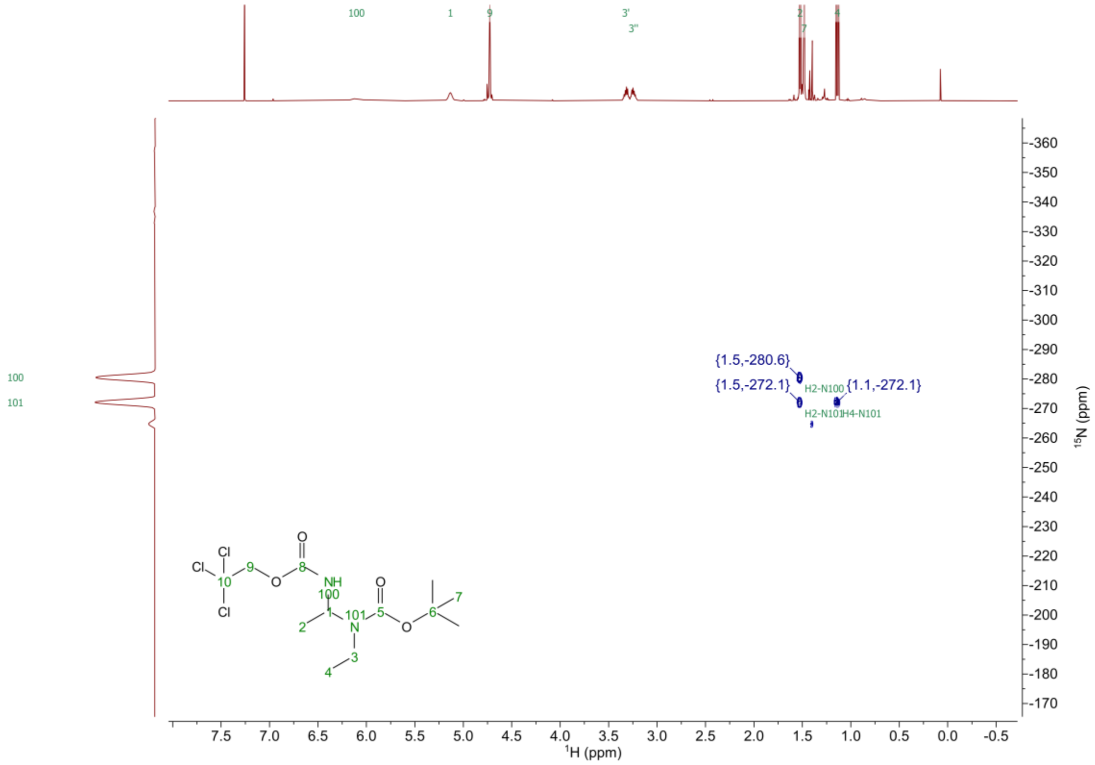

Compound **7v**: ^{1}H NMR (600 MHz, $CDCl_3$, 323 K)

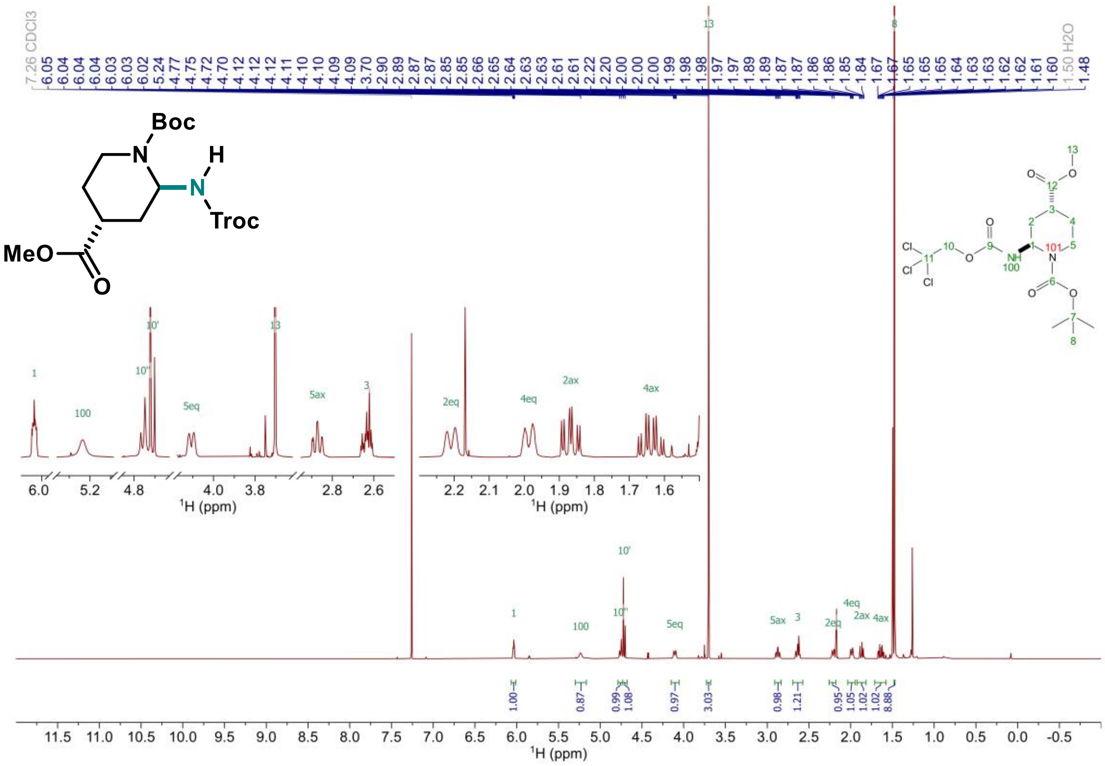

Compound **7v**: ^{13}C NMR (151 MHz, $CDCl_3$, 323 K)

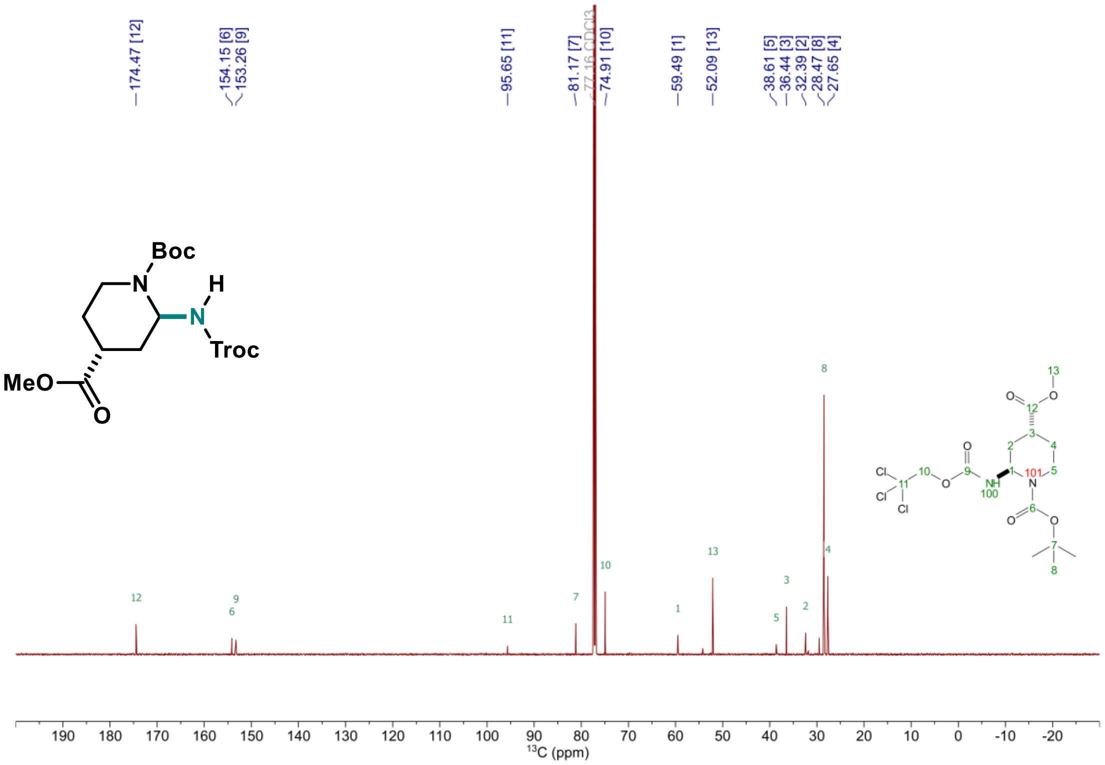

Compound **7v**: variable temperature ^{1}H NMR (600 MHz, $CDCl_3$)

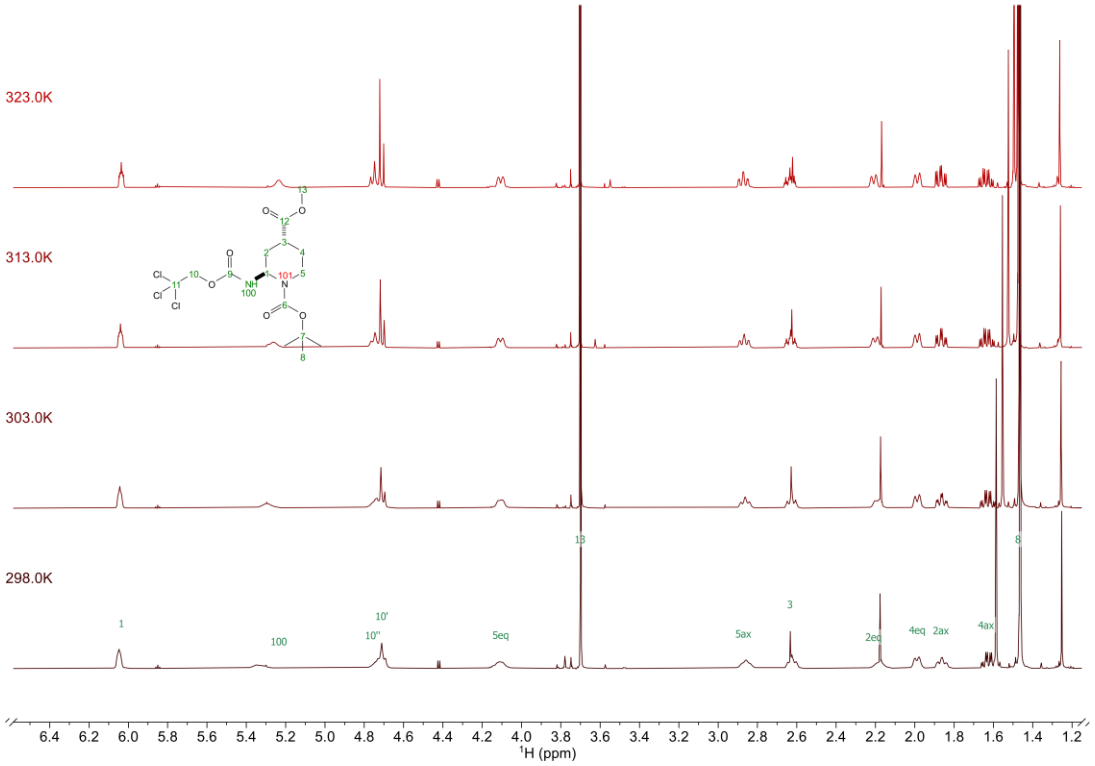

Compound **7v**: ^{1}H-^{13}C HSQC ($CDCl_3$, 323 K)

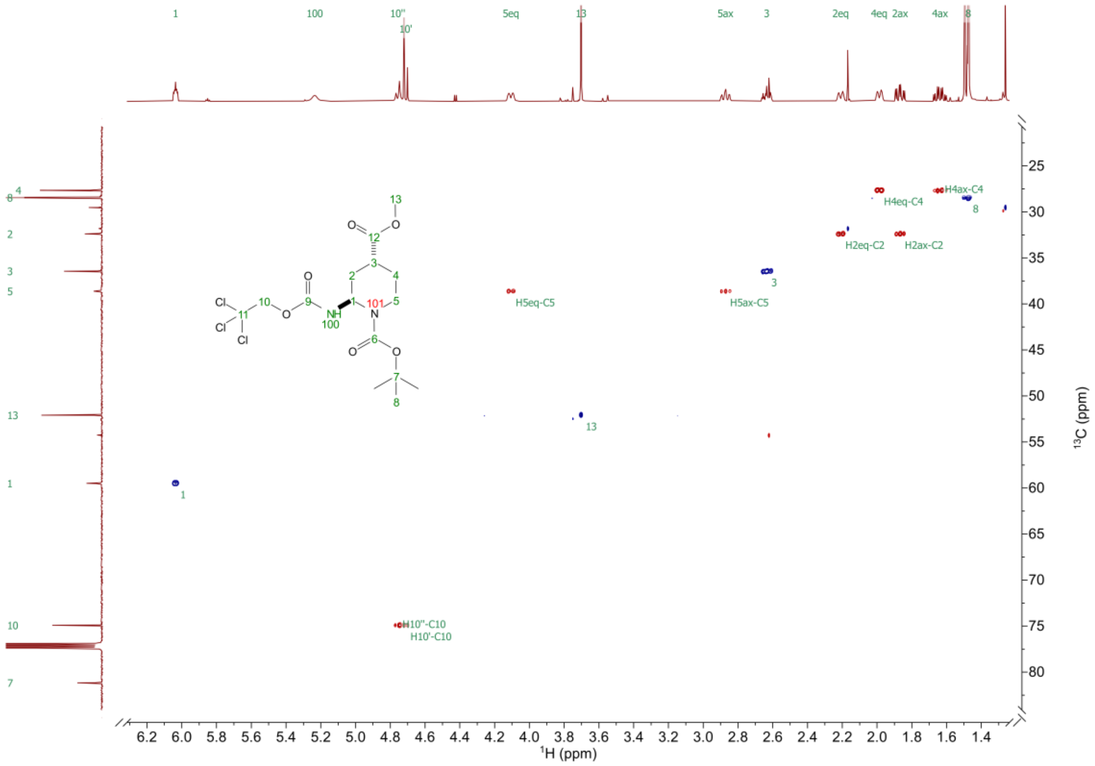

Compound **7v**: ^{1}H-^{13}C HMBC ($CDCl_3$, 323 K)

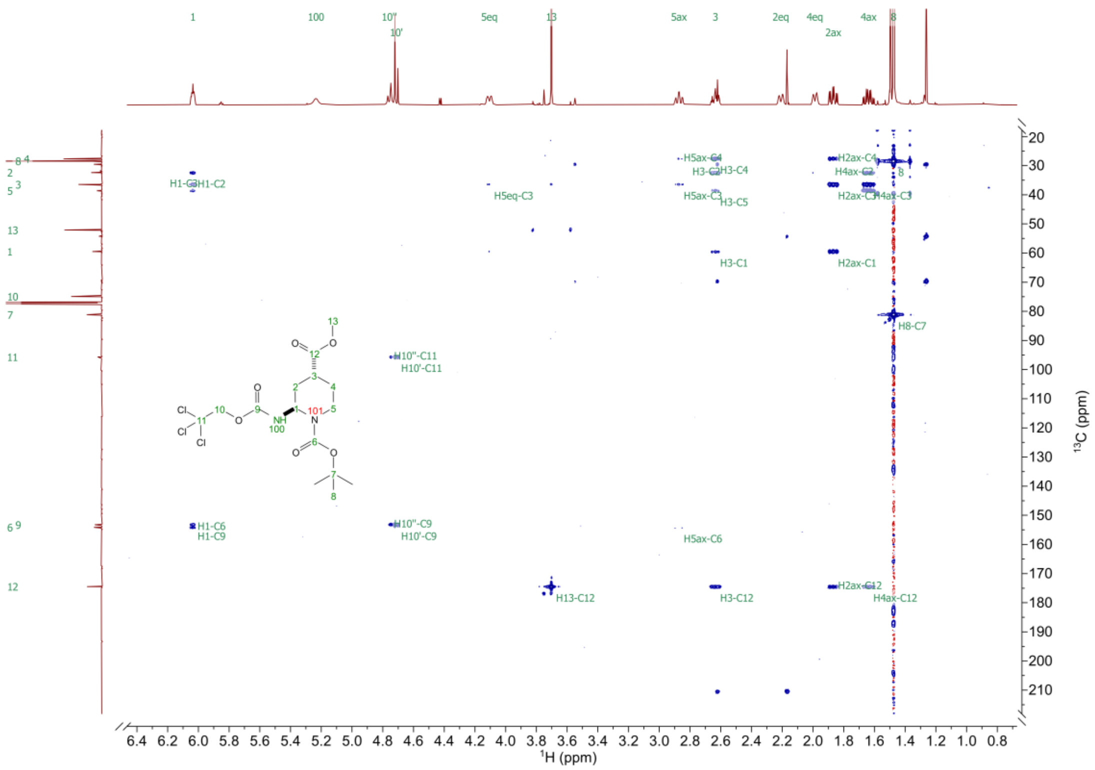

Compound **7v**: ^{1}H-^{1}H COSY ($CDCl_3$, 323 K)

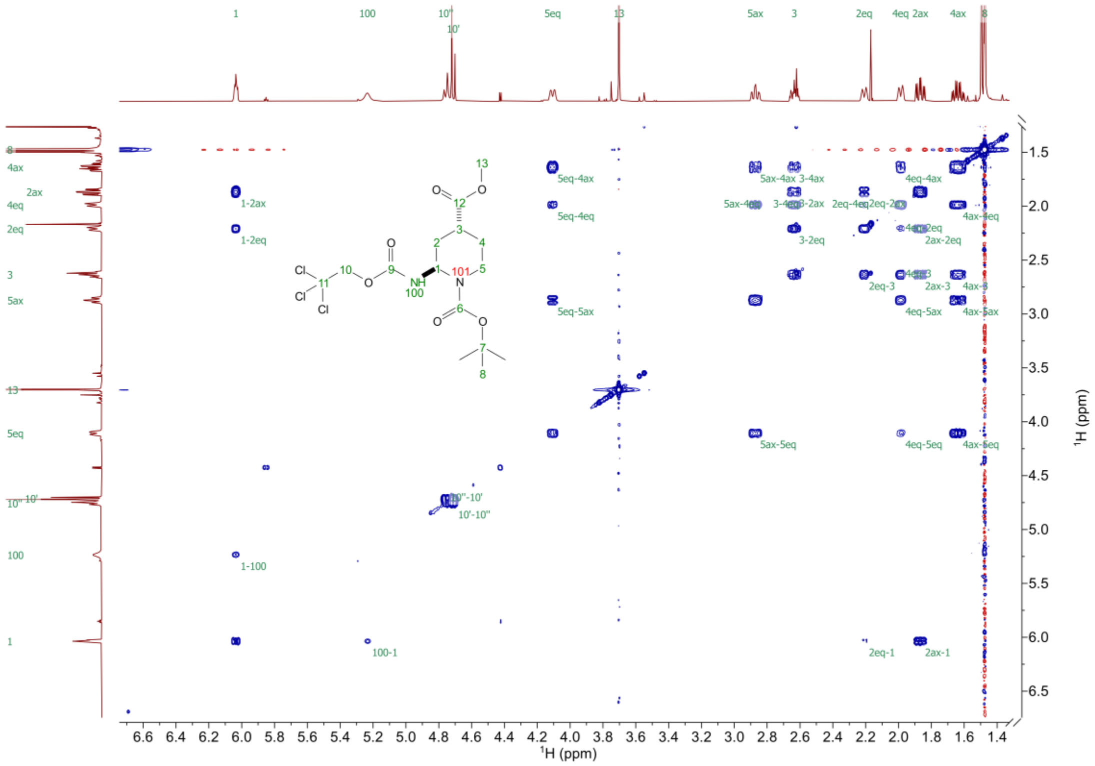

Compound **7v**: ^{1}H-^{1}H NOESY ($CDCl_3$, 323 K)

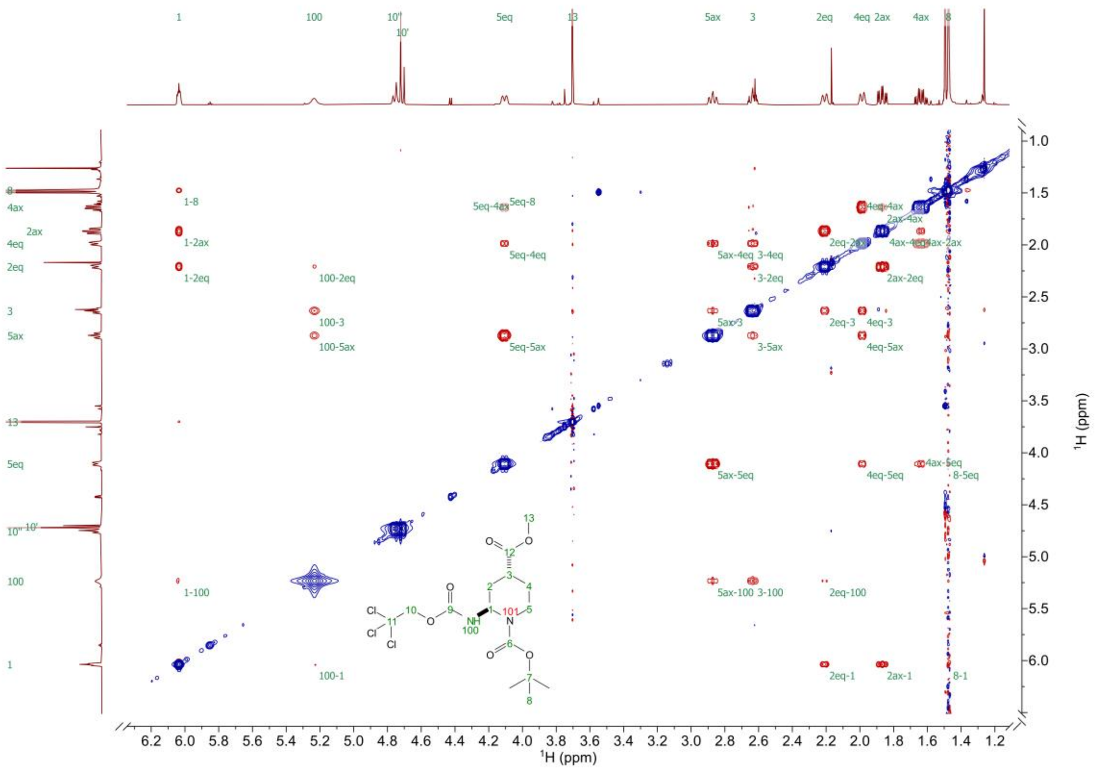

Compound **7v**: ^{1}H-^{15}N HMBC ($CDCl_3$, 323 K)

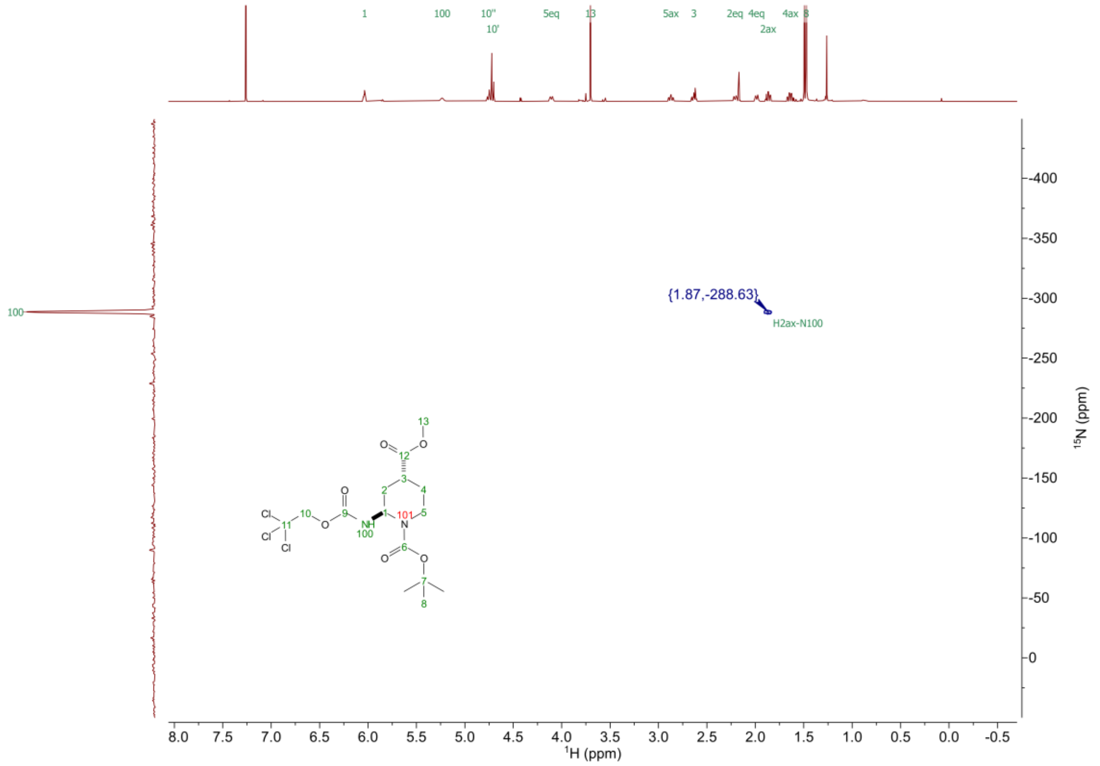

Compound **7w**, major diastereomer: ^{1}H NMR (600 MHz, $CDCl_3$, 323 K)

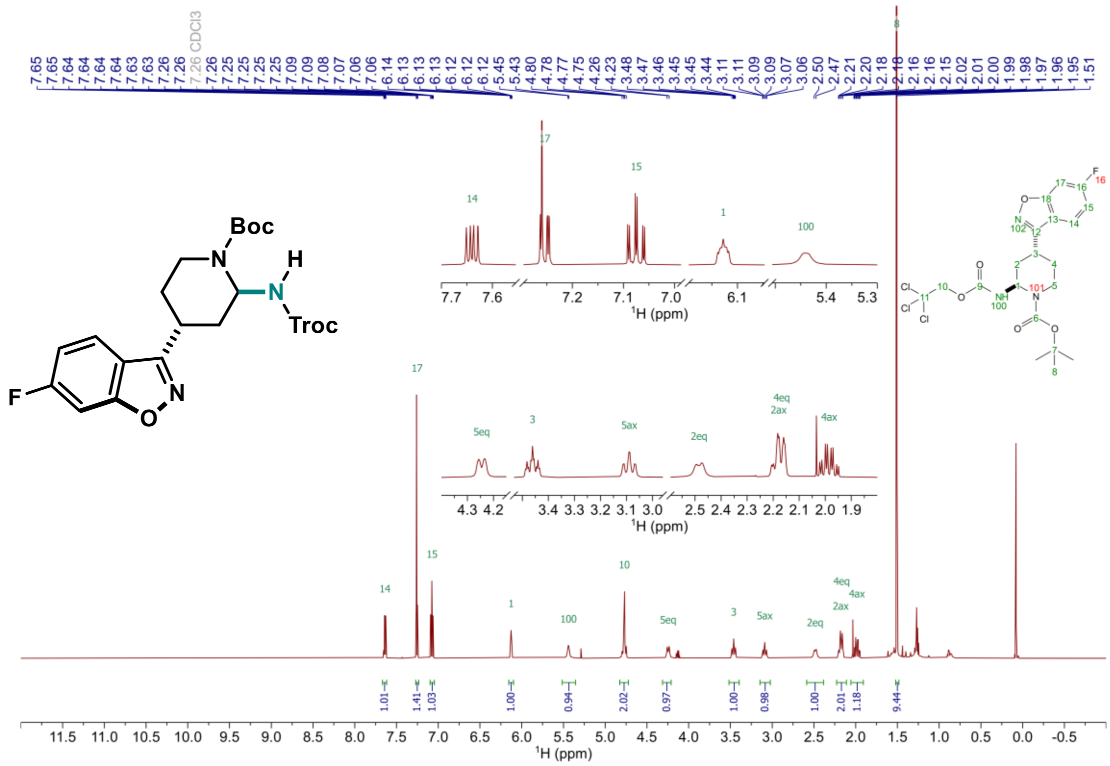

Compound **7w**, major diastereomer: ^{13}C NMR (151 MHz, $CDCl_3$, 323 K)

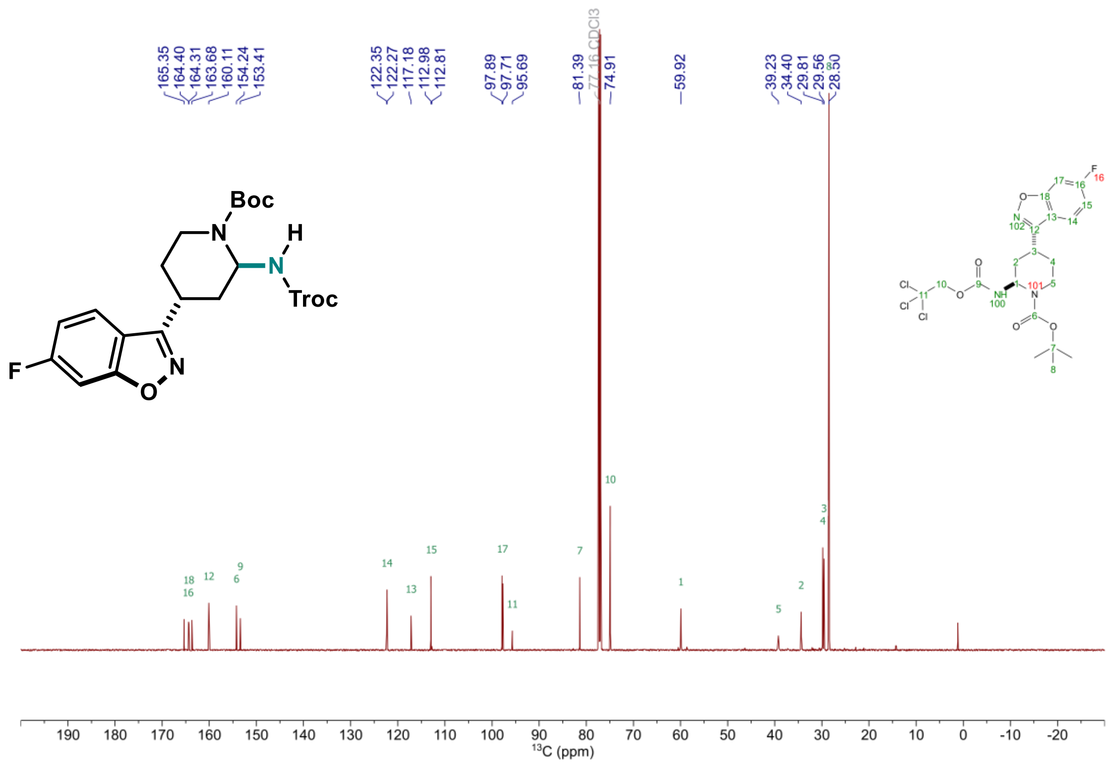

Compound **7w**, major diastereomer: ^{19}F NMR (565 MHz, $CDCl_3$, 323 K)

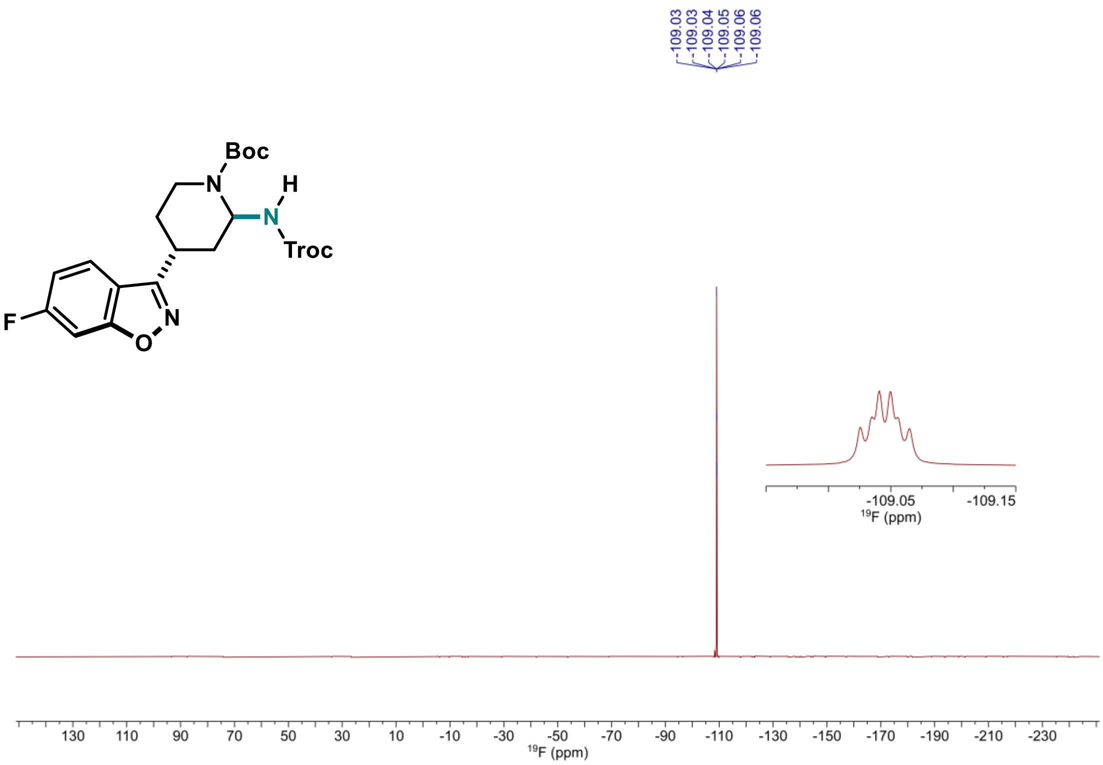

Compound **7w**, major diastereomer: variable temperature ^{1}H NMR (600 MHz, $CDCl_3$)

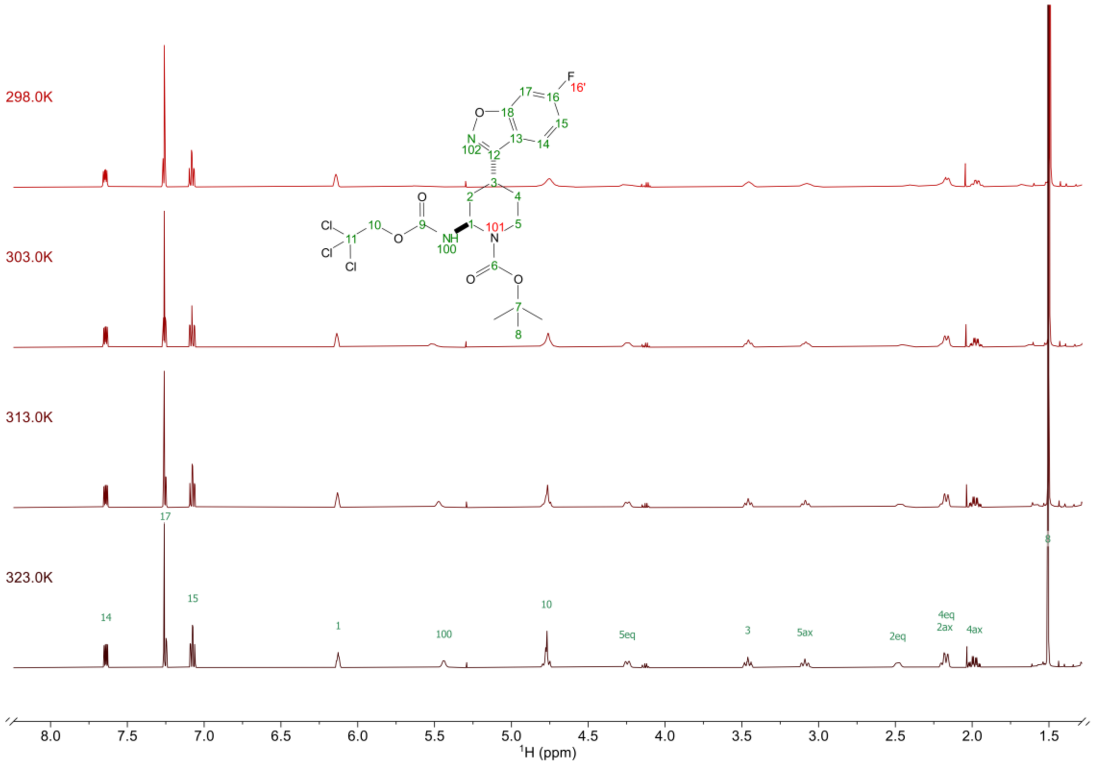

Compound **7w**, major diastereomer: ^{1}H-^{13}C HSQC ($CDCl_3$, 323 K)

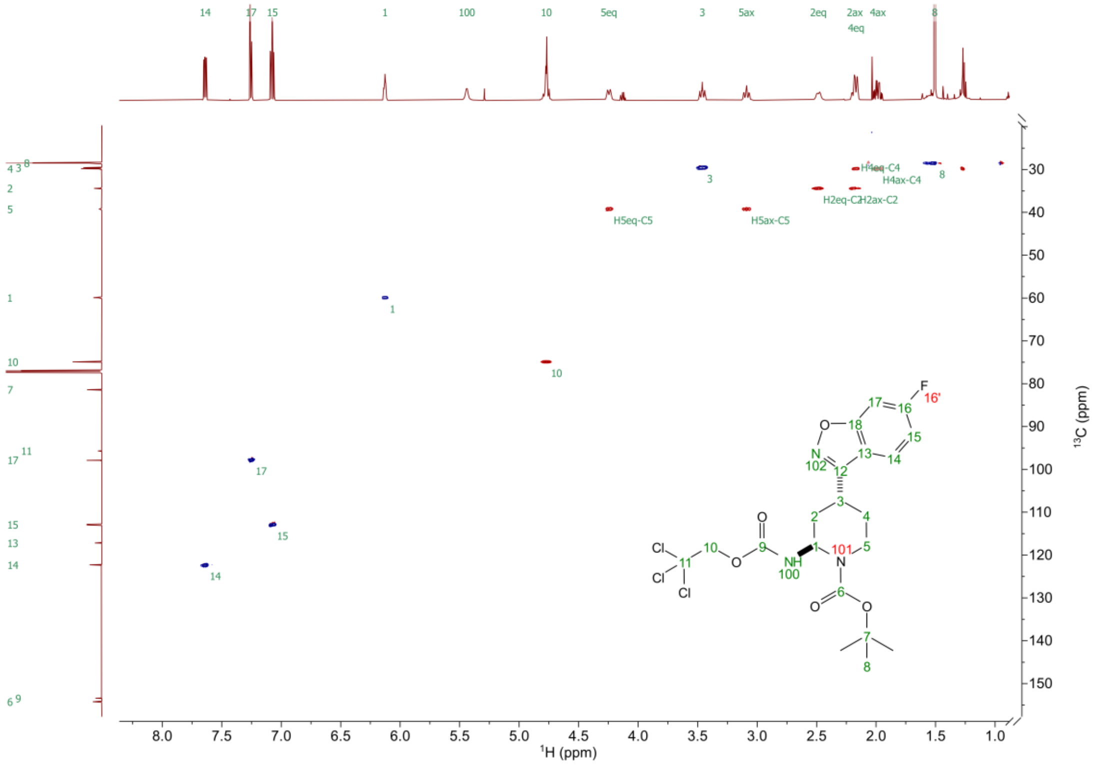

Compound **7w**, major diastereomer: ^{1}H-^{13}C HMBC ($CDCl_3$, 323 K)

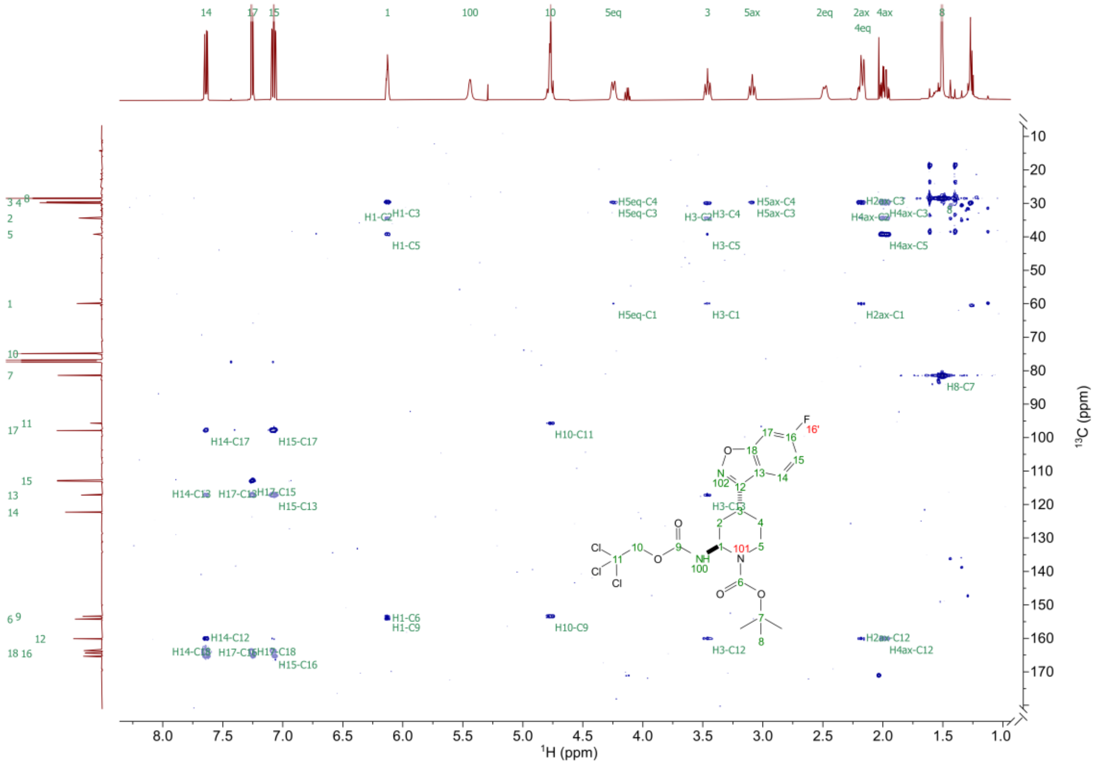

Compound **7w**, major diastereomer: ^{1}H-^{1}H COSY ($CDCl_3$, 323 K)

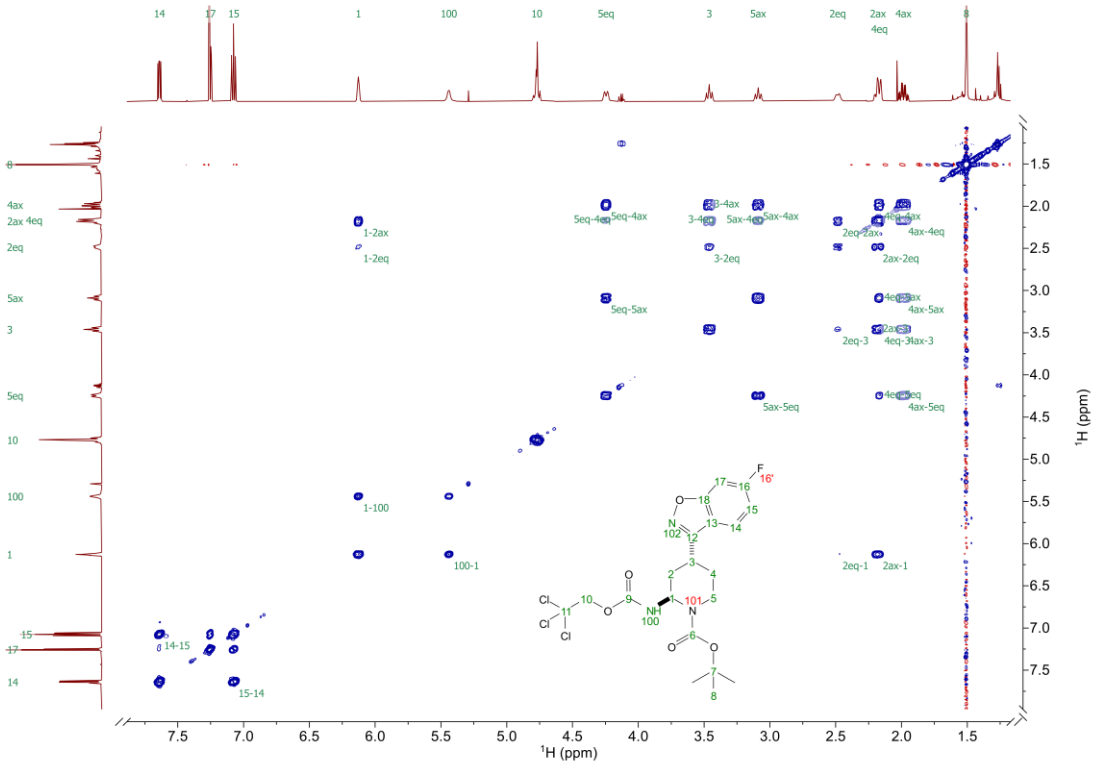

Compound **7w**, major diastereomer: ^{1}H-^{1}H NOESY ($CDCl_3$, 323 K)

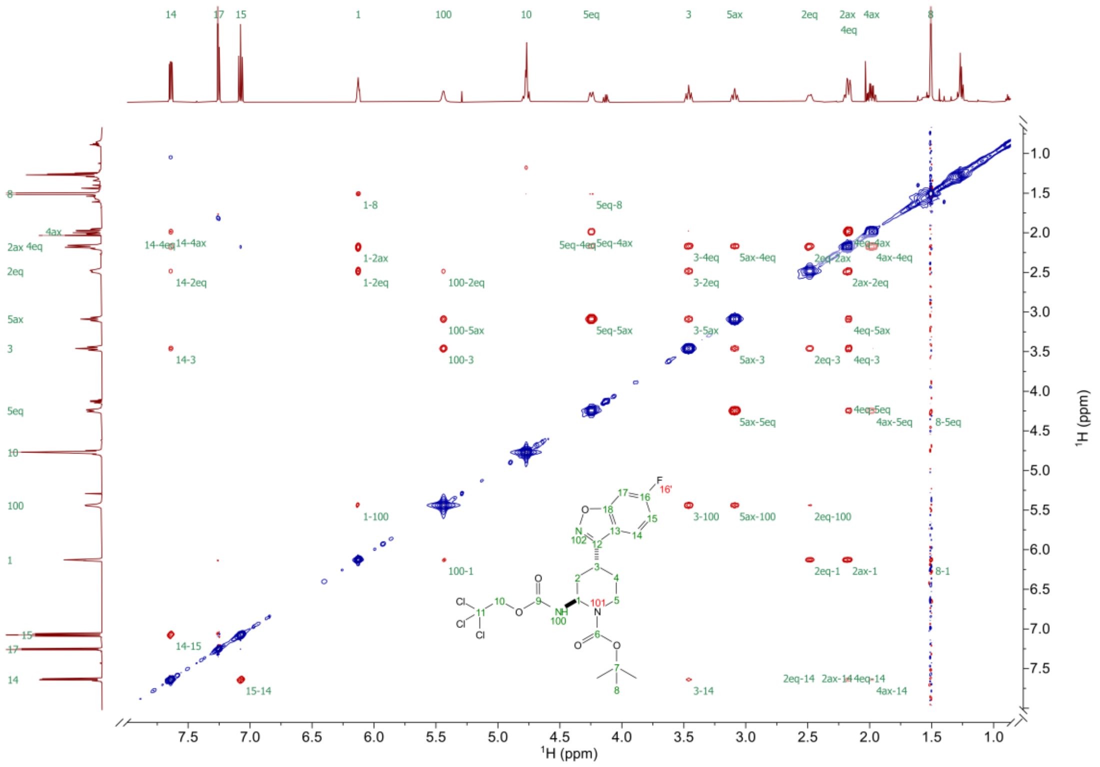

Compound **7w**, major diastereomer: ^{1}H-^{15}N HMBC ($CDCl_3$, 323 K)

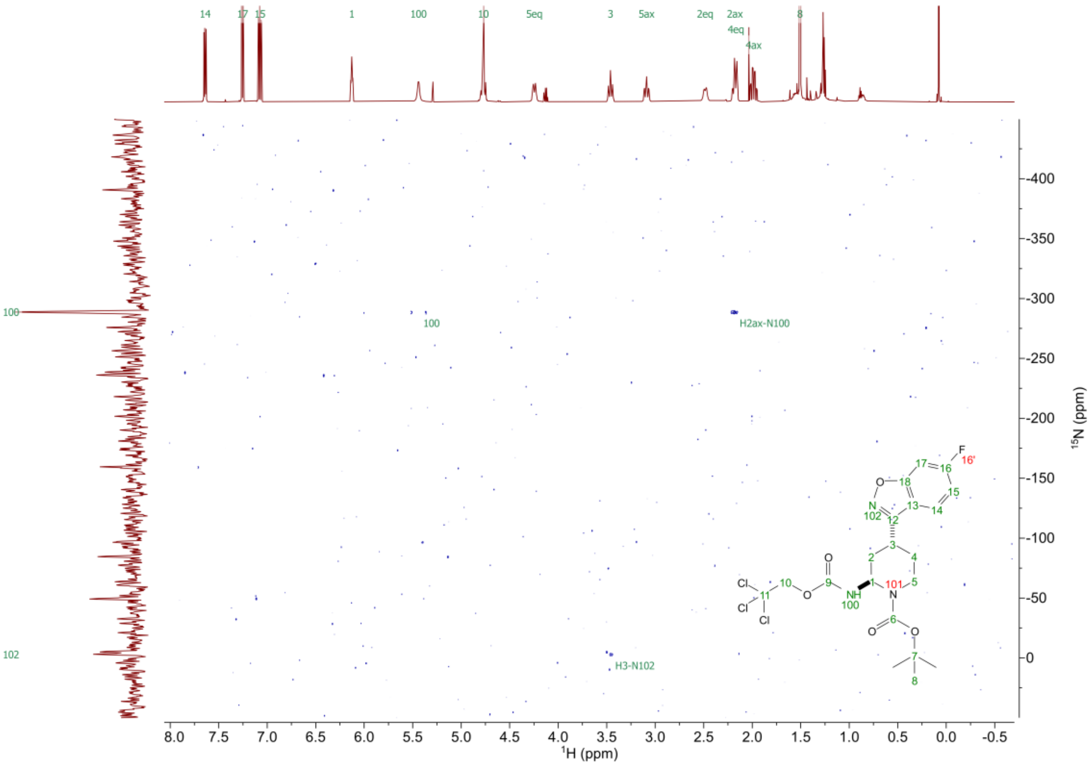

Compound **7w**, minor diastereomer: ^{1}H NMR (600 MHz, $CDCl_3$, 323 K)

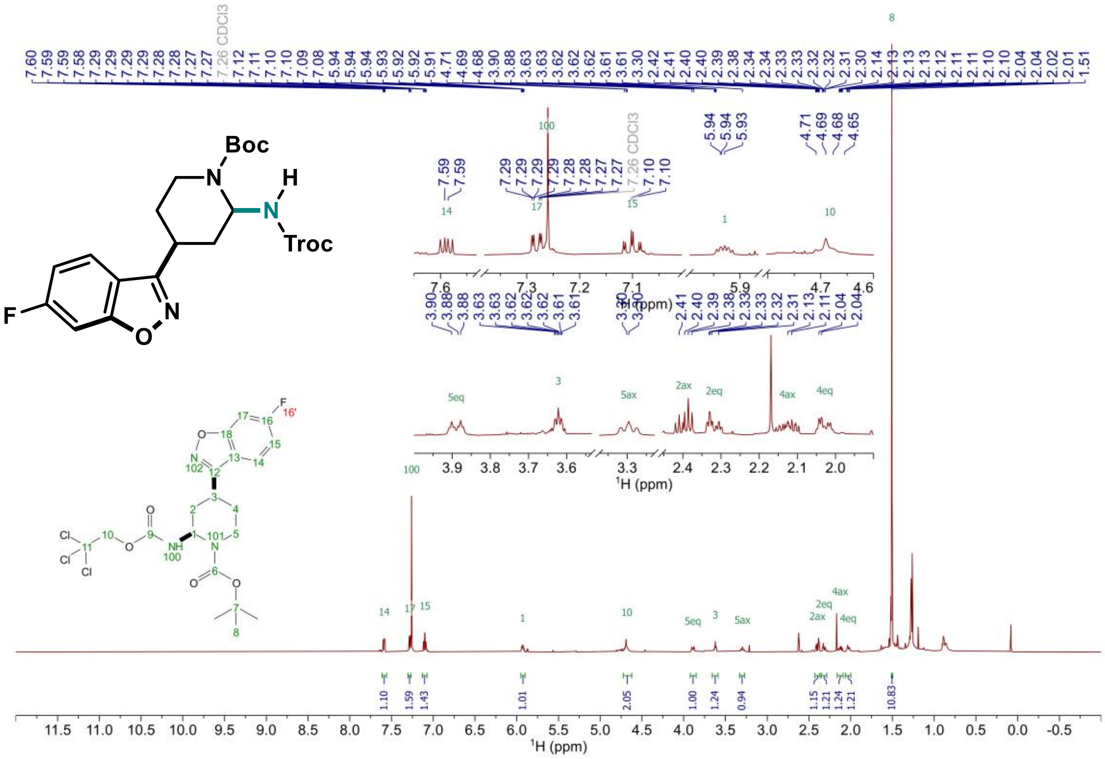

Compound **7w**, minor diastereomer: ^{13}C NMR (151 MHz, $CDCl_3$, 323 K)

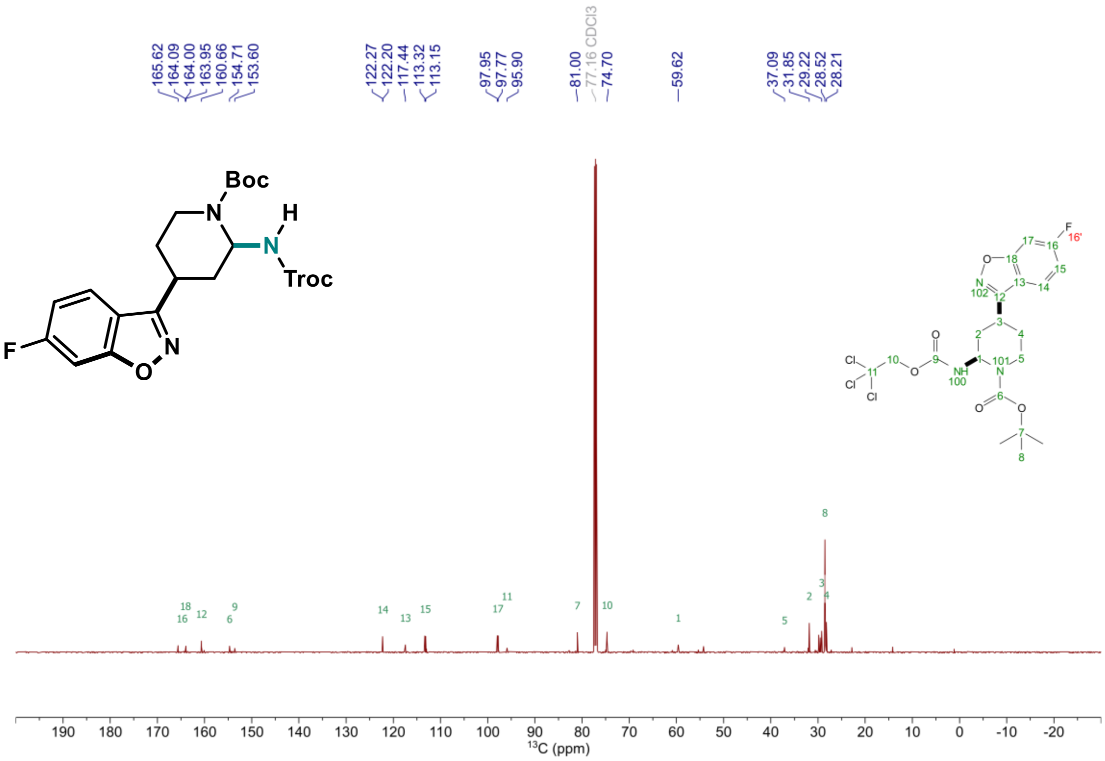

Compound **7w**, minor diastereomer: ^{19}F NMR (565 MHz, $CDCl_3$, 323 K)

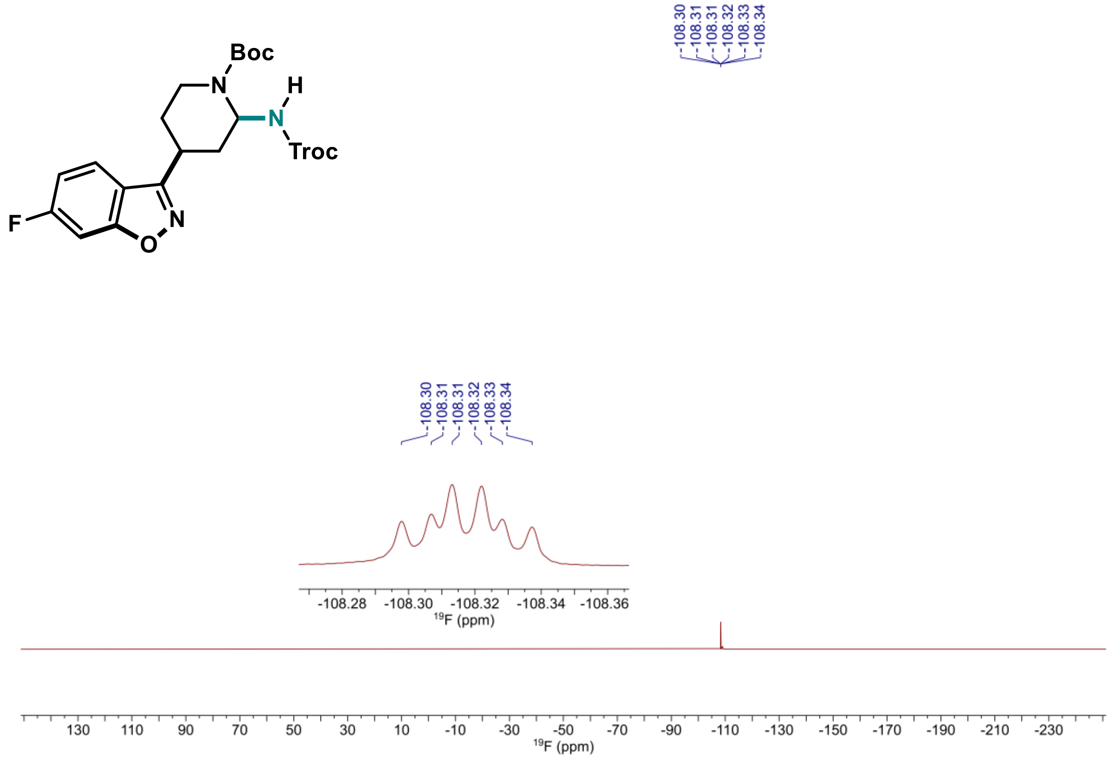

Compound **7w**, minor diastereomer: variable temperature ^{1}H NMR (600 MHz, $CDCl_3$)

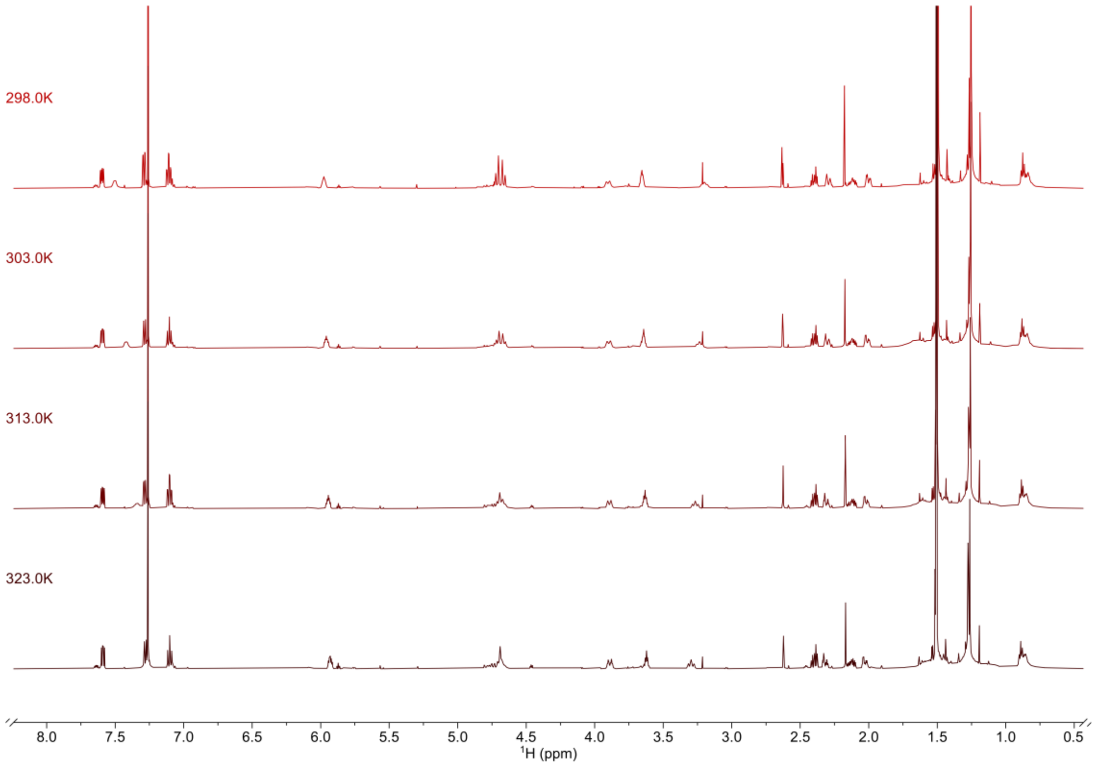

Compound **7w**, minor diastereomer: ^{1}H-^{13}C HSQC ($CDCl_3$, 323 K)

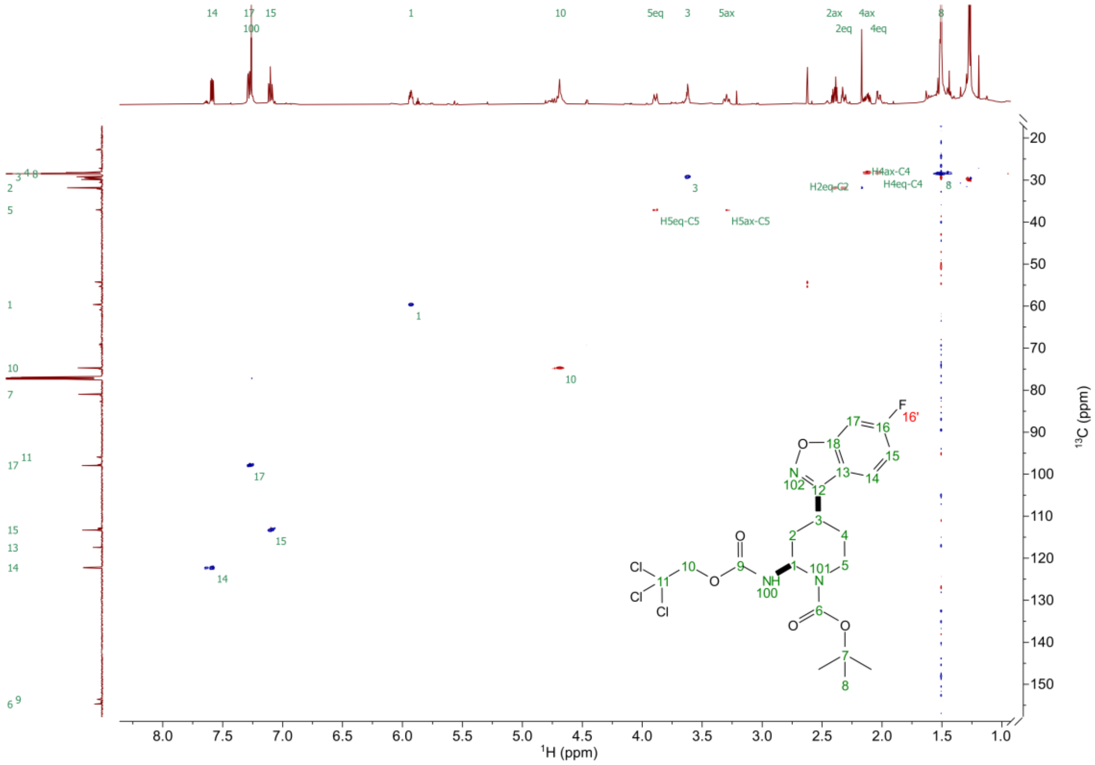

Compound **7w**, minor diastereomer: ^{1}H-^{13}C HMBC ($CDCl_3$, 323 K)

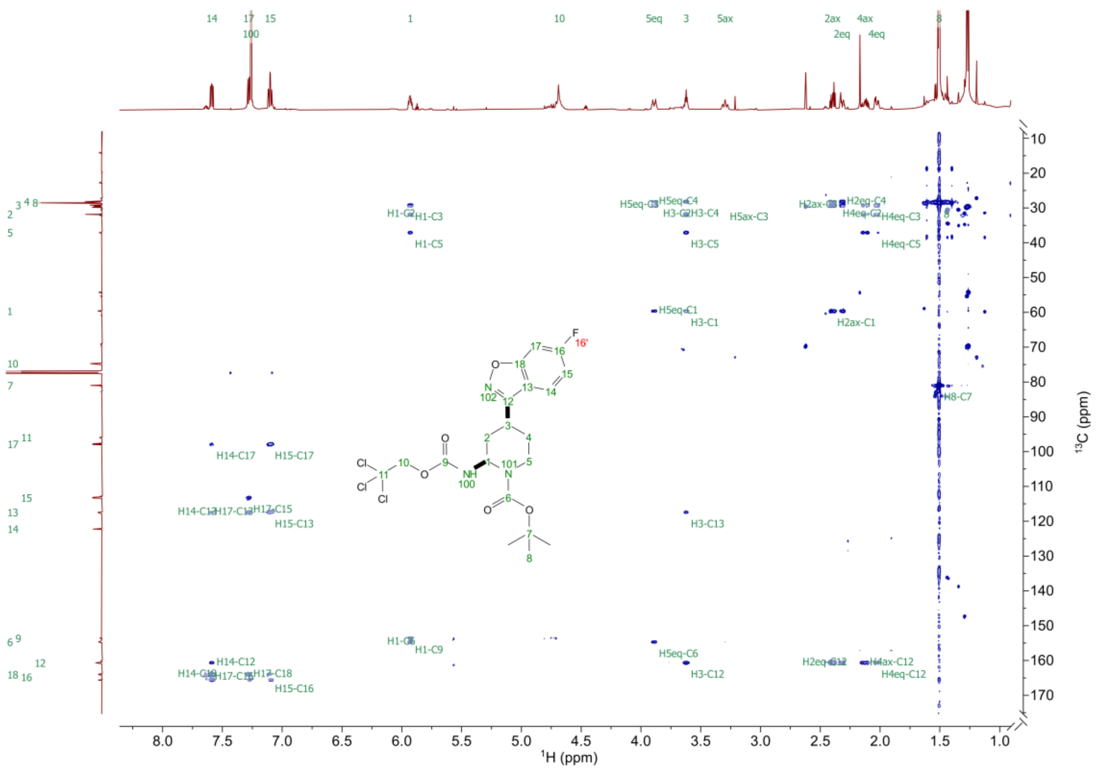

Compound **7w**, minor diastereomer: ^{1}H-^{1}H COSY ($CDCl_3$, 323 K)

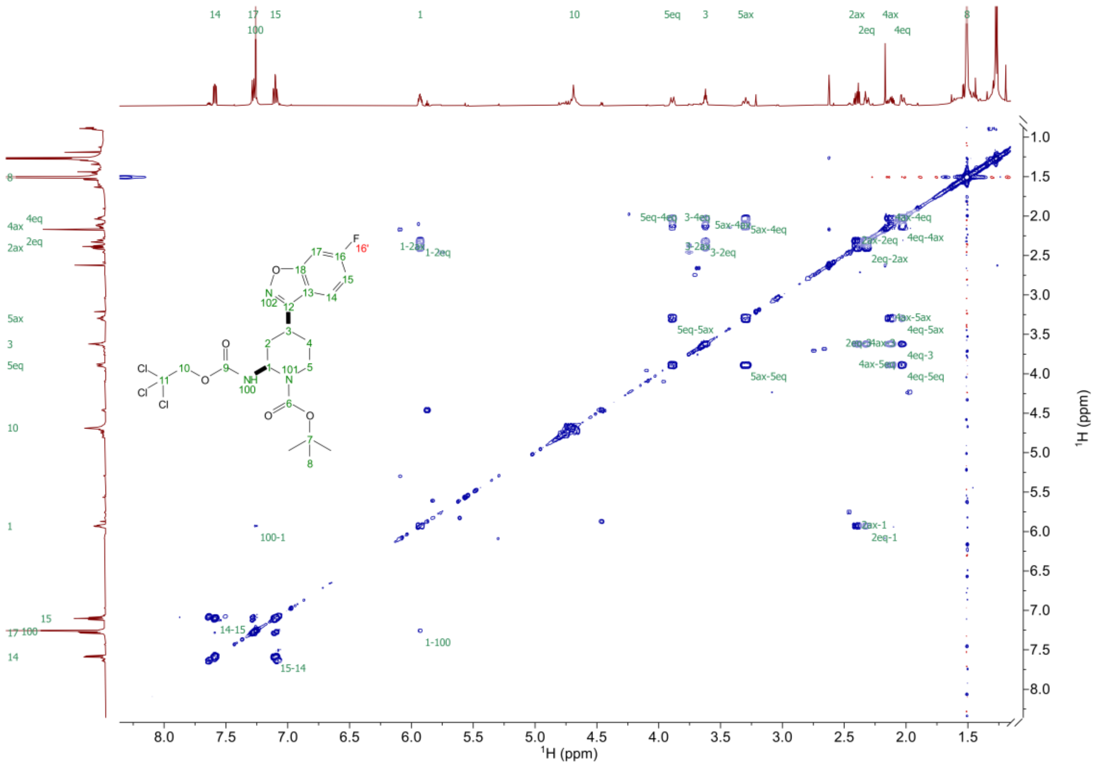

Compound **7w**, minor diastereomer: ^{1}H-^{1}H NOESY ($CDCl_3$, 323 K)

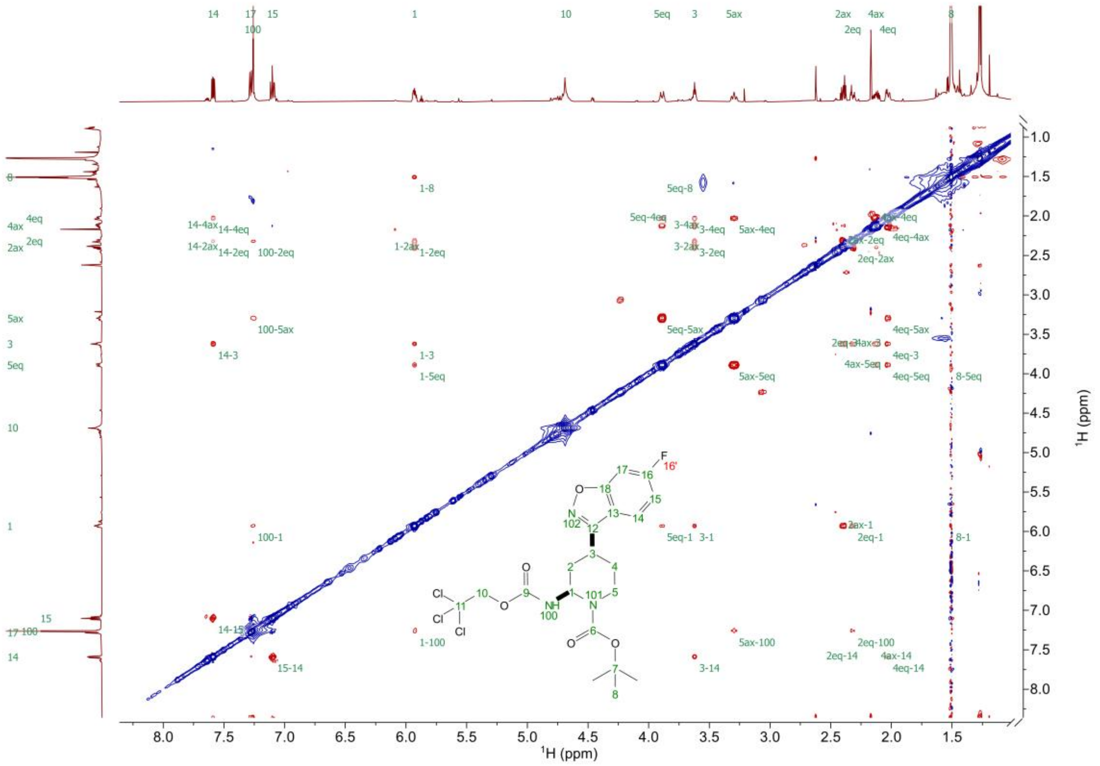

Compound **7w**, minor diastereomer: ^{1}H-^{15}N HMBC ($CDCl_3$, 323 K)

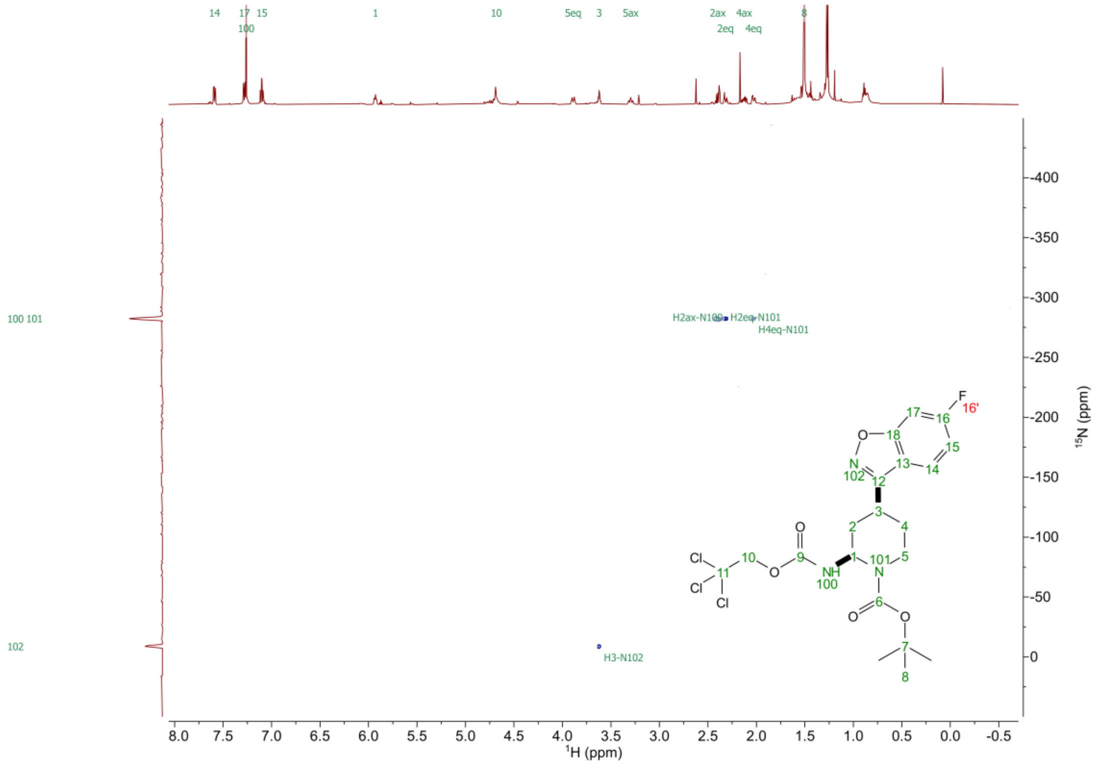

Compound **7x**: ^{1}H NMR (600 MHz, $CDCl_3$, 323 K)

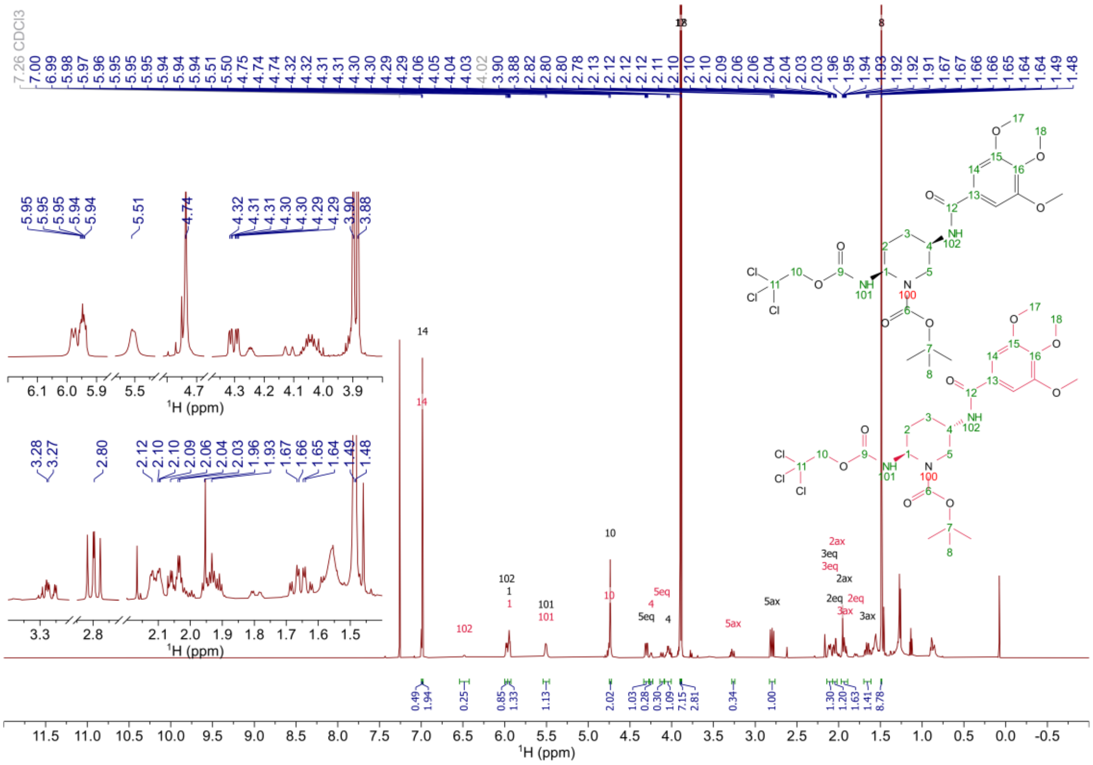

Compound **7x**: ^{13}C NMR (151 MHz, $CDCl_3$, 323 K)

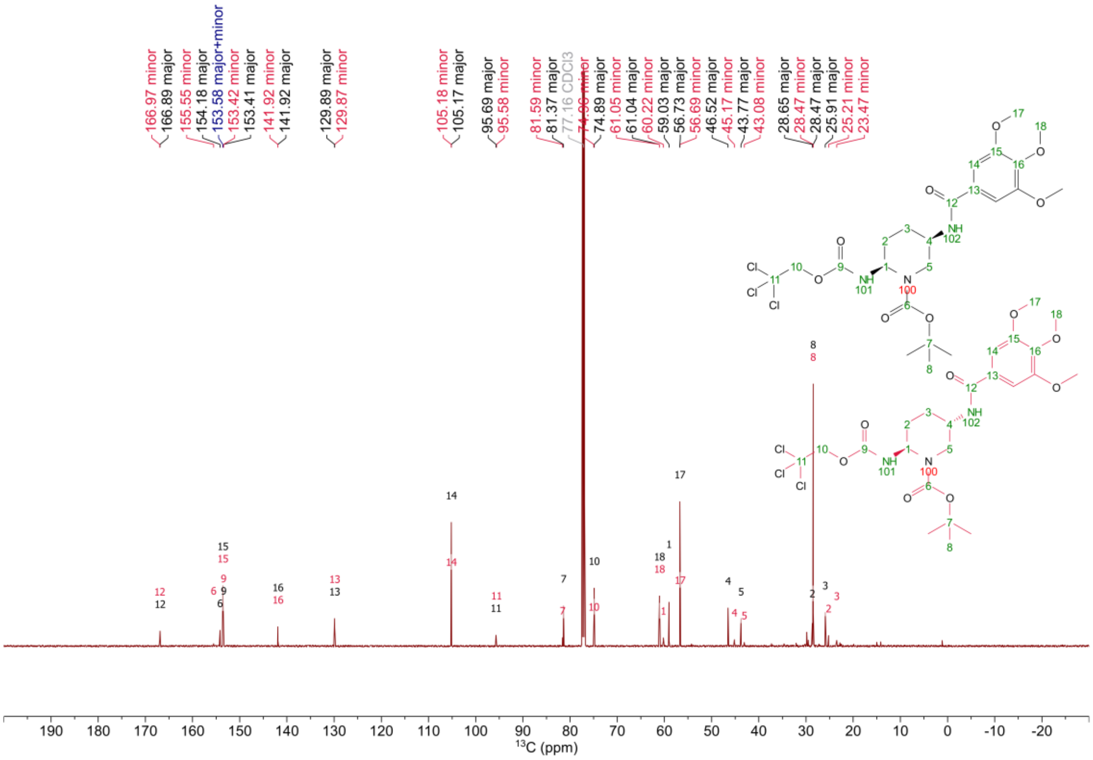

Compound **7x**: variable temperature ^{1}H NMR (600 MHz, $CDCl_3$)

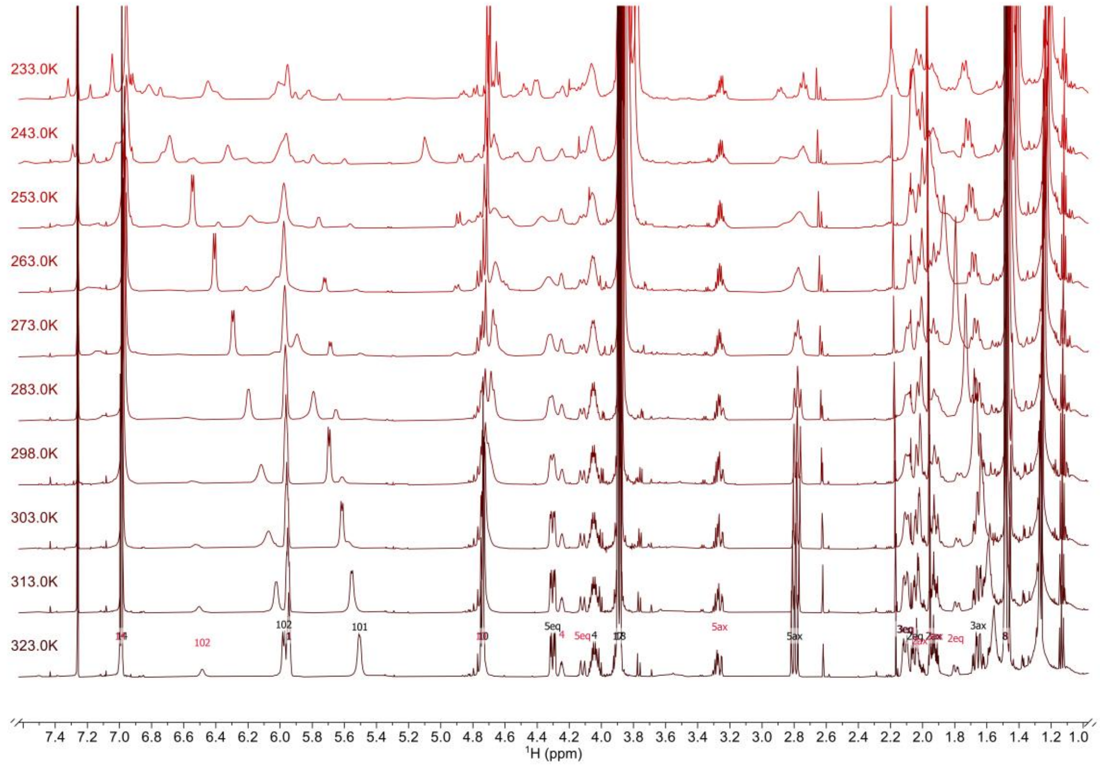

Compound **7x**: ^{1}H-^{13}C HSQC ($CDCl_3$, 323 K)

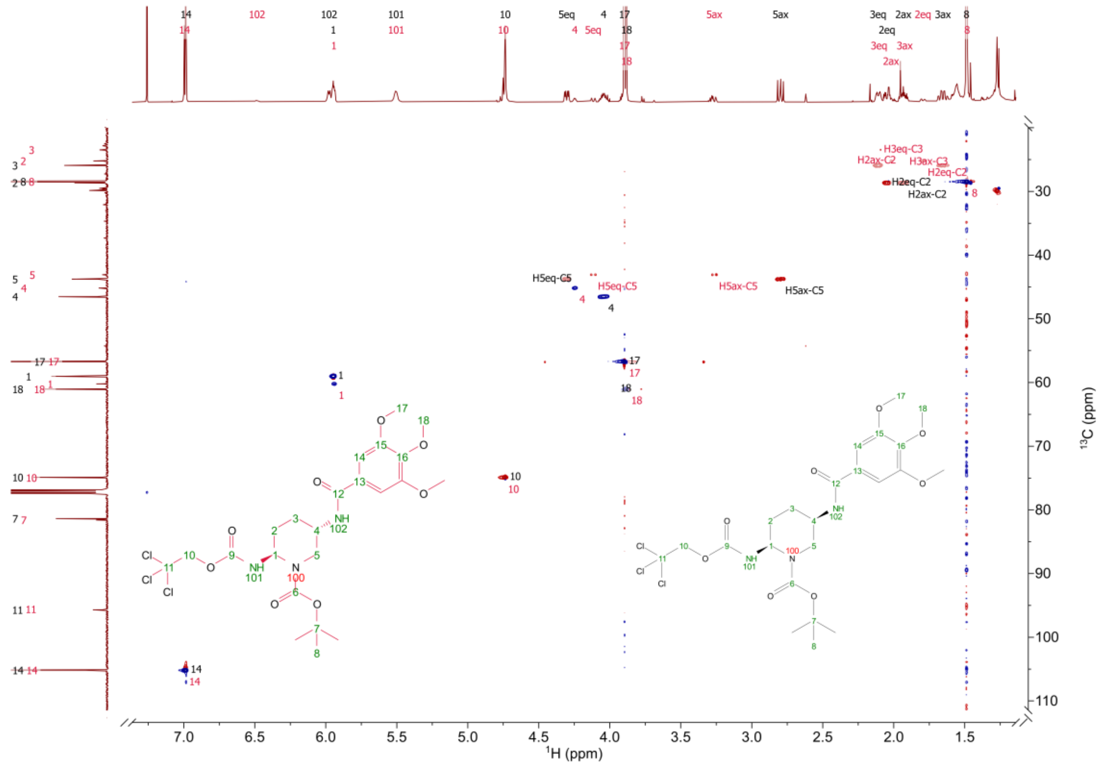

Compound **7x**: ^{1}H-^{13}C HMBC ($CDCl_3$, 323 K)

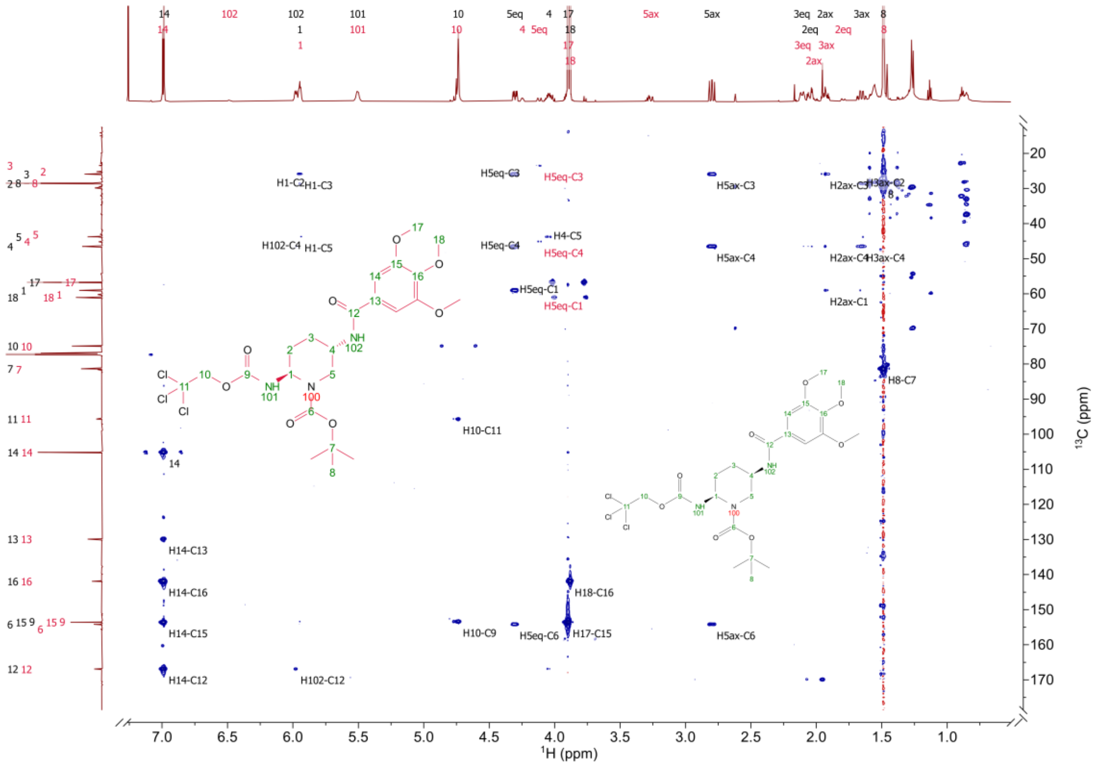

Compound **7x**: ^{1}H-^{1}H COSY ($CDCl_3$, 323 K)

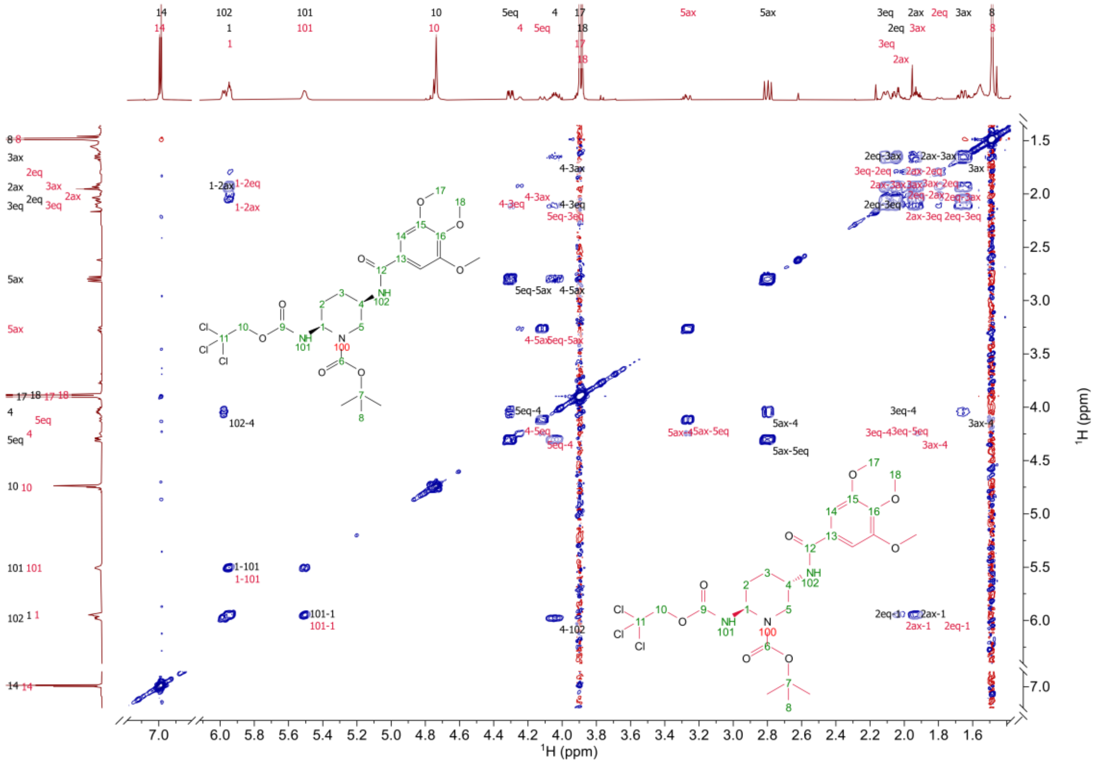

Compound **7x** ^{1}H-^{1}H NOESY ($CDCl_3$, 323 K)

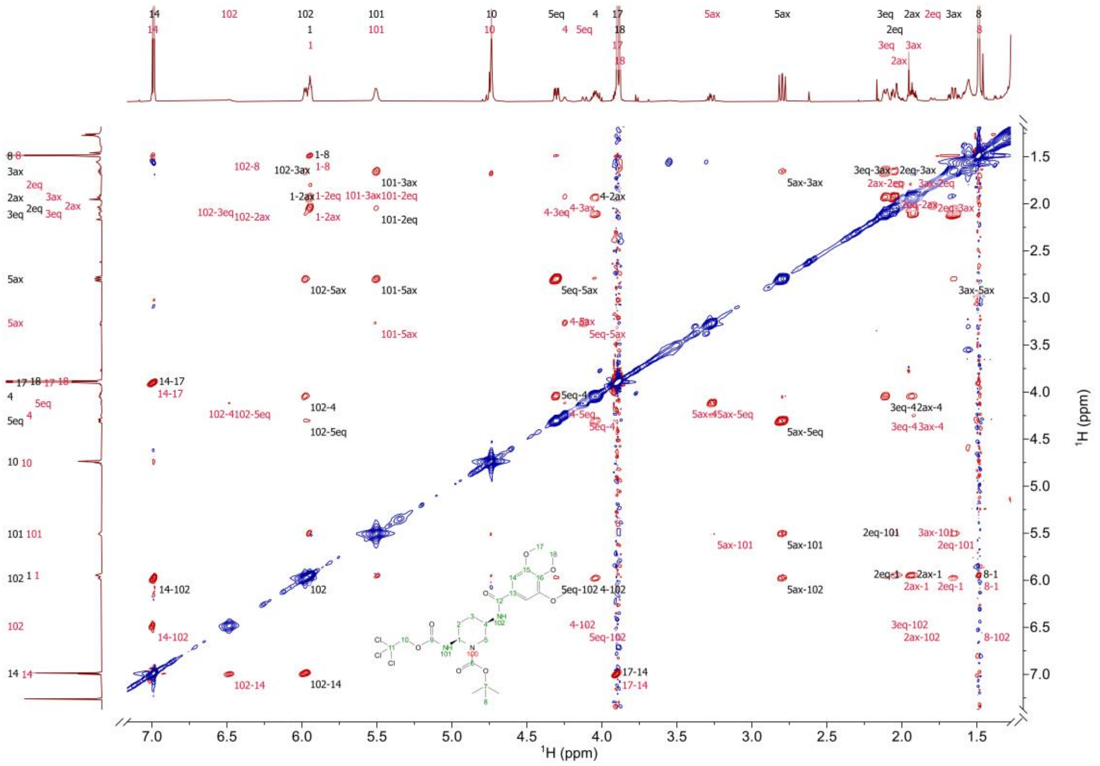

Compound **7x**: ^{1}H-^{15}N HMBC ($CDCl_3$, 323 K)

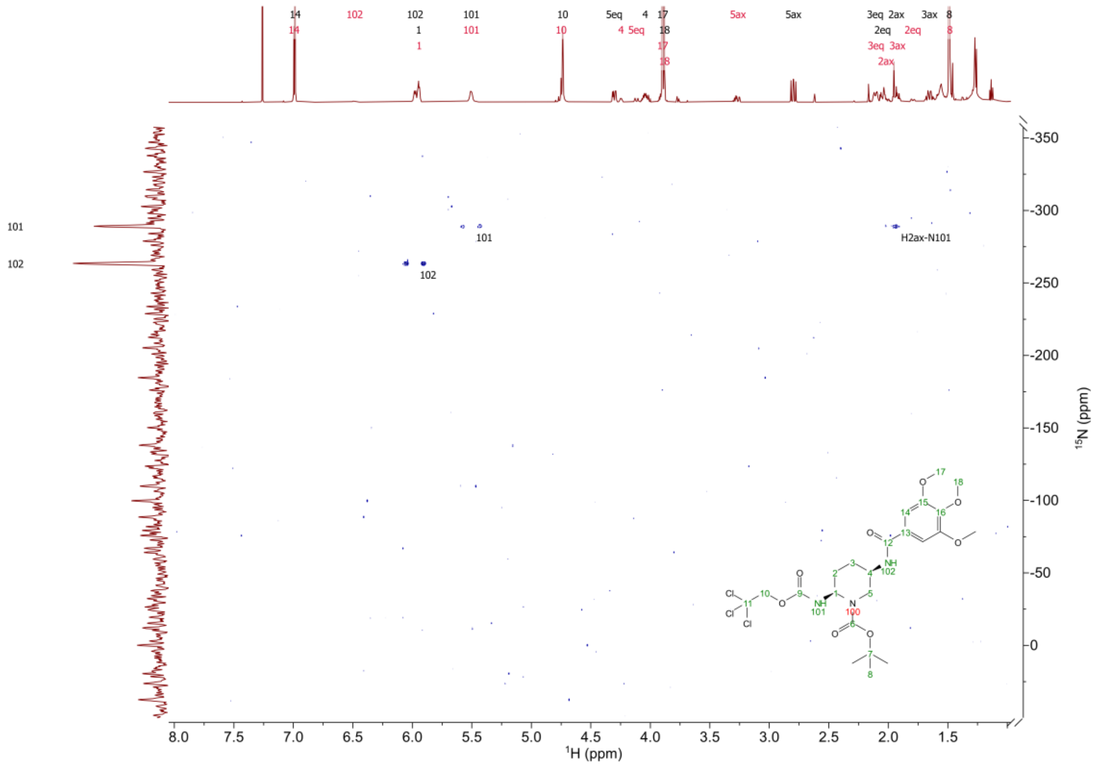

Compound **7x**: 1D selective TOCSY with excitation of H5ax

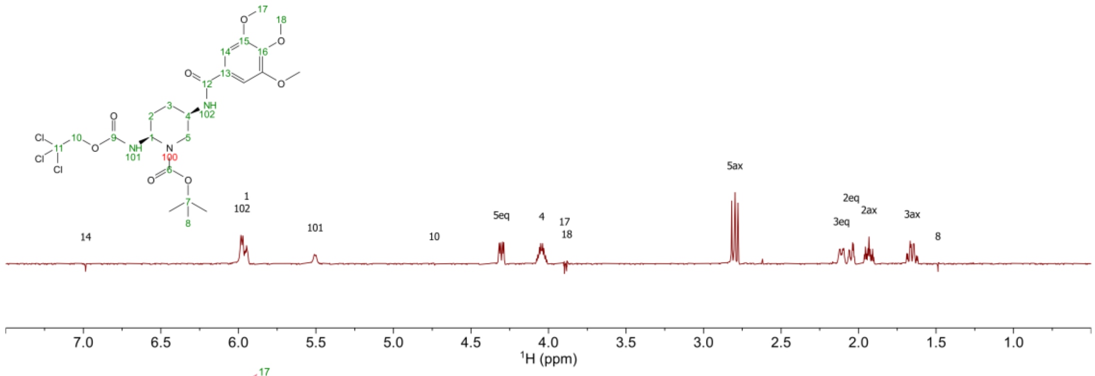

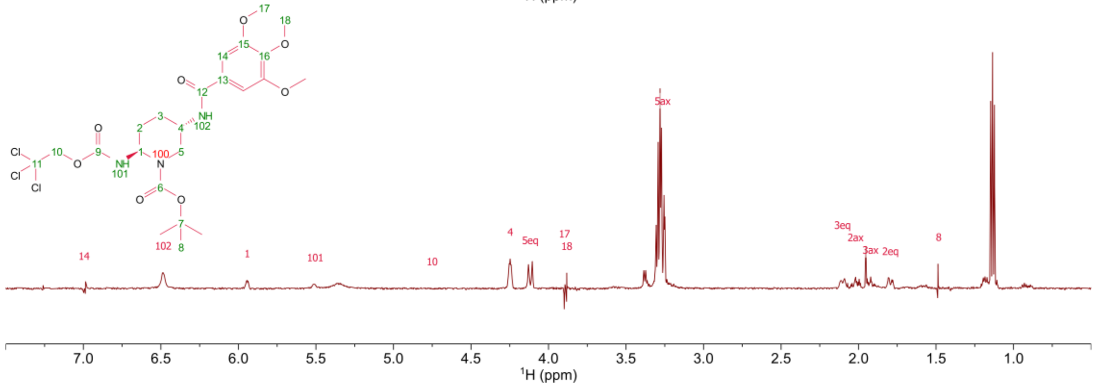

Compound **7y**: ^{1}H NMR (600 MHz, $CDCl_3$, 298 K)

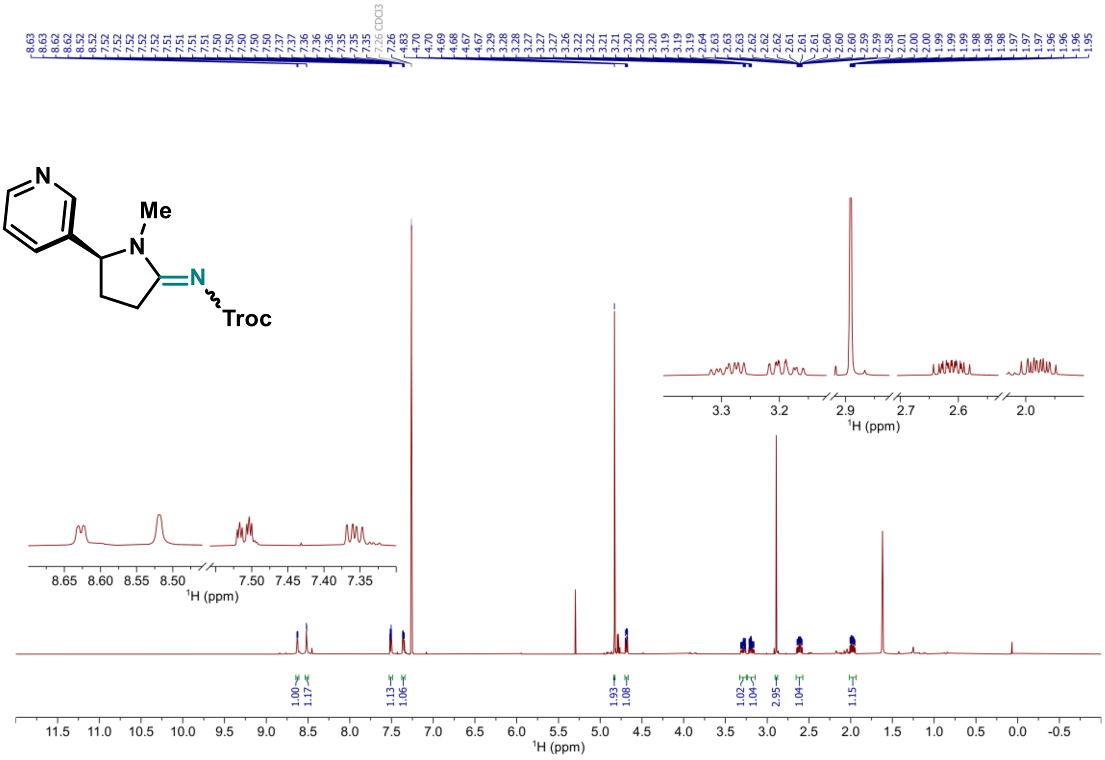

Compound **7y**: ^{13}C NMR (151 MHz, $CDCl_3$, 298 K)

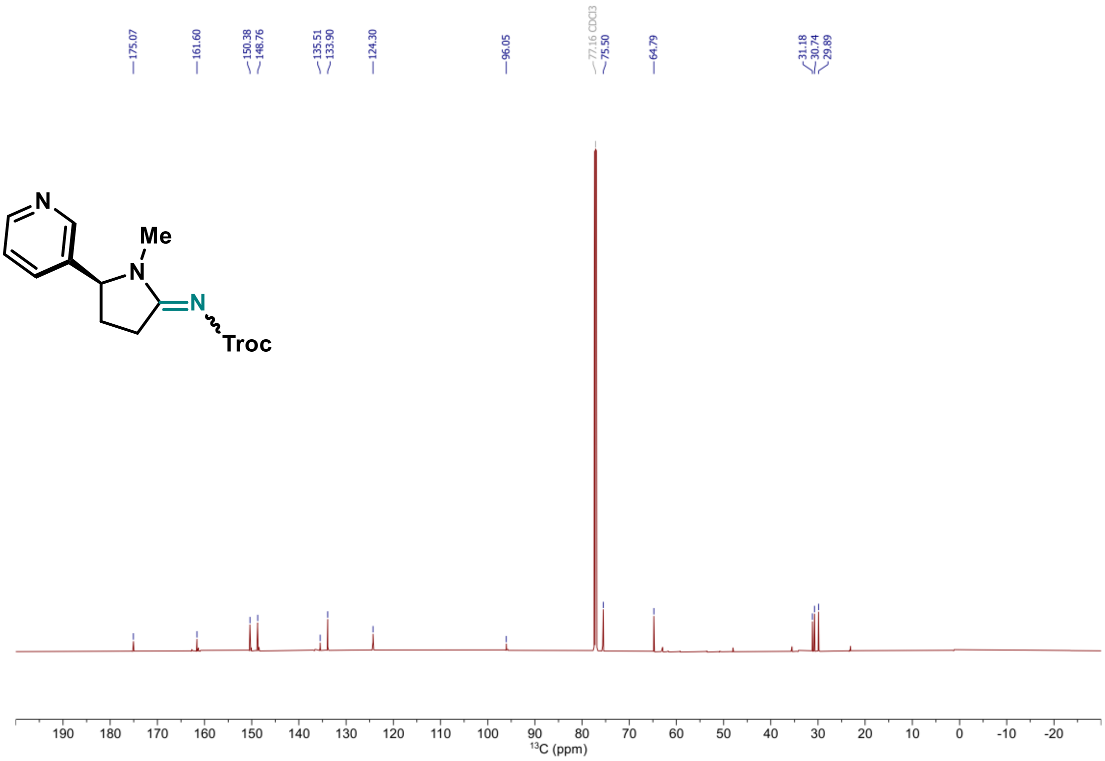

Compound **7z**: ^{1}H NMR (600 MHz, DMSO-d_6, 383 K)

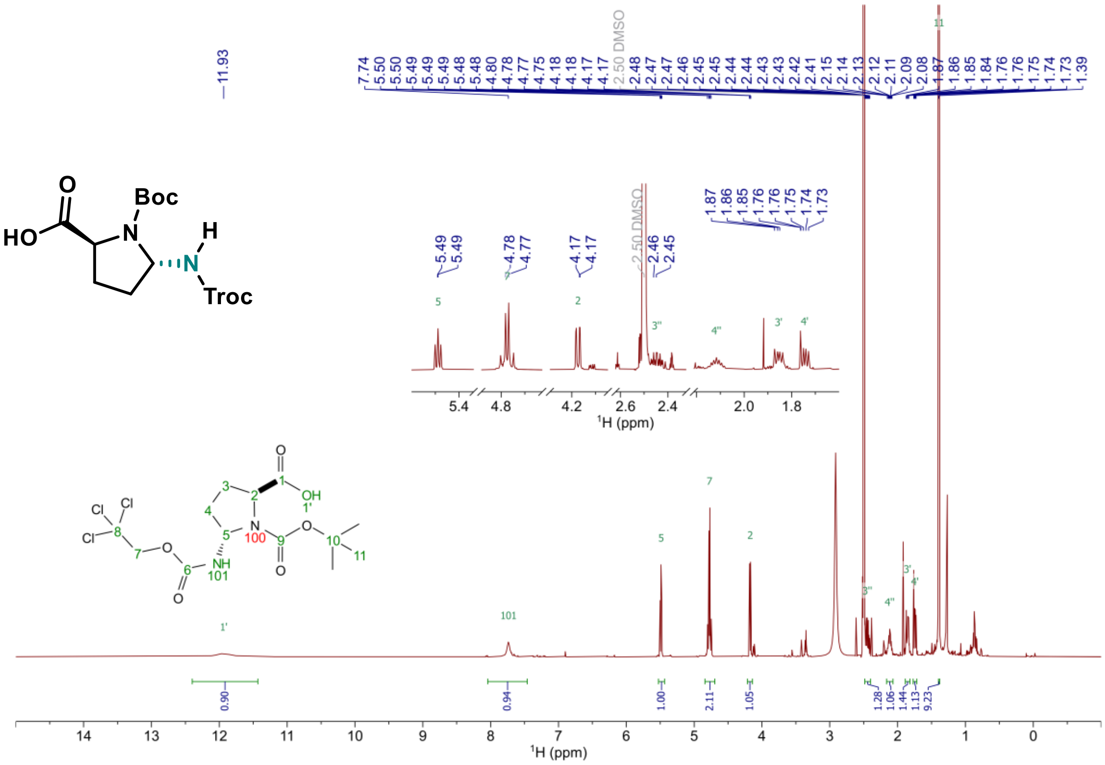

Compound **7z**: ^{13}C NMR (151 MHz, DMSO-d_6, 383 K)

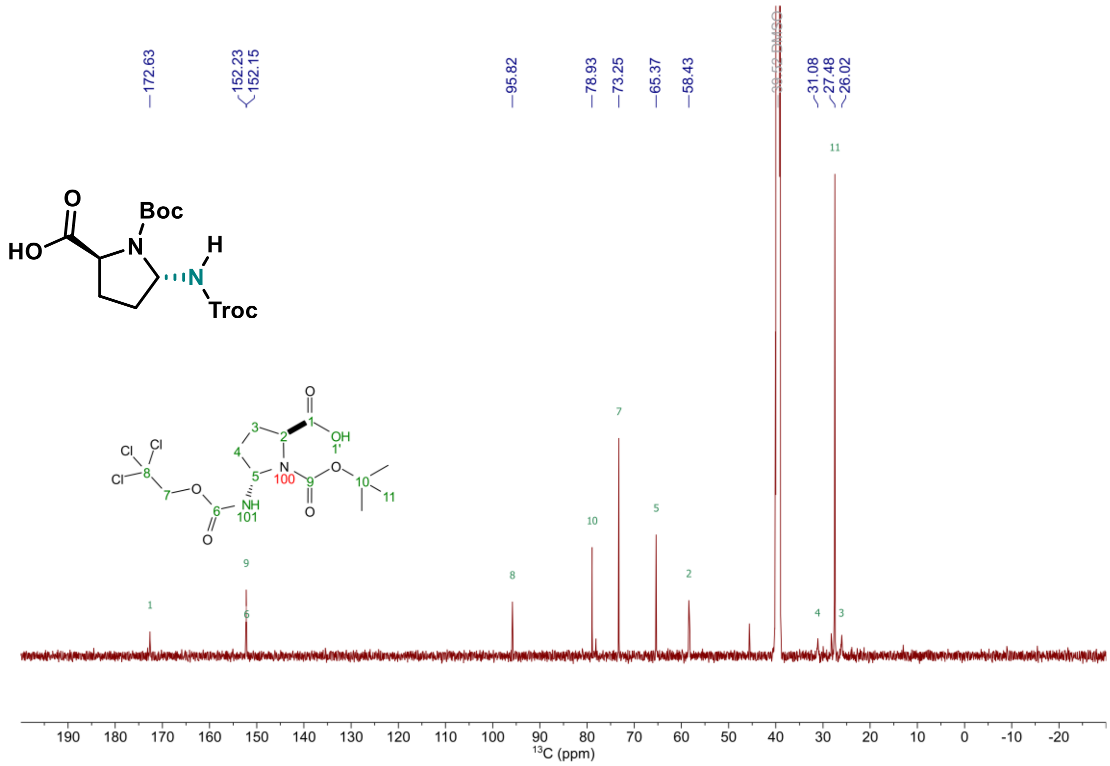

Compound **7z**: variable temperature ^{1}H NMR (600 MHz, DMSO-d_6)

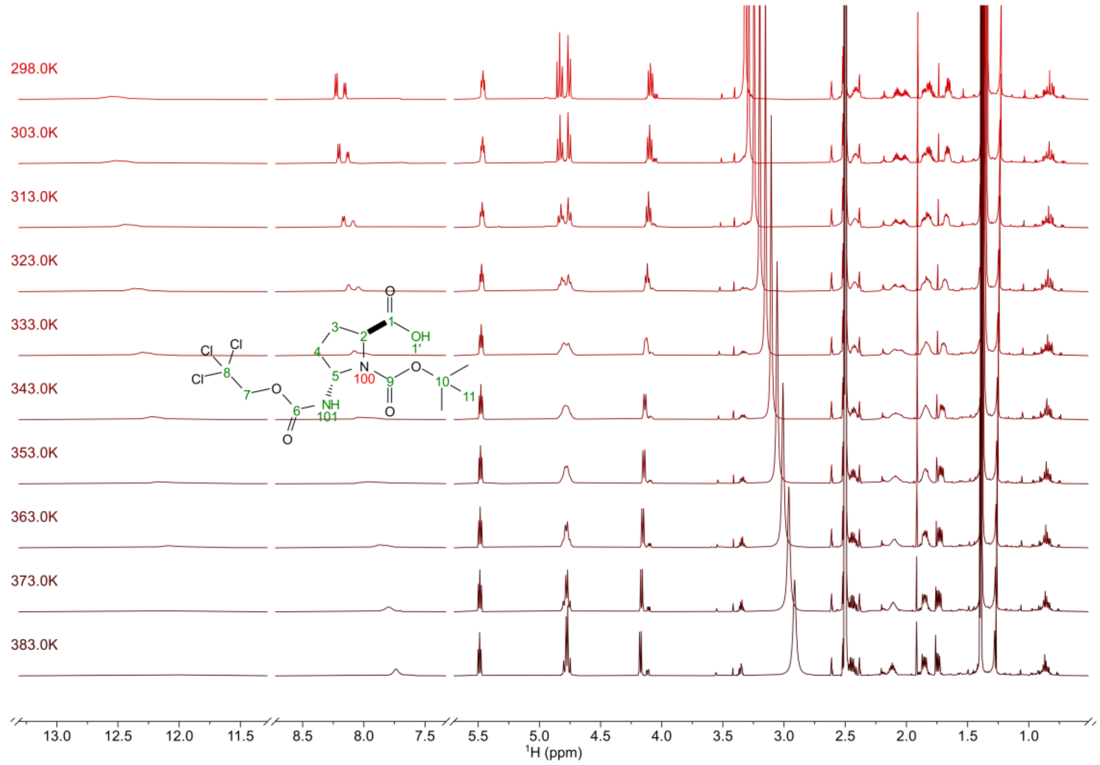

Compound **7z**: ^{1}H-^{13}C HSQC (DMSO-d_6, 383 K)

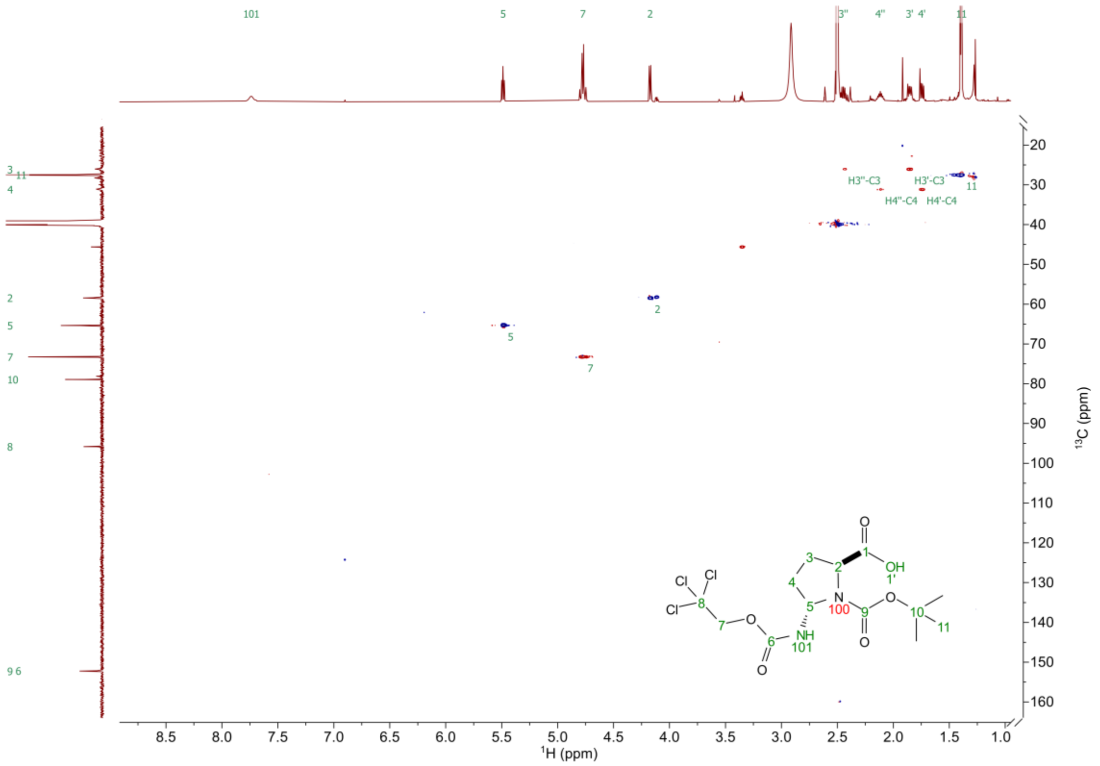

Compound **7z**: ^{1}H-^{1}H COSY (DMSO-d_6, 383 K)

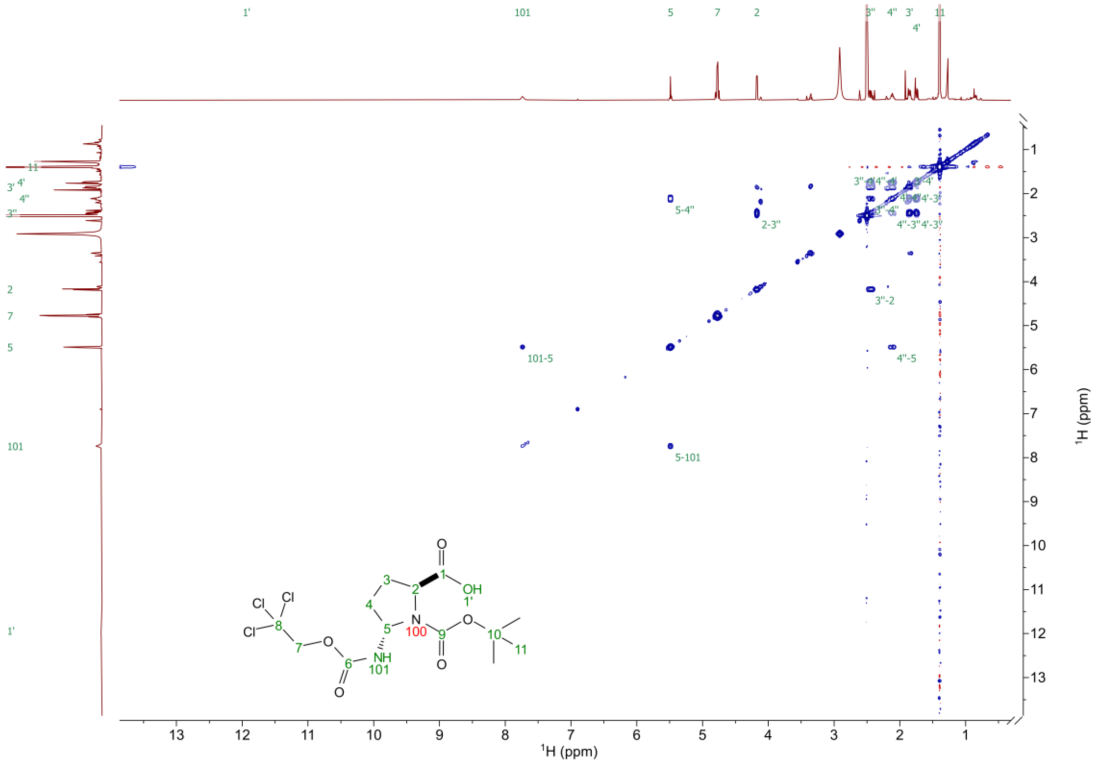

Compound **7z**: ^{1}H-^{1}H NOESY (DMSO-d_6, 383 K)

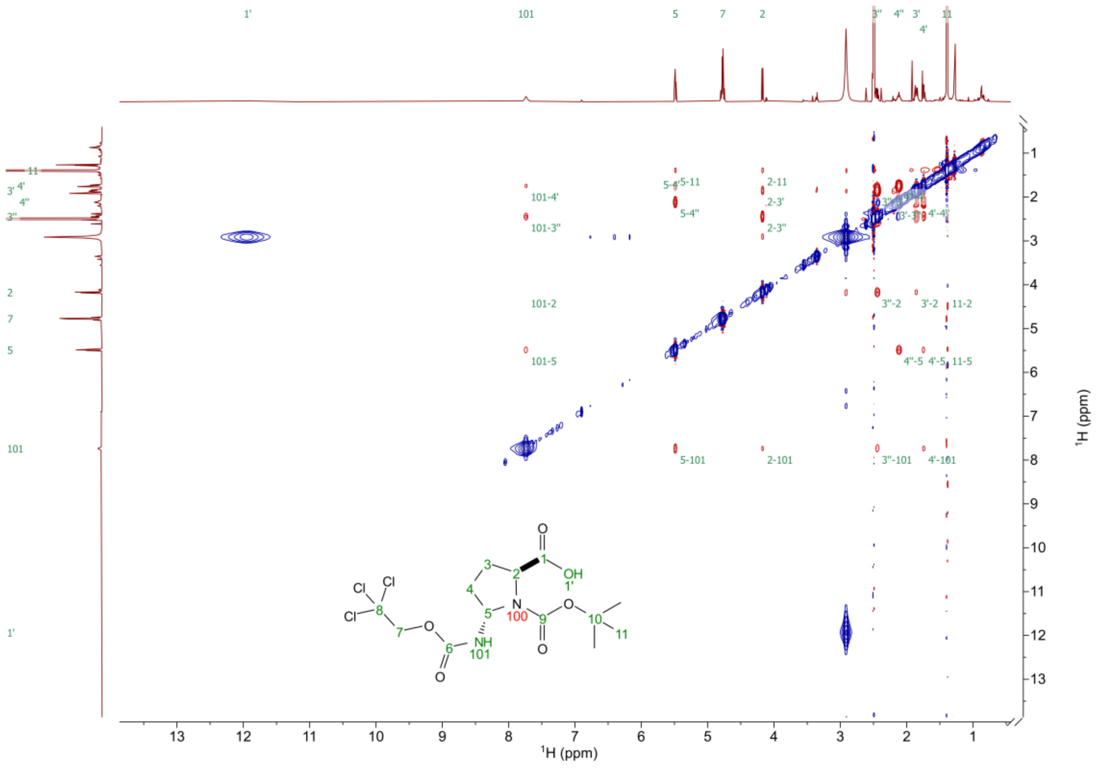

Compound **9a**: ^{1}H NMR (600 MHz, $CDCl_3$, 298 K)

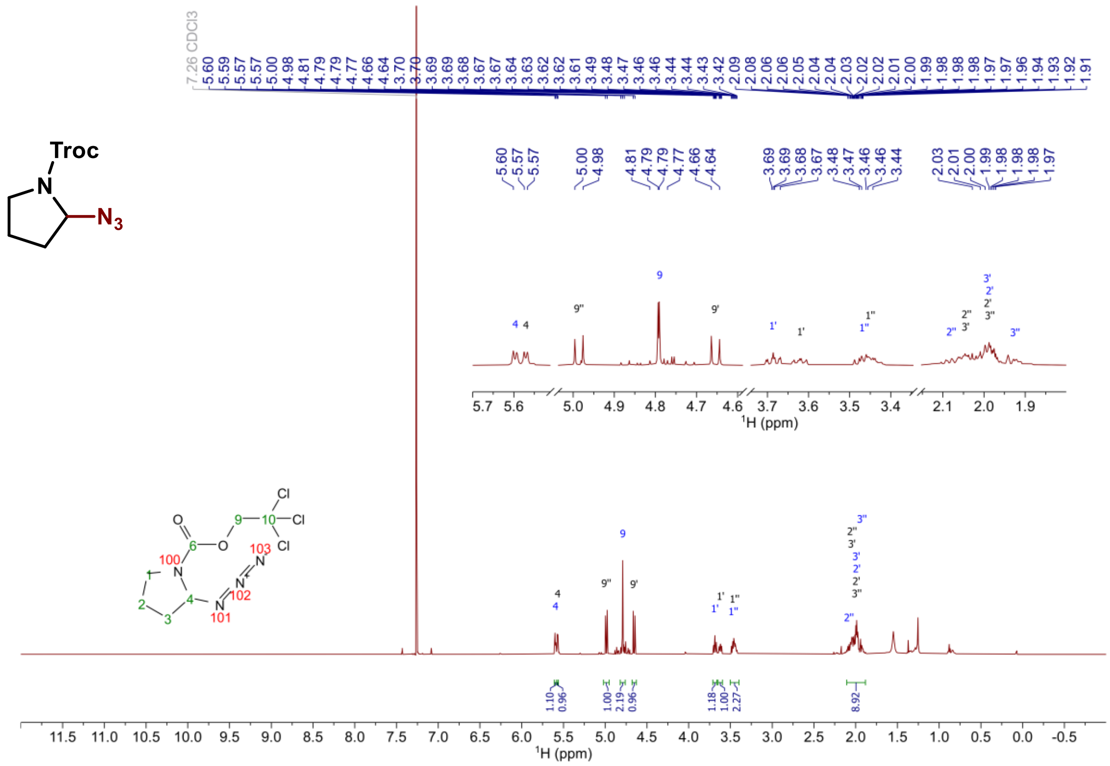

Compound **9a**: ^{13}C NMR (151 MHz, $CDCl_3$, 298 K)

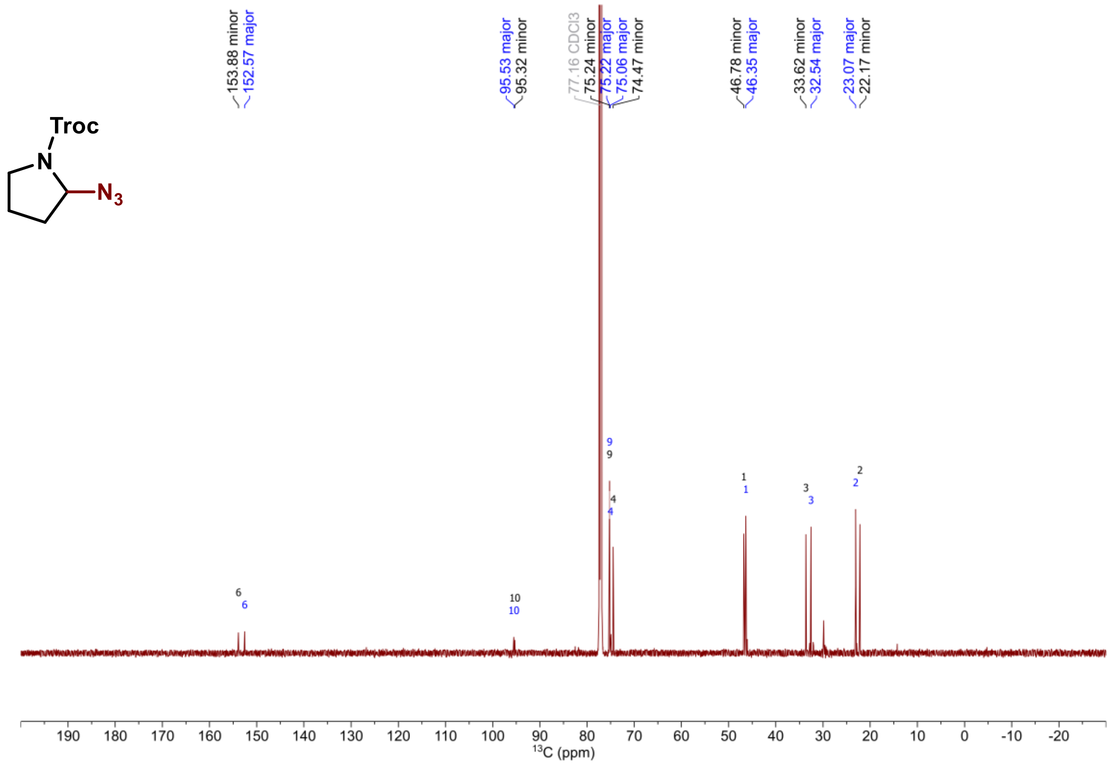

Compound **9a**: ^{1}H-^{13}C HSQC ($CDCl_3$, 298 K)

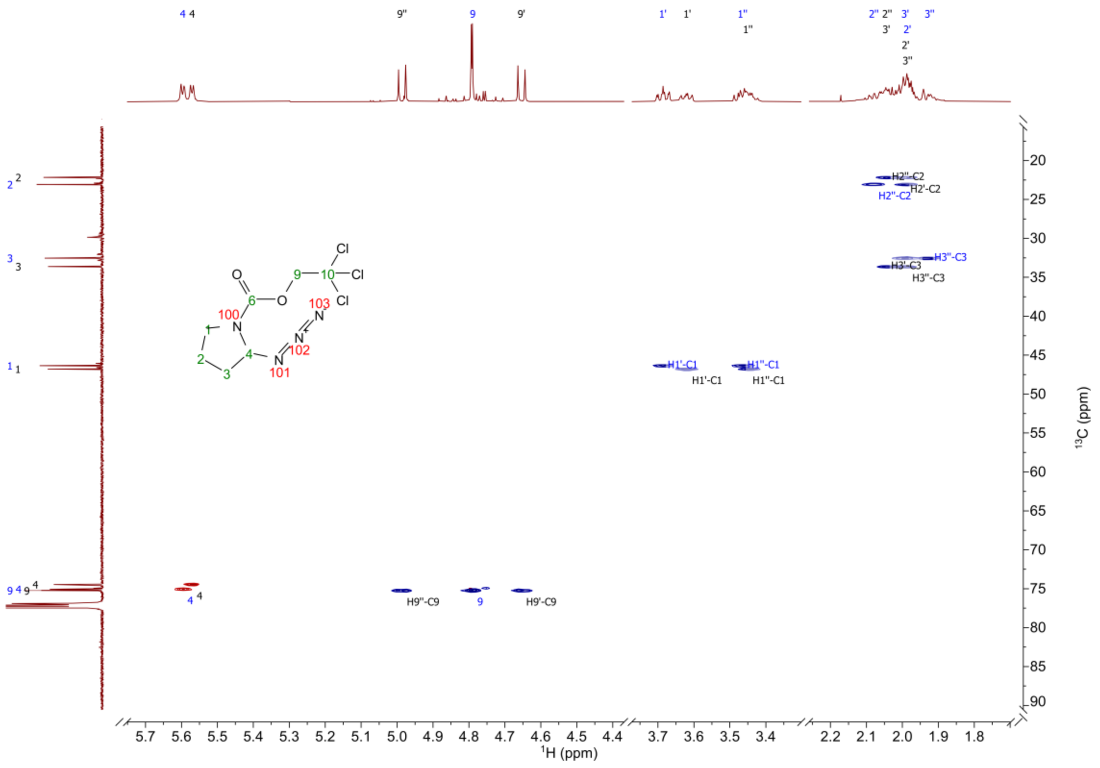

Compound **9a**: ^{1}H-^{13}C HMBC ($CDCl_3$, 298 K)

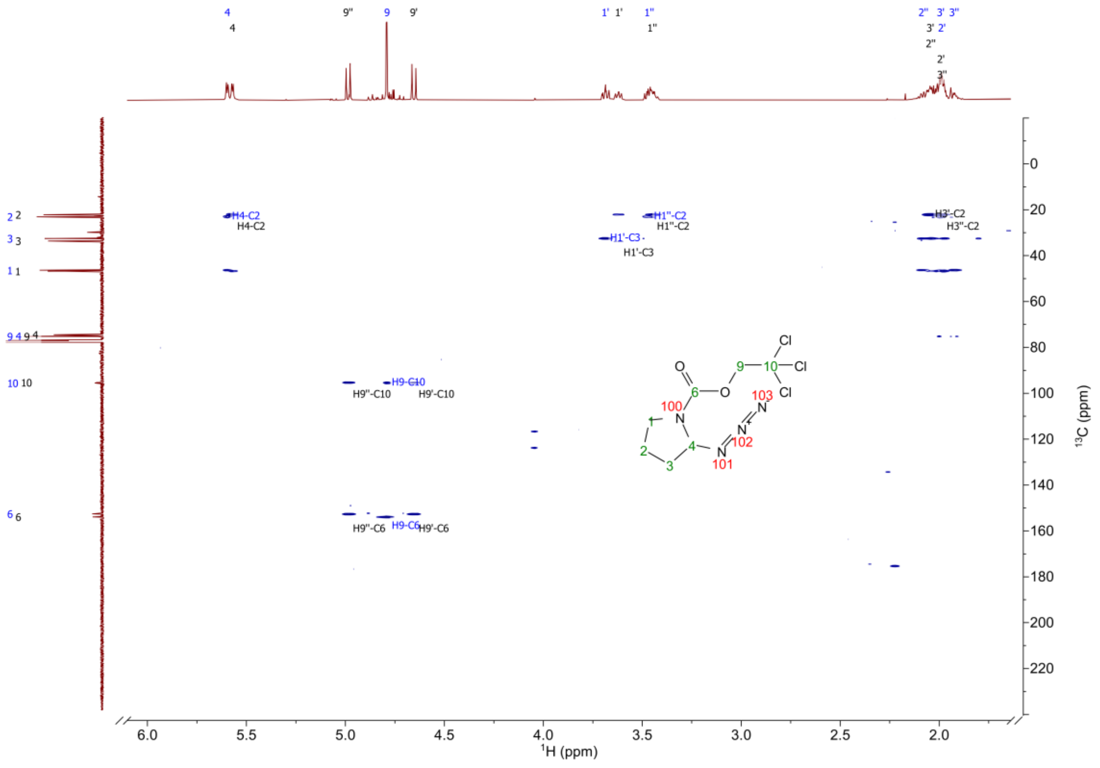

Compound **9a**: ^1H-^1H COSY ($CDCl_3$, 298 K)

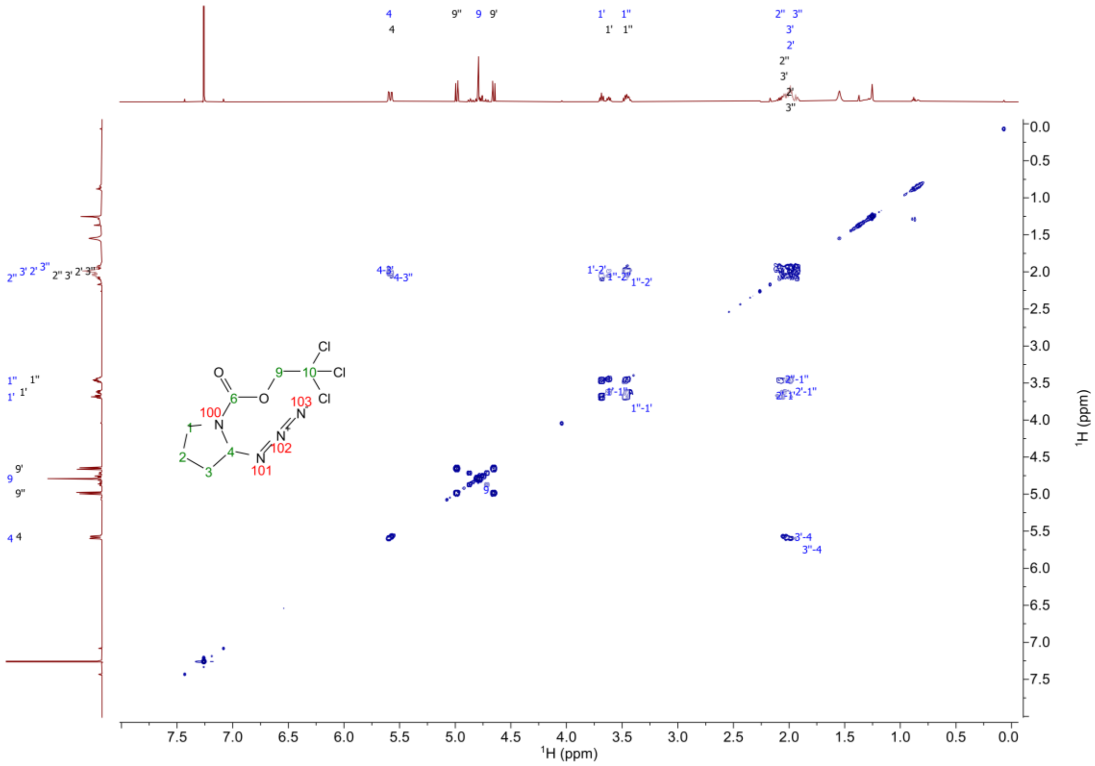

Compound **9a**: ^1H-^1H NOESY ($CDCl_3$, 298 K)

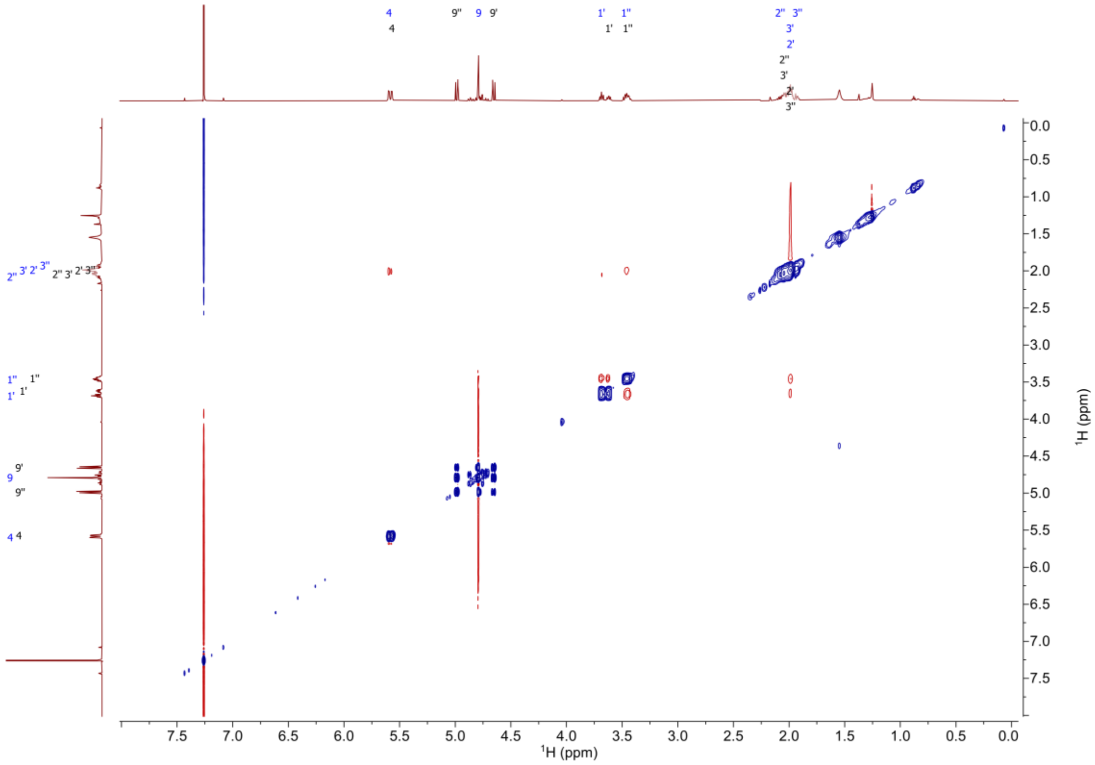

Compound **9b**: ^{1}H NMR (600 MHz, $CDCl_3$, 298 K)

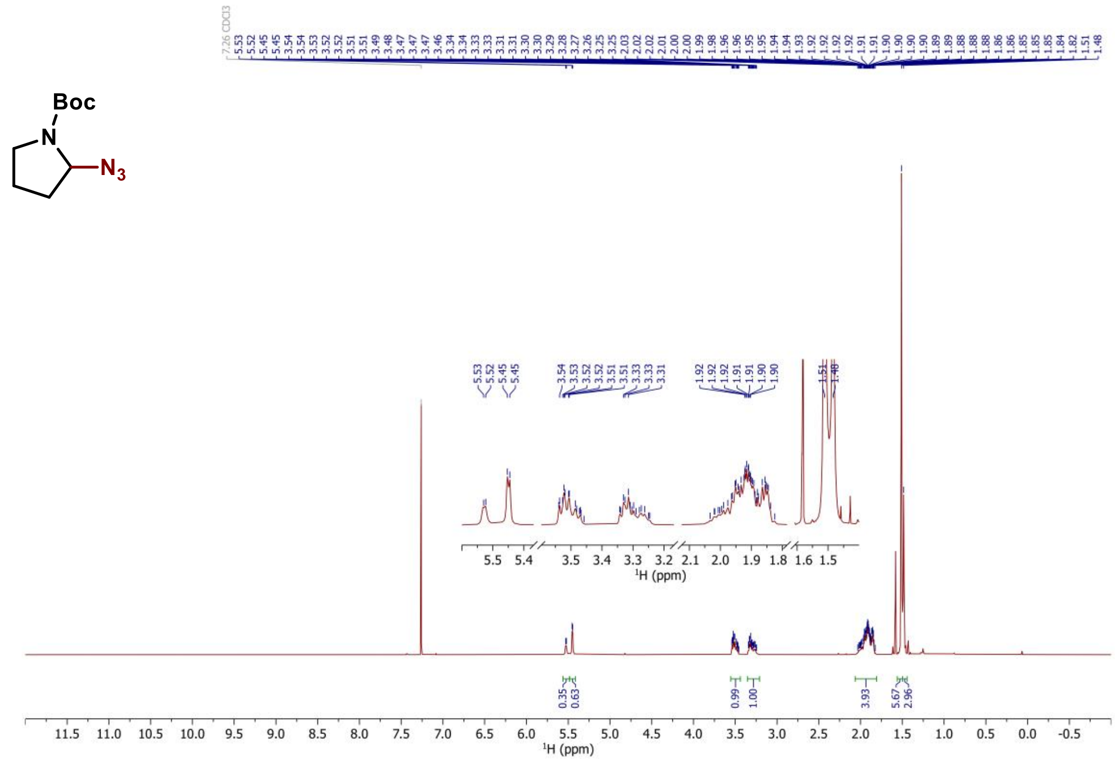

Compound **9b**: ^{13}C NMR (151 MHz, $CDCl_3$, 298 K)

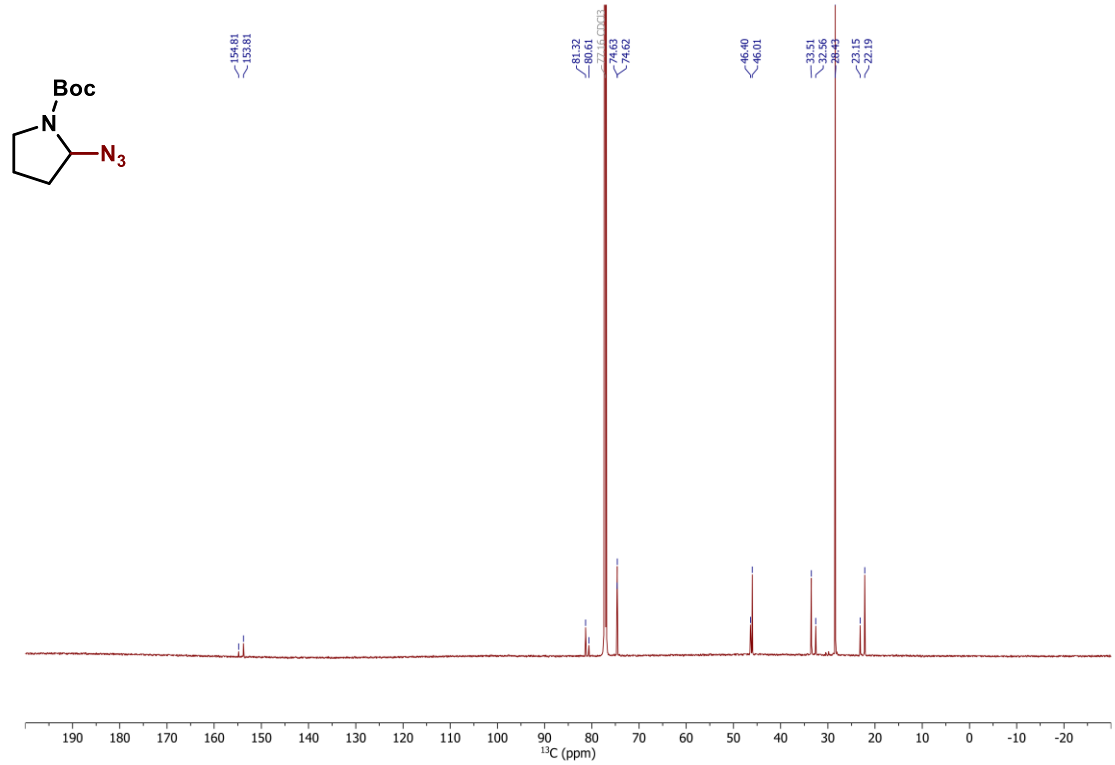

Compound **9c**: ^{1}H NMR (600 MHz, $CDCl_3$, 253 K)

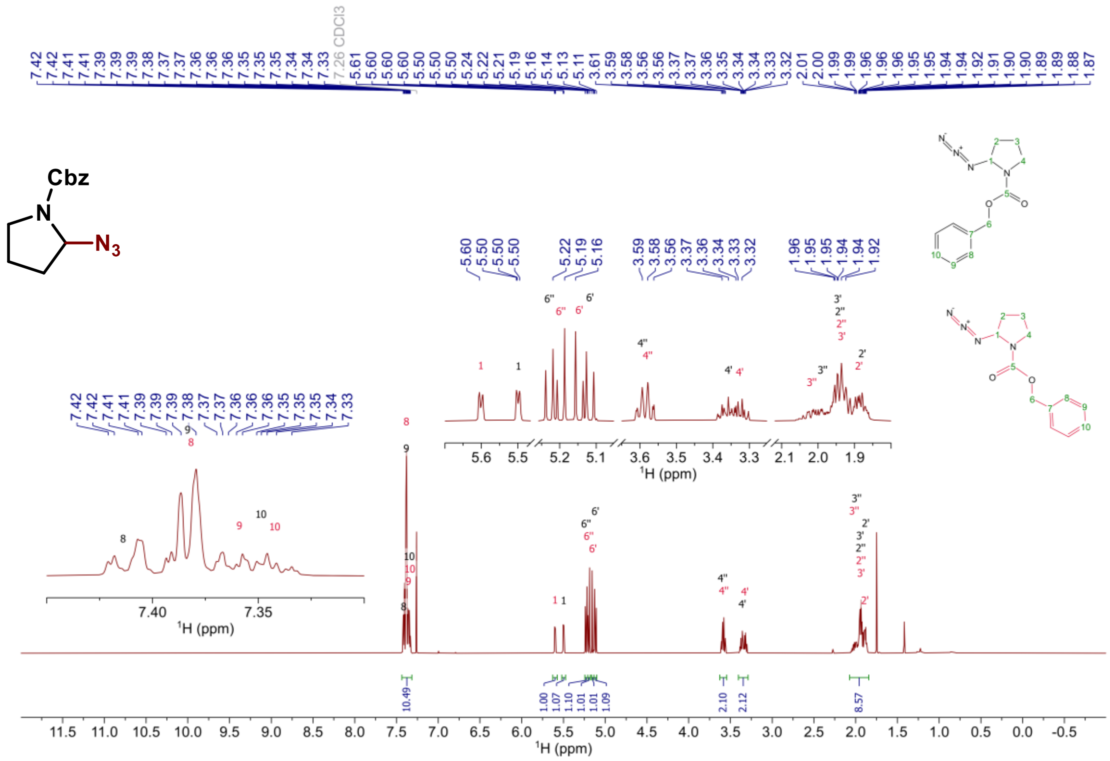

Compound **9c**: ^{13}C NMR (151 MHz, $CDCl_3$, 253 K)

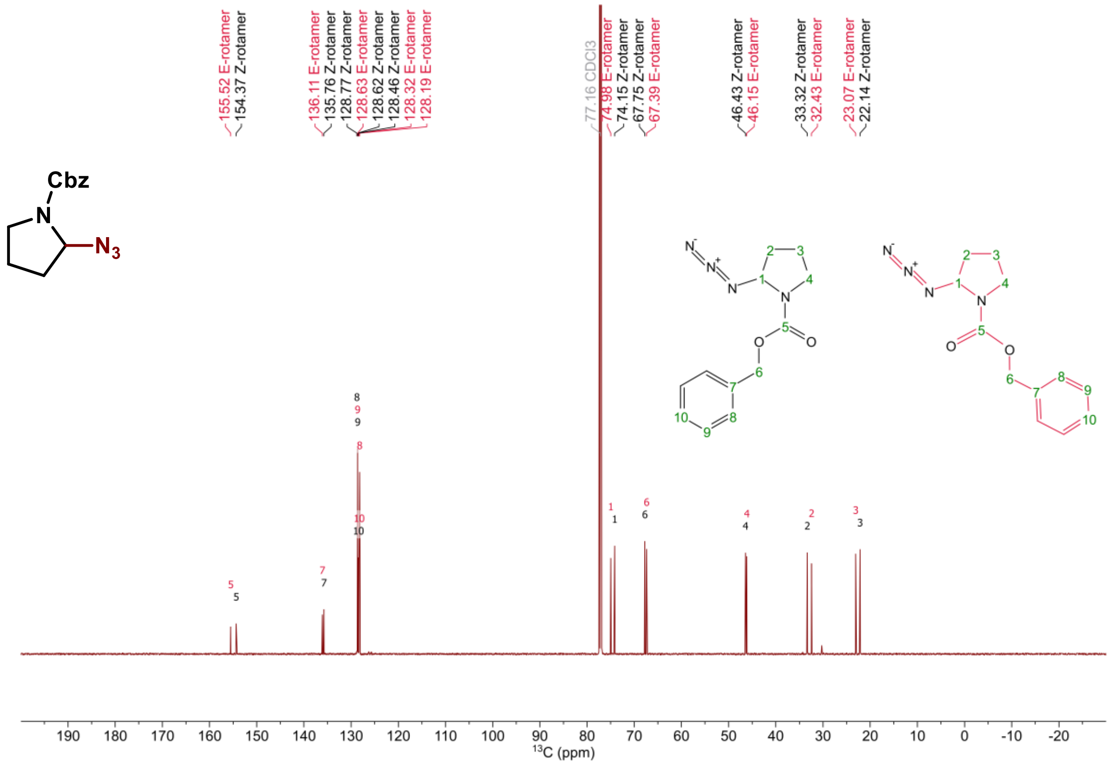

Compound **9c**: ^{1}H-^{13}C HSQC ($CDCl_3$, 253 K)

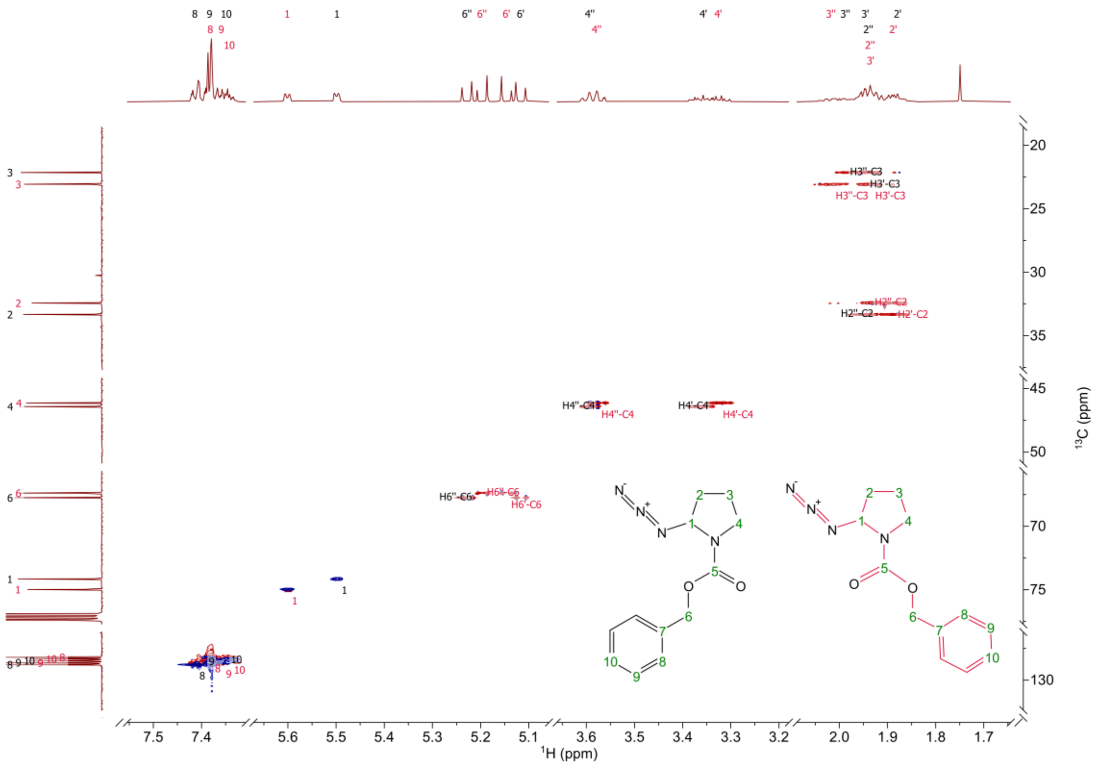

Compound **9c**: ^{1}H-^{13}C HMBC ($CDCl_3$, 253 K)

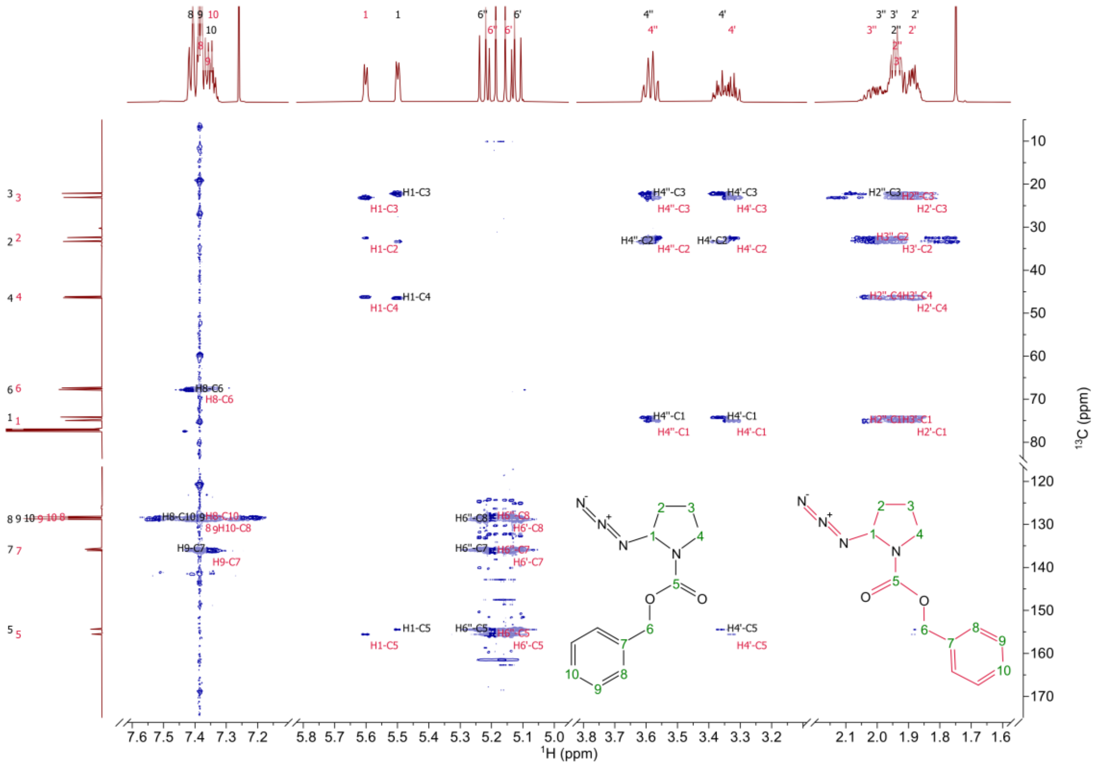

Compound **9c**: ^1H-^1H COSY ($CDCl_3$, 253 K)

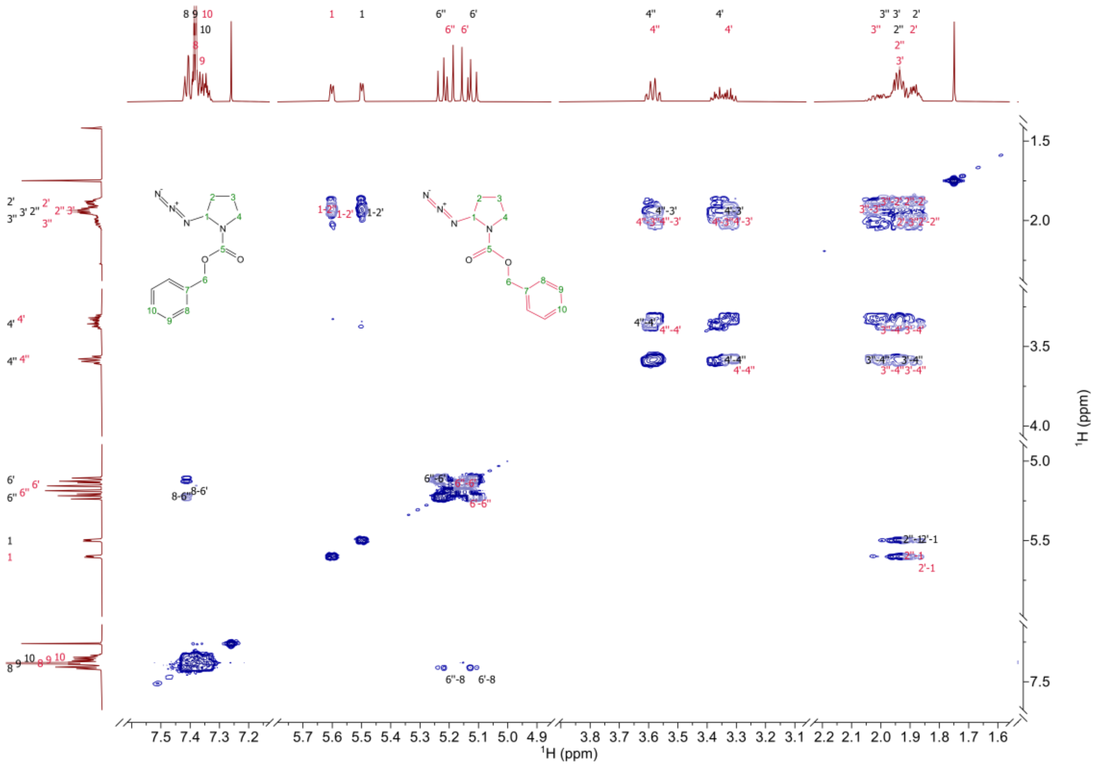

Compound **9c**: ^1H-^1H NOESY ($CDCl_3$, 253 K)

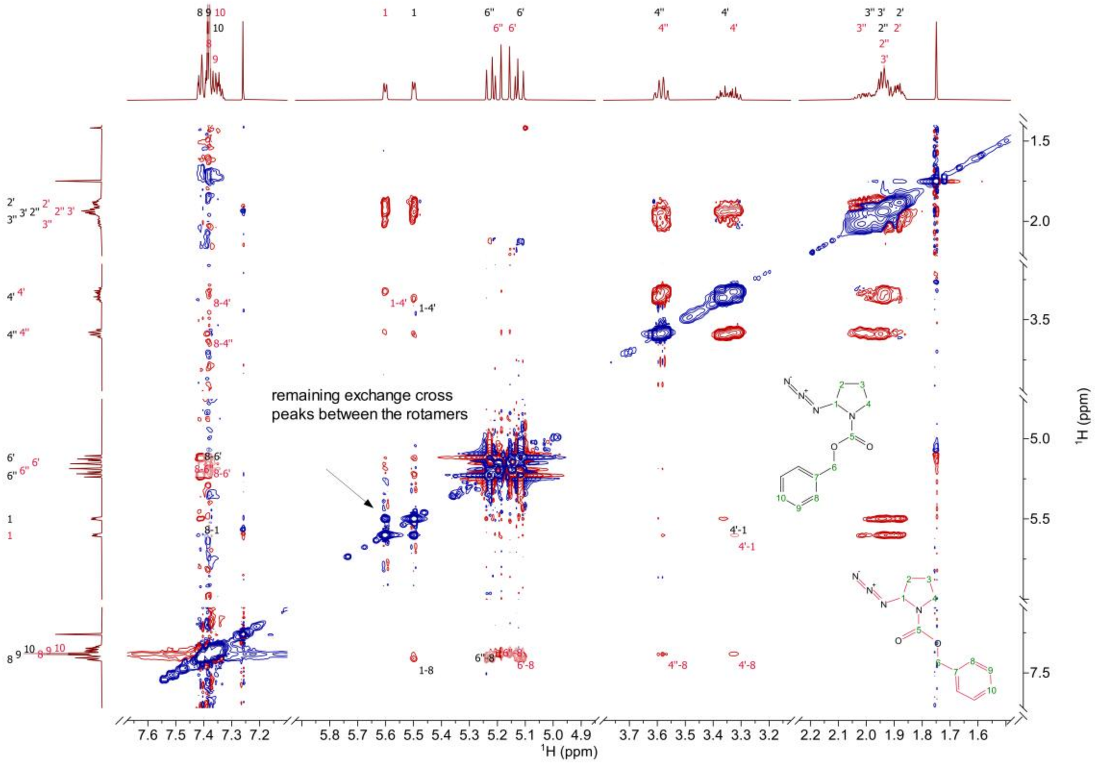

Compound **9c**: ^{1}H-^{15}N HMBC ($CDCl_3$, 253 K)

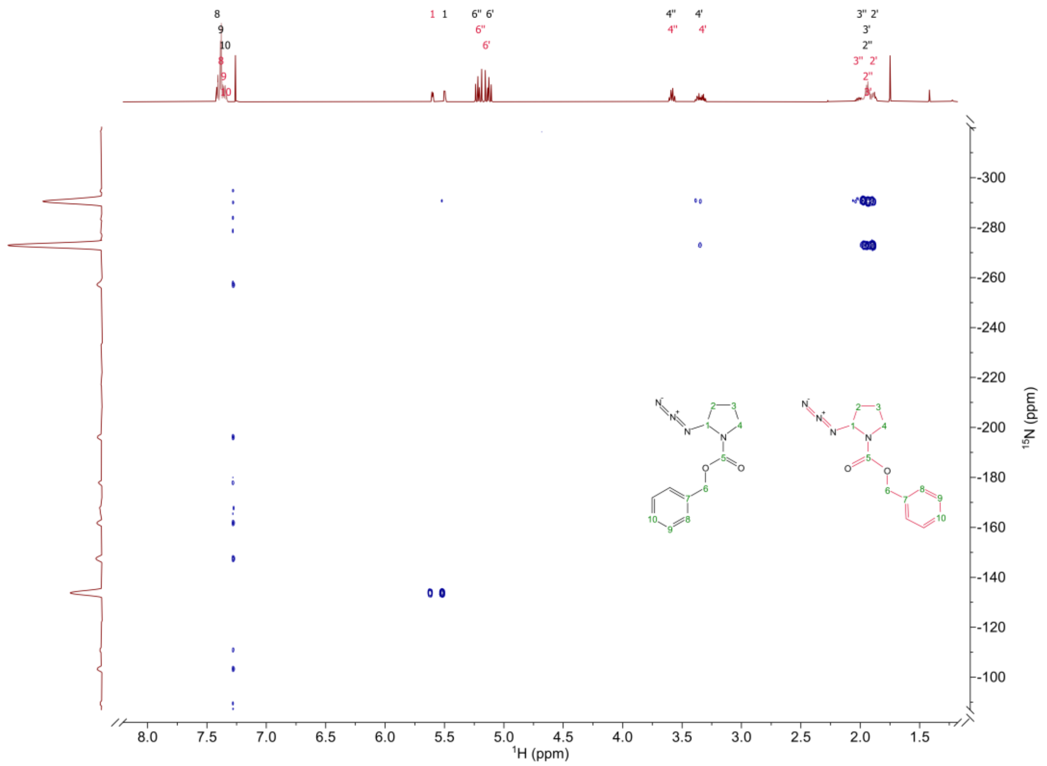

Compound **9c**: 1D selective TOCSY with excitation of H1

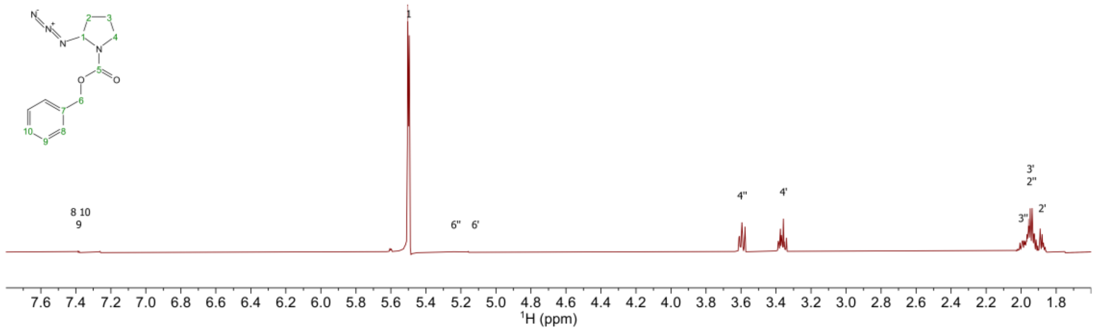

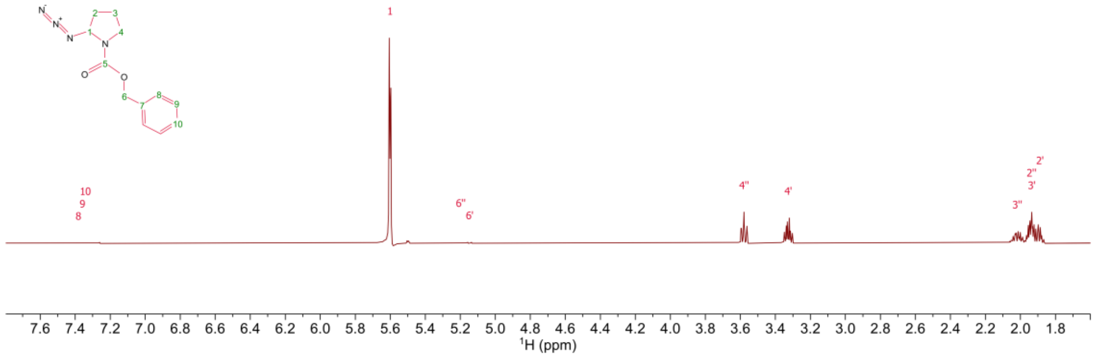

Compound **9d**, trans diastereomer: ^{1}H NMR (600 MHz, $CDCl_3$, 253 K)

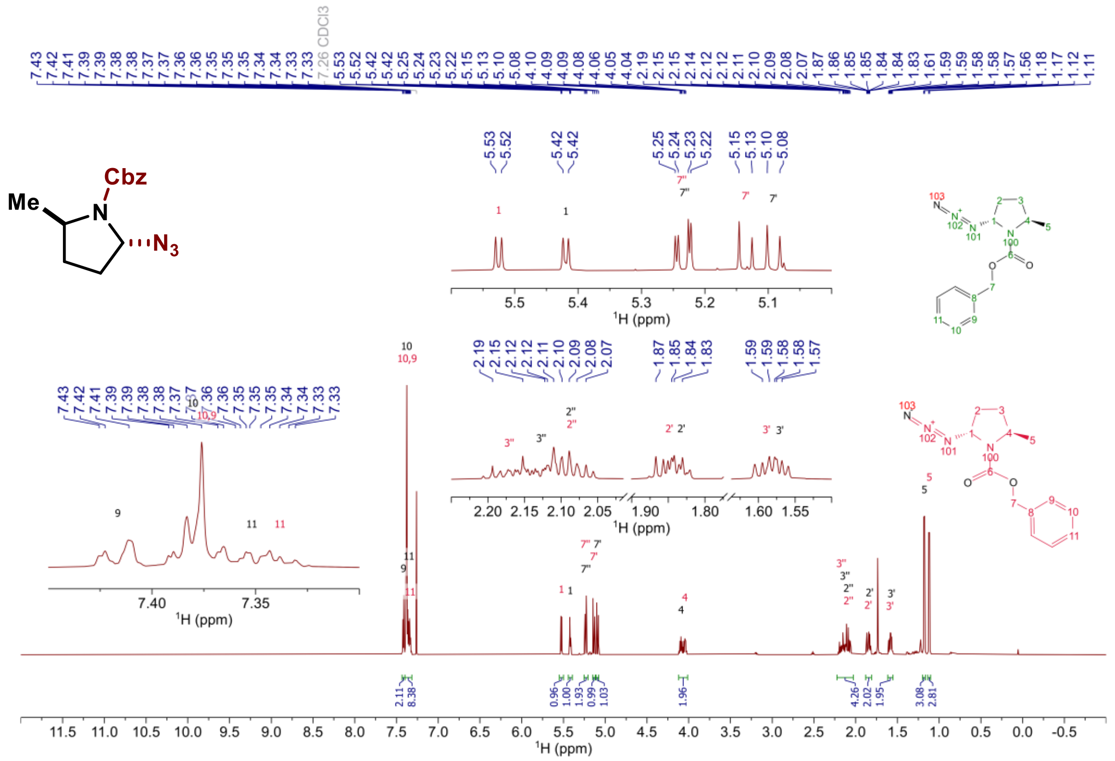

Compound **9d**, trans diastereomer: ^{13}C NMR (151 MHz, $CDCl_3$, 253 K)

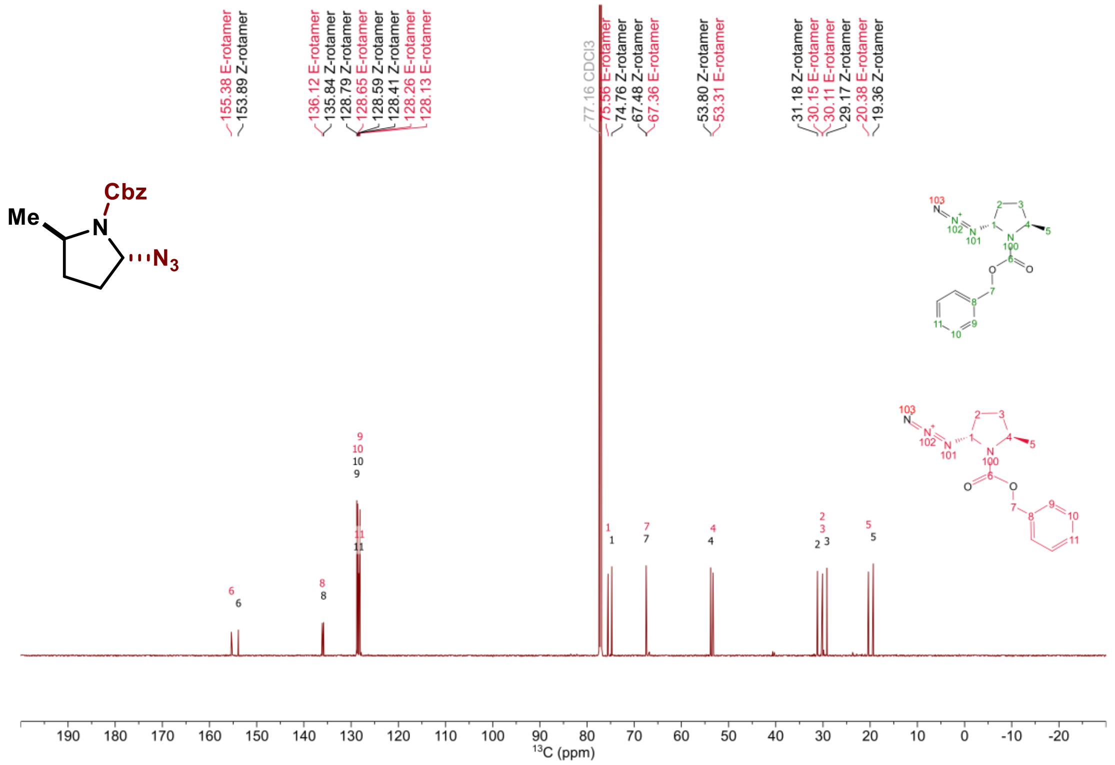

Compound **9d**, trans diastereomer: variable temperature ^{1}H NMR (600 MHz, $CDCl_3$)

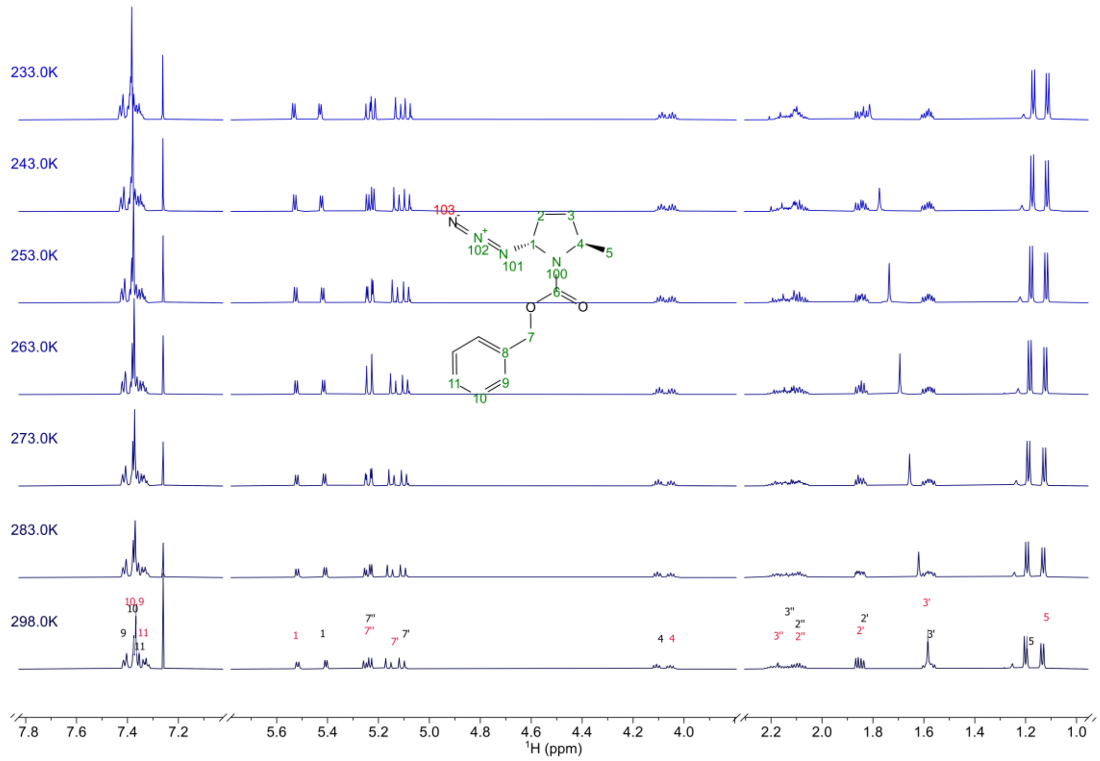

Compound **9d**, trans diastereomer: ^{1}H-^{13}C HSQC ($CDCl_3$, 253 K)

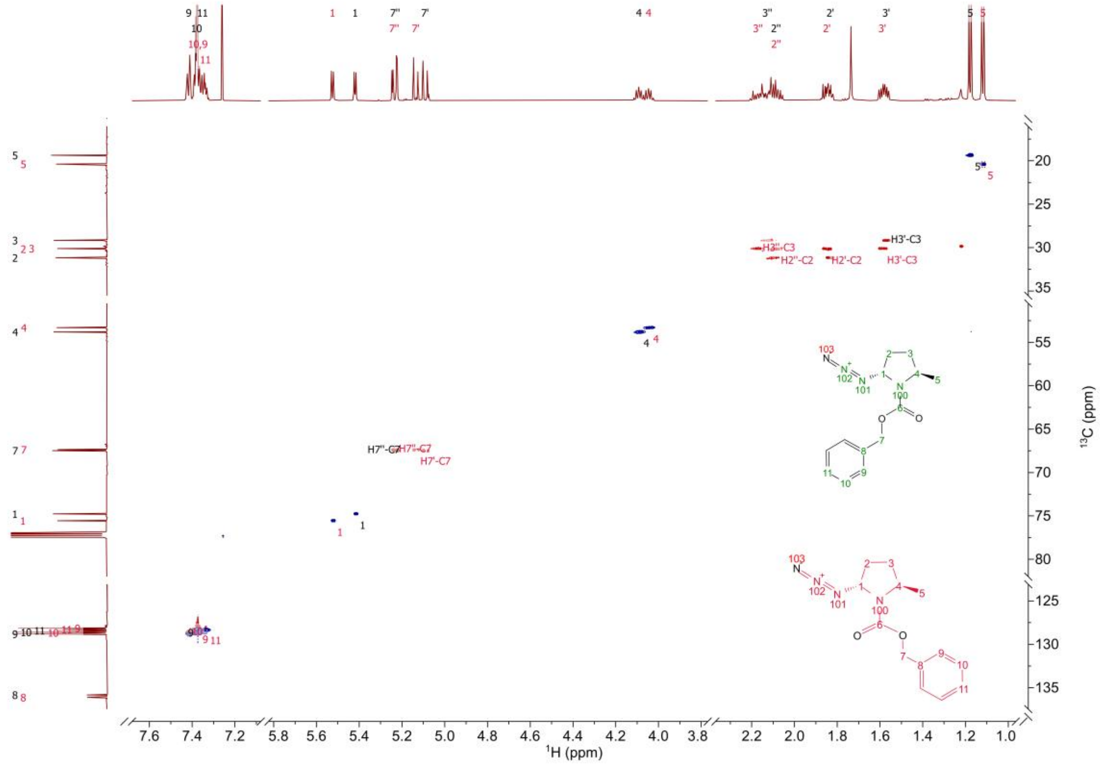

Compound **9d**, trans diastereomer: ^{1}H-^{13}C HMBC ($CDCl_3$, 253 K)

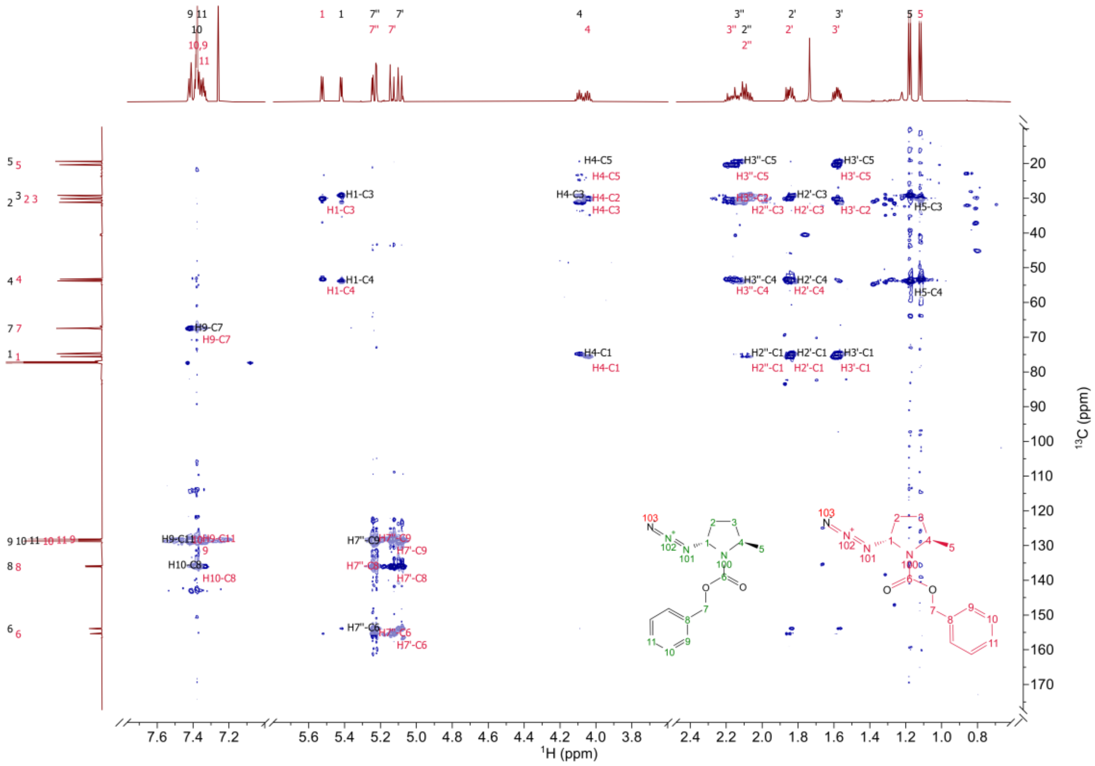

Compound **9d**, trans diastereomer: ^{1}H-^{1}H COSY ($CDCl_3$, 253 K)

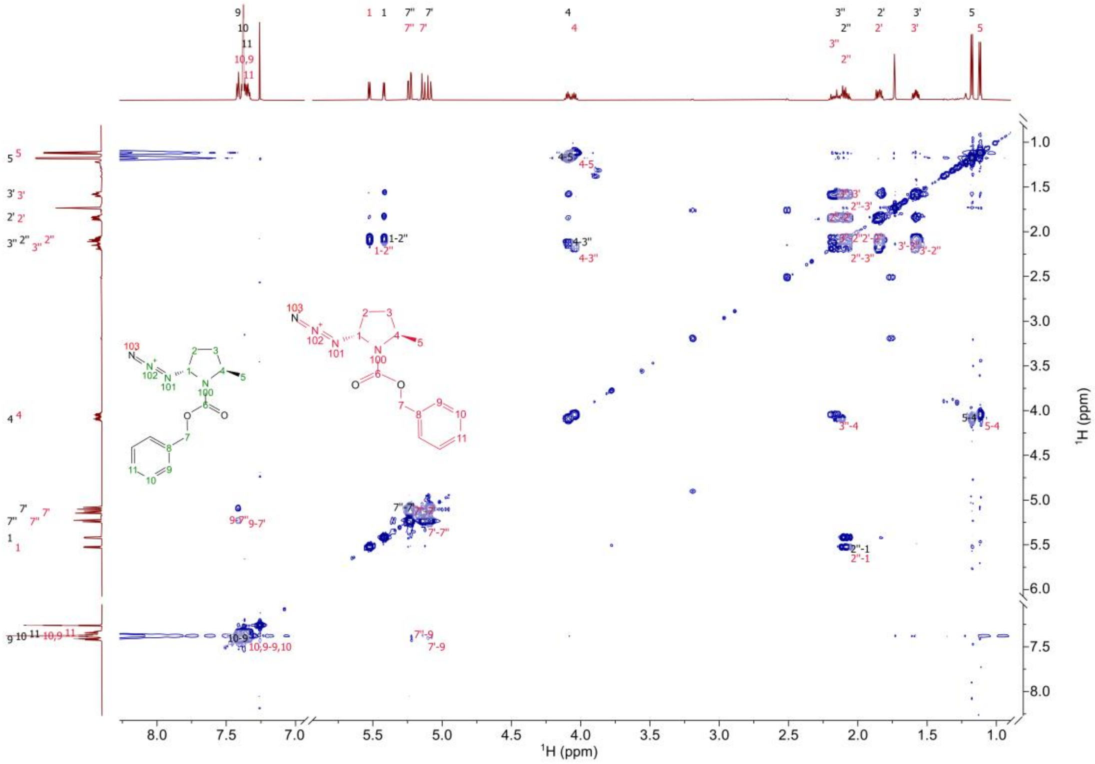

Compound **9d**, trans diastereomer: ^{1}H-^{1}H NOESY ($CDCl_3$, 253 K)

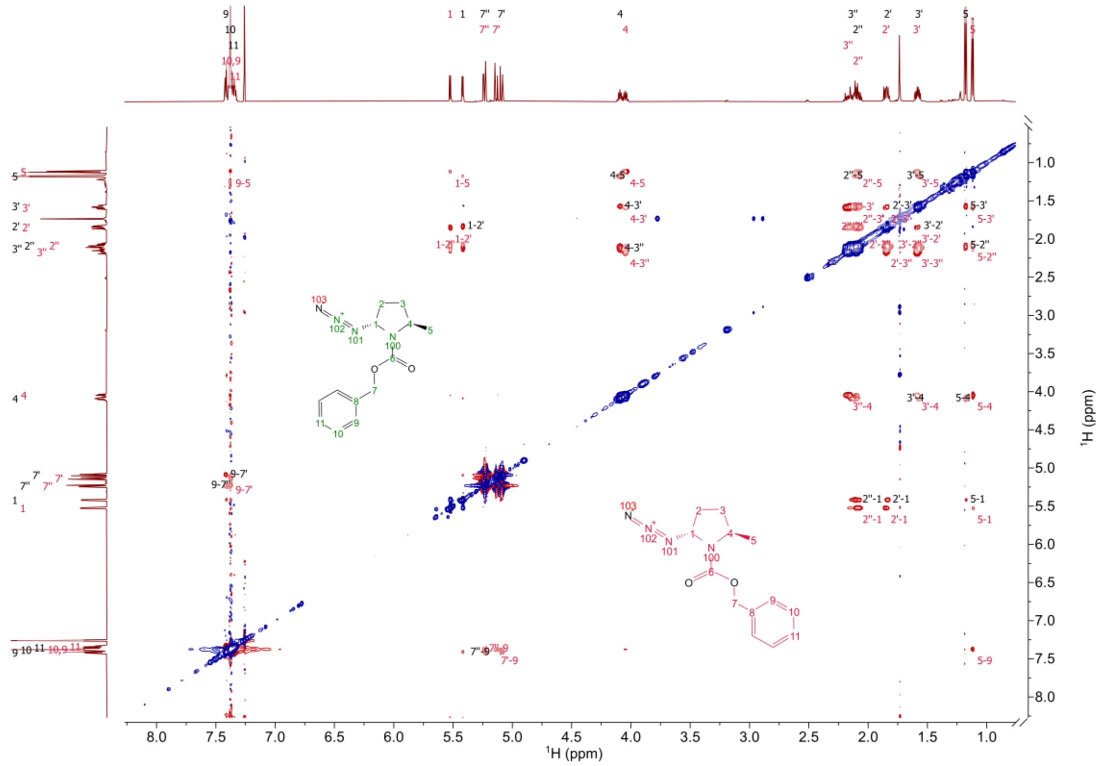

Compound **9d**, trans diastereomer: ^{1}H-^{15}N HMBC ($CDCl_3$, 253 K)

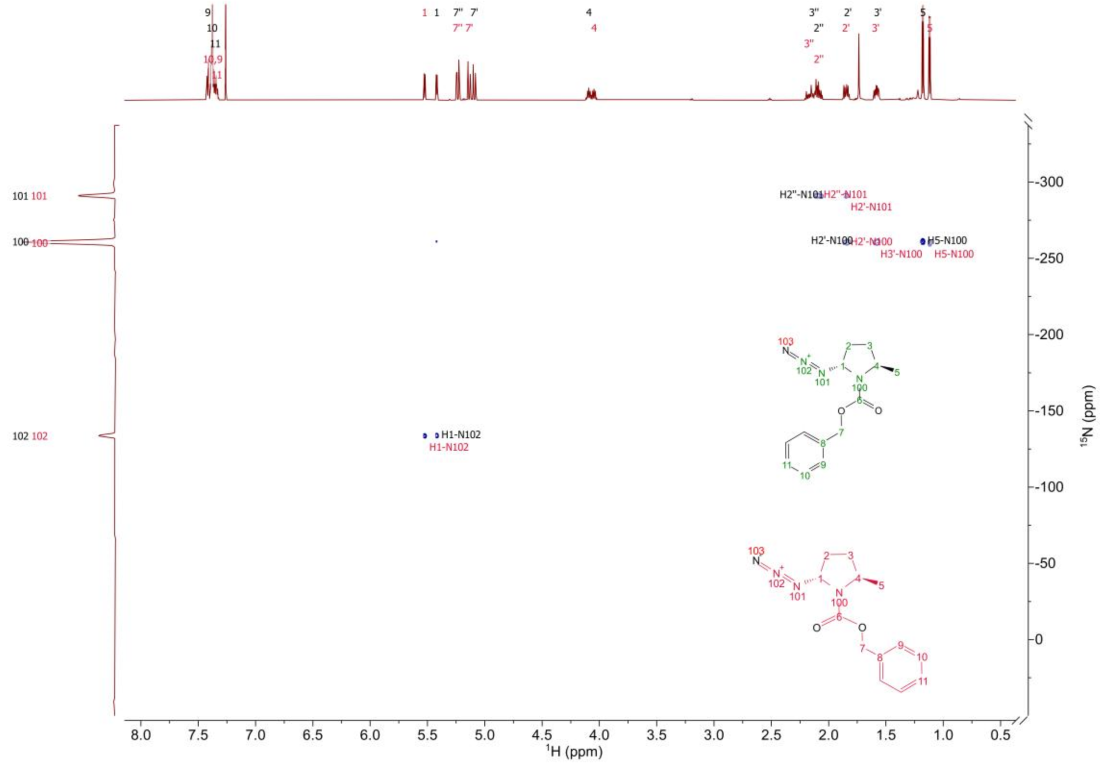

Compound **9d**, trans diastereomer: 1D selective TOCSY with excitation of H1

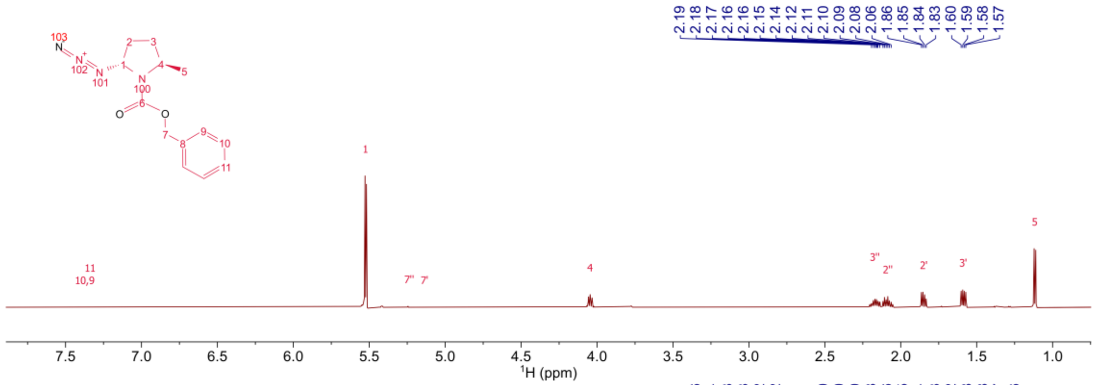

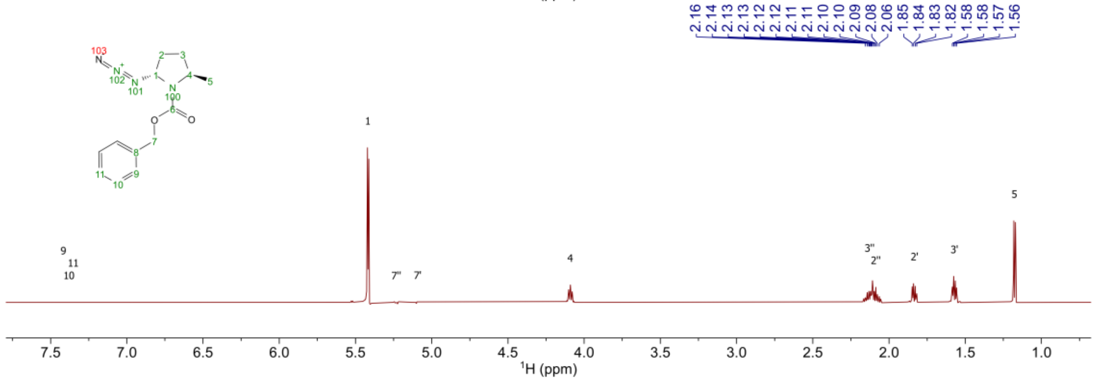

Compound **9d**, cis diastereomer: ^{1}H NMR (600 MHz, $CDCl_3$, 253 K)

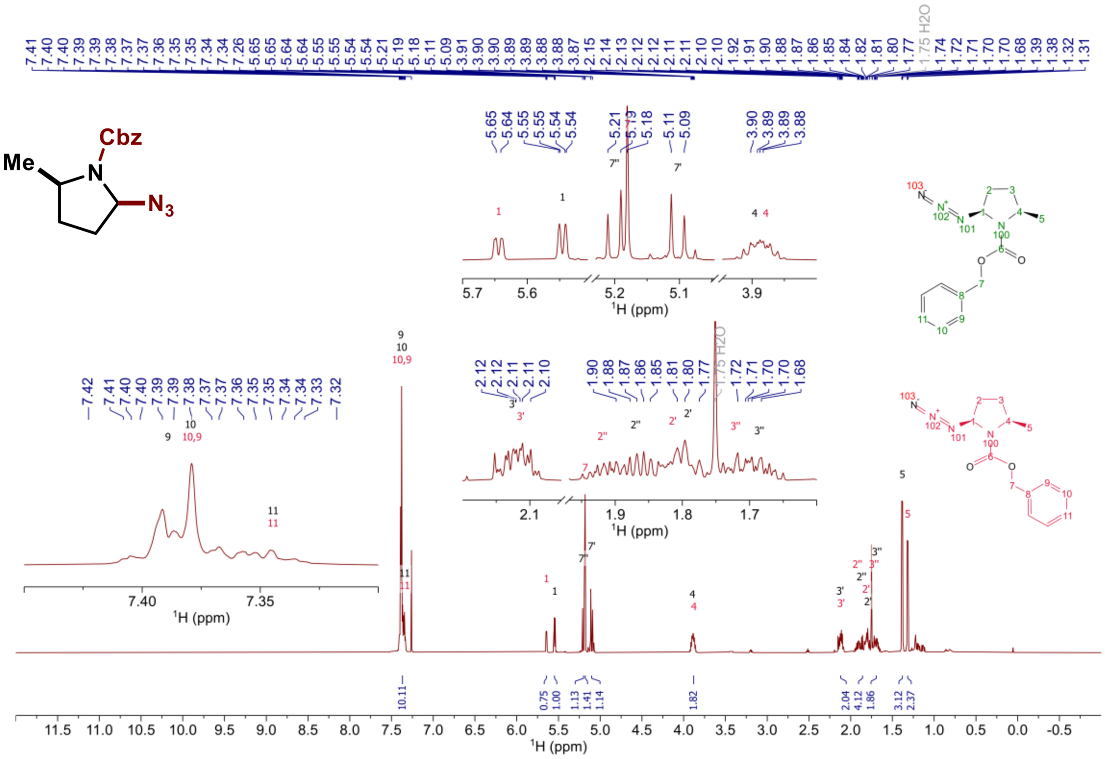

Compound **9d**, cis diastereomer: ^{13}C NMR (151 MHz, $CDCl_3$, 253 K)

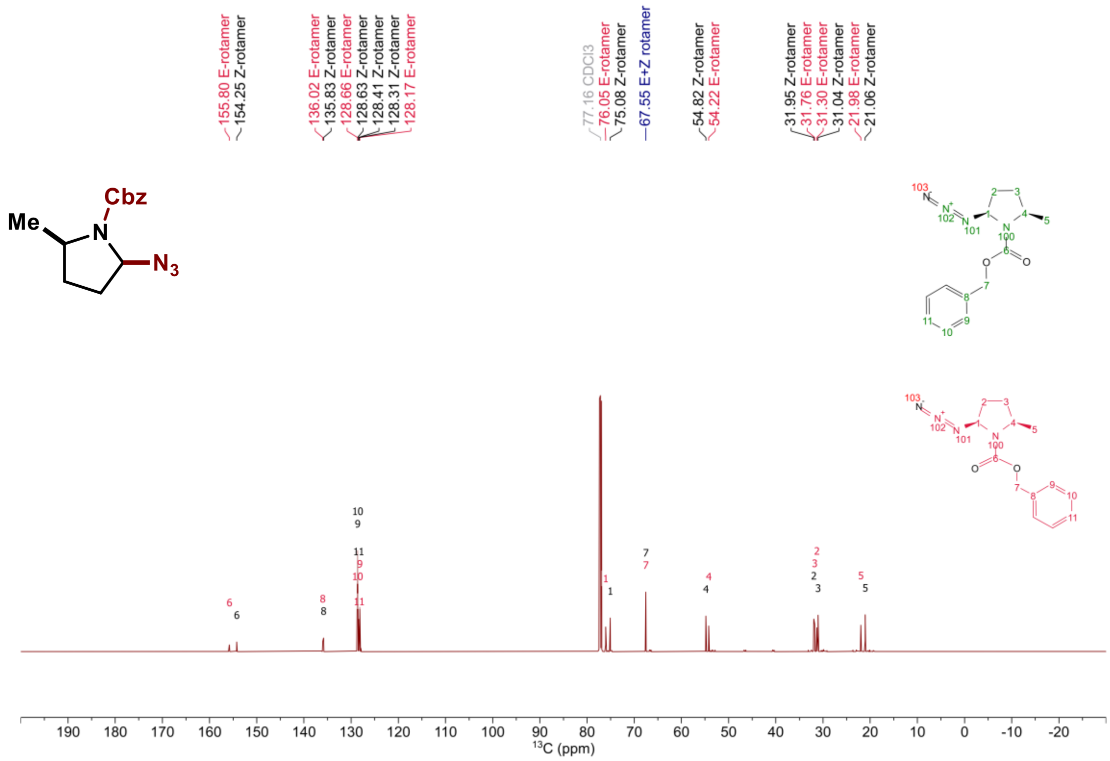

Compound **9d**, cis diastereomer: variable temperature ^{1}H NMR (600 MHz, $CDCl_3$)

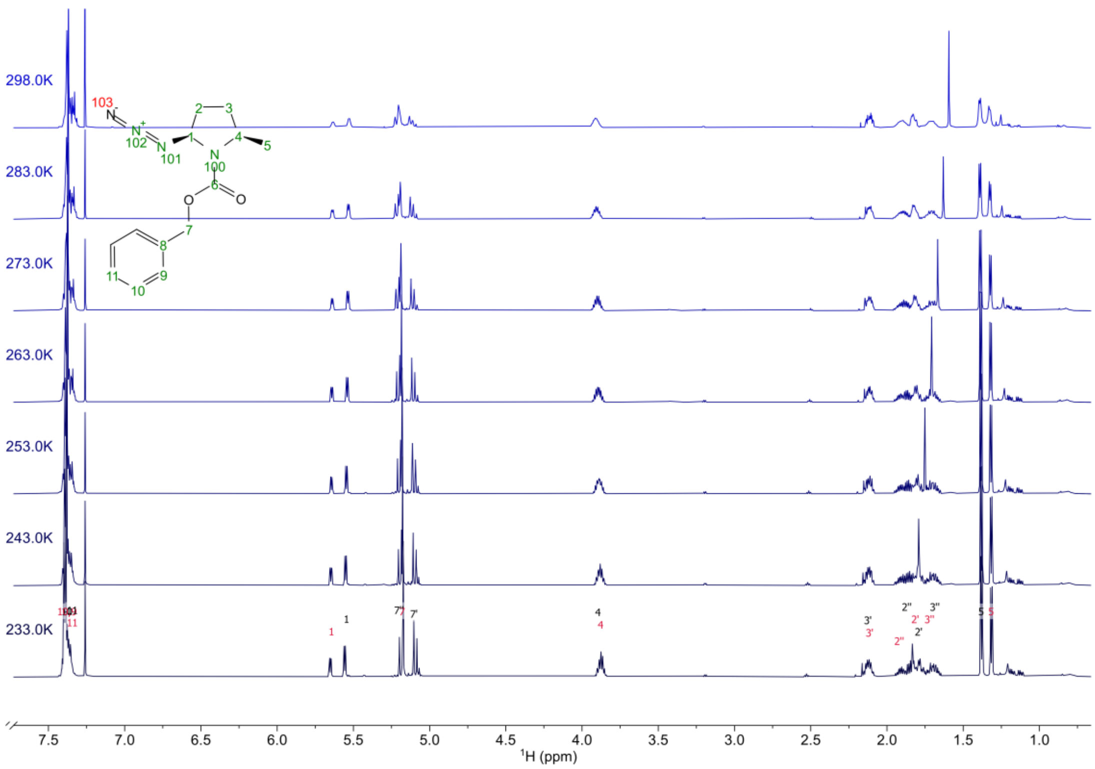

Compound **9d**, cis diastereomer: ^{1}H-^{13}C HSQC ($CDCl_3$, 253 K)

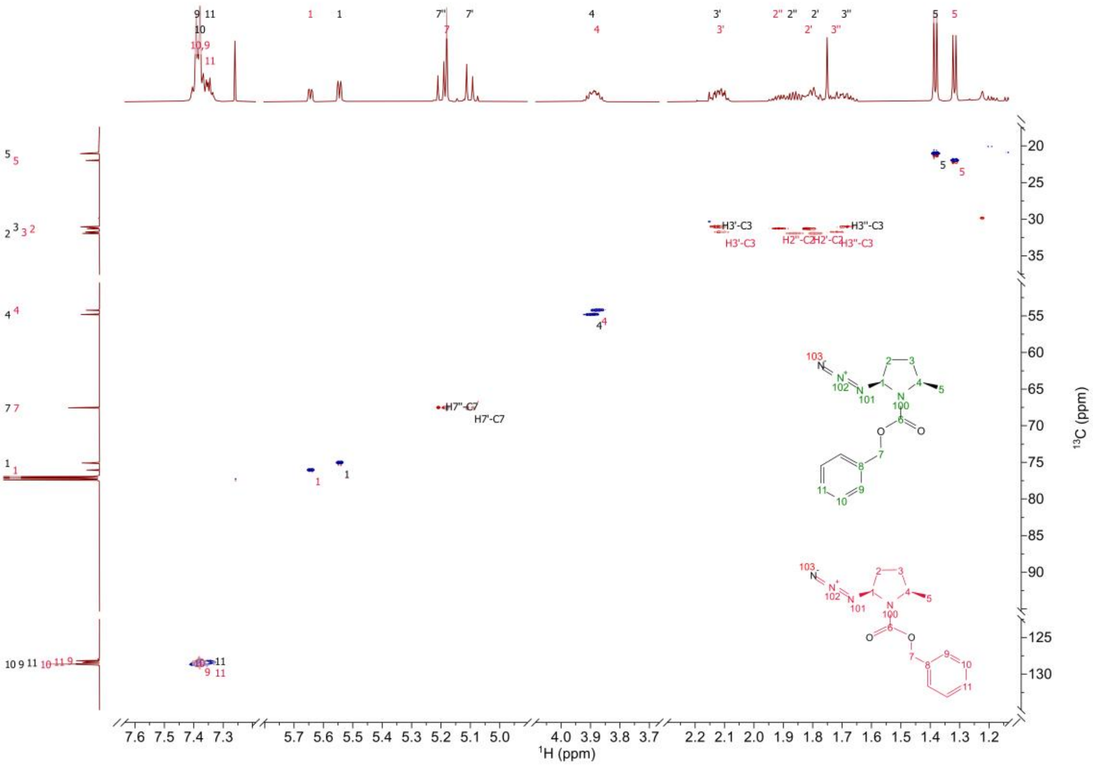

Compound **9d**, cis diastereomer: ^{1}H-^{13}C HMBC ($CDCl_3$, 253 K)

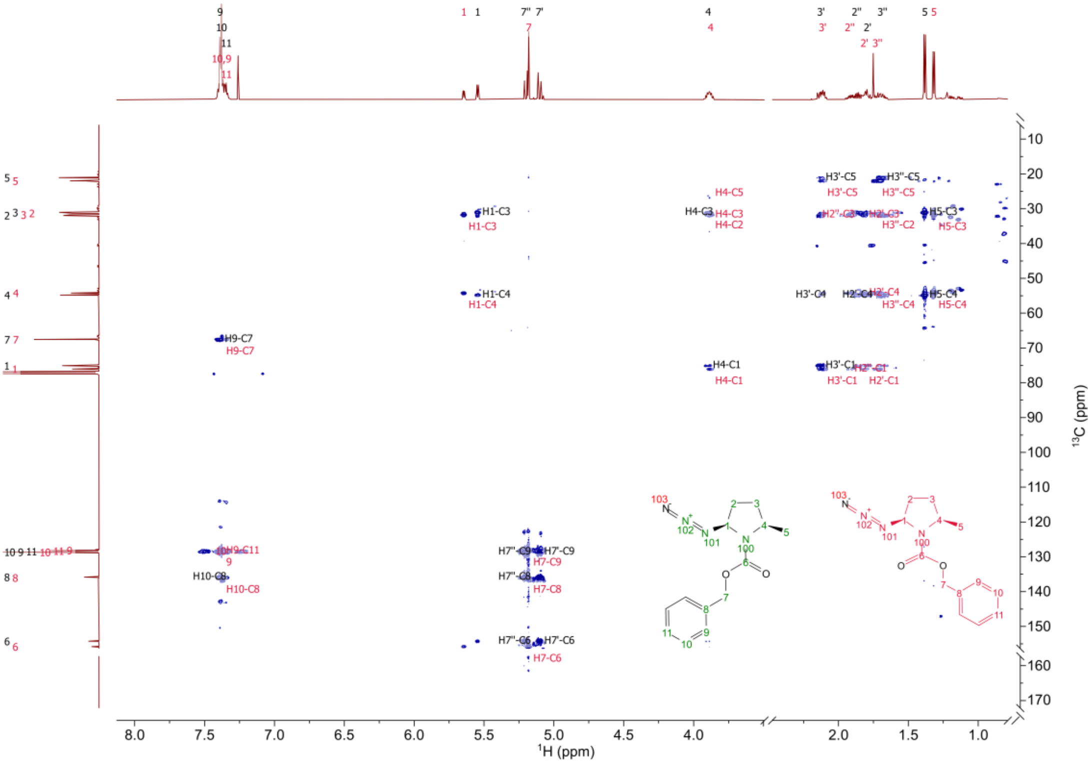

Compound **9d**, cis diastereomer: ^{1}H-^{1}H COSY ($CDCl_3$, 253 K)

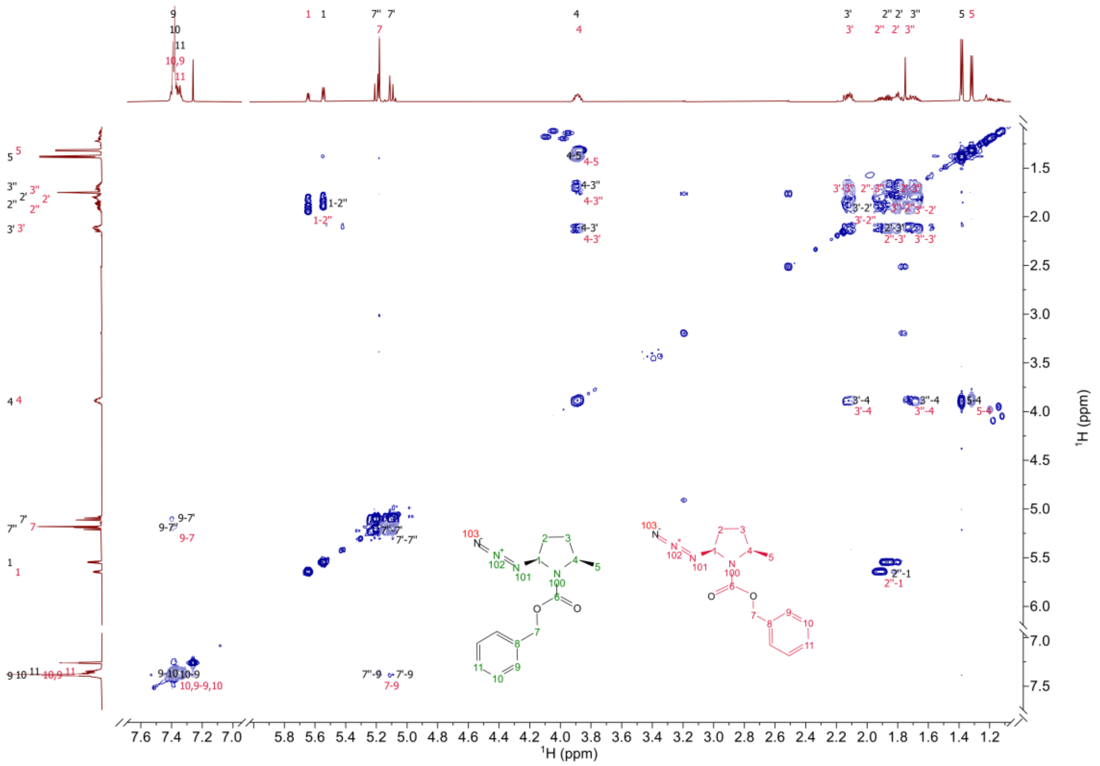

Compound **9d**, cis diastereomer: ^{1}H-^{1}H NOESY ($CDCl_3$, 253 K)

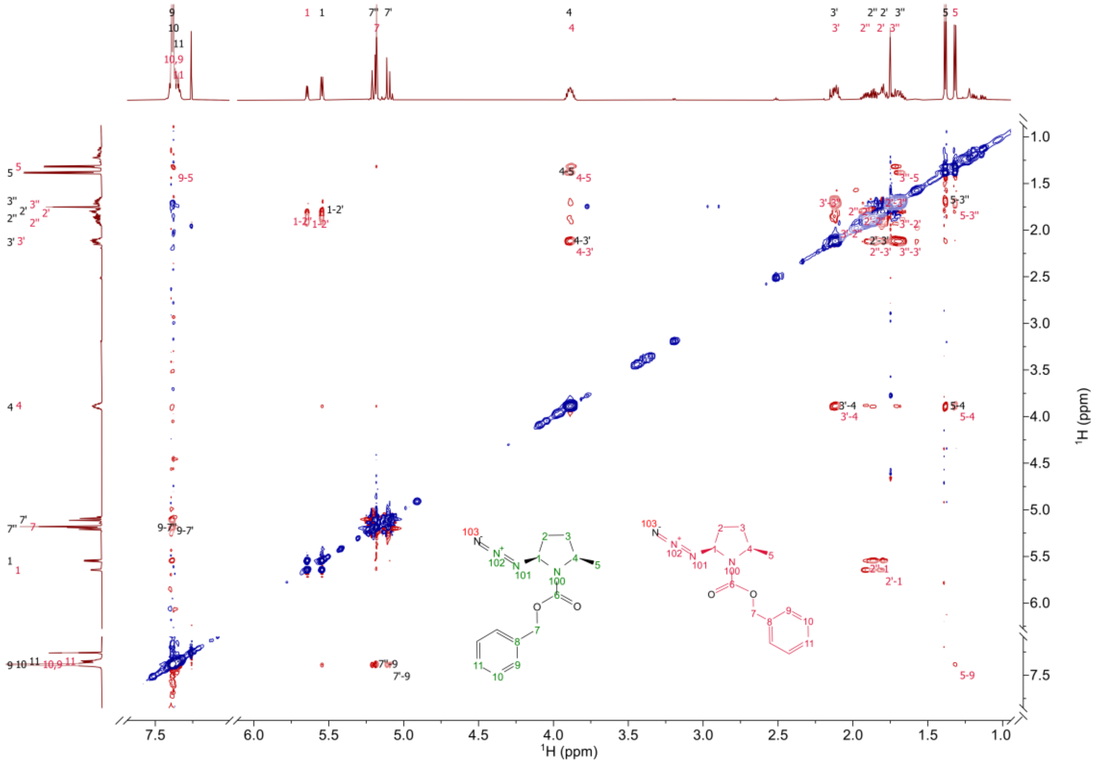

Compound **9d**, cis diastereomer: ^{1}H-^{15}N HMBC ($CDCl_3$, 253 K)

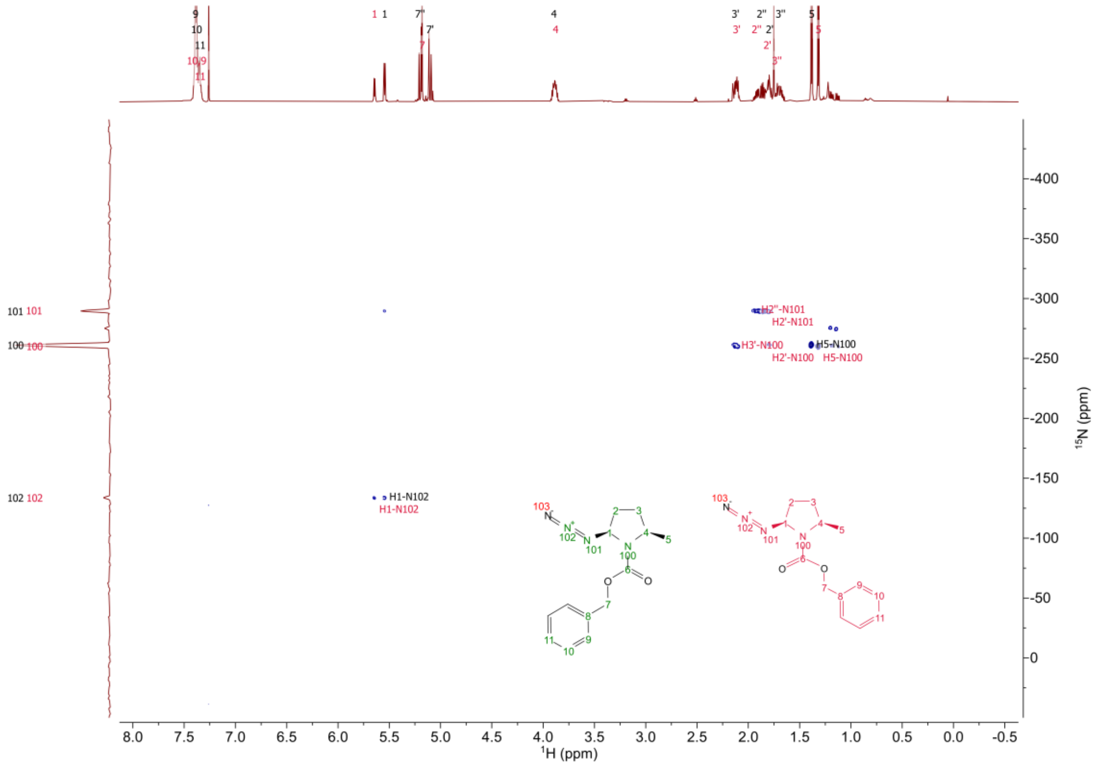

Compound **9d**, cis diastereomer: 1D selective TOCSY with excitation of H1

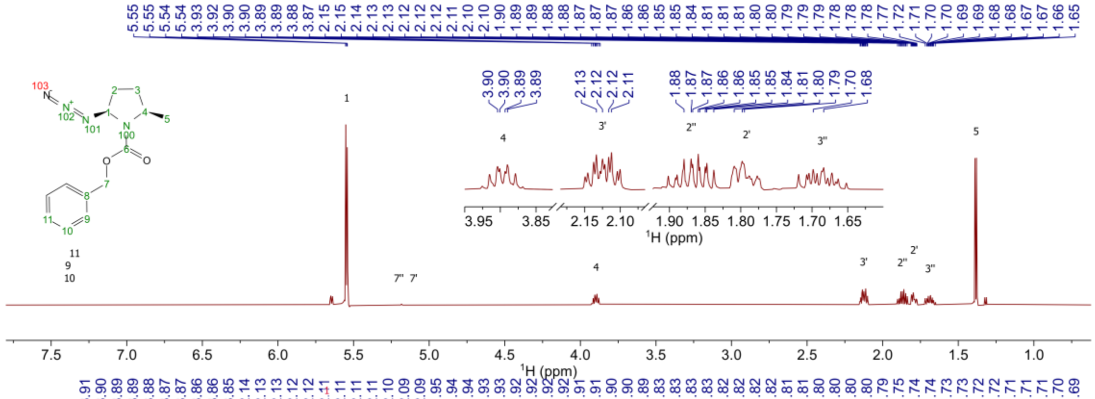

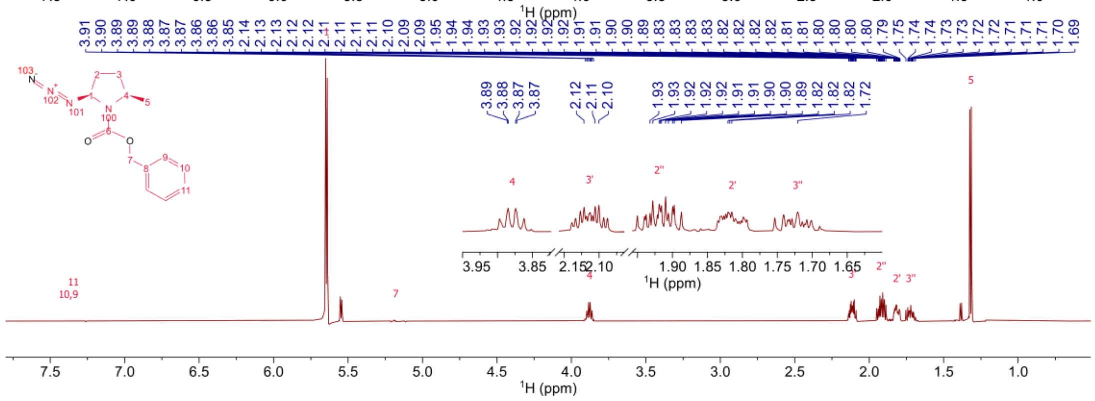

Compound **9e**, trans diastereomer: ^{1}H NMR (600 MHz, $CDCl_3$, 233 K)

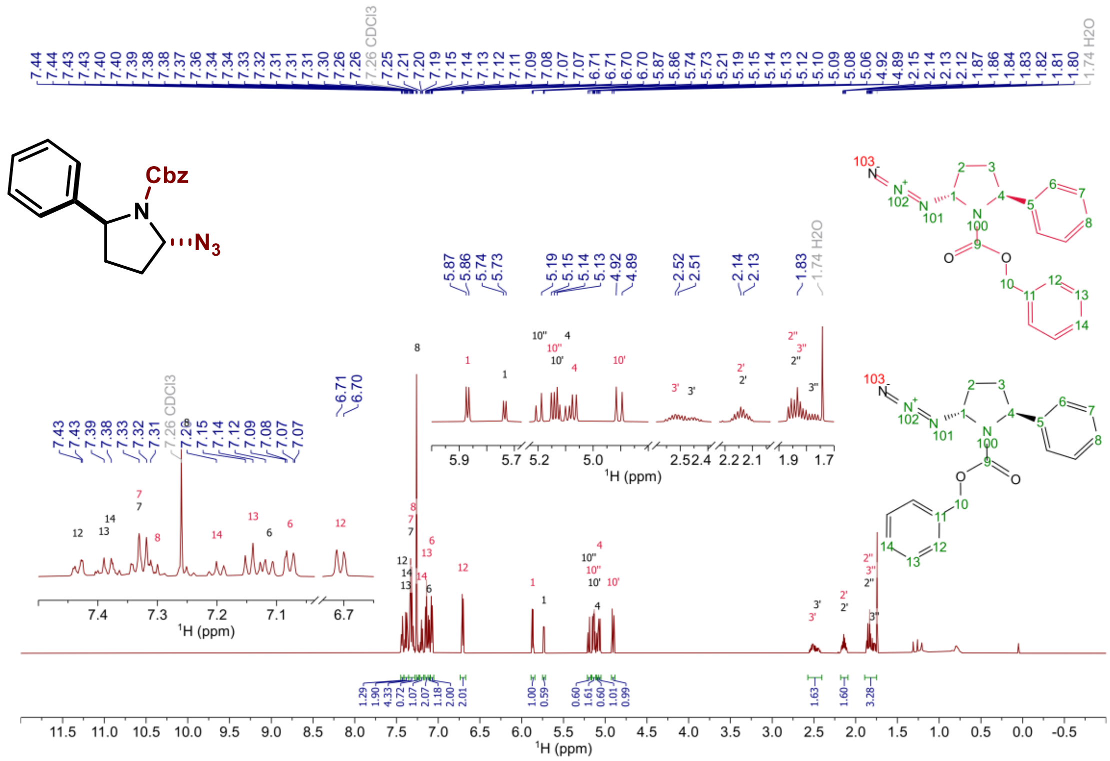

Compound **9e**, trans diastereomer: ^{13}C NMR (151 MHz, $CDCl_3$, 233 K)

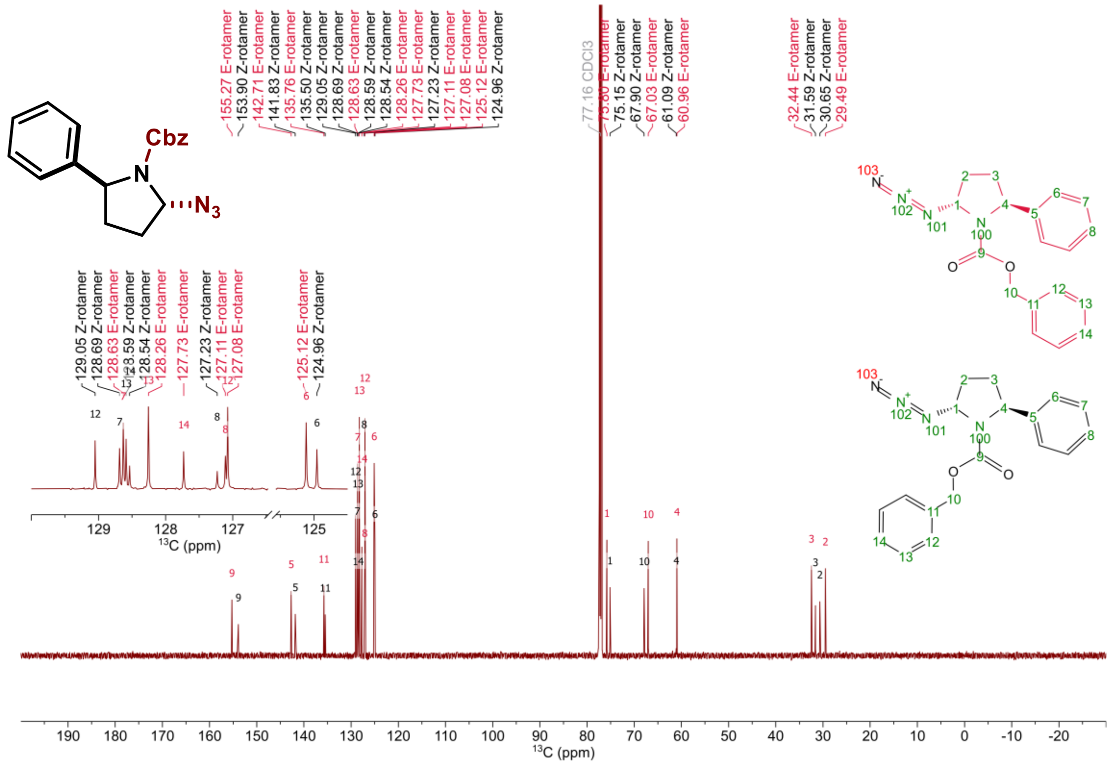

Compound **9e**, trans diastereomer: variable temperature ^{1}H NMR (600 MHz, $CDCl_3$)

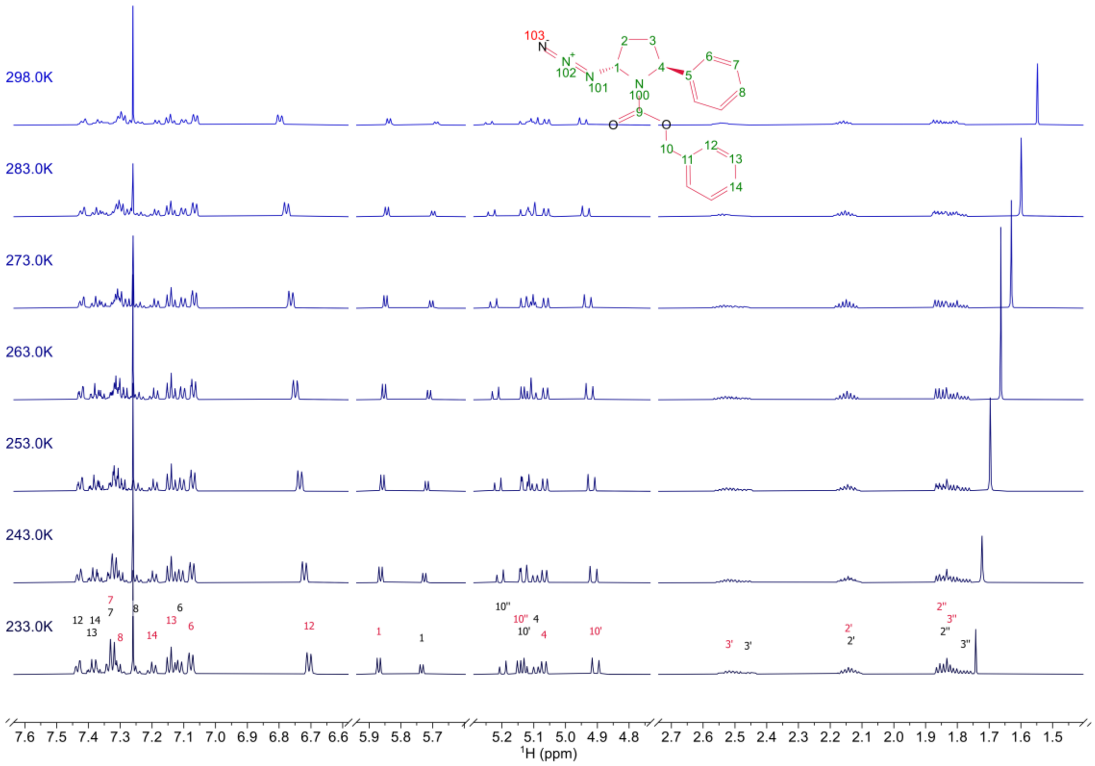

Compound **9e**, trans diastereomer: ^{1}H-^{13}C HSQC ($CDCl_3$, 233 K)

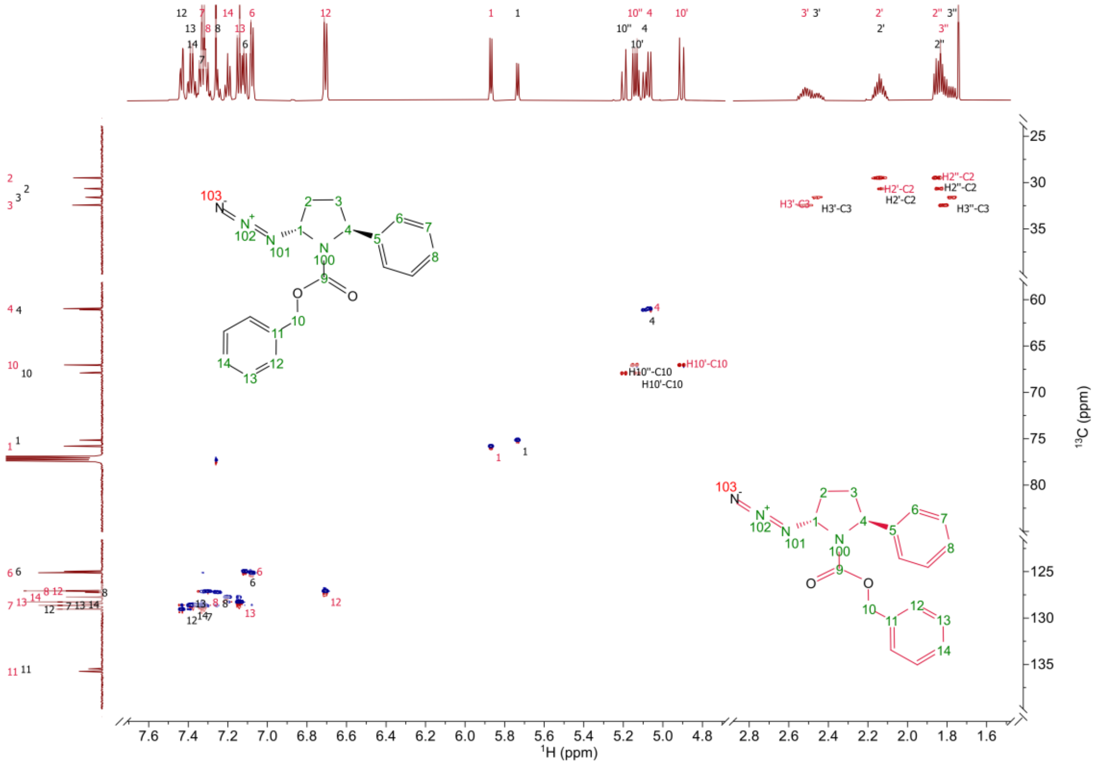

Compound **9e**, trans diastereomer: ^{1}H-^{13}C HMBC ($CDCl_3$, 233 K)

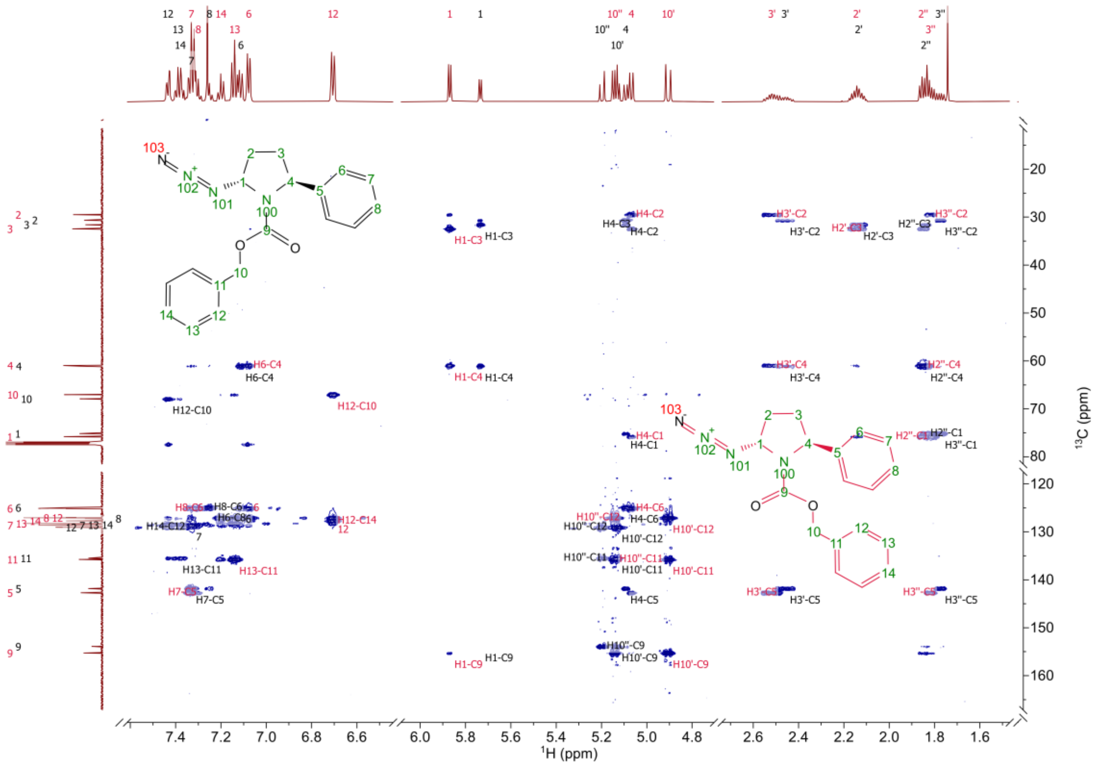

Compound **9e**, trans diastereomer: ^{1}H-^{1}H COSY ($CDCl_3$, 233 K)

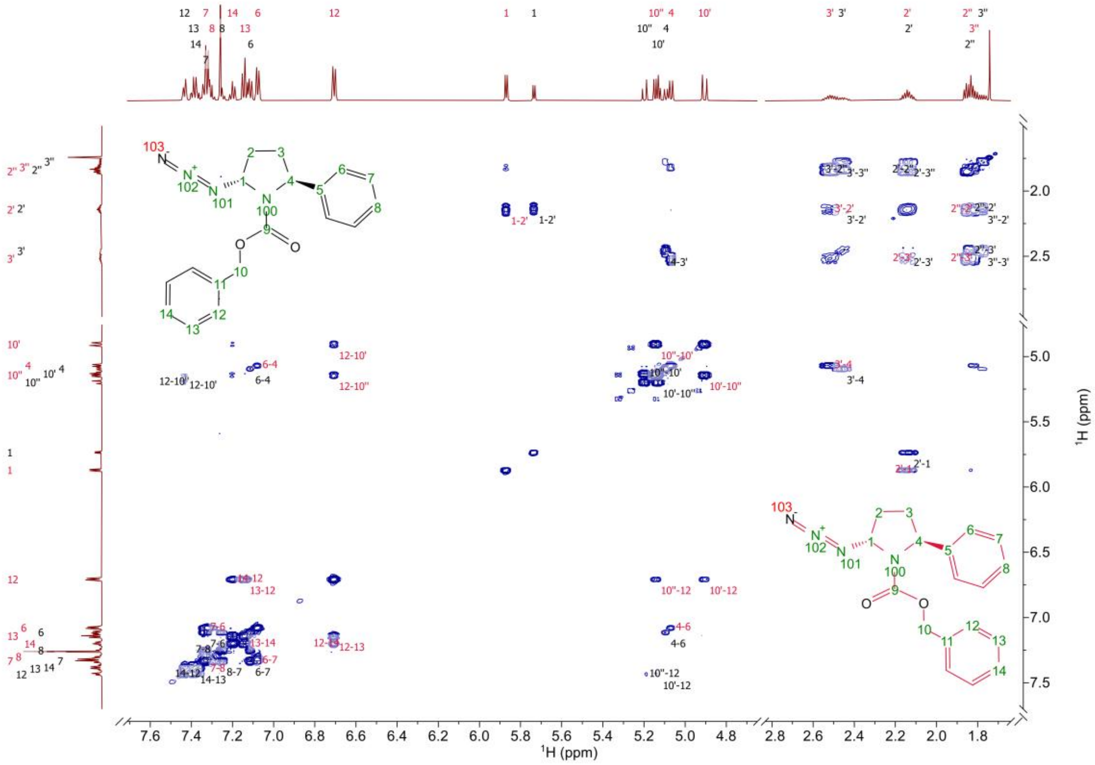

Compound **9e**, trans diastereomer: ^1H-^1H NOESY ($CDCl_3$, 233 K)

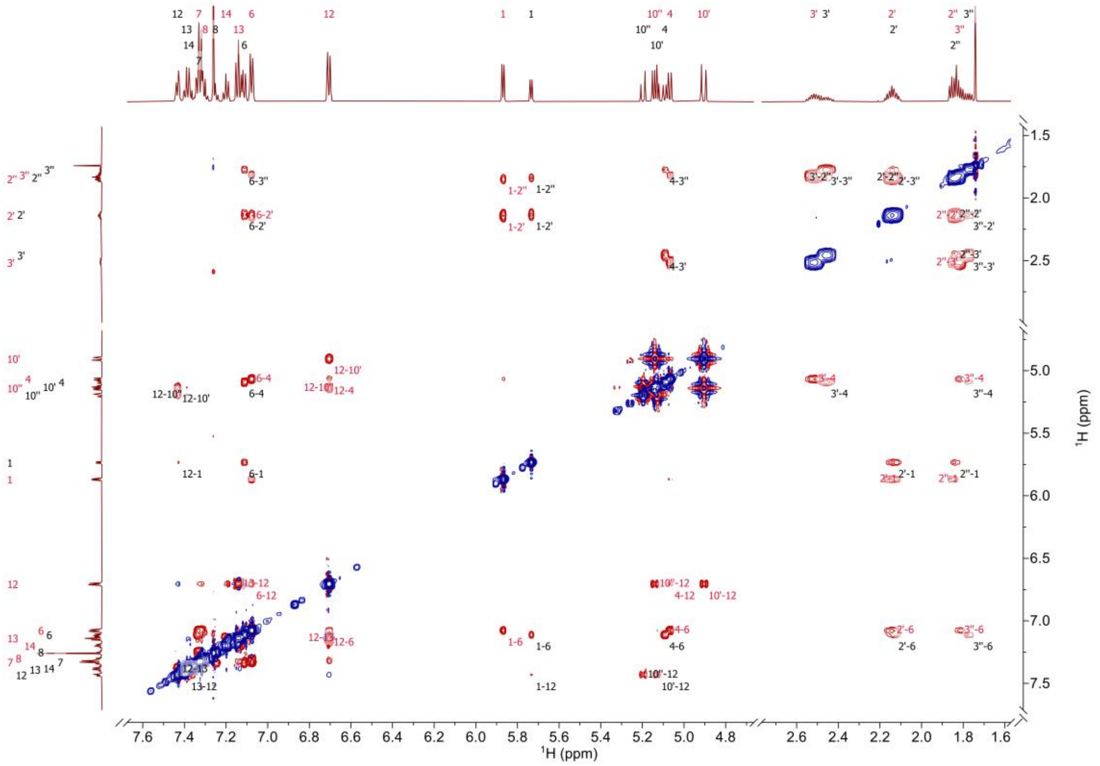

Compound **9e**, trans diastereomer: ^1H-^{15}N HMBC ($CDCl_3$, 233 K)

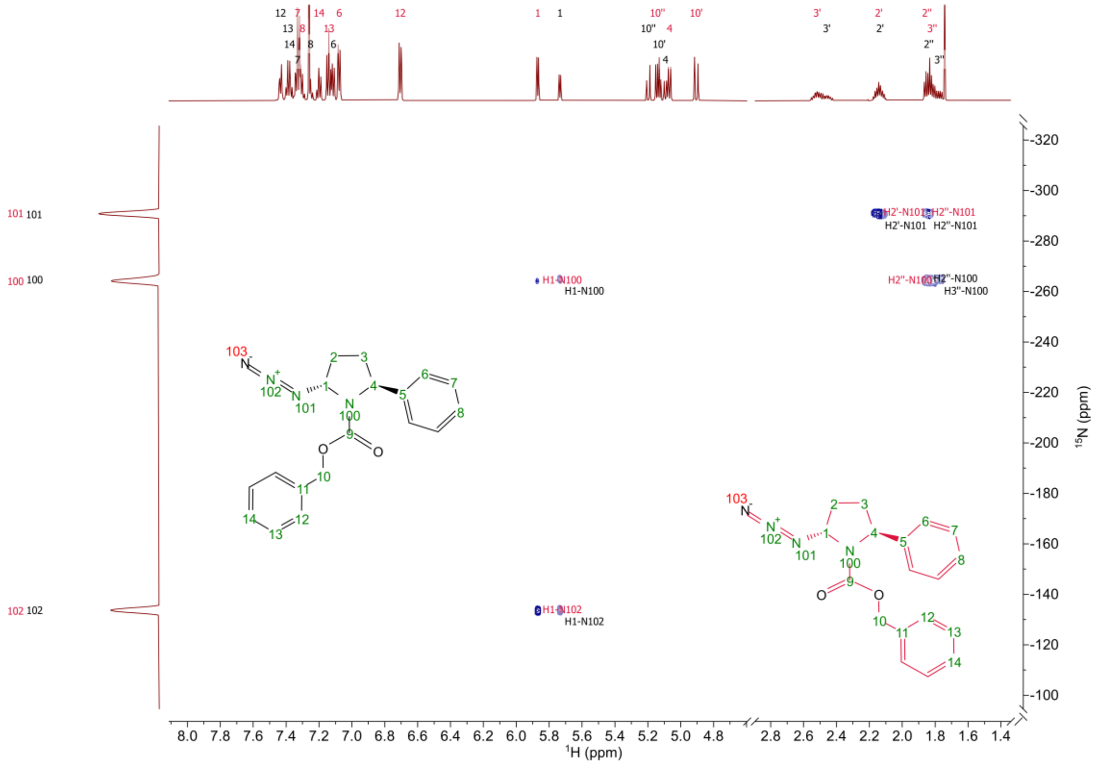

Compound **9e**, trans diastereomer: 1D selective TOCSY

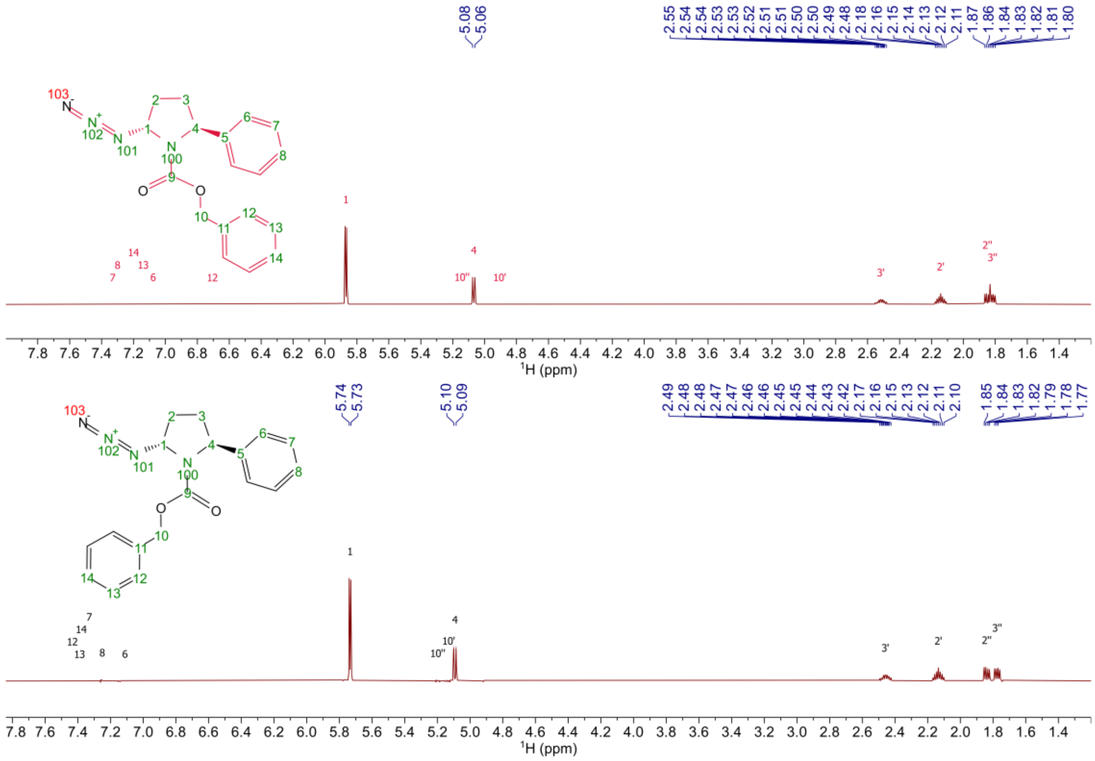

Compound **9e**, cis diastereomer: ^{1}H NMR (600 MHz, $CDCl_3$, 233 K)

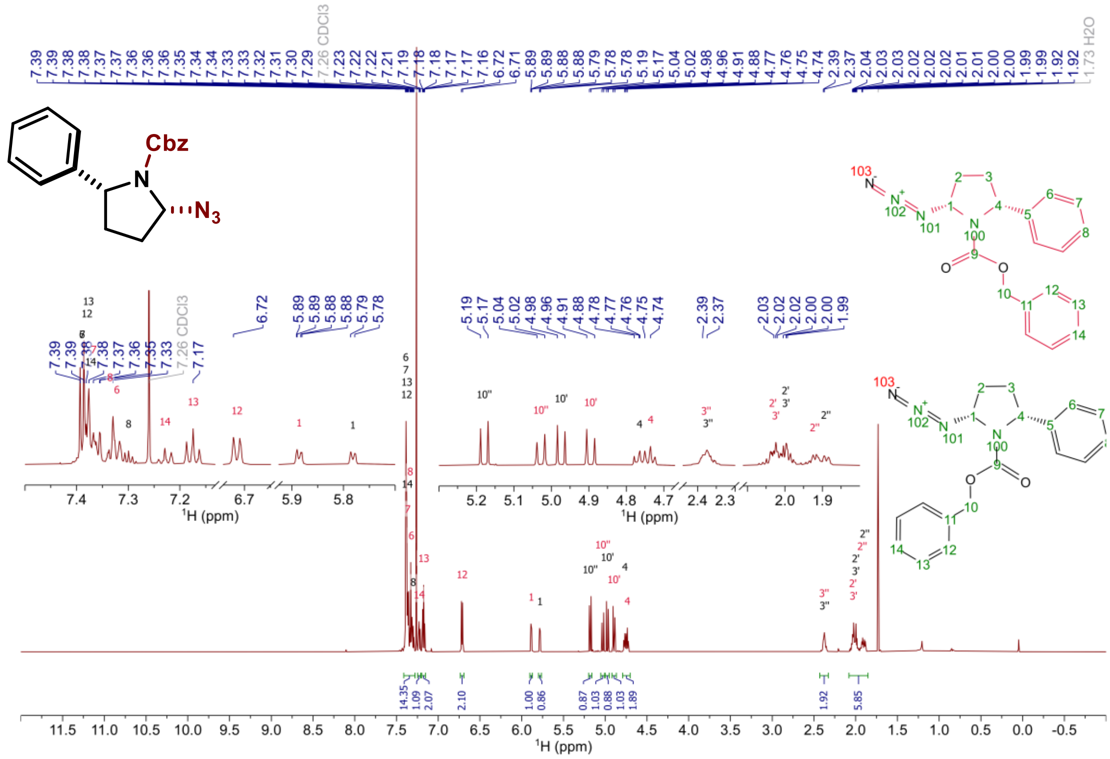

Compound **9e**, cis diastereomer: ^{13}C NMR (151 MHz, $CDCl_3$, 233 K)

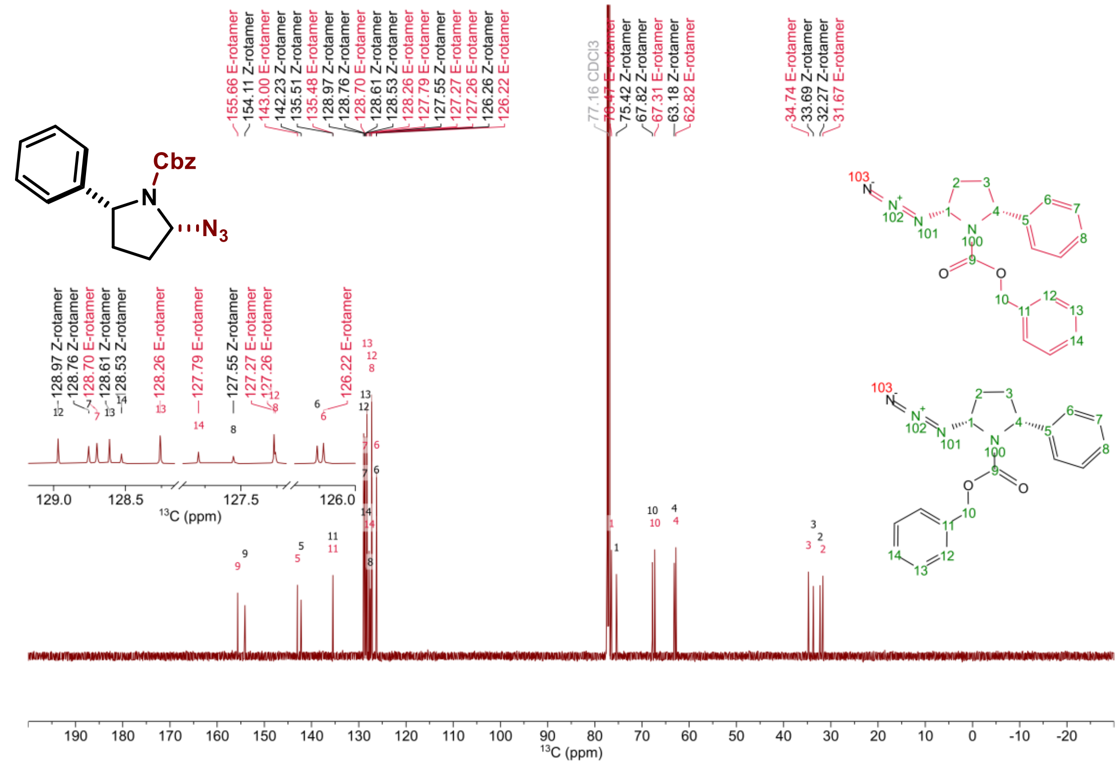

Compound **9e**, cis diastereomer: ^{1}H-^{13}C HSQC ($CDCl_3$, 233 K)

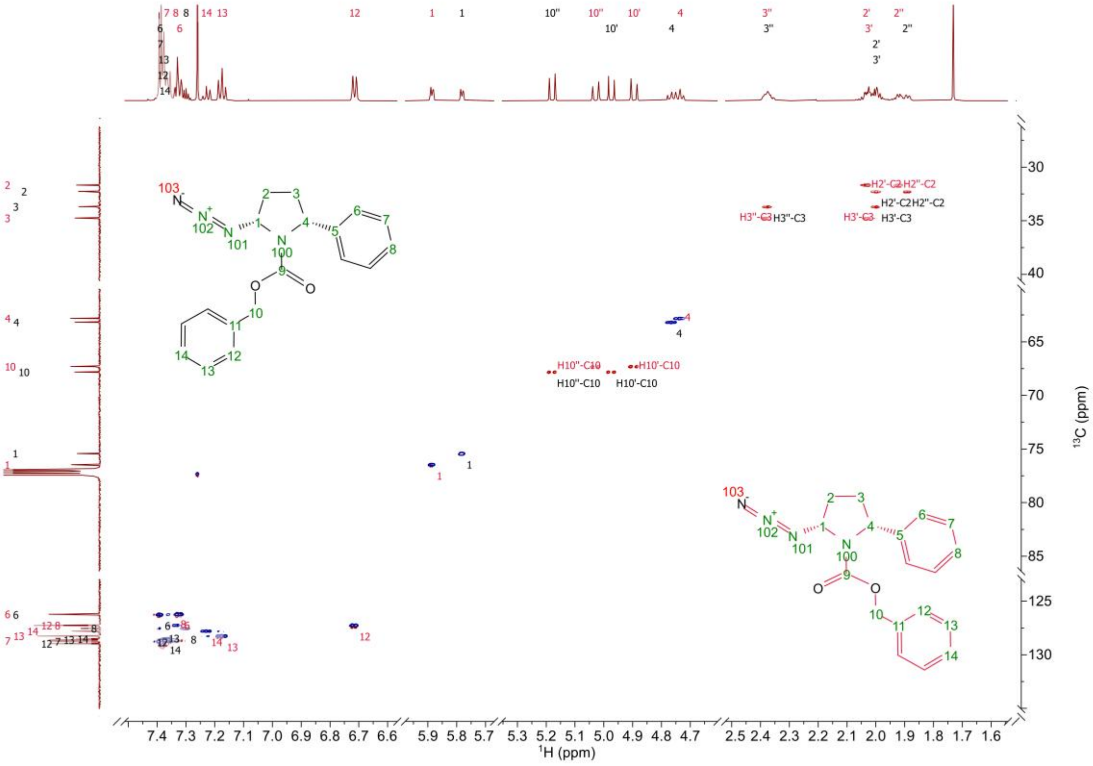

Compound **9e**, cis diastereomer: ^1H-^{13}C HMBC ($CDCl_3$, 233 K)

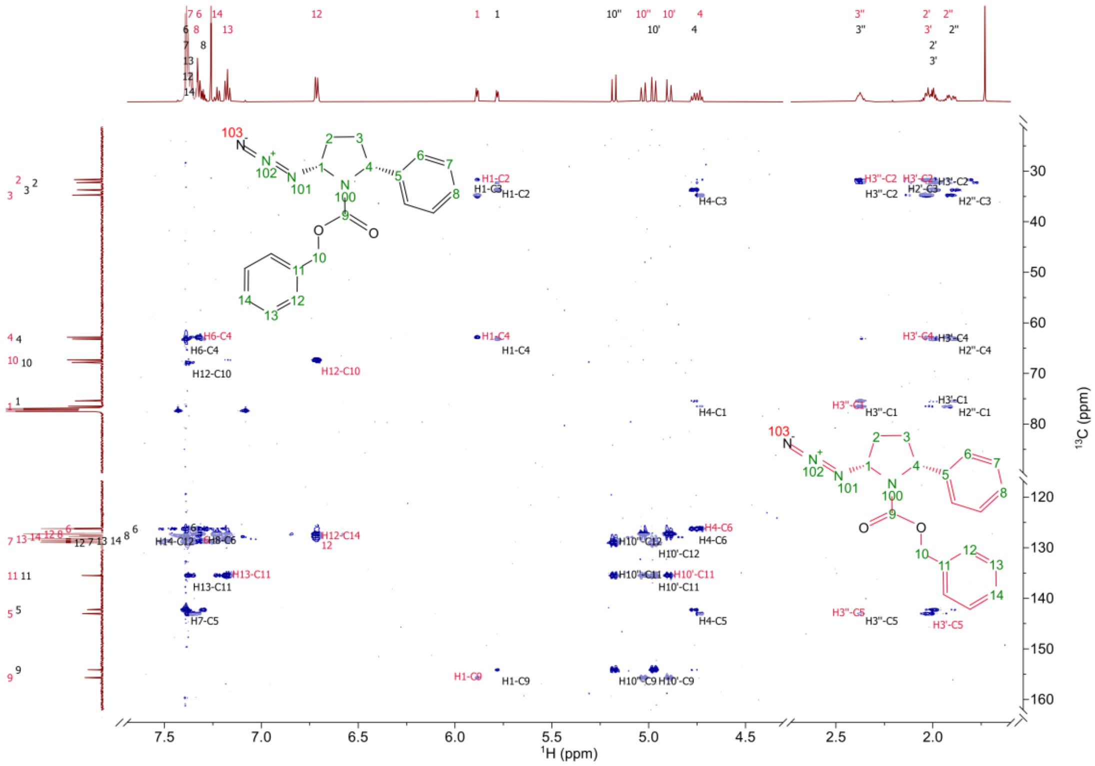

Compound **9e**, cis diastereomer: ^1H-^1H COSY ($CDCl_3$, 233 K)

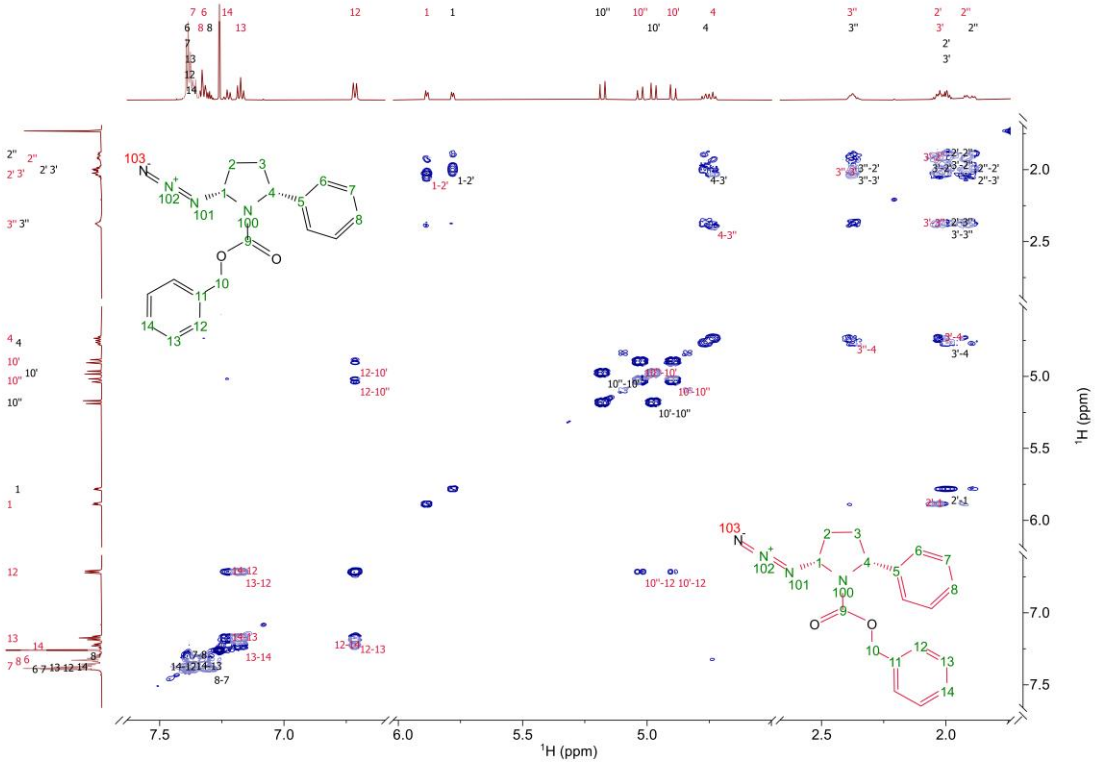

Compound **9e**, cis diastereomer: ^{1}H-^{1}H NOESY ($CDCl_3$, 233 K)

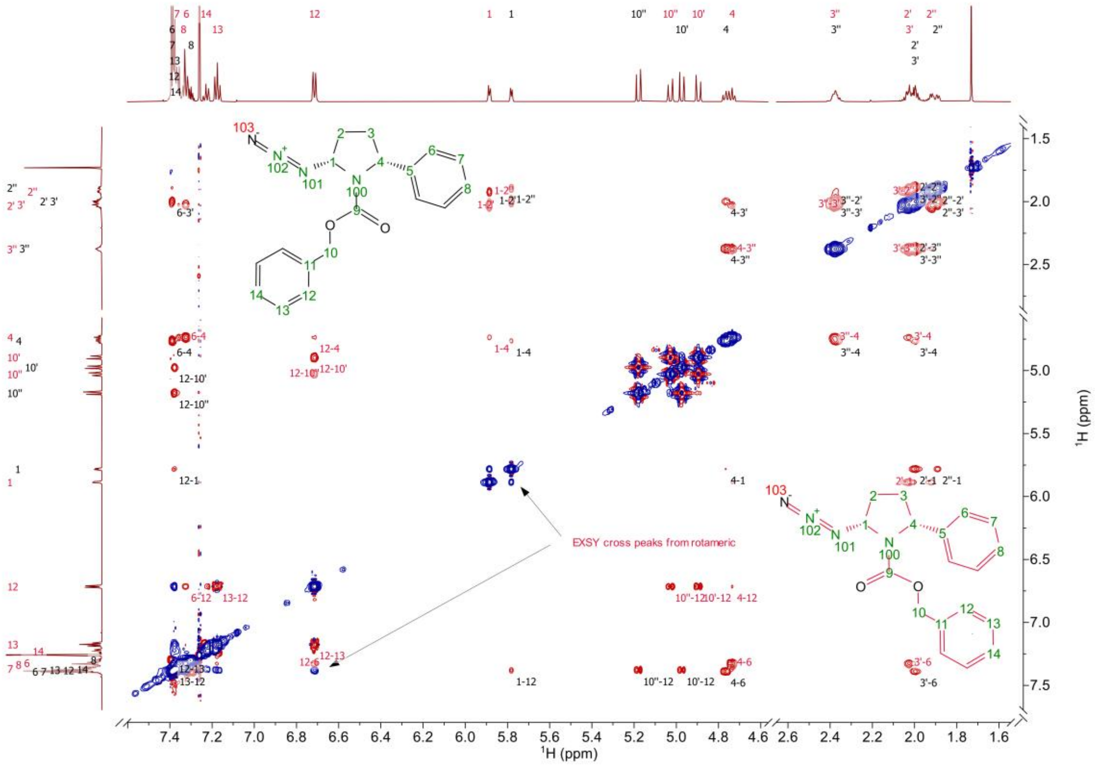

Compound **9e**, cis diastereomer: ^{1}H-^{15}N HMBC ($CDCl_3$, 233 K)

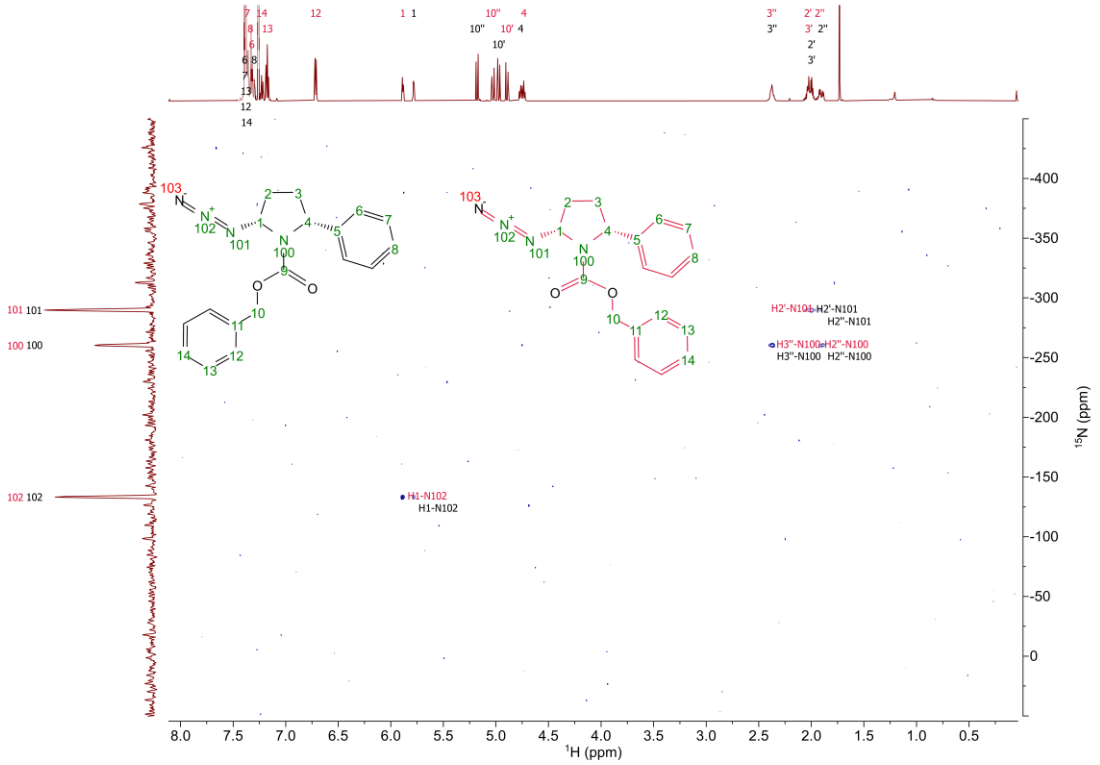

Compound **9e**, cis diastereomer: 1D selective TOCSY

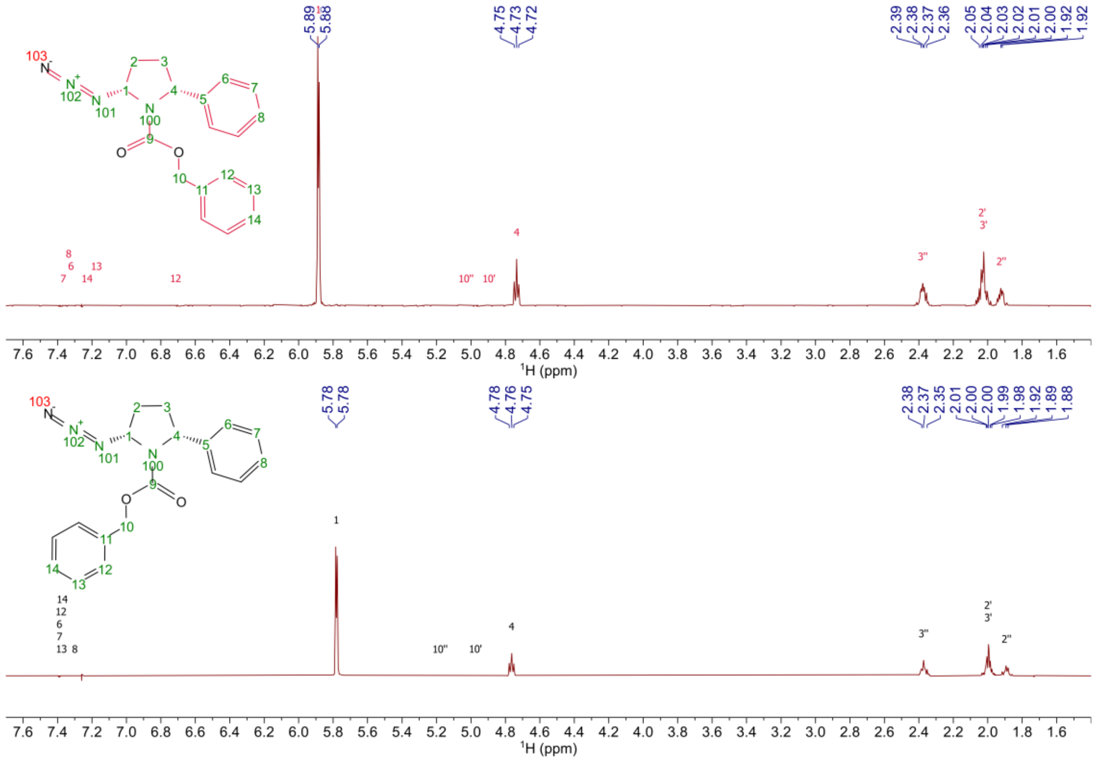

Compound **9f**: ^{1}H NMR (600 MHz, $CDCl_3$, 298 K)

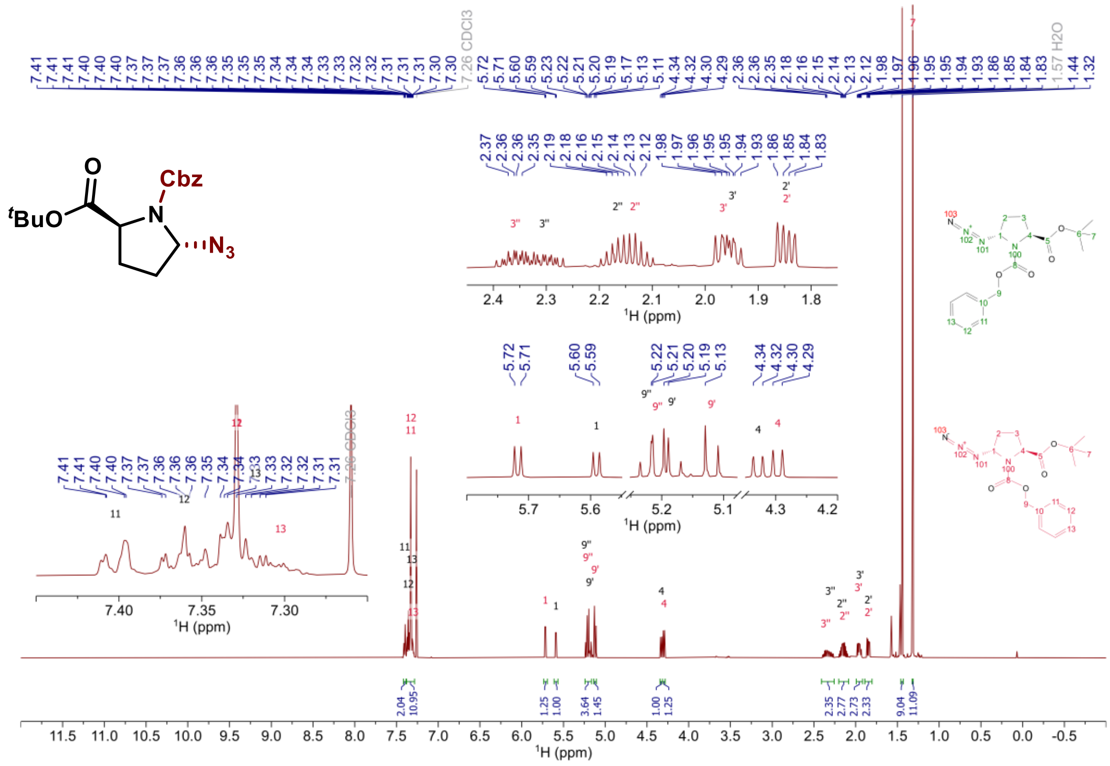

Compound **9f**: ^{13}C NMR (151 MHz, $CDCl_3$, 298 K)

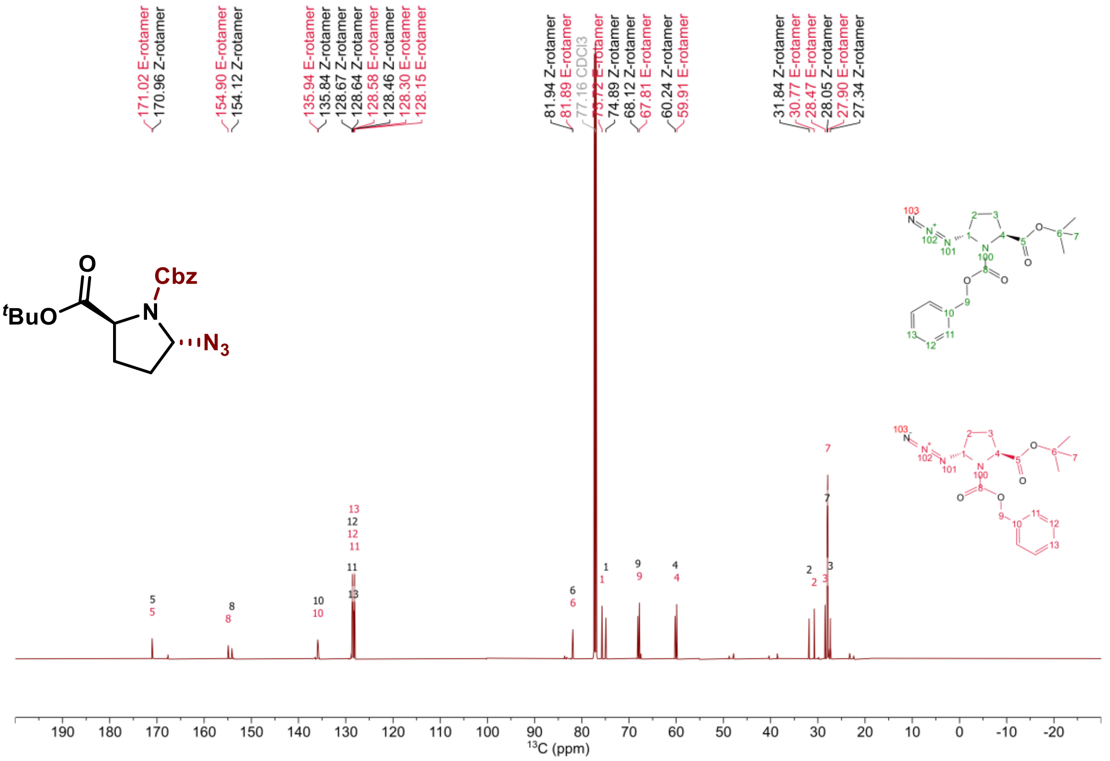

Compound **9f**: ^1H-^{13}C HSQC ($CDCl_3$, 298 K)

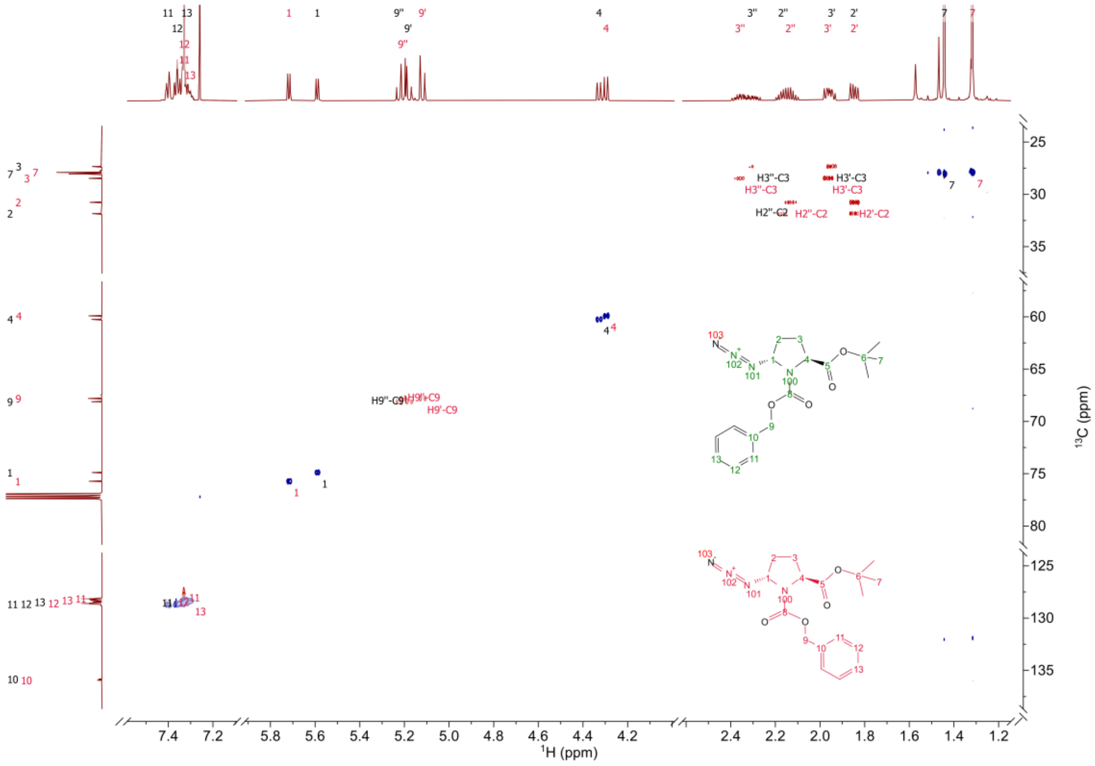

Compound **9f**: ^{1}H-^{13}C HMBC ($CDCl_3$, 298 K)

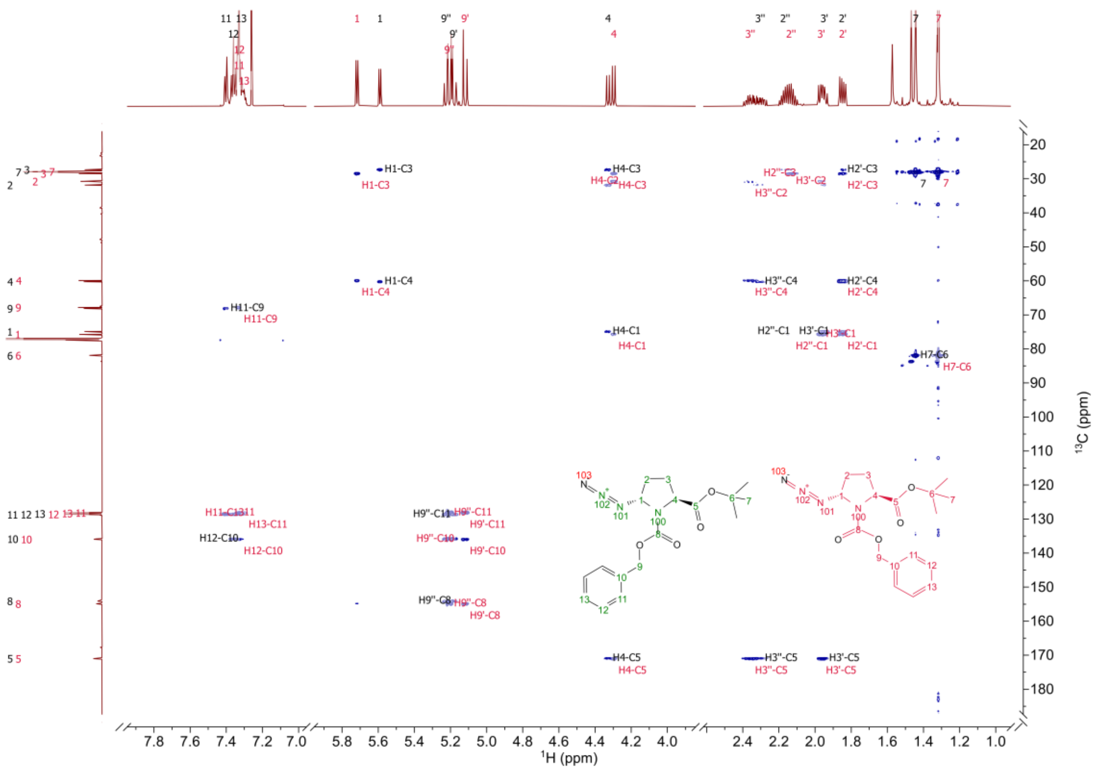

Compound **9f**: ^{1}H-^{1}H COSY ($CDCl_3$, 298 K)

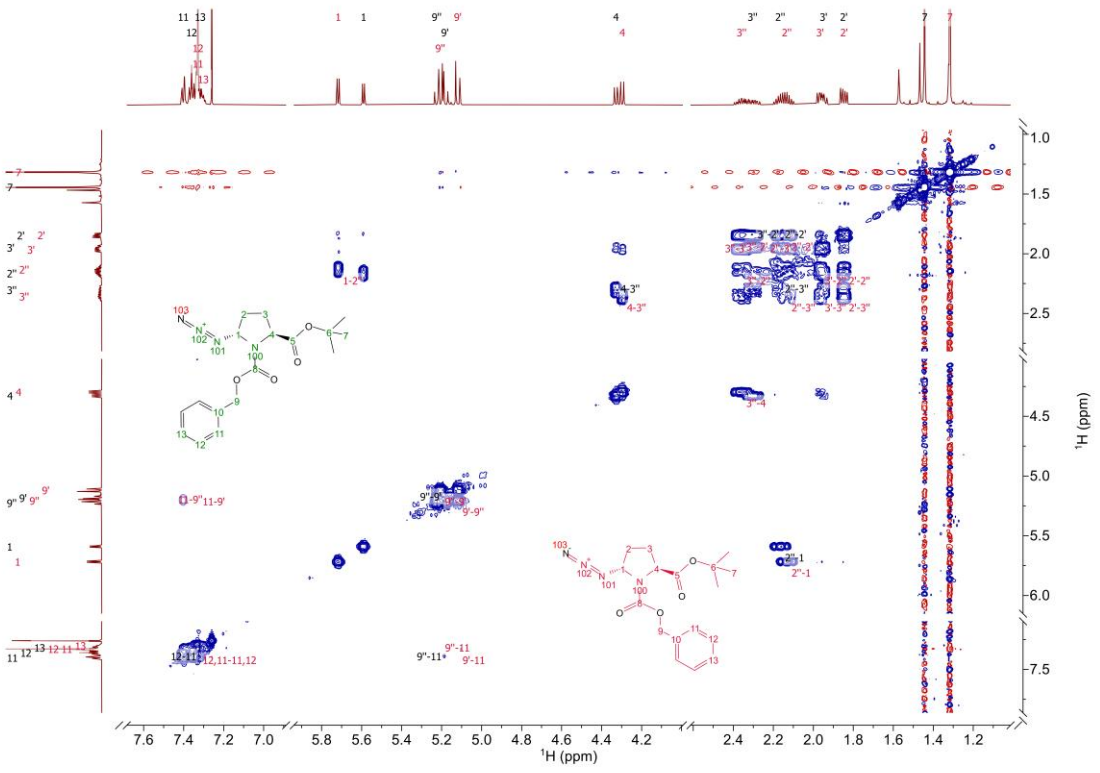

Compound **9f**: ^{1}H-^{1}H NOESY ($CDCl_3$, 298 K)

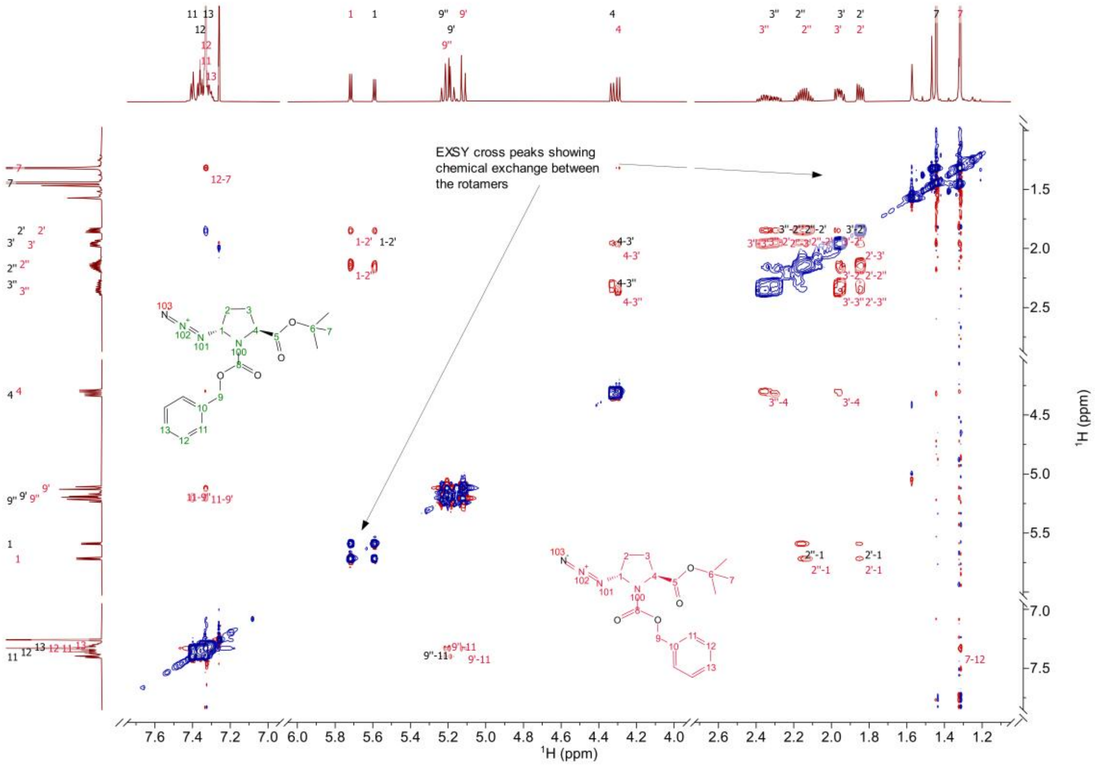

Compound **9f**: ^{1}H-^{15}N HMBC ($CDCl_3$, 298 K)

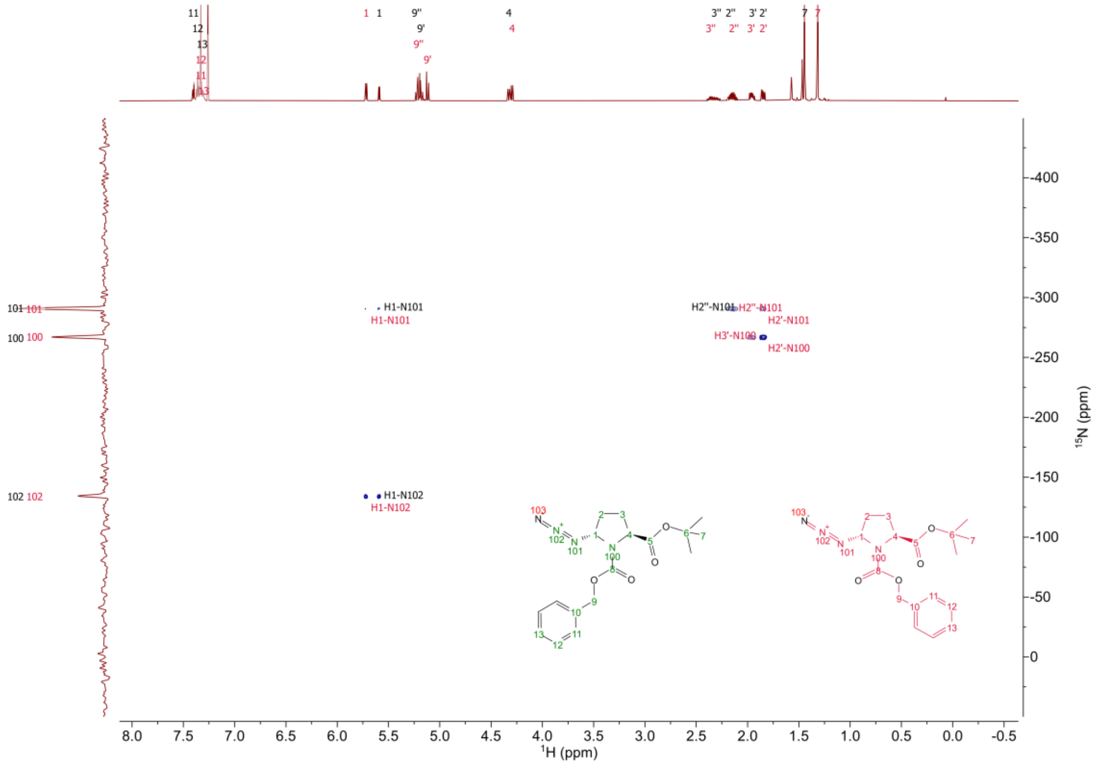

Compound **9h**: ^{1}H NMR (600 MHz, $CDCl_3$, 333 K)

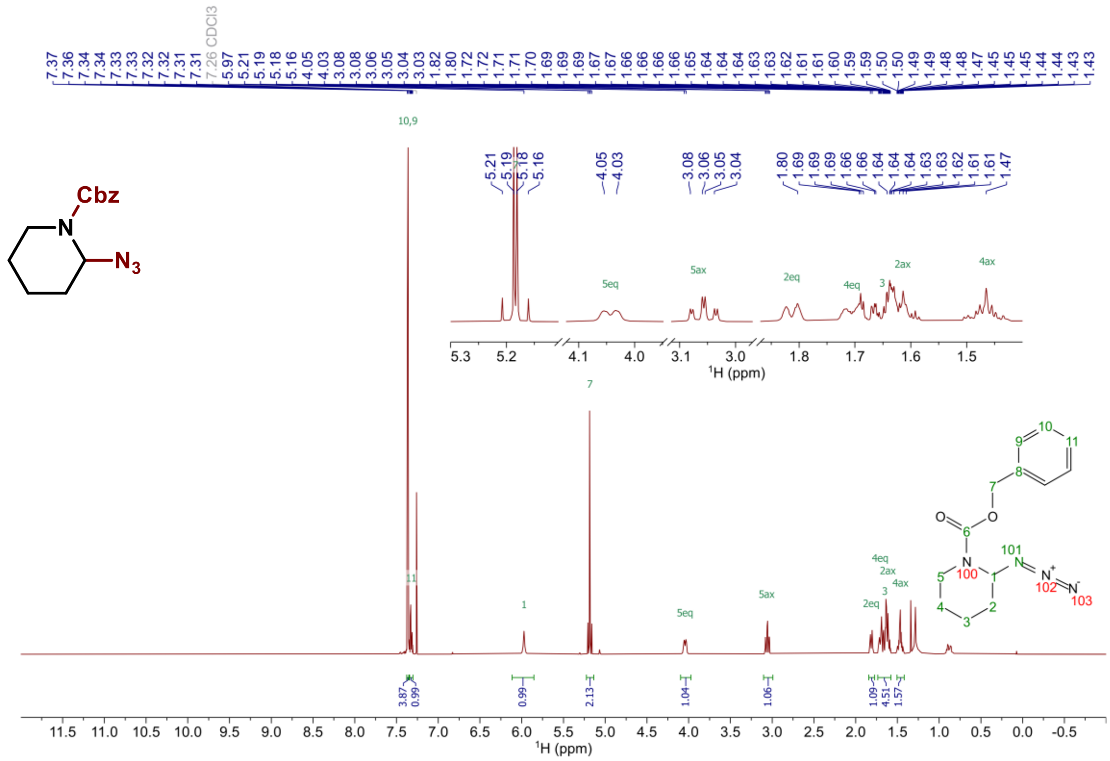

Compound **9h**: ^{13}C NMR (151 MHz, $CDCl_3$, 333 K)

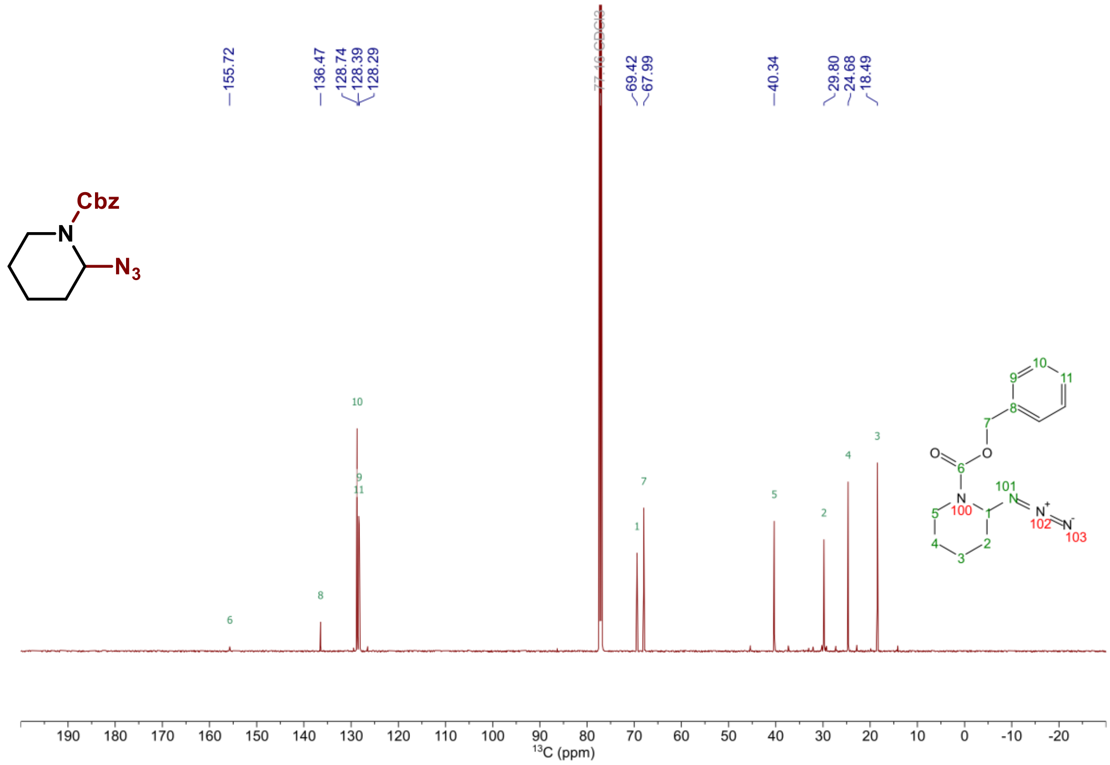

Compound **9h**: variable temperature ^1H NMR (600 MHz, $CDCl_3$)

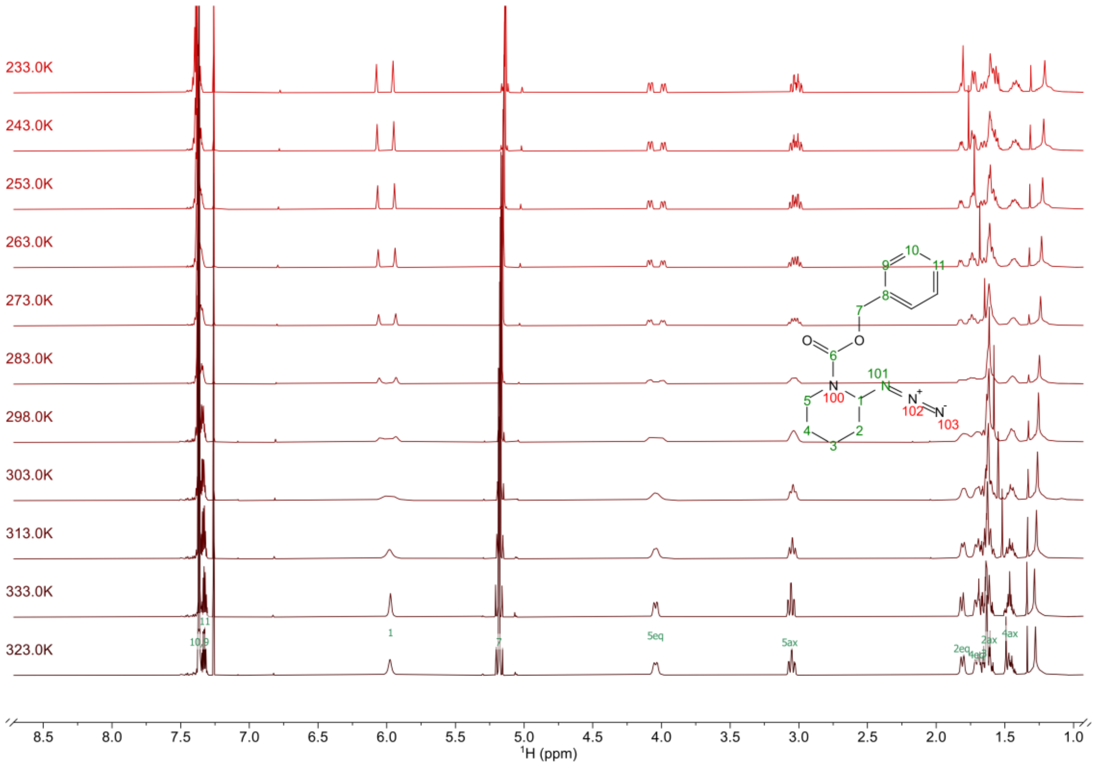

Compound **9h**: ^1H-^{13}C HSQC ($CDCl_3$, 333 K)

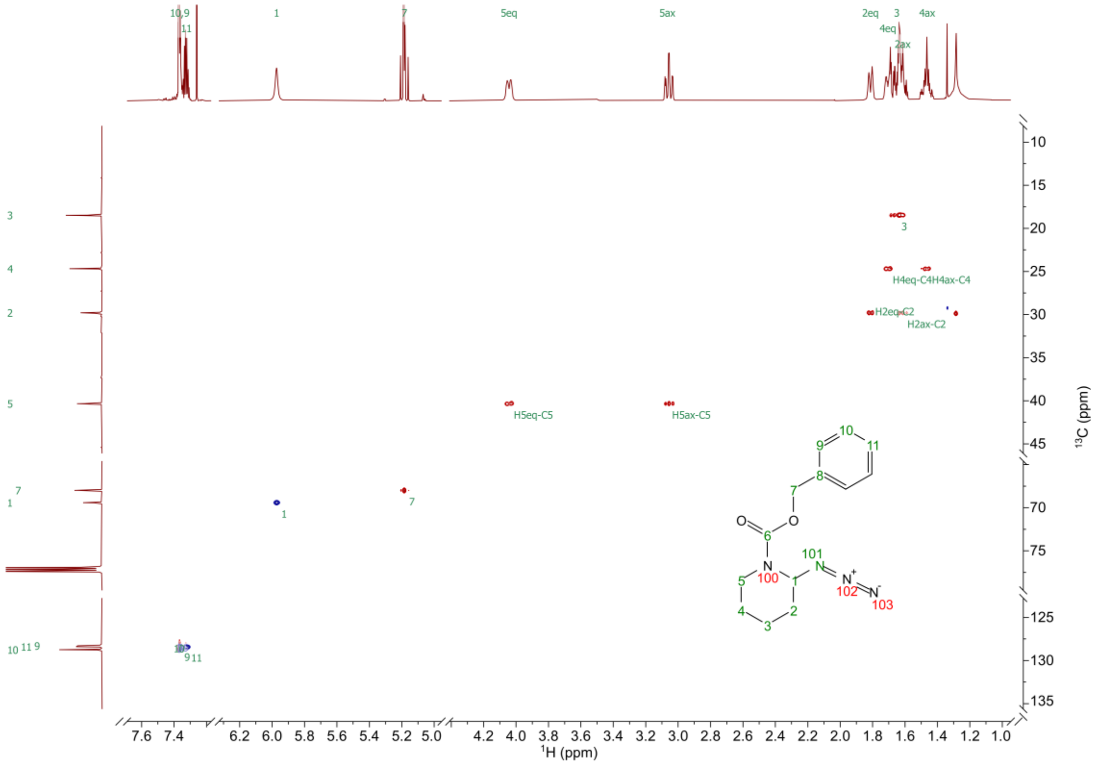

Compound **9h**: ^{1}H-^{13}C HMBC ($CDCl_3$, 333 K)

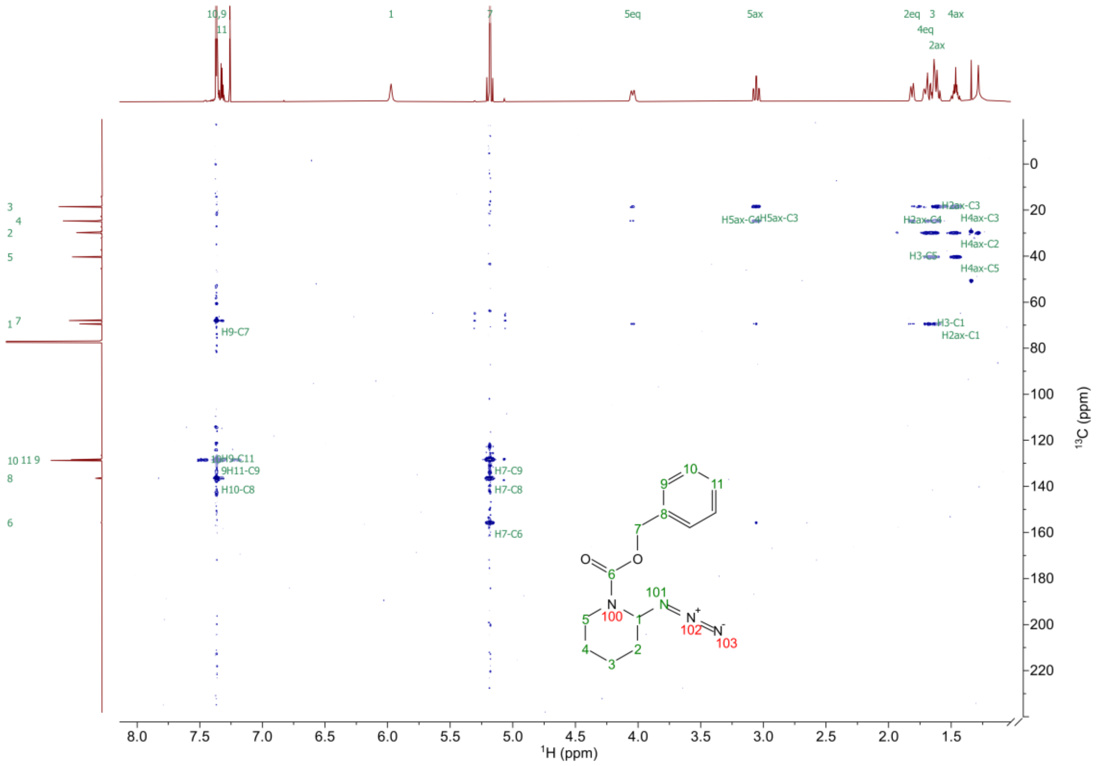

Compound **9h**: ^{1}H-^{1}H COSY ($CDCl_3$, 333 K)

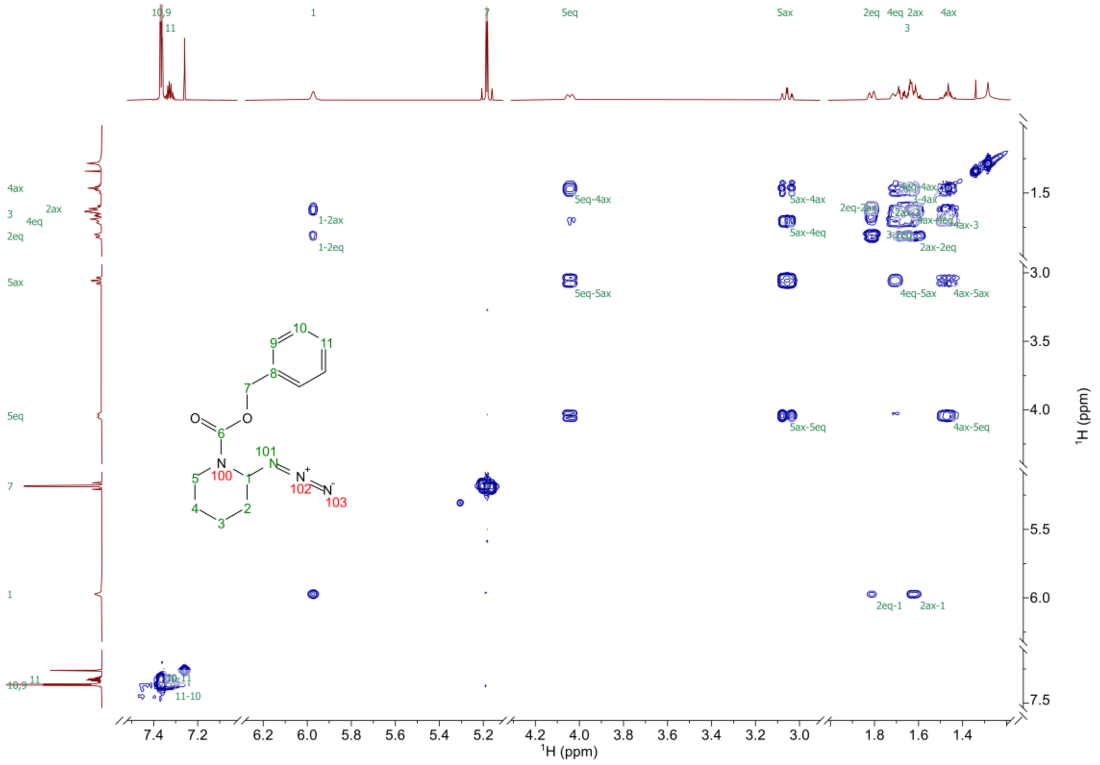

Compound **9h**: ^{1}H-^{15}N HMBC ($CDCl_3$, 333 K)

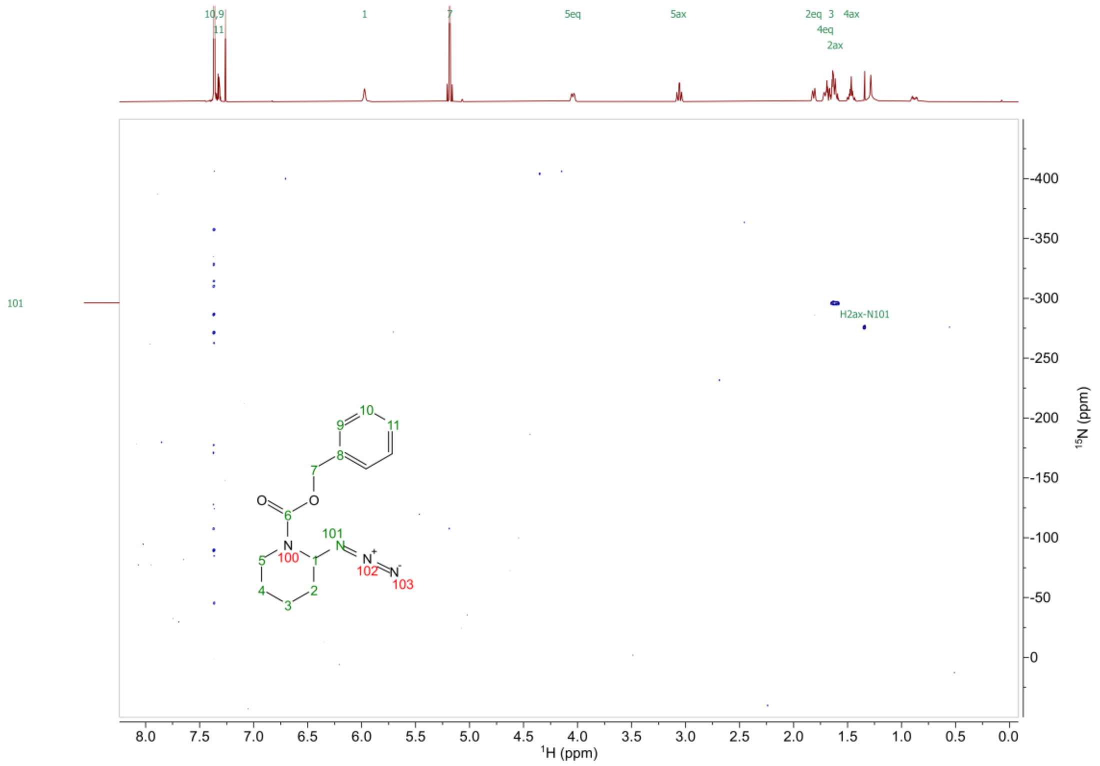

Compound **9i**: ^{1}H NMR ($CDCl_3$, 253 K)

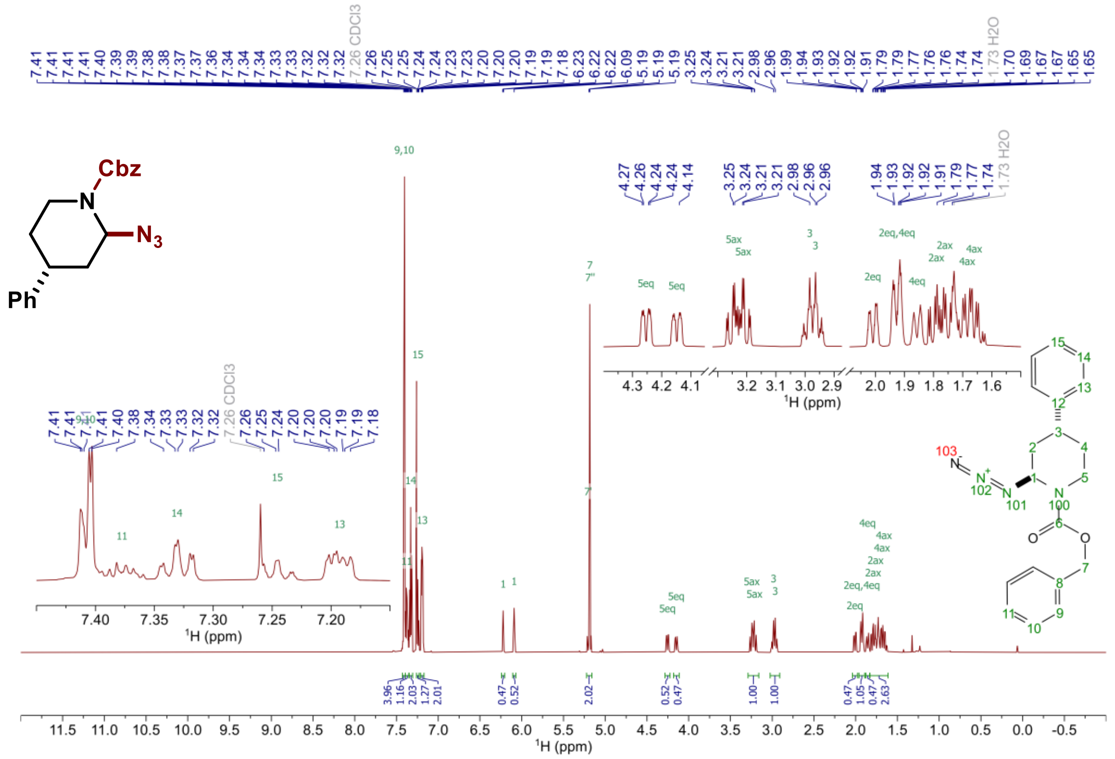

Compound **9i**: ^{13}C NMR ($CDCl_3$, 253 K)

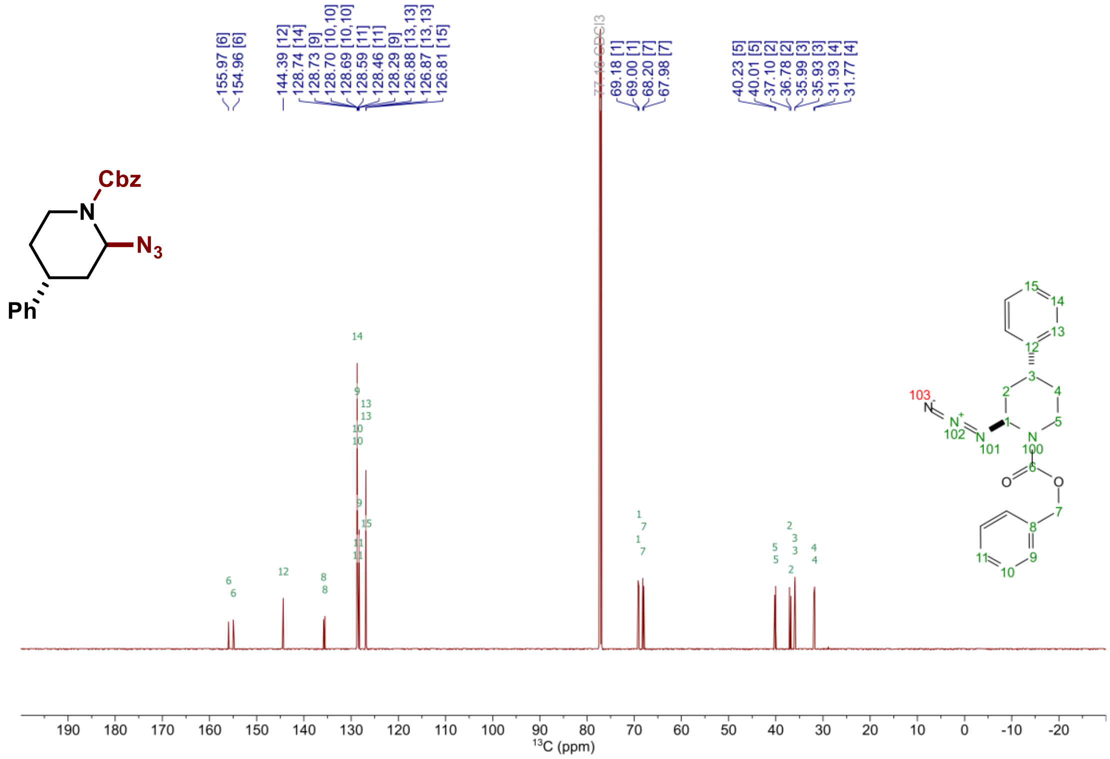

Compound **9i**: ^{1}H-^{13}C HSQC ($CDCl_3$, 253 K)

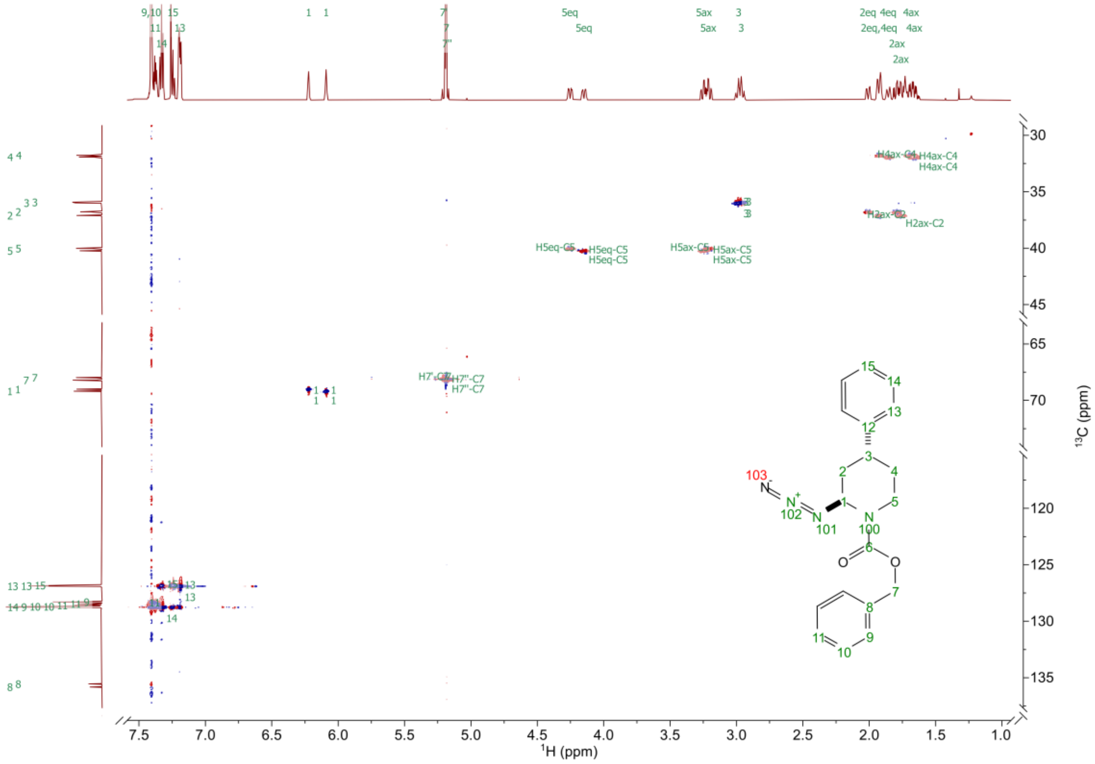

Compound **9i**: ^{1}H-^{13}C HMBC (CDCl$_3$, 253 K)

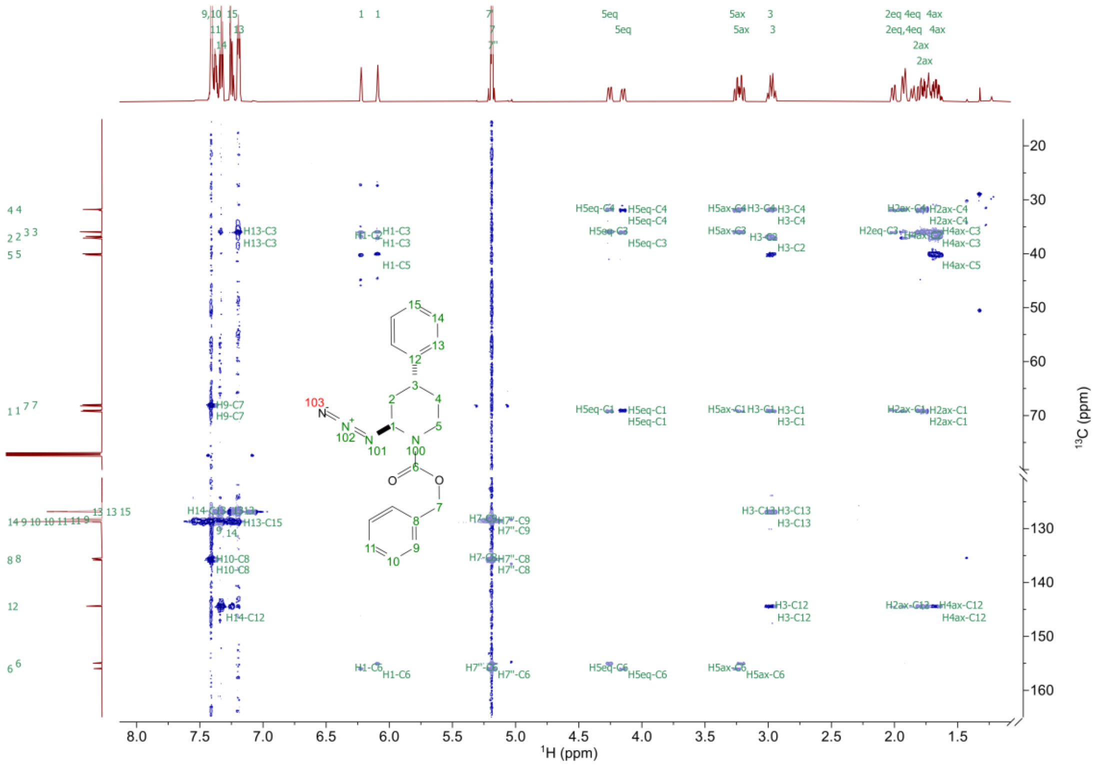

Compound **9i**: ^{1}H-^{1}H COSY (CDCl$_3$, 253 K)

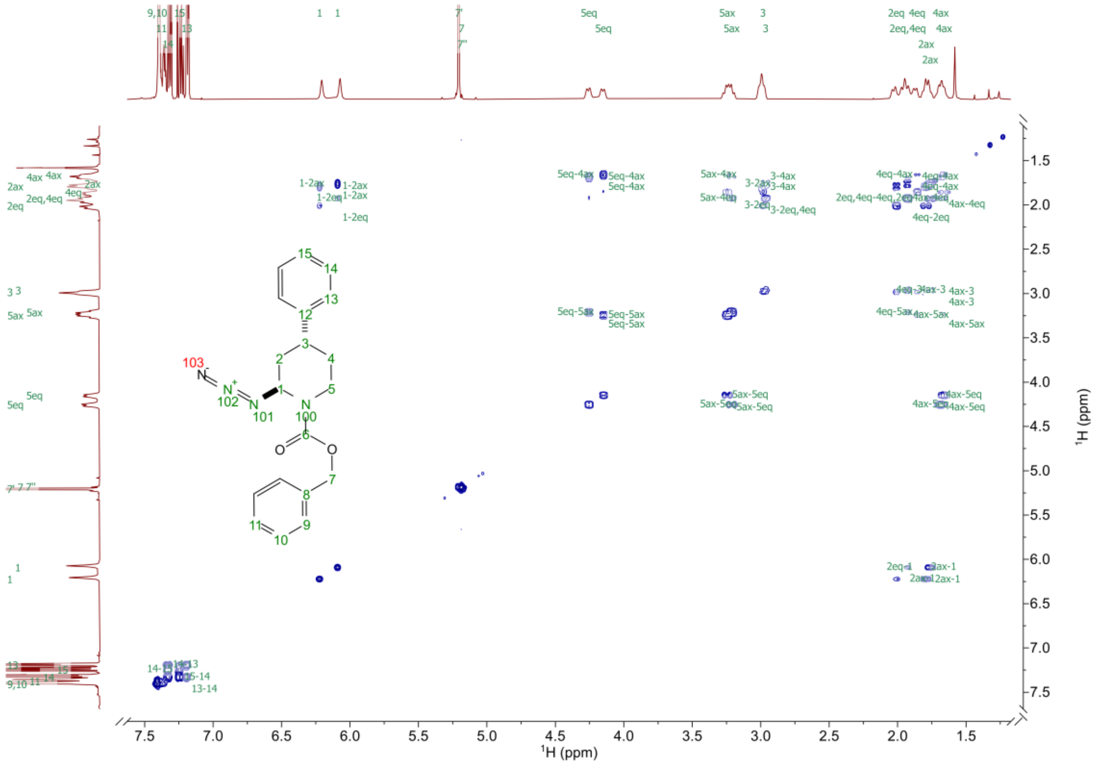

Compound **9i**: ^{1}H-^{1}H NOESY ($CDCl_3$, 253 K)

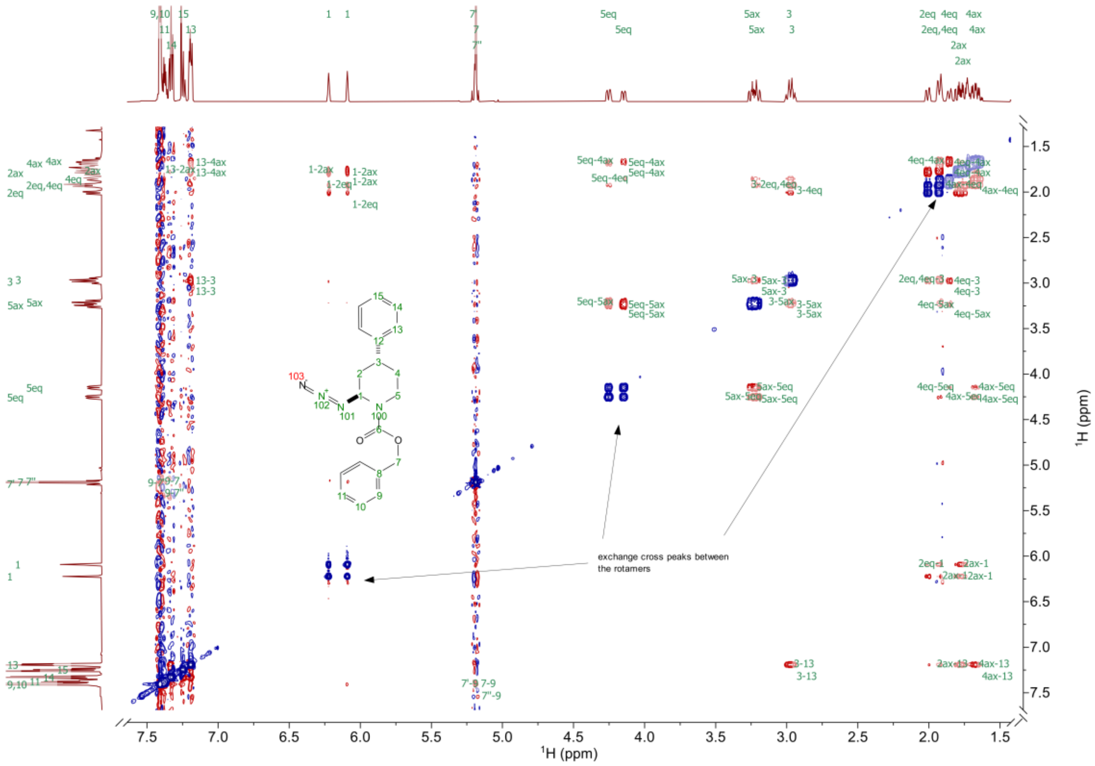

Compound **9i**: ^{1}H-^{15}N HMBC ($CDCl_3$, 253 K)

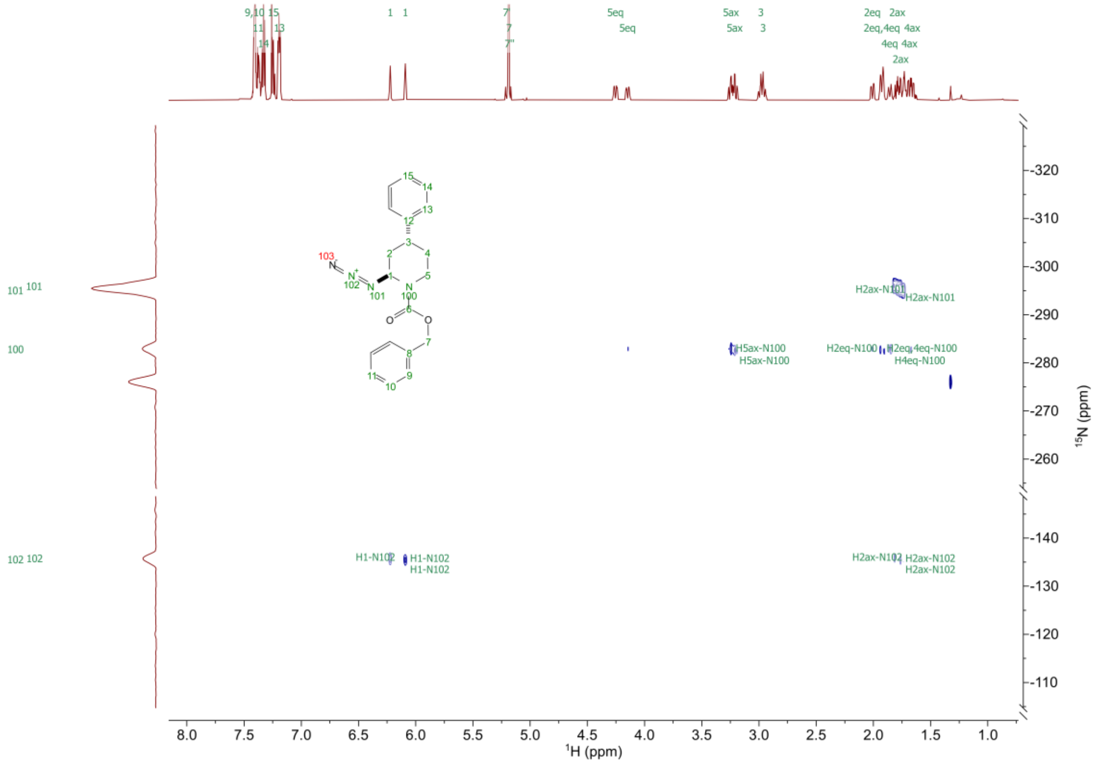

Compound **9i**: 1D selective TOCSY from excitation of H1

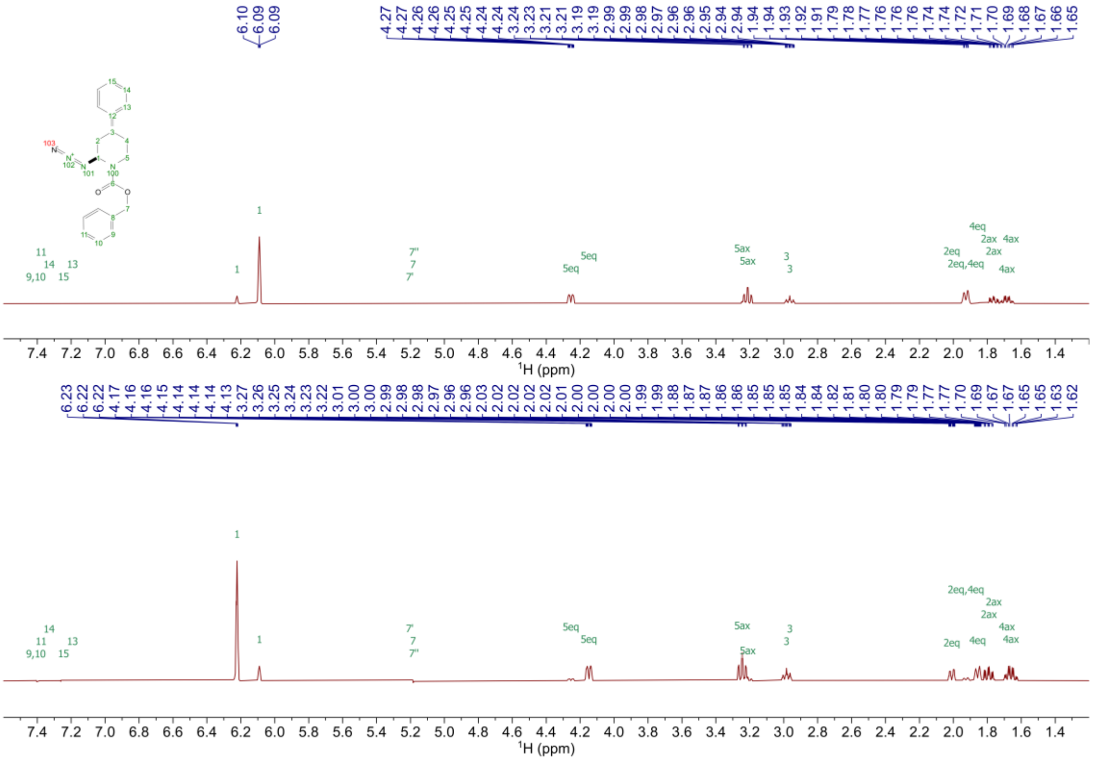

Compound **9j**: ^{1}H NMR (600 MHz, $CDCl_3$, 233 K)

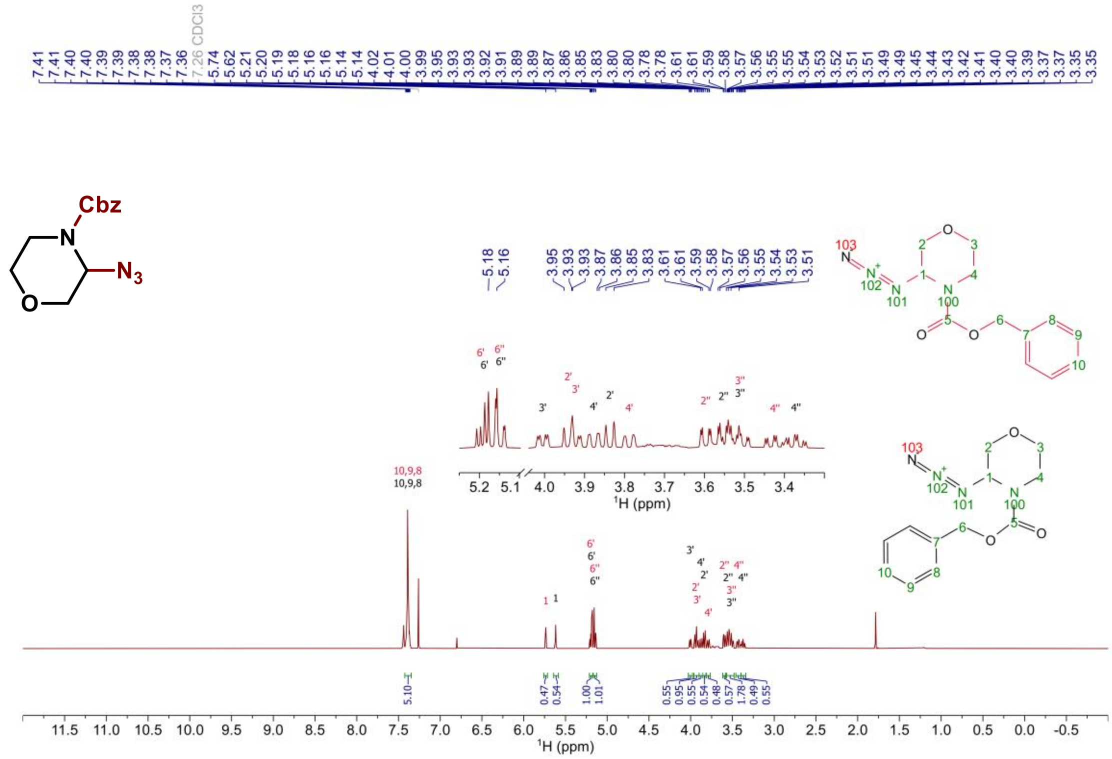

Compound **9j**: ^{13}C NMR (151 MHz, $CDCl_3$, 233 K)

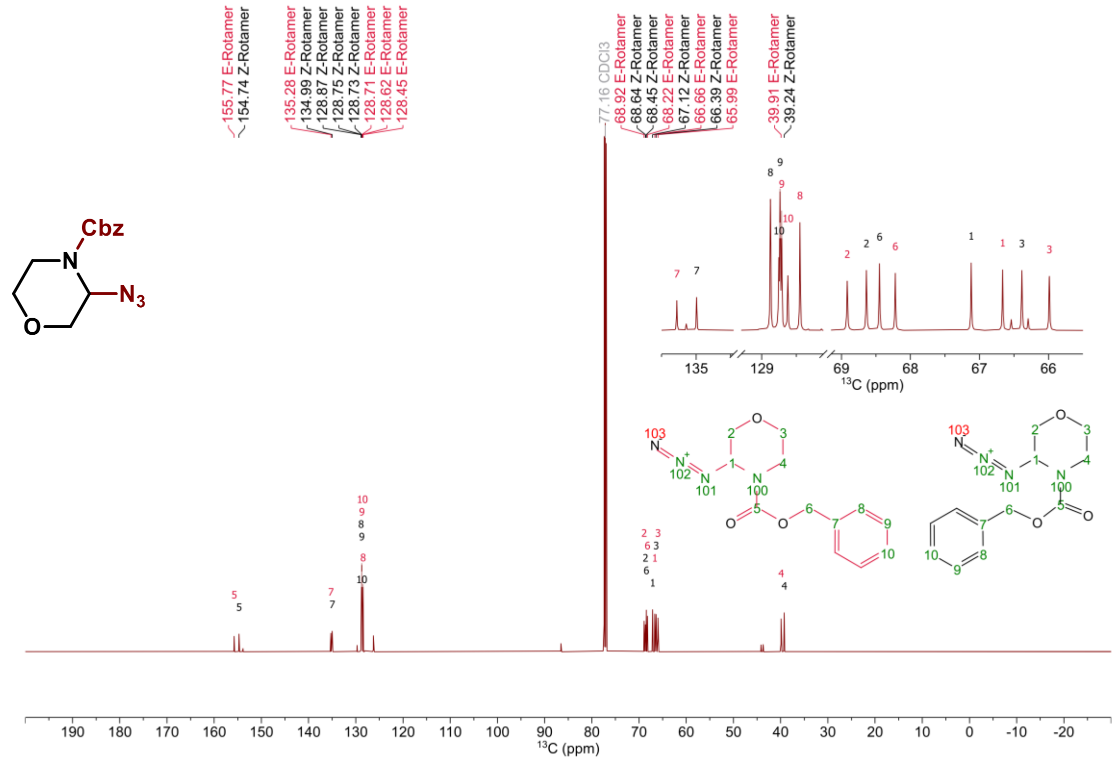

Compound **9j**: ^{1}H-^{13}C HSQC ($CDCl_3$, 233 K)

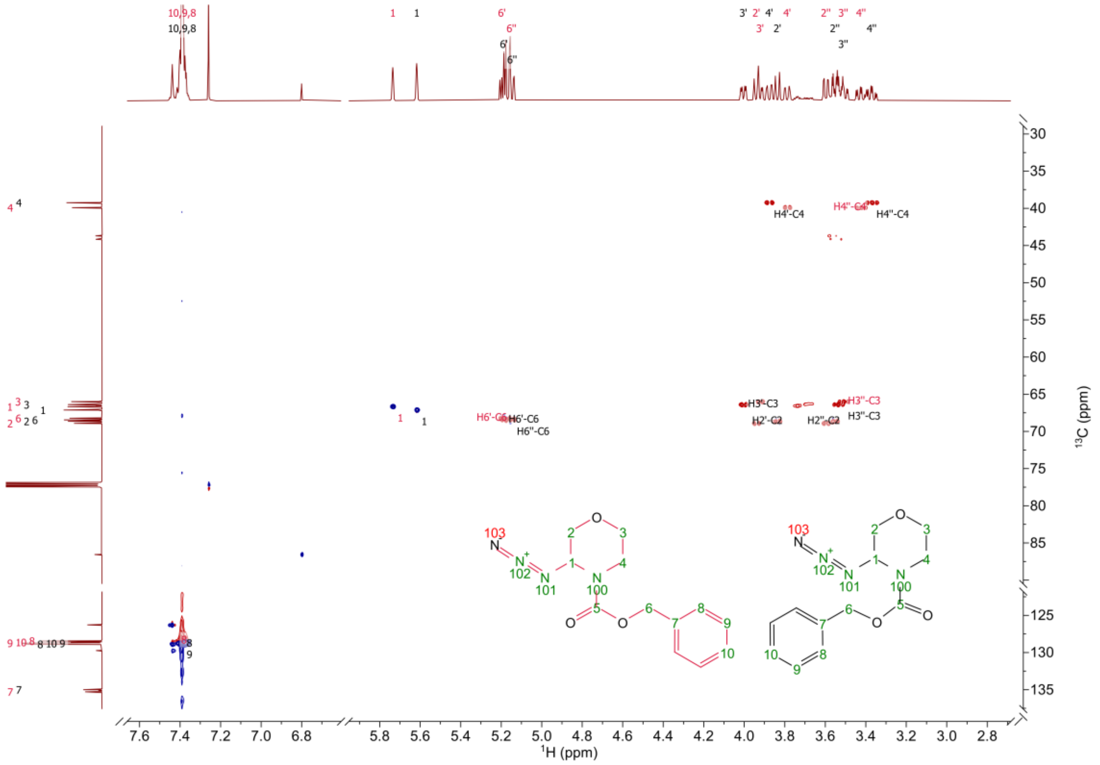

Compound **9j**: ^1H-^{13}C HMBC ($CDCl_3$, 233 K)

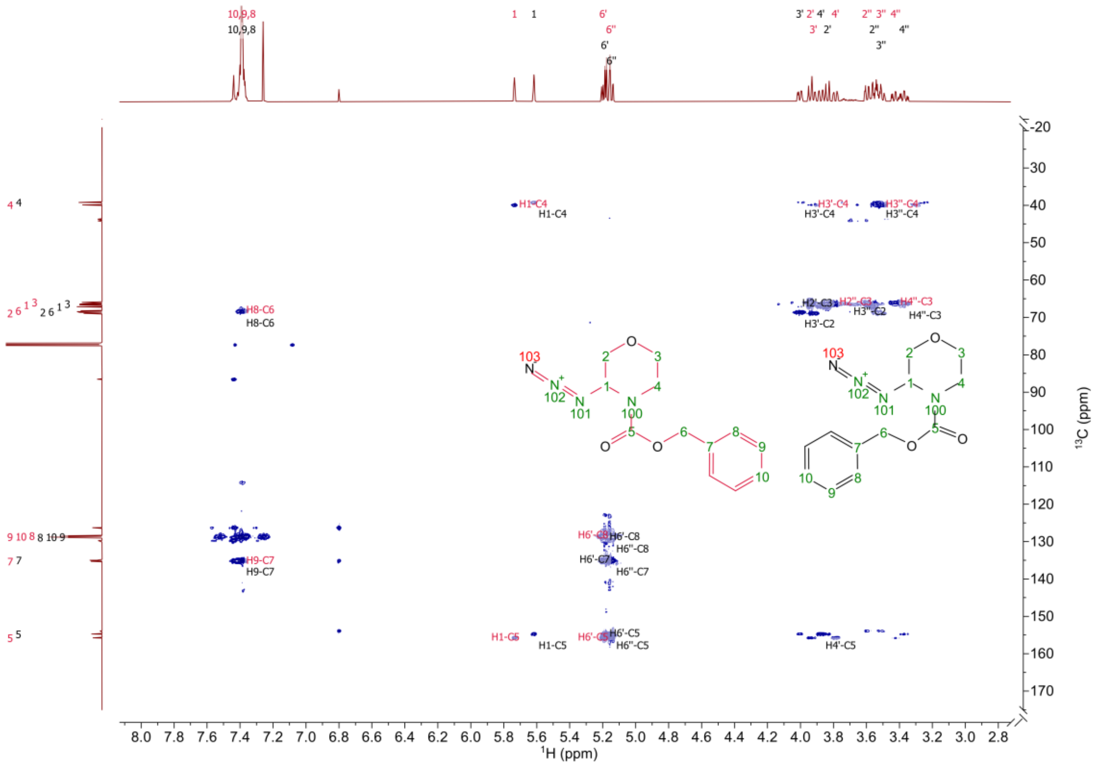

Compound **9j**: ^1H-^1H COSY ($CDCl_3$, 233 K)

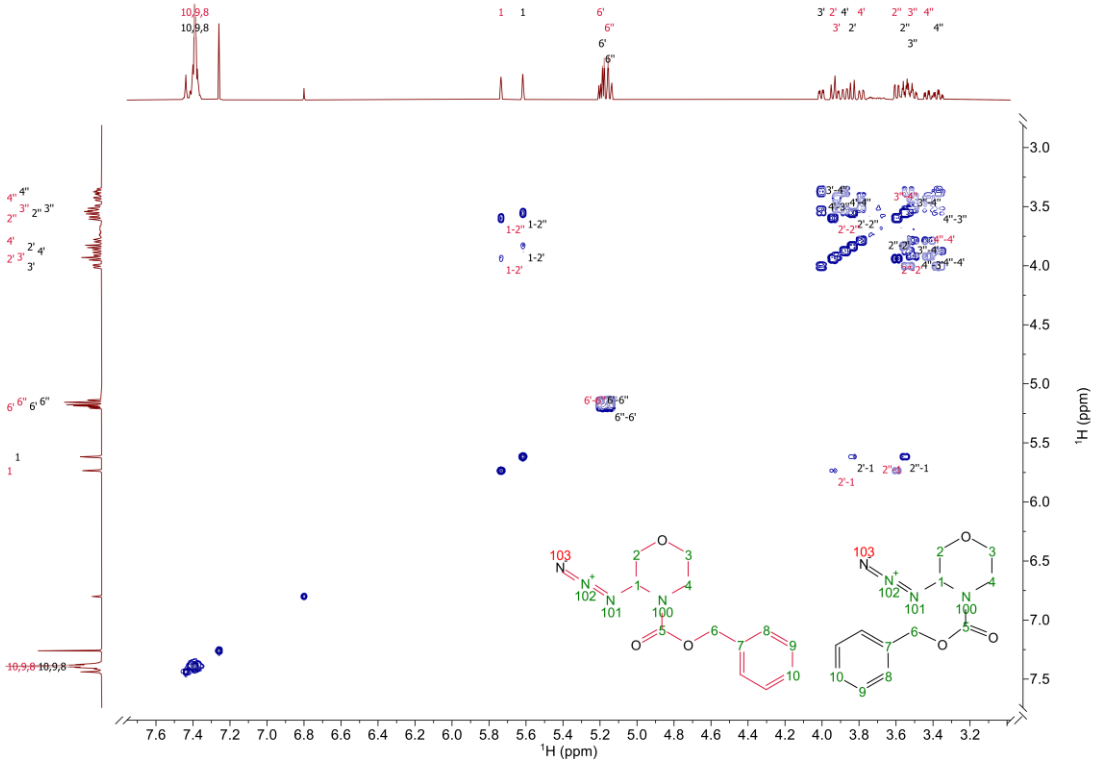

Compound **9j**: ^{1}H-^{1}H NOESY ($CDCl_3$, 233 K)

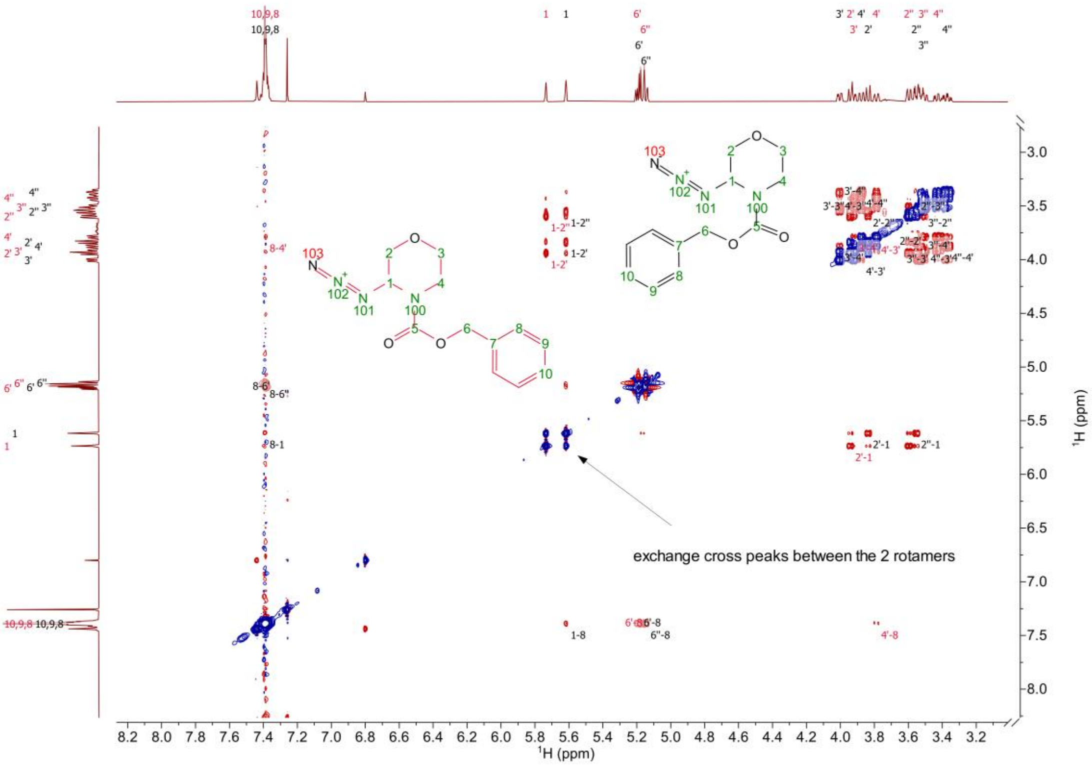

Compound **9j**: ^{1}H-^{15}N HMBC ($CDCl_3$, 233 K)

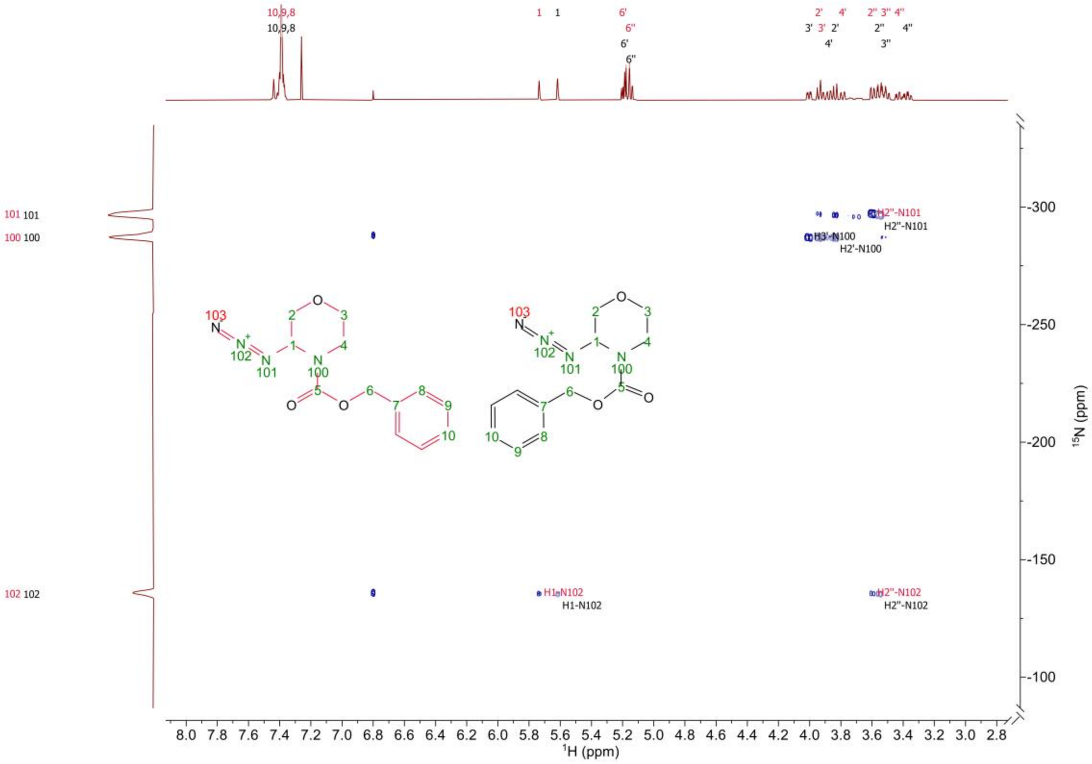

Compound **9j**: 1D selective TOCSY from excitation of H1

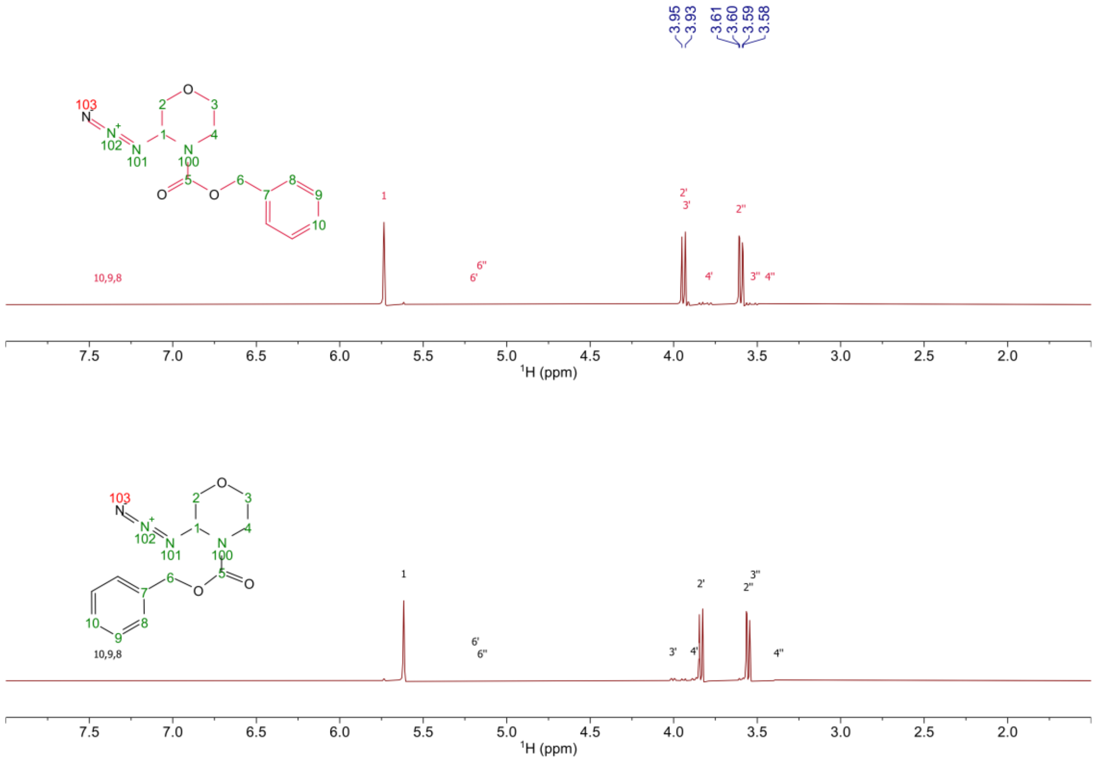

Compound **9k**: ^{1}H NMR (600 MHz, $CDCl_3$, 298 K)

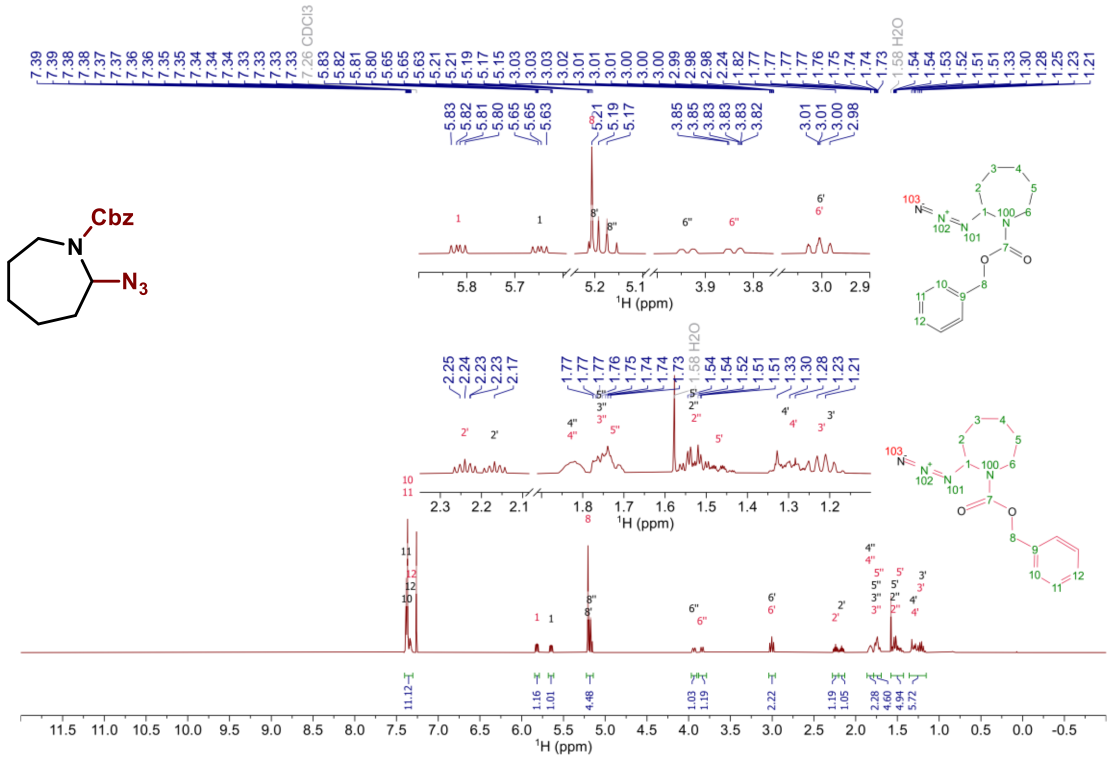

Compound **9k**: ^{13}C NMR (151 MHz, $CDCl_3$, 298 K)

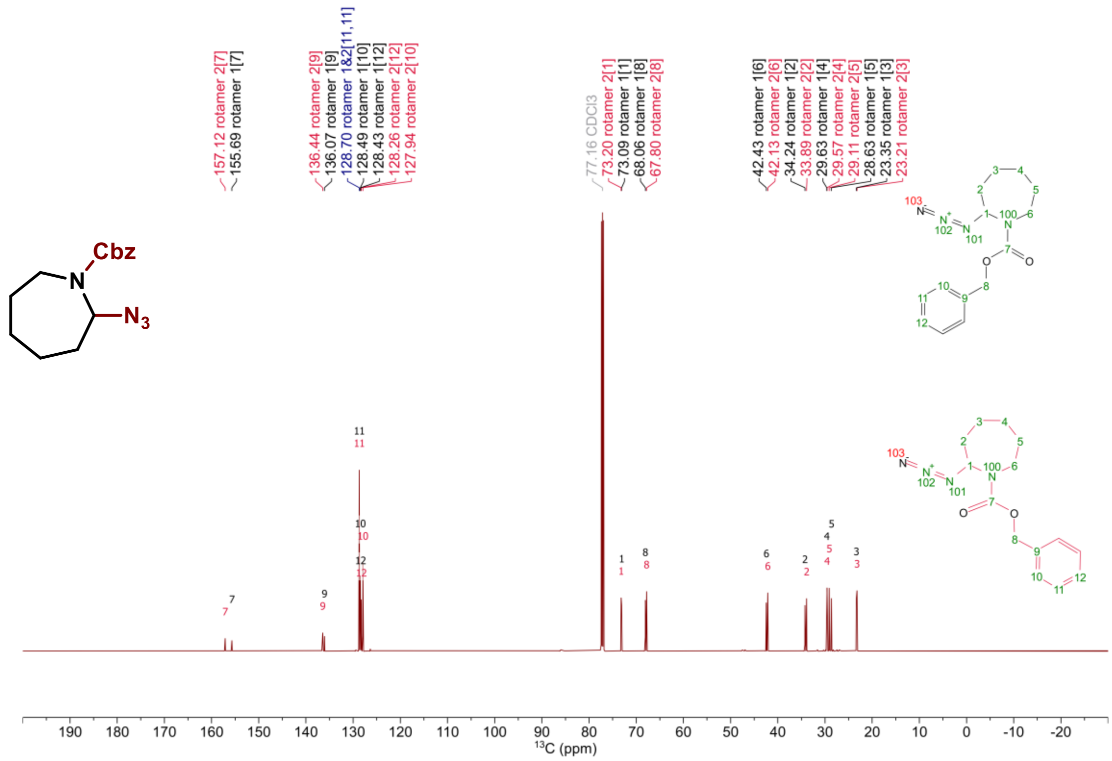

Compound **9k**: ^{1}H-^{13}C HSQC ($CDCl_3$, 298 K)

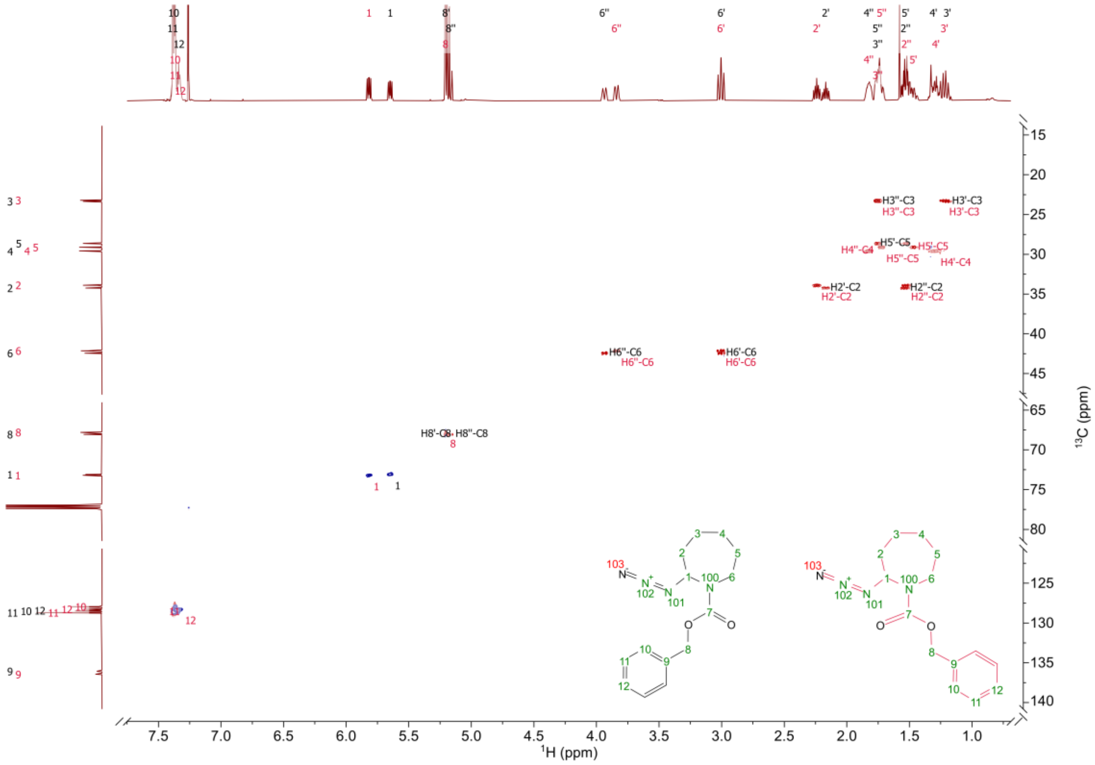

Compound **9k**: ^{1}H-^{13}C HMBC (CDCl$_3$, 298 K)

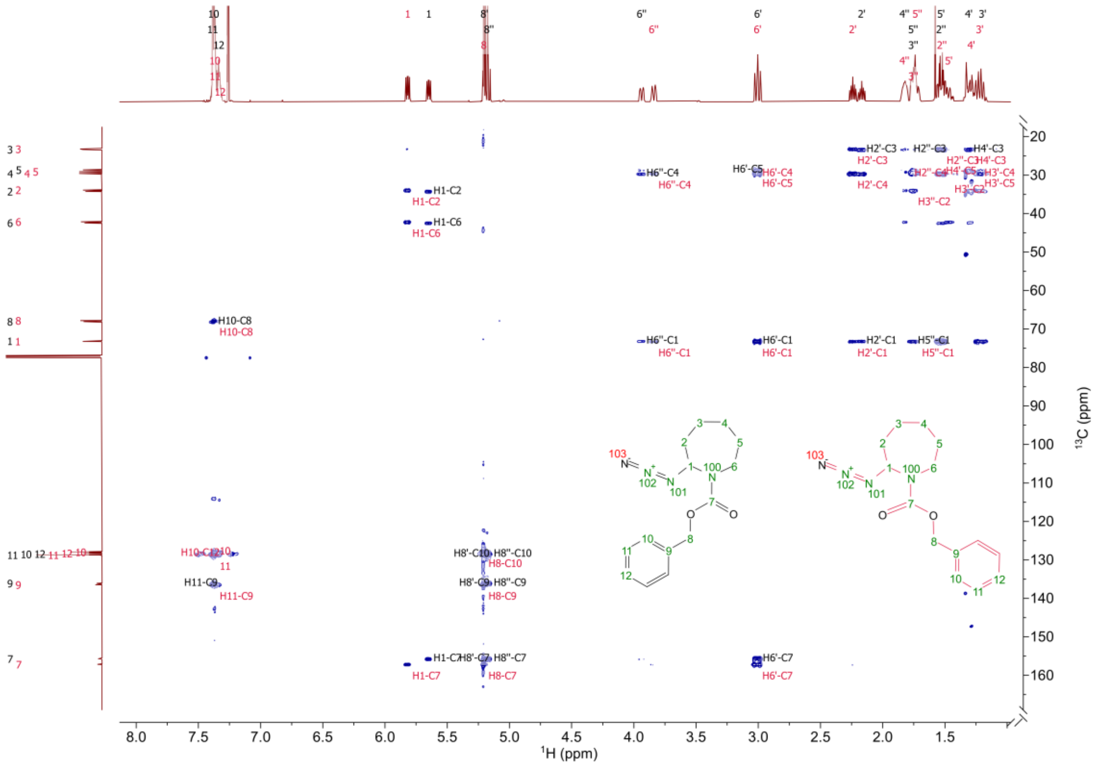

Compound **9k**: ^{1}H-^{1}H COSY (CDCl$_3$, 298 K)

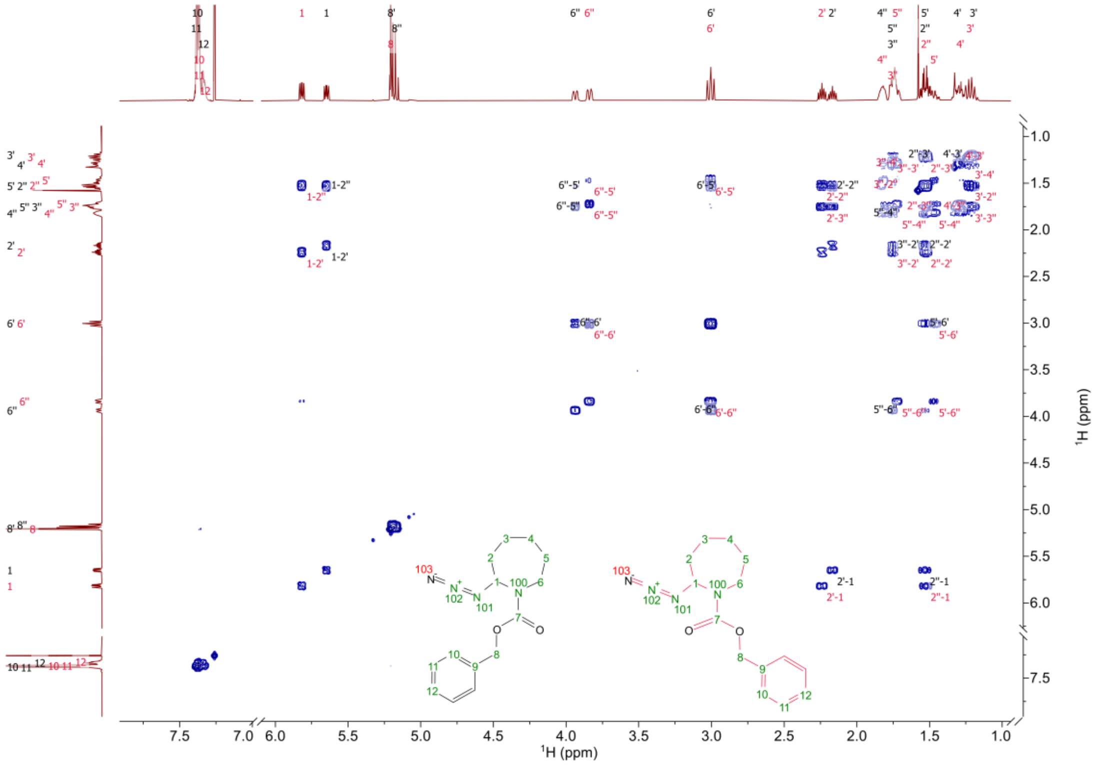

Compound **9k**: ^{1}H-^{1}H NOESY ($CDCl_3$, 298 K)

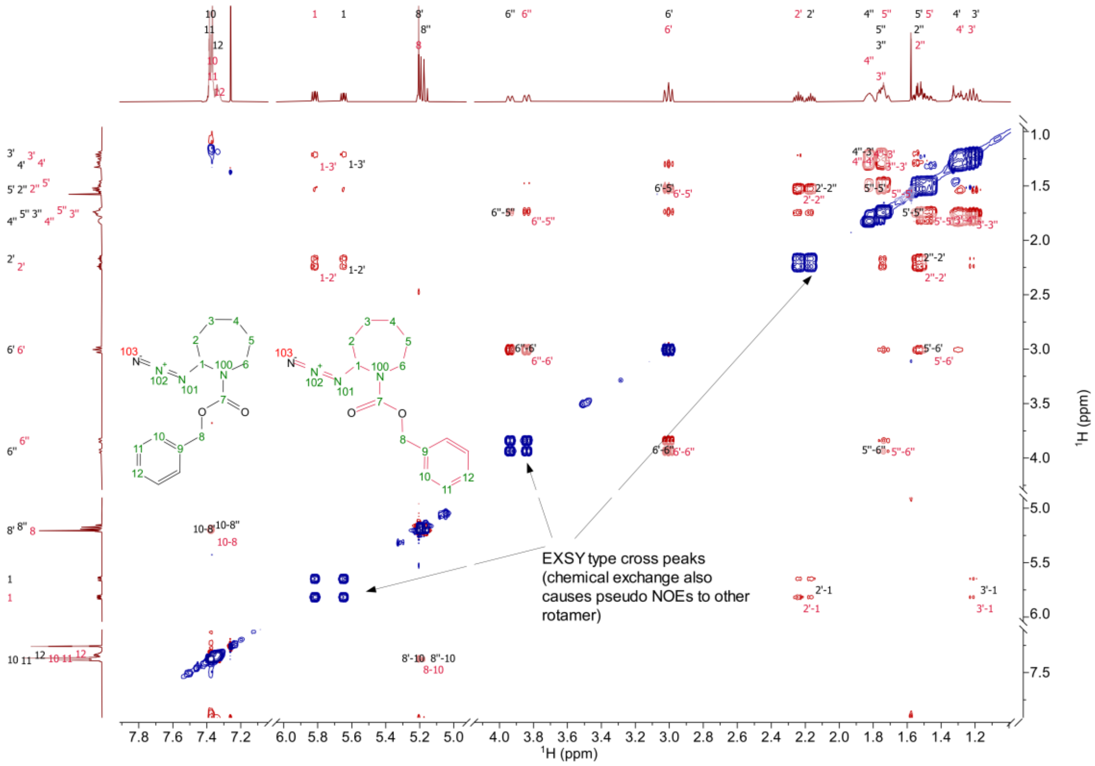

Compound **9k**: ^{1}H-^{15}N HMBC ($CDCl_3$, 298 K)

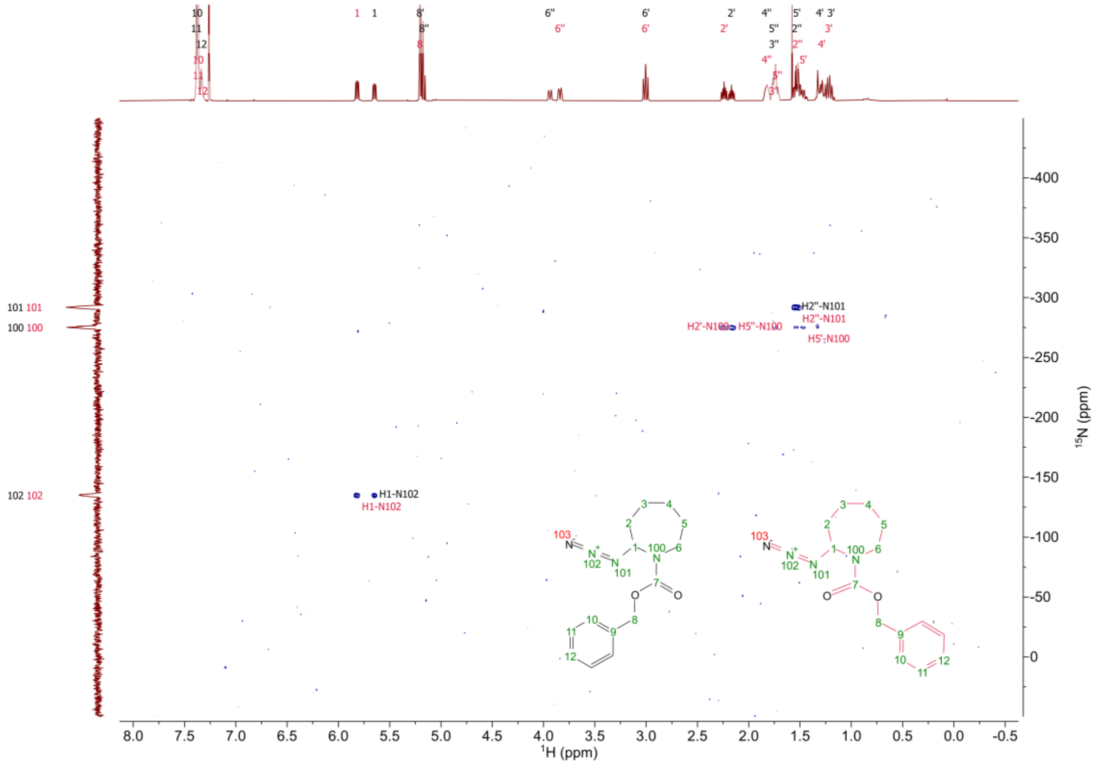

Compound **9l**, major regioisomer: ^{1}H NMR (600 MHz, $CDCl_3$, 273 K)

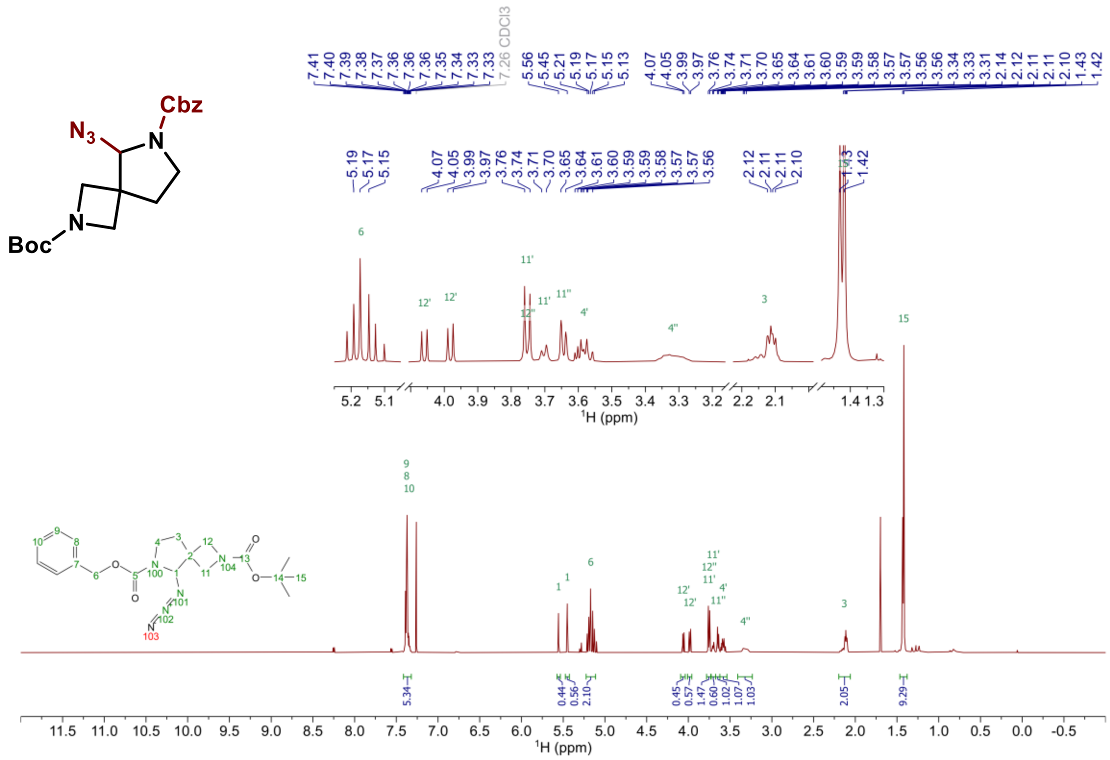

Compound **9l**, major regioisomer: ^{13}C NMR (151 MHz, $CDCl_3$, 273 K)

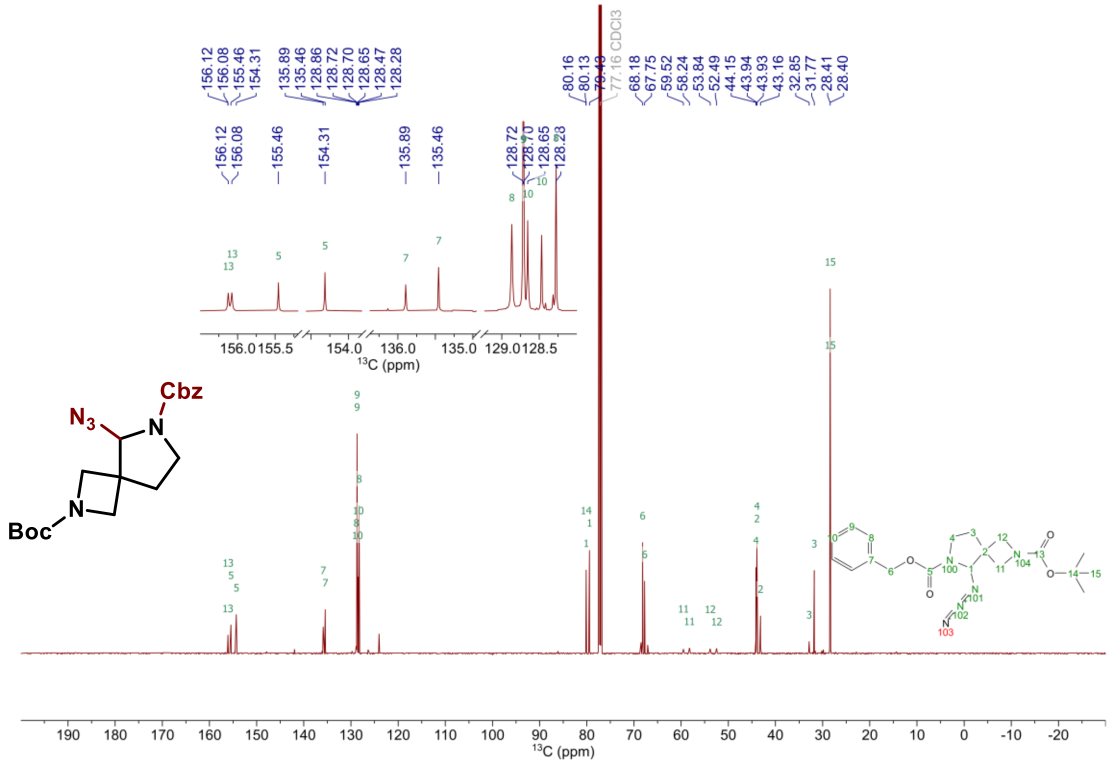

Compound **9l**, major regioisomer: variable temperature ^{1}H NMR (600 MHz, $CDCl_3$)

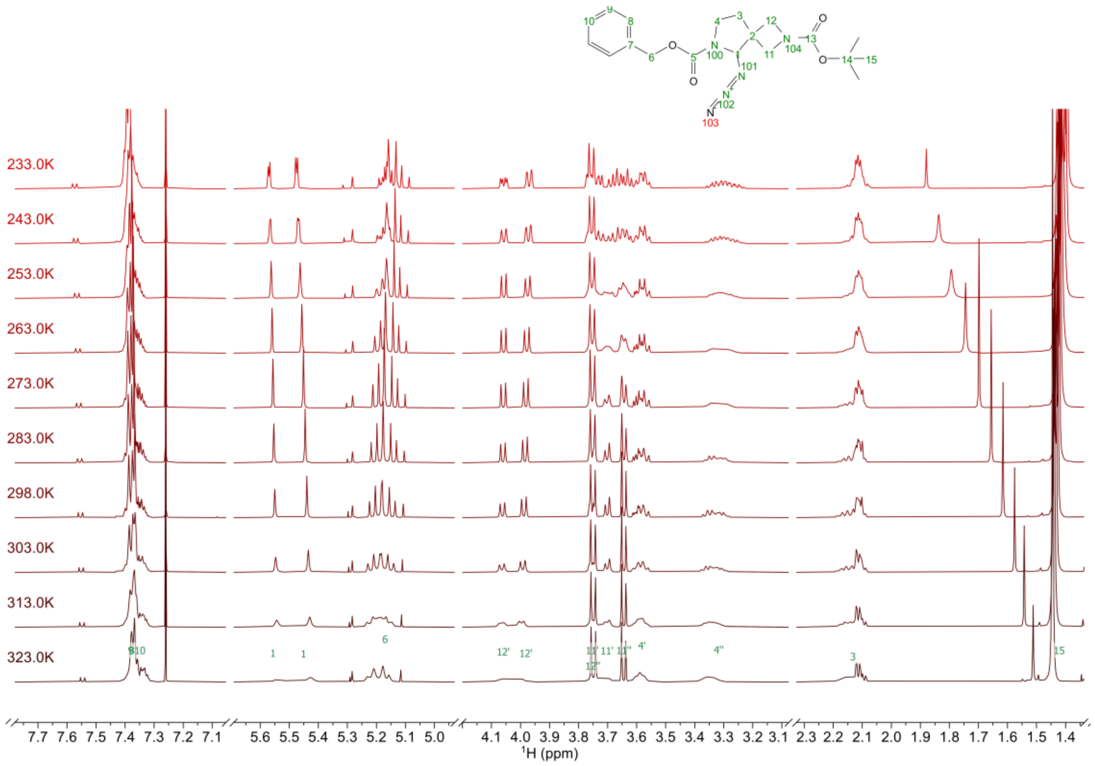

Compound **9l**, major regioisomer: ^{1}H-^{13}C HSQC ($CDCl_3$, 273 K)

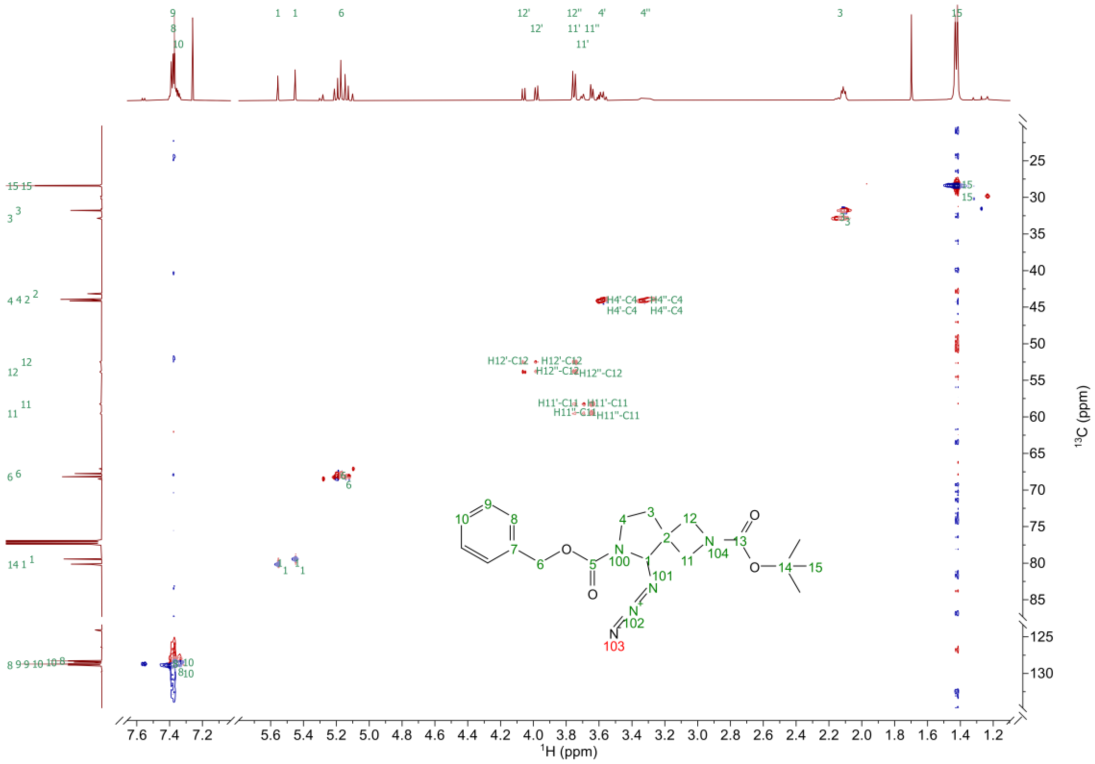

Compound **9l**, major regioisomer: ^{1}H-^{13}C HMBC ($CDCl_3$, 273 K)

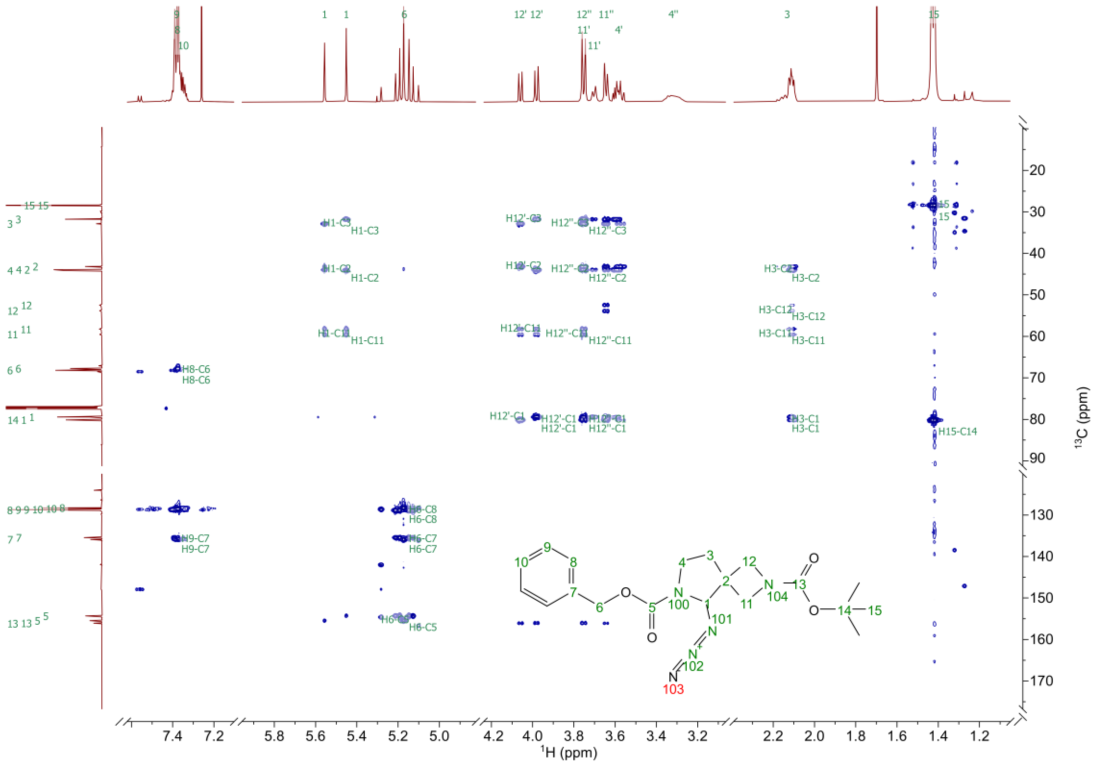

Compound **9l**, major regioisomer: ^{1}H-^{1}H COSY ($CDCl_3$, 273 K)

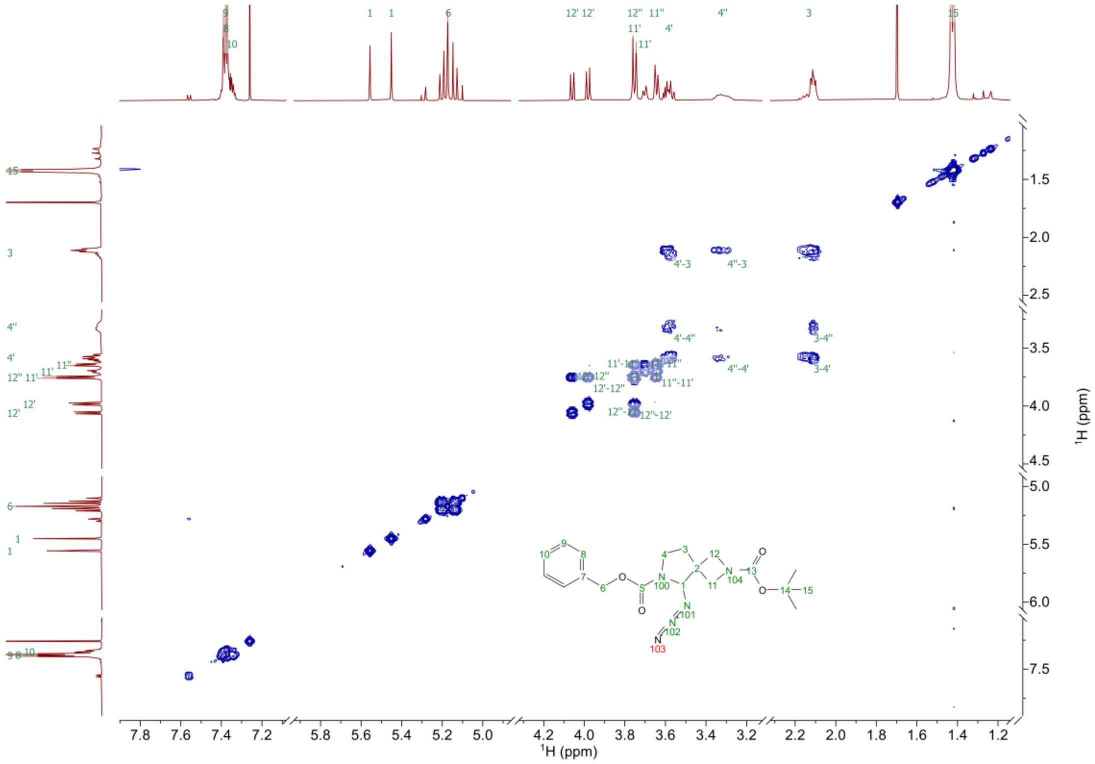

Compound **9l**, major regioisomer: ^{1}H-^{1}H NOESY ($CDCl_3$, 273 K)

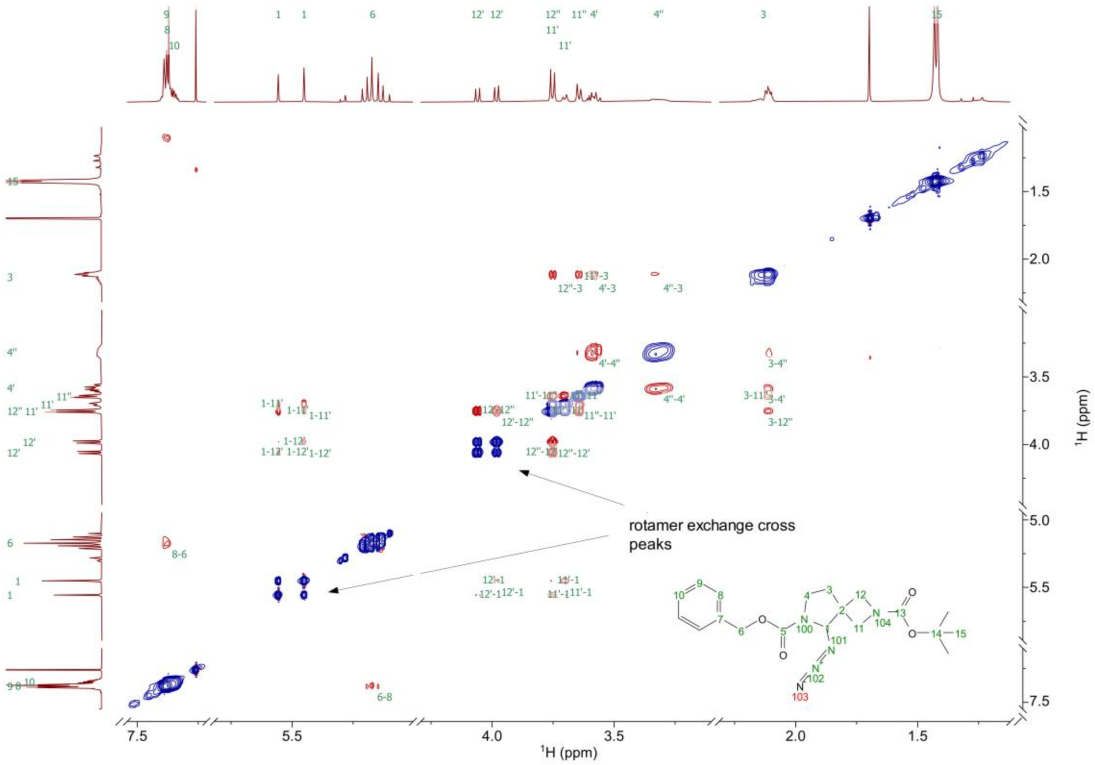

Compound **9l**, major regioisomer: ^{1}H-^{15}N HMBC ($CDCl_3$, 273 K)

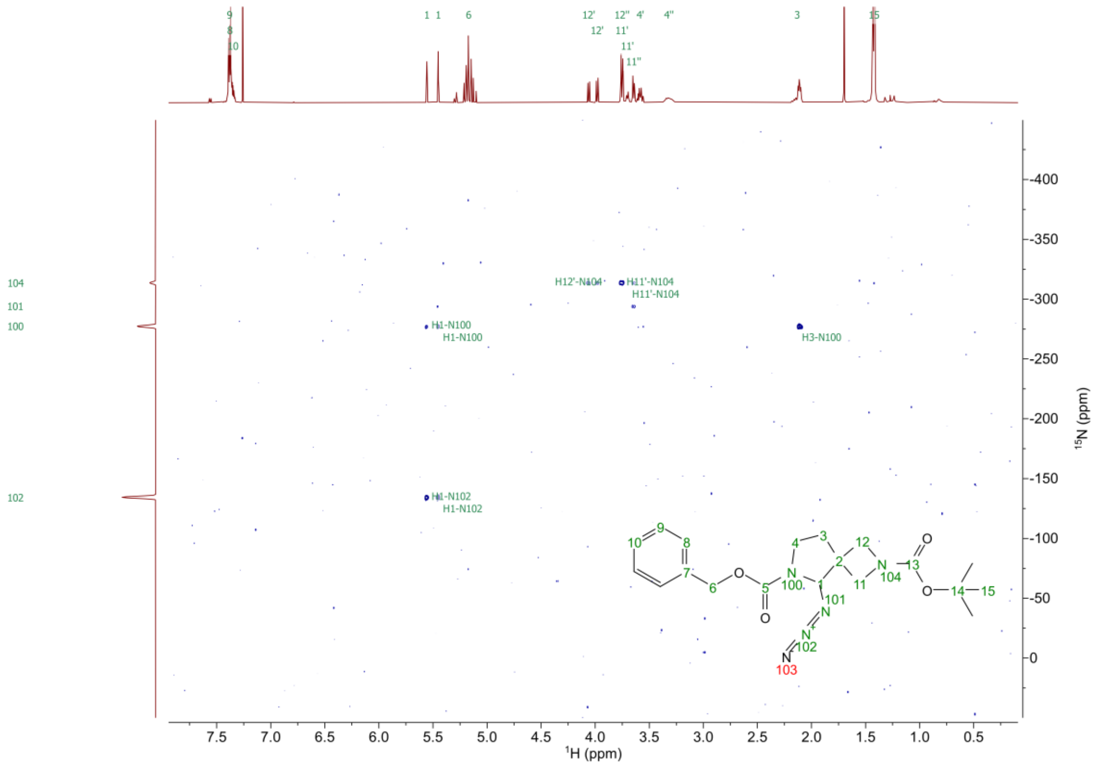

Compound **9l**, minor regioisomer: ^{1}H NMR (600 MHz, $CDCl_3$, 298 K)

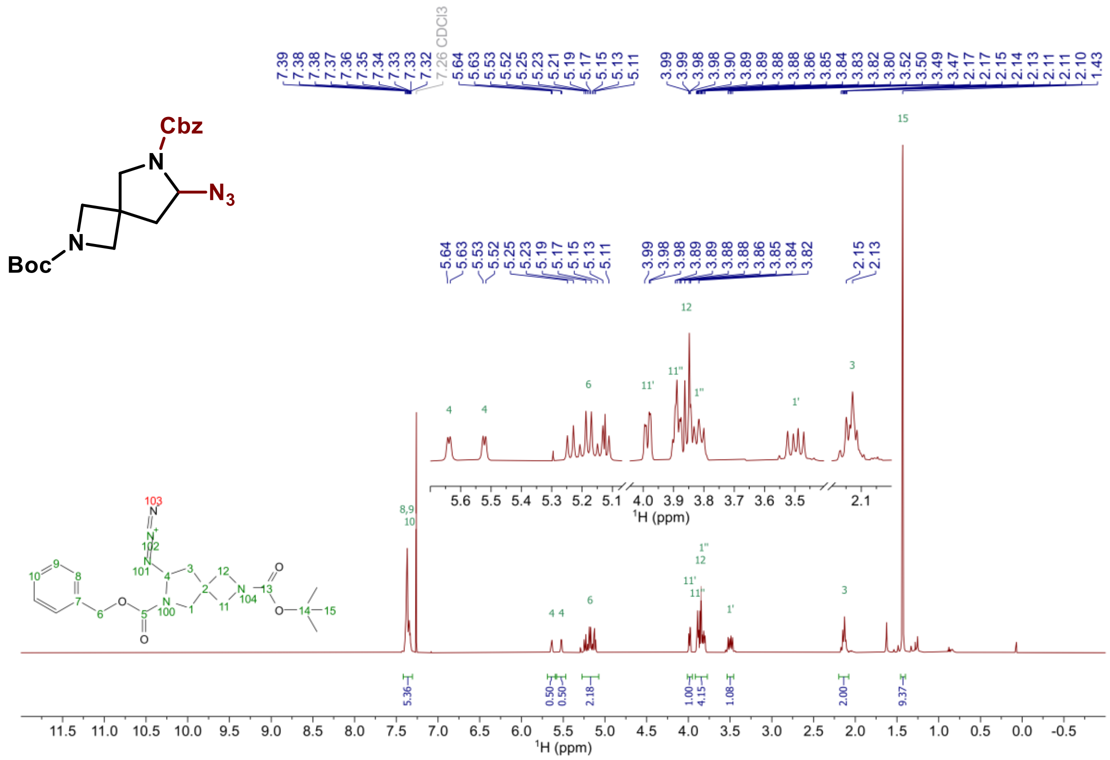

Compound **9l**, minor regioisomer: ^{13}C NMR (151 MHz, $CDCl_3$, 298 K)

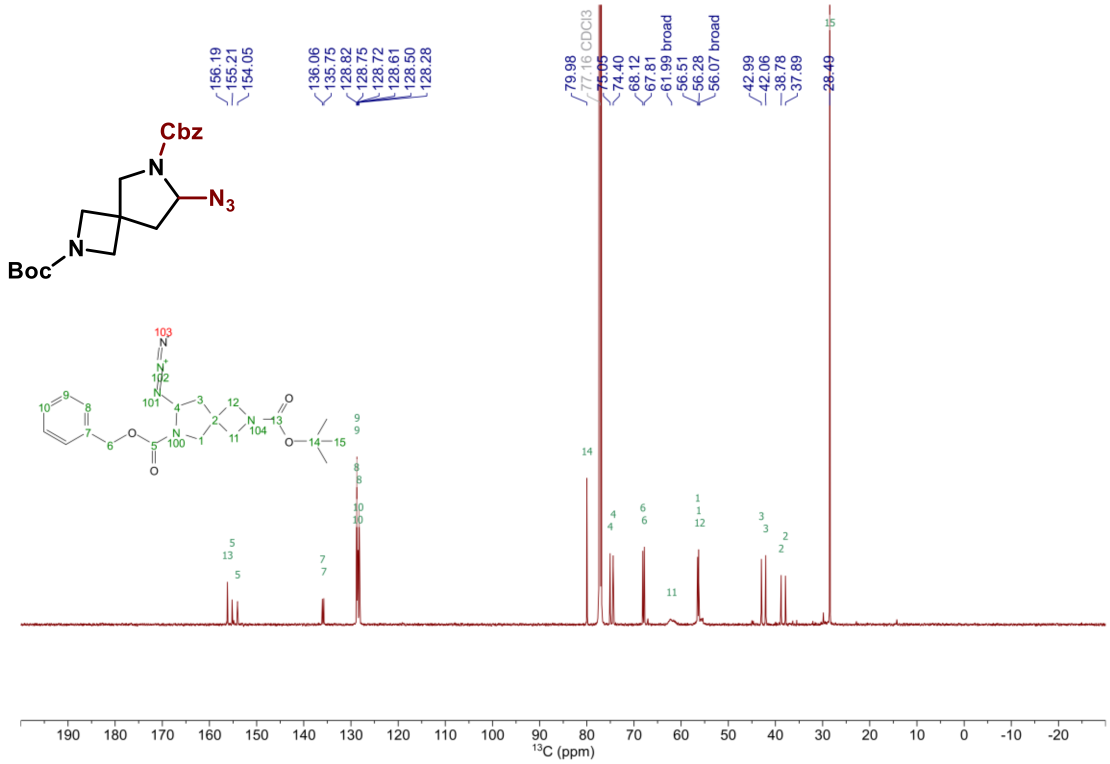

Compound **9l**, minor regioisomer: ^{1}H-^{13}C HSQC ($CDCl_3$, 298 K)

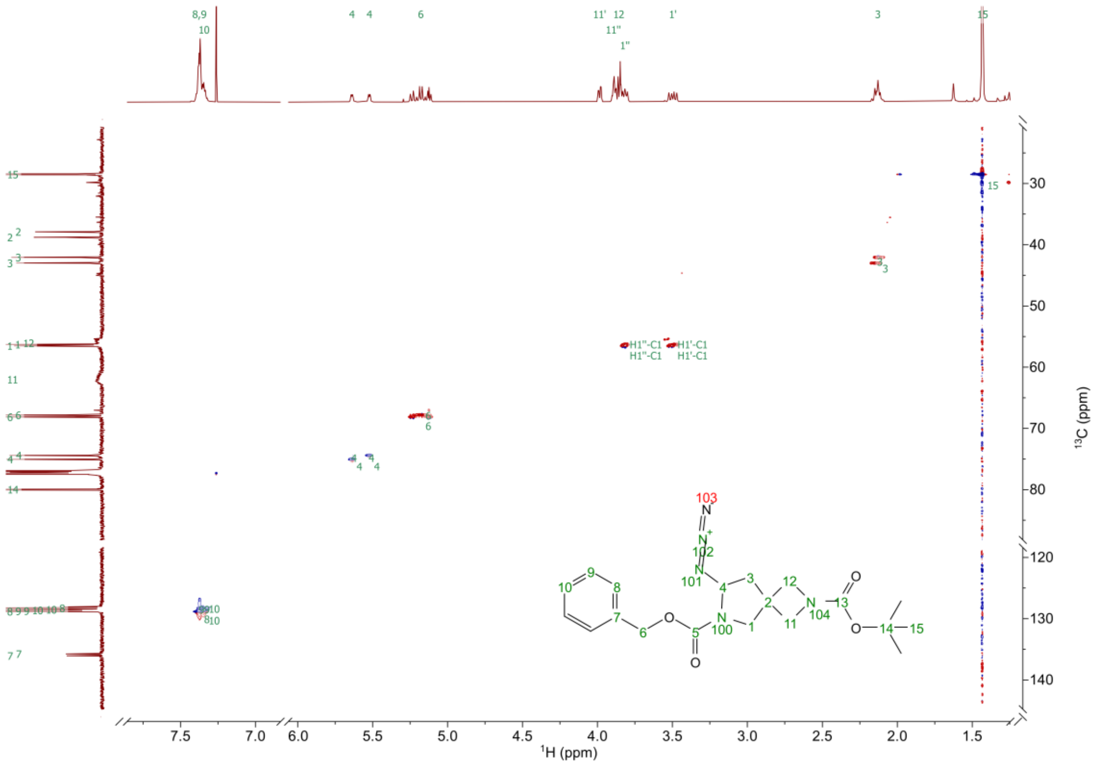

Compound **9l**, minor regioisomer: ^{1}H-^{13}C HMBC ($CDCl_3$, 298 K)

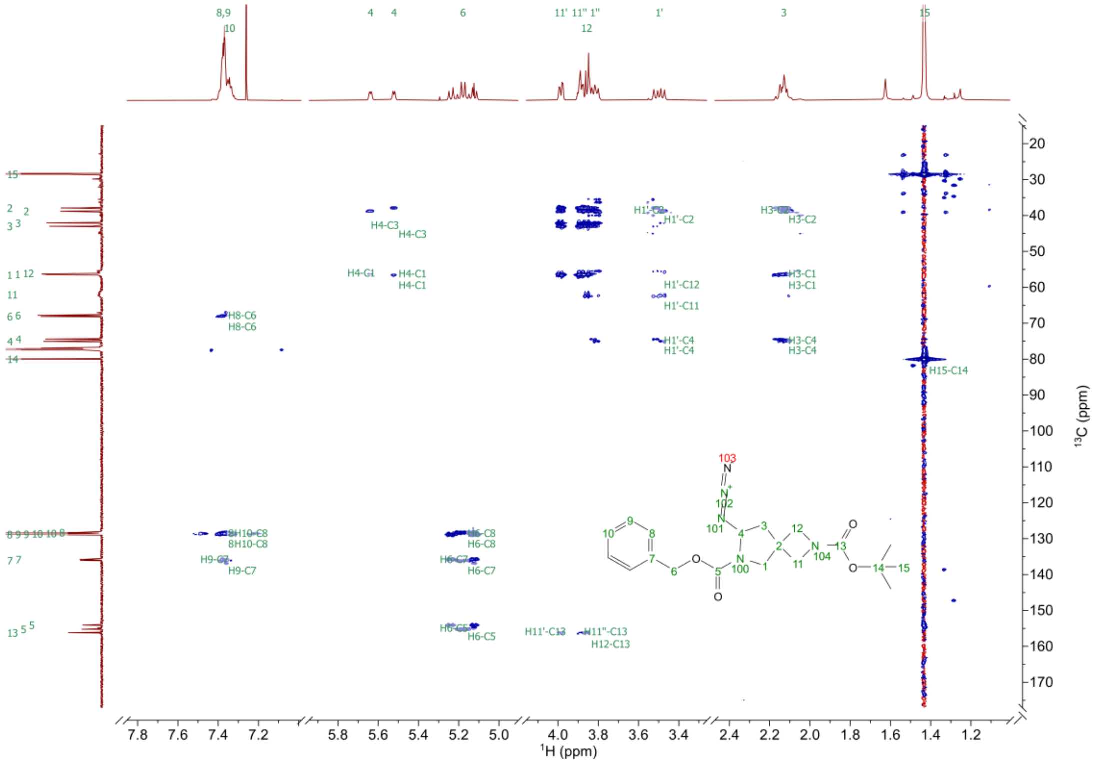

Compound **9l**, minor regioisomer: ^1H-^1H COSY ($CDCl_3$, 298 K)

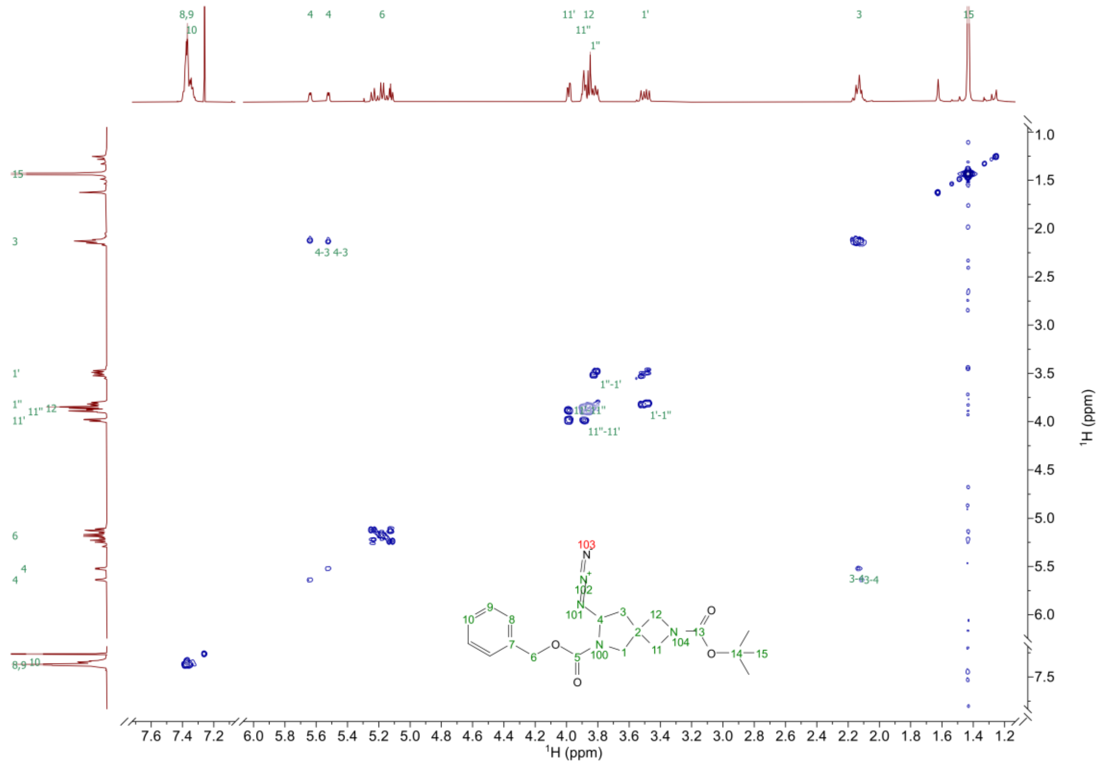

Compound **9l**, minor regioisomer: ^1H-^1H NOESY ($CDCl_3$, 298 K)

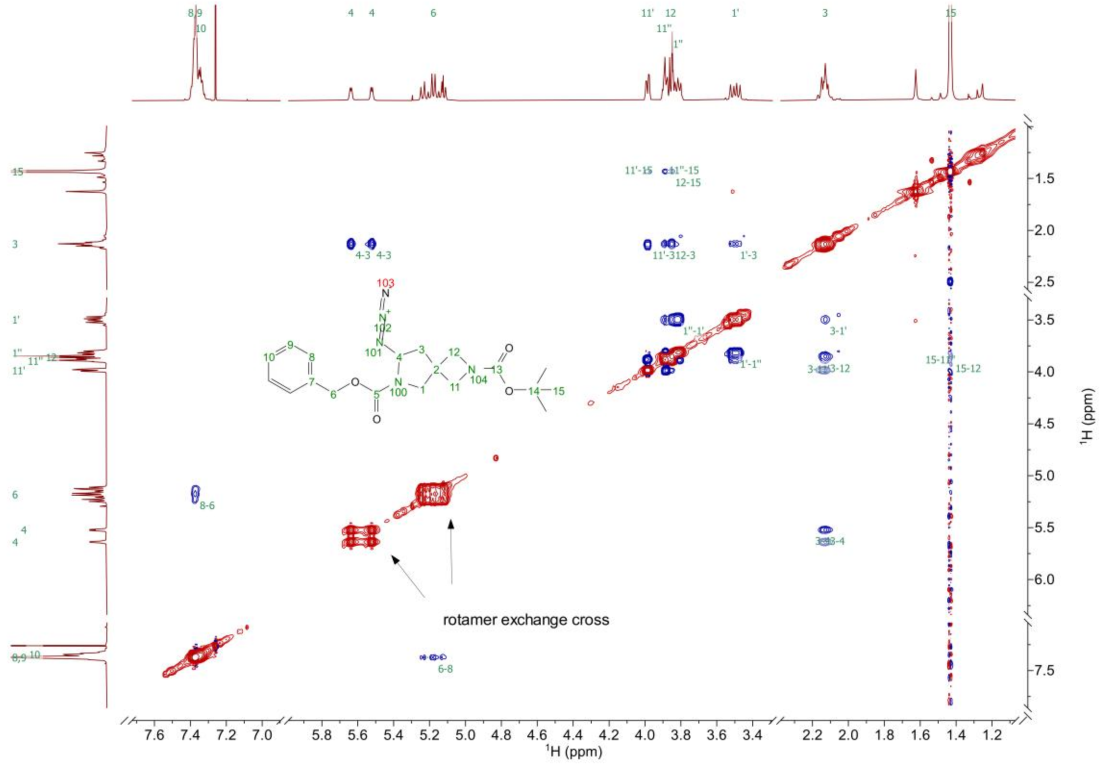

Compound **9l**, minor regioisomer: ^{1}H-^{15}N HMBC ($CDCl_3$, 298 K)

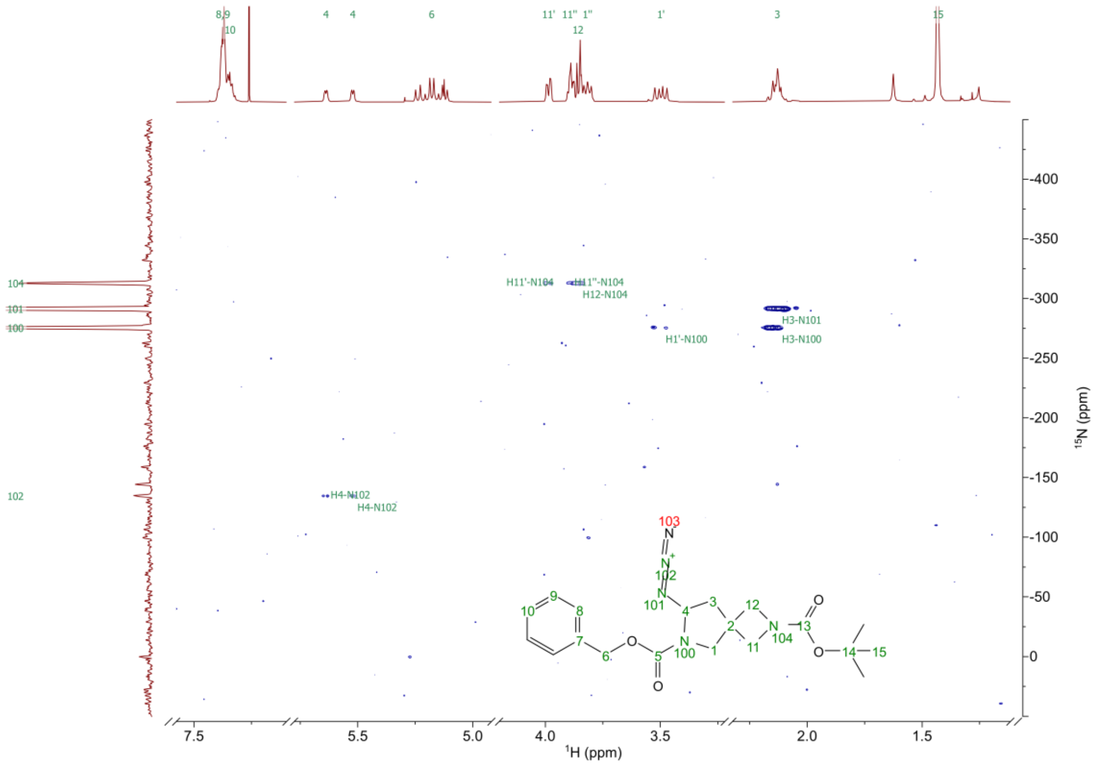

Compound **9m**: ^{1}H NMR (600 MHz, $CDCl_3$, 298 K)

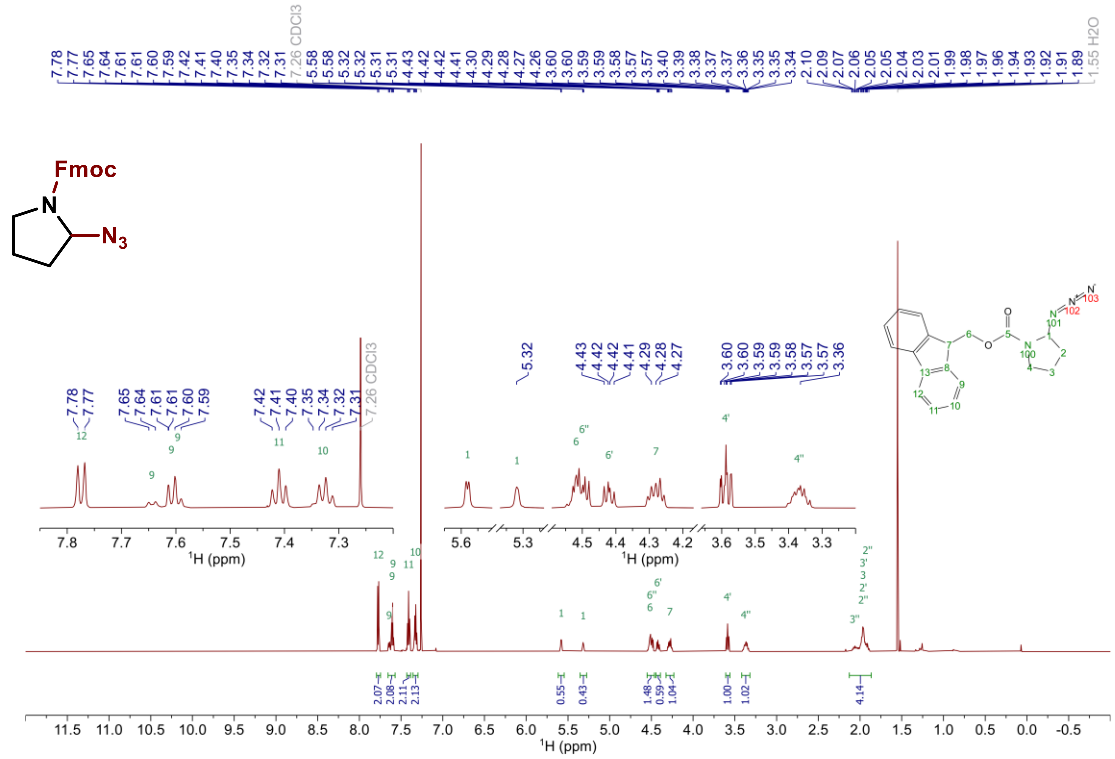

Compound **9m**: ^{13}C NMR (151 MHz, $CDCl_3$, 298 K)

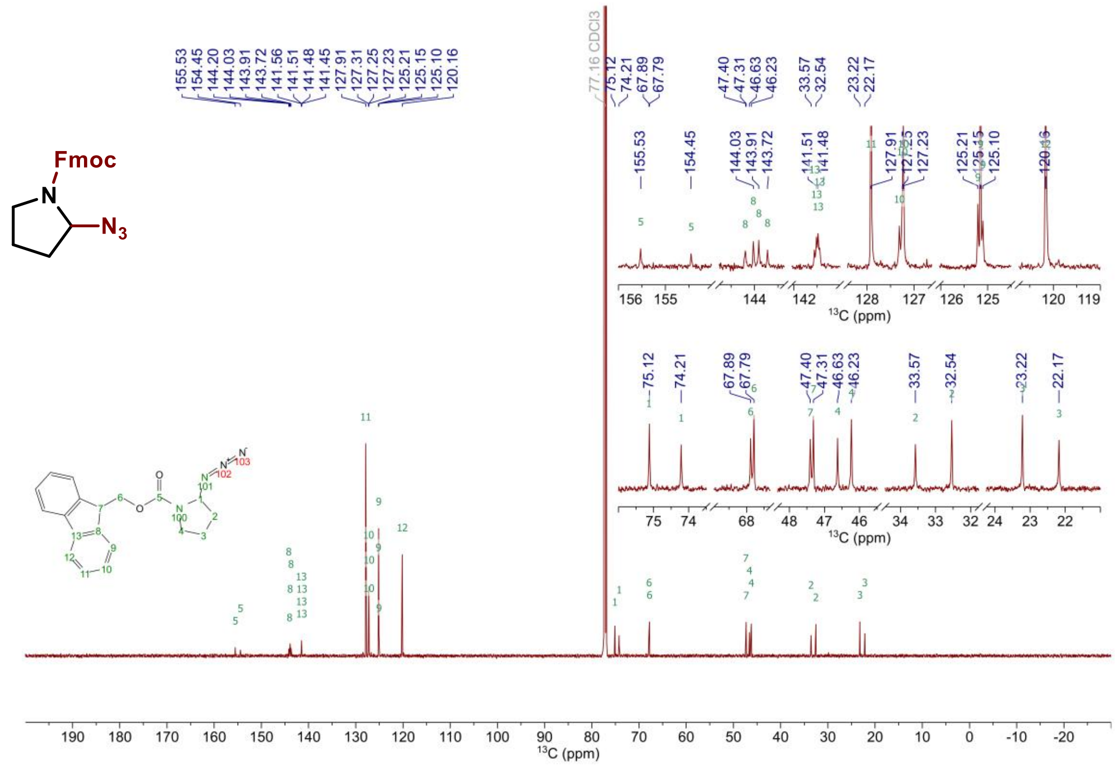

Compound **9m**: ^{1}H-^{13}C HSQC ($CDCl_3$, 298 K)

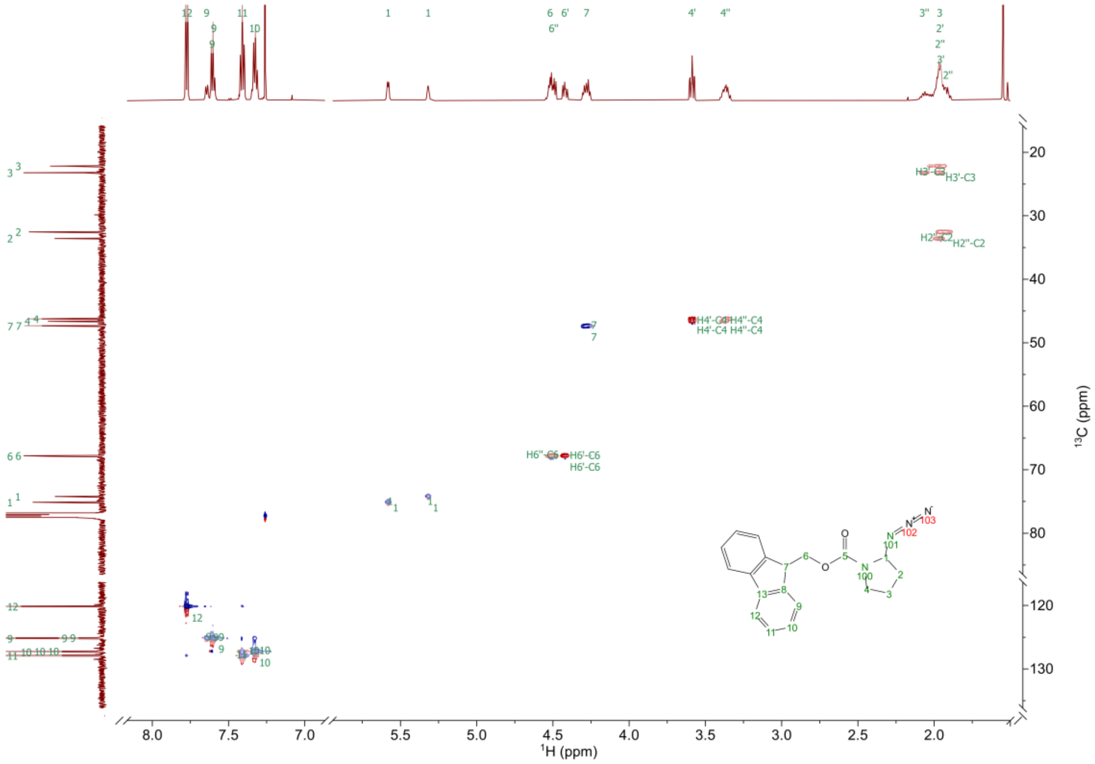

Compound **9m**: ^{1}H-^{13}C HMBC (CDCl$_3$, 298 K)

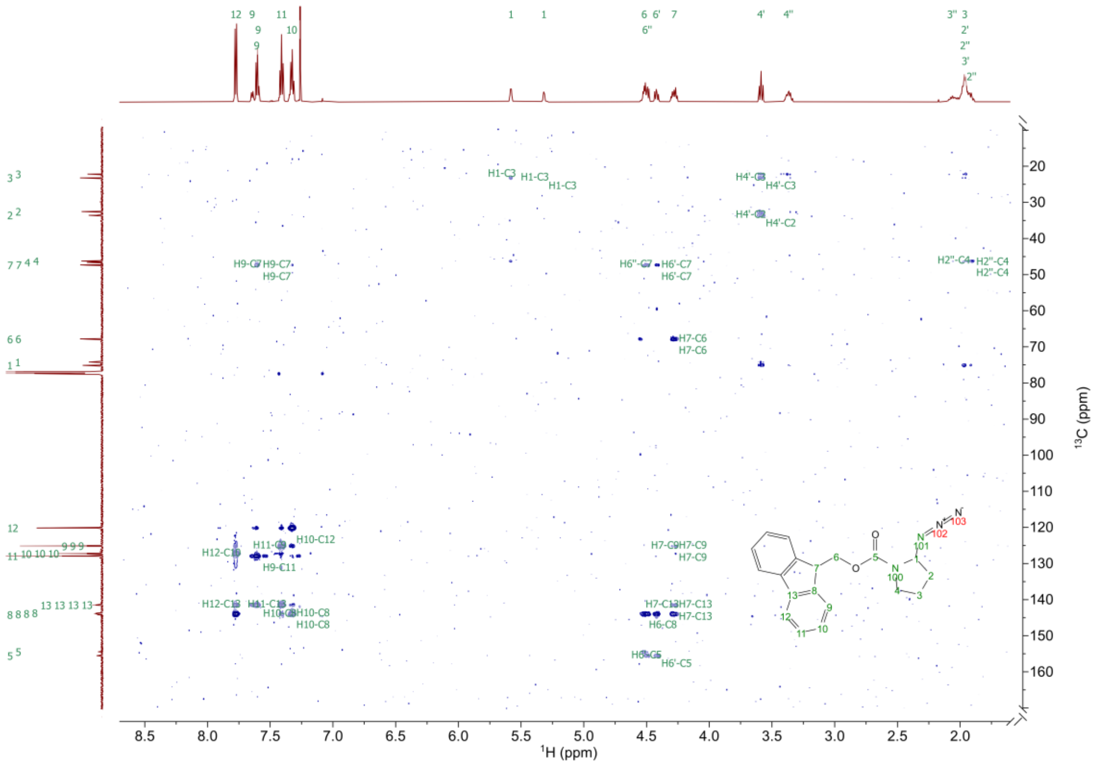

Compound **9m**: ^{1}H-^{1}H COSY (CDCl$_3$, 298 K)

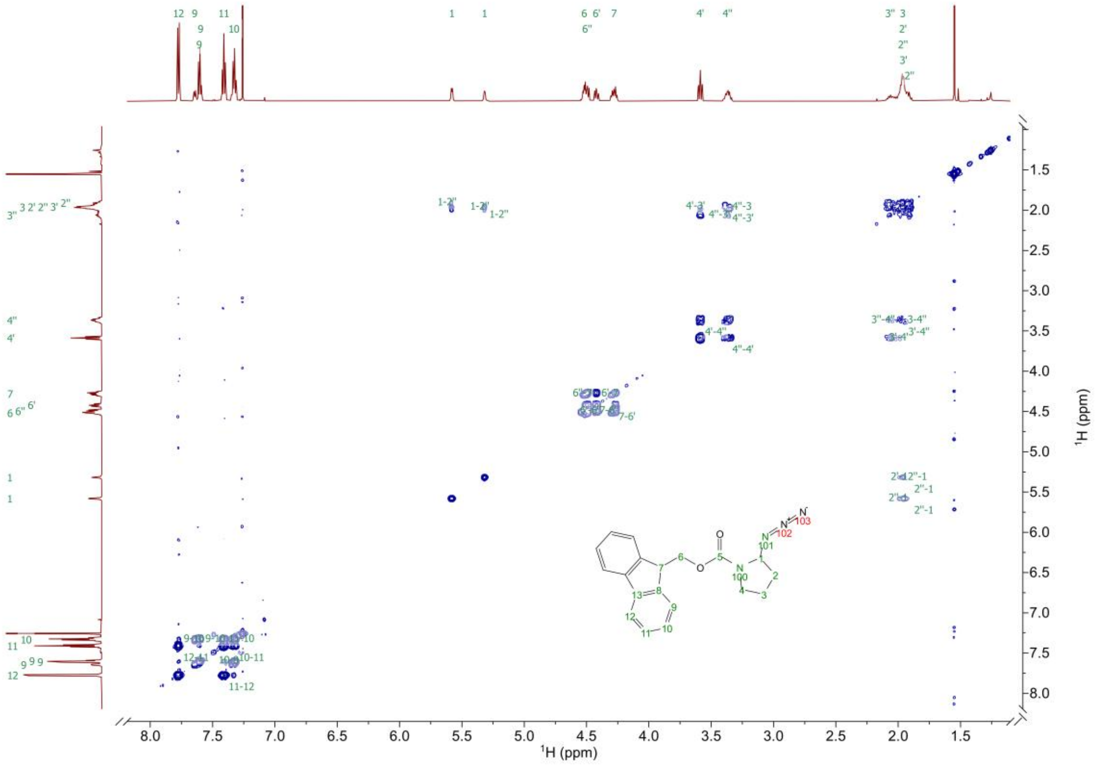

Compound **9m**: ^{1}H-^{1}H NOESY ($CDCl_3$, 298 K)

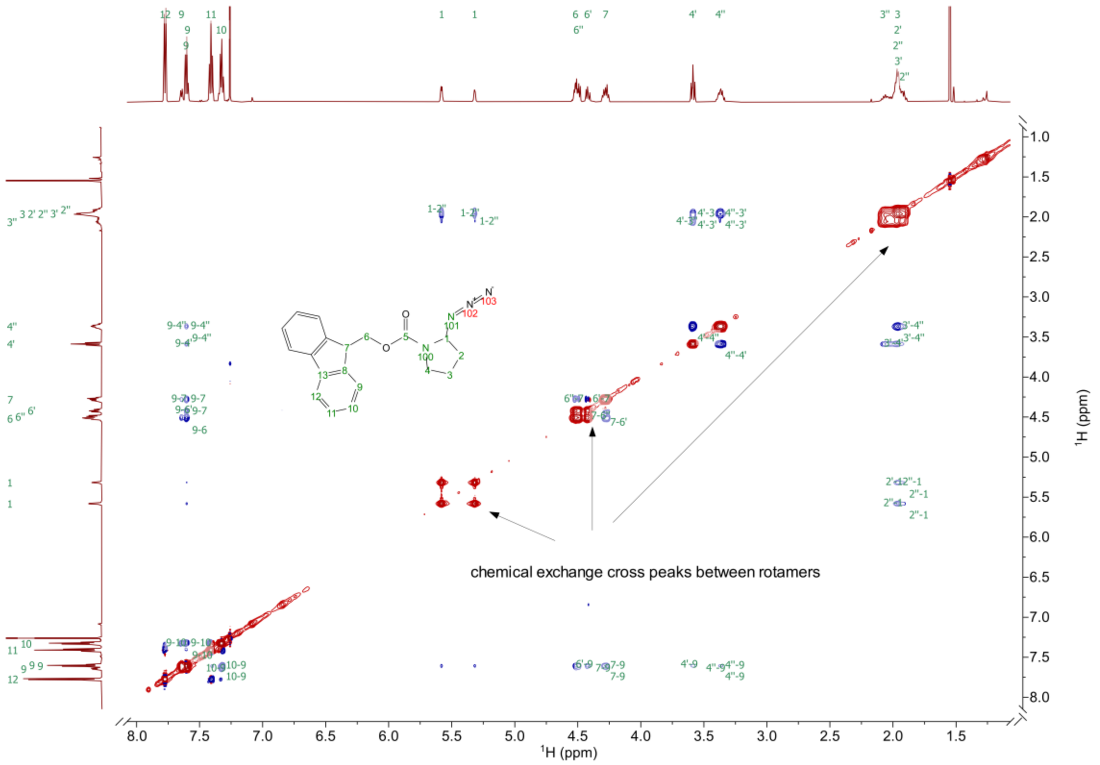

Compound **9m**: ^{1}H-^{15}N HMBC ($CDCl_3$, 298 K)

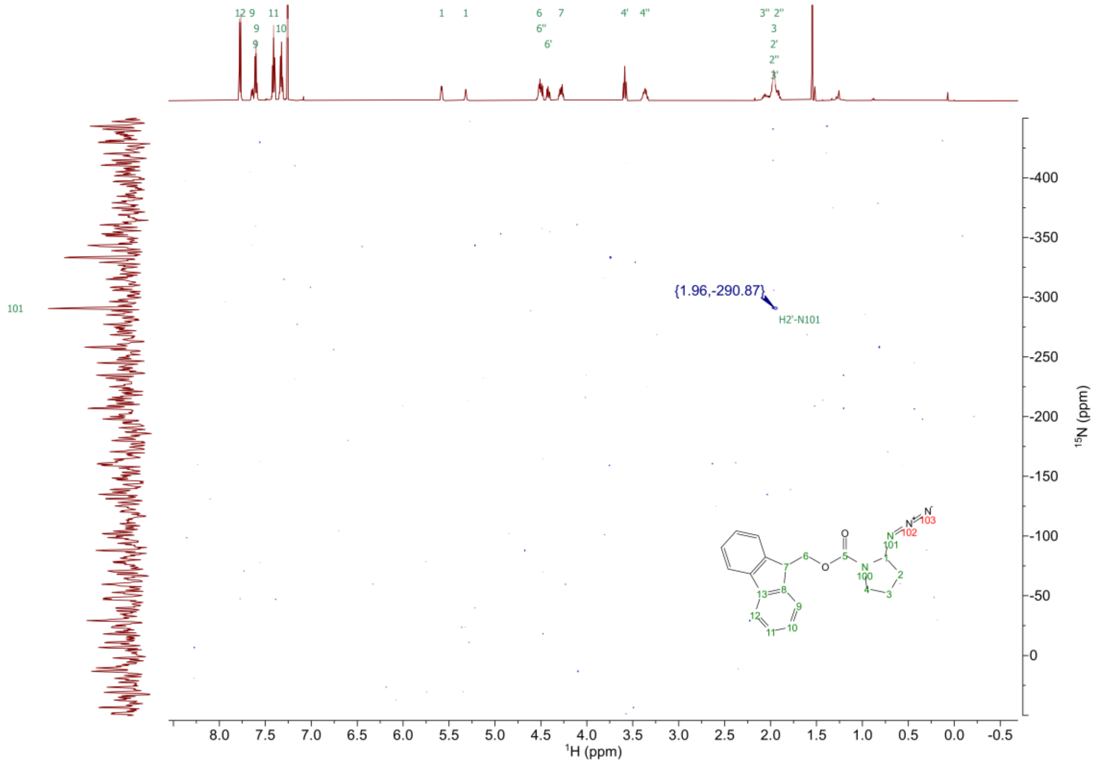

Compound **9n**: ^{1}H NMR (600 MHz, $CDCl_3$, 298 K)

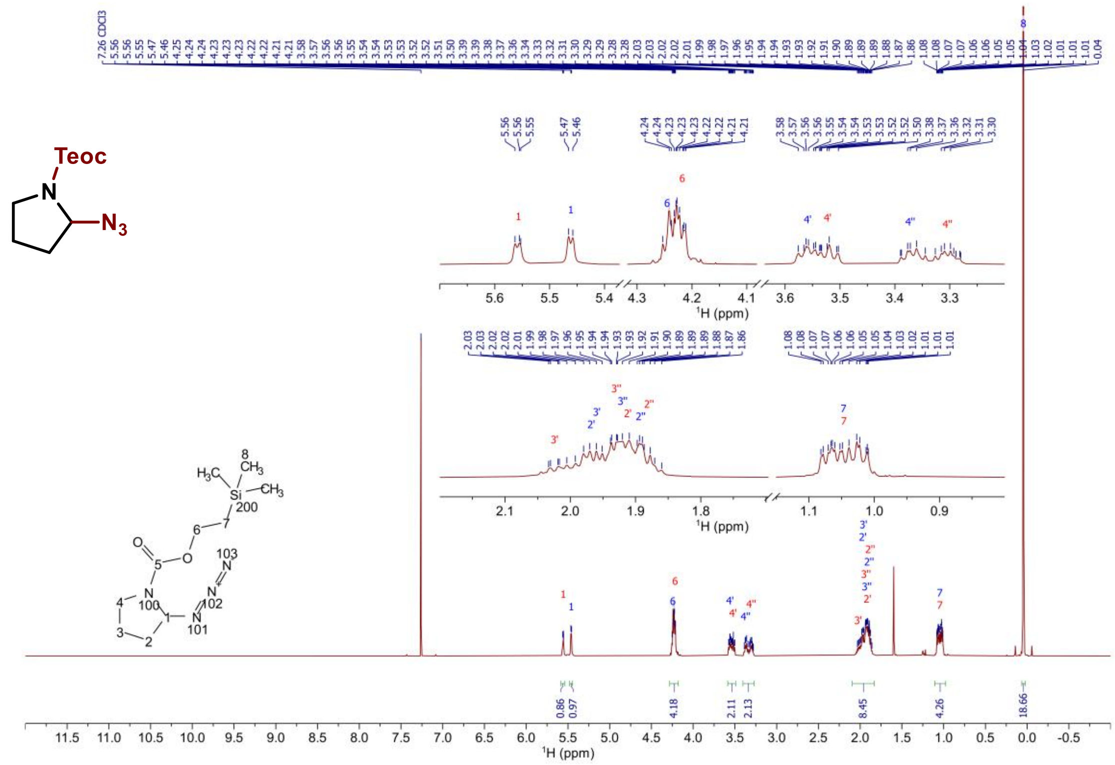

Compound **9n**: ^{13}C NMR (151 MHz, $CDCl_3$, 298 K)

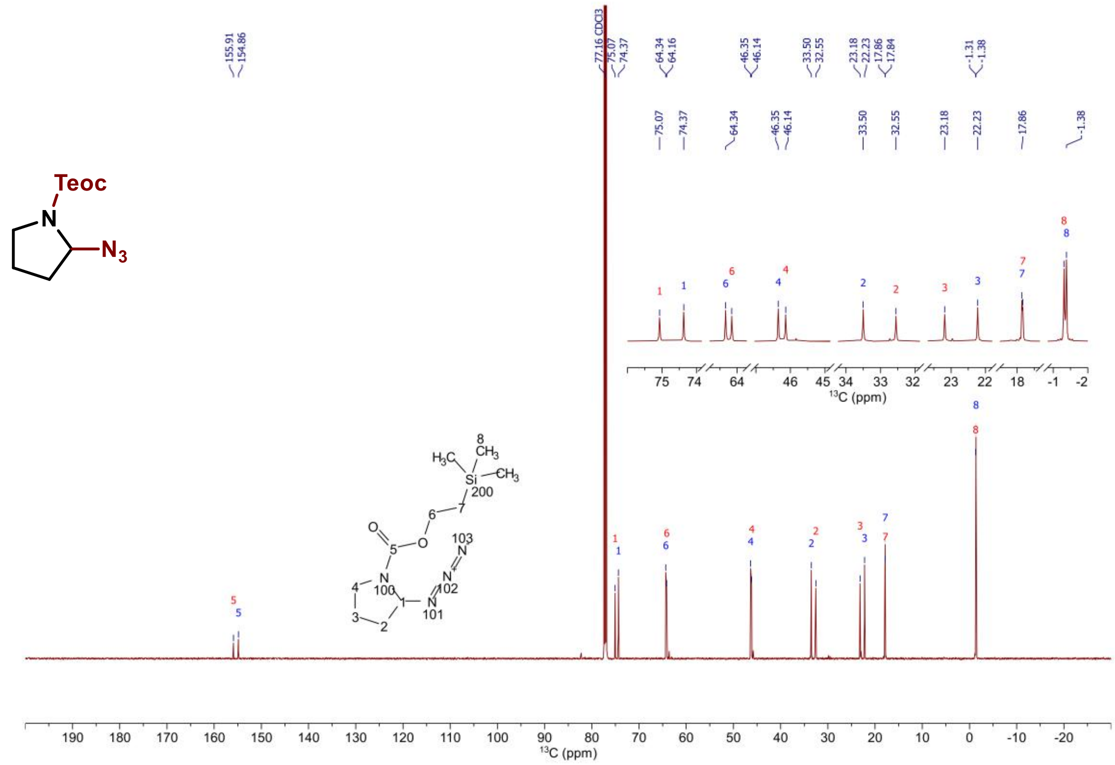

Compound **9n**: ^{1}H-^{13}C HSQC ($CDCl_3$, 298 K)

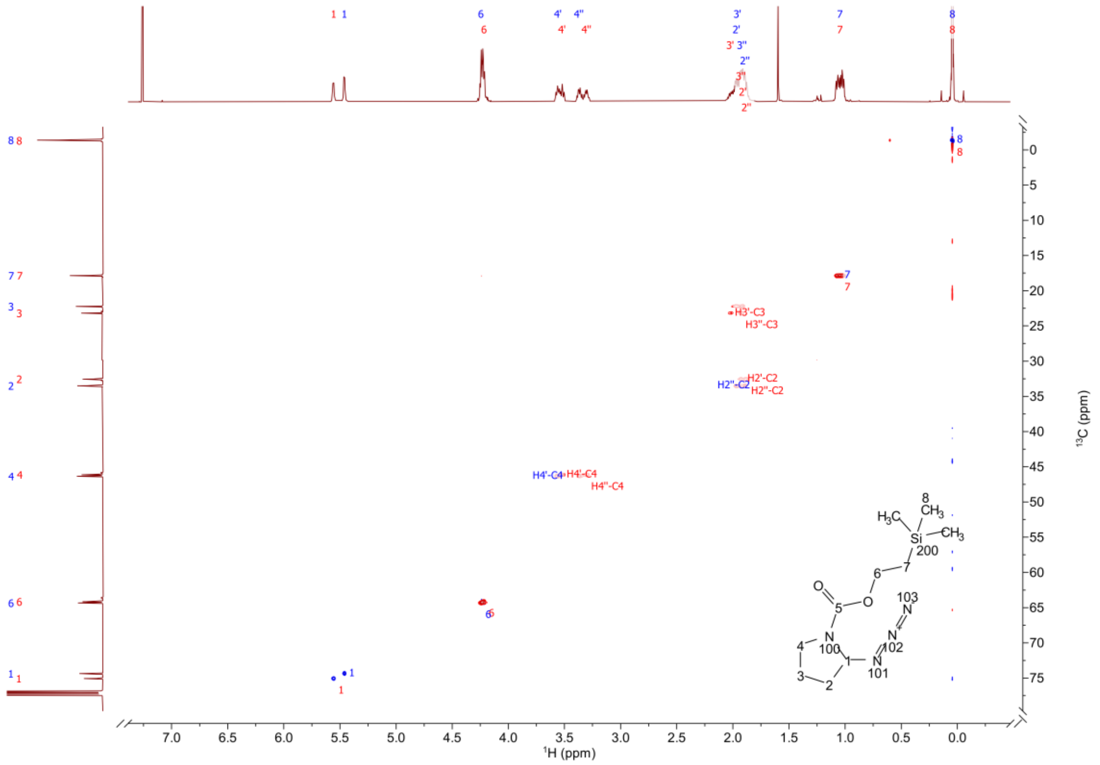

Compound **9n**: ^{1}H-^{13}C HMBC ($CDCl_3$, 298 K)

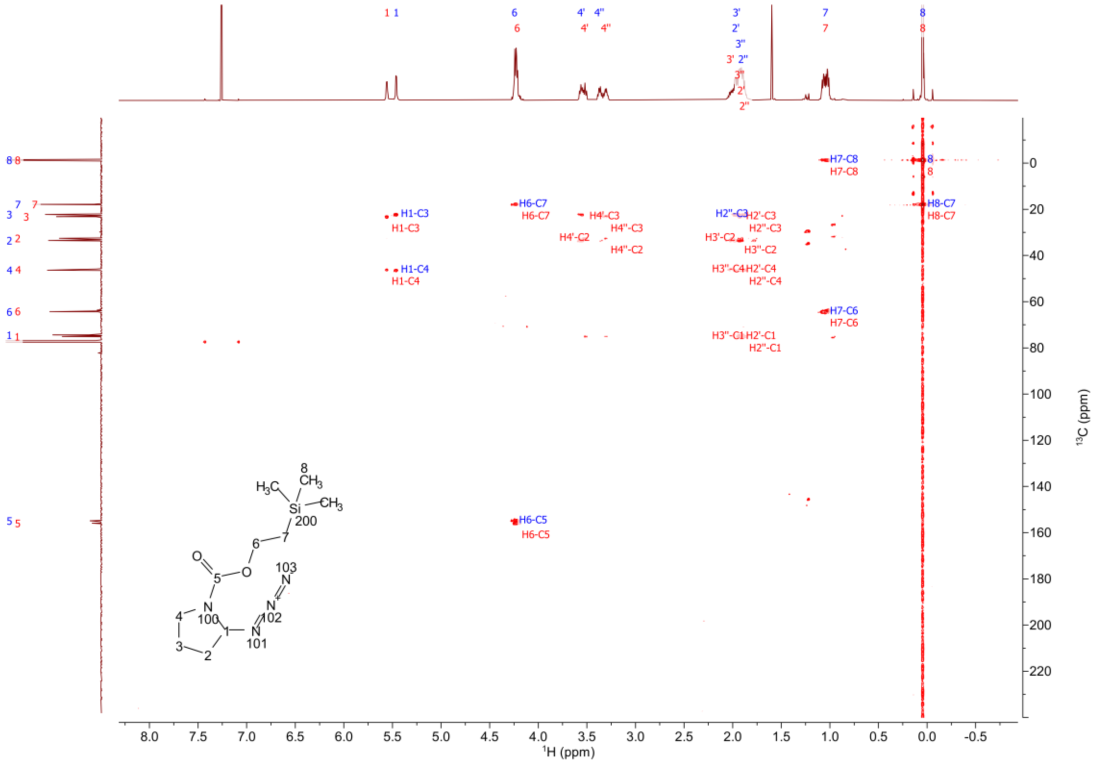

Compound **9n**: ^{1}H-^{1}H COSY ($CDCl_3$, 298 K)

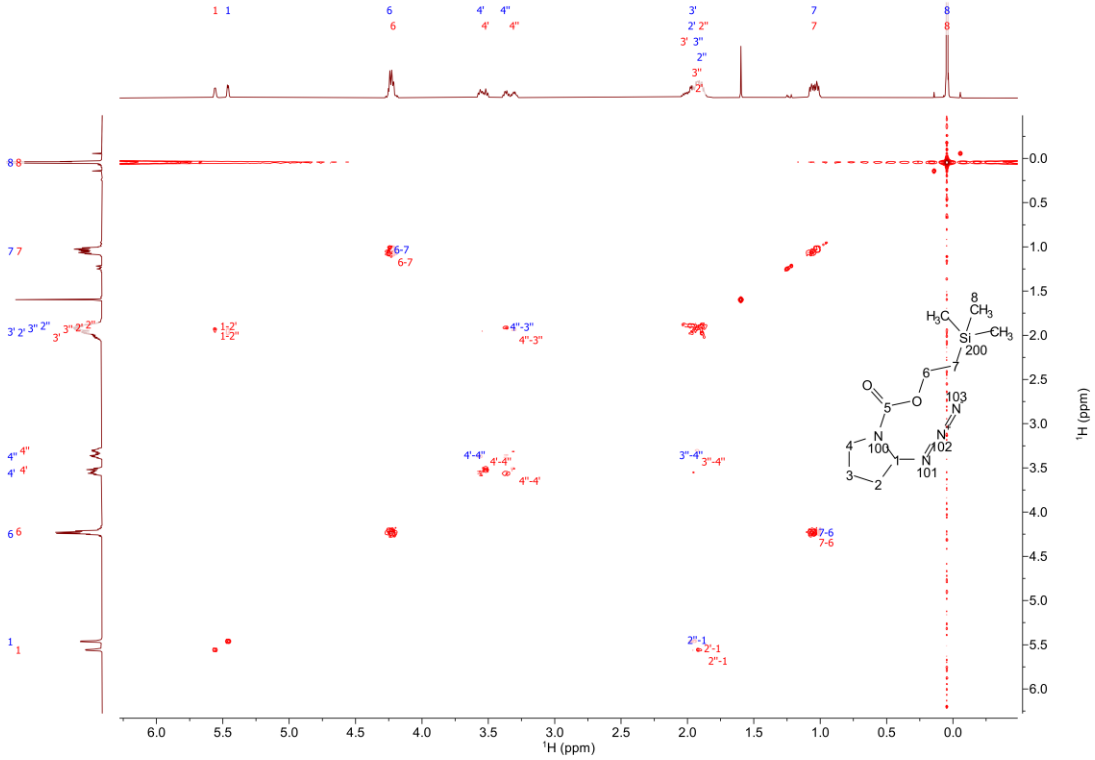

Compound **9n**: ^{1}H-^{1}H NOESY ($CDCl_3$, 298 K)

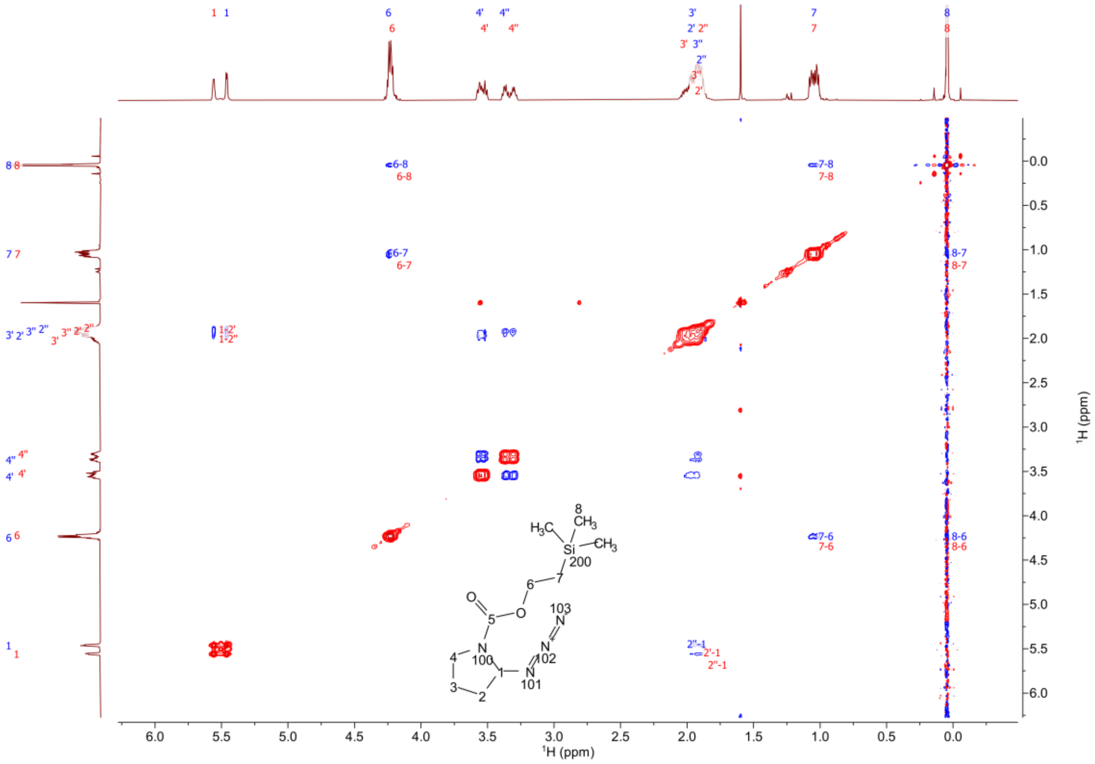

Compound **9n**: ^{29}Si (119 MHz, $CDCl_3$, 298 K)

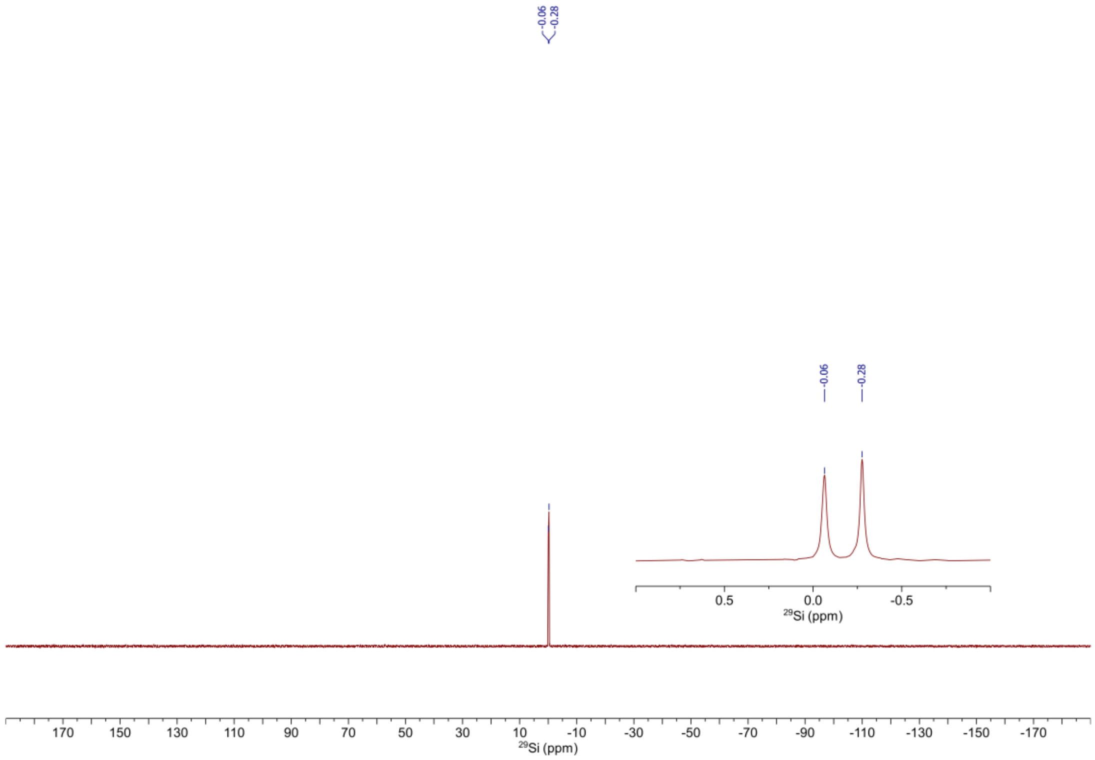

Compound **9o**: ^{1}H NMR (600 MHz, $CDCl_3$, 298 K)

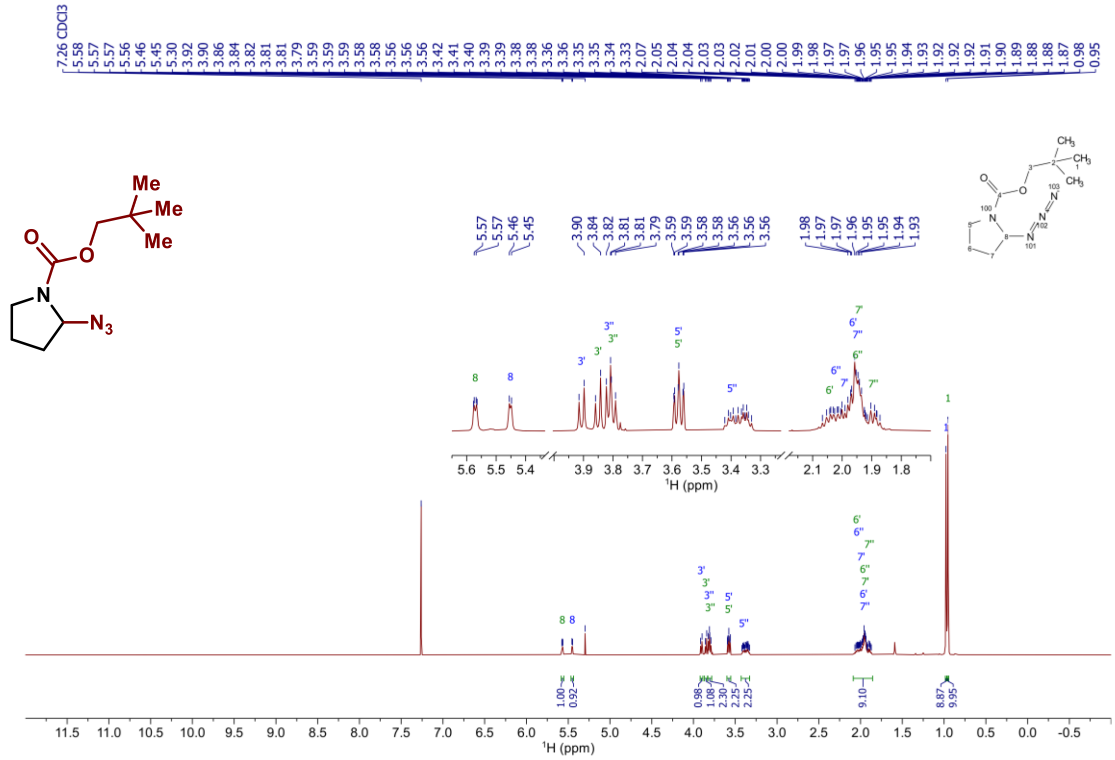

Compound **9o**: ^{13}C NMR (151 MHz, $CDCl_3$, 298 K)

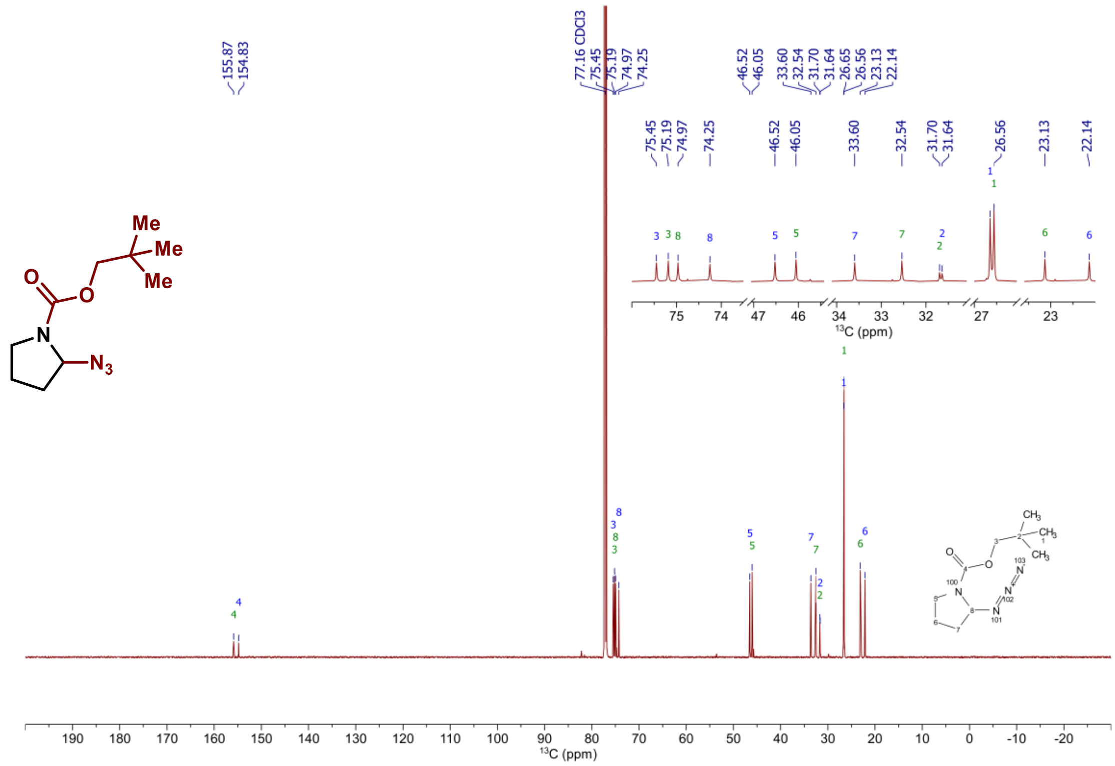

Compound **9o**: ^{1}H-^{13}C HSQC ($CDCl_3$, 298 K)

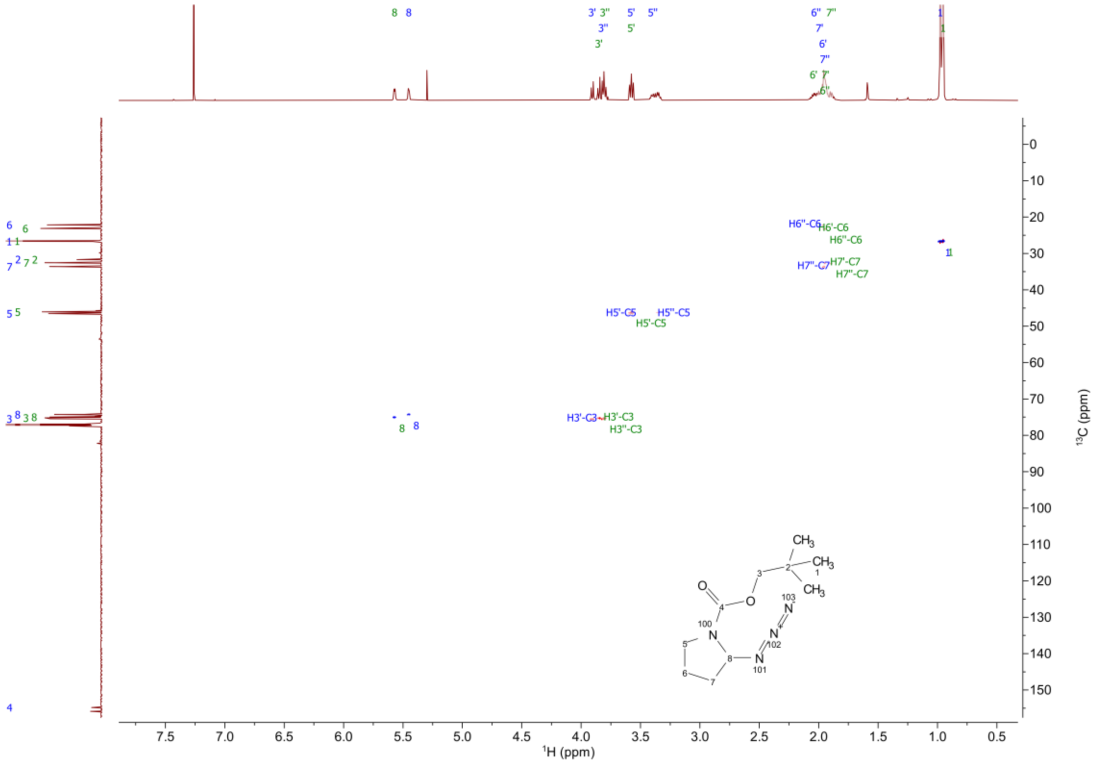

Compound **9o**: ^{1}H-^{13}C HMBC ($CDCl_3$, 298 K)

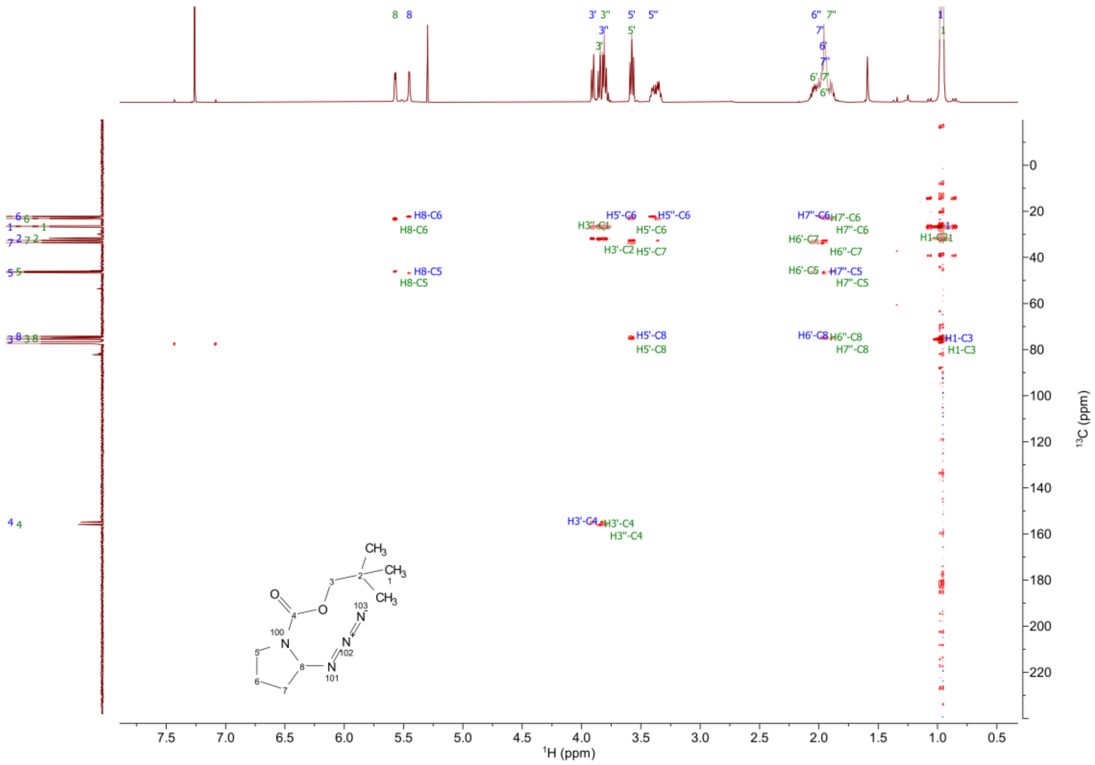

Compound **9o**: ^{1}H-^{1}H COSY ($CDCl_3$, 298 K)

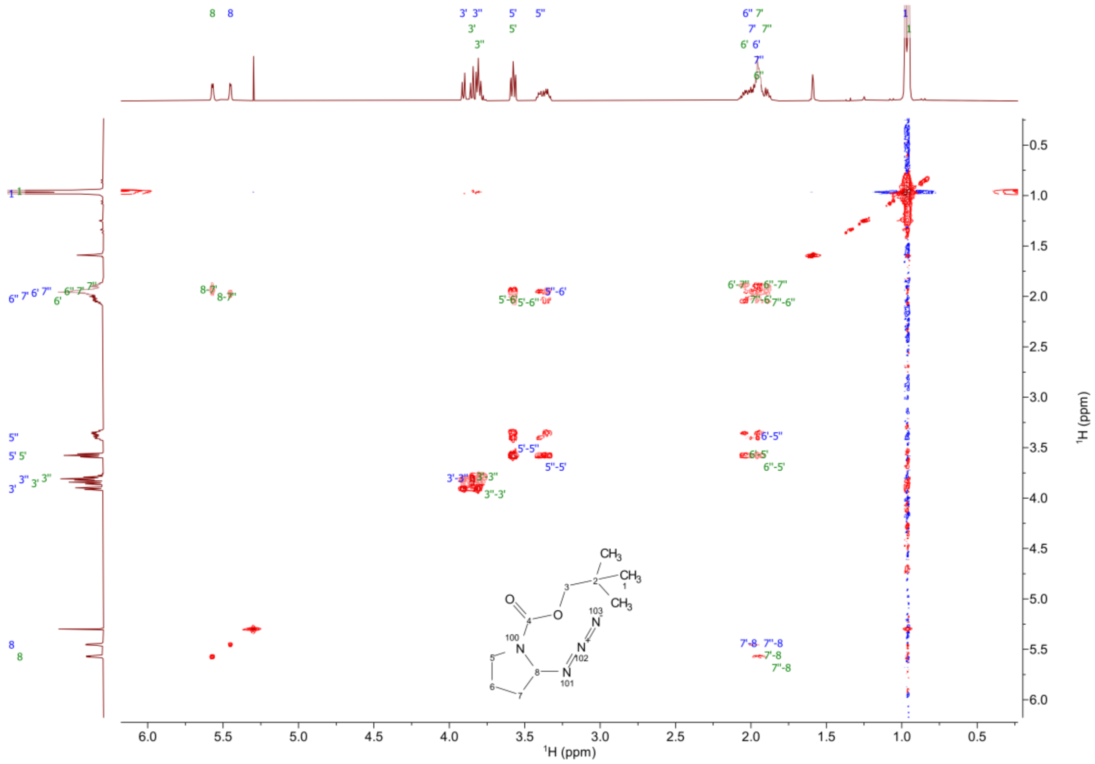

Compound **9o**: ^{1}H-^{1}H NOESY ($CDCl_3$, 298 K)

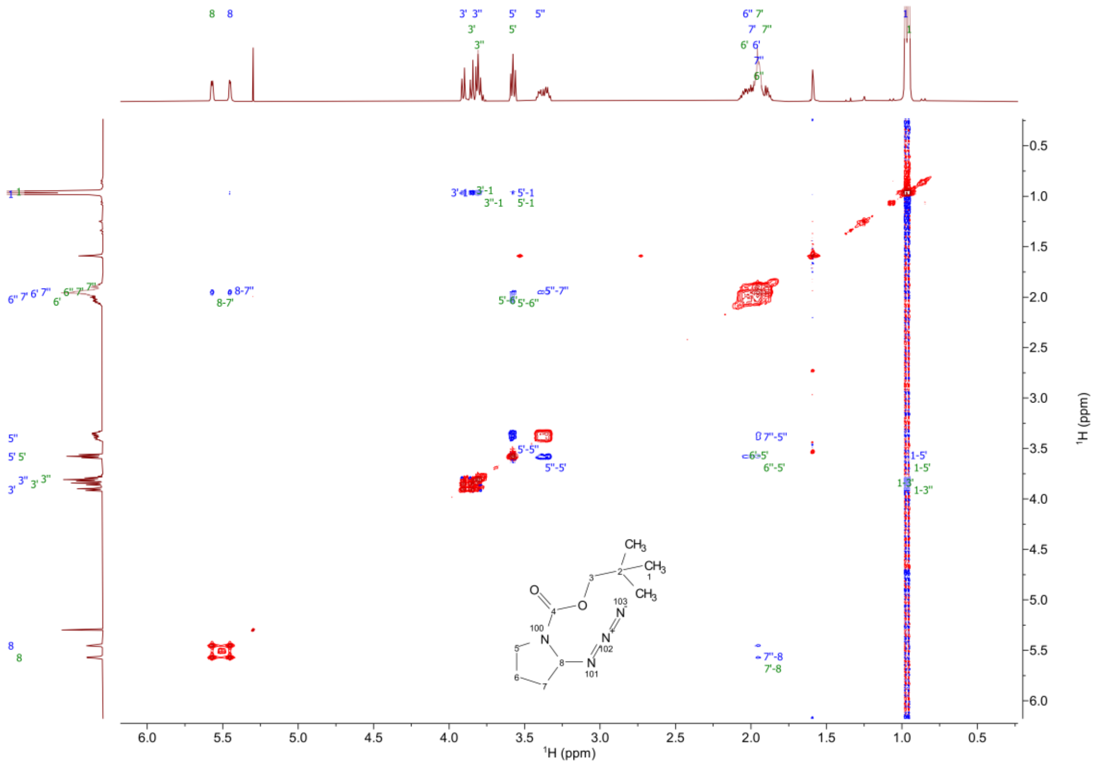

Compound **9p**: ^{1}H NMR (600 MHz, $CDCl_3$, 298 K)

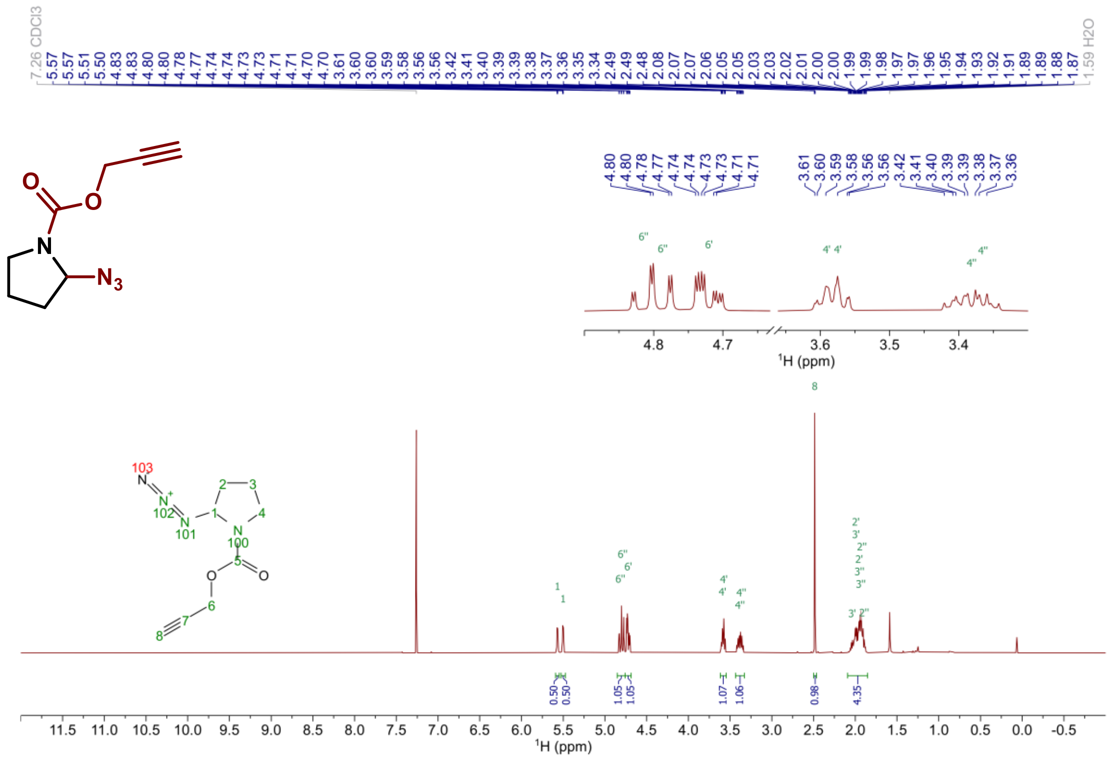

Compound **9p**: ^{13}C NMR (151 MHz, $CDCl_3$, 298 K)

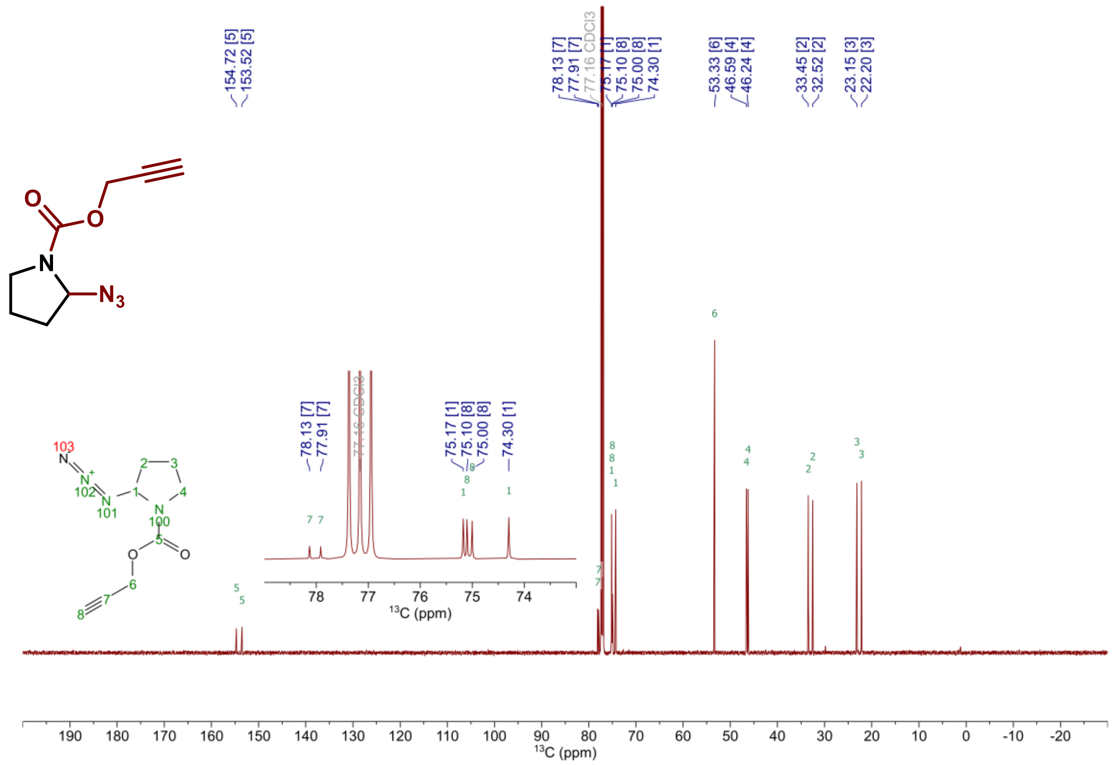

Compound **9p**: ^{1}H-^{13}C HSQC ($CDCl_3$, 298 K)

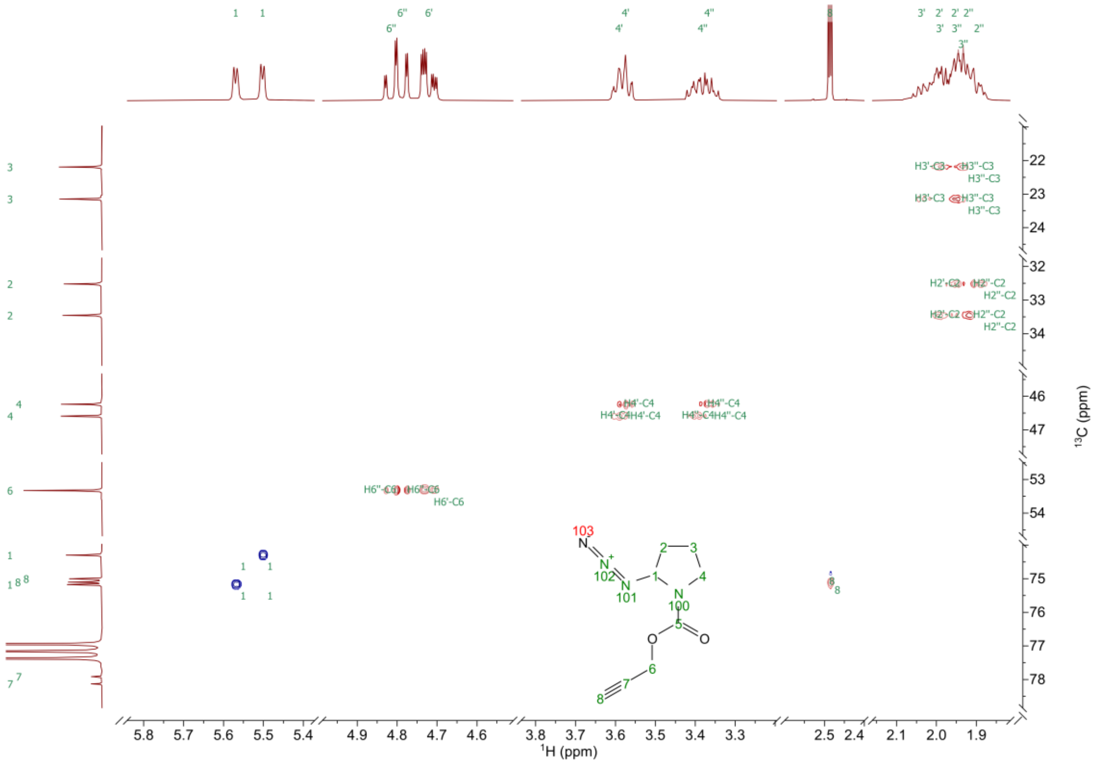

Compound **9p**: ^{1}H-^{13}C HMBC ($CDCl_3$, 298 K)

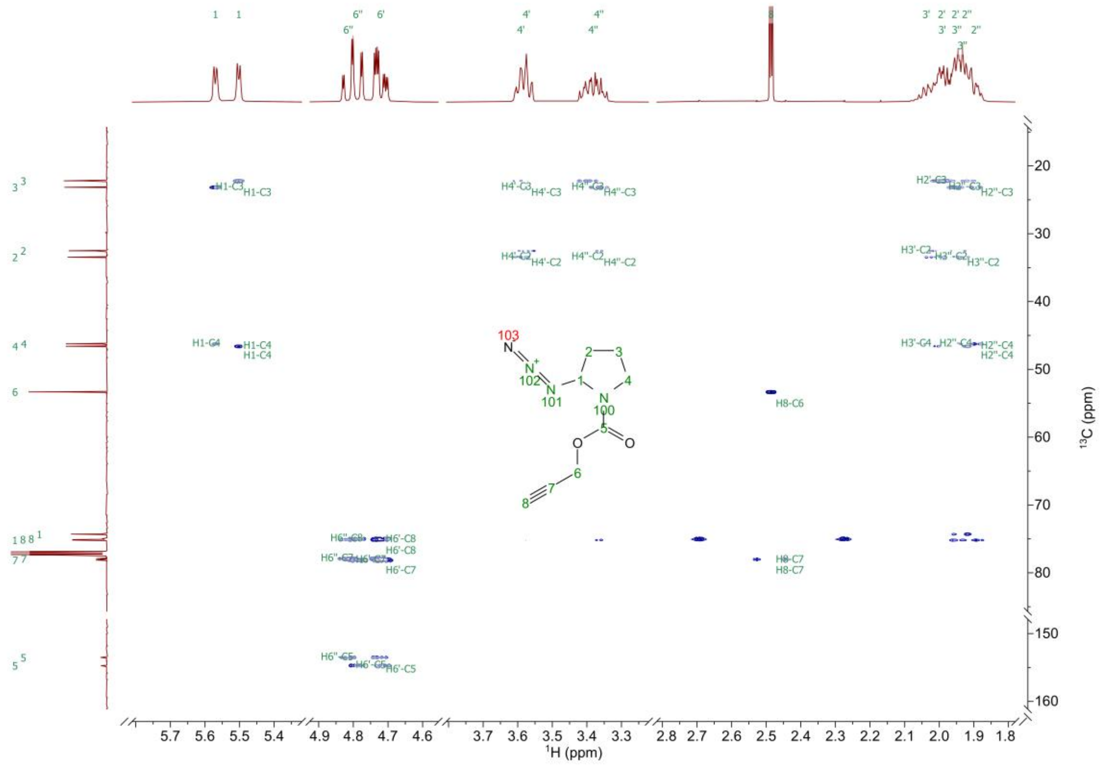

Compound **9p**: ^{1}H-^{1}H COSY ($CDCl_3$, 298 K)

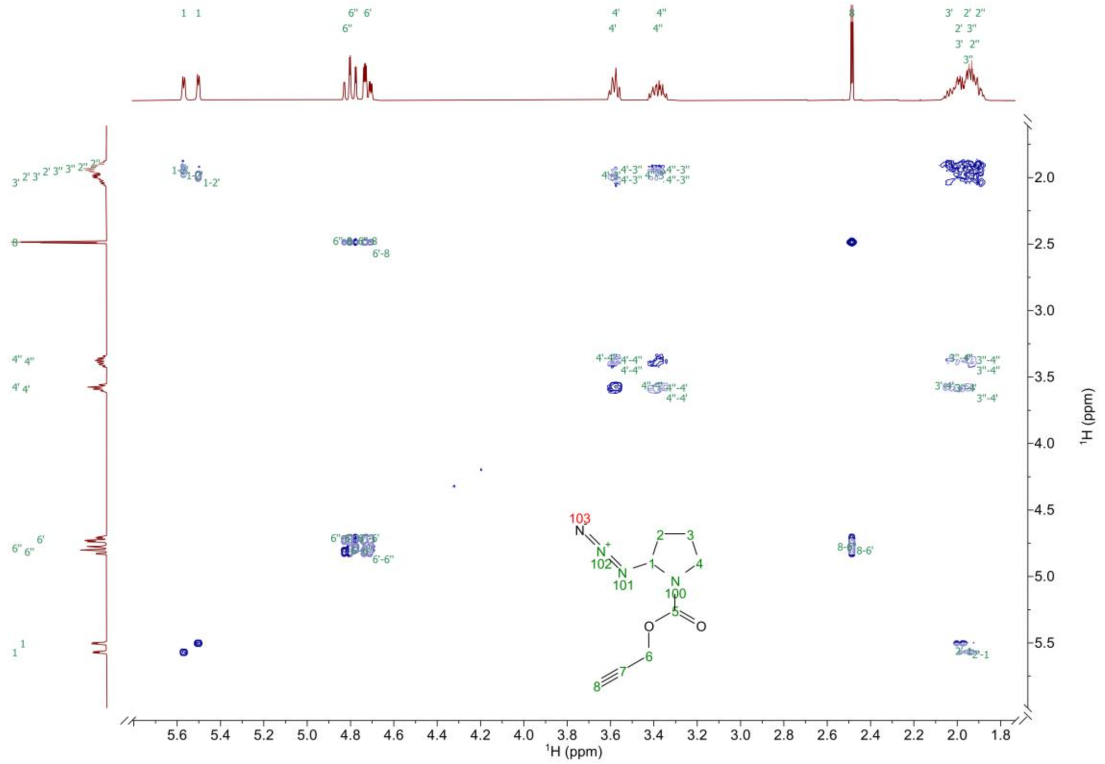

Compound **9p**: ^{1}H-^{1}H NOESY ($CDCl_3$, 298 K)

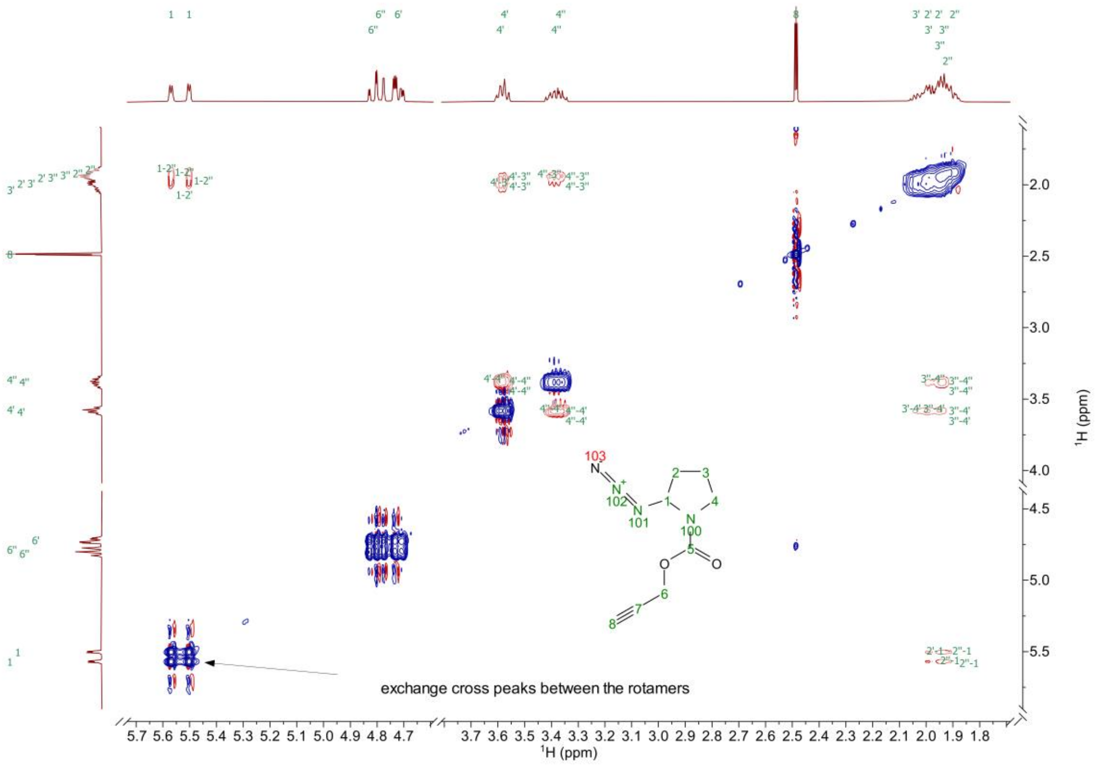

Compound **9p**: ^{1}H-^{15}N HMBC ($CDCl_3$, 298 K)

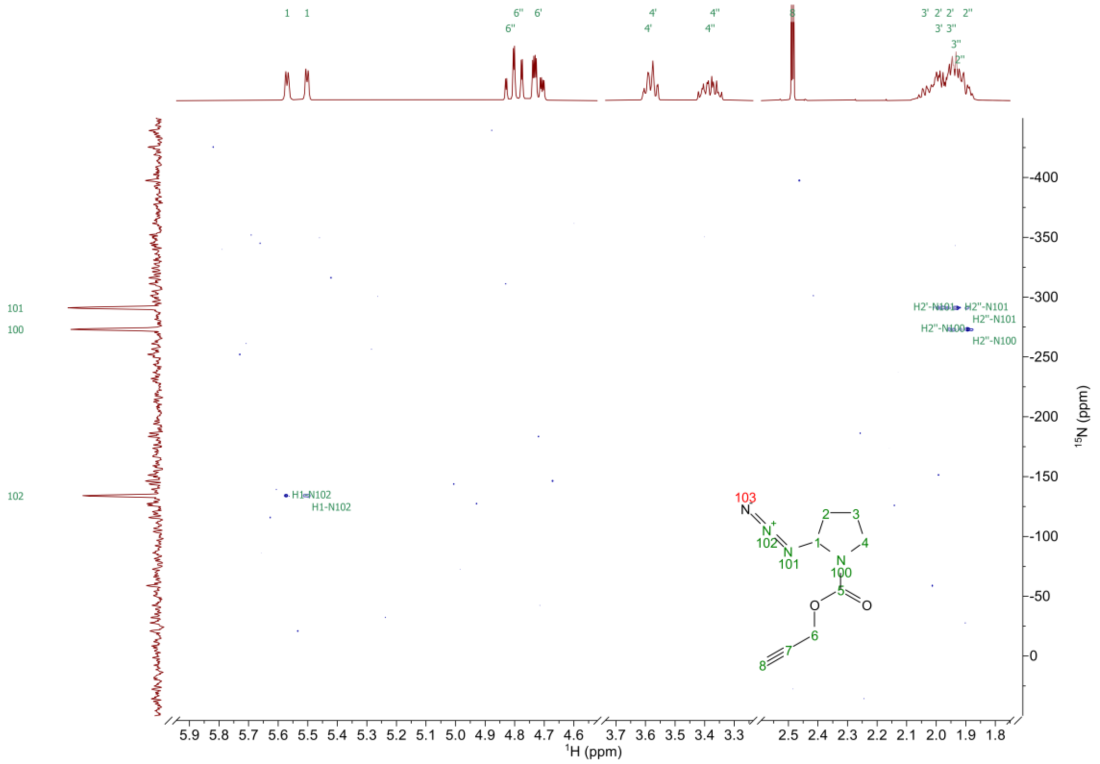

Compound **9q**: ^{1}H NMR (600 MHz, $CDCl_3$, 253 K)

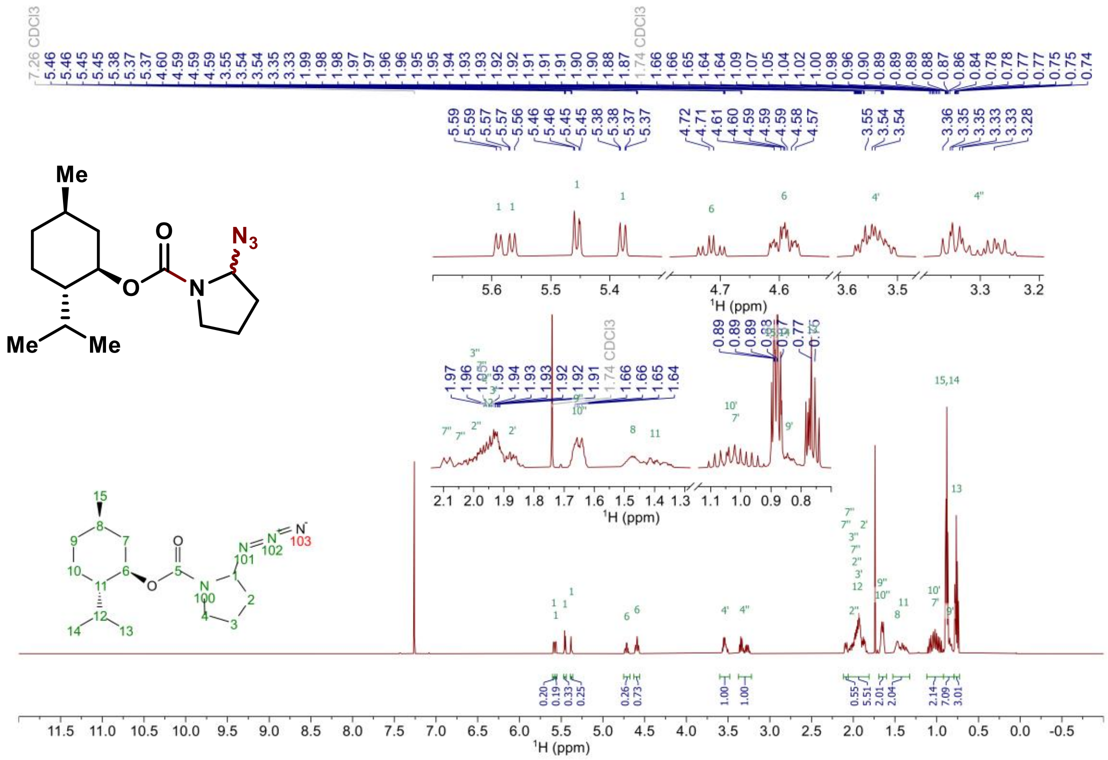

Compound **9q**: ^{13}C NMR (151 MHz, $CDCl_3$, 253 K)

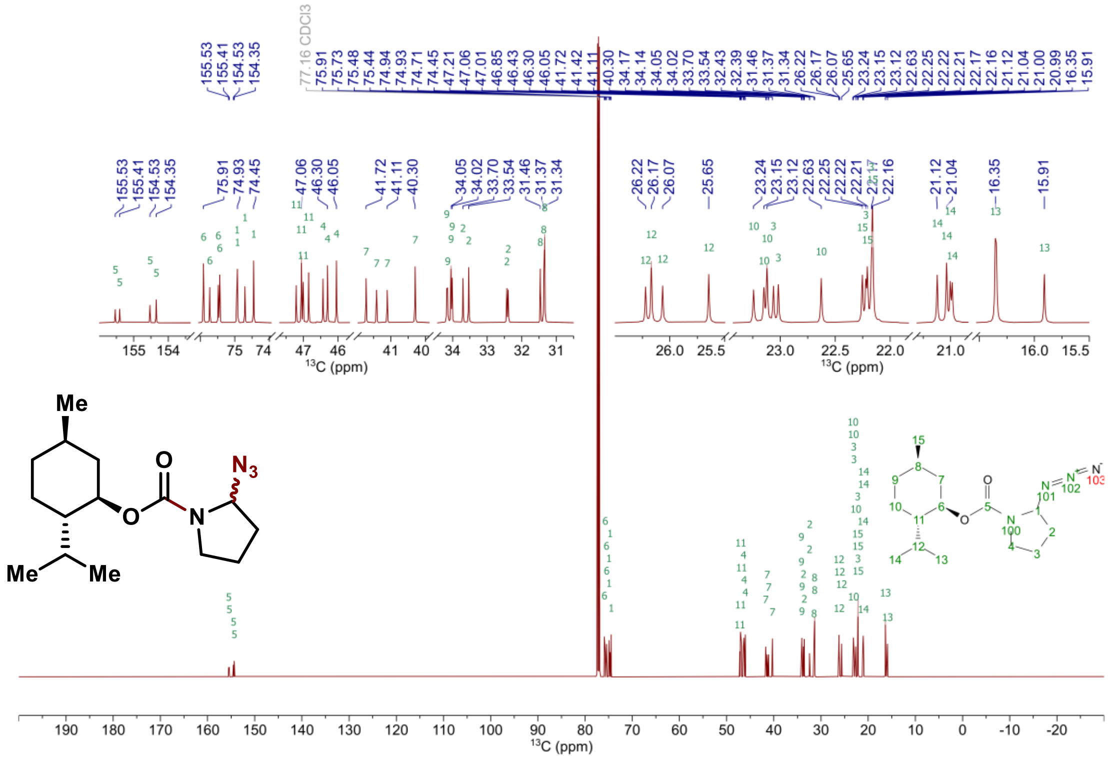

Compound **9q**: variable temperature ^{1}H NMR (600 MHz, $CDCl_3$)

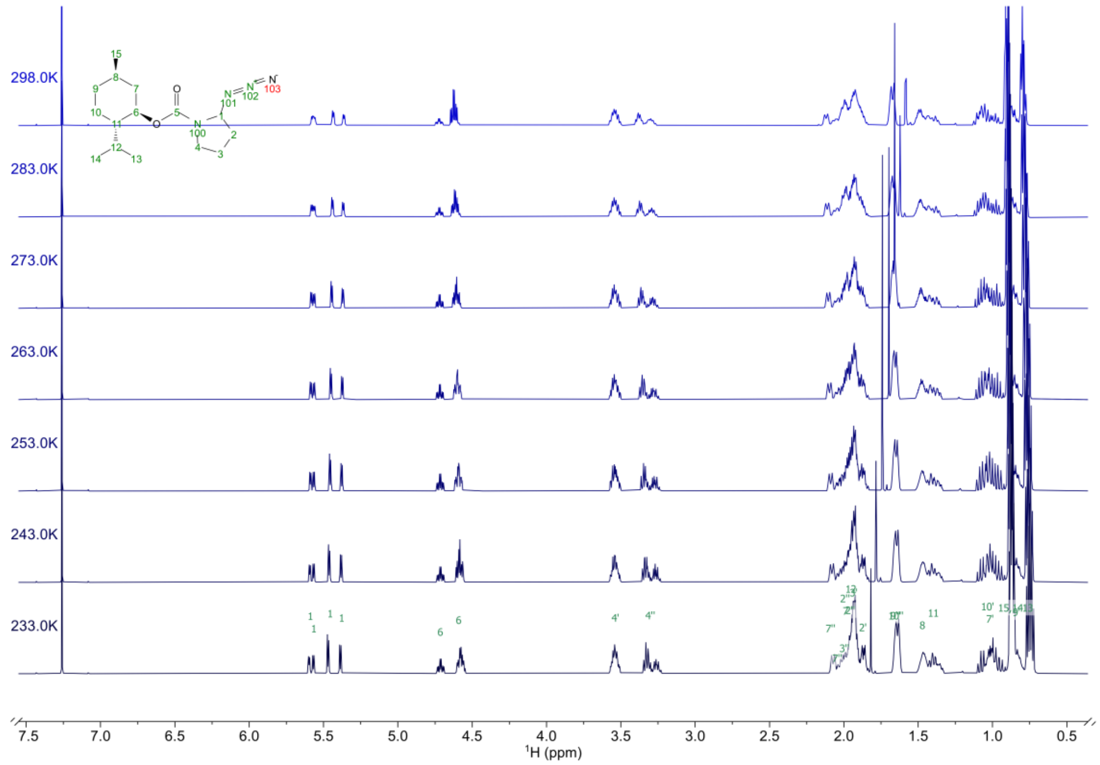

Compound **9q**: ^{1}H-^{13}C HSQC ($CDCl_3$, 253 K)

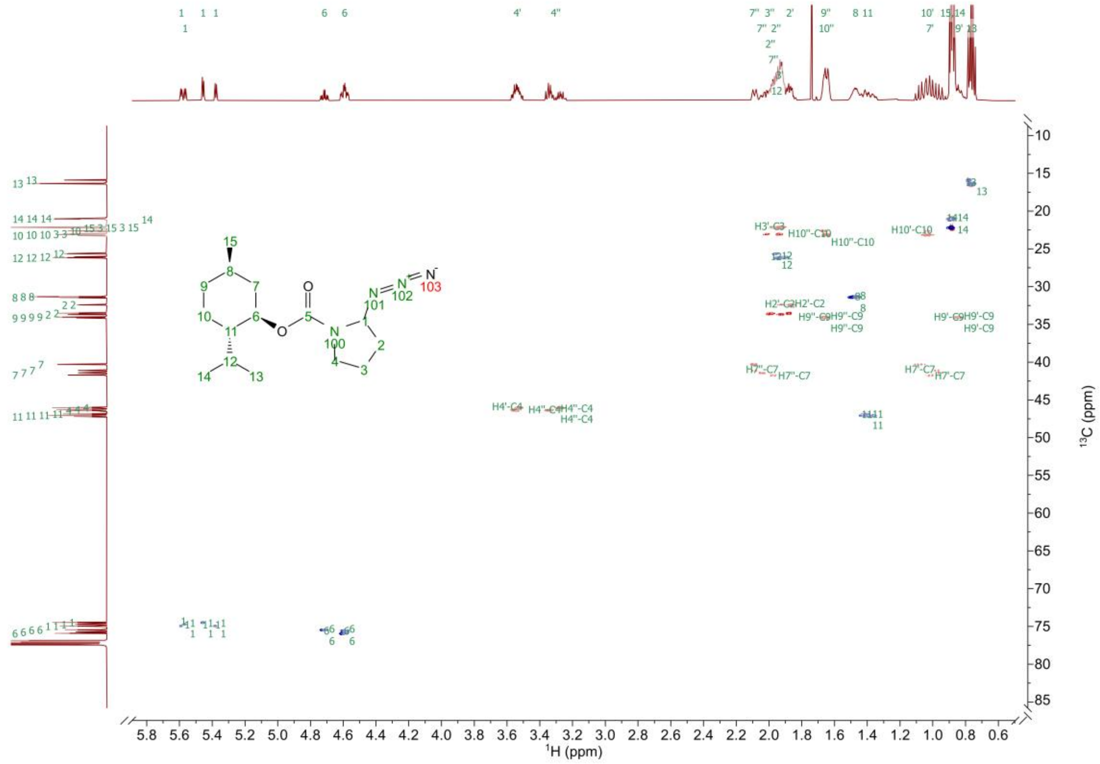

Compound **9q**: ^{1}H-^{13}C HMBC ($CDCl_3$, 253 K)

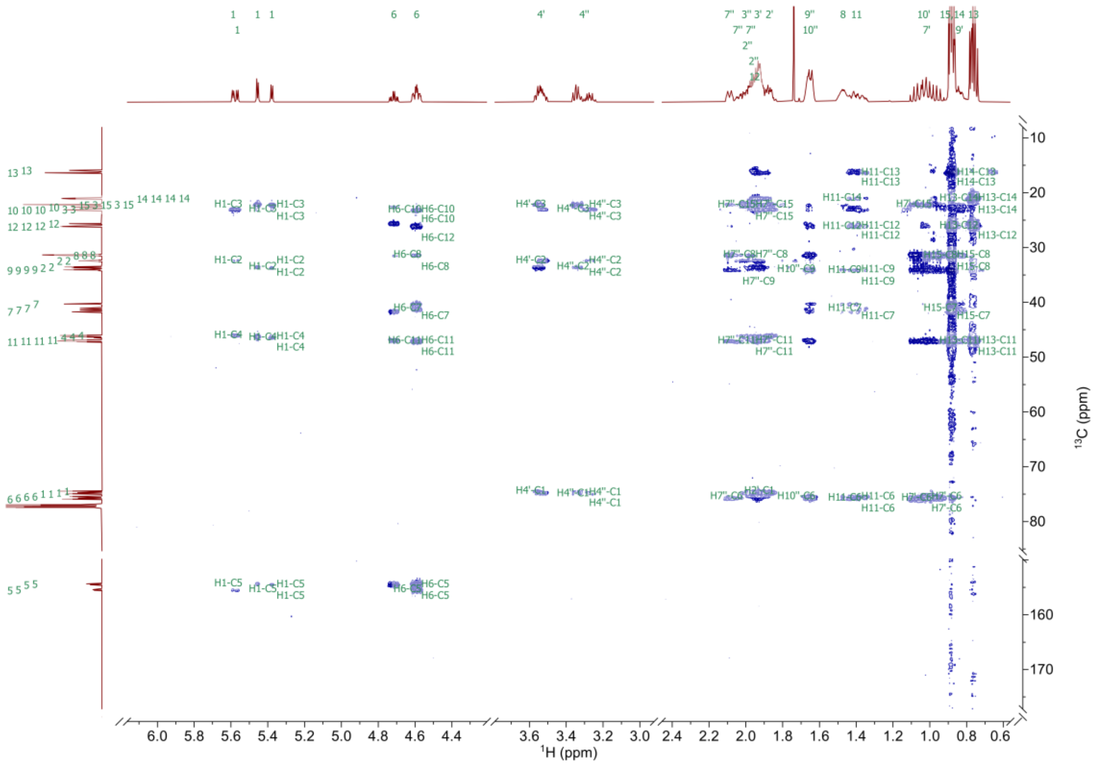

Compound **9q**: ^{1}H-^{1}H COSY ($CDCl_3$, 253 K)

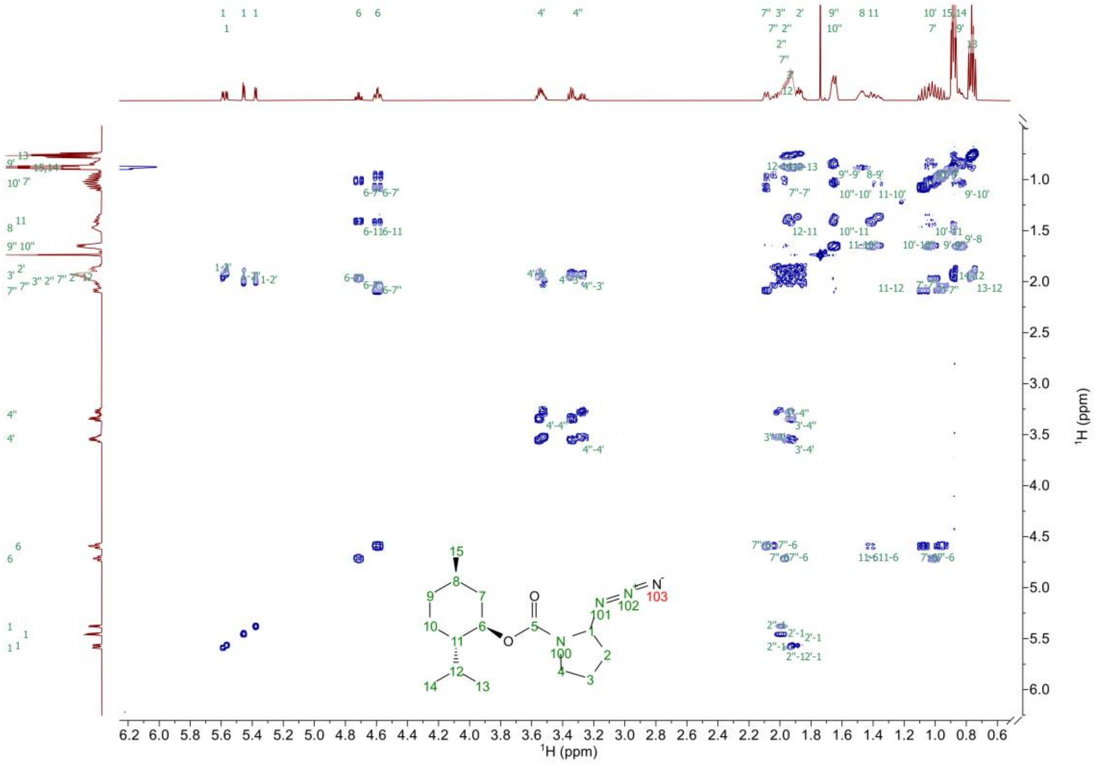

Compound **9q**: ^{1}H-^{1}H NOESY ($CDCl_3$, 253 K)

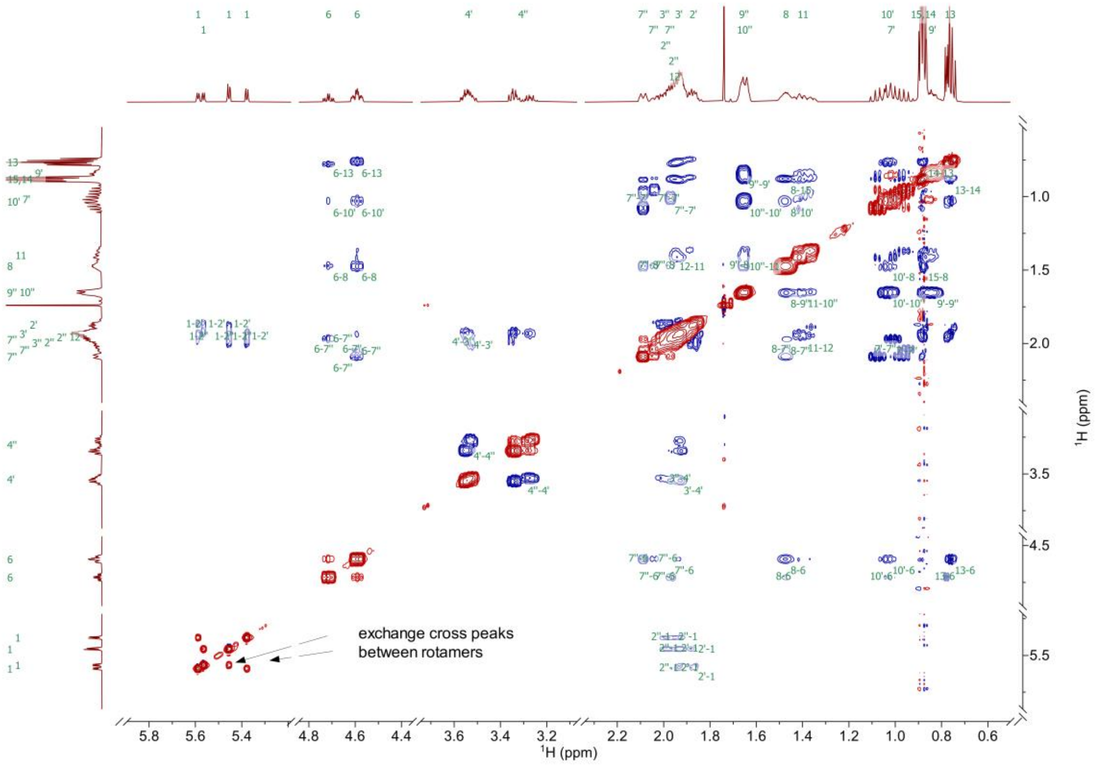

Compound **9q**: ^{1}H-^{15}N HMBC ($CDCl_3$, 253 K)

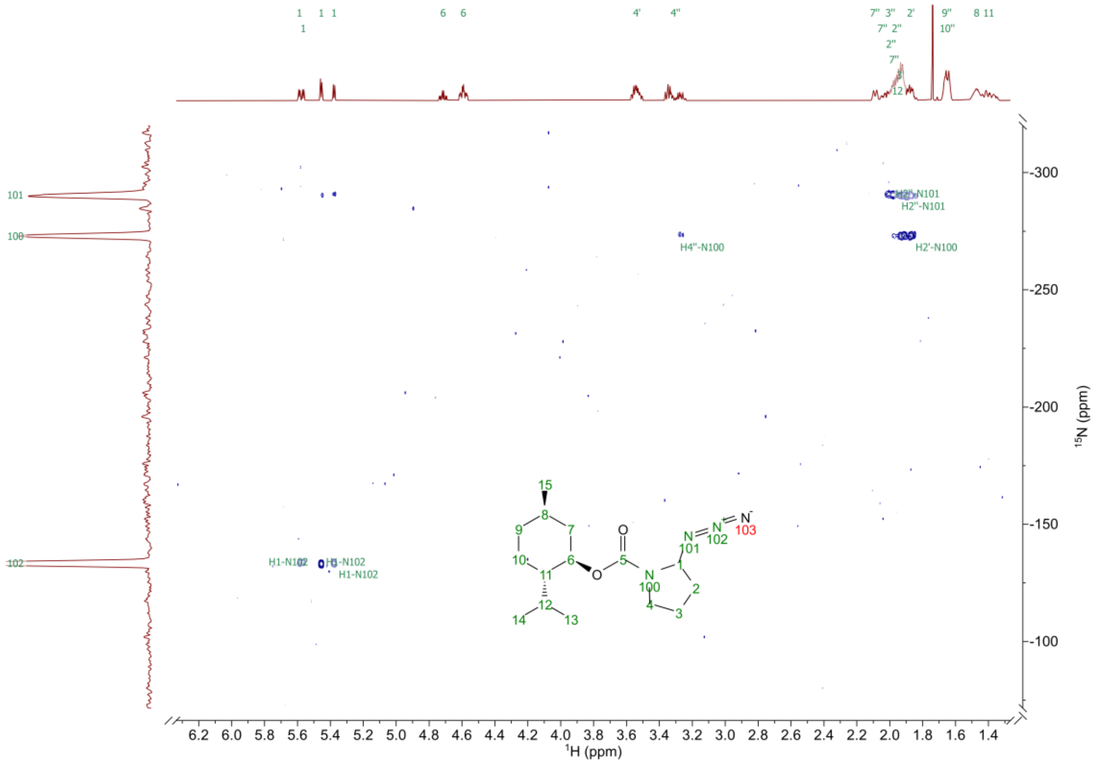

Compound **9r**: ^{1}H NMR (600 MHz, $CDCl_3$, 298 K)

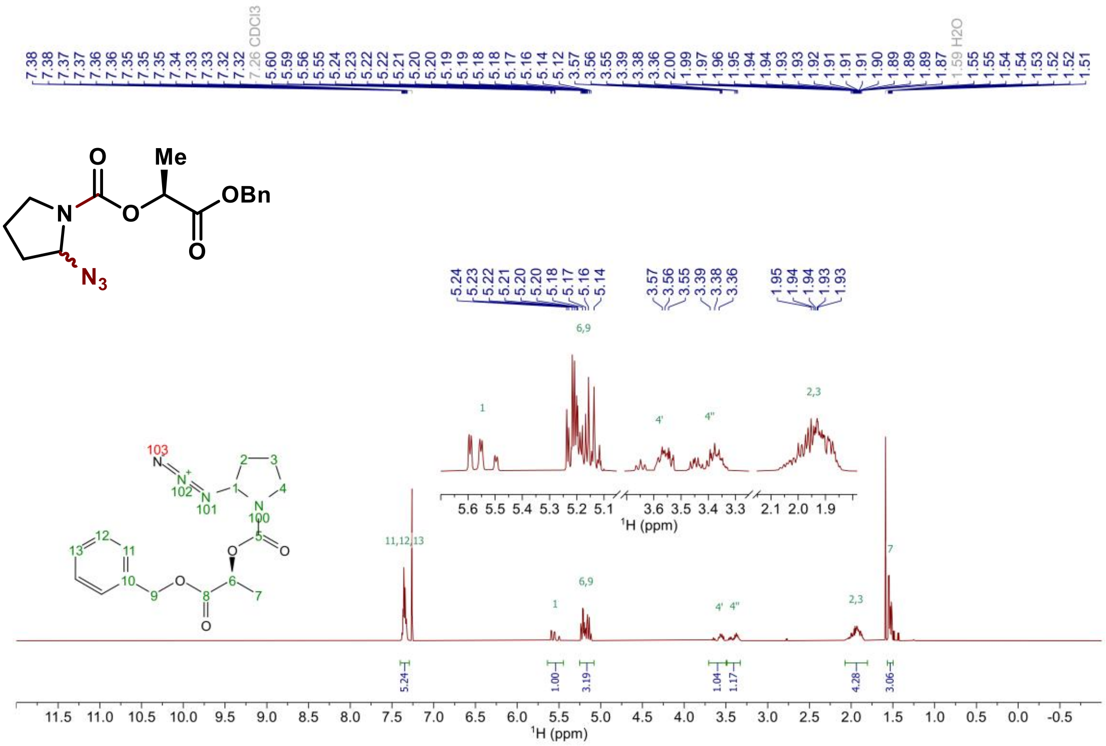

Compound **9r**: ^{13}C NMR (151 MHz, $CDCl_3$, 298 K)

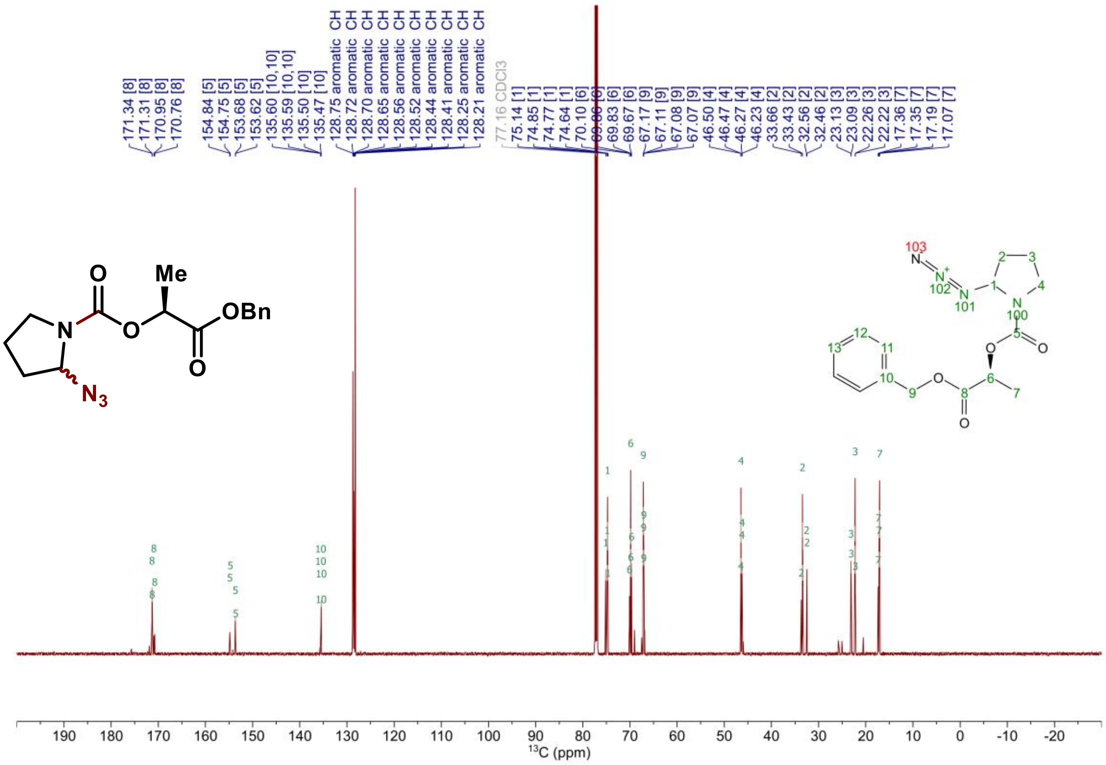

Compound **9r**: ^{1}H-^{13}C HSQC ($CDCl_3$, 298 K)

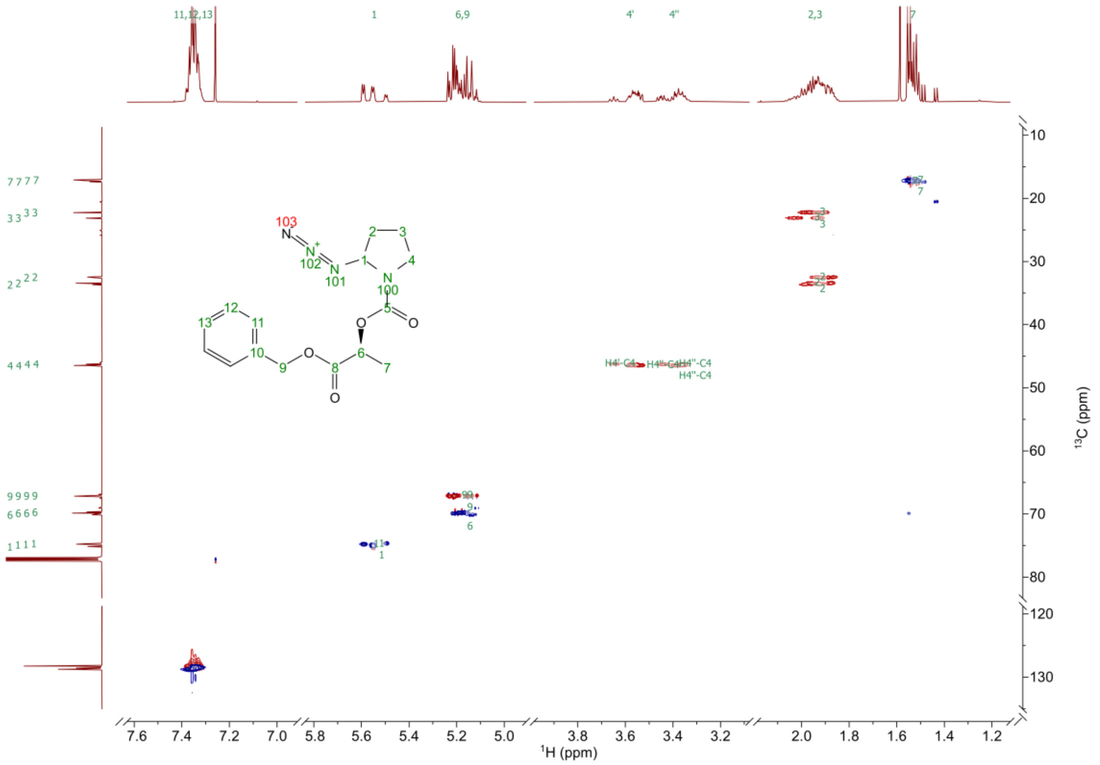

Compound **9r**: ^{1}H-^{13}C HMBC ($CDCl_3$, 298 K)

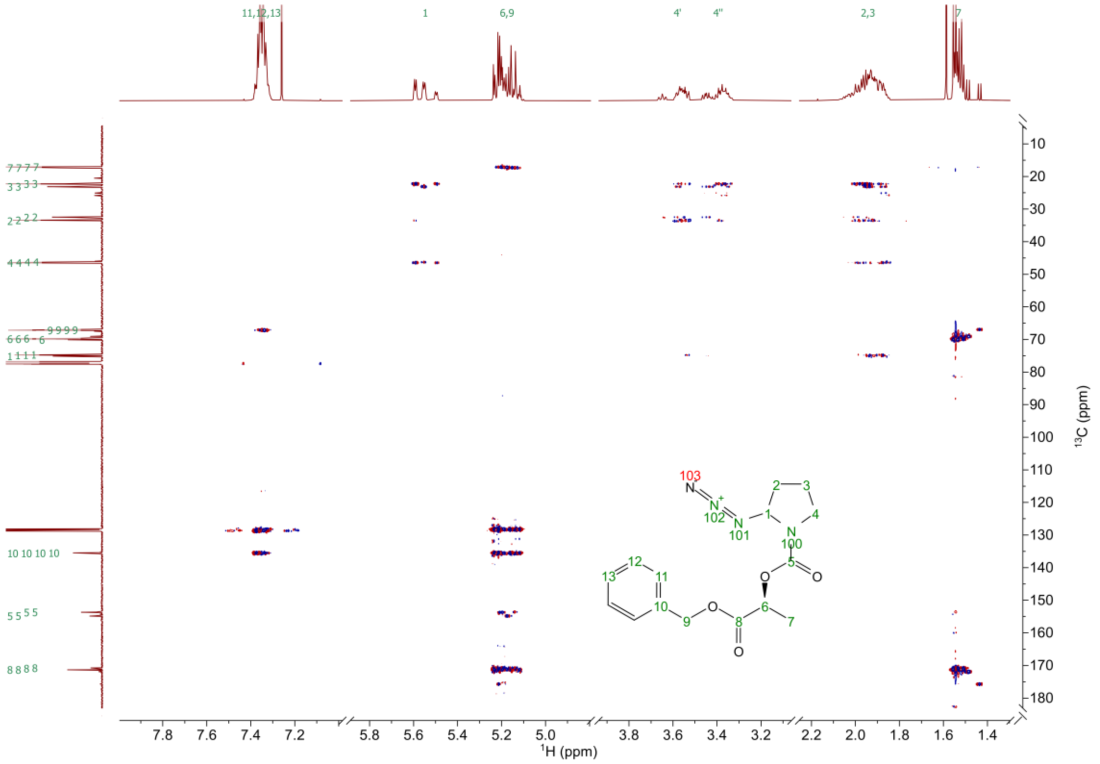

Compound **9r**: ^{1}H-^{1}H COSY ($CDCl_3$, 298 K)

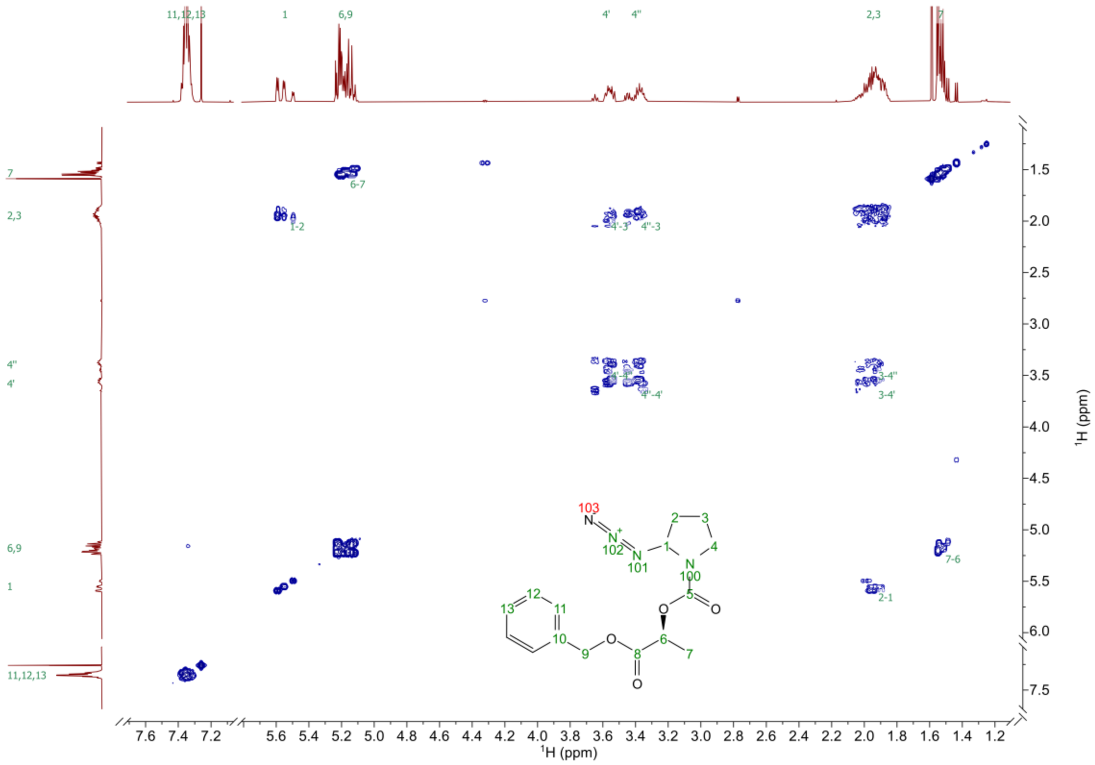

Compound **9r**: ^{1}H-^{1}H NOESY ($CDCl_3$, 298 K)

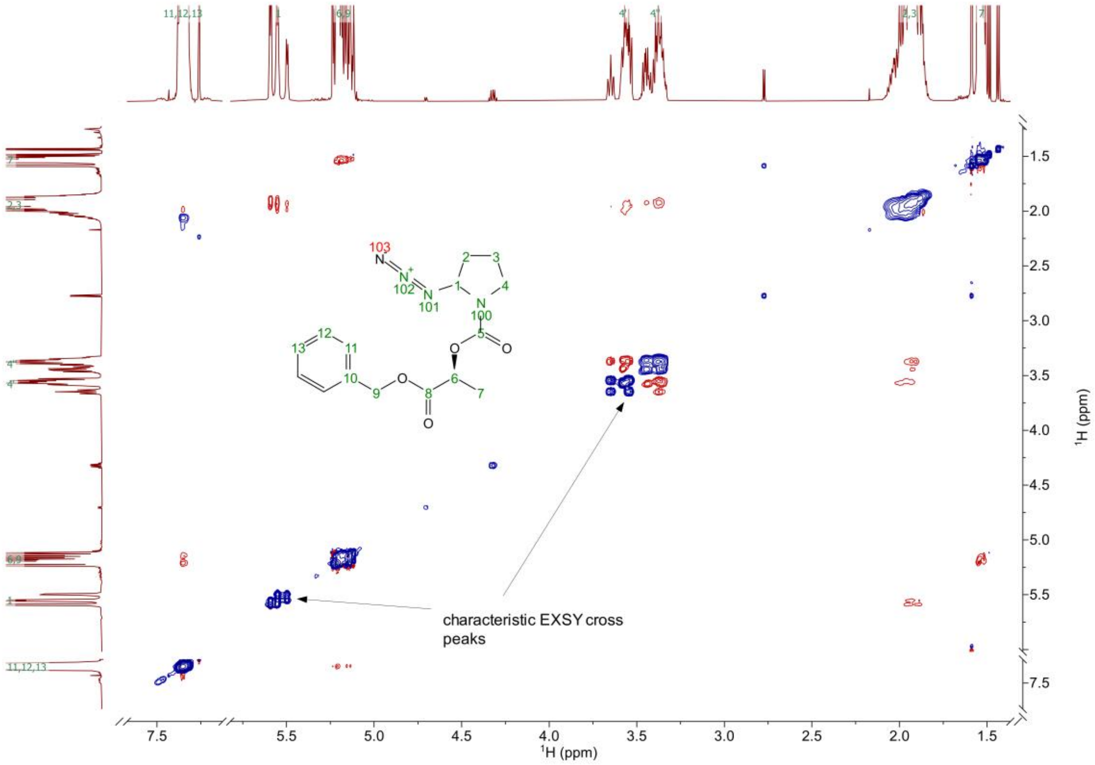

Compound **9r**: ^{1}H-^{15}N HMBC (CDCl$_3$, 298 K)

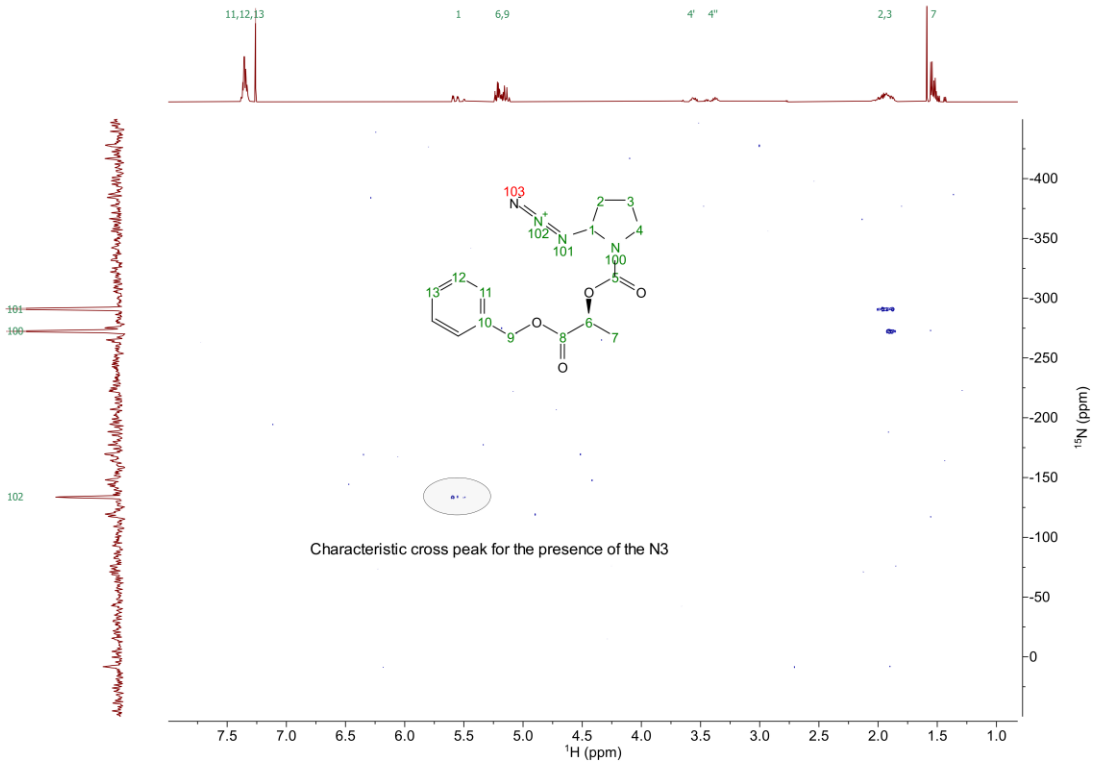

Compound **9s**: ^{1}H NMR (600 MHz, CDCl$_3$, 298 K)

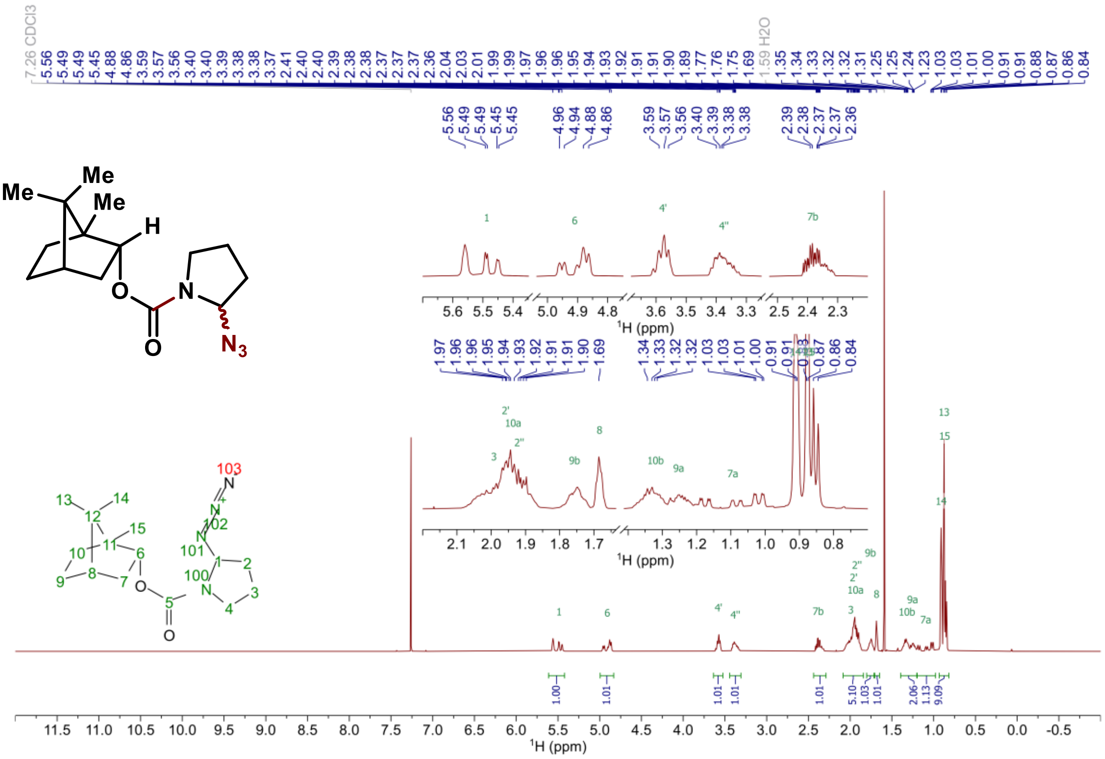

Compound **9s**: ^{13}C NMR (151 MHz, $CDCl_3$, 298 K)

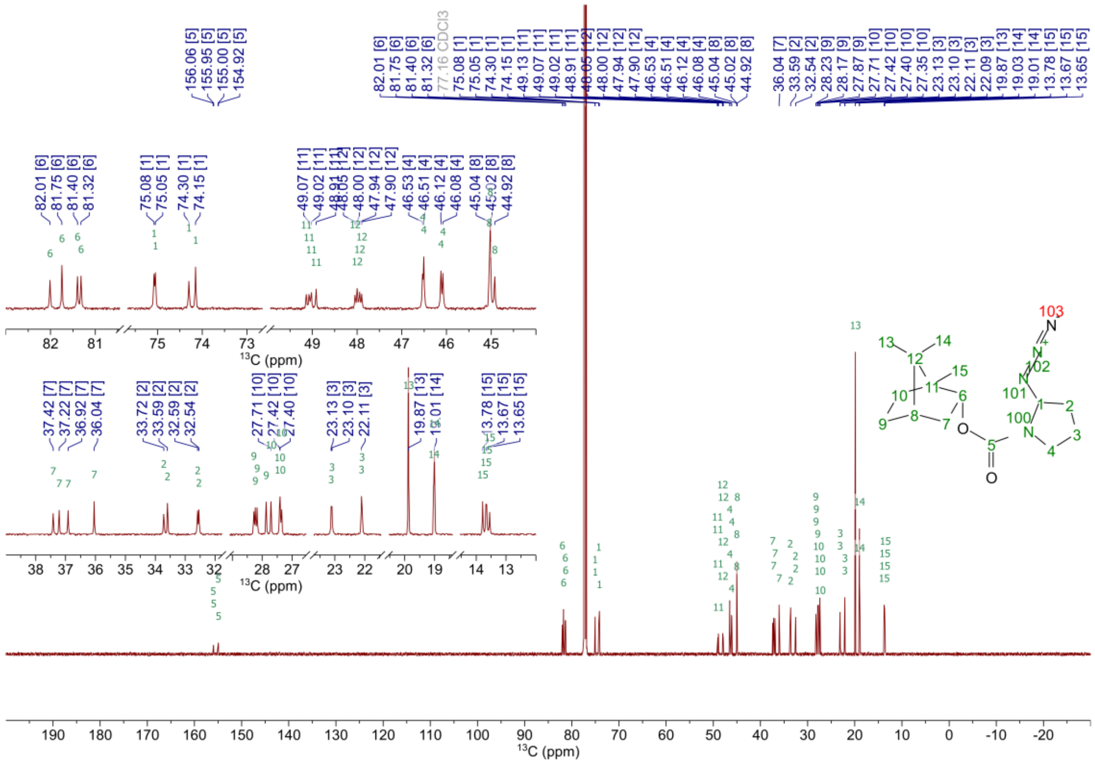

Compound **9s**: ^{1}H-^{13}C HSQC ($CDCl_3$, 298 K)

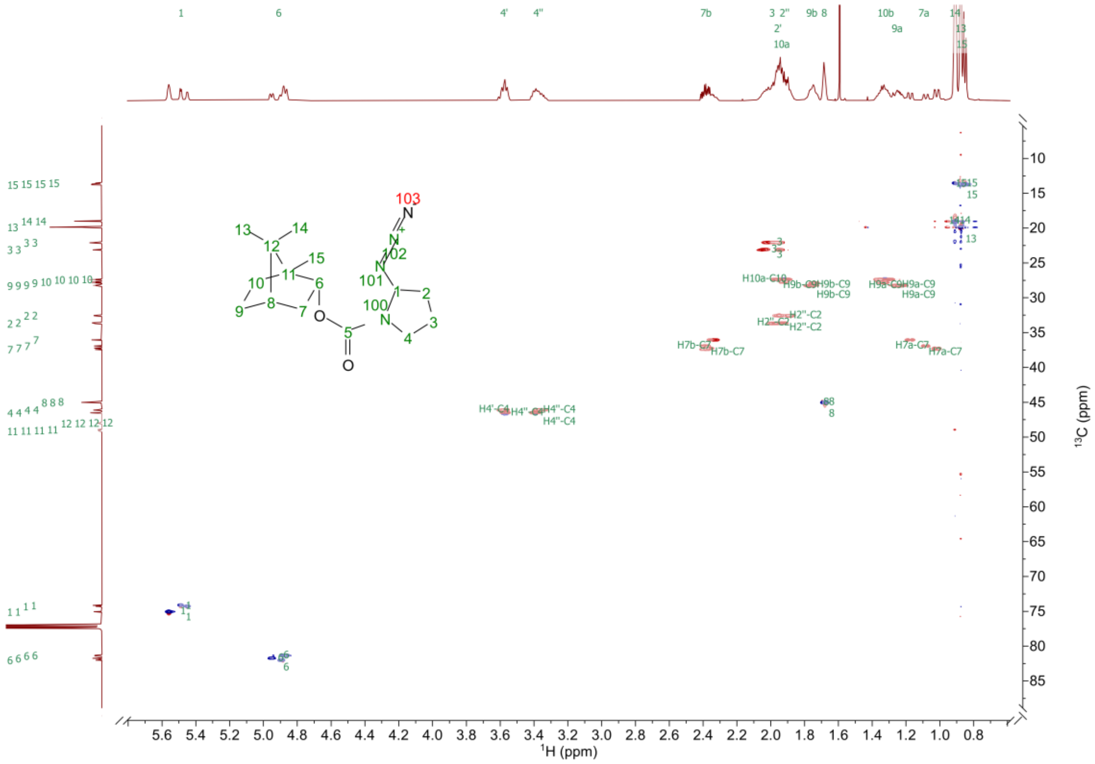

Compound **9s**: ^{1}H-^{13}C HMBC ($CDCl_3$, 298 K)

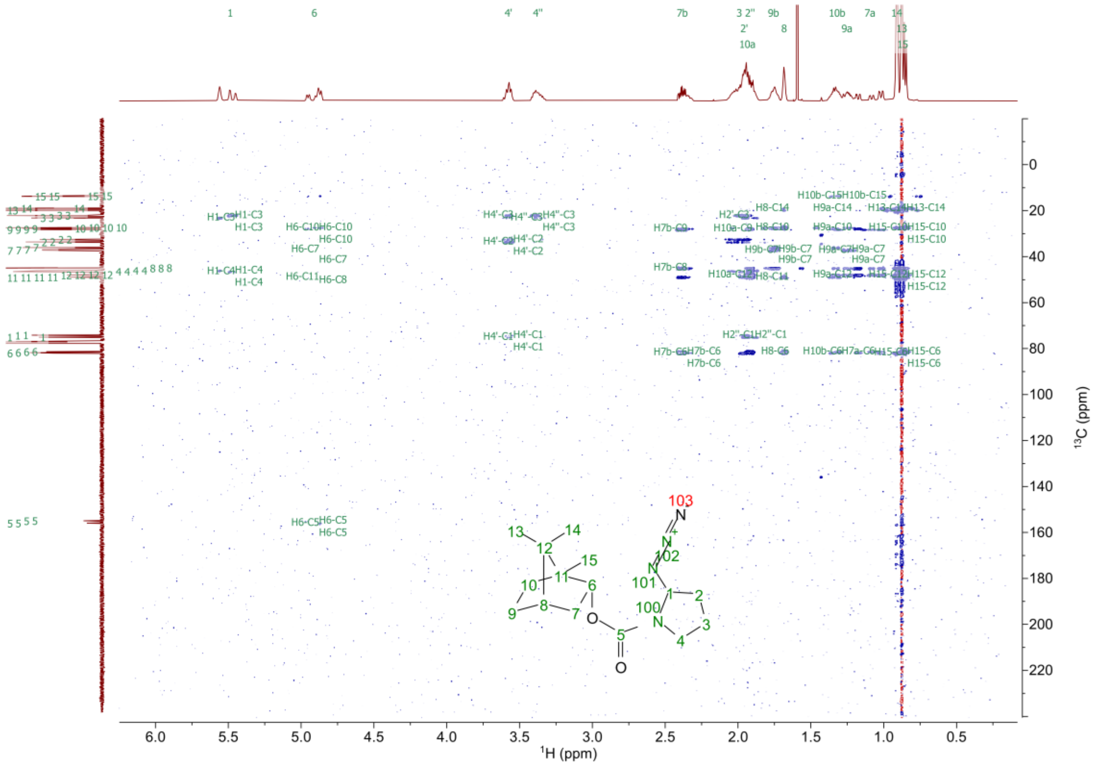

Compound **9s**: ^{1}H-^{1}H COSY ($CDCl_3$, 298 K)

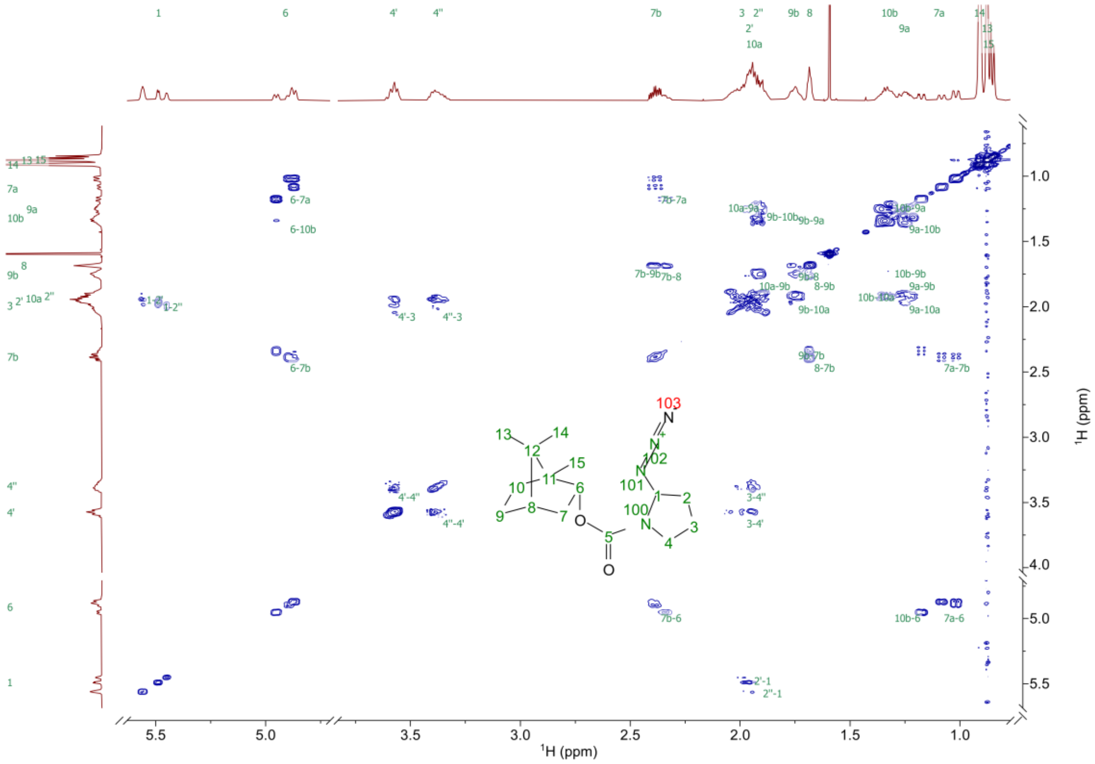

Compound **9s**: ^{1}H-^{1}H NOESY ($CDCl_3$, 298 K)

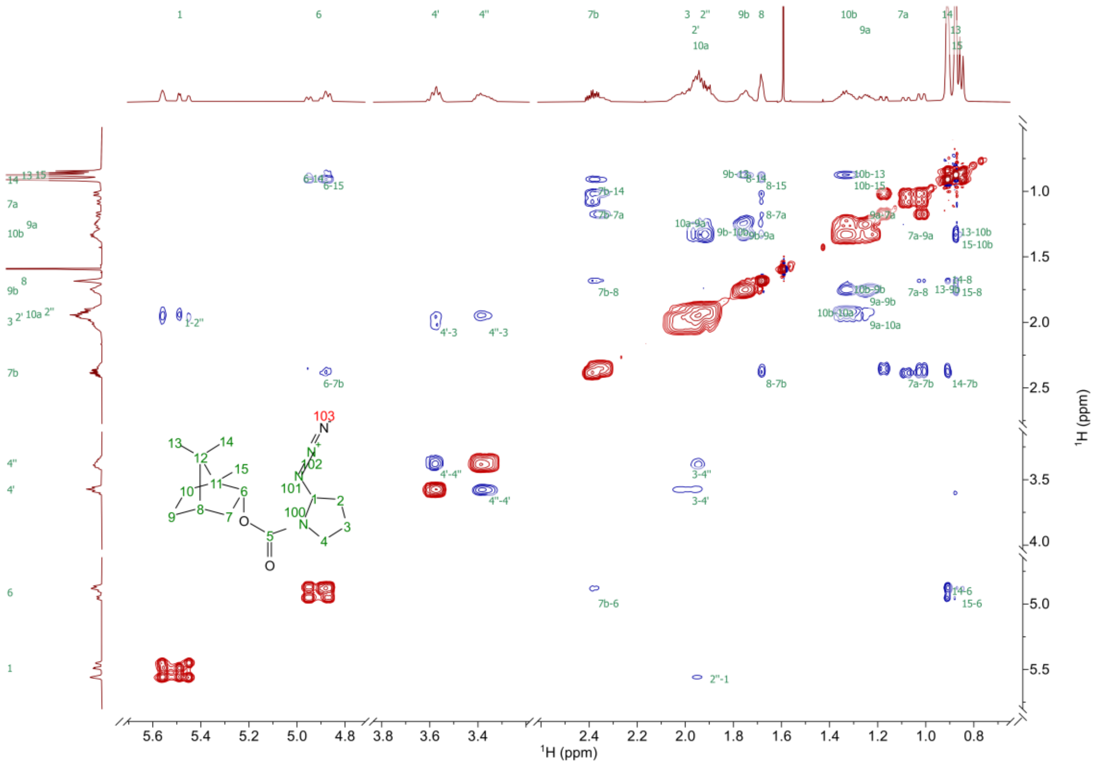

Compound **9s**: ^{1}H-^{15}N HMBC ($CDCl_3$, 298 K)

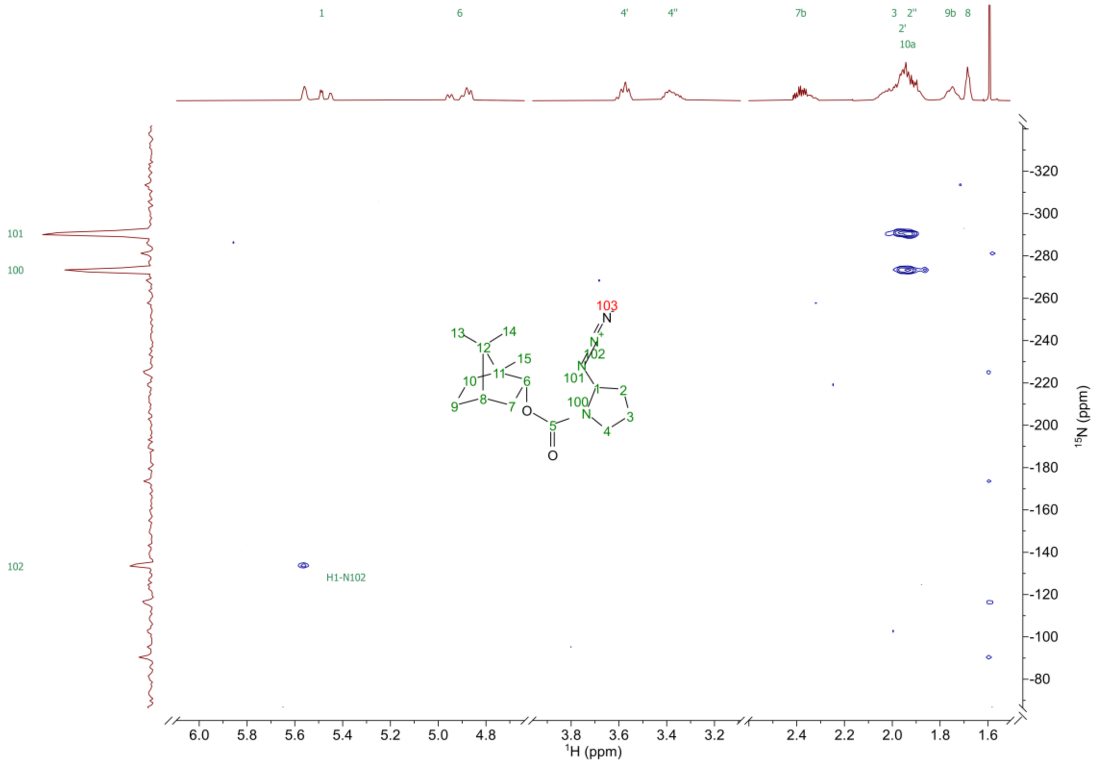

Compound **9t**: ^{1}H NMR (600 MHz, $CDCl_3$, 298 K)

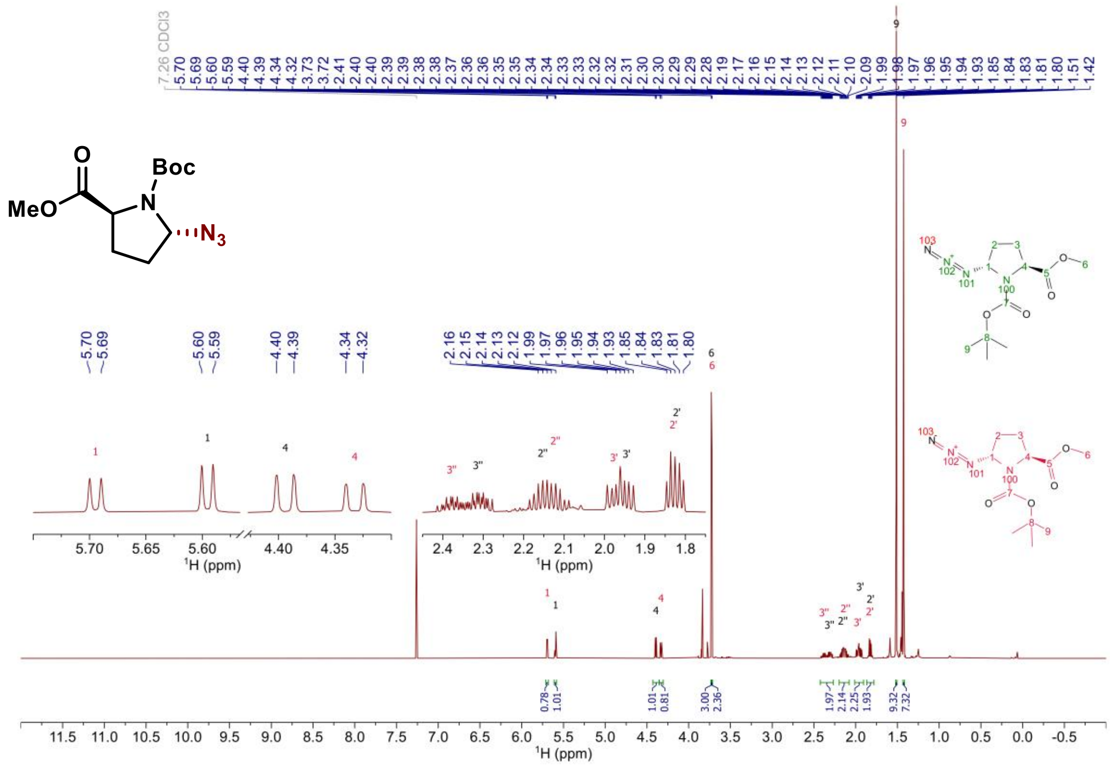

Compound **9t**: ^{13}C NMR (151 MHz, $CDCl_3$, 298 K)

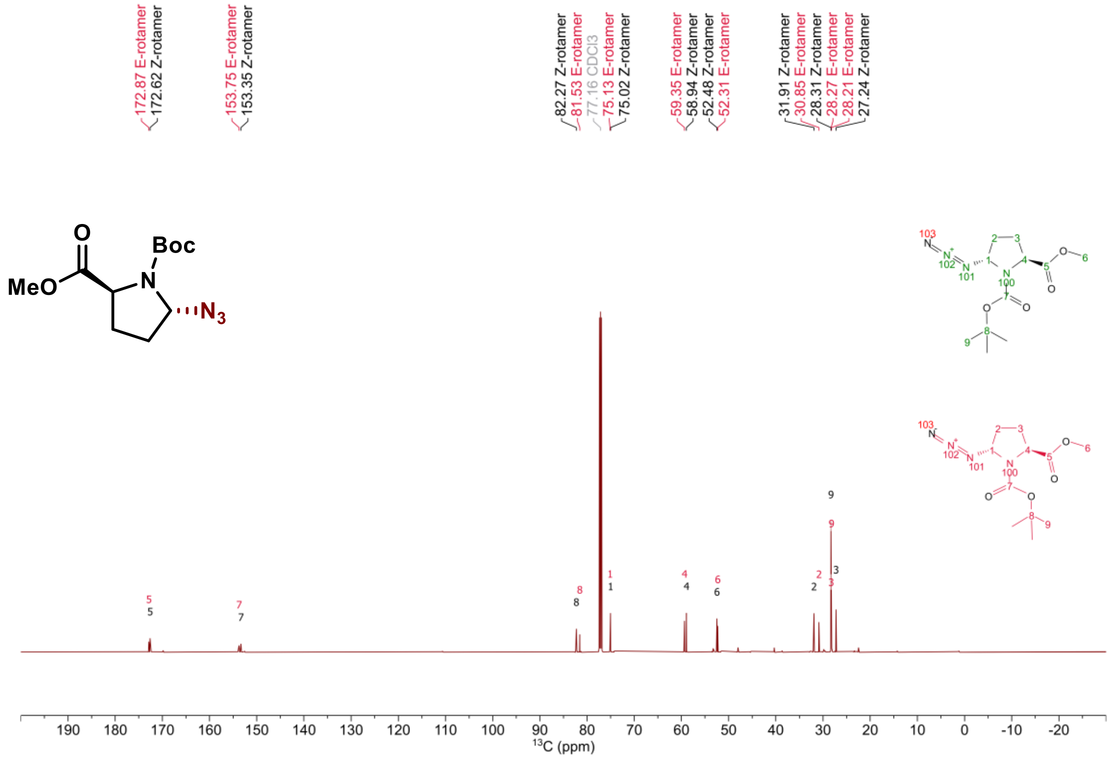

Compound **9t**: ^{1}H-^{13}C HSQC ($CDCl_3$, 298 K)

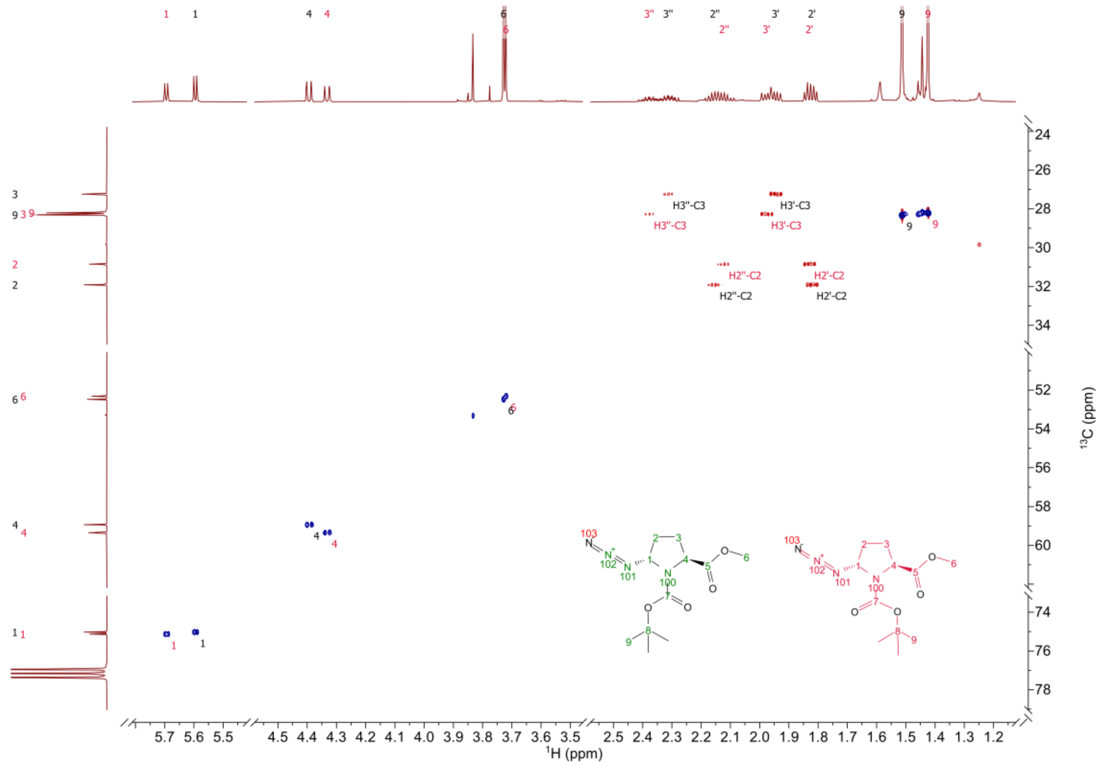

Compound **9t**: ^{1}H-^{13}C HMBC ($CDCl_3$, 298 K)

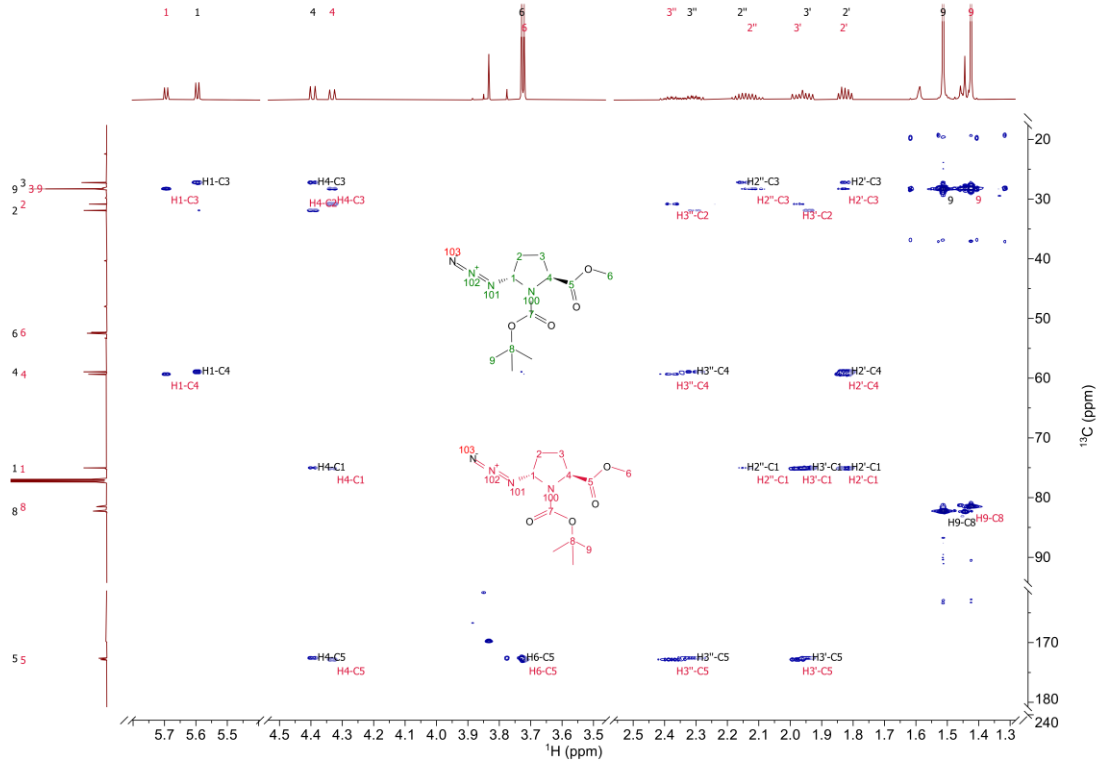

Compound **9t**: ^{1}H-^{1}H COSY ($CDCl_3$, 298 K)

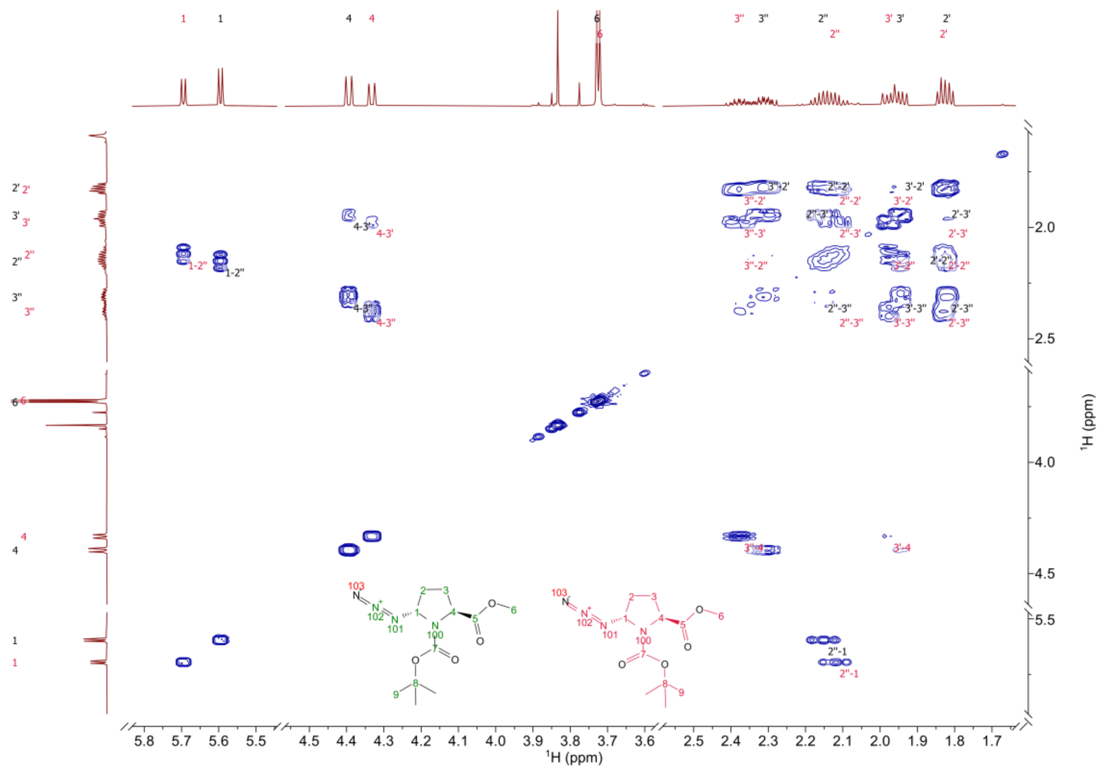

Compound **9t**: ^{1}H-^{1}H NOESY ($CDCl_3$, 298 K)

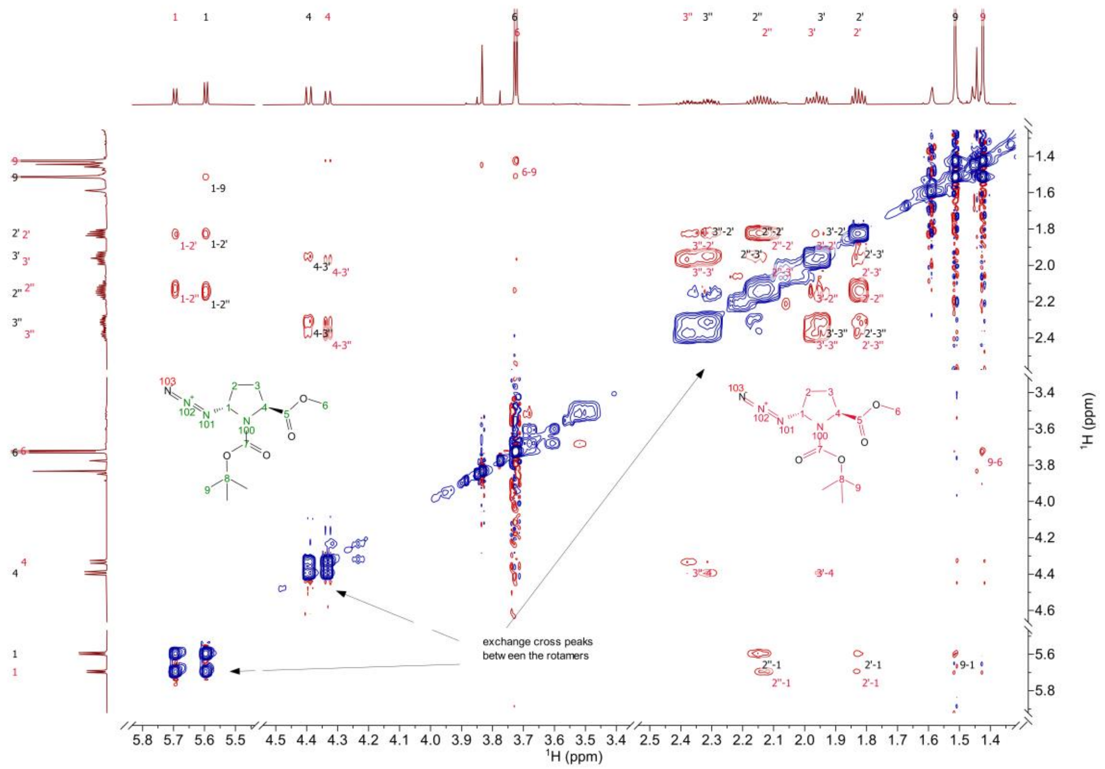

Compound **9t**: ^{1}H-^{15}N HMBC ($CDCl_3$, 298 K)

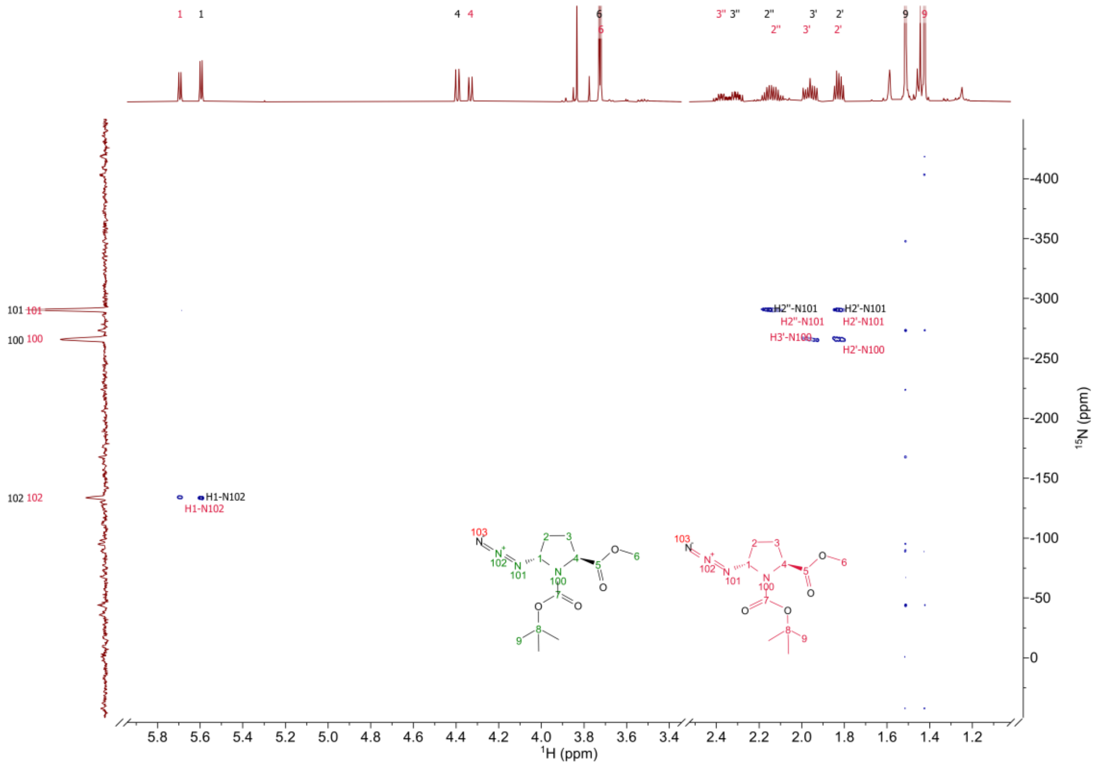

Compound **9t**: 1D selective TOCSY

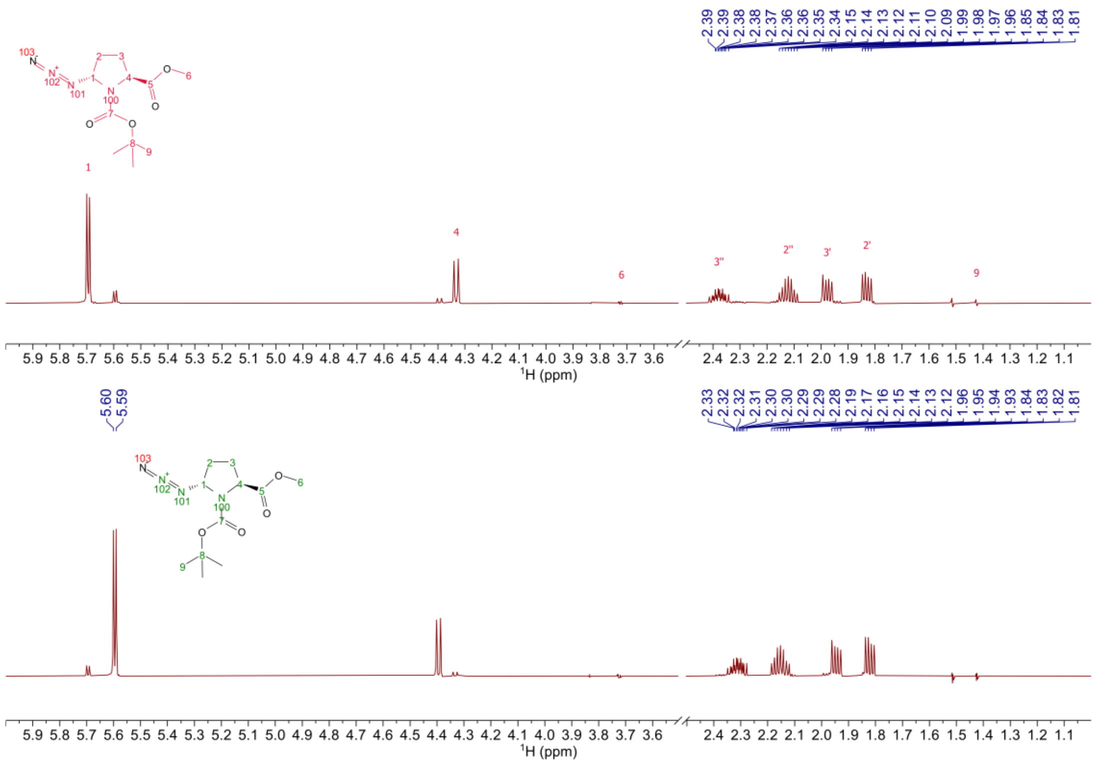

Compound **9u**: ^{1}H NMR (600 MHz, $CDCl_3$, 298 K)

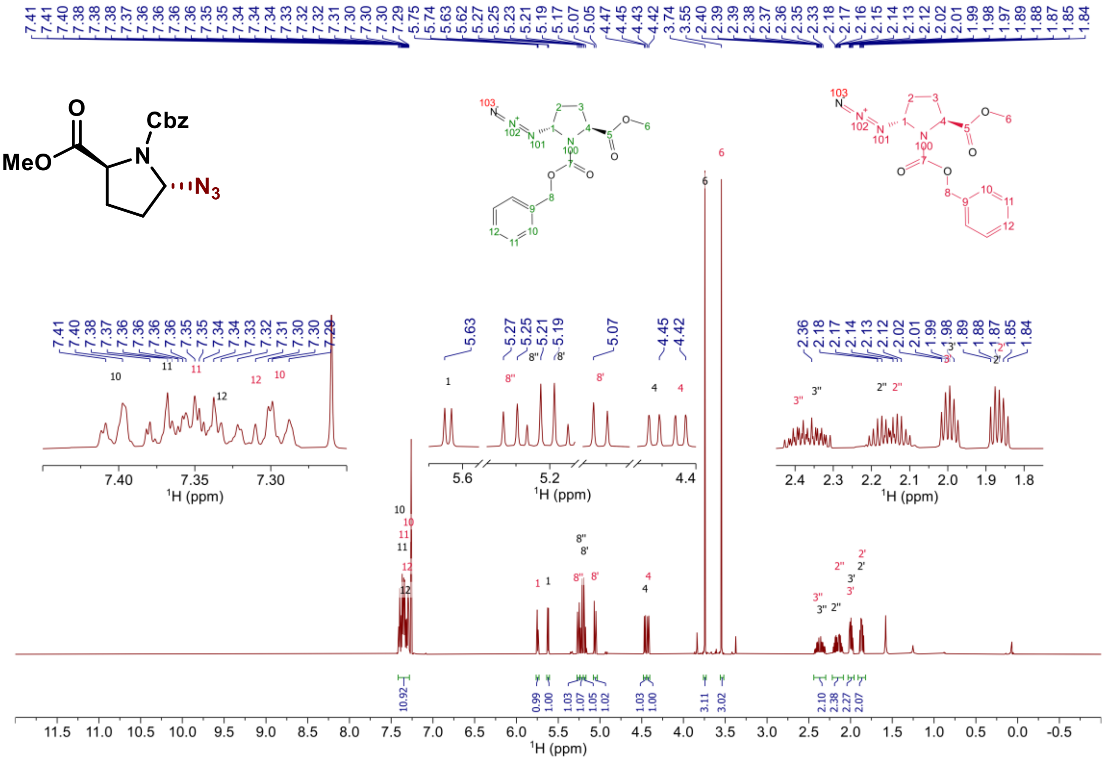

Compound **9u**: ^{13}C NMR (151 MHz, $CDCl_3$, 298 K)

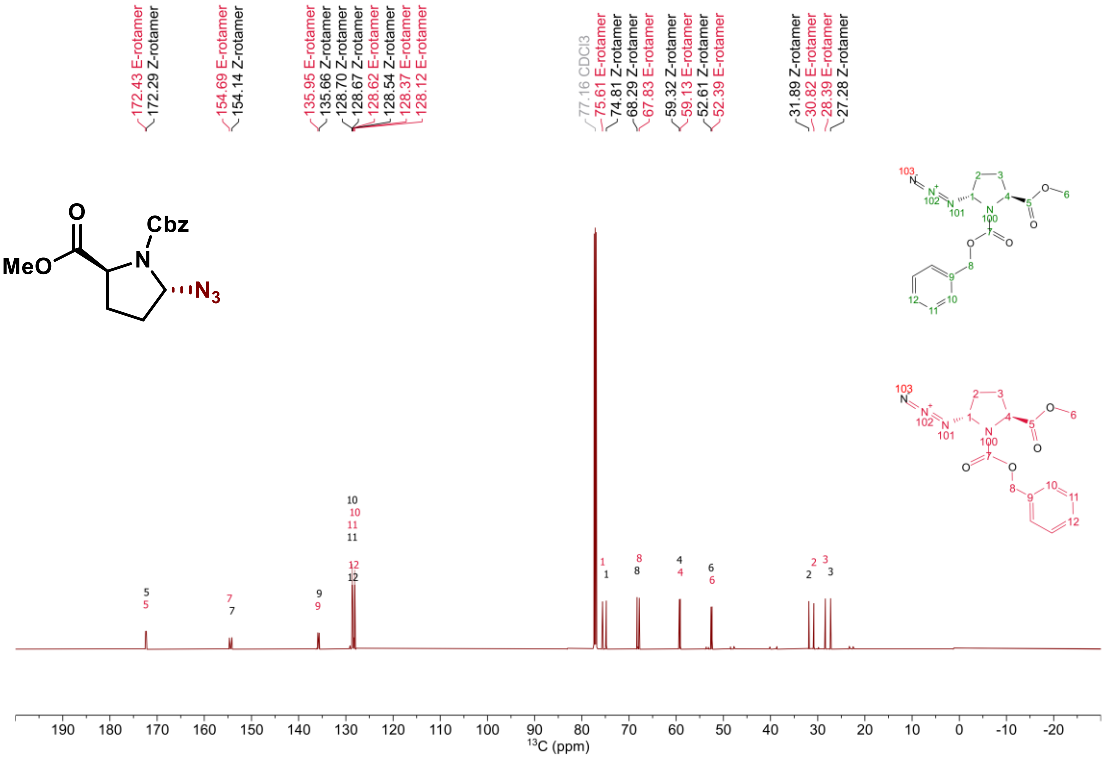

Compound **9u**: ^{1}H-^{13}C HSQC ($CDCl_3$, 298 K)

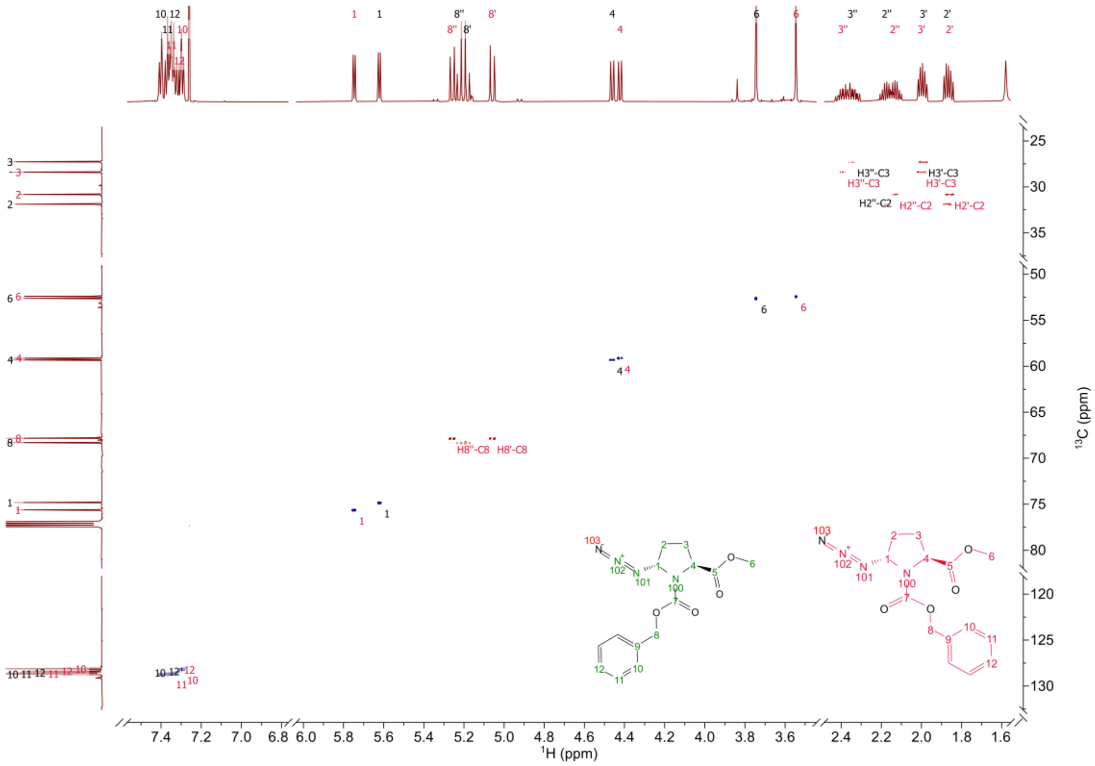

Compound **9u**: ^{1}H-^{13}C HMBC ($CDCl_3$, 298 K)

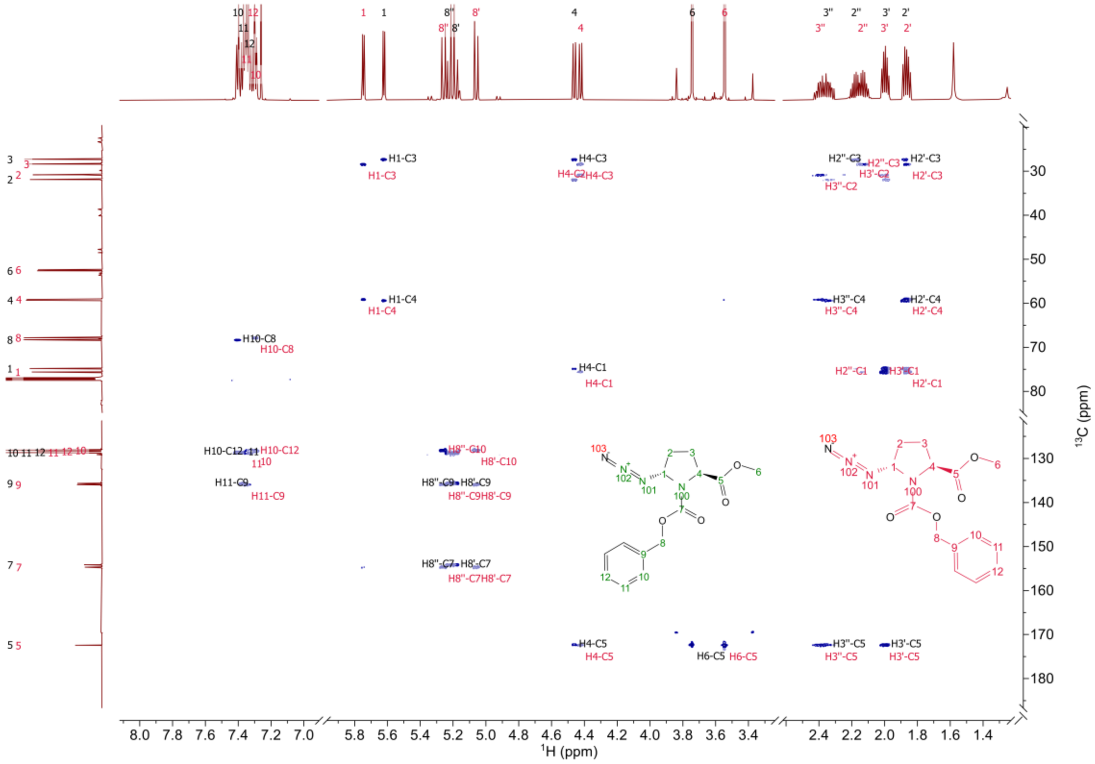

Compound **9u**: ^{1}H-^{1}H COSY ($CDCl_3$, 298 K)

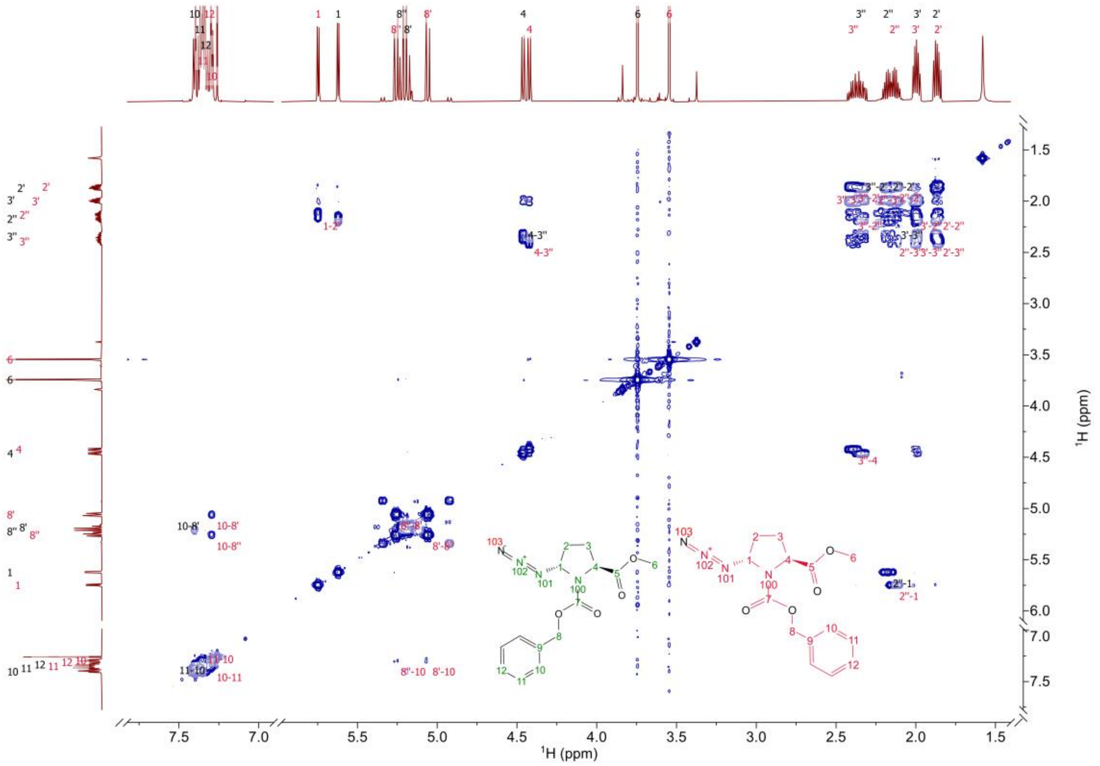

Compound **9u**: ^{1}H-^{1}H NOESY ($CDCl_3$, 298 K)

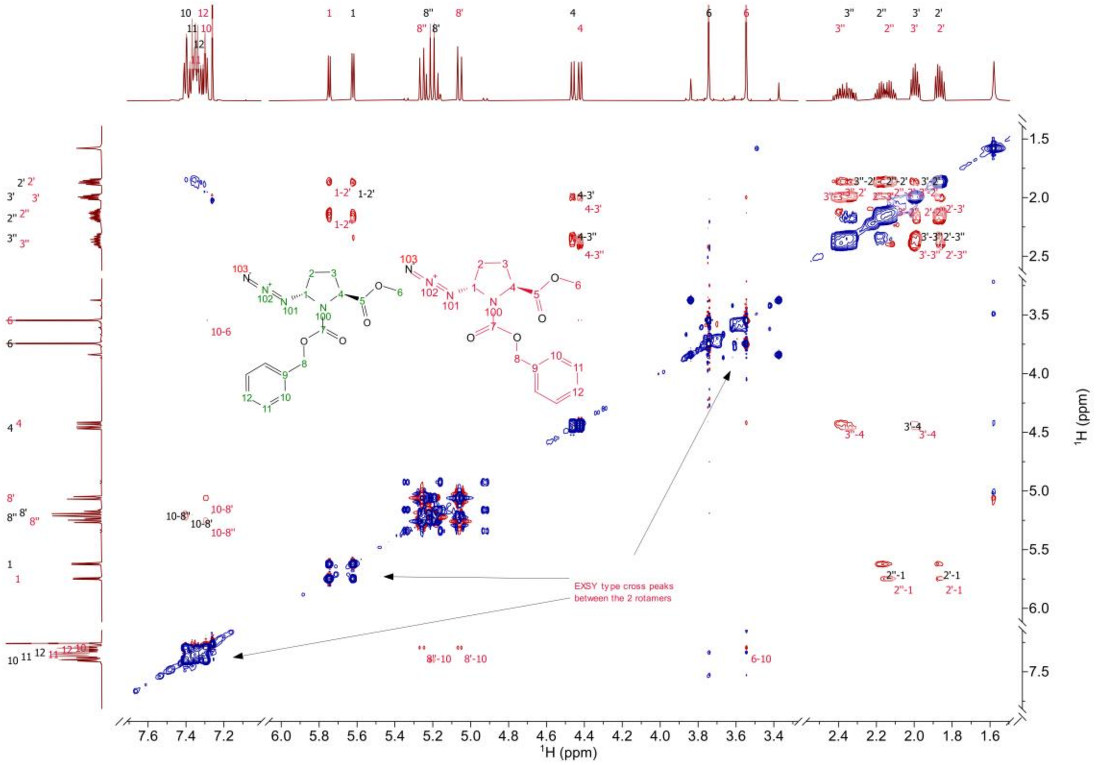

Compound **9u**: ^{1}H-^{15}N HMBC ($CDCl_3$, 298 K)

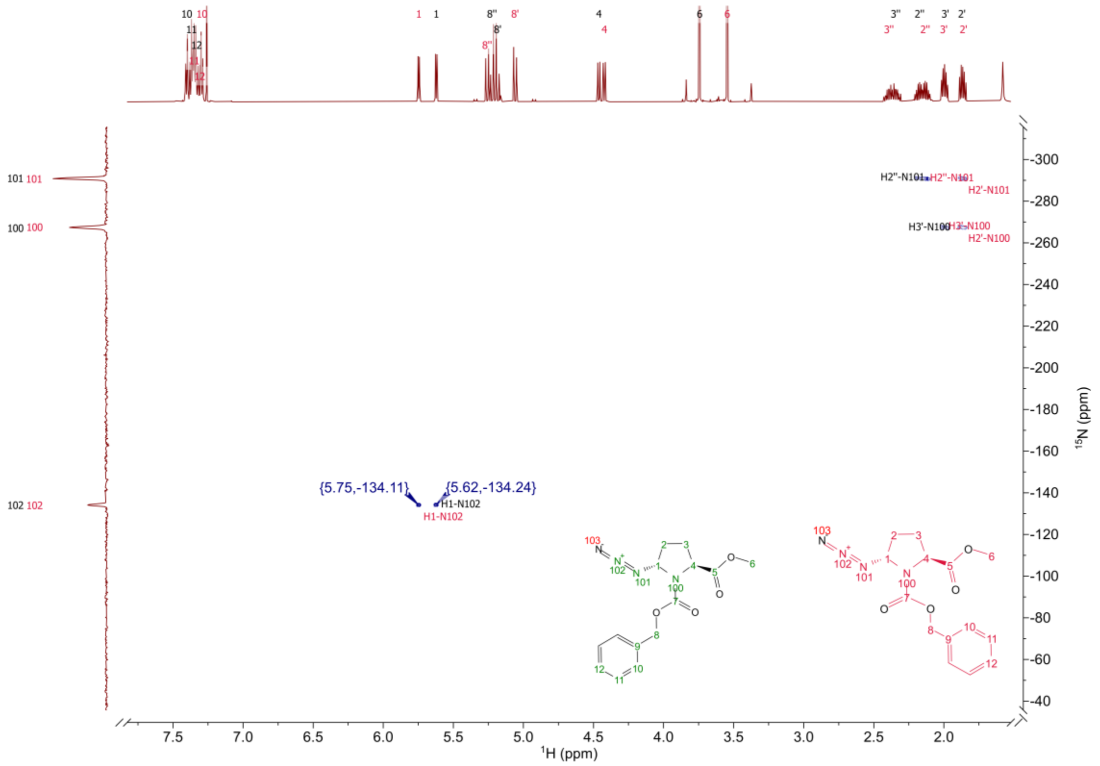

Compound **10a**: ^{1}H NMR (400 MHz, $CDCl_3$, 298 K)

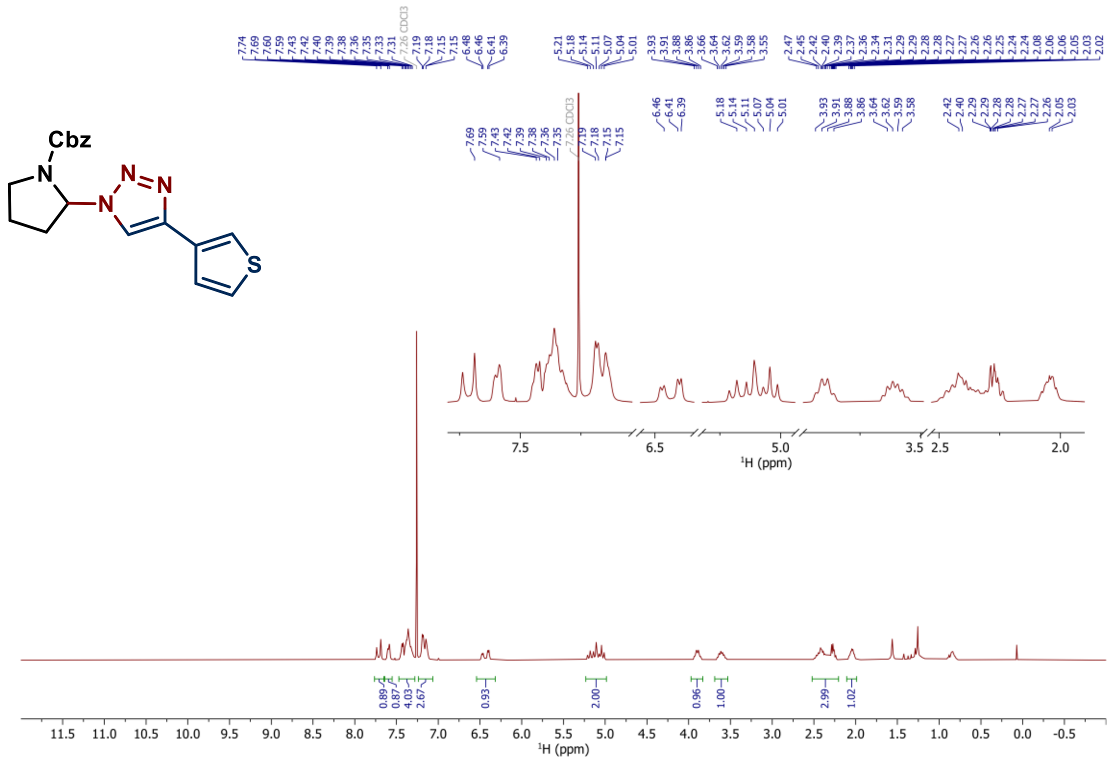

Compound **10a**: ^{13}C NMR (151 MHz, Acetone-$_6$, 298 K)

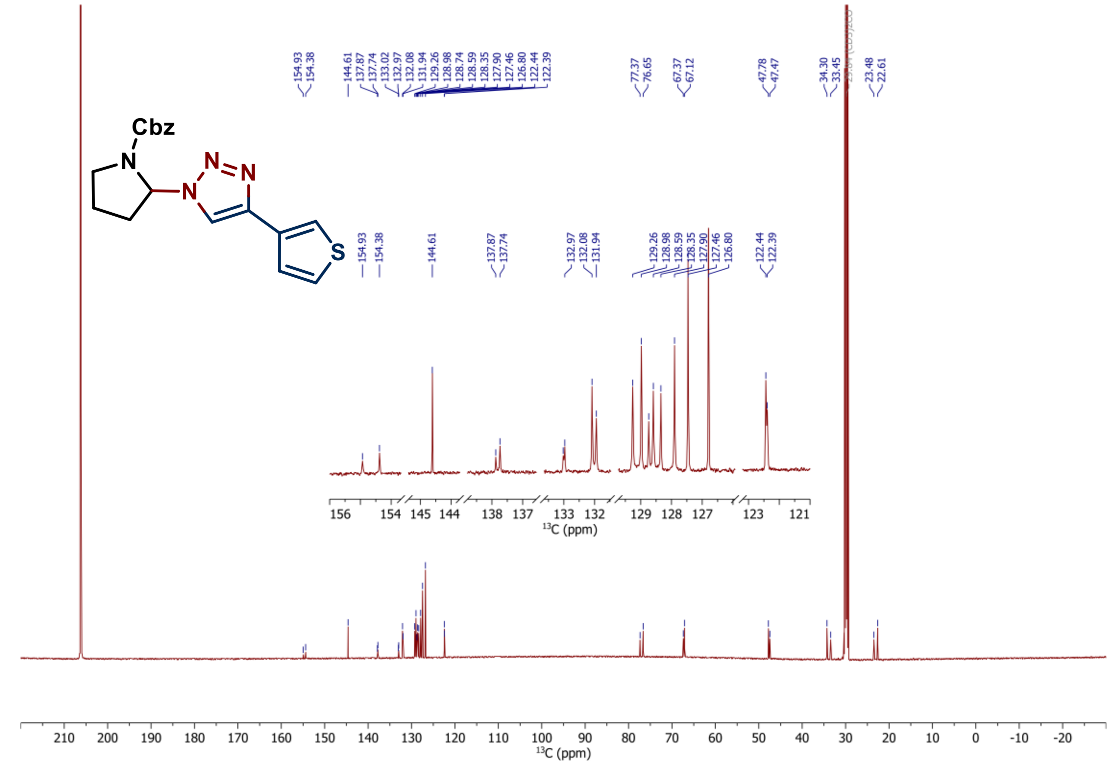

Compound **10b**: ^{1}H NMR (600 MHz, $CDCl_3$, 233 K)

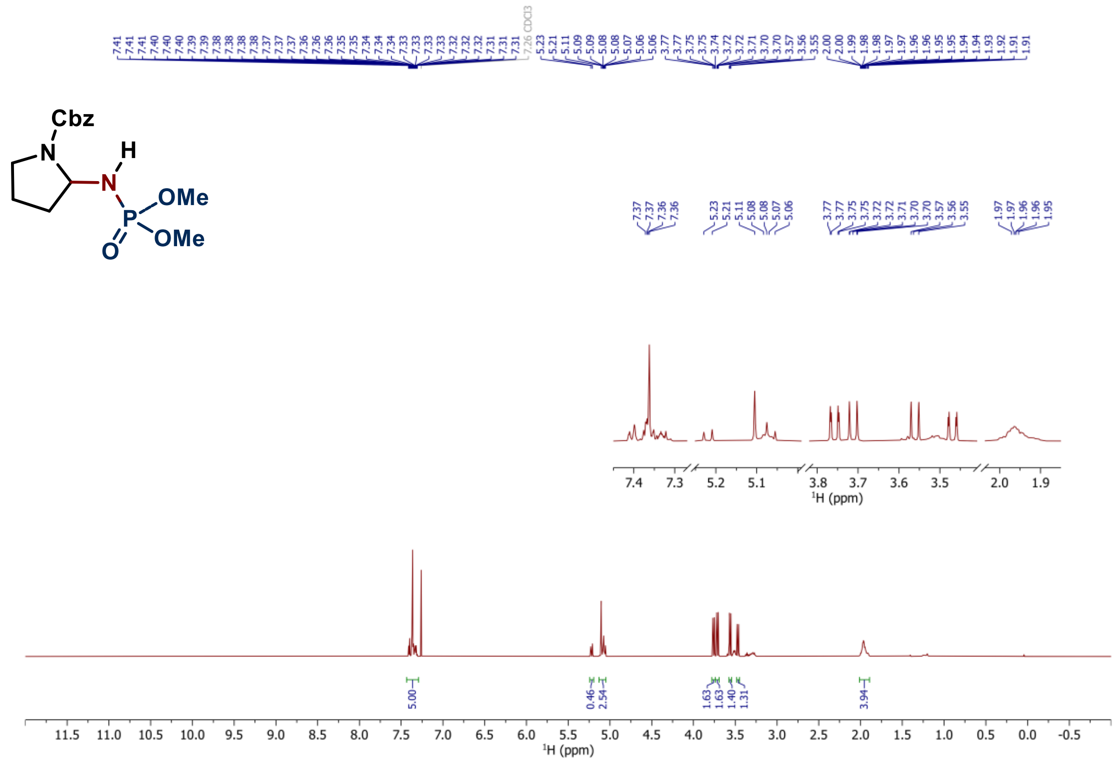

Compound **10b**: ^{13}C NMR (151 MHz, $CDCl_3$, 233 K)

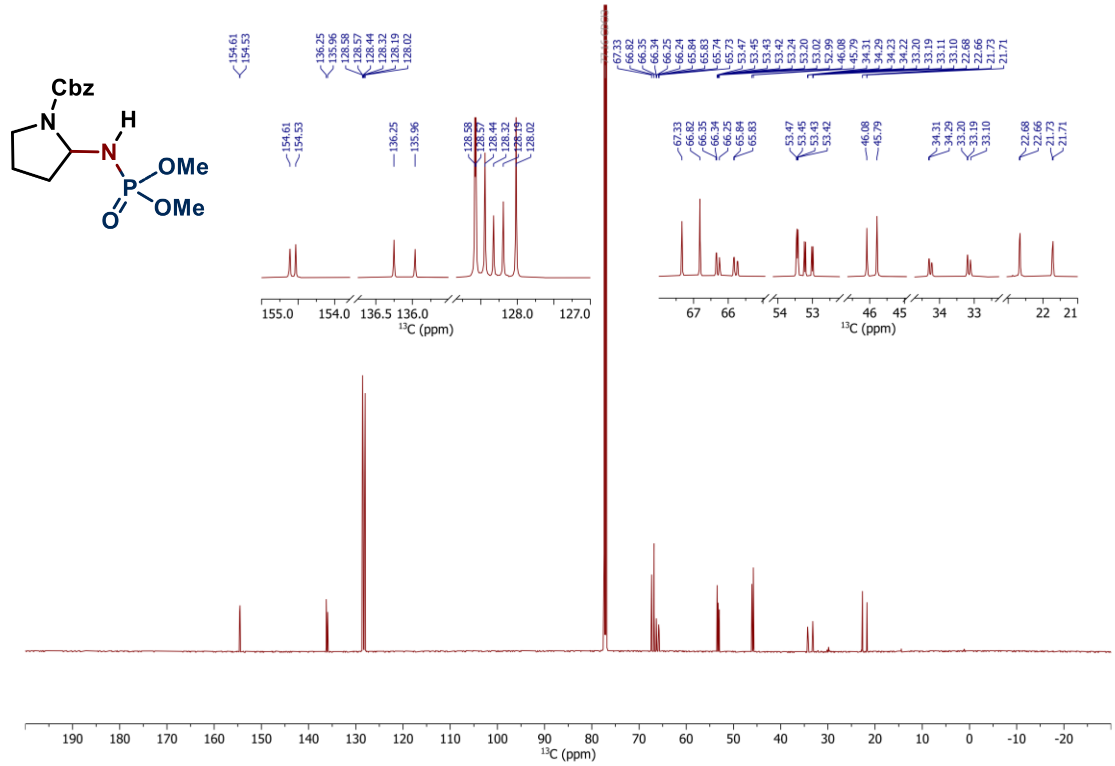

Compound **10b**: $^{31}P\ \{^{1}H\}$ NMR (243 MHz, $CDCl_3$, 233 K)

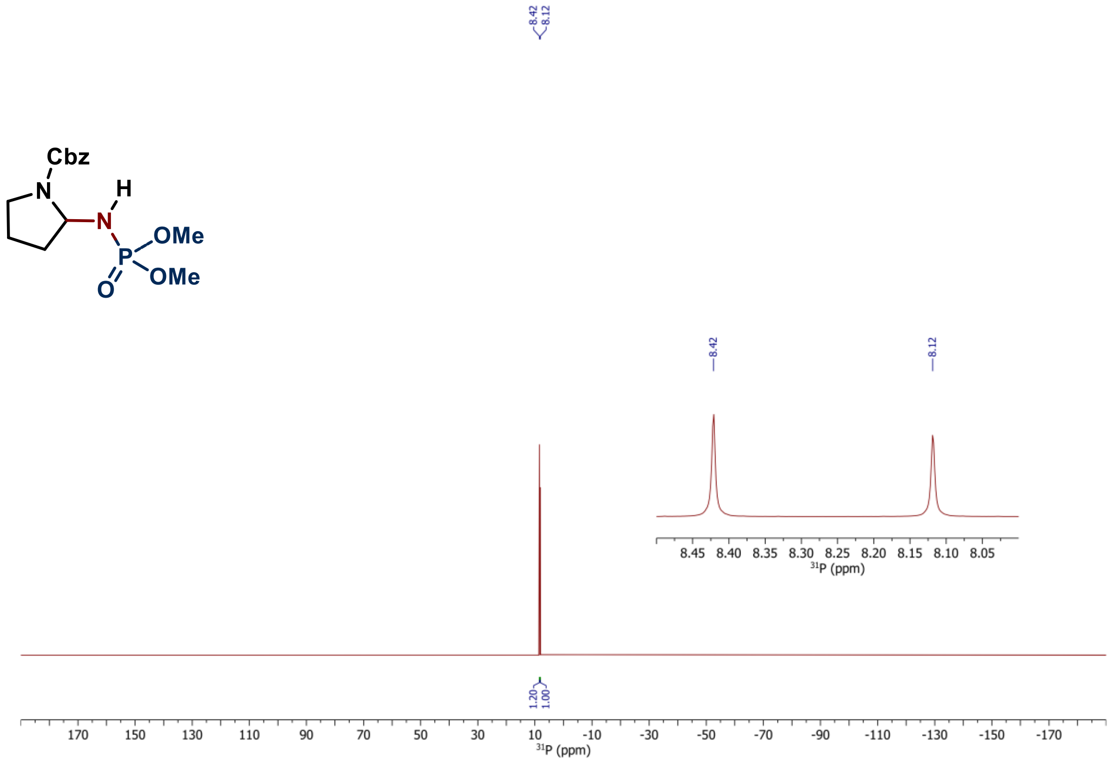

Compound **10c**: ^{1}H NMR (600 MHz, $CDCl_3$, 298 K)

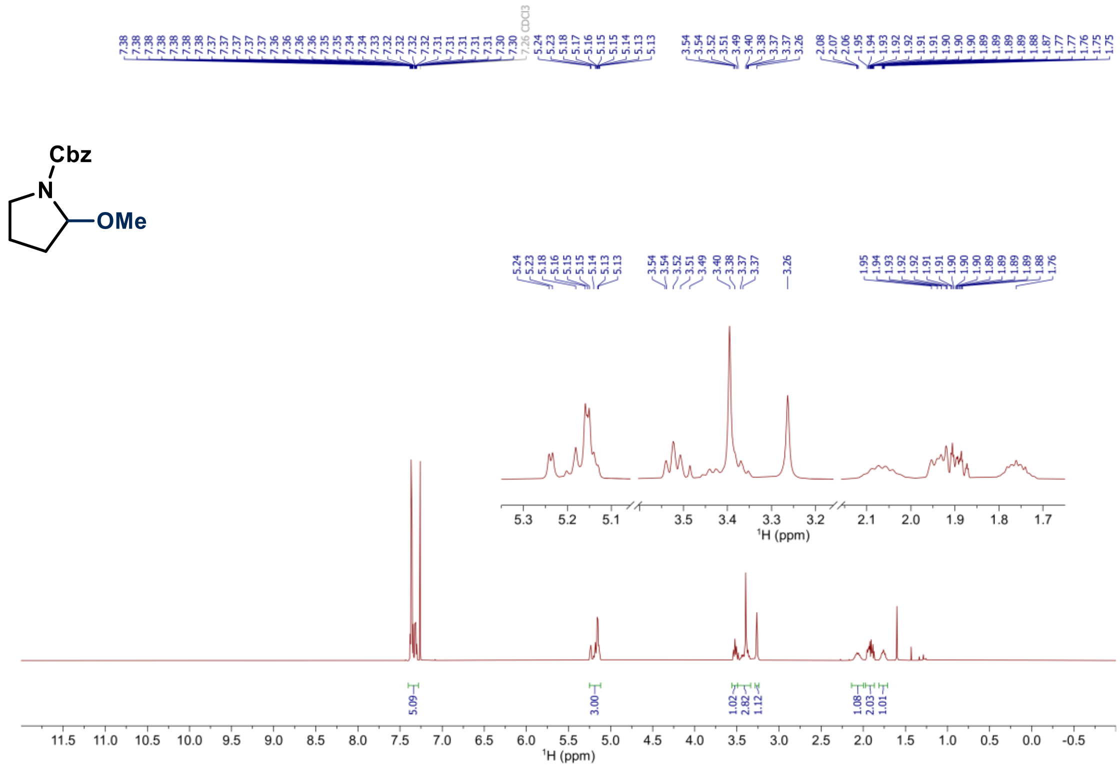

Compound **10c**: ^{13}C NMR (151 MHz, $CDCl_3$, 298 K)

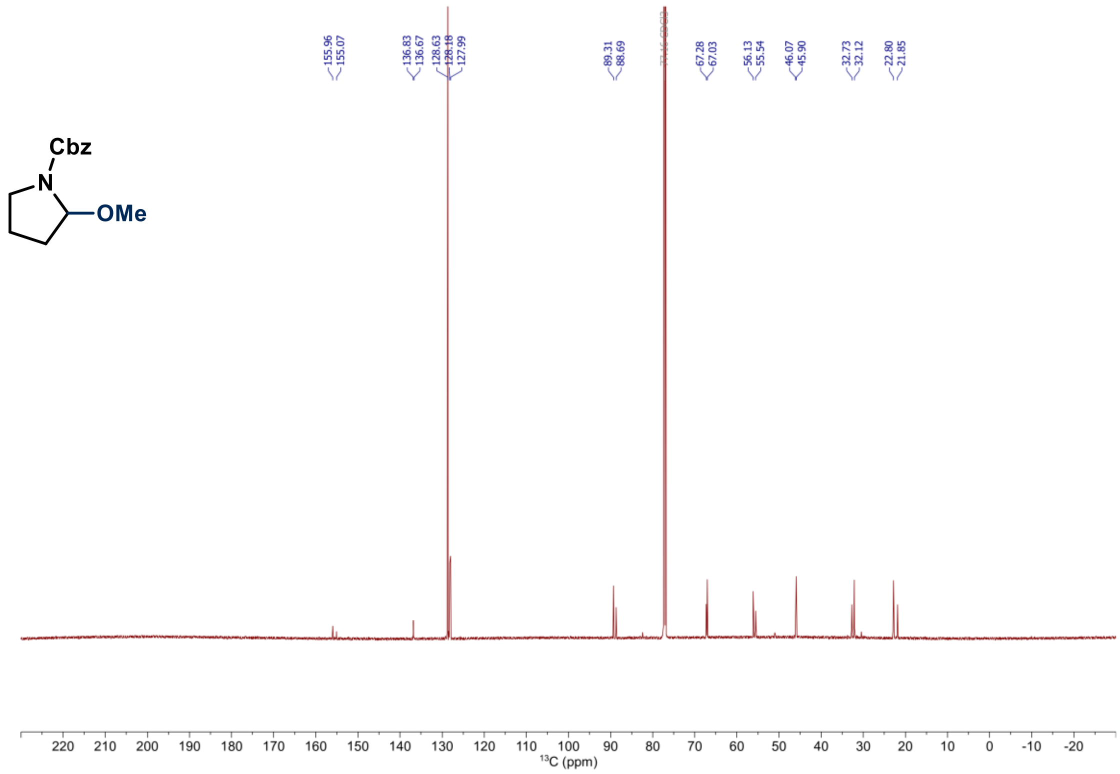

Compound **10d**: ^{1}H NMR (600 MHz, $CDCl_3$, 298 K)

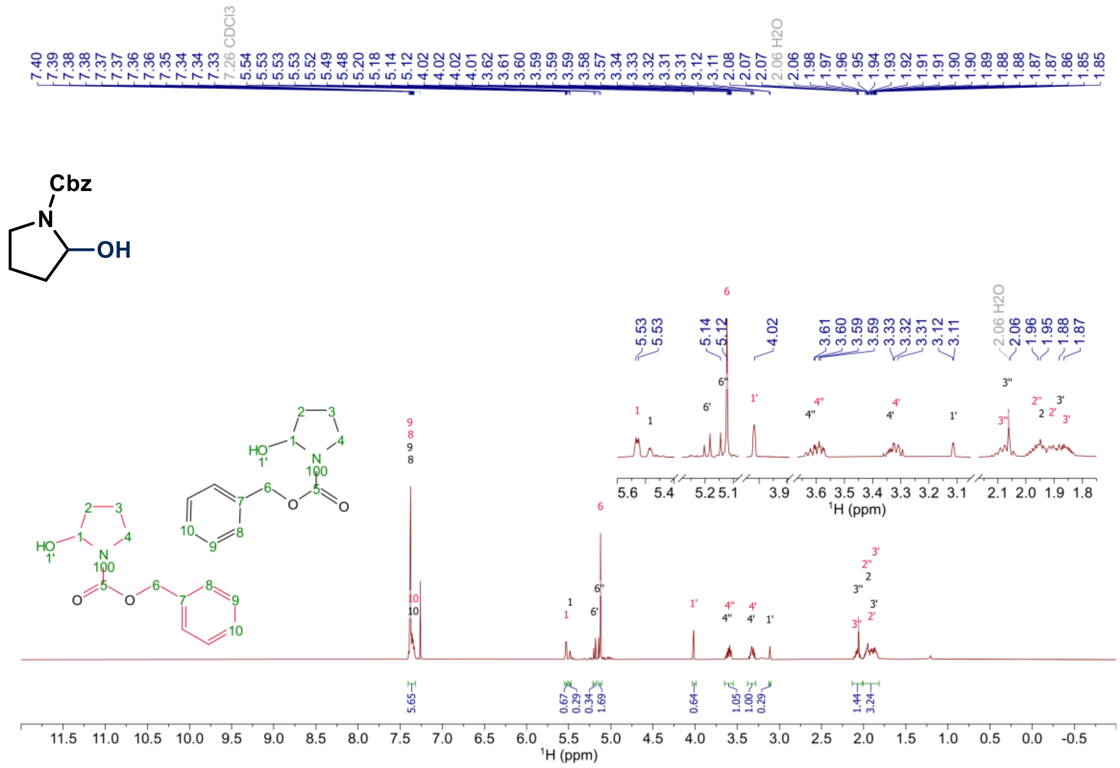

Compound **10d**: ^{13}C NMR (151 MHz, $CDCl_3$, 298 K)

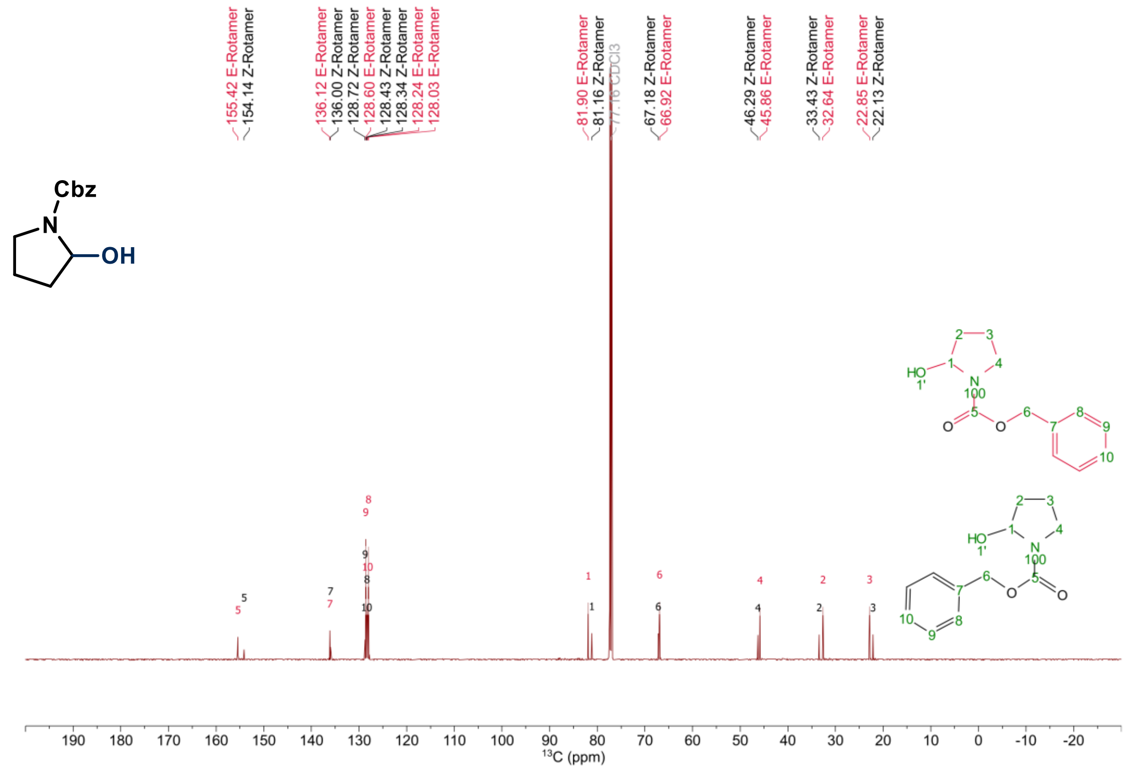

Compound **10d**: variable-temperature ^{1}H NMR (600 MHz, $CDCl_3$)

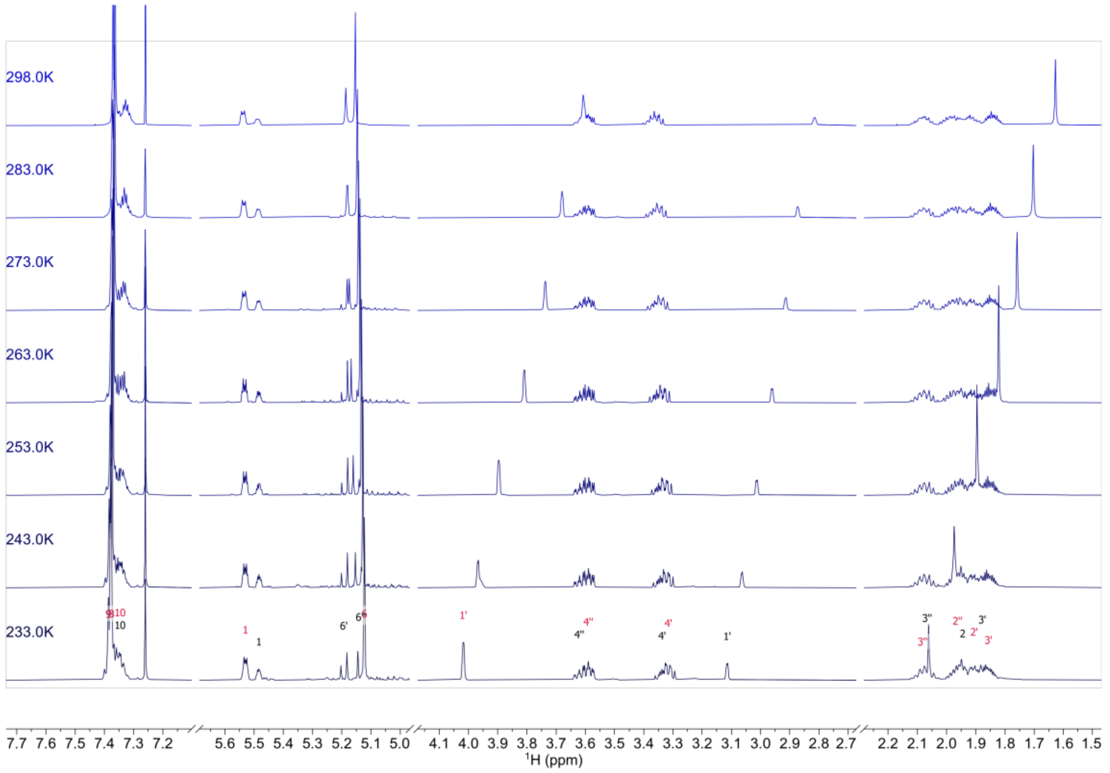

Compound **10d**: ^{1}H-^{13}C HSQC

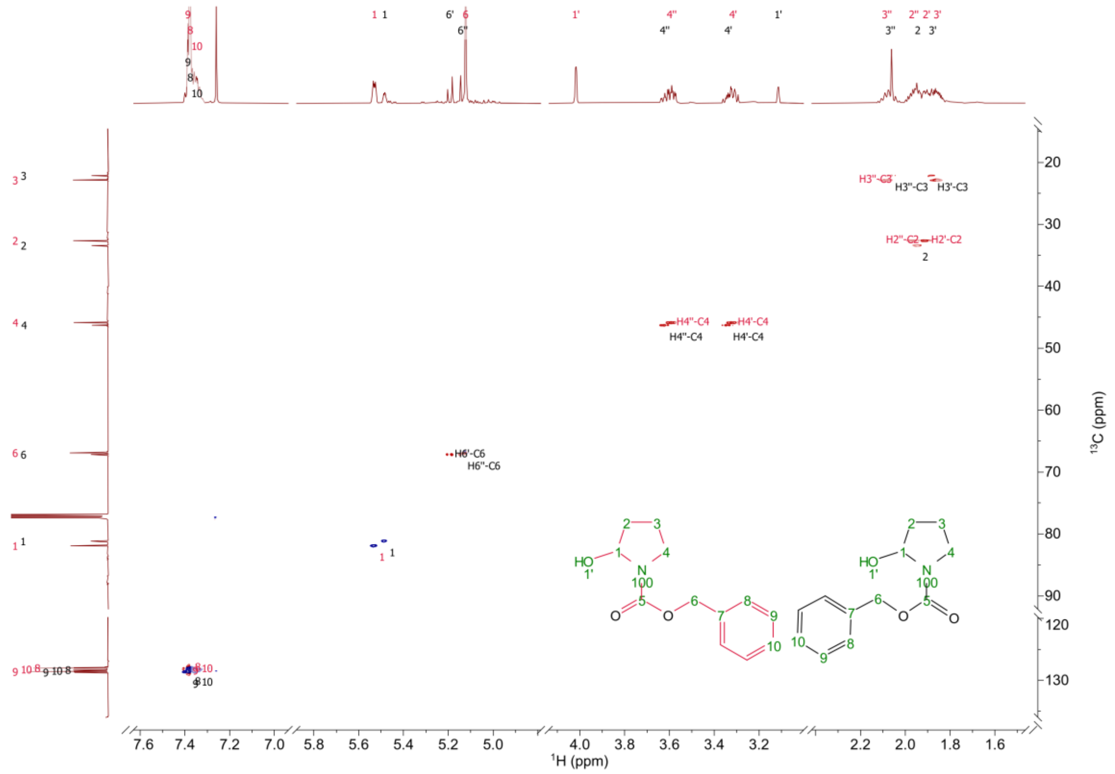

Compound **10d**: ^{1}H-^{13}C HMBC

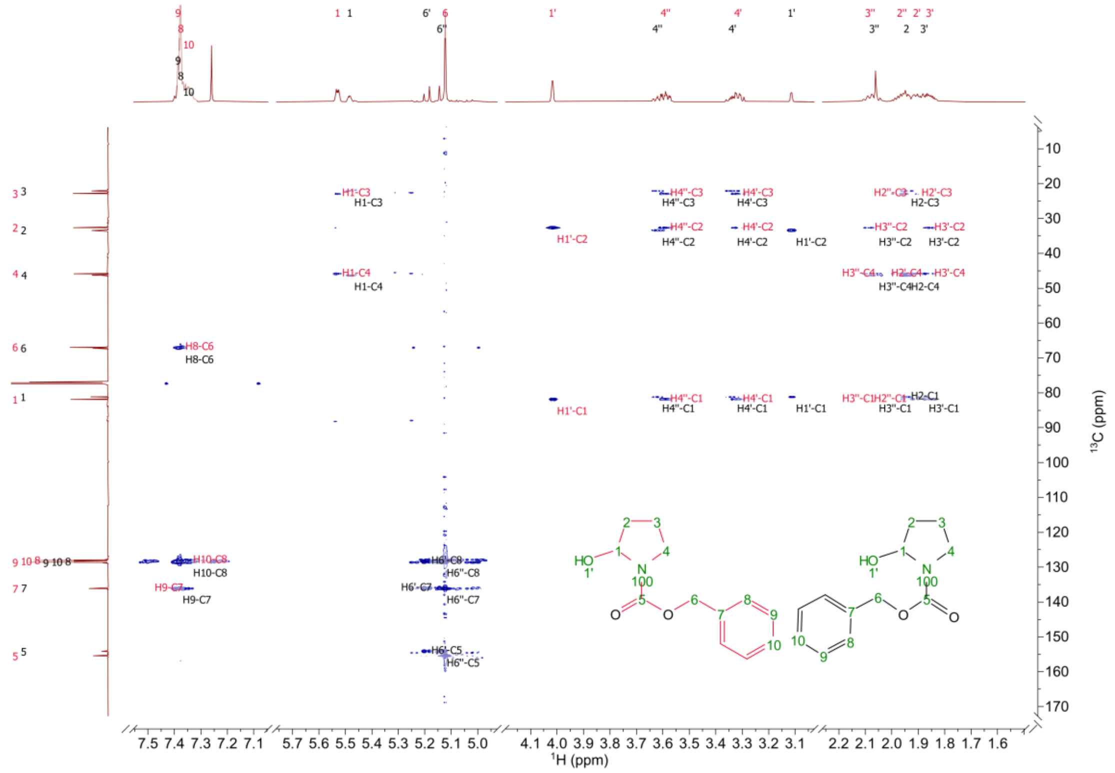

Compound **10d**: ^{1}H-^{1}H COSY

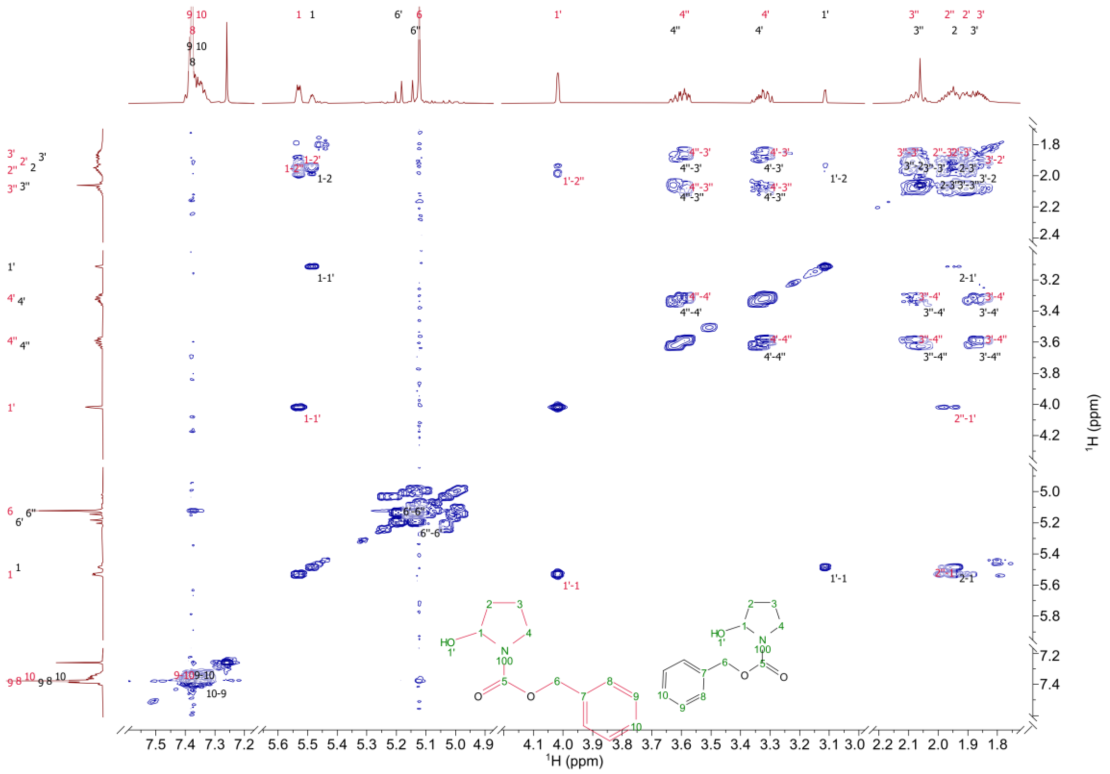

Compound **10d**: ^{1}H-^{1}H NOESY

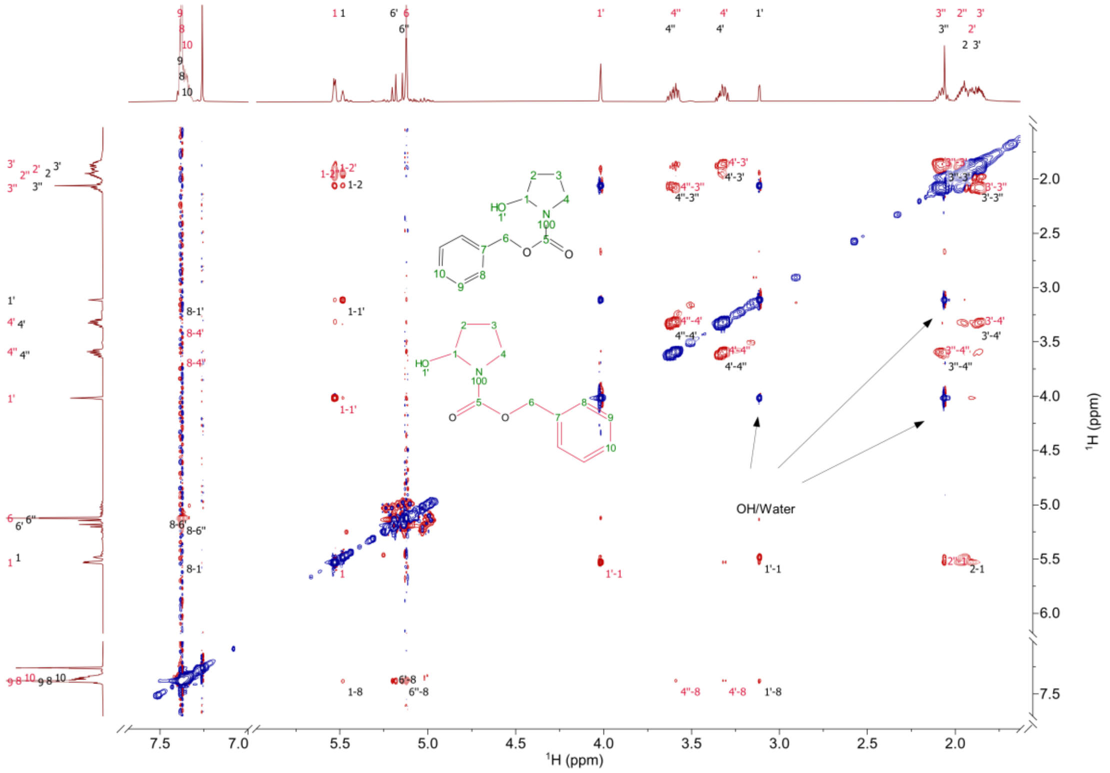

Compound **10d**: ^{1}H-^{15}N HMBC

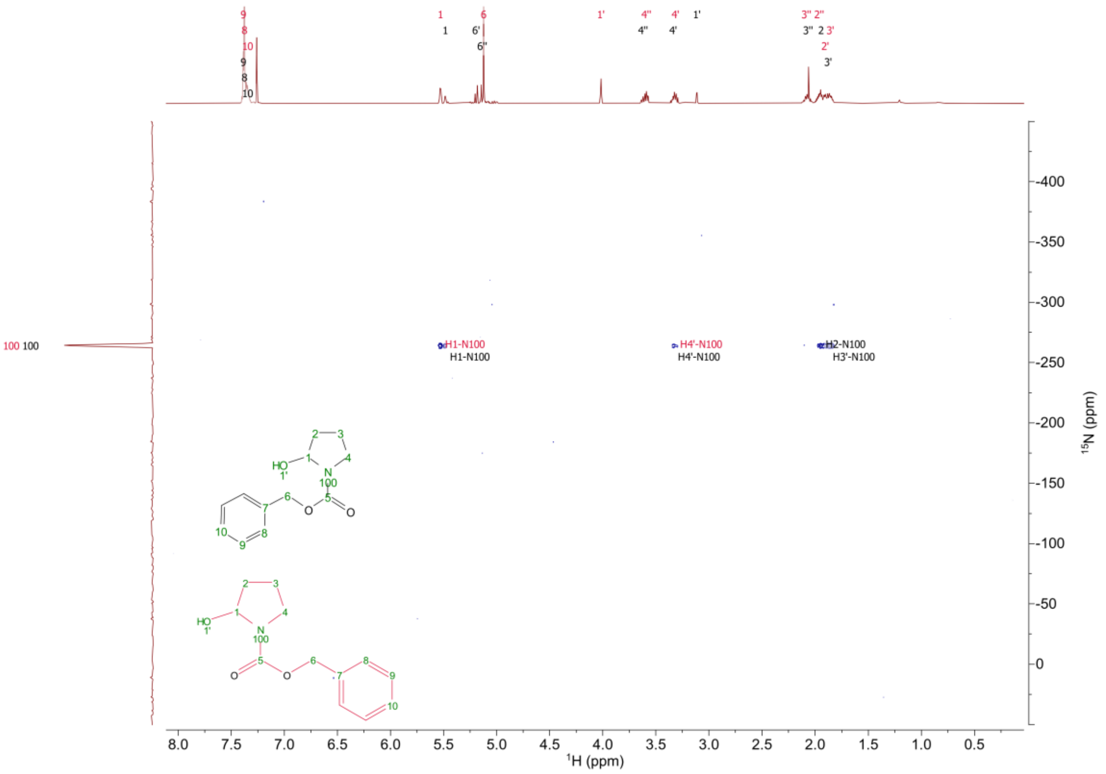

Compound **10d**: 1D selective TOCSY ^{1}H-^{1}H NOESY

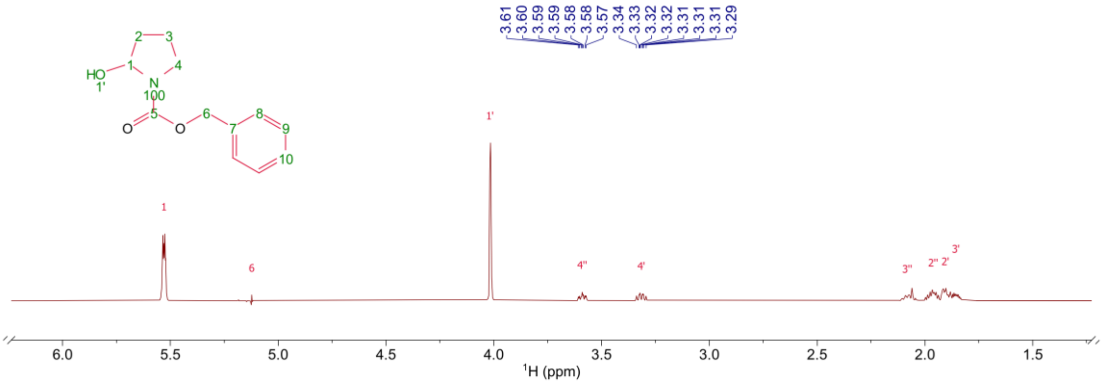

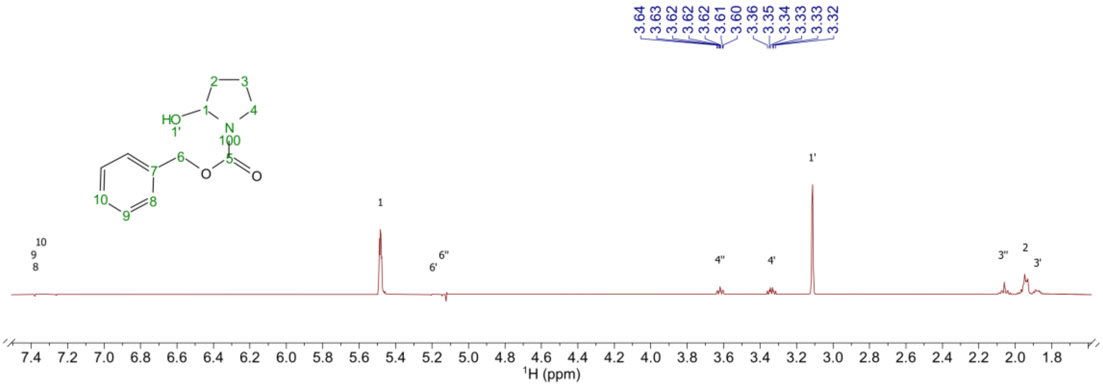

Compound **10e**: ^{1}H NMR (400 MHz, $CDCl_3$, 298 K)

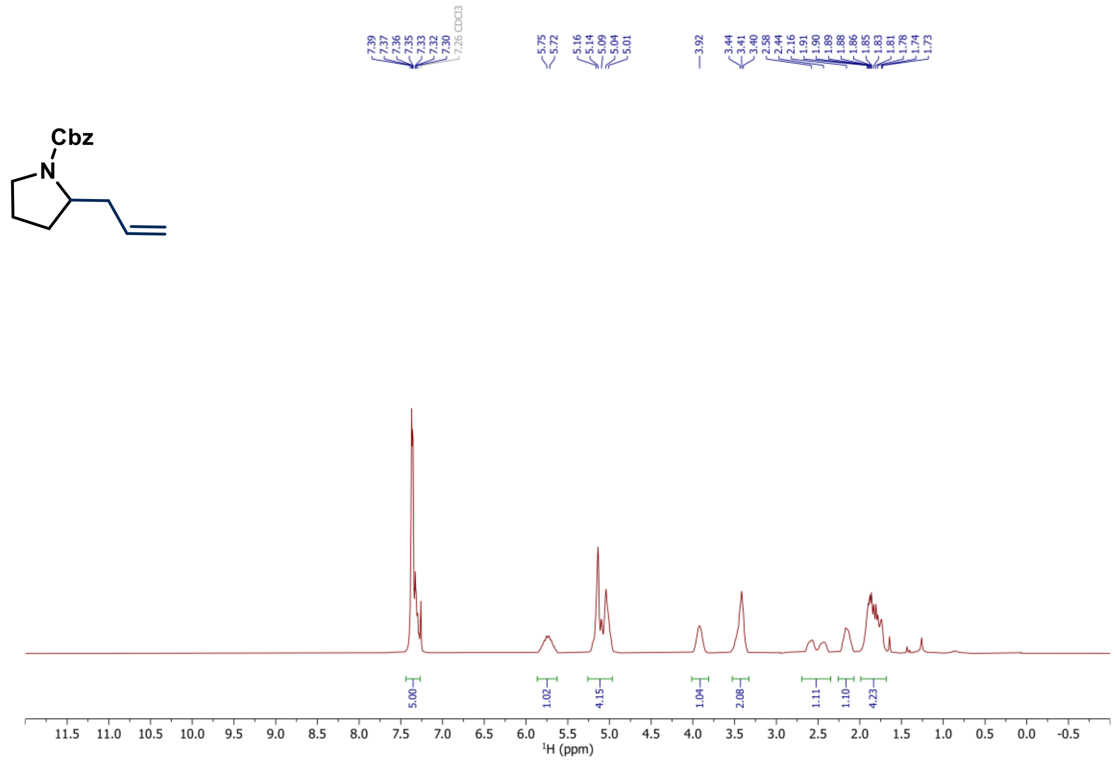

Compound **10e**: ^{13}C NMR (101 MHz, $CDCl_3$, 298 K)

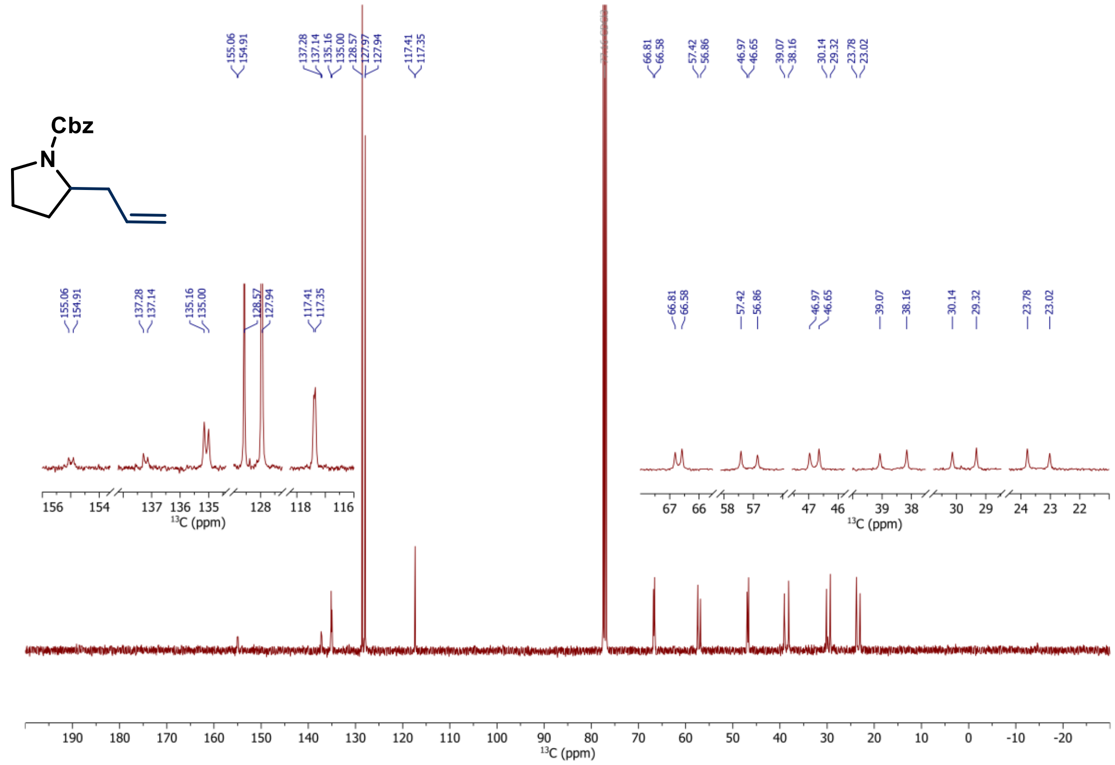

Compound **10f**: ^{1}H NMR (400 MHz, $CDCl_3$, 298 K)

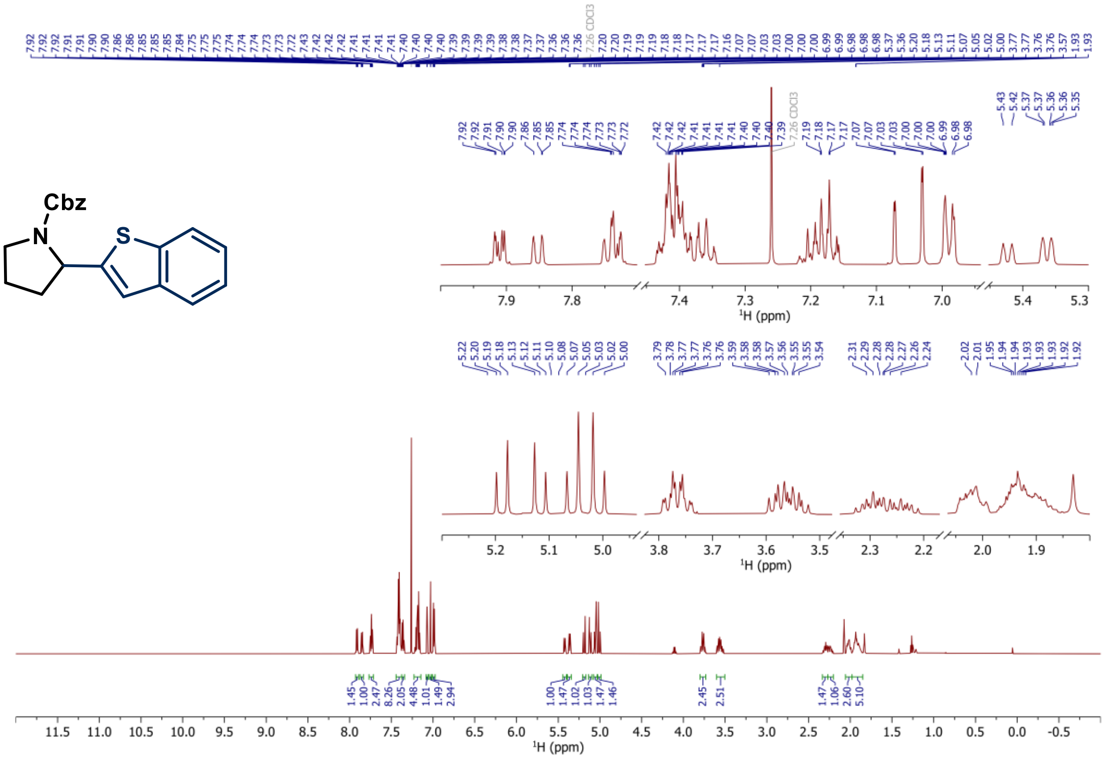

Compound **10f**: ^{13}C NMR (101 MHz, $CDCl_3$, 298 K)

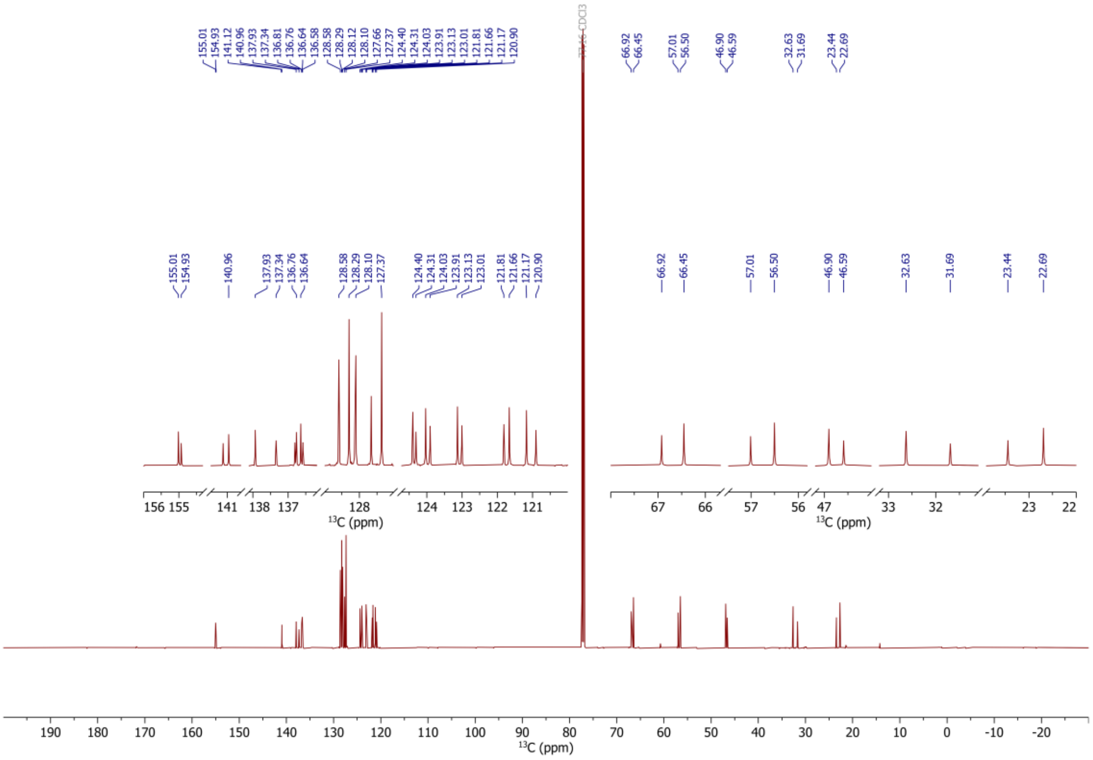

11. References

(1) Stamoulis, A.; Mato, M.; Bruzzese, P. C.; Leutzsch, M.; Cadranel, A.; Gil-Sepulcre, M.; Neese, F.; Cornella, J. Red-Light-Active *N,C,N*-Pincer Bismuthinidene: Excited State Dynamics and Mechanism of Oxidative Addition into Aryl Iodides. *J. Am. Chem. Soc.* **2025**, *147*, 6037–6048.

(2) Ruppel, J. V.; Kamble, R. M.; Zhang, X. P. Cobalt-Catalyzed Intramolecular C–H Amination with Arylsulfonyl Azides. *Org. Lett.* **2007**, *9*, 4889–4892.

(3) Porcs-Makkay, M.; Lukács, G.; Pandur, A.; Simig, G.; Volk, B. Synthesis of 4-Unsubstituted 2H-1,2,3-Benzothiadiazine 1,1-Dioxides via Ortho Lithiation of Protected Benzaldehyde Derivatives. *Tetrahedron* **2014**, *70*, 286–293.

(4) Mohan, R.; Podgorski, D.; Krabbe, S.; Le, L.; Sierszulski, P. Bismuth Compounds in Organic Synthesis: Synthesis of Dioxanes, Dioxepines, and Dioxolanes Catalyzed by Bismuth(III) Triflate. *Synthesis* **2010**, *2010*, 2771–2775.

(5) Hwang, Y.; Baek, S. B.; Kim, D.; Chang, S. Chain Walking as a Strategy for Iridium-Catalyzed Migratory Amidation of Alkenyl Alcohols to Access α-Amino Ketones. *J. Am. Chem. Soc.* **2022**, *144*, 4277–4285.

(6) Lin, K.; Lu, H. DMAP Catalyzed One-Pot Curtius Rearrangement Using 1,1-Dimethyl-2,2,2-Trichloroethoxycarbonyl Azide. *Org. Lett.* **2023**, *25*, 4534–4539.

(7) Lee, J.; Lee, J.; Jung, H.; Kim, D.; Park, J.; Chang, S. Versatile Cp*Co(III)(LX) Catalyst System for Selective Intramolecular C–H Amidation Reactions. *J. Am. Chem. Soc.* **2020**, *142*, 12324–12332.

(8) Campbell-Verduyn, L. S.; Mirfeizi, L.; Dierckx, R. A.; Elsinga, P. H.; Feringa, B. L. Phosphoramidite Accelerated Copper(I)-Catalyzed [3+2] Cycloadditions of Azides and Alkynes. *Chem. Commun.* **2009**, *16*, 2139–2141.

(9) Keipour, H.; Jalba, A.; Delage-Laurin, L.; Ollevier, T. Copper-Catalyzed Carbenoid Insertion Reactions of α-Diazoesters and α-Diazoketones into Si–H and S–H Bonds. *J. Org. Chem.* **2017**, *82*, 3000–3010.

(10) Wakaki, T.; Sakai, K.; Enomoto, T.; Kondo, M.; Masaoka, S.; Oisaki, K.; Kanai, M. C(sp^3)–H Cyanation Promoted by Visible-Light Photoredox/Phosphate Hybrid Catalysis. *Chem. Eur. J.* **2018**, *24*, 8051–8055.

(11) Hindle, A.; Baj, K.; Iggo, J. A.; Cox, D. J.; Pask, C. M.; Nelson, A.; Marsden, S. P. Modular Synthesis of Bicyclic Twisted Amides and Anilines. *Chem. Commun.* **2023**, *59*, 6239–6242.

(12) Yamasaki, R.; Okada, Y.; Iizumi, H.; Ito, A.; Fukuda, K.; Okamoto, I. Structure and Additive-Free Transamidation of Planar *N*-Cyano Amides. *J. Org. Chem.* **2023**, *88*, 5704–5712.

(13) Zhang, Z.; Górski, B.; Leonori, D. Merging Halogen-Atom Transfer (XAT) and Copper Catalysis for the Modular Suzuki-Miyaura-Type Cross-Coupling of Alkyl Iodides and Organoborons. *J. Am. Chem. Soc.* **2022**, *144*, 1986–1992.

(14) Wang, J.; Qin, T.; Chen, T.-G.; Wimmer, L.; Edwards, J. T.; Cornella, J.; Vokits, B.; Shaw, S. A.; Baran, P. S. Nickel-Catalyzed Cross-Coupling of Redox-Active Esters with Boronic Acids. *Angew. Chem., Int. Ed.* **2016**, *55*, 9676–9679.

(15) Fuentes, J. A.; Carpenter, I.; Kann, N.; Clarke, M. L. Highly Enantioselective Hydrogenation and Transfer Hydrogenation of Cycloalkyl and Heterocyclic Ketones Catalysed by an Iridium Complex of a Tridentate Phosphine-Diamine Ligand. *Chem. Commun.* **2013**, *49*, 10245–10247.

(16) Radomkit, S.; White, J. A. H.; Chong, E.; Zhang, Y. MSTFA as an Effective TMS Source for the TMSOTf-Catalzyed Synthesis of Cyclic Acetals. *J. Org. Chem.* **2024**, *89*, 5555–5559.

(17) Luo, W.; Fang, Y.; Zhang, L.; Xu, T.; Liu, Y.; Li, Y.; Jin, X.; Bao, J.; Wu, X.; Zhang, Z. Bromomethyl Silicate: A Robust Methylene Transfer Reagent for Radical-polar Crossover Cyclopropanation of Alkenes: Bromomethyl Silicate: A Robust Methylene Transfer Reagent for Radical-Polar Crossover Cyclopropanation of Alkenes. *Eur. J. Org. Chem.* **2020**, *2020*, 1778–1781.

(18) Pulcinella, A.; Bonciolini, S.; Lukas, F.; Sorato, A.; Noël, T. Photocatalytic Alkylation of C(sp^3)–H Bonds Using Sulfonylhydrazones. *Angew. Chem., Int. Ed.* **2023**, *62*, e202215374.

(19) Bogdos, M. K.; Müller, P.; Morandi, B. Structural Evidence for Aromatic Heterocycle N–O Bond Activation via Oxidative Addition. *Organometallics* **2023**, *42*, 211–217.

(20) Wu, X.; Chen, W.; Holmberg-Douglas, N.; Bida, G. T.; Tu, X.; Ma, X.; Wu, Z.; Nicewicz, D. A.; Li, Z. ^{11}C, ^{12}C and ^{13}C-Cyanation of Electron-Rich Arenes via Organic Photoredox Catalysis. *Chem* **2023**, *9*, 343–362.

(21) Myers, E. L.; de Vries, J. G.; Aggarwal, V. K. Reactions of Iminium Ions with Michael Acceptors through a Morita-Baylis-Hillman-Type Reaction: Enantiocontrol and Applications in Synthesis. *Angew. Chem., Int. Ed.* **2007**, *46*, 1893–1896.

(22) Al-Sehemi, A. G.; Atkinson, R. S.; Fawcett, J. Kinetic Resolution of Amines with Enantiopure 3-*N,N*-Diacylaminoquinazolin-4(*3H*)-Ones. *J. Chem. Soc., Perkin Trans. 1* **2002**, *2*, 257–274.

(23) Honzawa, S.; Sugihara, T.; Uchida, M.; Tashiro, T. Alpha-Oxidation of Amine Derivatives by Bis(2,2,2-Trichloroethyl) Azodicarboxylate and Application of Its Products as Iminium Ion Equivalents. *Heterocycles* **2017**, *95*, 994.

(24) Rickertsen, D. R. L.; Crow, J. L.; Das, T.; Ghiviriga, I.; Hirschi, J. S.; Seidel, D. Acridine/Lewis Acid Complexes as Powerful Photocatalysts: A Combined Experimental and Mechanistic Study. *ACS Catal.* **2024**, *14*, 14574–14585.

(25) Vránová, I.; Alonso, M.; Lo, R.; Sedlák, R.; Jambor, R.; Růžička, A.; De Proft, F.; Hobza, P.; Dostál, L. From Dibismuthenes to Three- and Two-Coordinated Bismuthinidenes by Fine Ligand Tuning: Evidence for Aromatic BiC_3N Rings through a Combined Experimental and Theoretical Study. *Chem. Eur. J.* **2015**, *21*, 16917–16928.

(26) Moon, H. W.; Nöthling, N.; Leutzsch, M.; Kuziola, J.; Cornella, J. Characterization of Iminobismuthanes and Catalytic Reduction of Organic Azides via Bi(I)/Bi(III) Redox Cycling. *Angew. Chem., Int. Ed.* **2024**, e202417864.

(27) Mato, M.; Stamoulis, A.; Cleto Bruzzese, P.; Cornella, J. Activation and C–C Coupling of Aryl Iodides via Bismuth Photocatalysis. *Angew. Chem., Int. Ed.* **2025**, *64*, e202418367.

(28) Lee, W.; Kim, D.; Seo, S.; Chang, S. Photoinduced α-C–H Amination of Cyclic Amine Scaffolds Enabled by Polar-Radical Relay. *Angew. Chem., Int. Ed.* **2022**, *61*, e202202971.

(29) Geraci, A.; Baudoin, O. Fe-Catalyzed α-C(sp^3)–H Amination of N-Heterocycles. *Angew. Chem., Int. Ed.* **2024**, e202417414.

(30) Nyfeler, E.; Renaud, P. Decarboxylative Radical Azidation Using MPDOC and MMDOC Esters. *Org. Lett.* **2008**, *10*, 985–988.

(31) Magnus, P.; Hulme, C.; Weber, W. α-Azidonation of Amides, Carbamates, and Ureas with the Iodosylbenzene/Trimethylsilyl Azide Reagent Combination: *N*-Acyliminium Ion Precursors. *J. Am. Chem. Soc.* **1994**, *116*, 4501–4502.

(32) Walęcka-Kurczyk, A.; Adamek, J.; Walczak, K.; Michalak, M.; Październiok-Holewa, A. Non-Kolbe Electrolysis of *N*-Protected-α-Amino Acids: A Standardized Method for the Synthesis of N-Protected (1-Methoxyalkyl)Amines. *RSC Adv.* **2022**, *12*, 2107–2114.

(33) Takahata, H.; Kubota, M.; Momose, T. New Synthesis of All the Four Isomers of 2-(2-Hydroxypropyl)Pyrrolidines via Iterative Asymmetric Dihydroxylation to Cause Enantiomeric Enhancement. *Tetrahedron Asymmetry* **1997**, *8*, 2801–2810.

(34) Neese, F. The ORCA Program System. *Wiley Interdiscip. Rev. Comput. Mol. Sci.* **2012**, *2*, 73–78.

(35) Neese, F. Software Update: The ORCA Program System—Version 5.0. *Wiley Interdiscip. Rev. Comput. Mol. Sci.* **2022**, *12*.

(36) Becke, A. D. Density-Functional Exchange-Energy Approximation with Correct Asymptotic Behavior. *Phys. Rev. A Gen. Phys.* **1988**, *38*, 3098–3100.

(37) Weigend, F.; Ahlrichs, R. Balanced Basis Sets of Split Valence, Triple Zeta Valence and Quadruple Zeta Valence Quality for H to Rn: Design and Assessment of Accuracy. *Phys. Chem. Chem. Phys.* **2005**, *7*, 3297–3305.

(38) Pantazis, D. A.; Neese, F. All-Electron Scalar Relativistic Basis Sets for the 6p Elements. *Theor. Chem. Acc.* **2012**, *131*, 1292.

(39) Pantazis, D. A.; Chen, X.-Y.; Landis, C. R.; Neese, F. All-Electron Scalar Relativistic Basis Sets for Third-Row Transition Metal Atoms. *J. Chem. Theory Comput.* **2008**, *4*, 908–919.

(40) Pantazis, D. A.; Neese, F. All-Electron Scalar Relativistic Basis Sets for the Lanthanides. *J. Chem. Theory Comput.* **2009**, *5*, 2229–2238.

(41) Pantazis, D. A.; Neese, F. All-Electron Scalar Relativistic Basis Sets for the Actinides. *J. Chem. Theory Comput.* **2011**, *7*, 677–684.

(42) Grimme, S.; Ehrlich, S.; Goerigk, L. Effect of the Damping Function in Dispersion Corrected Density Functional Theory. *J. Comput. Chem.* **2011**, *32*, 1456–1465.

(43) Grimme, S.; Antony, J.; Ehrlich, S.; Krieg, H. A Consistent and Accurate Ab Initio Parametrization of Density Functional Dispersion Correction (DFT-D) for the 94 Elements H-Pu. *J. Chem. Phys.* **2010**, *132*, 154104..
